# Design, Synthesis and Antiplasmodial Activities of a Library of Fluorine-Based 3-Benzylmenadiones

**DOI:** 10.3390/molecules30112446

**Published:** 2025-06-03

**Authors:** Matthieu Roignant, Jimmy Richard, Maxime Donzel, Matthias Rottmann, Pascal Mäser, Elisabeth Davioud-Charvet

**Affiliations:** 1 Laboratoire d’Innovation Moléculaire et Applications (LIMA), Team Bio(IN)organic and Medicinal Chemistry, European School of Chemistry, Polymers and Materials (ECPM), UMR7042 Université de Strasbourg-CNRS-UHA, 25 rue Becquerel, F-67087 Strasbourg, France; roignant.matthieu@unistra.fr (M.R.); jrichards@unistra.fr (J.R.); mdonzel@unistra.fr (M.D.); 2 Swiss Tropical and Public Health Institute, Kreuzstrasse 2, CH-4123 Allschwil, Switzerland; matthias.rottmann@swisstph.ch (M.R.); pascal.maeser@swisstph.ch (P.M.); 3 University of Basel, Petersgraben 1, CH-4001 Basel, Switzerland

**Keywords:** antiplasmodial, benzylation, fluorine, menadione, plasmodione

## Abstract

Plasmodione is a potent early antiplasmodial compound. A metabolic study on mice treated with plasmodione revealed that 6-hydroxy–plasmodione was the main metabolite eliminated in the urine of treated mice. To block the metabolic pathway in the host, the introduction of fluorine at C-6 of the 3-benzylmenadione core was applied and showed potent antiplasmodial activity similar to that of the plasmodione analogue in vitro. In this work, a library of 38 6-fluoro-3-benzylmenadione analogues (*a* series) was constructed by incorporating structurally diverse groups in place of the 4-(trifluoromethyl) substituent present in the antiplasmodial plasmodione, via three synthetic routes. All new compounds were tested against the *P. falciparum* NF54 strain and for cytotoxicity with the rat L6 line. With a fluorine atom at C-6, **A-a-21** was revealed to be the only compound from the *a* series, superior to the 6-H- analogue from the *b* series, with an IC_50_ value of 70 nM versus 200 nM. Then, five other fluorine-based 3-benzylmenadiones, in which the fluorine was introduced in various positions of the 3-benzylmenadione core, were synthetized to assist our understanding of the impact of fluorine on antiplasmodial potencies in vitro; in particular, the aim here was to compare the effects of human serum and *P. berghei* species in these drug screens. This was also conducted in vivo with the *P. berghei*-infected mouse model. In the *P. berghei* species assay, PD and the 4′-fluoro-3′-trifluoromethyl-benzylmenadione **A-b-9** exhibited a similar antiplasmodial behavior toward *P. falciparum* versus *P. berghei.* In the human serum versus Albumax assays, only the 6-fluoro–plasmodione showed a lower shift factor between Albumax assays and human serum conditions, suggesting a lower protein binding for the 6-F-PD compared to plasmodione or **A-b-9**. In vivo, 6-fluoro–plasmodione proved to be the most potent 3-benzylmenadione, reducing parasitemia by 50% after oral administration at 50 mg/kg.

## 1. Introduction

With more than 600,000 deaths per year [1], malaria is the most devastating parasitic disease, disproportionally affecting the public health and economic welfare of the world’s poorest communities. The causative agents of malaria are the protozoa of the genus *Plasmodium*, with *P. falciparum* causing the most severe form of the disease, malaria tropica. The parasites are transmitted to humans by the bites of infected mosquitoes of the genus Anopheles. Once in the human body, they migrate to the liver, replicate inside hepatocytes and finally escape into the bloodstream where the erythrocytic cycle takes place and causes the typical symptoms of malaria. The majority of the currently available antimalarials and of the newly developed drug candidates target the asexual blood stages of *P. falciparum*, i.e., the stages that are responsible for pathology in humans. However, parasites are evolving resistance to antimalarial treatments, and there is an urgent need for new drugs. Even the efficacy of artemisinin-based combination therapies, the spearhead of malaria treatment, is threatened by drug resistance [2]. Thus, the development of novel antimalarials that can overcome artemisinin resistance is of the highest priority. It is noteworthy that many efforts have been made to render artemisinin derivatives, which are more metabolically resistant, through the introduction of fluorine [3]. The role and impact of the substitution of an H atom with fluorine have been widely exploited to improve drug potencies [4].

Plasmodione (PD) (3-[4-(trifluoromethyl)benzyl]-menadione) is an early antimalarial lead, which rapidly kills *P. falciparum* asexual blood stages, notably early rings but also young gametocytes [5]. While PD disrupts the clinically relevant intra-erythrocytic life cycle of the parasite, its low bioavailability is responsible for the weak activity in vivo in the *P. berghei* murine model following i.p. administration. We have shown that PD is a safe agent for possible human use, including in G6PD-deficient erythrocytes [6]. We further identified a synergy between PD and artemisinin (or with its main metabolite DHA) on artemisinin-susceptible parasites [5], highlighting the potential of redox-active 3-benzylmenadiones (bMDs) in antimalarial drug combinations. Indeed, we have shown that PD-treated mice eliminate the drug, mainly as a 6-hydroxy-PD metabolite in urine [7]. Thus, despite limited MedChem efforts, we have developed improved synthetic methodologies, supporting the further optimization of this family of compounds and allowing us to design a library of 6-fluoro-bMDs to counteract the host’s drug metabolism in the liver.

**Scheme 1:**
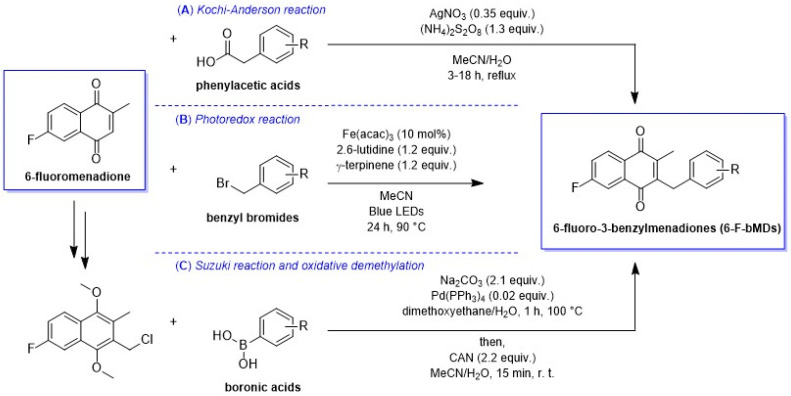
Synthetic pathways **A**-**B**-**C** to prepare the library of 6-fluoro-3-benzylmenadione (*a* compounds) and 6H-3-benzylmenadione (*b* compounds) derivatives.

The bMDs are currently synthesized via the well-known Kochi–Anderson reaction (Scheme 1) between the corresponding phenylacetic acid and menadione [8]. This reaction is very efficient with a high yield and good tolerance for diverse functional groups such as -NO_2_, -CF_3_ or various halides. However, the starting phenylacetic acid materials are not widely commercially available and must be used in excess, and heterocycles, i.e., alkyne-, alcohols- or amino-functionalized starting materials, are not compatible with this reaction.

In our team, we have developed a photoredox reaction generating a benzylic radical from benzyl bromides [9]. The reaction is applicable to more accessible starting materials than phenylacetic acids and has been applied to the synthesis of a large diversity of bMDs in one step (Scheme 1). In both Ag(II)-catalyzed [8] and photocatalyzed [9] benzylations, no obvious differences have been observed between products carrying EDG or EWG on these examples. Also, we established a new process to prepare 20 heteroaromatic analogues of PD [10] (Scheme 1). With these new methodologies in hand, the efforts of the present study aim to provide approaches for preparing more diverse 6-fluoro-bMDs and for screening the most potent antiplasmodial representatives in vitro and in vivo for rapid drug improvement.

## 2. Results

### 2.1. Synthesis

Previously, we reported a safe, economical and efficient multigram synthesis of 6-fluoromenadione [11]. The library of 6-fluoro-3-benzylmenadiones (*a* compounds) was obtained via three synthetic pathways, **A**-**B**-**C** (Scheme 1). The Kochi–Anderson reaction between 6-fluoromenadione and a phenylacetic acid was used to form 6-fluoro-3-benzylmenadiones (route **A**). Based on our previous work, the second strategy was a photoredox benzylation of quinones with a benzyl bromide (route **B**) [9]. The last synthetic pathway was a Suzuki reaction followed by an oxidative demethylation reaction (route **C**) [10]. In each route, some related 6-H-3-benzylmenadiones (*b* compounds) were also synthetized, both to compare biological activities and to complete our library [10,12].

#### 2.1.1. The Kochi–Anderson Reaction

The silver-catalyzed coupling between 6-fluoromenadione and commercially available mono-, bi- or tri-substituted phenylacetic acid led us to synthetize 27 new derivatives in addition to the known 6-fluoro–plasmodione (6-F-PD) (Table 1). Some related 6-H-3-benzylmenadiones were synthesized via the Kochi–Anderson reaction for comparison. The synthetized 6-H-3-benzylmenadiones, including PD, previously reported in a patent [13], are highlighted in grey in Table 1. Two more pairs of 6-fluoro- and 6-H- 3-benzylmenadione analogs were obtained from synthetized phenylacetic acid following the protocols presented in the literature (**A-a-23** [14], **A-a-24** [15]).

**Scheme 2:**
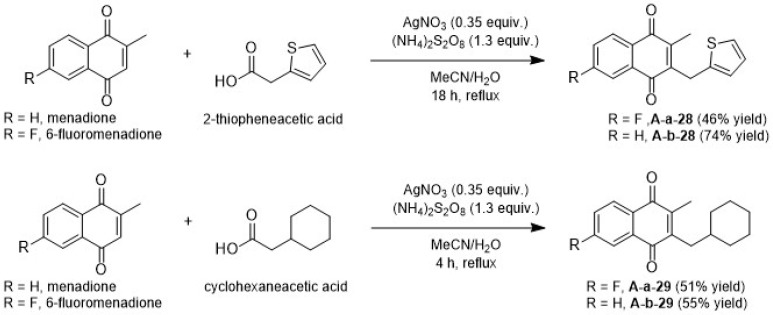
Synthesis of 6-fluoro- and 6-H-3-(thiophen-2-ylmethyl)-menadione **A-a/b-28** and 6-F- and -(cyclohexylmethyl)-menadione derivatives **A-a/b-29**.

Furthermore, the radical decarboxylation reaction was also performed with commercial 2-thiopheneacetic acid and 6-fluoromenadione or menadione, respectively, to form corresponding analogs **A-a-28** and **A-b-28**, allowing us to investigate the essential requirement of a phenyl ring in the benzylic chain (Scheme 2). The pair of 6-F- and 6-H- 3-cyclohexyl-menadiones **A-a-29** and **A-b-29** (Scheme 2) was also synthetized from cyclohexaneacetic acid as controls.

#### 2.1.2. The Photoredox Reaction

The 6-fluoro-3-benzylmenadiones were also synthesized by a blue-light-induced process in the presence of 2,6-lutidine, Fe(acac)_3_, γ-terpinene and atmospheric oxygen (Table 2) [9]. Due to the electronegativity of fluorine atom, yields were generally weaker with the 6-fluoromenadione than with menadione.

#### 2.1.3. The Synthetic Route via the Suzuki Coupling C

**Scheme 3:**
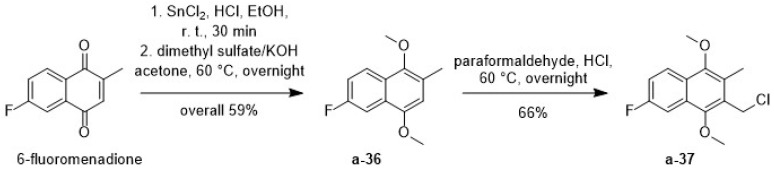
Protection of 6-fluoromenadione as 1,4-dimethoxy-2-methyl-naphthalene followed by chloromethylation.

A series of 6-fluoro-3-benzylmenadione derivatives was synthetized by Suzuki reaction according to the protocol presented in the literature [16]. The starting 6-fluoromenadione was first reduced and the dihydronaphthoquinone was protected to tolerate the basic conditions of the Suzuki reaction (Scheme 3). The first step was the protection of the naphthoquinone as dimethoxynaphthalene **a-36** with a 59% yield. Then, the chloromethylation was performed with paraformaldehyde in the presence of HCl to form the methyl chloride derivative **a-37**.

**Scheme 4:**
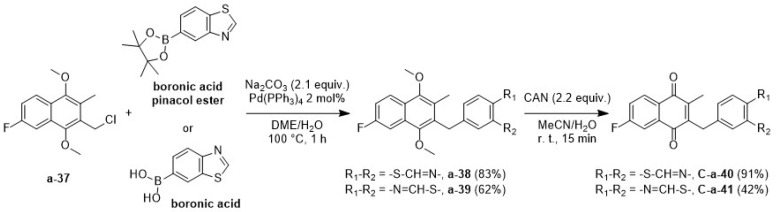
Synthesis of 6-fluoro-3-benzylmenadiones **C-a-40** and **C-a-41** via the Suzuki coupling and demethylation approach (route **C**).

The Suzuki reaction was performed between **a-37** and boronic acid pinacol ester or boronic acid to obtain protected 6-fluoro-1,4-dimethoxy-2-methyl-naphthalenes **a-38** and **a-39** with a good yield (Scheme 4). Oxidative demethylation of 1,4-dimethoxy-naphthalenes in the presence of cerium ammonium nitrate enabled the formation of **C-a-40** (91%) and **C-a-41** (42%).

**Scheme 5:**
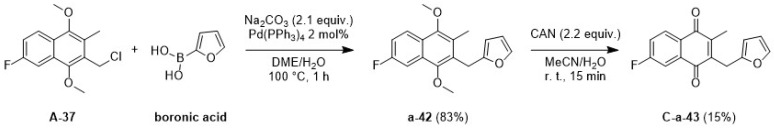
Synthesis of the furanyl derivative **C-a-43** via the Suzuki coupling and oxidative demethylation approach.

A furanyl derivative was also synthetized following this strategy (Scheme 5). Protected intermediate **a-42** was obtained with a 83% yield. Deprotection of methyl groups led to **C-a-43** with a low yield (15%).

**Scheme 6:**
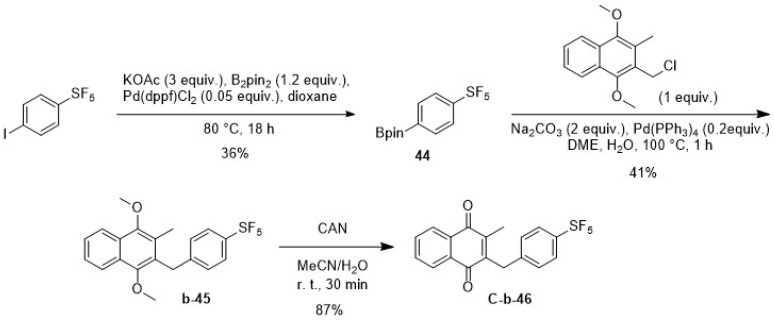
Synthesis of the 4′-SF_5_-substituted 3-benzylmenadione **C-b-46** via route **C**.

Finally, the 4′-SF_5_-substituted 3-benzylmenadione was produced for comparison with antiplasmodial potency of PD and fluoro-based analogs. Its preparation was performed by using the starting 6-H-1,4-dimethoxynaphthalene 2-methyl-3-methyl chloride [10] (Scheme 6). A *Miyaura borylation* reaction was carried out to produce the boronate by cross-coupling of bis(pinacolato)diboron (B_2_pin_2_). The *borylation* reaction could be achieved with the starting p-SF_5_-aryl iodide at 80 °C in 18 h with the low yield of 36%. Once the Bpin ester **44** was prepared, the Suzuki coupling approach was then applied at 100 °C for one hour to generate the benzylated 1,4-dimethoxy-naphthalene **b-45** with a 41% yield. Then, the oxidative demethylation process enabled the production of the final 4′-SF_5_-substituted 3-benzylmenadione **C-b-46** with a 87% yield.

### 2.2. Physicochemical Properties

To optimize the efficacy of bMDs against plasmodial parasites and predict the suitability of the compounds for oral drug administration, it is important to characterize the pharmacological profile of the final compounds. By introducing a fluorine atom at C-6, and varying the functional groups on the benzyl chain, we first characterize several key physicochemical parameters of the selected 6*H*-bMDs, presented in Table 3, including their molecular weight (MW), number of fluorine atoms, their predicted n-octanol/water partition coefficient (cLog P), and calculated total polar surface area (tPSA). In addition, Table 3 shows their experimental thermodynamic aqueous solubility, determined in PBS-10% DMSO (in μM), and their lipophilicity indexes (CHI: Chromatographic Hydrophobicity Index). The lead antimalarial plasmodione, PD, has low aqueous solubility and relatively high lipophilicity.

Adding fluorine increases the lipophilicity of 6-F-PD and decreases its aqueous solubility, as expected. However, moving the fluorine at C-7 (7-F-PD) or at C-4′ with the -CF_3_ group at C-3′ (**A-b-9**) confers a higher aqueous solubility than it does for PD. The lead antimalarial plasmodione, PD (cLogP: 5.4; tPSA: 34.14), has low aqueous solubility (0.44 µM) and a relatively high lipophilicity index (115). Despite improvements in the physicochemical properties of **A-b-10** (cLogP: 3.9; tPSA: 57.93) and **A-b-12** (cLogP: 4.2; tPSA: 85.95), there is no correlation with their ability to kill the *P. falciparum* parasite (IC_50_ > 200 nM). Compounds with poor aqueous solubility and high lipophilicity are the most likely ones to be the most potent against *P. falciparum.* The relationship between physicochemical properties and antiplasmodial activity may involve other factors rather than just tPSA, lipophilicity and aqueous solubility.

### 2.3. Biological Activities

#### 2.3.1. In Vitro Antiplasmodial Activities

Considering the potency of plasmodione and the observed main metabolite 6-hydroxy–plasmodione eliminated in the urine of PD-treated mice [7], our primary goal was to introduce a fluorine atom at C-6 of the menadione core to block the process of drug metabolism from occurring in the host’s liver. In this aim, we prioritized the preparation of a library of 6-fluoro-3-benzylmenadiones by investigating the replacement of the trifluoromethyl group by diverse EWG and EDG groups in various loci of the aryl ring. For SAR, all compounds were tested for the inhibition of parasite growth in RBCs infected with a *P. falciparum* chloroquine-sensitive strain, NF54. The parasites were cultured in vitro according to known conditions [17]. IC_50_ values were determined by measuring the incorporation of the nucleic acid precursor [^3^H]hypoxanthine after 72 h of incubation [18]. The cytotoxicity of the active compounds was assessed against a rat myoblast cell line (L6 cells). Table 4 shows a summary of the investigations performed to introduce diverse substitution patterns after the introduction of a fluorine atom at C-6.

The four compounds, **C-a-43**, **A-b-43**, **A-a-28** and **A-b-28v,** and both compounds, **A-a-29** and **A-b-29** (Table 4), containing a 2-unsubstituted furan, a thiophene ring, or a cyclohexane, respectively, as phenyl replacements, were evaluated; however, none of these could compete with the potent antiplasmodial activities of PD or 6-fluoro-PD.

With a fluorine atom at C-6 in the menadione core, the absolute antiparasitic activities were rather disappointing in vitro. This result can be explained by the low aqueous thermodynamic solubility of 6-fluoro-PD compared to that of PD in PBS—10% DMSO: 0.06 ± 0.01 µM versus 0.44 ± 0.05 µM—due to the increase in lipophilicity that was induced by adding a fluorine atom (Table 3). When examining the 30 pairs of 6-fluoro-/6-H- 3-benzylmenadiones from Table 4, except for six pairs—**A-a-6**/**A-b-6**, **A-a-10**/**A-b-10**, **A-a-11**/**A-b-11**, **A-a-12**/**A-b-12**, **A-a-21**/**A-b-21**, **B-a-31**/**B-b-31**—the antiplasmodial activity of the 6-fluoro-3-benzylmenadione was always lower than that of the 6-H- analogue. Moreover, in the case where we did not have a 6-H-analogue for the comparison, the IC_50_ values of the 6-fluoro-3-benzylmenadiones were too high to be considered in the evaluation of the antiplasmodial activity in the *P. berghei*-infected mouse model.

This means that increasing the lipophilicity of the final molecule by adding a fluorine atom at C-6 must be compensated for through appropriate structural diversity at the benzylic chain, causing it to reach the ideal pharmacokinetic parameters—such as aqueous solubility (Table 3)—for effective antiplasmodial activity per os. In this set of compounds, we did not succeed in obtaining a 6-fluoro-benzylmenadione; the exception to this was **A-a-1**, which showed more potent in vitro activity than plasmodione. It is noteworthy that 6-F-PD was found to be as potent as PD—IC_50_ of 59 ± 11 nM versus 58 ± 11 nM for PD in antiplasmodial assays when using the multi-resistant *P. falciparum* strain Dd2 [12]; this justifies the selection of 6-F-PD in the in vivo tests in the *P. berghei*-infected mouse model.

In Table 4, it can be seen that the most active 3-benzylmenadiones with IC_50_ values below 100 nM against *P. falciparum* NF54 are as follows: 6-F- derivatives **A-a-1** and **A-a-21**; 6-H- derivatives (from the most potent to the less potent) PD [8], **B-b-1** [9], **B-b-2** [9], **A-b-3**, **A-b-4** [13], **B-b-5**, **A-b-8**, **A-b-9**, **A-b-14**, **A-b-15**, **A-b-24**, **B-b-35** [9], **B-b-37**, **C-b-49** and **A-b-47** [9]. With a C-2′-CF_3_ group (*ortho* instead of *para*, such as in PD) and a fluorine atom at C-4′, the compound—recently produced and tested against *P. falciparum* NF54—exhibited an IC_50_ of 20 nM [19] versus 26 nM for **A-a-1** (with a C-4′-CF_3_ group (*para*, such as in PD) and a fluorine atom at C-6). With a fluorine atom at C-6, **A-a-21** is the only compound from the *a* series, superior to the 6-H-analogue from the *b* series, with improved antiplasmodial potency (70 nM versus 200 nM). We will investigate its antiplasmodial profile with various strains and determine how we can exploit this observed potency in the future.

Because the number (3 to 5) and the locus of fluorine atoms on the 3-benzylmenadione core seem to be factors that govern the pharmacokinetics of the final molecules, we selected five fluorine-based 3-benzylmenadiones to investigate the effects of the process on antiplasmodial activity when using *P. falciparum* versus *P. berghei* (species shift assay); we will also investigate a case using the *P. falciparum* strain NF54 (72 h assay) in the presence of 0.5% Albumax versus 50% human serum (serum shift assay).

#### 2.3.2. In Vitro Species and Serum Shift Assays

Because the evaluation of antiplasmodial drugs in vivo is performed in the *P. berghei*-infected mouse model, we made sure beforehand that we had evaluated the compounds in in vitro drug assays in the presence of *P. falciparum* or *P. berghei*, using a parasitic cycle period of 24 h (Table 5A). We selected 5 key compounds, including plasmodione, among the 40 newly synthetized 6-fluoro-3-benzylmenadiones from Table 4 and Table 5; these were screened in the drug assays in the presence of the *P. falciparum* NF54 strain (72 h parasitic cycle) or the *P. berghei* strain (24 h parasitic cycle).

The objective was to test the compounds with a species shift of ≥1.0 to perform an accurate evaluation of the antiplasmodial activity of the compounds (Table 5A, green line). Under these conditions, only PD and the 4′-fluoro-3′-trifluoromethyl-benzylmenadione **A-b-9** exhibited similar antiplasmodial behavior toward *P. falciparum* versus *P. berghei.* We also considered the serum conditions to be close to the conditions found in the *P. berghei*-infected mouse model to identify compounds that lose in vitro potency in the presence of 50% human serum, as this could be an indication of high protein binding (Table 5B).

In general, the five key compounds including plasmodione revealed significant antiplasmodial activity under the NF54 0.5% Albumax assay conditions, but lower activity was found in the NF54 50% human serum assay, except for the most potent compound 6-F-PD with a shift of 0.9 (Table 5B, green line). Compared to the positive control artesunate (AS), exhibiting a shift of 0.6, the serum shifts were observed to extend in the range 4.0 to 6.1. As discussed earlier [20], these data guide us to select compounds that show low shift factors between the Albumax versus human serum conditions; this enables us to test for the in vivo antiplasmodial activities of key compounds in the *P. berghei* murine model. Among the 38 newly synthetized 6-fluoro-3-benzylmenadiones from Table 4 and Table 5 screened in the drug assays with the NF54, only 6-F-PD showed a low serum shift combined to a potent antiplasmodial activity in the same range as PD; this was also reported [12] in a case against the multi-resistant Dd2 strain, showing moderate toxicity in the rat L6 strain assay.

#### 2.3.3. In Vivo Antiplasmodial Activities

With a limited number of compounds, we assessed their antiplasmodial effects in vivo using the *P. berghei*-infected mouse malaria model. Using this test in vivo, both antiplasmodial activity and survival time can be analysed (Table 6). Compounds were administered 4, 24, 48 and 72 h post-infection. Samples were collected on day 4 (96 h post-infection). Antiplasmodial activity was calculated as the difference between the mean percent parasitemia for the control (n = 4 mice) and treated groups (n = 3 mice); these findings are expressed as a percentage, relative to the control group. Control mice were euthanized on day 4 to prevent death typically occurring on day 6. Among the five tested compounds, **6-F-PD** was found to display the most effective bMD, decreasing parasitemia in vivo (Table 6, green line). Compound **6-F-PD** has the lowest aqueous solubility, but this is offset by the lowest capacity to bind to human serum proteins in the serum shift assay, making it the most effective in vivo compound from a series of five differently fluorine-functionalized 3-benzylmenadiones.

Also, as can be seen, the results of a fluorine scan at C-7, C-3′ or C-4′, around the phenyl ring of menadione core or the benzyl chain, and -CF_3_ displacement at C-4′ (*para* substitution) or at C-3′ (*meta* substitution), are summarized here; they indicate that there is no advantage to the additional substituents in the bMD skeleton.

## 3. Discussion and Conclusions

In this work, our aim was to introduce a fluorine atom at C-6 of 3-benzylmenadiones to block the process of drug metabolism in the host in vivo. Compounds with an IC_50_ against *P. falciparum* < 100 nM and selectivity > 100 as compared to the mammalian cell line, were assessed. The study identified 6-F-PD as the best candidate, based on (i) similar antiplasmodial potencies against both chloroquine-sensitive (NF54) and multi-resistant (Dd2) strains, (ii) a species shift parameter ≥ 1 as reporter of a more potent activity towards *P. falciparum* versus *P. berghei*, in addition to (iii) low serum protein binding expressed as a serum shift parameter of ≤1.0. These findings will be further analyzed with other drug combinations. The next challenge will be to continue our efforts to introduce structural diversity and improve the 6-fluoro-bMD-based scaffold activity in vivo by incorporating groups that are known to improve pharmacokinetics in vivo following per os administration.

## 4. Materials and Methods

### 4.1. General Information

The starting materials and reagents were obtained from Sigma-Aldrich, ABCR GmbH & Co., Alfa Aesar, BLD Pharm, Fluorochem and Apollo Scientific and were used without further purification. The solvents were obtained from Carlo Erba, VWR and Fisher Scientific. All reactions were performed in standard glassware. Thin-layer chromatography (TLC) was performed using Merck silica gel plates (60 F-254, 0.25 mm) on aluminum sheets and using a UV lamp (325 and 254 nm). The crude mixtures were purified using flash chromatography on silica gel 60 (230–400 mesh, 0.040–0.063 mm) purchased from VWR.

The NMR spectra were recorded on a Bruker Avance 400 apparatus (^1^H NMR, 400 MHz—^13^C NMR, 101 MHz—^19^F NMR, 377 MHz) or a Bruker Avance III HD 500 MHz apparatus (^1^H NMR, 500 MHz—^13^C NMR, 126 MHz—^19^F NMR, 471 MHz) at the ECPM. All chemical shifts refer to the partial deuterated NMR solvent used here (CDCl_3_: ^1^H NMR, 7.26 ppm and ^13^C NMR, 77.16 ppm—CD_3_CN: ^1^H NMR, 1.94 ppm and ^13^C NMR, 1.32 and 118.26 ppm). The coupling constants (J) are given in Hertz (Hz). The resonance patterns are reported with the following notations: br (broad), s (singlet), d (doublet), t (triplet), q (quartet), m (multiplet) and dd (doublet of doublets).

High-resolution mass spectrometry (HRMS) analyses were performed with a Bruker MicroTOF mass analyzer under ESI in the positive or negative ionization detection mode or under APCI in the positive ionization detection mode (measurement accuracy ≤15 ppm). These analyses were performed at the Service de Spectrométrie de Masse Fédération Chimie Le Bel in Strasbourg.

The melting points were measured on a Stuart SMP10 with a temperature accuracy of ±1.0 °C at 20 °C and ±2.5 °C at 300 °C.

### 4.2. General Procedure

#### 4.2.1. General Procedure A: Synthesis of 6-R-3-Benzylmenadiones Using the Kochi–Anderson Reaction

A solution of 6-fluoro-menadione or menadione (100 mg, 1.0 equiv.) and phenylacetic acid (1.0–2.0 equiv.) in a mixture of acetonitrile and water (0.05 M, ratio 3/1, *v*/*v*) was stirred at 80 °C. After cooling at room temperature, AgNO_3_ (0.35 equiv.) was added; this was followed by ammonium persulfate (1.3 equiv.). The yellow mixture reaction, protected from light, was stirred at reflux for 3–18 h (Monitoring by TLC). After this time, the mixture reaction was cooled at room temperature and acetonitrile was evaporated under vacuum. The aqueous phase was extracted four times with dichloromethane. The organic layer was washed twice with a saturated aqueous solution of NaHCO_3_, dried over MgSO_4_ and concentrated under reduced pressure. The crude residue was purified by flash chromatography on silica gel to obtain the desired product.

#### 4.2.2. General Procedure B: Synthesis of 6-R-3-Benzylmenadiones Using a Photoredox Reaction

In accordance with a previous report [9] a solution of 6-fluoro-menadione or menadione (100 mg, 1.0 equiv.), benzyl bromide (1.5 eq) and Fe(acac)_3_ (0.1 equiv.) in acetonitrile (0.1 M) was placed in a 10 mL sealable tube; to this was added 2.6-lutidine (1.2 equiv.) and γ-terpinene (1.2 equiv.). The tube was sealed, put under blue light irradiation and stirred at 90 °C for 24 h. After completion, the mixture reaction was cooled at room temperature and was partitioned between ethyl acetate and a 1 M aqueous solution of HCl. The aqueous layer was extracted once with ethyl acetate. The organic extracts were washed once with brine, dried over MgSO_4_ and concentrated under reduced pressure. The crude residue was purified by flash chromatography on silica gel to obtain the desired product.

#### 4.2.3. General Procedure C: Synthesis of 6-Fluoro-3-benzylmenadiones Derivatives Using the Suzuki Reaction

In a flame-dried sealable tube under argon, 2-(chloromethyl)-6-fluoro-1,4-dimethoxy-2-methylnaphthalene (100 mg, 1.0 equiv.), boronic acid (1.2 equiv.) and sodium carbonate (2.1 equiv.) were introduced successively to a mixture of dimethoxyethane and water (0.17 M, ratio 2/1, *v*/*v*). The solvent was degassed and tetrakis(triphenylphosphine)palladium (0.02 equiv.) was added to the solution. The tube was sealed and stirred at 100 °C for 1 h. The reaction mixture was diluted with water and extracted three times with dichloromethane. The organic layer was washed with brine, dried over MgSO_4_ and concentrated under reduced pressure. The crude residue was purified by flash chromatography on silica gel to obtain the expected product.

#### 4.2.4. General Procedure D: Demethylation Reaction

A solution of ceric ammonium nitrate (2.2 equiv.) in water (0.5 M) was added dropwise to a solution of freshly obtained product (1.0 equiv.) in acetonitrile (0.08 M). The solution was stirred at room temperature for 15 min. The reaction mixture was diluted with water and extracted twice with dichloromethane. The organic layer was washed with brine, dried over MgSO_4_ and concentrated under reduced pressure. The crude residue was purified by flash chromatography on silica gel to obtain the desired product.

### 4.3. Pharmacokinetics

*Thermodynamic Aqueous Solubility*: The shake-flask method enabled us to determine the apparent thermodynamic solubilities of the DMSO stock solutions of the compounds. The compound dilutions were prepared from the 200 μM stock solution in pure DMSO to the buffer PBS at pH 7.4, containing 90% PBS: 137 mM NaCl, 2.7 mM KCl,1.4 mM KH_2_PO_4_, 4.3 mM Na_2_HPO_4_ and 10% DMSO final. The mixture was equilibrated at 20 °C for 24 h. After centrifugation, the supernatant was injected into an HPLC column coupled to an LC-MS apparatus (with UV–Vis detection) in order to determine the compound concentration. A reference solution at 200 μM was made in CH_3_CN/water and a dilution at 100 μM enabled us to conduct an analysis of the linearity of the UV response. These solutions were also injected in order to calibrate the concentration determination process. Comparison of the responses of the injections of three dilutions obtained from a DMSO reference solution enabled us to determine solubility, if lower than 200 μM. Measurements were performed in duplicate.

*Determination of CHI values*: Compound lipophilicity influences permeability and propensity to nonspecific binding to plasma proteins. Although precise, the classical “shake flask” method is not always appropriate for the determination of high hydrophobicity, which can be alternatively measured using the Chromatographic Hydrophobicity Index method. This HPLC-based technique leads to rapid and cheap estimates of partition coefficients. Lipophilicity is determined through the partition of the compound between a hydro-organic mobile phase and a reverse stationary phase, typically a C18 column, using a fast gradient. Compound solution samples (200 μM) were prepared in a 50/50 *v/v* mixture of water and acetonitrile. A measure of 5 μL was injected onto the HPLC (with UV–Vis detection). CHI values were derived from the retention time and a comparison of this value with the 10 reference compounds of the known CHI. The measurements were performed in duplicate. In general, CHI values correlate satisfactorily with logD_7.4_ values. The CHI is approximately equal to the proportion of acetonitrile in the mobile phase when the compound is eluted from the column.

### 4.4. Parasite Culture and Antiplasmodial Drug Assays

The compounds were tested for their inhibition of parasite growth in RBCs infected with the chloroquine-sensitive *P. falciparum* parasite strain NF54. *P. falciparum* NF54 wild-type parasites cultured in a medium containing 0.5% Albumax II were used to test for compound activity on parasite multiplication using a [^3^H]-hypoxanthine incorporation assay, as recently described by the STPH authors M. R. and P. M. in their recently published article [21].

### 4.5. In Vitro Serum Shift Assay

The parasites were cultured in vitro according to ref. [17]. The IC_50_ values were determined by measuring the incorporation of the nucleic acid precursor [^3^H]hypoxanthine after 72 h of incubation, as described in [18]. The IC_50_ values were either determined in the presence of 0.5 % Albumax (a serum substitute corresponding to a final assay concentration of 10 % bovine serum albumin) or 50 % human serum (human serum, off the clot, delipidized, C11-064 Braunschwig, Switzerland). The serum shift protocol with the *P. falciparum* strain NF54 (72 h assay), comparing 0.5% Albumax with 50% human serum, has been described previously in [20]. For in vitro efficacy testing with *P. berghei* (GFP ANKA malaria strain donation from A. P. Waters and C. J. Janse, Leiden University), heparinized blood with parasites was taken from donor mice, washed with medium and diluted with hypoxanthine-free culture medium and red blood cells (RBCs) from uninfected mice to be used in the assay, as described above (with shortened assay duration). The parasites were incubated for 16 h, and 0.25 µCi of [^3^H]hypoxanthine was added per well for an additional 8 h before harvesting.

### 4.6. Cytotoxicity Assays with the Rat L6 Cell Line

Cell proliferation was assessed with resazurin, and the generally cytotoxic agent podophyllotoxin served as the positive control, as previously reported by the STPH authors M. R. and P. M. in their recently published article [19].

### 4.7. Drug Assay In Vivo in P. berghei-Infected Mice

The selected compounds were tested in the murine *P. berghei* model, essentially as described in [22,23,24]. The infection was initiated at day 0 with the *P. berghei* GFP ANKA malaria strain (donation from A. P. Waters and C. J. Janse, Leiden University). From the donor mice with approximately 30% parasitemia, heparinized blood was taken and diluted in physiological saline to 10^8^ parasitized erythrocytes/mL. An aliquot (0.2 mL) of this suspension was injected intravenously into experimental and control groups of mice. Usually, in untreated control mice, parasitemia rises regularly to ~30% by day 3 post-infection. In these conditions, control mice die between days 6 and 7 post-infection. In the experiments described here, however, the control animals (n = 5) were sacrificed on day 4 post-infection for ethical reasons.

The compounds were prepared to appropriate concentrations as solution/suspensions in Tween 80/alcohol (7:3, Tween 80 and absolute ethanol, respectively), followed by 10× dilution in water. They were administered to the groups of three mice in four doses (at 4 h, 24 h, 48 h and 72 h post-infection). The route of administration was per os (p.o., 50 mg/kg).

The degree of infection (parasitemia expressed in % of infected erythrocytes) was determined through a FACS analysis on day 4 (96 h post-infection). The difference of the mean infection rate of the control group (=100%) to the test group was calculated and expressed as a percentage reduction. For example, activity determination with a mean of 2% parasitemia in treated mice and a mean of 40% parasitemia in the control animals was calculated as follows: (40–2%)/40% × 100 = 95% activity. The survival time in days was recorded up to 30 days after infection. A compound was considered to be curative if the animal survived to day 30 after infection with no detectable parasites (confirmed using light microscopy).

## 5. Patents

Three patents were filled for the synthetic routes of preparing some of the compounds described in this manuscript.

Donzel M., Elhabiri M., Davioud-Charvet E. Photoredox radical benzylation process. EP N° 21305263.2 (5 March 2021). PCT No. PCT/EP2022/055568, EP 21305263.2 (4 March 2022). WO2022184904 A1 (9 September 2022).

Trometer N., Donzel M., Roignant M., Davioud-Charvet E. 6-Substituted menadiones and their preparation. EP N° 21305973.6 (13 July 2021). PCT No. EP2022069555 (13 July 2022). WO2023285509 A1 (19 January 2023).

Donzel M., Roignant M., Elhabiri M., Davioud-Charvet E. Preparation of heteroaromatic analogues of 3-benzylmenadione derivatives. EP N° 21305755.7 (4 June 2021). PCT No. EP4347566A1 (4 June 2022). WO2022254034 A1 (8 December 2022).

The older patent on the antimalarial applications of 3-benzylmenadione analogues is given under reference number [13].

## Figures and Tables

**Table 1:** The library of 6-R-3-benzylmenadione derivatives (**A-a-1** to **A-a-27**, R = F and **A-b-1** to **A-b-27**, R = H) synthetized via the Kochi–Anderson reaction **A**. The grey lines in the Table indicate that the synthesis and the antiplasmodial activity of the compounds were described in a patent [13] or in references [8,12].

** 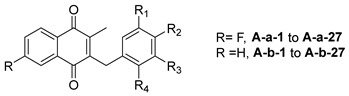 **						
Cpnd	R	R1	R2	R3	R4	Yield (%)
6-F-PD [12]	F	H	CF_3_	H	H	55
PD [8]	H	H	CF_3_	H	H	80
**A-a-1**	F	H	H	CF_3_	H	44
**A-a-2**	F	H	H	H	CF_3_	38
**A-a-3**	F	H	OCF_3_	H	H	56
**A-b-3 [13]**	H	H	OCF_3_	H	H	78
**A-a-4**	F	H	F	H	H	77
**A-b-4 [13]**	H	H	F	H	H	66
**A-a-5**	F	H	H	F	H	34
**A-a-6**	F	H	H	H	F	45
**A-b-6**	H	H	H	H	F	67
**A-a-7**	F	H	CF_3_	F	H	75
**A-b-7**	H	H	CF_3_	F	H	82
**A-a-8**	F	H	CF_3_	Br	H	64
**A-b-8**	H	H	CF_3_	Br	H	84
**A-a-9**	F	H	F	CF_3_	H	52
**A-b-9**	H	H	F	CF_3_	H	92
**A-a-10**	F	H	CN	H	H	75
**A-b-10 [8]**	H	H	CN	H	H	63
**A-a-11**	F	H	H	H	CN	48
**A-b-11**	H	H	H	H	CN	56
**A-a-12**	F	H	NO_2_	H	H	72
**A-b-12 [8]**	H	H	NO_2_	H	H	89
**A-a-13**	F	H	I	H	H	85
**A-a-14**	F	H	Cl	H	Cl	59
**A-b-14**	H	H	Cl	H	Cl	59
**A-a-15**	F	H	Cl	H	CF_3_	46
**A-b-15**	H	H	Cl	H	CF_3_	53
**A-a-16**	F	H	OMe	H	H	79
**A-b-16 [13]**	H	H	OMe	H	H	45
**A-a-17**	F	H	H	OMe	H	59
**A-b-17**	H	H	H	OMe	H	75
**A-a-18**	F	H	H	H	OMe	69
**A-b-18 [13]**	H	H	H	H	OMe	82
**A-a-19**	F	H	OMe	OMe	H	52
**A-b-19 [13]**	H	H	OMe	OMe	H	94
**A-a-20**	F	H	OMe	H	OMe	71
**A-b-20 [13]**	H	H	OMe	H	OMe	37
**A-a-21**	F	H	-O-CH_2_-O-		H	66
**A-b-21**	H	H	-O-CH_2_-O-		H	60
**A-a-22**	F	H	-O-(CH_2_)_2_-O-		H	79
**A-b-22**	H	H	-O-(CH_2_)_2_-O-		H	91
**A-a-23**	F	H	-O-CMe_2_-O-		H	83
**A-b-23**	H	H	-O-CMe_2_-O-		H	85
**A-a-24**	F	H	-O-CF_2_-O-		H	78
**A-b-24**	H	H	-O-CF_2_-O-		H	73
**A-a-25**	F	H	OMe	OMe	OMe	80
**A-b-25 [13]**	H	H	OMe	OMe	OMe	76
**A-a-26**	F	OMe	H	H	OMe	63
**A-b-26 [13]**	H	OMe	H	H	OMe	80
**A-a-27**	F	OMe	OMe	OMe	H	85
**A-b-27 [13]**	H	OMe	OMe	OMe	H	85

**Table 2:** The library of 6-R-3-benzylmenadione derivatives (**B-a-30** to **B-a-35**, R = F and **B-b-1** to **B-b-35**, R = H) synthetized via the photoredox reaction **B**. The grey lines in Table indicate that the synthesis and the antiplasmodial activity of the compounds were described in reference [9].

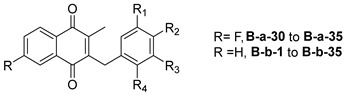						
Cpnd	R	R1	R2	R3	R4	Yield (%)
6-F-PD [9]	F	H	CF_3_	H	H	67
**B-b-1 [9]**	H	H	H	CF_3_	H	76
**B-b-2 [9]**	H	H	H	H	CF_3_	77
**B-b-3 [9]**	H	H	OCF_3_	H	H	80
**B-b-4 [9]**	H	H	F	H	H	66
**B-b-5**	H	H	H	F	H	60
**B-b-26 [9]**	H	OMe	H	H	OMe	78
**B-a-30**	F	H	H	CN	H	43
**B-b-30**	H	H	H	CN	H	66
**B-a-31**	F	H	H	H	H	67
**B-a-32**	F	H	NO_2_	F	H	36
**B-a-33**	F	H	F	I	H	36
**B-b-33**	H	H	F	I	H	56
**B-a-34**	F	H	Br	H	CF_3_	22
**B-b-34**	H	H	Br	H	CF_3_	69
**B-a-35**	F	OMe	H	OMe	H	62
**B-b-35 [9]**	H	OMe	H	OMe	H	68

**Table 3:** Pharmacokinetic parameters of plasmodione, 6-fluoro–plasmodione and selected 3-(substituted-)benzylmenadiones. The CHIs and thermodynamic aqueous solubilities in PBS-10% DMSO are experimentally measured values; ^a^ meanwhile, the MW, cLogP and tPSA values are calculated in ChemDraw 21.0.0.28.

Cpnd	MW ^a^ (g·mol^−1^)	cLogP^a^	tPSA ^a^ (Å^2^)	CHI	log D_7.4_	n Fluorine	Thermodynamic aq. Solubility in PBS-10% DMSO (µM)
PD	330.31	5.416	34.14	115	4.68	3	0.44 ± 0.05
6-F-PD	348.30	5.594	34.14	117	4.75	4	0.06 ± 0.01
7-F-PD	348.30	5.594	34.14	nd	nd	4	0.59 ± 0.01
**A-b-3**	346.31	5.561	43.37	117	4.80	3	0.16 ± 0.04
**A-b-4**	280.30	4.676	34.14	108	4.30	1	1.33 ± 0.02
**A-b-9**	348.30	5.559	34.14	nd	nd	4	0.67 ± 0.01
**A-b-10**	287.32	3.966	57.93	97	3.75	0	0.82 ± 0.01
**A-b-12**	307.30	4.276	85.95	112	3.99	0	0.24 ± 0.01
**A-b-16**	292.33	4.452	43.37	106	4.22	0	1.07 ± 0.02
**A-b-26**	322.36	4.541	52.6	107	4.26	0	0.59 ± 0.01
**B-b-35**	322.36	4.541	52.6	107	4.27	0	0.75 ± 0.14
**A-b-47**	352.26	5.038	34.14	115	4.67	5	0.33 ± 0.01
atovaquone	366.84	6.351	54.37	nd	nd	0	1.55 ± 0.04

**Table 4:** Antiplasmodial activities of 6-fluoro-(3-benzyl)-menadiones and their corresponding 6-H-2-methyl-3-benzylmenadiones. The grey lines in the Table indicate that the antiplasmodial activity of the compounds was described in a patent [13] or in references [8,9,10].

** 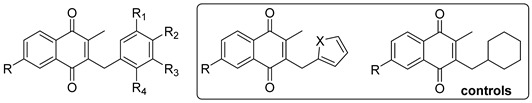 **								
Cpnd	R	R1	R2	R3	R4	X	IC_50_ (µM)	Tox/L6 (µM)
6-F-PD [9]	F	H	CF_3_	H	H	-	0.181	44.7
PD [10]	H	H	CF_3_	H	H	-	0.043	141.7
**A-a-1**	F	H	H	CF_3_	H	-	0.082	12.5
**B-b-1 [9]**	H	H	H	CF_3_	H	-	0.062	57.1
**A-a-2**	F	H	H	H	CF_3_	-	0.138	53.4
**B-b-2 [9]**	H	H	H	H	CF_3_	-	0.026	249.8
**A-a-3**	F	H	OCF_3_	H	H	-	0.330	138.8
**A-b-3**	H	H	OCF_3_	H	H	-	0.083	nd
**A-a-4**	F	H	F	H	H	-	0.302	174.5
**A-b-4 [13]**	H	H	F	H	H	-	0.029	51.7
**A-a-5**	F	H	H	F	H	-	0.246	6.6
**B-b-5**	H	H	H	F	H	-	0.089	71.0
**A-a-6**	F	H	H	H	F	-	0.119	7.6
**A-b-6**	H	H	H	H	F	-	0.453	nd
**A-a-7**	F	H	CF_3_	F	H	-	0.735	85.5
**A-b-7**	H	H	CF_3_	F	H	-	2.958	49.2
**A-a-8**	F	H	CF_3_	Br	H	-	0.167	nd
**A-b-8**	H	H	CF_3_	Br	H	-	0.081	nd
**A-a-9**	F	H	F	CF_3_	H	-	0.149	nd
**A-b-9**	H	H	F	CF_3_	H	-	0.077	nd
**A-a-10**	F	H	CN	H	H	-	0.231	95
**A-b-10 [8]**	H	H	CN	H	H	-	0.374	112.5
**A-a-11**	F	H	H	H	CN	-	0.264	327.0
**A-b-11**	H	H	H	H	CN	-	0.312	186.2
**A-a-12**	F	H	NO_2_	H	H	-	0.177	307.0
**A-b-12 [8]**	H	H	NO_2_	H	H	-	0.201	194.5
**A-a-13**	F	H	I	H	H	-	0.198	40.9
**B-b-13** [9]	H	H	I	H	H	-	0.115	138.6
**A-a-14**	F	H	Cl	H	Cl	-	0.137	nd
**A-b-14**	H	H	Cl	H	Cl	-	0.067	nd
**A-a-15**	F	H	Cl	H	CF_3_	-	0.103	nd
**A-b-15**	H	H	Cl	H	CF_3_	-	0.080	nd
**A-a-16**	F	H	OMe	H	H	-	1.118	195.9
**A-a-17**	F	H	H	OMe	H	-	0.361	165.6
**A-a-18**	F	H	H	H	OMe	-	0.572	189.3
**A-a-19**	F	H	OMe	OMe	H	-	0.171	5.6
**A-b-19 [13]**	H	H	OMe	OMe	H	-	0.142	5.6
**A-a-20**	F	H	OMe	H	OMe	-	0.316	106.6
**A-a-21**	F	H	-O-CH_2_-O-		H	-	0.070	165.6
**A-b-21**	H	H	-O-CH_2_-O-		H	-	0.200	97.9
**A-a-22**	F	H	-O-(CH_2_)_2_-O-		H	-	0.311	nd
**A-b-22**	H	H	-O-(CH_2_)_2_-O-		H	-	0.280	nd
**A-a-23**	F	H	-O-CMe_2_-O-		H	-	0.338	nd
**Cpnd**	**R**	**R1**	**R2**	**R3**	**R4**	**X**	**IC_50_ (µM)**	**Tox/L6 (µM)**
**A-b-23**	H	H	-O-CMe_2_-O-		H	-	0.152	nd
**A-a-24**	F	H	-O-CF_2_-O-		H	-	0.206	nd
**A-b-24**	H	H	-O-CF_2_-O-		H	-	0.077	nd
**A-a-25**	F	H	OMe	OMe	OMe	-	0.340	215.5
**A-a-26**	F	OMe	H	H	OMe	-	0.291	91.7
**A-b-26 [9]**	H	OMe	H	H	OMe	-	0.067	158.5
**A-a-27**	F	OMe	OMe	OMe	H	-	0.265	72.4
**A-a-28**	F	-	-	-	-	S	0.474	5.8
**A-b-28**	H	-	-	-	-	S	0.115	5.2
**B-a-30**	F	H	H	CN	H	-	0.326	33.3
**B-b-3** **0**	H	H	H	CN	H	-	0.190	72.6
**B-a-31**	F	H	H	H	H	-	0.300	174.5
**B-b-31 [9]**	H	H	H	H	H	-	1.695	332.8
**B-a-32**	F	H	NO_2_	F	H	-	0.705	107.4
**B-b-32 [9]**	H	H	NO_2_	F	H	-	0.598	189.2
**B-a-33**	F	H	F	I	H	-	0.376	nd
**B-b-33**	H	H	F	I	H	-	0.136	nd
**B-a-34**	F	H	Br	H	CF_3_	-	0.169	nd
**B-b-34**	H	H	Br	H	CF_3_	-	0.078	nd
**B-a-35**	F	OMe	H	OMe	H	-	0.711	293.0
**B-b-35 [9]**	H	OMe	H	OMe	H	-	0.052	70.3
**C-a-40**	F	H	-S-CH=N-		H	-	2.062	nd
**C-b-40**	H	H	-S-CH=N-		H	-	0.235	50.29
**C-a-41**	F	H	-N=CH-S-		H	-	2.527	nd
**C-b-41**	H	H	-N=CH-S-		H	-	0.370	49.81
**C-a-43**	F	-	-	-	-	O	0.947	3.8
**A-b-43** [8]	H	-	-	-	-	O	0.501	4.2
**C-b-46**	H	H	-SF_5_	H	H	-	0.048	35.1
**A-b-47** [9]	H	bMD-F_5_				-	0.060	161.0
**A-a-29**	F	Controls					0.718	177.8
**A-b-29**	H						0.913	229.9

**Table 5:** In vitro antiplasmodial activities of 6-fluoro–plasmodione versus plasmodione and related fluoro-substituted 3-benzylmenadiones with *P. falciparum* strain NF54 (72 h and 24 h assays) comparing (A.) species differences and serum influence (B.) with 0.5% Albumax or 50% human serum.

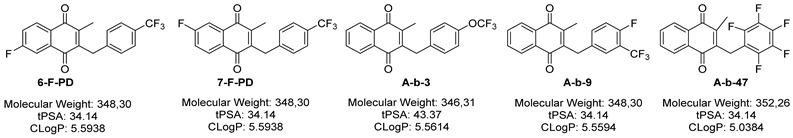					
**A.**					
**Cpnd**	**IC_50_ (nM)**	**IC_50_ (nM)**		**IC_50_ (nM)**	**Species Shift**
	***Pf* NF54, 72 h**	***Pf* NF54, 24 h**		***Pb*, 24 h**	***Pf* versus *Pb***
PD	59	130		130	1.0
6-F-PD	120	104		170	1.6
7-F-PD	93	157		138	0.9
**A-b-3**	83	223		191	0.9
**A-b-9**	62	143		149	1.0
**A-b-47**	91	184		265	1.4
AS	4.8	2.1		13	6.1
CQ	11	4.9		16	3.3
**B.**					
**Cpnd**	**IC_50_ (nM) NF54**		**IC_50_ (nM) NF54**		**Serum Shift**
	**72 h, 0.5% Albumax**		**72 h, 50% Human Serum**		
PD	57		270		4.7
6-F-PD	93		80		0.9
7-F-PD	89		377		4.2
**A-b-3**	83		473		5.7
**A-b-9**	59		355		6.1
**A-b-47**	81		324		4.0
AS	1.7		1.0		0.6
CQ	5.0		6.6		1.3

**Table 6:** In vivo antiplasmodial activities of 6-fluoro–plasmodione versus plasmodione and related fluoro-substituted 3-benzylmenadiones ^1^.

** 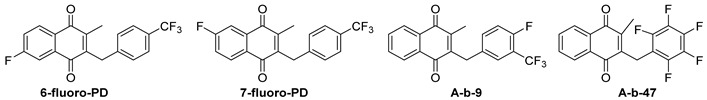 **									
Cpnd	Oral Dose (mg/kg)	Parasitized RBC over 100				Avg.	% of Control	Activity (%)	Avg. Mouse Survival in Days
Control	0	59.90	51.10	71.10	68.50	58.90			4.0
PD	4 × 100	61.4	64.8	57.0		60.90	103.40	−3.40	4.0
PD	4 × 50	66.5	70.6	71.8		69.63	118.22	−18.22	4.0
6-F-PD	4 × 50	28.2	35.8	27.8		30.60	51.95	48.05	7.0
7-F-PD ^2^	4 × 50	64.7	69.0	62.2		65.30	110.87	−10.87	4.0
**A-b-9**	4 × 50	70.3	49.1	52.4		57.27	97.23	2.77	4.0
**A-b-47** ^3^	4 × 50	65.4	62.4	72.5		66.77	113.36	−13.36	4.0

^1^: Three–four mice per experiment. Compounds were formulated in [70% Tween 80 − 30% EtOH]/water (10:90) and administered *p.o.* All animals were euthanized S2 (infected and treated). ^2^: the synthesis and the antiplasmodial activity of **7-F-PD** was described, under the product code 17d, in ref. [12]. ^3^: The synthesis and the in vitro antiplasmodial activity of **A-b-47** was described in the patent ref. [13].

## Data Availability

The original contributions presented in the study are included in the article/in the Supplementary Materials; further inquiries can be directed to the corresponding authors.

## Supplementary Materials

The following supporting information can be downloaded at: https://www.mdpi.com/article/10.3390/molecules30112446/s1. They include Pages S2–S180: ^1^H, ^19^F and ^13^C NMR spectra of key/new compounds.
