# Supplementary material for: Design, Synthesis and Antiplasmodial Activities of a Library of Fluorine-Based 3-Benzylmenadiones
Source: Molecules. 2025 Jun 3;30(11):2446. doi: 10.3390/molecules30112446 (PMC12156041; doi:10.3390/molecules30112446)

Supplementary Materials

Article

Design, Synthesis and Antiplasmodial Activities of a Library of Fluorine-Based-3-Benzylmenadiones

Matthieu Roignant [1], Jimmy Richard [1], Maxime Donzel [1], Matthias Rottmann [2,3], Pascal Mäser [2,3], and Elisabeth Davioud-Charvet [1,*]

[1] UMR7042 Université de Strasbourg-CNRS-UHA, Laboratoire d'Innovation Moléculaire et Applications (LIMA), Team Bio(IN)organic and Medicinal Chemistry, European School of Chemistry, Polymers and Materials (ECPM), 25 rue Becquerel, F-67087 Strasbourg, France; roignant.matthieu@gmail.com (M.Roi.); jrichards@unistra.fr (J.R.); mdonzel@unistra.fr (M.D.);

[2] Swiss Tropical and Public Health Institute, Kreuzstrasse 2, CH-4123 Allschwil, Switzerland; matthias.rottmann@swisstph.ch (M.Rot.); pascal.maeser@swisstph.ch (P.M.);
jennifer.keiser@swisstph.ch (J.K.)

[3] University of Basel, Petersgraben 1, CH-4001 Basel, Switzerland

* Correspondence: elisabeth.davioud@unistra.fr

Content: Pages S2-S13: S1. Characterisation of key/new compounds; S14-S192: S2. NMR spectra of key/new compounds.

S1. Characterisation of key/new compounds

*6-fluoro-2-methyl-3-(3-(trifluoromethyl)benzyl)naphthalene-1,4-dione (**A-a-1**):* According to the general procedure A, 3-trifluoromethylphenylacetic acid (2.0 equiv.) was used. **A-a-1** was isolated by purification by flash chromatography on silica gel (Toluene/Cyclohexane, 7/3, v/v, UV) as a yellow solid (80 mg, 44% yield). **^{1}H NMR** ($CDCl_3$, 400 MHz): δ 8.12 (dd, J = 8.6, 5.3 Hz, 1H), 7.71 (dd, J = 8.6, 2.6 Hz, 1H), 7.50-7.43 (m, 2H), 7.42-7.32 (m, 3H), 4.07 (s, 2H), 2.26 (s, 3H). **^{13}C {^{1}H} NMR** ($CDCl_3$, 101 MHz): δ 183.9, 183.5 (d, $^4J_{C-F}$ = 1.0 Hz), 166.1 (d, $^1J_{C-F}$ = 257.1 Hz), 145.3, 144.6 (d, $^4J_{C-F}$ = 1.9 Hz), 138.9, 134.6 (d, $^3J_{C-F}$ = 7.8 Hz), 132.0 (d, $^4J_{C-F}$ = 1.0 Hz), 131.1 (q, $^2J_{C-F}$ = 32.1 Hz), 129.8 (d, $^3J_{C-F}$ = 8.9 Hz), 129.3, 128.8 (d, $^4J_{C-F}$ = 3.0 Hz), 125.41 (q, $^3J_{C-F}$ = 3.8 Hz), 124.1 (d, $^1J_{C-F}$ = 272 Hz), 123.6 (q, $^3J_{C-F}$ = 3.8 Hz), 120.9 (d, $^2J_{C-F}$ = 22.6 Hz), 113.3 (d, $^2J_{C-F}$ = 23.5 Hz), 32.4, 13.5. **^{19}F NMR** ($CDCl_3$, 377 MHz): δ -62.59, -102.26 (td, J = 8.3, 5.3 Hz). **HRMS (ESI+)** *m*/*z*: $[M+H]^+$ calculated for $C_{19}H_{13}F_4O_2$: <u>349.084619</u>, found <u>349.084198</u>. **M.p.** = 81-83 °C.

*6-fluoro-2-methyl-3-(2-(trifluoromethyl)benzyl)naphthalene-1,4-dione (**A-a-2**):* According to the general procedure A, 2-trifluoromethylphenylacetic acid (1.5 equiv.) was used. **A-a-2** was isolated by purification by flash chromatography on silica gel (Toluene/Cyclohexane, 7/3, v/v, UV) as a yellow solid (70 mg, 38% yield). **^{1}H NMR** ($CDCl_3$, 400 MHz): δ 8.18 (dd, J = 8.6, 5.2 Hz, 1H), 7.74 (dd, J = 8.5, 2.6 Hz, 1H), 7.70 (d, J = 7.6 Hz, 1H), 7.44-7.28 (m, 3H), 6.94 (d, J = 7.6 Hz, 1H), 4.23 (s, 2H), 2.10 (s, 3H). **^{13}C {^{1}H} NMR** ($CDCl_3$, 101 MHz): δ 183.8, 183.4 (d, $^4J_{C-F}$ = 2.0 Hz), 166.1 (d, $^1J_{C-F}$ = 257.6 Hz), 146.7, 144.5 (d, $^5J_{C-F}$ = 1.0 Hz), 136.5 (d, $^4J_{C-F}$ = 2.0 Hz), 134.6 (d, $^3J_{C-F}$ = 8.0 Hz), 132.2 (d, $^4J_{C-F}$ = 1.0 Hz), 129.9 (d, $^3J_{C-F}$ = 7.0 Hz), 128.9 (d, $^3J_{C-F}$ = 3.0 Hz), 128.7 (d, $^2J_{C-F}$ = 33.3 Hz), 128.2, 126.7, 126.5 (q, $^3J_{C-F}$ = 5.7 Hz), 124.7 (d, $^1J_{C-F}$ = 274.7 Hz), 121.0 (d, $^2J_{C-F}$ = 22.2 Hz), 113.4 (d, $^2J_{C-F}$ = 24.0 Hz), 28.8 (d, $^3J_{C-F}$ = 2.0 Hz), 13.2. **^{19}F NMR** ($CDCl_3$, 377 MHz): δ -60.88, -102.13 (td, J = 8.4, 5.3 Hz). **HRMS (ESI+)** *m/z:* $[M+Na]^+$ calculated for $C_{19}H_{12}F_4NaO_2$: <u>371.066563</u>, found <u>371.066903</u>. **M.p.** = 102-103 °C.

*6-fluoro-2-methyl-3-(4-(trifluoromethoxy)benzyl)naphthalene-1,4-dione (**A-a-3**):* According to the general procedure A, 4-trifluoromethoxylphenylacetic acid (2.0 equiv.) was used. **A-a-3** was isolated by purification by flash chromatography on silica gel (Toluene/Cyclohexane, 7/3, v/v, UV) as a yellow solid (108 mg, 56% yield). **^{1}H NMR** ($CDCl_3$, 400 MHz): δ 8.06 (dd, J = 8.6, 5.3 Hz, 1H), 7.65 (dd, J = 8.6, 2.6 Hz, 1H), 7.29 (td, J = 8.3, 2.6 Hz, 1H), 7.18 (d, J = 8.7 Hz, 2H), 7.05 (d, J = 8.2 Hz, 2H), 3.95 (s, 2H), 2.19 (s, 3H). **^{13}C {^{1}H} NMR** ($CDCl_3$, 101 MHz): δ 184.0, 183.6 (d, $^4J_{C-F}$ = 1.0 Hz), 166.3 (d, $^1J_{C-F}$ = 258.6 Hz), 148.0 (d, $^4J_{C-F}$ = 2.0 Hz), 145.04 (d, $^5J_{C-F}$ = 2.0 Hz), 145.00, 136.7, 134.6 (d, $^3J_{C-F}$ = 8.1 Hz), 130.0 (2C), 129.8 (d, $^3J_{C-F}$ = 8.1 Hz), 128.8 (d, $^3J_{C-F}$ = 3.0 Hz), 121.4 (2C), 120.9 (d, $^2J_{C-F}$ = 23.2 Hz), 120.5 (d, $^1J_{C-F}$ = 258.6 Hz), 113.3 (d, $^2J_{C-F}$ = 23.2 Hz), 32.0, 13.5. **^{19}F NMR** ($CDCl_3$, 377 MHz): δ -57.95, -102.33 (td, J = 8.2, 5.4 Hz). **HRMS (ESI+)** *m*/*z*: $[M+H]^+$ calculated for $C_{19}H_{13}F_4O_3$: <u>365.079534</u>, found <u>365.080865</u>. **M.p.** = 70-72 °C.

*6-fluoro-3-(4-fluorobenzyl)-2-methylnaphthalene-1,4-dione (**A-a-4**):* According to the general procedure A, 4-fluorophenylacetic acid (2.0 equiv.) was used. **A-a-4** was isolated by purification by flash chromatography on silica gel (Toluene/Cyclohexane, gradient from 6/4 to 7/3, v/v, UV) as a yellow solid (120 mg, 77% yield). **^{1}H NMR** ($CDCl_3$, 400 MHz): δ 8.11 (dd, J = 8.6, 5.3 Hz, 1H), 7.71 (dd, J = 8.6, 2.7 Hz, 1H), 7.35 (td, J = 8.3, 2.7 Hz, 1H), 7.22-7.13 (m, 2H), 7.00 6.91 (m, 2H), 3.98 (s, 2H), 2.25 (s, 3H). **^{13}C {^{1}H} NMR** ($CDCl_3$, 101 MHz): δ 184.1, 183.6, 166.1 (d, $^1J_{C-F}$ = 257.0 Hz), 161.8 (d, $^1J_{C-F}$ = 245.0 Hz), 145.4, 144.8, 134.7 (d, $^3J_{C-F}$ = 8.1 Hz), 133.6 (d, $^4J_{C-F}$ = 3.0 Hz), 130.2 (2C, d, $^3J_{C-F}$ = 7.1 Hz), 129.7 (d, $^3J_{C-F}$ = 8.1 Hz), 128.8 (d, $^4J_{C-F}$ = 3.0 Hz), 120.9 (d, $^2J_{C-F}$ = 23.2 Hz), 115.6 (2C, d, $^2J_{C-F}$ = 21.2 Hz), 113.3 (d, $^2J_{C-F}$ = 24.2 Hz), 31.8, 13.4. **^{19}F NMR** ($CDCl_3$, 377 MHz): δ -102.41 (td, J = 8.6, 5.3 Hz), -116.34 (s). **HRMS (ESI+)** m/z: [M+H]+ calculated for $C_{18}H_{13}F_2O_2$: <u>299.087813</u>, found <u>299.087919</u>. **M.p.** = 117-119 °C.

*6-fluoro-3-(3-fluorobenzyl)-2-methylnaphthalene-1,4-dione (**A-a-5**):* According to the general procedure A, 3-fluorophenylacetic acid (2.0 equiv.) was used. **A-a-5** was isolated by purification by flash chromatography on silica gel (Toluene/Cyclohexane, 7/3, v/v, UV) as a yellow solid (54 mg, 34% yield). **^{1}H NMR** ($CDCl_3$, 400 MHz): δ 8.13 (dd, J = 8.6, 5.3 Hz, 1H), 7.73 (dd, J = 8.6, 2.6 Hz, 1H), 7.36 (td, J = 8.3, 2.7 Hz, 1H), 7.26-7.20 (m, 1H), 7.02-6.98 (m, 1H), 6.95-6.85 (m, 2H), 4.02 (s, 2H), 2.24 (s, 3H). **^{13}C {^{1}H} NMR** ($CDCl_3$, 101 MHz): δ 184.0, 183.6 (d, $^4J_{C-F}$ = 1.4 Hz), 166.1 (d, $^1J_{C-F}$ = 257.0 Hz), 163.1 (d, $^1J_{C-F}$ = 246.1 Hz), 145.1, 144.9 (d, $^5J_{C-F}$ = 1.8 Hz), 140.4 (d, $^3J_{C-F}$ = 7.4 Hz), 134.6 (d, $^3J_{C-F}$ = 7.8 Hz), 130.2 (d, $^3J_{C-F}$ = 8.3 Hz), 129.8 (d, $^3J_{C-F}$ = 8.9 Hz), 128.8 (d, $^4J_{C-F}$ = 3.3 Hz), 124.3 (d, $^4J_{C-F}$ = 2.9 Hz), 120.9 (d, $^2J_{C-F}$ = 22.5 Hz), 115.6 (d, $^2J_{C-F}$ = 21.6 Hz), 113.6 (d, $^2J_{C-F}$ = 21.0 Hz), 113.3 (d, $^2J_{C-F}$ = 23.4 Hz), 32.3 (d, $^4J_{C-F}$ = 1.7 Hz), 13.4. **^{19}F NMR** ($CDCl_3$, 377 MHz): δ -102.34 (td, J = 8.3, 5.3 Hz), -112.8 (m). **HRMS (ESI+)** *m*/*z*: $[M+H]^+$ calculated for $C_{18}H_{13}F_2O_2$: <u>299.087813</u>, found <u>299.087790</u>. **M.p.** = 102-103 °C.

*6-fluoro-3-(2-fluorobenzyl)-2-methylnaphthalene-1,4-dione (**A-a-6**):* According to the general procedure A, 2-fluorophenylacetic acid (2.0 equiv.) was used. **A-a-6** was isolated by purification by flash chromatography on silica gel (Toluene/Cyclohexane, 7/3, v/v, UV) as a yellow solid (71 mg, 45% yield). **^{1}H NMR** ($CDCl_3$, 400 MHz): δ 8.12 (dd, J = 8.6, 5.3 Hz, 1H), 7.72 (dd, J = 8.6, 2.6 Hz, 1H), 7.35 (td, J = 8.3, 2.6 Hz, 1H), 7.21-7.13 (m, 2H), 7.02 (t, J = 8.2 Hz, 2H), 4.04 (s, 2H), 2.22 (s, 3H). **^{13}C {^{1}H} NMR** ($CDCl_3$, 101 MHz): δ 184.0, 183.5 (d, $^4J_{C\text{-}F}$ = 1.3 Hz), 166.1 (d, $^1J_{C\text{-}F}$ = 256.8 Hz), 160.9 (d, $^1J_{C\text{-}F}$ = 245.6 Hz), 145.5, 145.4-144.3 (m), 134.7 (d, $^3J_{C\text{-}F}$ = 7.8 Hz), 130.5 (d, $^3J_{C\text{-}F}$ = 4.2 Hz), 129.7 (d, $^3J_{C\text{-}F}$ = 8.9 Hz), 128.8 (d, $^4J_{C\text{-}F}$ = 3.2 Hz), 128.4 (d, $^3J_{C\text{-}F}$ = 8.2 Hz), 124.8 (d, $^2J_{C\text{-}F}$ = 15.5 Hz), 124.3 (d, $^4J_{C\text{-}F}$ = 3.5 Hz), 120.8 (d, $^2J_{C\text{-}F}$ = 22.6 Hz), 115.5 (d, $^2J_{C\text{-}F}$ = 22.2 Hz), 113.3 (d, $^2J_{C\text{-}F}$ = 23.5 Hz), 25.8 (d, $^3J_{C\text{-}F}$ = 3.5 Hz), 13.2 (d, $^6J_{C\text{-}F}$ = 2.7 Hz). **^{19}F NMR** ($CDCl_3$, 377 MHz): δ -102.49 (td, J = 8.3, 5.3 Hz), -116.38 (dt, J = 12.0, 7.0 Hz). **HRMS (ESI+)** *m/z*: $[M+H]^+$ calculated for $C_{18}H_{13}F_2O_2$: 299.087813, found 299.087148. **M.p.** = 96-98 °C.

*6-fluoro-3-(3-fluoro-4-(trifluoromethyl)benzyl)-2-methylnaphthalene-1,4-dione (**A-a-7**):* According to the general procedure A, 3-fluoro-4-(trifluoromethyl)phenylacetic acid (2.0 equiv.) was used. This reaction was realized with 1.58 mmol (300 mg) of 6-fluoro-menadione. **A-a-7** was isolated by purification by flash chromatography on silica gel (Toluene/Cyclohexane, 7/3, v/v, UV) as a yellow solid (434 mg, 75% yield). **^{1}H NMR** ($CDCl_3$, 400 MHz): δ 8.12 (dd, J = 8.6, 5.2 Hz, 1H), 7.70 (dd, J = 8.5, 2.7 Hz, 1H), 7.49 (t, J = 7.8 Hz, 1H), 7.37 (td, J = 8.3, 2.7 Hz, 1H), 7.11 (d, J = 8.1 Hz, 1H), 7.05 (d, J = 11.3 Hz, 1H), 4.06 (s, 2H), 2.25 (s, 3H). **^{13}C {^{1}H} NMR** ($CDCl_3$, 101 MHz): δ 183.7, 183.4 (d, $^4J_{C\text{-}F}$ = 2.0 Hz), 166.2 (d, $^1J_{C\text{-}F}$ = 252.5 Hz), 157.9 (d, $^1J_{C\text{-}F}$ = 262.6 Hz), 145.6, 145.1 (d, $^3J_{C\text{-}F}$ = 8.1 Hz), 143.9 (d, $^5J_{C\text{-}F}$ = 2.0 Hz), 134.5 (d, $^3J_{C\text{-}F}$ = 8.1 Hz), 129.9 (d, $^3J_{C\text{-}F}$ = 9.1 Hz), 128.7 (d, $^4J_{C\text{-}F}$ = 3.0 Hz), 127.5-127.4 (m), 124.3 (d, $^4J_{C\text{--}F}$ = 4.0 Hz), 124.0, 121.3, 121.1 (d, $^2J_{C\text{-}F}$ = 23.2 Hz), 117.0 (d, $^2J_{C\text{-}F}$ = 21.2 Hz), 113.3 (d, $^2J_{C\text{-}F}$ = 23.2 Hz), 32.3 (d, $^4J_{C\text{-}F}$ = 1.0 Hz), 13.5. **^{19}F NMR** ($CDCl_3$, 377 MHz): δ -61.25 (d, J = 12.4 Hz), -102.03 (td, J = 8.2, 5.2 Hz), -114.02 (pd, J = 12.2, 7.5 Hz). **HRMS (ESI+)** *m/z*: $[M+H]^+$ calculated for $C_{19}H_{12}F_5O_2$: 367.075197, found 367.072748. **M.p.** = 103-105 °C.

*3-(3-bromo-4-(trifluoromethyl)benzyl)-6-fluoro-2-methylnaphthalene-1,4-dione (**A-a-8**):* According to the general procedure A, 2-(3-bromo-4-(trifluoromethyl)phenyl)acetic acid (1.0 equiv.) was used. **A-a-8** was isolated by purification by flash chromatography on silica gel (Toluene/Cyclohexane, 7/3, v/v, UV) as a yellow solid (434 mg, 75% yield). **^{1}H NMR** ($CDCl_3$, 400 MHz): δ 8.15 (dd, J = 8.6, 5.2 Hz, 1H), 7.73 (dd, J = 8.5, 2.6 Hz, 1H), 7.61-7.54 (m, 2H), 7.38 (td, J = 8.3, 2.7 Hz, 1H), 7.26 (d, J = 8.1 Hz, 1H), 4.04 (s, 2H), 2.26 (s, 3H). **^{13}C {^{1}H} NMR** ($CDCl_3$, 101 MHz): δ 183.8, 183.4 (d, $^4J_{C\text{-}F}$ = 1.4 Hz), 166.2 (d, $^1J_{C\text{-}F}$ = 257.4 Hz), 145.6, 143.9 (d, $^5J_{C\text{-}F}$ = 1.8 Hz), 143.8 (d, $^5J_{C\text{-}F}$ = 1.0 Hz), 134.9, 134.5 (d, $^3J_{C\text{-}F}$ = 7.9 Hz), 129.0, 128.8 (d, $^3J_{C\text{-}F}$ = 3.2 Hz), 128.7 (d, $^2J_{C\text{-}F}$ = 31.5 Hz), 128.2 (q, $^3J_{C\text{-}F}$ = 5.3 Hz), 127.5, 123.0 (q, $^1J_{C\text{-}F}$ = 273.1 Hz), 121.1 (d, $^2J_{C\text{-}F}$ = 22.6 Hz), 120.5 (q, $^4J_{C\text{-}F}$ = 1.9 Hz), 113.5 (d, $^2J_{C\text{-}F}$ = 23.5 Hz), 32.1, 13.6. **^{19}F NMR** ($CDCl_3$, 377 MHz): δ -62.45, -101.95 (td, J = 8.3, 5.3 Hz). **HRMS (APCI+)** *m/z*: $[M]^+$ calculated for $C_{19}H_{11}BrF_4O_2$: 425.9873, found 425.9897. **M.p.** = 105-107 °C.

*6-fluoro-3-(4-fluoro-3-(trifluoromethyl)benzyl)-2-methylnaphthalene-1,4-dione (**A-a-9**):* According to the general procedure A, 3-fluoro-4-(trifluoromethyl)phenylacetic acid (1.5 equiv.) was used. This reaction was realized with 1.58 mmol (300 mg) of 6-fluoro-menadione. **A-a-9** was isolated by purification by flash chromatography on silica gel (Toluene/Cyclohexane, 6/4, v/v, UV) as a yellow solid (302 mg, 52% yield). **^{1}H NMR** ($CDCl_3$, 400 MHz): δ 8.14 (dd, J = 8.6, 5.2 Hz, 1H), 7.72 (dd, J = 8.5, 2.6 Hz, 1H), 7.46 (dd, J = 6.6, 1.8 Hz, 1H), 7.43-7.33 (m, 2H), 7.15-7.05 (m, 1H), 4.02 (s, 2H), 2.27 (s, 3H). **^{13}C {^{1}H} NMR** ($CDCl_3$, 101 MHz): δ 183.9, 183.5 (d, $^4J_{C\text{-}F}$ = 1.4 Hz), 166.2 (d, $^1J_{C\text{-}F}$ = 257.2 Hz), 158.6 (qd, J = 255.5, 1.9 Hz), 145.2, 144.5 (d, $^5J_{C\text{-}F}$ = 1.6 Hz), 134.5 (d, $^3J_{C\text{-}F}$ = 7.9 Hz), 134.2 (d, $^4J_{C\text{-}F}$ = 3.9 Hz), 134.1 (d, $^3J_{C\text{-}F}$ = 8.3 Hz), 129.9 (d, $^3J_{C\text{-}F}$ = 8.9 Hz), 128.8 (d, $^4J_{C\text{-}F}$ = 3.3 Hz), 127.2 (qd, J = 4.6, 1.5 Hz), 122.4 (qd, J = 272.8, 0.9 Hz), 121.1 (d, $^2J_{C\text{-}F}$ = 22.6 Hz), 118.7 (qd, J = 32.8, 12.5 Hz), 117.3 (d, $^2J_{C\text{-}F}$ = 20.7 Hz), 113.4 (d, $^2J_{C\text{-}F}$ = 23.5 Hz), 31.8, 13.5. **^{19}F NMR** ($CDCl_3$, 377 MHz): δ -61.40 (d, J = 12.7 Hz), -102.08 (td, J = 8.3, 5.3 Hz), -117.50 (ddp, J = 17.8, 11.2, 6.2 Hz). **HRMS (APCI+)** *m/z*: $[M]^+$ calculated for $C_{19}H_{11}F_5O_2$: 366.0674, found 366.0655. **M.p.** = 122-124 °C.

*4-((7-fluoro-3-methyl-1,4-dioxo-1,4-dihydronaphthalen-2-yl)methyl)benzonitrile (**A-a-10**):* According to the general procedure A, 4-cyanophenylacetic acid (2 equiv.) was used. **A-a-10** was isolated by purification by flash chromatography on silica gel (Toluene/Cyclohexane, gradient from 7/3 to 8/2, v/v, UV) as a yellow solid (119 mg, 74% yield). **^{1}H NMR** ($CDCl_3$, 400 MHz): δ 8.11 (dd, J = 8.6, 5.2 Hz, 1H), 7.69 (dd, J = 8.5, 2.7 Hz, 1H), 7.58-7.52 (m, 2H), 7.36 (td, J = 8.3, 2.7 Hz, 1H), 7.39-7.30 (m, 3H), 4.06 (s, 2H), 2.23 (s, 3H). **^{13}C {^{1}H} NMR** ($CDCl_3$, 101 MHz): δ 183.7, 183.4 (d, $^4J_{C\text{-}F}$ = 2.0 Hz), 166.1 (d, $^1J_{C\text{-}F}$ = 257.0 Hz), 145.5, 144.1 (d, $^5J_{C\text{-}F}$ = 2.0 Hz), 143.6, 134.4 (d, $^3J_{C\text{-}F}$ = 8.1 Hz), 132.6 (2C), 129.8 (d, $^3J_{C\text{-}F}$ = 9.1 Hz), 129.4 (2C),

128.7 (d, $^{3}J_{C-F}$ = 3.0 Hz), 121.0 (d, $^{2}J_{C-F}$ = 22.2 Hz), 118.8, 113.3 (d, $^{2}J_{C-F}$ = 24.3 Hz), 110.6, 32.8, 13.5. **^{19}F NMR** (CDCl$_3$, 377 MHz): δ -102.00 (td, *J* = 8.3, 5.1 Hz). **HRMS (ESI+)** *m/z*: [M+H]$^+$ calculated for $C_{19}H_{13}FNO_2$: 306.092483, found 306.091877. **M.p.** = 130-132 °C.

*2-((7-fluoro-3-methyl-1,4-dioxo-1,4-dihydronaphthalen-2-yl)methyl)benzonitrile (**A-a-11**):* According to the general procedure A, 2-cyanophenylacetic acid (2.0 equiv.) was used. **A-a-11** was isolated by purification by flash chromatography on silica gel (Toluene/Cyclohexane, 9/1 then Cyclohexane/Ethyl acetate, 9/1, v/v, UV) as a yellow solid (77 mg, 48% yield). **^{1}H NMR** (CDCl$_3$, 400 MHz): δ 8.13 (dd, *J* = 8.6, 5.2 Hz, 1H), 7.70 (dd, *J* = 8.5, 2.6 Hz, 1H), 7.65 (dd, *J* = 7.7, 1.2 Hz, 1H), 7.45 (td, *J* = 7.7, 1.4 Hz, 1H), 7.37 (td, *J* = 8.3, 2.7 Hz, 1H), 7.34-7.28 (m, 1H), 7.11 (d, *J* = 7.9 Hz, 1H), 4.24 (s, 2H), 2.19 (s, 3H). **^{13}C {^{1}H} NMR** (CDCl$_3$, 101 MHz): δ 183.6, 183.3 (d, $^{4}J_{C-F}$ = 2.0 Hz), 166.0 (d, $^{1}J_{C-F}$ = 257.6 Hz), 146.3, 143.7, 141.8, 134.5 (d, $^{3}J_{C-F}$ = 8.1 Hz), 133.20, 133.15, 129.8 (d, $^{3}J_{C-F}$ = 9.1 Hz), 128.8 (d, $^{4}J_{C-F}$ = 4.0 Hz), 128.5, 127.2, 121.0 (d, $^{2}J_{C-F}$ = 23.2 Hz), 117.9, 113.3 (d, $^{2}J_{C-F}$ = 23.2 Hz), 112.9, 31.0, 13.5. **^{19}F NMR** (CDCl$_3$, 377 MHz): δ -102.01 (td, *J* = 8.2, 5.3 Hz). **HRMS (ESI+)** *m/z*: [M+Na]$^+$ calculated for $C_{19}H_{12}FNNaO_2$: 328.074428, found 328.074636. **M.p.** = 160-162 °C.

*6-fluoro-2-methyl-3-(4-nitrobenzyl)naphthalene-1,4-dione (**A-a-12**):* According to the general procedure A, 4-nitrophenylacetic acid (2.0 equiv.) was used. **A-a-12** was isolated by purification by flash chromatography on silica gel (Toluene/cyclohexane, 8/2, UV) as an orange solid (144 mg, 64% yield). **^{1}H NMR** (CDCl$_3$, 400 MHz): δ 8.15 (dd, *J* = 8.6, 5.2 Hz, 1H), 7.73 (dd, *J* = 8.5, 2.6 Hz, 1H), 7.61-7.54 (m, 2H), 7.38 (td, *J* = 8.3, 2.7 Hz, 1H), 7.26 (d, *J* = 8.1 Hz, 1H), 4.04 (s, 2H), 2.26 (s, 3H). **^{13}C {^{1}H} NMR** (CDCl$_3$, 101 MHz): δ 183.8, 183.4 (d, $^{4}J_{C-F}$ = 1.4 Hz), 166.2 (d, $^{1}J_{C-F}$ = 257.4 Hz), 145.6, 143.9 (d, $^{5}J_{C-F}$ = 1.8 Hz), 143.8 (d, $^{5}J_{C-F}$ = 1.0 Hz), 134.9, 134.5 (d, $^{3}J_{C-F}$ = 7.9 Hz), 129.0, 128.8 (d, $^{3}J_{C-F}$ = 3.2 Hz), 128.7 (d, $^{2}J_{C-F}$ = 31.5 Hz), 128.2 (q, $^{3}J_{C-F}$ = 5.3 Hz), 127.5, 123.0 (q, $^{1}J_{C-F}$ = 273.1 Hz), 121.1 (d, $^{2}J_{C-F}$ = 22.6 Hz), 120.5 (q, $^{4}J_{C-F}$ = 1.9 Hz), 113.5 (d, $^{2}J_{C-F}$ = 23.5 Hz), 32.1, 13.6. **^{19}F NMR** (CDCl$_3$, 377 MHz): δ -62.45, -101.95 (td, *J* = 8.3, 5.3 Hz). **HRMS (APCI+)** *m/z*: [M]$^+$ calculated for $C_{19}H_{11}BrF_4O_2$: 425.9873, found 425.9897. **M.p.** = 105-107 °C.

*6-fluoro-3-(4-iodobenzyl)-2-methylnaphthalene-1,4-dione (**A-a-13**):* According to the general procedure A, 4-iodophenylacetic acid (2.0 equiv.) was used. The reaction was realized with 1.58 mmol (300 mg) of 6-fluoro-menadione. **A-a-13** was isolated by purification by flash chromatography on silica gel (Toluene/Cyclohexane, gradient from 6/4 to 7/3, v/v, UV) as a yellow solid (584 mg, 85% yield). **^{1}H NMR** (CDCl$_3$, 400 MHz): δ 8.11 (dd, *J* = 8.6, 5.2 Hz, 1H), 7.70 (dd, *J* = 8.6, 2.7 Hz, 1H), 7.62-7.53 (m, 2H), 7.35 (td, *J* = 8.3, 2.7 Hz, 1H), 7.00-6.92 (m, 2H), 3.94 (s, 2H), 2.23 (s, 3H). **^{13}C {^{1}H} NMR** (CDCl$_3$, 101 MHz): δ 183.9, 183.5, 167.6 (d, $^{1}J_{C-F}$ = 257.6 Hz), 144.94, 144.93, 137.8 (2C), 137.6, 134.6 (d, $^{3}J_{C-F}$ = 8.1 Hz), 130.7 (2C), 129.7 (d, $^{3}J_{C-F}$ = 9.1 Hz), 128.7 (d, $^{4}J_{C-F}$ = 3.0 Hz), 120.9 (d, $^{2}J_{C-F}$ = 23.2 Hz), 113.3 (d, $^{2}J_{C-F}$ = 23.2 Hz), 91.9, 32.1, 13.5. **^{19}F NMR** (CDCl$_3$, 377 MHz): δ -102.25 (td, *J* = 8.3, 5.2 Hz). **HRMS (ESI+)** *m/z*: [M+H]$^+$ calculated for $C_{18}H_{13}FIO_2$: 406.993882, found 406.991205. **M.p.** = 115-117 °C.

*3-(2,4-dichlorobenzyl)-6-fluoro-2-methylnaphthalene-1,4-dione (**A-a-14**):* According to the general procedure A, 2,4-dichlorophenylacetic acid (1.5 equiv.) was used. **A-a-14** was isolated by purification by flash chromatography on silica gel (Toluene/Cyclohexane, 1/1, v/v, UV) as a yellow solid (108 mg, 59% yield). **^{1}H NMR** (CDCl$_3$, 400 MHz): δ 8.15 (dd, *J* = 8.6, 5.2 Hz, 1H), 7.71 (dd, *J* = 8.5, 2.7 Hz, 1H), 7.42-7.35 (m, 2H), 7.10 (dd, *J* = 8.3, 2.2 Hz, 1H), 6.91 (d, *J* = 8.4 Hz, 1H), 4.07 (s, 2H), 2.14 (s, 3H). **^{13}C {^{1}H} NMR** (CDCl$_3$, 126 MHz): δ 183.7, 183.4 (d, $^{4}J_{C-F}$ = 1.5 Hz), 166.2 (d, $^{1}J_{C-F}$ = 257.1 Hz), 146.4, 144.2 (d, $^{5}J_{C-F}$ = 1.9 Hz), 134.7, 134.6 (d, $^{3}J_{C-F}$ = 8.1 Hz), 134.2, 133.0, 129.92, 129.87 (d, $^{3}J_{C-F}$ = 8.8 Hz), 129.6, 128.8 (d, $^{4}J_{C-F}$ = 3.2 Hz), 127.4, 121.0 (d, $^{2}J_{C-F}$ = 22.4 Hz), 113.4 (d, $^{2}J_{C-F}$ = 23.5 Hz), 29.7, 13.5. **^{19}F NMR** (CDCl$_3$, 377 MHz): δ -102.08 (td, *J* = 8.2, 5.0 Hz). **HRMS (APCI+)** *m/z*: [M+H]$^+$ calculated for $C_{18}H_{12}Cl_2FO_2$: 349.0193, found 349.0187. **M.p.** = 127-128 °C.

*3-(4-chloro-2-(trifluoromethyl)benzyl)-6-fluoro-2-methylnaphthalene-1,4-dione (**A-a-15**):* According to the general procedure A, 2-trifluoromethyl-4-chlorophenylacetic acid (2.0 equiv.) was used. **A-a-15** was isolated by purification by flash chromatography on silica gel (Toluene/Cyclohexane, 7/3, v/v, UV) as a yellow solid (93 mg, 46% yield). **^{1}H NMR** (CDCl$_3$, 400 MHz): δ 8.17 (dd, *J* = 8.5, 5.2 Hz, 1H), 7.72 (dd, *J* = 8.5, 2.4 Hz, 1H), 7.70-7.65 (m, 1H), 7.40 (td, *J* = 8.3, 2.5 Hz, 1H), 7.33 (d, *J* = 7.3 Hz, 1H), 6.89 (d, *J* = 8.3 Hz, 1H), 4.17 (s, 2H), 2.10 (s, 3H). **^{13}C {^{1}H} NMR** (CDCl$_3$, 101 MHz): δ 183.6, 183.3 (d, $^{4}J_{C-F}$ = 1.5 Hz), 166.2 (d, $^{1}J_{C-F}$ = 257.5 Hz), 146.8, 143.9, 135.1 (d, $^{5}J_{C-F}$ = 1.4 Hz), 134.6 (d, $^{3}J_{C-F}$ = 8.0 Hz), 132.8, 132.2, 130.2 (q, $^{2}J_{C-F}$ = 30.9 Hz), 130.0 (d, $^{3}J_{C-F}$ = 8.9 Hz), 129.8, 128.9 (d, $^{3}J_{C-F}$ = 3.2 Hz), 126.9 (q, $^{3}J_{C-F}$ = 6.0 Hz), 123.8 (q, $^{1}J_{C-F}$ = 274.4 Hz), 121.1 (d, $^{2}J_{C-F}$ = 22.5 Hz), 113.5 (d, $^{2}J_{C-F}$ = 23.4 Hz), 28.5 (q, $^{4}J_{C-F}$ = 2.7 Hz), 13.3. **^{19}F NMR** (CDCl$_3$, 377

MHz): δ -61.28, -101.88 (td, J = 8.2, 5.5 Hz). **HRMS (APCI+)** m/z: [M]$^+$ calculated for $C_{19}H_{11}ClF_4O_2$: 382.0378, found 382.0370. **M.p.** = 132-134 °C.

*6-fluoro-3-(4-methoxybenzyl)-2-methylnaphthalene-1,4-dione (**A-a-16**):* According to the general procedure A, 4-methoxyphenylacetic acid (2.0 equiv.) was used. **A-a-16** was isolated by purification by flash chromatography on silica gel (Toluene/Cyclohexane, 8/2, v/v, UV) as a yellow solid (129 mg, 79% yield). **^{1}H NMR** ($CDCl_3$, 400 MHz): δ 8.08 (dd, J = 8.6, 5.3 Hz, 1H), 7.69 (dd, J = 8.6, 2.7 Hz, 1H), 7.31 (td, J = 8.4, 2.7 Hz, 1H), 7.14 (d, J = 6.6 Hz, 2H), 6.82-6.76 (m, 2H), 3.94 (s, 2H), 3.74 (s, 3H), 2.25 (s, 3H). **^{13}C {^{1}H} NMR** ($CDCl_3$, 101 MHz): δ 184.1, 183.6 (d, $^4J_{C-F}$ = 1.0 Hz), 166.0 (d, $^1J_{C-F}$ = 257.6 Hz), 158.3, 145.8 (d, $^5J_{C-F}$ = 2.0 Hz), 144.3, 134.6 (d, $^3J_{C-F}$ = 7.1 Hz), 129.8, 129.7 (2C), 129.5 (d, $^3J_{C-F}$ = 9.1 Hz), 128.7 (d, $^3J_{C-F}$ = 3.0 Hz), 120.6 (d, $^2J_{C-F}$ = 23.2 Hz), 114.1 (2C), 113.1 (d, $^2J_{C-F}$ = 23.2 Hz), 55.2, 31.6, 13.3. **^{19}F NMR** ($CDCl_3$, 377 MHz): δ -102.62 (td, J = 8.4, 5.3 Hz). **HRMS (ESI+)** m/z: [M+Na]$^+$ calculated for$C_{19}H_{15}FNaO_3$: 333.089743, found 333.089190. **M.p.** = 102-104 °C.

*6-fluoro-3-(3-methoxybenzyl)-2-methylnaphthalene-1,4-dione (**A-a-17**):* According to the general procedure A, 3-methoxyphenylacetic acid (2.0 equiv.) was used. **A-a-17** was isolated by purification by flash chromatography on silica gel (Toluene/Cyclohexane, 8/2, v/v, UV) as a yellow solid (97 mg, 59% yield). **^{1}H NMR** ($CDCl_3$, 400 MHz): δ 8.10 (dd, J = 8.6, 5.3 Hz, 1H), 7.70 (dd, J = 8.6, 2.7 Hz, 1H), 7.33 (td, J = 8.3, 2.7 Hz, 1H), 7.18 (t, J = 7.9 Hz, 1H), 6.83-6.69 (m, 3H), 3.99 (s, 2H), 3.76 (s, 3H), 2.24 (s, 3H). **^{13}C {^{1}H} NMR** ($CDCl_3$, 101 MHz): δ 184.0, 183.6 (d, $^4J_{C-F}$ = 2.0 Hz), 166.0 (d, $^1J_{C-F}$ = 257.6 Hz), 159.9, 145.4 (d, $^5J_{C-F}$ = 2.0 Hz), 144.9, 139.4, 134.7 (d, $^3J_{C-F}$ = 8.1 Hz), 129.7, 129.6 (d, $^3J_{C-F}$ = 8.1 Hz), 128.8 (d, $^3J_{C-F}$ = 3.0 Hz), 121.0, 120.7 (d, $^2J_{C-F}$ = 22.2 Hz), 114.8, 113.1 (d, $^2J_{C-F}$ = 23.2 Hz), 111.5, 55.2, 32.5, 13.4. **^{19}F NMR** ($CDCl_3$, 377 MHz): δ -102.57 (td, J = 8.5, 5.3 Hz). **HRMS (ESI+)** m/z: [M+K]$^+$ calculated for $C_{19}H_{15}FKO_3$: 349.063681, found 349.063210. **M.p.** = 85-87 °C.

*6-fluoro-3-(2-methoxybenzyl)-2-methylnaphthalene-1,4-dione (**A-a-18**):* According to the general procedure A, 2-methoxyphenylacetic acid (2.0 equiv.) was used. **A-a-18** was isolated by purification by flash chromatography on silica gel (Toluene/Cyclohexane, 7/3, v/v, UV) as a yellow solid (112 mg, 69% yield). **^{1}H NMR** ($CDCl_3$, 400 MHz): δ 8.11 (dd, J = 8.6, 5.3 Hz, 1H), 7.71 (dd, J = 8.6, 2.6 Hz, 1H), 7.33 (td, J = 8.4, 2.7 Hz, 1H), 7.17 (ddd, J = 8.2, 7.4, 1.7 Hz, 1H), 7.05 (ddd, J = 7.1, 1.8, 0.8 Hz, 1H), 6.88-6.79 (m, 2H), 4.00 (s, 2H), 3.83 (s, 3H), 2.18 (s, 3H). **^{13}C {^{1}H} NMR** ($CDCl_3$, 101 MHz): δ 184.1, 183.5 (d, $^4J_{C-F}$ = 1.0 Hz), 165.9 (d, $^1J_{C-F}$ = 257.6 Hz), 157.2, 145.8 (d, $^5J_{C-F}$ = 2.0 Hz), 145.1, 134.9 (d, $^3J_{C-F}$ = 8.1 Hz), 129.53 (d, $^3J_{C-F}$ = 9.1 Hz), 129.46, 128.8 (d, $^3J_{C-F}$ = 3.0 Hz), 127.7, 126.1, 120.6, 120.5 (d, $^2J_{C-F}$ = 22.2 Hz), 113.1 (d, $^2J_{C-F}$ = 23.2 Hz), 110.4, 55.4, 27.0, 13.1. **^{19}F NMR** ($CDCl_3$, 377 MHz): δ -102.83 (td, J = 8.2, 5.3 Hz). **HRMS (ESI+)** m/z: [M+Na]$^+$ calculated for $C_{19}H_{15}FNaO_3$: 333.089743, found 333.090114. **M.p.** = 100-102 °C.

*3-(3,4-dimethoxybenzyl)-6-fluoro-2-methylnaphthalene-1,4-dione (**A-a-19**):* According to the general procedure A, 2,3-dimethoxyphenylacetic acid (1.5 equiv.) was used. **A-a-19** was isolated by purification by flash chromatography on silica gel (Toluene, UV) as a yellow solid (93 mg, 52% yield). **^{1}H NMR** ($CDCl_3$, 400 MHz): δ 8.08 (dd, J = 8.6, 5.3 Hz, 1H), 7.69 (dd, J = 8.6, 2.6 Hz, 1H), 7.32 (td, J = 8.3, 2.7 Hz, 1H), 6.78 (d, J = 1.6 Hz, 1H), 6.77-6.68 (m, 2H), 3.93 (s, 2H), 3.83 (s, 3H), 3.80 (s, 3H), 2.25 (s, 3H). **^{13}C {^{1}H} NMR** ($CDCl_3$, 101 MHz): δ 184.1, 183.7 (d, $^4J_{C-F}$ = 1.4 Hz), 166.0 (d, $^1J_{C-F}$ = 256.6 Hz), 149.1, 147.8, 145.6, 144.4, 134.6 (d, $^3J_{C-F}$ = 7.8 Hz), 130.3, 129.6 (d, $^3J_{C-F}$ = 8.8 Hz), 128.7 (d, $^4J_{C-F}$ = 3.2 Hz), 120.7 (d, $^2J_{C-F}$ = 22.5 Hz), 120.5, 113.2 (d, $^2J_{C-F}$ = 23.4 Hz), 112.3, 111.4, 55.9 (2C), 32.1, 13.3. **^{19}F NMR** ($CDCl_3$, 377 MHz): δ -102.58 (td, J = 8.3, 5.3 Hz). **HRMS (ESI+)** m/z: [M+H]$^+$ calculated for $C_{20}H_{18}FO_4$: 341.118364, found 341.117769. **M.p.** = 134-135 °C.

*3-(2,4-dimethoxybenzyl)-6-fluoro-2-methylnaphthalene-1,4-dione (**A-a-20**):* According to the general procedure A, 2,4-dimethoxyphenylacetic acid (2.0 equiv.) was used. **A-a-20** was isolated by purification by flash chromatography on silica gel (Toluene/Cyclohexane, 8/2, v/v, UV) as an orange solid (127 mg, 71% yield). **^{1}H NMR** ($CDCl_3$, 400 MHz): δ 8.09 (dd, J = 8.6, 5.3 Hz, 1H), 7.69 (dd, J = 8.6, 2.6 Hz, 1H), 7.31 (td, J = 8.3, 2.7 Hz, 1H), 6.96 (d, J = 8.3 Hz, 1H), 6.42 (d, J = 2.4 Hz, 1H), 6.36 (dd, J = 8.4, 2.4 Hz, 1H), 3.90 (s, 2H), 3.79 (s, 3H), 3.74 (s, 3H), 2.18 (s, 3H). **^{13}C {^{1}H} NMR** ($CDCl_3$, 101 MHz): δ 184.2, 183.6 (d, $^4J_{C-F}$ = 1.0 Hz), 165.9 (d, $^1J_{C-F}$ = 257.6 Hz), 159.6, 158.1, 146.0 (d, $^5J_{C-F}$ = 2.0 Hz), 144.9, 134.9 (d, $^3J_{C-F}$ = 8.1 Hz), 130.0, 129.5 (d, $^3J_{C-F}$ = 9.1 Hz), 128.8 (d, $^3J_{C-F}$ = 3.0 Hz), 120.4 (d, $^2J_{C-F}$ = 22.2 Hz), 118.4, 113.1 (d, $^2J_{C-F}$ = 24.2 Hz), 104.1, 98.6, 55.3 (2C), 26.4, 13.0. **^{19}F NMR** ($CDCl_3$, 377 MHz): δ -102.94 (td, J = 8.3, 5.3 Hz). **HRMS (ESI+)** m/z: [M+K]$^+$ calculated for $C_{20}H_{17}FKO_4$: 379.074245, found 379.074459. **M.p.** = 100-102 °C.

*3-(benzo[d][1,3]dioxol-5-ylmethyl)-6-fluoro-2-methylnaphthalene-1,4-dione (**A-a-21**):* According to the general procedure A, 3,4-methylenedioxyphenylacetic acid (2.0 equiv.) was used. **A-a-21** was isolated

by purification by flash chromatography on silica gel (Toluene, then Cyclohexane/Ethyl acetate, 9/1, v/v, UV) as an orange solid (82 mg, 48% yield). **^{1}H NMR** ($CDCl_3$, 400 MHz): δ 8.09 (dd, *J* = 8.6, 5.3 Hz, 1H), 7.69 (dd, *J* = 8.6, 2.6 Hz, 1H), 7.33 (td, *J* = 8.3, 2.7 Hz, 1H), 6.72-6.63 (m, 3H), 5.88 (s, 2H), 3.91 (s, 2H), 2.24 (s, 3H). **^{13}C {^{1}H} NMR** ($CDCl_3$, 101 MHz): δ 184.1, 183.7 (d, $^4J_{C-F}$ = 1.0 Hz), 166.0 (d, $^1J_{C-F}$ = 257.0 Hz), 147.9, 146.3, 145.5 (d, $^5J_{C-F}$ = 2.0 Hz), 144.6, 134.7 (d, $^3J_{C-F}$ = 7.1 Hz), 131.5, 129.6 (d, $^3J_{C-F}$ = 9.1 Hz), 128.8 (d, $^3J_{C-F}$ = 3.0 Hz), 121.6, 120.7 (d, $^2J_{C-F}$ = 22.2 Hz), 113.2 (d, $^2J_{C-F}$ = 23.2 Hz), 109.2, 108.4, 101.1, 32.2, 13.3. **^{19}F NMR** ($CDCl_3$, 377 MHz): δ -102.54 (td, *J* = 8.4, 5.3 Hz). **HRMS (ESI+)** *m/z*: $[M+H]^+$ calculated for $C_{19}H_{14}FO_4$: 325.087064, found 325.087288. **M.p.** = 110-112 °C.

3-((2,3-dihydrobenzo[b][1,4]dioxin-6-yl)methyl)-6-fluoro-2-methylnaphthalene-1,4-dione (***A-a-22***): According to the general procedure A, 2-(2,3-dihydro-1,4-benzodioxin-6-yl)acetic acid (2.0 equiv.) was used. **A-a-22** was isolated by purification by flash chromatography on silica gel (Toluene, UV) as a yellow solid (140 mg, 79% yield). **^{1}H NMR** ($CDCl_3$, 400 MHz): δ 8.09 (dd, *J* = 7.9, 5.4 Hz, 1H), 7.70 (d, *J* = 7.3 Hz, 1H), 7.33 (t, *J* = 7.3 Hz, 1H), 6.78-6.64 (m, 3H), 4.19 (s, 4H), 3.90 (s, 2H), 2.24 (s, 3H). **^{13}C {^{1}H} NMR** ($CDCl_3$, 101 MHz): δ 184.1, 183.6 (d, $^4J_{C-F}$ = 1.1 Hz), 166.0 (d, $^1J_{C-F}$ = 256.5 Hz), 145.6 (d, $^5J_{C-F}$ = 1.7 Hz), 144.5, 143.6, 142.3, 134.7 (d, $^3J_{C-F}$ = 7.8 Hz), 131.0, 129.6 (d, $^3J_{C-F}$ = 8.8 Hz), 128.8 (d, $^4J_{C-F}$ = 3.3 Hz), 121.7, 120.7 (d, $^2J_{C-F}$ = 22.6 Hz), 117.4, 117.2, 113.2 (d, $^2J_{C-F}$ = 23.4 Hz), 64.4, 64.3, 31.7, 13.3. **^{19}F NMR** ($CDCl_3$, 377 MHz): δ -102.63 (q, *J* = 7.8 Hz). **HRMS (ESI+)** *m/z*: $[M+K]^+$ calculated for $C_{20}H_{15}FKO_4$: 377.058595 found 377.058159. **M.p.** = 99-101 °C.

3-((2,2-dimethylbenzo[d][1,3]dioxol-5-yl)methyl)-6-fluoro-2-methylnaphthalene-1,4-dione (***A-a-23***): According to the general procedure A, 2-(2,2-dimethylbenzo[*d*][1,3]dioxol-5-yl)acetic acid (2.0 equiv.) was used. **A-a-23** was isolated by purification by flash chromatography on silica gel (Toluene/Cyclohexane, gradient from 6/4 to 7/3, v/v, UV) as an orange solid (99 mg, 53% yield). **^{1}H NMR** ($CDCl_3$, 400 MHz): δ 8.12 (dd, *J* = 8.6, 5.3 Hz, 1H), 7.73 (dd, *J* = 8.6, 2.6 Hz, 1H), 7.35 (td, *J* = 8.3, 2.7 Hz, 1H), 6.67-6.56 (m, 3H), 3.92 (s, 2H), 2.25 (s, 3H), 1.63 (s, 6H). **^{13}C {^{1}H} NMR** ($CDCl_3$, 101 MHz): δ 184.2, 183.7, 166.1 (d, $^1J_{C-F}$ = 256.6 Hz), 147.8, 146.2, 145.7, 144.5, 134.7 (d, $^3J_{C-F}$ = 7.9 Hz), 130.8, 129.6 (d, $^3J_{C-F}$ = 8.9 Hz), 128.8 (d, $^4J_{C-F}$ = 3.4 Hz), 121.1, 120.7 (d, $^2J_{C-F}$ = 22.7 Hz), 118.0, 113.2 (d, $^2J_{C-F}$ = 23.4 Hz), 108.9, 108.3, 32.2, 26.0 (2C), 13.4. **^{19}F NMR** ($CDCl_3$, 377 MHz): δ -102.64 (td, *J* = 8.5, 5.3 Hz). **HRMS (APCI+)** *m/z*: $[M]^+$ calculated for $C_{21}H_{17}FO_4$: 352.1105, found 352.1093. **M.p.** = 132-134 °C.

3-((2,2-difluorobenzo[d][1,3]dioxol-5-yl)methyl)-6-fluoro-2-methylnaphthalene-1,4-dione (***A-a-24***): According to the general procedure A, 2-(2,2-difluoro-2*H*-1,3-benzodioxol-5-yl)acetic acid (1.5 equiv.) was used. **A-a-24** was isolated by purification by flash chromatography on silica gel (Toluene/Cyclohexane, 7/3, v/v, UV) as a yellow solid (69 mg, 78% yield). **^{1}H NMR** ($CDCl_3$, 400 MHz): δ 8.13 (dd, *J* = 8.5, 5.2 Hz, 1H), 7.71 (dd, *J* = 8.5, 2.5 Hz, 1H), 7.36 (td, *J* = 8.3, 2.5 Hz, 1H), 6.95 (s, 1H), 6.94 (s, 2H), 4.00 (s, 2H), 2.26 (s, 3H). **^{13}C {^{1}H} NMR** ($CDCl_3$, 101 MHz): δ 183.9, 183.6 (d, $^4J_{C-F}$ = 1.3 Hz), 166.1 (d, $^1J_{C-F}$ = 257.1 Hz), 145.0, 144.9 (d, $^5J_{C-F}$ = 1.8 Hz), 144.1, 142.5, 134.6 (d, $^3J_{C-F}$ = 7.9 Hz), 134.0, 131.7 (t, $^1J_{C-F}$ = 255.1 Hz), 129.8 (d, $^3J_{C-F}$ = 8.9 Hz), 128.8 (d, $^4J_{C-F}$ = 3.2 Hz), 123.7, 121.0 (d, $^2J_{C-F}$ = 22.5 Hz), 113.3 (d, $^2J_{C-F}$ = 23.5 Hz), 110.0, 109.5, 32.4, 13.4. **^{19}F NMR** ($CDCl_3$, 377 MHz): δ -50.02, -102.19 (td, *J* = 8.3, 5.3 Hz). **HRMS (APCI+)** *m/z*: $[M]^+$ calculated for $C_{19}H_{11}F_3O_4$: 360.0604, found 360.0588. **M.p.** = 92-94 °C.

6-fluoro-2-methyl-3-(2,3,4-trimethoxybenzyl)naphthalene-1,4-dione (***A-a-25***): According to the general procedure A, 2,3,4-trimethoxyphenylacetic acid (2.0 equiv.) was used. **A-a-25** was isolated by purification by flash chromatography on silica gel (Toluene/Cyclohexane, gradient from 8/2 to 10/0, v/v, UV) as a yellow solid (156 mg, 80% yield). **^{1}H NMR** ($CDCl_3$, 400 MHz): δ 8.06 (dd, *J* = 8.6, 5.3 Hz, 1H), 7.66 (dd, *J* = 8.6, 2.7 Hz, 1H), 7.30 (td, *J* = 8.3, 2.7 Hz, 1H), 6.70 (d, *J* = 8.6 Hz, 1H), 6.52 (d, *J* = 8.6 Hz, 1H), 3.97-3.72 (m, 11H), 2.14 (s, 3H). **^{13}C {^{1}H} NMR** ($CDCl_3$, 101 MHz): δ 184.0, 183.4 (d, $^4J_{C-F}$ = 1.0 Hz), 165.8 (d, $^1J_{C-F}$ = 257.6 Hz), 152.5, 151.7, 145.9 (d, $^5J_{C-F}$ = 2.0 Hz), 144.7, 142.3, 134.8 (d, $^3J_{C-F}$ = 8.1 Hz), 129.5 (d, $^3J_{C-F}$ = 8.1 Hz), 128.8 (d, $^3J_{C-F}$ = 3.0 Hz), 123.8, 123.5, 120.5 (d, $^2J_{C-F}$ = 22.2 Hz), 113.0 (d, $^2J_{C-F}$ = 24.2 Hz), 107.2, 60.72, 60.67, 55.9, 26.7, 13.0. **^{19}F NMR** ($CDCl_3$, 377 MHz): δ -102.81 (td, *J* = 8.2, 5.1 Hz). **HRMS (ESI+)** *m/z*: $[M + H]^+$ calculated for $C_{21}H_{20}FO_5$: 371.128928, found 371.127202. **M.p.** = 86-87 °C.

3-(2,5-dimethoxybenzyl)-6-fluoro-2-methylnaphthalene-1,4-dione (***A-a-26***): According to the general procedure A, 2,5-dimethoxyphenylacetic acid (2.0 equiv.) was used. **A-a-26** was isolated by purification by flash chromatography on silica gel (Toluene/Cyclohexane, gradient from 9/1 to 10/0, v/v, UV) as a yellow solid (112 mg, 63% yield). **^{1}H NMR** ($CDCl_3$, 400 MHz): δ 8.12 (dd, *J* = 8.6, 5.3 Hz, 1H), 7.72 (dd, *J* = 8.6, 2.6 Hz, 1H), 7.35 (td, *J* = 8.3, 2.6 Hz, 1H), 6.78 (d, *J* = 8.8 Hz, 1H), 6.69 (dd, *J* = 8.8, 3.0 Hz, 1H), 6.61 (d, *J* = 3.0 Hz, 1H), 3.98 (s, 2H), 3.79 (s, 3H), 3.70 (s, 3H), 2.17 (s, 3H). **^{13}C {^{1}H} NMR** ($CDCl_3$, 101 MHz): δ

184.0, 183.4 (d, $^{4}J_{C-F}$ = 1.0 Hz), 165.9 (d, $^{1}J_{C-F}$ = 257.6 Hz), 153.6, 151.5, 146.6 (d, $^{5}J_{C-F}$ = 1.0 Hz), 145.2, 134.8 (d, $^{3}J_{C-F}$ = 7.1 Hz), 129.5 (d, $^{3}J_{C-F}$ = 8.1 Hz), 128.8 (d, $^{3}J_{C-F}$ = 3.0 Hz), 127.4, 120.5 (d, $^{2}J_{C-F}$ = 23.2 Hz), 116.3, 113.1 (d, $^{2}J_{C-F}$ = 23.3 Hz), 111.2, 111.0, 55.9, 55.6, 26.9, 13.0. **^{19}F NMR** ($CDCl_3$, 377 MHz): δ -102.84 (td, J = 8.3, 5.3 Hz). **HRMS (ESI+)** *m/z*: $[M+H]^+$ calculated for $C_{20}H_{18}FO_4$: 341.118364, found 341.117365. **M.p.** = 97-99 °C.

*6-fluoro-2-methyl-3-(3,4,5-trimethoxybenzyl)naphthalene-1,4-dione (**A-a-27**):* According to the general procedure A, 3,4,5-trimethoxyphenylacetic acid (2.0 equiv.) was used. **A-a-27** was isolated by purification by flash chromatography on silica gel (Cyclohexane/Ethyl Acetate, 9/1, v/v, UV) as a yellow solid (166 mg, 85% yield). **^{1}H NMR** ($CDCl_3$, 400 MHz): δ 8.24 (dd, J = 8.6, 5.3 Hz, 1H), 7.85 (dd, J = 8.6, 2.7 Hz, 1H), 7.48 (td, J = 8.3, 2.7 Hz, 1H), 6.59 (s, 2H), 4.08 (d, J = 8.4 Hz, 2H), 3.94 (d, J = 9.2 Hz, 9H), 2.42 (s, 3H). **^{13}C {^{1}H} NMR** ($CDCl_3$, 101 MHz): δ 184.0, 183.7, 166.0 (d, $^{1}J_{C-F}$ = 257.6 Hz), 153.4 (2C), 145.3 (d, $^{5}J_{C-F}$ = 1.0 Hz), 144.6, 136.8, 134.6 (d, $^{3}J_{C-F}$ = 8.1 Hz), 133.4, 129.0 (d, $^{3}J_{C-F}$ = 8.1 Hz), 128.7 (d, $^{3}J_{C-F}$ = 3.0 Hz), 120.8 (d, $^{2}J_{C-F}$ = 23.2 Hz), 113.3 (d, $^{2}J_{C-F}$ = 24.2 Hz), 105.9 (2C), 60.8, 56.2 (2C), 32.7, 13.4. **^{19}F NMR** ($CDCl_3$, 377 MHz): δ -102.46 (td, J = 8.5, 4.9 Hz). **HRMS (ESI+)** *m/z*: $[M+H]^+$ calculated for $C_{21}H_{20}FO_5$: 371.128928, found 371.127247. **M.p.** = 122-124 °C.

*6-fluoro-2-methyl-3-(thiophen-2-ylmethyl)naphthalene-1,4-dione (**A-a-28**):* According to the general procedure A, 2-thiopheneacetic acid (1.5 equiv.) was used. **A-a-28** was isolated by purification by flash chromatography on silica gel (Toluene/Cyclohexane, 8/2, v/v, UV) as a yellow solid (70 mg, 46% yield). **^{1}H NMR** ($CDCl_3$, 400 MHz): δ 8.10 (dd, J = 8.6, 5.3 Hz, 1H), 7.73 (dd, J = 8.6, 2.6 Hz, 1H), 7.34 (td, J = 8.3, 2.7 Hz, 1H), 7.11 (dd, J = 4.9, 1.4 Hz, 1H), 6.92-6.85 (m, 2H), 4.17 (s, 2H), 2.29 (s, 3H). **^{13}C {^{1}H} NMR** ($CDCl_3$, 101 MHz): δ 184.1, 183.2 (d, $^{4}J_{C-F}$ = 1.4 Hz), 166.1 (d, $^{1}J_{C-F}$ = 256.9 Hz), 144.6 (d, $^{5}J_{C-F}$ = 1.9 Hz), 144.4, 139.7, 134.6 (d, $^{3}J_{C-F}$ = 7.9 Hz), 129.7 (d, $^{3}J_{C-F}$ = 8.8 Hz), 128.8 (d, $^{4}J_{C-F}$ = 3.2 Hz), 127.0, 125.9, 124.3, 120.8 (d, $^{2}J_{C-F}$ = 22.6 Hz), 113.3 (d, $^{2}J_{C-F}$ = 23.2 Hz), 27.1, 13.2. **^{19}F NMR** ($CDCl_3$, 377 MHz): δ -102.36 (td, J = 8.3, 5.3 Hz). **HRMS (ESI+)** *m/z*: $[M+H]^+$ calculated for $C_{16}H_{12}FO_2S$: 287.053655, found 287.054700. **M.p.** = 60-62 °C.

*3-(cyclohexylmethyl)-6-fluoro-2-methylnaphthalene-1,4-dione (**A-a-29**):* According to the general procedure A, cyclohexylacetic acid (2.0 equiv.) was used. **A-a-29** was isolated by purification by flash chromatography on silica gel (Toluene/cyclohexane, 8/2, v/v, UV) as a yellow solid (77 mg, 51% yield). **^{1}H NMR** ($CDCl_3$, 400 MHz): δ 8.09 (dd, J = 8.6, 5.3 Hz, 1H), 7.69 (dd, J = 8.7, 2.6 Hz, 1H), 7.32 (td, J = 8.3, 2.7 Hz, 1H), 2.54 (d, J = 7.2 Hz, 2H), 2.17 (s, 3H), 1.70-1.58 (m, 5H), 1.54 (ddt, J = 14.3, 7.0, 3.4 Hz, 1H), 1.20-1.10 (m, 3H), 1.08-0.99 (m, 2H). **^{13}C {^{1}H} NMR** ($CDCl_3$, 101 MHz): δ 184.1, 184.0 (d, $^{4}J_{C-F}$ = 2.0 Hz), 166.0 (d, $^{1}J_{C-F}$ = 260.6 Hz), 146.9 (d, $^{5}J_{C-F}$ = 1.0 Hz), 144.4, 134.9 (d, $^{3}J_{C-F}$ = 8.1 Hz), 130.1 (d, $^{3}J_{C-F}$ = 9.1 Hz), 128.9 (d, $^{3}J_{C-F}$ = 3.0 Hz), 120.6 (d, $^{2}J_{C-F}$ = 22.2 Hz), 113.5 (d, $^{2}J_{C-F}$ = 24.2 Hz), 38.5, 34.8, 33.8 (2C), 26.43 (2C), 26.38, 13.5. **^{19}F NMR** ($CDCl_3$, 377 MHz): δ -103.04 (td, J = 8.4, 5.3 Hz). **HRMS (ESI+)** *m/z*: $[M+H]^+$ calculated for $C_{18}H_{20}FO_2$: 287.144185, found 287.144119. **M.p.** = 90-91 °C.

*3-((7-fluoro-3-methyl-1,4-dioxo-1,4-dihydronaphthalen-2-yl)methyl)benzonitrile (**B-a-30**):* According to the general procedure B, 3-cyanobenzyl bromide was used. **B-a-30** was isolated by purification by flash chromatography on silica gel (Toluene/Cyclohexane, gradient from 8/2 to 10/0, v/v, UV) as a yellow solid (65 mg, 43% yield). **^{1}H NMR** ($CDCl_3$, 400 MHz): δ 8.12 (dd, J = 8.6, 5.2 Hz, 1H), 7.69 (dd, J = 8.5, 2.6 Hz, 1H), 7.51-7.45 (m, 3H), 7.41-7.33 (m, 2H), 4.04 (s, 2H), 2.24 (s, 3H). **^{13}C {^{1}H} NMR** ($CDCl_3$, 101 MHz): 183.7, 183.4 (d, $^{4}J_{C-F}$ = 1.0 Hz), 166.1 (d, $^{1}J_{C-F}$ = 258.6 Hz), 145.4, 144.1 (d, $^{5}J_{C-F}$ = 2.0 Hz), 139.5, 134.4 (d, $^{3}J_{C-F}$ = 8.1 Hz), 133.2, 132.1, 130.5, 129.8 (d, $^{3}J_{C-F}$ = 9.1 Hz), 129.6, 128.7 (d, $^{4}J_{C-F}$ = 4.0 Hz), 121.1 (d, $^{2}J_{C-F}$ = 22.2 Hz), 118.7, 113.3 (d, $^{2}J_{C-F}$ = 24.2 Hz), 112.9, 32.2, 13.5. **^{19}F NMR** ($CDCl_3$, 377 MHz): δ -102.04 (td, J = 8.3, 5.3 Hz). **HRMS (ESI+)** *m/z*: $[M+K]^+$ calculated for $C_{19}H_{12}FKNO_2$: 344.048365, found 344.049085. **M.p.** = 138-139 °C.

*3-benzyl-6-fluoro-2-methylnaphthalene-1,4-dione (**B-a-31**):* According to the general procedure B, benzyl bromide was used. **B-a-31** was isolated by purification by flash chromatography on silica gel (Toluene/Cyclohexane, 7/3, v/v, UV) as a yellow solid (94 mg, 67% yield). **^{1}H NMR** ($CDCl_3$, 400 MHz): δ 8.12 (dd, J = 8.6, 5.3 Hz, 1H), 7.72 (dd, J = 8.6, 2.7 Hz, 1H), 7.35 (td, J = 8.3, 2.7 Hz, 1H), 7.30-7.16 (m, 5H), 4.02 (s, 2H), 2.25 (s, 3H). **^{13}C {^{1}H} NMR** ($CDCl_3$, 101 MHz): δ 184.2, 183.6 (d, $^{4}J_{C-F}$ = 1.0 Hz), 166.1 (d, $^{1}J_{C-F}$ = 263.0 Hz), 145.6 (d, $^{5}J_{C-F}$ = 2.0 Hz), 144.8, 137.9, 134.7 (d, $^{3}J_{C-F}$ = 11.1 Hz), 129.7 (d, $^{3}J_{C-F}$ = 9.1 Hz), 128.9, 128.8 (2C), 128.7 (2C), 126.7, 120.7 (d, $^{2}J_{C-F}$ = 22.2 Hz), 113.3 (d, $^{2}J_{C-F}$ = 23.2 Hz), 32.6, 13.4. **^{19}F NMR** ($CDCl_3$, 377 MHz): δ -102.57 (td, J = 8.6, 5.3 Hz). **HRMS (ESI+)** *m/z*: $[M+H]^+$ calculated for $C_{18}H_{14}FO_2$: 281.097234, found 281.096747. **M.p.** = 88-90 °C.

*6-fluoro-3-(3-fluoro-4-nitrobenzyl)-2-methylnaphthalene-1,4-dione (**B-a-32**):* According to the general procedure B, 3-fluoro-4-nitrobenzyl bromide was used. **B-a-32** was isolated by purification by flash chromatography on silica gel (Cyclohexane/Ethyl acetate, gradient from 9/1 to 8/2 then Toluene/Cyclohexane, 3/2, v/v, UV) as a yellow solid (62 mg, 30% yield). **^{1}H NMR** ($CDCl_3$, 400 MHz): δ 8.13 (dd, J = 8.6, 5.2 Hz, 1H), 7.97 (dd, J = 8.7, 7.7 Hz, 1H), 7.70 (dd, J = 8.5, 2.7 Hz, 1H), 7.38 (td, J = 8.3, 2.7 Hz, 1H), 7.19-7.11 (m, 2H), 4.08 (s, 2H), 2.26 (s, 3H). **^{13}C {^{1}H} NMR** ($CDCl_3$, 101 MHz): δ 183.5, 183.0 (d, $^4J_{C-F}$ = 2.0 Hz), 166.2 (d, $^1J_{C-F}$ = 258.6 Hz), 155.7 (d, $^1J_{C-F}$ = 266.6 Hz), 147.4 (d, $^3J_{C-F}$ = 8.1 Hz), 145.9, 143.3 (d, $^5J_{C-F}$ = 2.0 Hz), 134.4 (d, $^3J_{C-F}$ = 8.1 Hz), 130.0 (d, $^3J_{C-F}$ = 9.1 Hz), 129.1, 128.7 (d, $^4J_{C-F}$ = 3.0 Hz), 126.5 (d, $^4J_{C-F}$ = 3.0 Hz), 124.8 (d, $^4J_{C-F}$ = 3.0 Hz), 121.2 (d, $^2J_{C-F}$ = 22.2 Hz), 118.4 (d, $^2J_{C-F}$ = 21.2 Hz), 113.4 (d, $^2J_{C-F}$ = 23.2 Hz), 32.6, 13.6. **^{19}F NMR** ($CDCl_3$, 377 MHz): δ -101.75 (td, J = 8.2, 4.9 Hz), -116.56 (dd, J = 11.7, 7.6 Hz). **HRMS (ESI+)** *m/z*: $[M+H]^+$ calculated for $C_{18}H_{12}F_2NO_4$: 344.072891, found 344.070588. **M.p.** = 148-150 °C.

*6-fluoro-3-(4-fluoro-3-iodobenzyl)-2-methylnaphthalene-1,4-dione (**B-a-33**):* According to the general procedure B, 3-fluoro-4-nitrobenzyl bromide was used. This reaction was realized with 1.058 mmol (200 mg) of 6-fluoro-menadione. **B-a-33** was isolated by purification by flash chromatography on silica gel (Toluene/Cyclohexane, 6/4, v/v, UV) as a yellow solid (160 mg, 36% yield). **^{1}H NMR** ($CDCl_3$, 400 MHz): δ 8.13 (dd, J = 8.6, 5.3 Hz, 1H), 7.72 (dd, J = 8.5, 2.6 Hz, 1H), 7.59 (dd, J = 5.9, 2.0 Hz, 1H), 7.37 (td, J = 8.3, 2.6 Hz, 1H), 7.16 (ddd, J = 7.1, 4.7, 2.1 Hz, 1H), 6.95 (t, J = 8.1 Hz, 1H), 3.95 (s, 2H), 2.25 (s, 3H). **^{13}C {^{1}H} NMR** ($CDCl_3$, 101 MHz): δ 184.0, 183.5 (d, $^4J_{C-F}$ = 1.4 Hz), 166.2 (d, $^1J_{C-F}$ = 257.1 Hz), 160.7 (d, $^1J_{C-F}$ = 244.8 Hz), 145.1, 144.7 (d, $^5J_{C-F}$ = 1.5 Hz), 139.3 (d, $^4J_{C-F}$ = 1.5 Hz), 135.7 (d, $^4J_{C-F}$ = 3.7 Hz), 134.6 (d, $^3J_{C-F}$ = 7.9 Hz), 130.3 (d, $^3J_{C-F}$ = 7.2 Hz), 129.8 (d, $^3J_{C-F}$ = 8.9 Hz), 128.8 (d, $^4J_{C-F}$ = 3.3 Hz), 121.0 (d, $^2J_{C-F}$ = 22.6 Hz), 115.8 (d, $^2J_{C-F}$ = 24.0 Hz), 113.4 (d, $^2J_{C-F}$ = 23.5 Hz), 81.7 (d, $^2J_{C-F}$ = 25.7 Hz), 31.4, 13.5. **^{19}F NMR** ($CDCl_3$, 377 MHz): δ -96.86--96.94 (m), -102.19 (td, J = 8.3, 5.3 Hz). **HRMS (APCI+)** *m/z*: $[M]^+$ calculated for $C_{18}H_{11}F_2IO_2$: 423.9766, found 423.9742. **M.p.** = 124-126 °C.

*3-(4-bromo-2-(trifluoromethyl)benzyl)-6-fluoro-2-methylnaphthalene-1,4-dione (**B-a-34**):* According to the general procedure B, 4-bromo-1-(bromomethyl)-2-trifluoromethyl benzene was used. **B-a-34** was isolated by purification by flash chromatography on silica gel (Toluene/Cyclohexane, 4/6, v/v, UV) as a yellow solid (46 mg, 22% yield). **^{1}H NMR** ($CDCl_3$, 400 MHz): δ 8.17 (dd, J = 8.6, 5.2 Hz, 1H), 7.82 (d, J = 2.2 Hz, 1H), 7.72 (dd, J = 8.5, 2.7 Hz, 1H), 7.48 (dd, J = 8.3, 2.2 Hz, 1H), 7.40 (td, J = 8.3, 2.7 Hz, 1H), 6.82 (d, J = 8.3 Hz, 1H), 4.15 (s, 2H), 2.10 (s, 3H). **^{13}C {^{1}H} NMR** ($CDCl_3$, 101 MHz): δ 183.6, 183.3 (d, $^4J_{C-F}$ = 1.5 Hz), 166.2 (d, $^1J_{C-F}$ = 257.4 Hz), 146.9, 143.8, 135.6 (d, $^4J_{C-F}$ = 1.6 Hz), 135.2, 134.5 (d, $^3J_{C-F}$ = 7.9 Hz), 130.3 (q, $^2J_{C-F}$ = 30.8 Hz), 130.03, 130.01 (d, $^3J_{C-F}$ = 8.9 Hz), 129.7 (q, $^3J_{C-F}$ = 6.1 Hz), 128.9 (d, $^3J_{C-F}$ = 3.2 Hz), 123.6 (q, $^1J_{C-F}$ = 274.4 Hz), 121.1 (d, $^2J_{C-F}$ = 22.7 Hz), 120.4, 113.5 (d, $^2J_{C-F}$ = 23.5 Hz), 28.6 (q, $^4J_{C-F}$ = 2.8 Hz), 13.3. **^{19}F NMR** ($CDCl_3$, 377 MHz): δ -61.22, -101.86 (td, J = 8.3, 5.3 Hz). **HRMS (APCI+)** *m/z*: $[M]^+$ calculated for $C_{19}H_{11}BrF_4O_2$: 425.9873, found 425.9877. **M.p.** = 120-121 °C.

*3-(3,5-dimethoxybenzyl)-6-fluoro-2-methylnaphthalene-1,4-dione (**B-a-35**):* According to the general procedure B, 3,5-dimethoxybenzyl bromide was used. **B-a-35** was isolated by purification by flash chromatography on silica gel (Toluene/Cyclohexane, gradient from 5/5 to 7/3, v/v, UV) as a yellow solid (105 mg, 62% yield). **^{1}H NMR** ($CDCl_3$, 400 MHz): δ 8.11 (dd, J = 8.6, 5.3 Hz, 1H), 7.71 (dd, J = 8.6, 2.6 Hz, 1H), 7.34 (td, J = 8.3, 2.6 Hz, 1H), 6.35 (d, J = 2.3 Hz, 2H), 6.29 (t, J = 2.3 Hz, 1H), 3.95 (s, 2H), 3.74 (s, 6H), 2.24 (s, 3H). **^{13}C {^{1}H} NMR** ($CDCl_3$, 101 MHz): δ 184.1, 183.6 (d, $^4J_{C-F}$ = 1.0 Hz), 166.0 (d, $^1J_{C-F}$ = 252.5 Hz), 161.1 (2C), 145.2 (d, $^5J_{C-F}$ = 2.0 Hz), 145.0, 140.1, 134.7 (d, $^3J_{C-F}$ = 8.1 Hz), 129.7 (d, $^3J_{C-F}$ = 9.1 Hz), 128.8 (d, $^4J_{C-F}$ = 3.0 Hz), 120.7 (d, $^2J_{C-F}$ = 23.2 Hz), 113.3 (d, $^2J_{C-F}$ = 23.2 Hz), 107.0 (2C), 98.1, 55.4 (2C), 32.7, 13.4. **^{19}F NMR** ($CDCl_3$, 377 MHz): δ -102.59 (td, J = 8.3, 5.1 Hz). **HRMS (ESI+)** *m/z*: $[M+H]^+$ calculated for $C_{20}H_{18}FO_4$: 341.118364, found 341.117255. **M.p.** = 102-104 °C.

*6-fluoro-1,4-dimethoxy-2-methylnaphthalene (**a-36**):* 6-fluoromenadione (500 mg, 2.63 mmol, 1.0 equiv.) was suspended in ethanol (10 mL) and was heated to be dissolve. At room temperature, a solution of tin (II) chloride (1.2 g, 6.57 mmol, 2.5 equiv.) in hydrochloric acid (0.9 mL, 10.93 mmol, 4.12 equiv.) was added dropwise. The mixture reaction became brown/black. The solution was stirred 20 minutes and became light yellow. After this time, the solvent was removed under reduced pressure. The resulting mixture was poured into cold water and the precipitate was filtrated, washed with water and dissolve acetone (10 mL). The solution was dried over $MgSO_4$ before the next step. Under argon, to the previous solution was added dimethyl sulfate (1.66 g, 13.15 mmol, 5.0 equiv.) and the resulting mixture was stirred at 60 °C. A solution of potassium hydroxide (737 mg, 13.15 mmol, 5.0 equiv.) in methanol (2.5 mL) was added carefully dropwise (very exothermic). The mixture was stirred overnight

at 60 °C. After cooling down at room temperature, a solution of potassium hydroxide in water (20%) was added to the mixture. Organic solvent was removed under reduced pressure and the resulting aqueous phase was extracted three times with dichloromethane. Organic layer was dried over $MgSO_4$ and concentrated under vacuum. The crude residue was purified by flash chromatography on silica gel (Toluene, UV) to afford the expected product as a beige solid (341 mg, 59% yield). **^{1}H NMR** ($CDCl_3$, 400 MHz): δ 8.07 (dd, J = 9.2, 5.6 Hz, 1H), 7.88 (dd, J = 10.6, 2.7 Hz, 1H), 7.32 (ddd, J = 9.2, 8.3, 2.7 Hz, 1H), 6.64 (s, 1H), 3.95 (s, 3H), 3.89 (s, 3H), 2.48 (s, 3H). **^{13}C {^{1}H} NMR** ($CDCl_3$, 101 MHz): δ 160.4 (d, $^1J_{C-F}$ = 243.9 Hz), 150.9 (d, $^4J_{C-F}$ = 5.1 Hz), 147.1 (d, $^5J_{C-F}$ = 1.4 Hz), 126.1 (d, $^3J_{C-F}$ = 8.8 Hz), 125.8 (d, $^5J_{C-F}$ = 0.8 Hz), 124.8 (d, $^4J_{C-F}$ = 2.5 Hz), 124.2 (d, $^3J_{C-F}$ = 8.7 Hz), 116.3 (d, $^2J_{C-F}$ = 25.2 Hz), 108.0, 106.3 (d, $^2J_{C-F}$ = 22.5 Hz), 61.2, 55.5, 16.1. **^{19}F NMR** ($CDCl_3$, 377 MHz): δ -116.24 (ddd, J = 10.3, 8.4, 5.5 Hz). **HRMS (ESI+)** *m/z*: $[M+H]^+$ calculated for $C_{13}H_{14}FO_2$: 221.097234, found 221.097121. **M.p.** = 52-53 °C.

*3-(chloromethyl)-6-fluoro-1,4-dimethoxy-2-methylnaphthalene (**a-37**):* A solution of **a-36** (250 mg, 1.14 mmol, 1.0 equiv.) and paraformaldehyde (538 mg, 17.03 mmol, 15.0 equiv.) in hydrochloric acid (9 mL) was stirred at 60 °C overnight. The reaction mixture was cooled down, diluted with water, and extracted with ethyl acetate. The organic phase was dried over $MgSO_4$ and concentrated under reduced pressure. The crude residue was purified by flash chromatography on silica gel (Toluene/cyclohexane, 9/1, v/v, UV) to afford the expected product (200 mg, 66%, white solid). **^{1}H NMR** ($CDCl_3$, 400 MHz): δ 8.07 (dd, J = 9.2, 5.5 Hz, 1H), 7.68 (dd, J = 10.2, 2.5 Hz, 1H), 7.29 (ddd, J = 9.2, 8.2, 2.6 Hz, 1H), 4.89 (s, 2H), 4.02 (s, 3H), 3.87 (s, 3H), 2.52 (s, 3H). **^{13}C {^{1}H} NMR** ($CDCl_3$, 101 MHz): δ 161.1 (d, $^1J_{C-F}$ = 246.0 Hz), 150.9 (d, $^4J_{C-F}$ = 5.4 Hz), 150.7 (d, $^5J_{C-F}$ = 1.3 Hz), 128.2 (d, $^3J_{C-F}$ = 8.7 Hz), 128.0, 126.3 (d, $^5J_{C-F}$ = 0.6 Hz), 125.6 (d, $^4J_{C-F}$ = 2.5 Hz), 125.3 (d, $^3J_{C-F}$ = 8.8 Hz), 117.1 (d, $^2J_{C-F}$ = 25.3 Hz), 106.7 (d, $^2J_{C-F}$ = 22.4 Hz), 63.1, 61.6, 38.9, 11.6. **^{19}F NMR** ($CDCl_3$, 377 MHz): δ -114.27 (m). **HRMS (ESI+)** *m/z*: $[M+H]^+$ calculated for $C_{14}H_{15}ClFO_2$: 269.073912, found 269.073883. **M.p.** = 100-102 °C.

*5-((7-fluoro-1,4-dimethoxy-3-methylnaphthalen-2-yl)methyl)benzo[d]thiazole (**a-38**):* According to the general procedure C, 5-benzothiazole boronic acid was used. **a-38** was isolated by purification by flash chromatography on silica gel (Cyclohexane/Ethyl acetate, 7/3 then pentane/diethyl ether, 7/3, v/v, UV) as a colorless oil (114 mg, 83% yield). **^{1}H NMR** ($CDCl_3$, 400 MHz): δ 8.97 (s, 1H), 8.10 (dd, J = 9.2, 5.6 Hz, 1H), 7.84 (d, J = 8.2 Hz, 2H), 7.70 (dd, J = 10.4, 2.5 Hz, 1H), 7.34-7.25 (m, 2H), 4.43 (s, 2H), 3.85 (s, 3H), 3.83 (s, 3H), 2.27 (s, 3H). **^{13}C {^{1}H} NMR** ($CDCl_3$, 101 MHz): δ 161.0 (d, $^1J_{C-F}$ = 245.2 Hz), 150.8 (d, $^5J_{C-F}$ = 1.1 Hz), 150.2 (d, $^4J_{C-F}$ = 5.3 Hz), 139.0, 130.3, 128.4, 128.3, 126.4 (2C), 126.2 (d, $^4J_{C-F}$ = 2.4 Hz), 125.23, 125.17 (d, $^3J_{C-F}$ = 8.9 Hz), 122.7, 121.7 (2C), 116.1 (d, $^2J_{C-F}$ = 25.4 Hz), 106.3 (d, $^2J_{C-F}$ = 22.3 Hz), 62.3, 61.6, 32.8, 12.7. **^{19}F NMR** ($CDCl_3$, 377 MHz): δ -114.78 (ddd, J = 10.2, 8.5, 5.7 Hz). **HRMS (ESI+)** *m/z*: $[M+H]^+$ calculated for $C_{21}H_{19}FNO_2S$: 368.111504 found 368.110660.

*6-((7-fluoro-1,4-dimethoxy-3-methylnaphthalen-2-yl)methyl)benzo[d]thiazole (**a-39**):* According to the general procedure C, (1,3-benzothiazol-6-yl)boronic acid was used. **a-39** was isolated by purification by flash chromatography on silica gel (Cyclohexane/Ethyl acetate, 8/2, v/v, UV) as a colorless oil (85 mg, 62% yield). **^{1}H NMR** ($CDCl_3$, 400 MHz): δ 8.94 (s, 1H), 8.11 (dd, J = 9.2, 5.6 Hz, 1H), 8.04 (br d, J = 4.4 Hz, 1H), 7.71 (dd, J = 10.4, 2.5 Hz, 1H), 7.61 (s, 1H), 7.38 (br d, J = 2.8 Hz, 1H), 7.28 (td, J = 8.8, 2.5 Hz, 1H), 4.41 (s, 2H), 3.85 (s, 3H), 3.84 (s, 3H), 2.26 (s, 3H). **^{13}C {^{1}H} NMR** ($CDCl_3$, 101 MHz): δ 161.0 (d, $^1J_{C-F}$ = 245.4 Hz), 153.8, 152.2, 150.8 (d, $^4J_{C-F}$ = 1.2 Hz), 150.2 (d, $^3J_{C-F}$ = 5.3 Hz), 138.2, 134.7, 130.4, 128.3 (d, $^3J_{C-F}$ = 8.6 Hz), 127.1, 126.2 (d, $^4J_{C-F}$ = 2.5 Hz), 125.3, 125.2, 123.4, 120.7, 116.2 (d, $^2J_{C-F}$ = 25.4 Hz), 106.4 (d, $^2J_{C-F}$ = 22.3 Hz), 62.3, 61.7, 32.9, 12.7. **^{19}F NMR** ($CDCl_3$, 377 MHz): δ -114.66 (ddd, J = 10.2, 8.4, 5.7 Hz). **HRMS (ESI+)** *m/z*: $[M+H]^+$ calculated for $C_{21}H_{19}NO_2S$: 368.111504, found 368.111827.

*3-(benzo[d]thiazol-5-ylmethyl)-6-fluoro-2-methylnaphthalene-1,4-dione (**C-a-40**):* According to the general procedure D, from **a-38**, **C-a-40** was isolated by purification by flash chromatography on silica gel (Cyclohexane/Ethyl acetate, 7/3, v/v, UV) as a yellow solid (61 mg, 91% yield). **^{1}H NMR** ($CDCl_3$, 400 MHz): δ 9.04 (s, 1H), 8.13 (dd, J = 8.5, 5.2 Hz, 1H), 7.95 (s, 1H), 7.89 (d, J = 8.2 Hz, 1H), 7.72 (dd, J = 8.5, 2.5 Hz, 1H), 7.46-7.30 (m, 2H), 4.20 (s, 2H), 2.29 (s, 3H). **^{13}C {^{1}H} NMR** ($CDCl_3$, 101 MHz): δ 184.0, 183.6, 166.1 (d, $^1J_{C-F}$ = 256.9 Hz), 155.3, 154.2, 145.2 (d, $^5J_{C-F}$ = 1.8 Hz), 145.2, 136.5, 134.7 (d, $^3J_{C-F}$ = 7.9 Hz), 132.4, 129.8 (d, $^3J_{C-F}$ = 8.8 Hz), 128.8 (d, $^4J_{C-F}$ = 3.2 Hz), 126.8, 123.1, 122.2, 120.9 (d, $^2J_{C-F}$ = 22.6 Hz), 113.3 (d, $^2J_{C-F}$ = 23.5 Hz), 32.4, 13.6. **^{19}F NMR** ($CDCl_3$, 377 MHz): δ -102.4 (td, J = 8.3, 5.3 Hz). **HRMS (ESI+)** *m/z*: $[M+H]^+$ calculated for $C_{19}H_{13}FNO_2S$: 338.064554 found 338.063505. **M.p.** = 109-111 °C.

*3-(benzo[d]thiazol-6-ylmethyl)-6-fluoro-2-methylnaphthalene-1,4-dione (**C-a-41**):* According to the general procedure D, from **a-39**, **C-a-41** was isolated by purification by flash chromatography on silica

gel (Cyclohexane/Ethyl acetate, 8/2, v/v, UV) as a yellow solid (32 mg, 42% yield). **^{1}H NMR** ($CDCl_3$, 400 MHz): δ 8.96 (s, 1H), 8.13 (dd, J = 8.6, 5.2 Hz, 1H), 8.04 (d, J = 7.5 Hz, 1H), 7.80 (s, 1H), 7.73 (dd, J = 8.6, 2.6 Hz, 1H), 7.44-7.34 (m, 2H), 4.17 (s, 2H), 2.29 (s, 3H). **^{13}C {^{1}H} NMR** ($CDCl_3$, 101 MHz): δ 184.0, 183.7, 166.1 (d, $^1J_{C\text{-}F}$ = 257.0 Hz), 154.4, 152.2, 145.18, 145.17, 145.1, 135.8, 134.6 (d, $^3J_{C\text{-}F}$ = 7.8 Hz), 129.8 (d, $^3J_{C\text{-}F}$ = 8.8 Hz), 128.8 (d, $^4J_{C\text{-}F}$ = 3.1 Hz), 127.3, 123.8, 121.6, 120.9 (d, $^2J_{C\text{-}F}$ = 22.5 Hz), 113.4 (d, $^2J_{C\text{-}F}$ = 23.5 Hz), 32.6, 13.6. **^{19}F NMR** ($CDCl_3$, 377 MHz): δ -102.28 (td, J = 8.2, 5.3 Hz). **HRMS (ESI+)** *m/z*: $[M+H]^+$ calculated for $C_{19}H_{13}FNO_2S$: 338.064554, found 338.064817. **M.p.** = 170-172 °C.

*2-((7-fluoro-1,4-dimethoxy-3-methylnaphthalen-2-yl)methyl)furan (**a-42**):* According to the general procedure C, 2-furanylboronic acid was used. **a-42** was isolated by purification by flash chromatography on silica gel (Cyclohexane/Ethyl acetate, gradient from 100/0 to 99/1, v/v, UV) as a colorless oil (139 mg, 83% yield). **^{1}H NMR** ($CDCl_3$, 400 MHz): δ 8.09 (dd, J = 9.2, 5.6 Hz, 1H), 7.68 (dd, J = 10.4, 2.6 Hz, 1H), 7.32 (br d, J = 1.1 Hz, 1H), 7.26 (ddd, J = 9.1, 8.3, 2.6 Hz, 1H), 6.26 (dd, J = 3.1, 1.9 Hz, 1H), 5.87-5.82 (m, 1H), 4.23 (s, 2H), 3.87 (d, J = 3.4 Hz, 6H), 2.36 (s, 3H). **^{13}C {^{1}H} NMR** ($CDCl_3$, 101 MHz): δ 161.0 (d, $^1J_{C\text{-}F}$ = 245.2 Hz), 154.0, 150.5 (d, $^5J_{C\text{-}F}$ = 1.4 Hz), 150.2 (d, $^4J_{C\text{-}F}$ = 5.4 Hz), 141.3, 128.4, 128.3 (d, $^3J_{C\text{-}F}$ = 8.7 Hz), 126.3 (d, $^4J_{C\text{-}F}$ = 2.4 Hz), 125.3, 125.2 (d, $^3J_{C\text{-}F}$ = 8.9 Hz), 116.2 (d, $^2J_{C\text{-}F}$ = 25.4 Hz), 110.4, 106.4 (d, $^2J_{C\text{-}F}$ = 22.3 Hz), 106.0, 62.5, 61.6, 26.5, 12.4. **^{19}F NMR** ($CDCl_3$, 377 MHz): δ -114.95 (ddd, J = 10.4, 8.4, 5.7 Hz). **HRMS (ESI+)** *m/z*: $[M+H]^+$ calculated for $C_{18}H_{18}FO_3$: 301.123449, found 301.123811.

*6-fluoro-3-(furan-2-ylmethyl)-2-methylnaphthalene-1,4-dione (**C-a-43**):* According to the general procedure D, from **a-42**, **C-a-43** was isolated by purification by flash chromatography on silica gel (Cyclohexane/Ethyl acetate, gradient from 8/2 to 7/3, v/v, UV) as a sticky yellow dark solid (19 mg, 14% yield). **^{1}H NMR** ($CDCl_3$, 400 MHz): δ 8.12 (dd, J = 8.6, 5.3 Hz, 1H), 7.73 (dd, J = 8.6, 2.6 Hz, 1H), 7.35 (td, J = 8.3, 2.7 Hz, 1H), 7.27 (dd, J = 1.8, 0.7 Hz, 1H), 6.27 (dd, J = 3.2, 1.9 Hz, 1H), 6.08 (dd, J = 3.2, 0.8 Hz, 1H), 4.03 (s, 2H), 2.29 (s, 3H). **^{13}C {^{1}H} NMR** ($CDCl_3$, 101 MHz): δ 184.1, 183.0 (d, $^4J_{C\text{-}F}$ = 1.4 Hz), 166.1 (d, $^1J_{C\text{-}F}$ = 256.8 Hz), 151.0, 145.4, 142.6 (d, $^5J_{C\text{-}F}$ = 2.0 Hz), 141.7, 134.7 (d, $^3J_{C\text{-}F}$ = 7.9 Hz), 129.8 (d, $^3J_{C\text{-}F}$ = 8.9 Hz), 128.9 (d, $^4J_{C\text{-}F}$ = 3.3 Hz), 120.8 (d, $^2J_{C\text{-}F}$ = 22.6 Hz), 113.3 (d, $^2J_{C\text{-}F}$ = 23.5 Hz), 110.6, 106.9, 25.7, 13.2. **^{19}F NMR** ($CDCl_3$, 377 MHz): δ -102.49 (td, J = 8.3, 5.3 Hz). **HRMS (ESI+)** *m/z*: $[M+H]^+$ calculated for $C_{16}H_{12}FO_3$: 271.076499, found 271.078115.

*2-(3-fluorobenzyl)-3-methylnaphthalene-1,4-dione (**B-b-5**):* According to the general procedure B, 3-fluorobenzyl bromide was used. **B-b-5** was isolated by purification by flash chromatography on silica gel (Toluene/Cyclohexane, 8/2, v/v, UV) as a yellow solid (84 mg, 60% yield). **^{1}H NMR** ($CDCl_3$, 400 MHz): δ 8.08 (dd, J = 5.7, 3.3 Hz, 2H), 7.69 (dd, J = 5.8, 3.3 Hz, 2H), 7.22 (td, J = 7.9, 6.1 Hz, 1H), 7.04-6.99 (m, 1H), 6.95-6.84 (m, 2H), 4.02 (s, 2H), 2.24 (s, 3H). **^{13}C {^{1}H} NMR** ($CDCl_3$, 101 MHz): δ 185.3, 184.6, 163.0 (d, $^1J_{C\text{-}F}$ = 247.5 Hz), 144.8, 144.7, 140.6 (d, $^3J_{C\text{-}F}$ = 8.1 Hz), 133.7 (2C), 132.2, 132.0, 130.1 (d, $^3J_{C\text{-}F}$ = 9.1 Hz), 136.6, 126.4, 124.3 (d, $^4J_{C\text{-}F}$ = 3.0 Hz), 115.6 (d, $^2J_{C\text{-}F}$ = 21.2 Hz), 113.5 (d, $^2J_{C\text{-}F}$ = 21.2 Hz), 32.2 (d, $^4J_{C\text{-}F}$ = 2.0 Hz), 13.4. **^{19}F NMR** ($CDCl_3$, 377 MHz): δ -112.88 (ddd, J = 9.9, 8.7, 6.0 Hz). **HRMS (ESI+)** *m/z*: $[M+H]^+$ calculated for $C_{18}H_{14}FO_2$: 281.097234, found 281.097731. **M.p.** = 112-114 °C.

*2-(2-fluorobenzyl)-3-methylnaphthalene-1,4-dione (**A-b-6**):* According to the general procedure A, 2-fluorophenylacetic acid (2.0 equiv.) was used. **A-b-6** was isolated by purification by flash chromatography on silica gel (Toluene/Cyclohexane, 7/3, v/v, UV) as a yellow solid (109 mg, 67% yield). **^{1}H NMR** ($CDCl_3$, 400 MHz): δ 8.09 (ddd, J = 5.9, 3.2, 1.8 Hz, 2H), 7.70 (dd, J = 5.8, 3.3 Hz, 2H), 7.21-7.11 (m, 2H), 7.07-6.97 (m, 2H), 4.05 (s, 2H), 2.21 (s, 3H). **^{13}C {^{1}H} NMR** ($CDCl_3$, 101 MHz): δ 185.3, 184.6, 160.9 (d, $^1J_{C\text{-}F}$ = 245.4 Hz), 145.3, 144.4 (d, $^4J_{C\text{-}F}$ = 0.6 Hz), 133.7, 133.6, 132.3, 132.2, 130.5 (d, $^3J_{C\text{-}F}$ = 4.2 Hz), 128.3 (d, $^3J_{C\text{-}F}$ = 8.2 Hz), 126.6, 126.5, 125.0 (d, $^2J_{C\text{-}F}$ = 15.7 Hz), 124.3 (d, $^4J_{C\text{-}F}$ = 3.6 Hz), 115.5 (d, $^2J_{C\text{-}F}$ = 22.3 Hz), 25.7 (d, $^3J_{C\text{-}F}$ = 3.6 Hz), 13.1 (d, $^5J_{C\text{-}F}$ = 2.6 Hz). **^{19}F NMR** ($CDCl_3$, 377 MHz): δ -116.5 (m). **HRMS (ESI+)** *m/z*: $[M+H]^+$ calculated for $C_{18}H_{14}FO_2$: 281.097234, found 281.097166. **M.p.** = 135-137 °C.

*2-(3-bromo-4-(trifluoromethyl)benzyl)-3-methylnaphthalene-1,4-dione (**A-b-8**):* According to the general procedure A, 2-(3-bromo-4-(trifluoromethyl)phenyl)acetic acid (1.5 equiv.) was used. **A-b-8** was isolated by purification by flash chromatography on silica gel (Toluene/Cyclohexane, 7/3, v/v, UV) as an orange solid (167 mg, 70% yield). **^{1}H NMR** ($CDCl_3$, 400 MHz): δ 8.10 (ddd, J = 7.0, 4.9, 3.4 Hz, 2H), 7.76-7.69 (m, 2H), 7.58 (d, J = 7.8 Hz, 2H), 7.28 (s, 1H), 4.05 (s, 2H), 2.25 (s, 3H). **^{13}C {^{1}H} NMR** ($CDCl_3$, 101 MHz): δ 185.1, 184.5, 145.3, 144.0, 143.8, 135.0, 133.93, 133.88, 132.2, 132.0, 129.0, 128.6 (d, $^2J_{C\text{-}F}$ = 31.5 Hz), 128.1 (q, $^3J_{C\text{-}F}$ = 5.4 Hz), 127.5, 126.7, 126.6, 123.0 (d, $^1J_{C\text{-}F}$ = 273.1 Hz), 120.4 (m), 32.1, 13.6. **^{19}F NMR** ($CDCl_3$, 377 MHz): δ -62.42. **HRMS (ESI+)** *m/z*: $[M+Na]^+$ calculated for $C_{19}H_{13}BrF_3O_2$: 409.004553, found 409.003594. **M.p.** = 89-91 °C.

*2-(4-fluoro-3-(trifluoromethyl)benzyl)-3-methylnaphthalene-1,4-dione (**A-b-9**):* According to the general procedure A, 4-fluoro-3-(trifluoromethyl)phenylacetic acid (2.0 equiv.) was used. The reaction was realized with 1.74 mmol (300 mg) of menadione. **A-b-9** was isolated by purification by flash chromatography on silica gel (Toluene/Cyclohexane, 7/3, v/v, UV) as a yellow solid (556 mg, 92% yield). **^{1}H NMR** ($CDCl_3$, 400 MHz): δ 8.08 (td, J = 5.1, 3.3 Hz, 2H), 7.76-7.67 (m, 2H), 7.52-7.45 (m, 1H), 7.40 (ddd, J = 7.0, 4.4, 2.0 Hz, 1H), 7.14-7.05 (m, 1H), 4.03 (s, 2H), 2.26 (s, 3H). **^{13}C {^{1}H} NMR** ($CDCl_3$, 101 MHz): δ 185.2, 184.6, 158.6 (dq, J = 255.1, 1.9 Hz), 144.9, 144.3, 134.4 (d, $^4J_{C-F}$ = 4.0 Hz), 134.0 (d, $^3J_{C-F}$ = 8.3 Hz), 133.84, 133.81, 132.2, 132.0, 127.2 (qd, J = 4.6, 1.4 Hz), 126.7, 126.5, 122.6 (d, $^1J_{C-F}$ = 271.5 Hz), 118.5 (qd, J = 32.8, 12.6 Hz), 117.2 (d, $^2J_{C-F}$ = 20.7 Hz), 31.8, 13.5. **^{19}F NMR** ($CDCl_3$, 377 MHz): δ -61.36 (d, J = 12.6 Hz), -117.74 (ddq, J = 17.9, 11.3, 6.3 Hz). **HRMS (APCI+)** *m/z*: $[M+H]^+$ calculated for $C_{19}H_{13}F_4O_2$: 349.0846, found 349.0842. **M.p.** = 110-111 °C.

*2-((3-methyl-1,4-dioxo-1,4-dihydronaphthalen-2-yl)methyl)benzonitrile (**A-b-11**):* According to the general procedure A, 2-cyanophenylacetic acid (2.0 equiv.) was used. **A-b-11** was isolated by purification by flash chromatography on silica gel (Toluene/Cyclohexane, gradient from 9/1 to 10/0, v/v, UV) as a yellow solid (93 mg, 56% yield). **^{1}H NMR** ($CDCl_3$, 400 MHz): δ 8.10-8.03 (m, 2H), 7.72-7.68 (m, 2H), 7.64 (dd, J = 7.7, 1.2 Hz, 1H), 7.43 (td, J = 7.8, 1.4 Hz, 1H), 7.28 (td, J = 8.4, 8.0, 1.0 Hz, 1H), 7.11 (d, J = 7.9 Hz, 1H), 4.24 (s, 2H), 2.18 (s, 3H). **^{13}C {^{1}H} NMR** ($CDCl_3$, 101 MHz): δ 185.9, 184.3, 145.9, 143.5, 141.9, 133.8 (2C), 133.1 (2C), 132.1, 131.8, 128.5, 127.1, 126.6, 126.5, 118.0, 112.8, 30.9, 13.4. **HRMS (ESI+)** *m/z*: $[M+H]^+$ calculated for $C_{19}H_{14}NO_2$: 288.101905, found 288.101993. **M.p.** = 133-135 °C.

*2-(2,4-dichlorobenzyl)-3-methylnaphthalene-1,4-dione (**A-b-14**):* According to the general procedure A, 2,4-dichlorophenylacetic acid (1.0 equiv.) was used. **A-b-14** was isolated by purification by flash chromatography on silica gel (Toluene/Cyclohexane, 7/3, v/v, UV) as a yellow solid (113 mg, 59% yield). **^{1}H NMR** ($CDCl_3$, 400 MHz): δ 8.16-8.03 (m, 2H), 7.78-7.67 (m, 2H), 7.40 (d, J = 2.2 Hz, 1H), 7.09 (dd, J = 8.3, 2.2 Hz, 1H), 6.91 (d, J = 8.4 Hz, 1H), 4.07 (s, 2H), 2.14 (s, 3H). **^{13}C {^{1}H} NMR** ($CDCl_3$, 101 MHz): δ 184.9, 184.4, 146.0, 144.0, 134.7, 134.4, 133.7 (2C), 132.9, 132.2, 132.0, 129.9, 129.4, 127.3, 126.6, 126.5, 29.6, 13.4. **HRMS (APCI+)** *m/z*: $[M+H]^+$ calculated for $C_{18}H_{13}Cl_2O_2$: 331.0287, found 331.0274. **M.p.** = 120-122 °C.

*2-(4-chloro-2-(trifluoromethyl)benzyl)-3-methylnaphthalene-1,4-dione (**A-b-15**):* According to the general procedure A, 2-trifluoromethyl-4-chlorophenylacetic acid (2.0 equiv.) was used. **A-b-15** was isolated by purification by flash chromatography on silica gel (Toluene/Cyclohexane, 6/4, v/v, UV) as a yellow solid (113 mg, 53% yield). **^{1}H NMR** ($CDCl_3$, 400 MHz): δ 8.16-8.03 (m, 2H), 7.77-7.69 (m, 2H), 7.67 (d, J = 2.3 Hz, 1H), 7.32 (dd, J = 8.4, 2.3 Hz, 1H), 6.91 (d, J = 8.4 Hz, 1H), 4.17 (s, 2H), 2.10 (s, 3H). **^{13}C {^{1}H} NMR** ($CDCl_3$, 101 MHz): δ 184.9, 184.4, 146.5, 143.7, 135.3 (q, $^3J_{C-F}$ = 1.5 Hz), 133.9 (2C), 132.6, 132.3, 132.2, 132.0, 130.1 (q, $^2J_{C-F}$ = 30.8 Hz), 129.9, 126.8 (d, $^3J_{C-F}$ = 6.8 Hz), 126.7, 126.6, 123.8 (q, $^1J_{C-F}$ = 274.4 Hz), 28.4 (q, $^3J_{C-F}$ = 2.8 Hz), 13.2. **^{19}F NMR** ($CDCl_3$, 377 MHz): δ -61.28. **HRMS (APCI+)** *m/z*: $[M+H]^+$ calculated for $C_{19}H_{13}ClF_3O_4$: 365.0551, found 365.0550. **M.p.** = 141-143 °C.

*2-(3,4-dimethoxybenzyl)-3-methylnaphthalene-1,4-dione (**A-b-19**):* According to the general procedure A, 2,3-dimethoxyphenylacetic acid (1.5 equiv.) was used. **A-b-19** was isolated by purification by flash chromatography on silica gel (Toluene, UV) as a yellow solid (113 mg, 53% yield). **^{1}H NMR** ($CDCl_3$, 400 MHz): δ 8.02 (td, J = 5.2, 3.4 Hz, 2H), 7.67-7.60 (m, 2H), 6.79 (s, 1H), 6.71 (s, 2H), 3.92 (s, 2H), 3.81 (s, 3H), 3.78 (s, 3H), 2.22 (s, 3H). **^{13}C {^{1}H} NMR** ($CDCl_3$, 101 MHz): δ 185.3, 184.7, 149.0, 147.6, 145.3, 144.1, 133.42, 133.40, 132.03, 131.97, 130.5, 126.4, 126.2, 120.5, 112.2, 111.3, 55.8 (2C), 31.9, 13.2. **HRMS (ESI+)** *m/z*: $[M+Na]^+$ calculated for $C_{20}H_{18}NaO_4$: 345.109730, found 345.110406. **M.p.** = 101-102 °C.

*2-(benzo[d][1,3]dioxol-5-ylmethyl)-3-methylnaphthalene-1,4-dione (**A-b-21**):* According to the general procedure A, 3,4-methylenedioxyphenylacetic acid (2.0 equiv.) was used. **A-b-21** was isolated by purification by flash chromatography on silica gel (Toluene/Cyclohexane, 9/1, v/v, UV) as an orange solid (107 mg, 60% yield). **^{1}H NMR** ($CDCl_3$, 400 MHz): δ 8.09-8.01 (m, 2H), 7.70-7.63 (m, 2H), 6.69 (d, J = 10.7 Hz, 3H), 5.87 (s, 2H), 3.91 (s, 2H), 2.23 (s, 3H). **^{13}C {^{1}H} NMR** ($CDCl_3$, 101 MHz): δ 185.4, 184.7, 147.9, 146.1, 145.3, 144.3, 133.53, 133.51, 132.14, 132.06, 131.7, 126.5, 126.3, 121.6, 109.2, 108.4, 101.0, 32.1, 13.3. **HRMS (ESI+)** *m/z*: $[M+H]^+$ calculated for $C_{19}H_{15}O_4$: 307.096485, found 307.096938. **M.p.** = 80-81 °C.

*2-((2,3-dihydrobenzo[b][1,4]dioxin-6-yl)methyl)-3-methylnaphthalene-1,4-dione (**A-b-22**):* According to the general procedure A, 2-(2,3-dihydro-1,4-benzodioxin-6-yl)acetic acid (2.0 equiv.) was used. **A-b-22** was isolated by purification by flash chromatography on silica gel (Toluene/Cyclohexane, 9/1, v/v, UV) as a yellowish oil (169 mg, 91% yield). **^{1}H NMR** ($CDCl_3$, 400 MHz): δ 8.08 (dd, J = 5.5, 3.4 Hz, 2H), 7.69

(dd, *J* = 5.5, 3.3 Hz, 2H), 6.79-6.68 (m, 3H), 4.20 (s, 4H), 3.92 (s, 2H), 2.24 (s, 3H). **^{13}C {^{1}H} NMR** ($CDCl_3$, 101 MHz): *δ* 185.3, 184.5, 145.3, 144.1, 143.4, 142.1, 133.4 (2C), 132.0, 132.0, 131.1, 126.4, 126.2, 121.6, 117.3, 117.2, 64.3, 64.2, 31.6, 13.2. **HRMS (ESI+)** *m/z*: $[M+H]^+$ calculated for $C_{20}H_{17}O_4$: 321.112135 found 321.111572.

*2-((2,2-dimethylbenzo[d][1,3]dioxol-5-yl)methyl)-3-methylnaphthalene-1,4-dione (**A-b-23**):* According to the general procedure A, 2-(2,2-dimethylbenzo[*d*][1,3]dioxol-5-yl)acetic acid (2.0 equiv.) was used. **A-b-23** was isolated by purification by flash chromatography on silica gel (Toluene/Cyclohexane, gradient from 7/3 to 8/2, v/v, UV) as an orange solid (109 mg, 56% yield). **^{1}H NMR** ($CDCl_3$, 400 MHz): *δ* 8.09 (dd, *J* = 5.8, 3.3 Hz, 2H), 7.70 (dd, *J* = 5.8, 3.3 Hz, 2H), 6.71-6.55 (m, 3H), 3.93 (s, 2H), 2.25 (s, 3H), 1.63 (s, 6H). **^{13}C {^{1}H} NMR** ($CDCl_3$, 101 MHz): *δ* 185.6, 184.8, 147.8, 146.1, 145.6, 144.3, 133.58, 133.55, 132.24, 132.17, 131.0, 126.6, 126.4, 121.1, 118.0, 109.0, 108.2, 32.2, 26.0 (2C), 13.4. **HRMS (APCI+)** *m/z*: $[M+H]^+$ calculated for $C_{21}H_{19}O_4$: 335.1278, found 335.1273. **M.p.** = 105-107 °C.

*2-((2,2-difluorobenzo[d][1,3]dioxol-5-yl)methyl)-3-methylnaphthalene-1,4-dione (**A-b-24**):* According to the general procedure A, 2-(2,2-difluoro-2*H*-1,3-benzodioxol-5-yl)acetic acid (1.5 equiv.) was used. **A-b-24** was isolated by purification by flash chromatography on silica gel (Toluene/cyclohexane, 8/2, v/v, UV) as a yellow solid (146 mg, 73% yield). **^{1}H NMR** ($CDCl_3$, 400 MHz): *δ* 8.09 (dq, *J* = 7.9, 3.9 Hz, 2H), 7.71 (dd, *J* = 5.8, 3.3 Hz, 2H), 6.96 (d, *J* = 9.3 Hz, 3H), 4.01 (s, 2H), 2.26 (s, 3H). **^{13}C {^{1}H} NMR** ($CDCl_3$, 101 MHz): *δ* 185.2, 184.7, 144.70, 144.68, 143.3 (d, $^{1}J_{C\text{-}F}$ = 164.8 Hz), 134.2, 133.8, 133.7, 132.2, 132.0, 131.7, 129.2, 126.6, 126.5, 123.7, 110.1, 109.4, 32.3, 13.4. **^{19}F NMR** ($CDCl_3$, 377 MHz): *δ* -50.01. **HRMS (ESI+)** *m/z*: $[M+H]^+$ calculated for $C_{19}H_{13}F_2O_4$: 343.077642, found 343.077299. **M.p.** = 79-81 °C.

*2-(cyclohexylmethyl)-3-methylnaphthalene-1,4-dione (**A-b-29**):* According to the general procedure A, cyclohexylacetic acid (2.0 equiv.) was used. **A-b-29** was isolated by purification by flash chromatography on silica gel (Toluene/cyclohexane, 8/2, v/v, UV) as a yellow solid (86 mg, 55% yield). **^{1}H NMR** ($CDCl_3$, 400 MHz): *δ* 8.07-8.03 (m, 2H), 7.68-7.64 (m, 2H), 2.55 (d, *J* = 7.2 Hz, 2H), 2.21-2.15 (m, 3H), 1.68-1.60 (m, 5H), 1.55 (ddd, *J* = 11.1, 7.6, 3.6 Hz, 1H), 1.18-1.10 (m, 3H), 1.07-1.02 (m, 2H). **^{13}C {^{1}H} NMR** ($CDCl_3$, 101 MHz): *δ* 185.4, 185.1, 146.7, 144.1, 133.4, 133.3, 132.3 (2C), 126.4, 126.2, 38.5, 34.6, 33.7 (2C), 26.45 (2C), 26.41, 13.4. **HRMS (ESI+)** *m/z*: $[M+H]^+$ calculated for $C_{18}H_{21}O_2$: 269.153606, found 269.153768. **M.p.** = 66-68 °C.

*3-((3-methyl-1,4-dioxo-1,4-dihydronaphthalen-2-yl)methyl)benzonitrile (**B-b-30**):* According to the general procedure B, 3-cyanobenzyl bromide was used. **B-b-30** was isolated by purification by flash chromatography on silica gel (Toluene/Cyclohexane, gradient from 8/2 to 10/0, v/v, UV) as a yellow solid (95 mg, 66% yield). **^{1}H NMR** ($CDCl_3$, 400 MHz): *δ* 8.09-8.01 (m, 2H), 7.72-7.66 (m, 2H), 7.52-7.45 (m, 3H), 7.36 (t, *J* = 7.7 Hz, 1H), 4.03 (s, 2H), 2.23 (s, 3H). **^{13}C {^{1}H} NMR** ($CDCl_3$, 101 MHz): *δ* 185.0, 184.4, 145.0, 143.9, 139.7, 133.8, 133.7, 133.2, 132.10, 132.07, 131.8, 130.3, 129.5, 126.6, 126.5, 118.7, 112.8, 32.1, 13.4. **HRMS (ESI+)** *m/z*: $[M+H]^+$ calculated for $C_{19}H_{14}NO_2$: 288.101905, found 288.102643. **M.p.** = 124-125 °C.

*2-(4-fluoro-3-iodobenzyl)-3-methylnaphthalene-1,4-dione (**B-b-33**):* According to the general procedure B, 4-(bromomethyl)-1-fluoro-2-iodobenzene was used. This reaction was realized with 2.37 mmol (400 mg) of 6-fluoro-menadione. **B-b-33** was isolated by purification by flash chromatography on silica gel (Toluene/Cyclohexane, gradient from 6/4 to 7/3, v/v, UV) as a yellow solid (545 mg, 46% yield). **^{1}H NMR** ($CDCl_3$, 400 MHz): *δ* 8.13-8.06 (m, 2H), 7.72 (dd, *J* = 5.8, 3.3 Hz, 2H), 7.60 (dd, *J* = 5.9, 2.2 Hz, 1H), 7.17 (ddd, *J* = 8.3, 4.8, 2.2 Hz, 1H), 6.98-6.92 (m, 1H), 3.96 (s, 2H), 2.25 (s, 3H). **^{13}C {^{1}H} NMR** ($CDCl_3$, 101 MHz): *δ* 185.3, 184.6, 160.6 (d, $^{1}J_{C\text{-}F}$ = 244.6 Hz), 144.8, 144.6, 139.3, 139.2, 135.9 (d, $^{4}J_{C\text{-}F}$ = 3.6 Hz), 133.79, 133.78, 132.1 (d, $^{3}J_{C\text{-}F}$ = 17.9 Hz), 130.4, 130.3, 126.6 (d, $^{3}J_{C\text{-}F}$ = 15.2 Hz), 115.7 (d, $^{2}J_{C\text{-}F}$ = 23.9 Hz), 81.6 (d, $^{2}J_{C\text{-}F}$ = 25.7 Hz), 31.4, 13.5. **^{19}F NMR** ($CDCl_3$, 377 MHz): *δ* -97.10 (q, *J* = 5.8 Hz). **HRMS (APCI+)** *m/z*: $[M+H]^+$ calculated for $C_{18}H_{13}FINO_2$: 406.9939, found 406.9916. **M.p.** = 149-151 °C.

*2-(4-bromo-2-(trifluoromethyl)benzyl)-3-methylnaphthalene-1,4-dione (**B-b-34**):* According to the general procedure B, 4-bromo-1-(bromomethyl)-2-trifluoromethyl benzene was used. **B-b-34** was isolated by purification by flash chromatography on silica gel (Toluene/Cyclohexane, 1/1, v/v, UV) as a yellow solid (141 mg, 69% yield). **^{1}H NMR** ($CDCl_3$, 400 MHz): *δ* 8.14-8.04 (m, 2H), 7.81 (d, *J* = 1.8 Hz, 1H), 7.76-7.69 (m, 2H), 7.47 (dd, *J* = 8.3, 1.6 Hz, 1H), 6.84 (d, *J* = 8.3 Hz, 1H), 4.15 (s, 2H), 2.09 (s, 3H). **^{13}C {^{1}H} NMR** ($CDCl_3$, 101 MHz): *δ* 184.8, 184.3, 146.5, 143.6, 135.9 (d, $^{3}J_{C\text{-}F}$ = 1.8 Hz), 135.2, 133.9, 133.8, 132.2, 131.9, 130.3 (q, $^{2}J_{C\text{-}F}$ = 30.8 Hz), 130.1, 129.6 (q, $^{3}J_{C\text{-}F}$ = 6.1 Hz), 126.7, 126.6, 123.6 (q, $^{1}J_{C\text{-}F}$ = 274.5 Hz), 120.2,

28.5 (q, $^4J_{C\text{-}F}$ = 2.6 Hz), 13.2. **^{19}F NMR** (CDCl$_3$, 377 MHz): δ -61.21. **HRMS (APCI+)** *m/z*: [M+H]$^+$ calculated for $C_{19}H_{13}BrF_3O_2$: 409.0046, found 409.0030. **M.p.** = 133-135 °C.

*4,4,5,5-tetramethyl-2-[4-(pentafluoro-λ^6-sulfanyl)phenyl]-1,3,2-dioxaborolane (**44**):* A flask was loaded with 1-iodo-4-(pentafluoro-λ^6-sulfanyl)benzene (1 equiv., 330 mg, 1 mmol) and bis(pinacolato)diboron (1.3 equiv., 330.072 mg, 1.3 mmol). Afterwards, 1,4 dioxane (5.72 mL) was transferred to the flask. To the prepared mixture, KOAc (3 equiv., 294.38 mg, 3 mmol) and (1,1'-Bis(diphenylphosphino)ferrocene)palladium(II) dichloride (0.05 equiv., 36.58 mg, 0.05 mmol) were quickly added and the mixture was heated to 80 °C and stirred vigorously for 18 h. The mixture was filtered on celite then the filtrate was concentrated. The crude residue was purified by flash chromatography on silica gel (Toluene) to afford 4,4,5,5-tetramethyl-2-[4-(pentafluoro-λ^6-sulfanyl)phenyl]-1,3,2-dioxaborolane (118 mg, 0.36 mmol, 35.75%) as a white-yellowish solid. According to this procedure, the desired reactant **44** was isolated by purification by flash chromatography on silica gel (Toluene) as a white-yellowish solid (36%). This product is used with no other purification. **^{1}H NMR** (CDCl$_3$, 400 MHz): δ 7.89 (d, *J* = 8.5 Hz, 2H), 7.74 (d, *J* = 8.2 Hz, 2H), 1.36 (s, 12H). **^{11}B NMR** (128 MHz, CDCl$_3$): δ 30.51. **^{19}F NMR** (377 MHz, CDCl$_3$) δ -62.20 (d, *J* = 138.3 Hz). **^{13}C {^{1}H} NMR** (CDCl$_3$, 101 MHz): δ 156.2 (p, $^2J_{C\text{-}F}$ = 16.7 Hz), 135.3 (2C), 125.2 (p, $^3J_{C\text{-}F}$ = 4.6 Hz, 2C), 84.6 (2C), 25.0 (4C) (the boron-bound carbon was not observed due to quadrupolar relaxation).

*1,4-dimethoxy-2-methyl-3-{[4-(pentafluoro-λ^6-sulfanyl)phenyl]methyl}naphthalene (**b-45**)*: In a flame dried sealable tube, 2-(chloromethyl)-1,4-dimethoxy-3-methylnaphthalene (1 equiv., 89.62 mg, 0.36 mmol), 4,4,5,5-tetramethyl-2-[4-(pentafluoro-λ^6-sulfanyl)phenyl]-1,3,2-dioxaborolane (1 equiv., 118 mg, 0.36 mmol), Na_2CO_3 (2.1 equiv., 79.56 mg, 0.75 mmol), DME (1.43 mL) and water (0.71 mL) were introduced successively under argon. The solvent was degassed and tetrakis(triphenylphosphine)palladium (0.02 equiv., 8.26 mg, 0.0071 mmol) was added. The tube was sealed and heated at 100 °C for 1 h. Water was added and the mixture was extracted 3 times with DCM, washed with brine, and dried over $MgSO_4$. The crude residue was purified by flash chromatography on silica gel (Cyclohexane/Toluene 6/4) to afford 1,4-dimethoxy-2-methyl-3-{[4-(pentafluoro-λ^6-sulfanyl)phenyl]methyl}naphthalene (62 mg, 0.15 mmol, 41%) as a colorless oil. **^{1}H NMR** (400 MHz, CDCl$_3$): δ ^{1}H NMR (400 MHz, CDCl$_3$) δ 8.18 – 8.03 (m, 2H), 7.63 (d, J = 8.4 Hz, 2H), 7.59 – 7.47 (m, 2H), 7.22 (d, J = 8.8 Hz, 2H), 4.32 (s, 3H), 3.89 (s, 3H), 3.86 (s, 3H), 2.27 (s, 3H).

^{19}F NMR (377 MHz, CDCl$_3$): δ -61.21. **^{13}C {^{1}H} NMR** (101 MHz, CDCl$_3$): δ 152.0 (p, $^2J_{C\text{-}F}$ = 17.1 Hz), 150.8, 150.7, 144.8, 128.4 (3C), 127.9, 127.3, 126.7, 126.2, 126.1 (p, $^3J_{C\text{-}F}$ = 4.5 Hz, 2C), 125.8, 122.6, 122.5, 62.4, 61.6, 32.5, 12.8. **HRMS (ESI+)** *m/z*: [M+H]$^+$ calculated for $C_{20}H_{20}F_5O_2S$: 419.1099, found 419.1074.

*2-methyl-3-{[4-(pentafluoro-λ^6-sulfanyl)phenyl]methyl}-1,4-dihydronaphthalene-1,4-dione (**C-b-46**)*: 1,4-dimethoxy-2-methyl-3-{[4-(pentafluoro-λ^6-sulfanyl)phenyl] methyl} naphtha-lene (1 equiv., 62 mg, 0.15 mmol) was dissolved in acetonitrile (1.79 mL) then a solution of CAN (2.2 equiv., 178.71 mg, 0.33 mmol) in H_2O (0.6 mL) was added dropwise. The mixture was stirred at room temperature for 30 mins. The mixture was extracted with dichloromethane. The organic layer was washed with brine, dried over $MgSO_4$, and concentrated under reduced pressure. The crude residue was purified by flash chromatography on silica gel (Cyclohexane/Toluene, 6/4) to afford 2-methyl-3-{[4-(pentafluoro-λ^6-sulfanyl)phenyl]methyl}-1,4-dihydronaphthalene-1,4-dione (50 mg, 0.13 mmol, 87%) as a yellow solid. **^{1}H NMR** (400 MHz, CDCl$_3$): δ 8.13 – 8.03 (m, 2H), 7.76 – 7.67 (m, 2H), 7.64 (d, *J* = 9.0 Hz, 2H), 7.32 (d, *J* = 8.7 Hz, 2H), 4.07 (s, 2H), 2.25 (s, 3H). **^{19}F NMR (377 MHz, CDCl$_3$)** δ -61.44. **^{13}C {^{1}H} NMR** (101 MHz, CDCl$_3$): δ 185.1, 184.5, 152.4 (p, $^2J_{C\text{-}F}$ = 16.7 Hz), 145.1, 144.2, 142.3, 133.9, 133.8, 132.2, 132.0, 128.9 (2C), 126.7, 126.6, 126.4 (p, $^3J_{C\text{-}F}$ = 4.8 Hz, 2C), 32.2, 13.5.**HRMS (ESI+)** *m/z*: [M+H]$^+$ calculated for $C_{18}H_{14}F_5O_2S$: 389.0629, found 389.0609. **M.p.** = 141-142°C.

S2. NMR spectra of key/new compounds

A-a-1 - ¹H NMR ($CDCl_3$, 400 MHz)

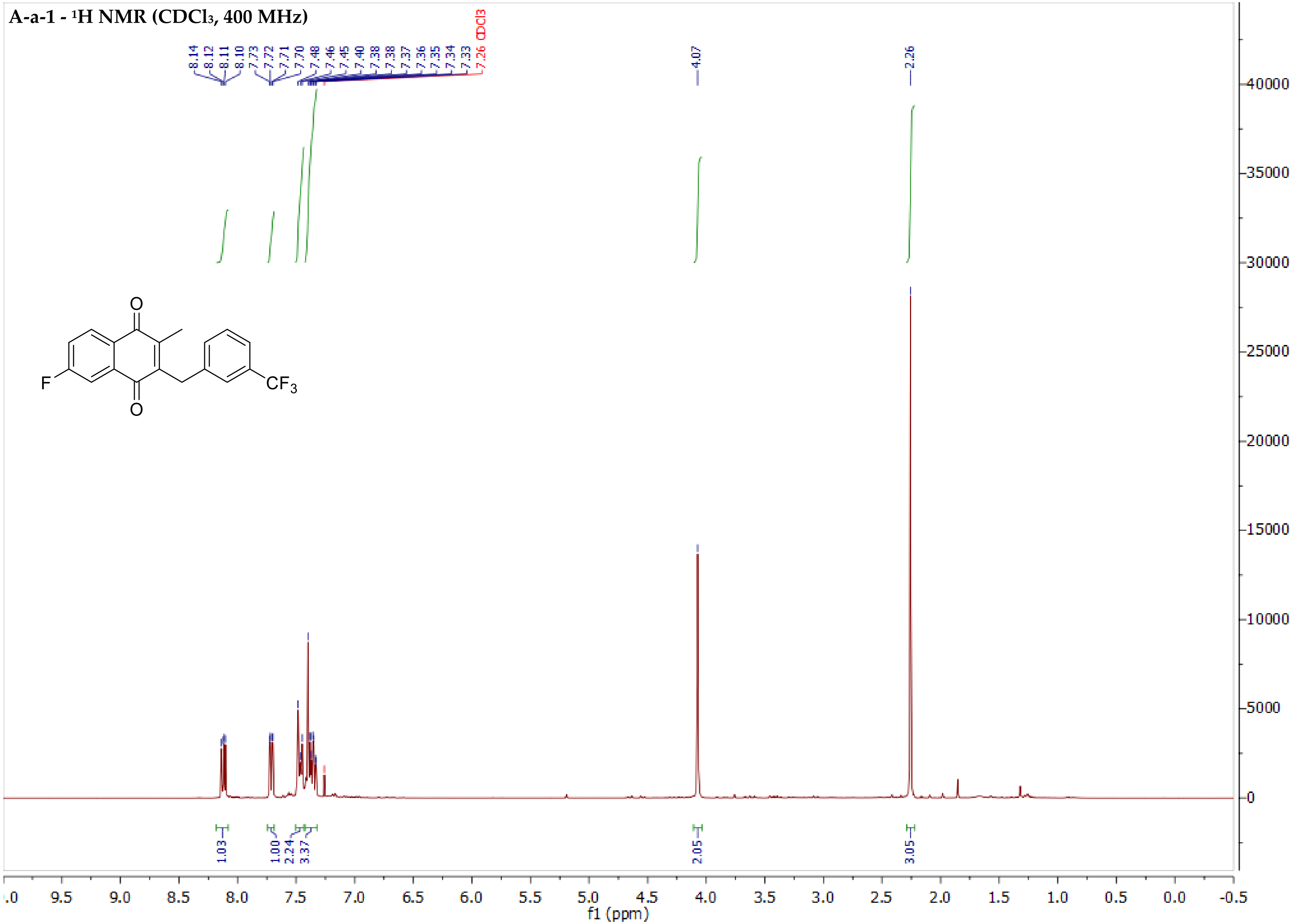

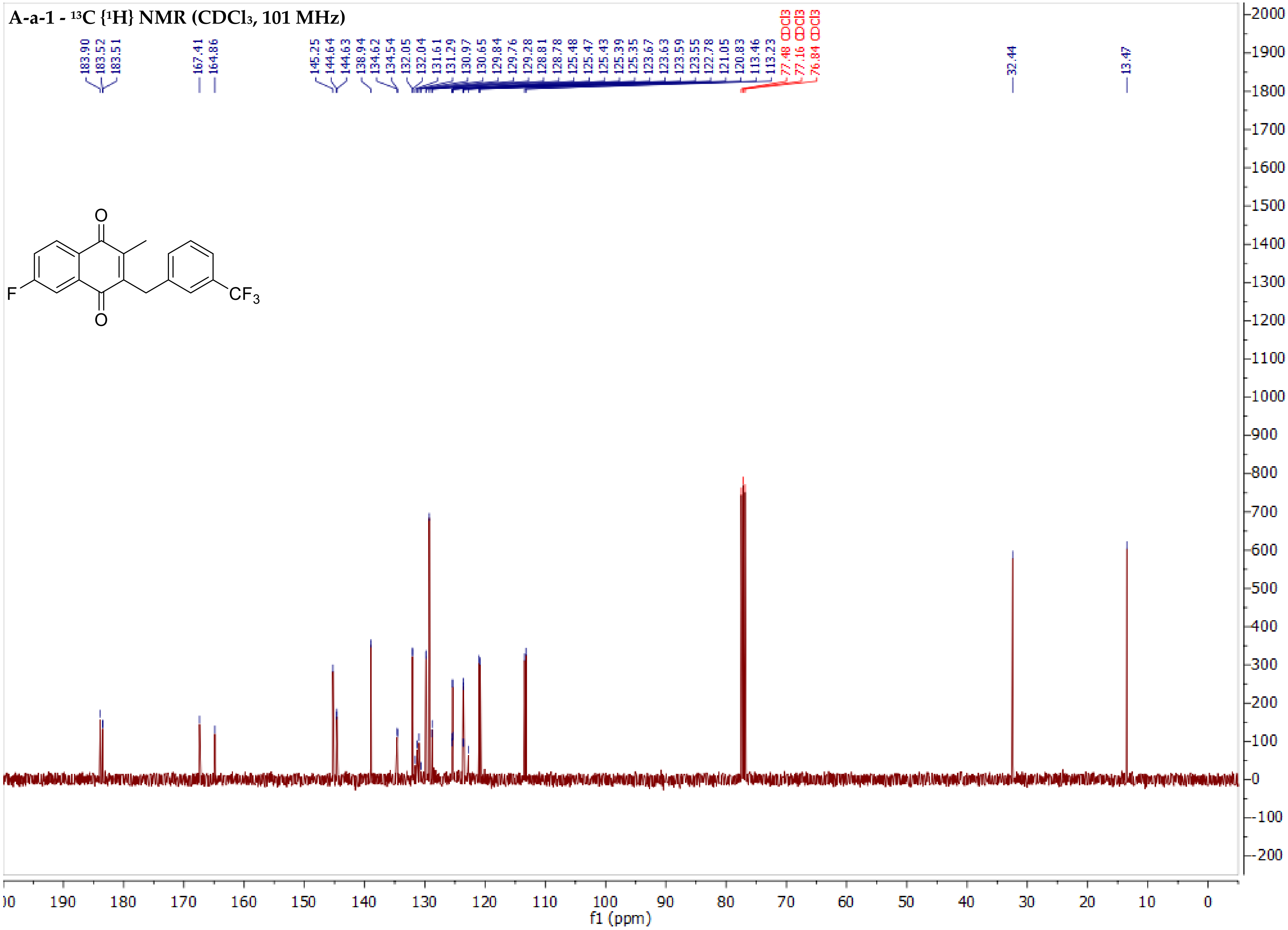
A-a-1 - ¹³C {¹H} NMR (CDCl₃, 101 MHz)
183.90
183.52
183.51
167.41
164.86
145.25
144.64
144.63
138.94
134.62
134.54
132.05
132.04
131.61
131.29
130.97
130.65
129.84
129.76
129.28
128.81
128.78
125.48
125.47
125.43
125.39
125.35
123.67
123.63
123.59
123.55
122.78
121.05
120.83
113.46
113.23
77.48 CDCl3
77.16 CDCl3
76.84 CDCl3
32.44
13.47
f1 (ppm)
F
O
O
CF3

A-a-1 - ^{19}F NMR ($CDCl_3$, 377 MHz)

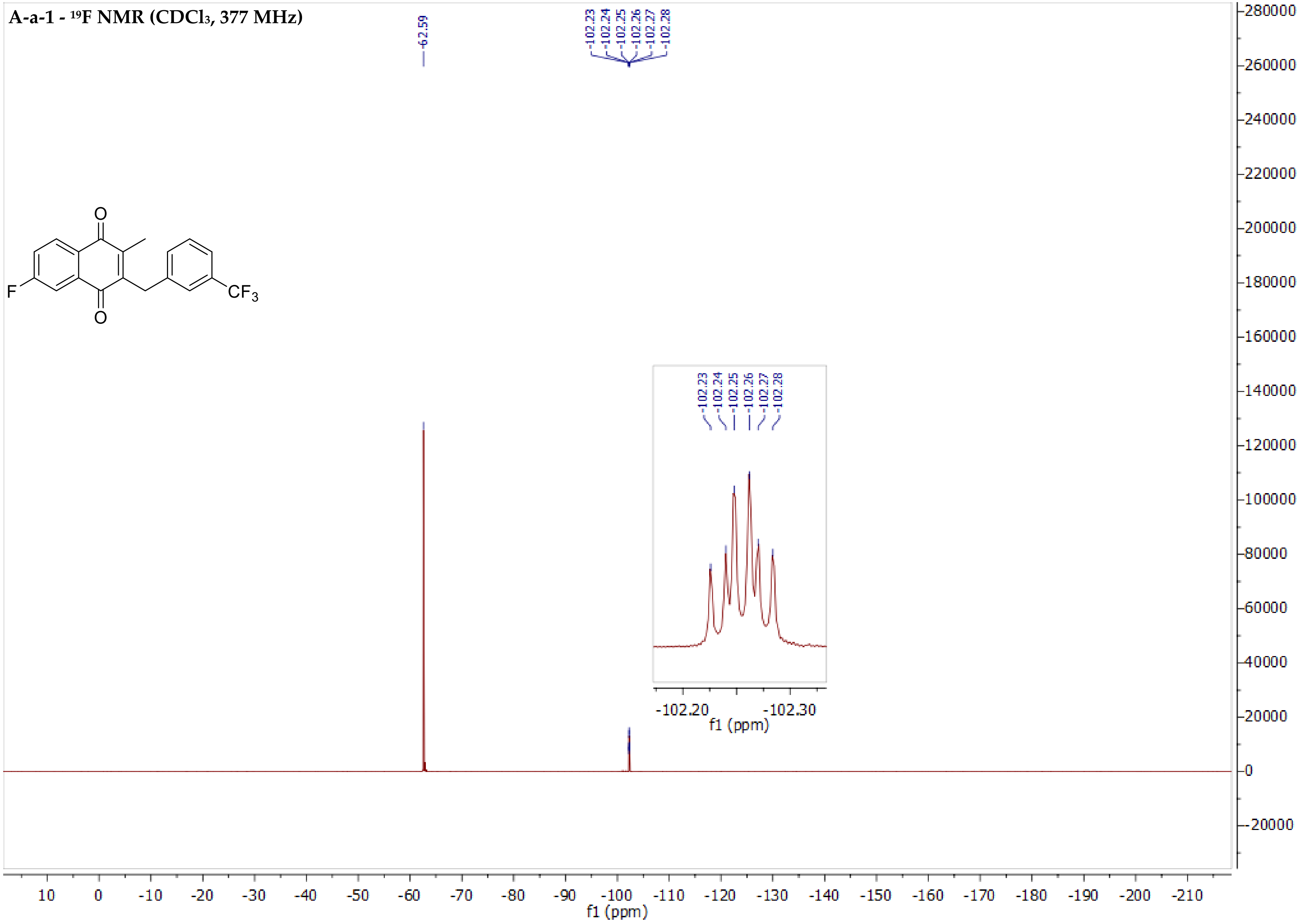

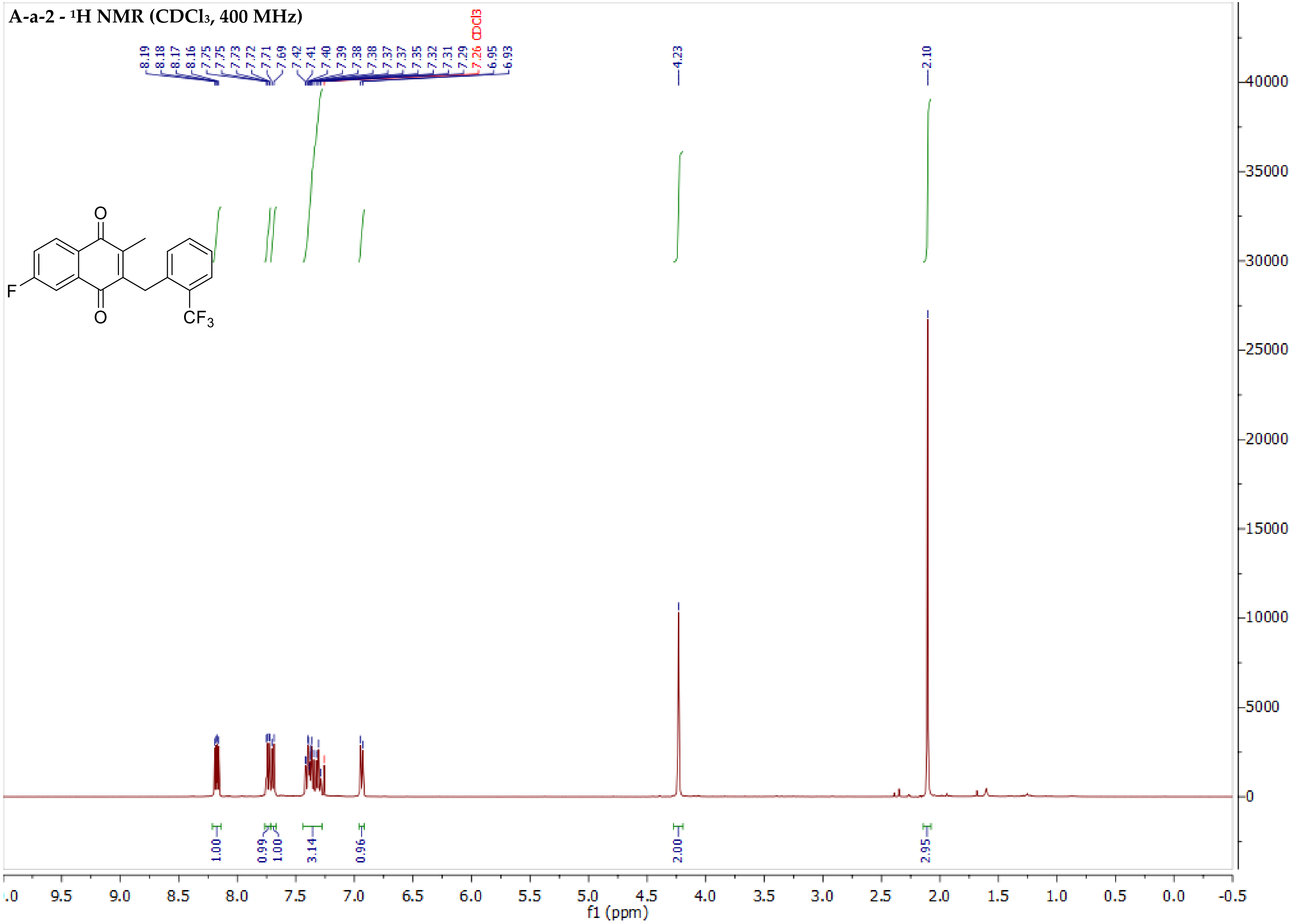

A-a-2 - ¹H NMR (CDCl₃, 400 MHz)
8.19
8.18
8.17
8.16
7.75
7.75
7.73
7.72
7.71
7.69
7.42
7.41
7.40
7.39
7.38
7.38
7.37
7.37
7.35
7.32
7.31
7.29
7.26 CDCl3
6.95
6.93
4.23
2.10
O
O
F
CF3
1.00
0.99
1.00
3.14
0.96
2.00
2.95
f1 (ppm)

A-a-2 - $^{13}C\{^1H\}$ NMR ($CDCl_3$, 101 MHz)

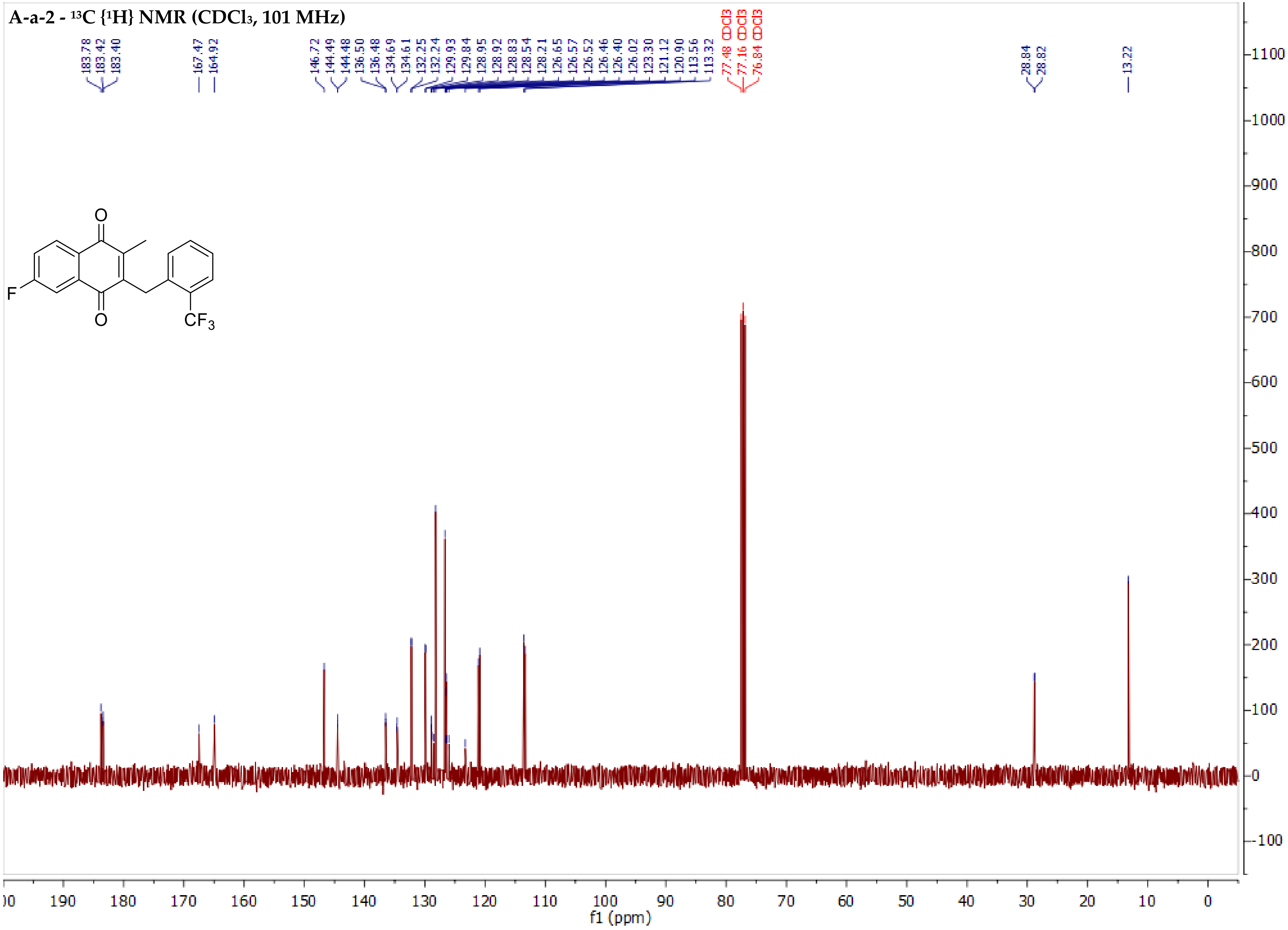

A-a-2 - ^{19}F NMR ($CDCl_3$, 377 MHz)

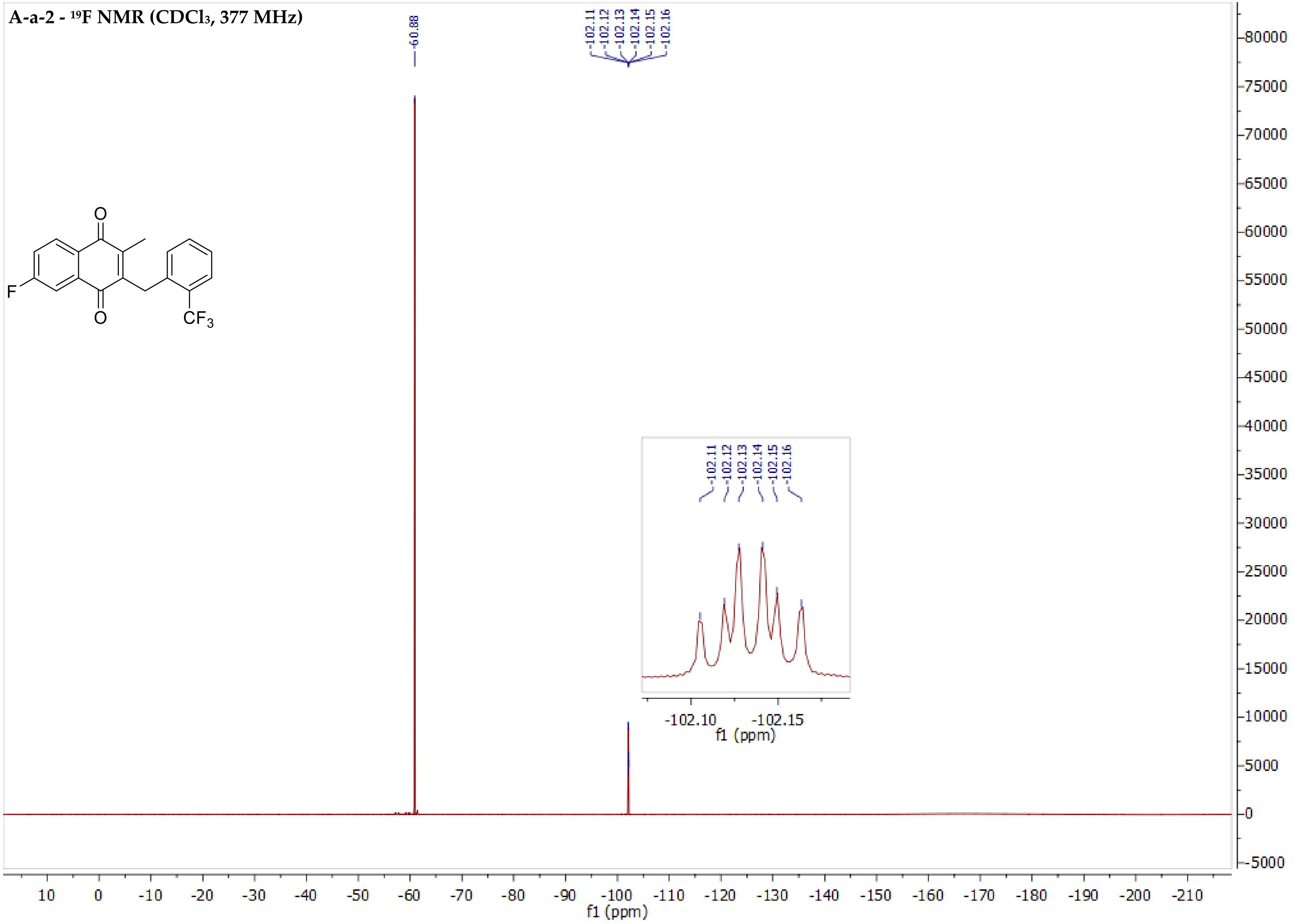

A-a-3 - ^1H NMR ($CDCl_3$, 400 MHz)

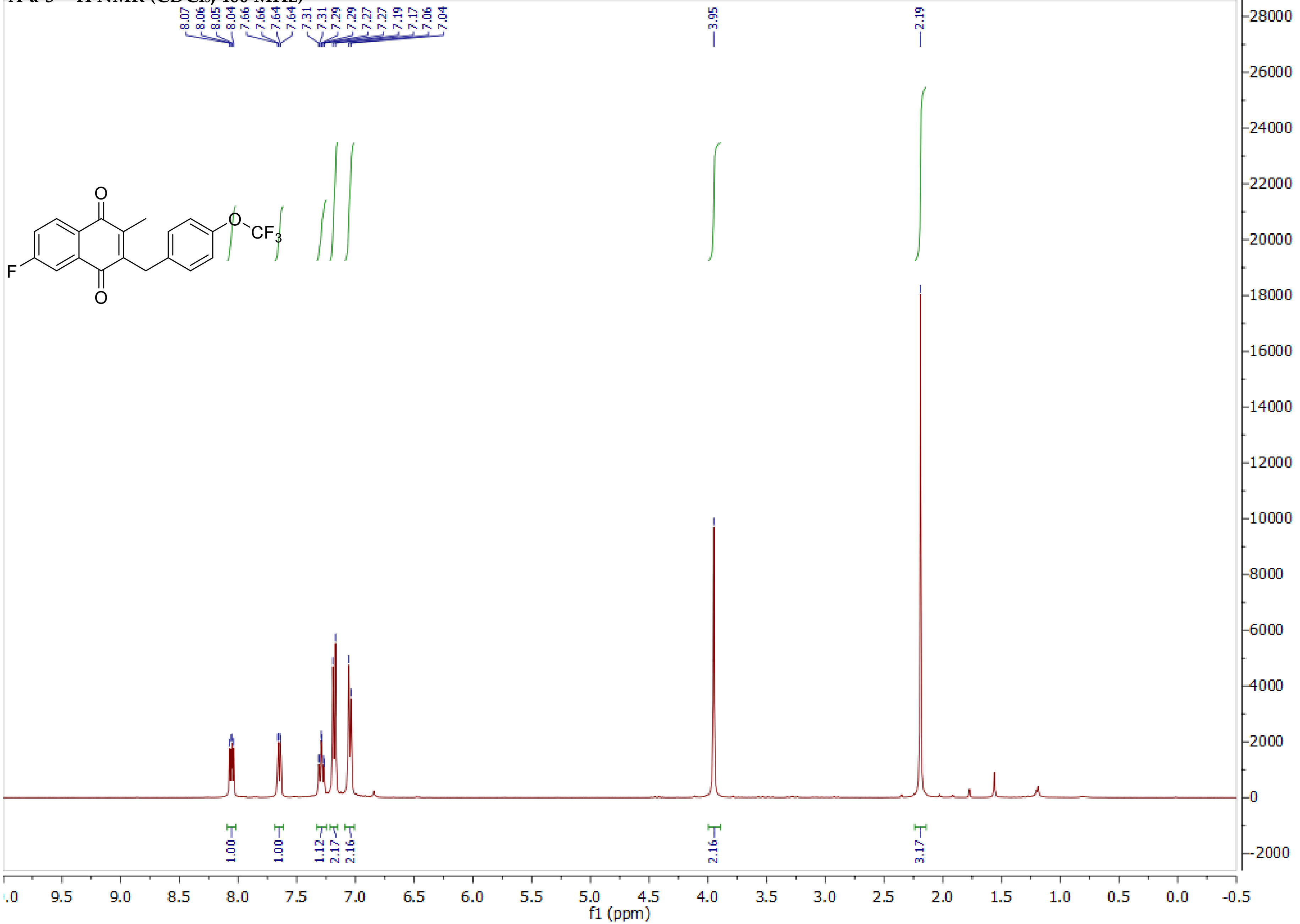

A-a-3 - $^{13}C\ \{^1H\}$ NMR ($CDCl_3$, 101 MHz)

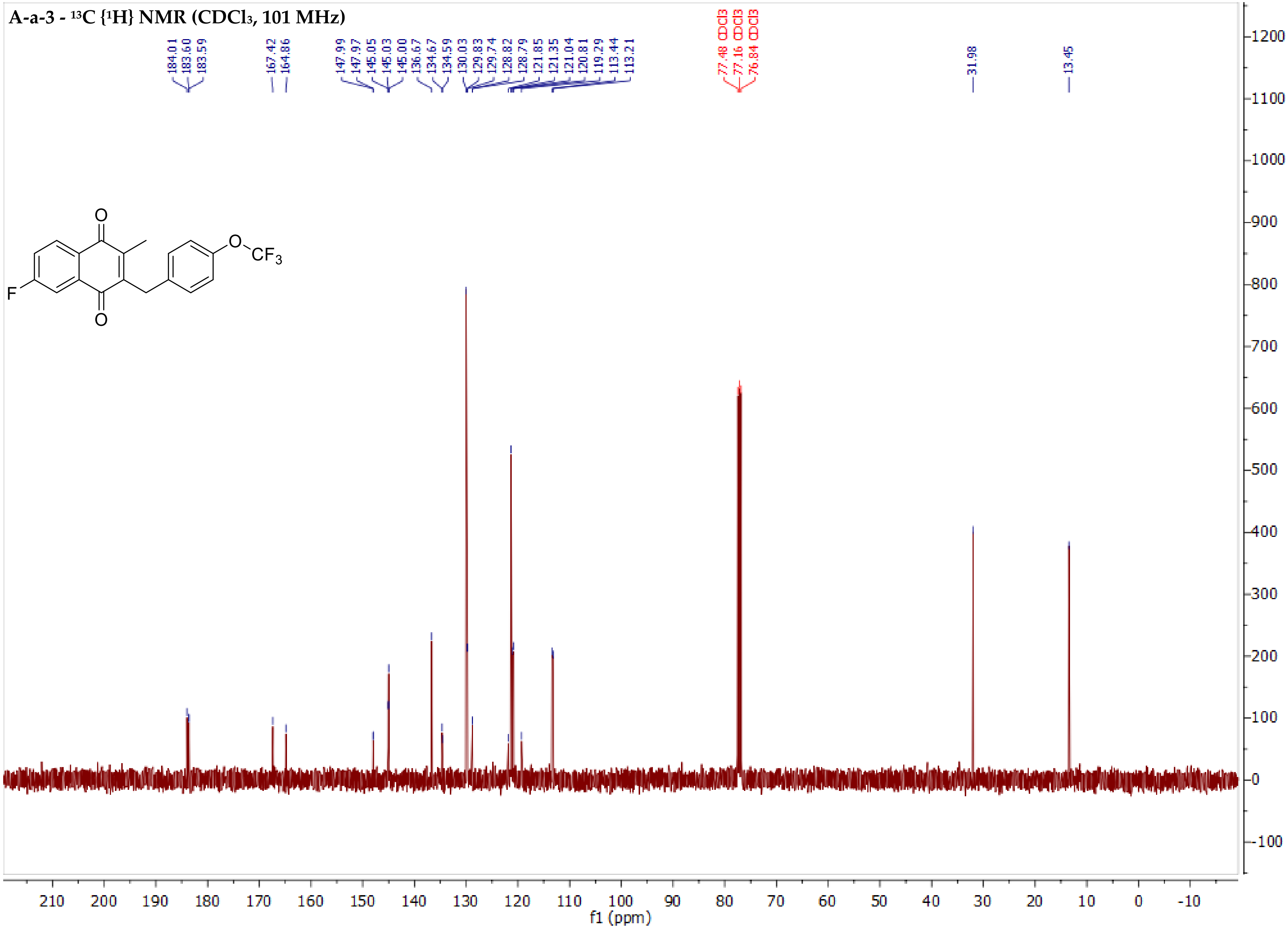

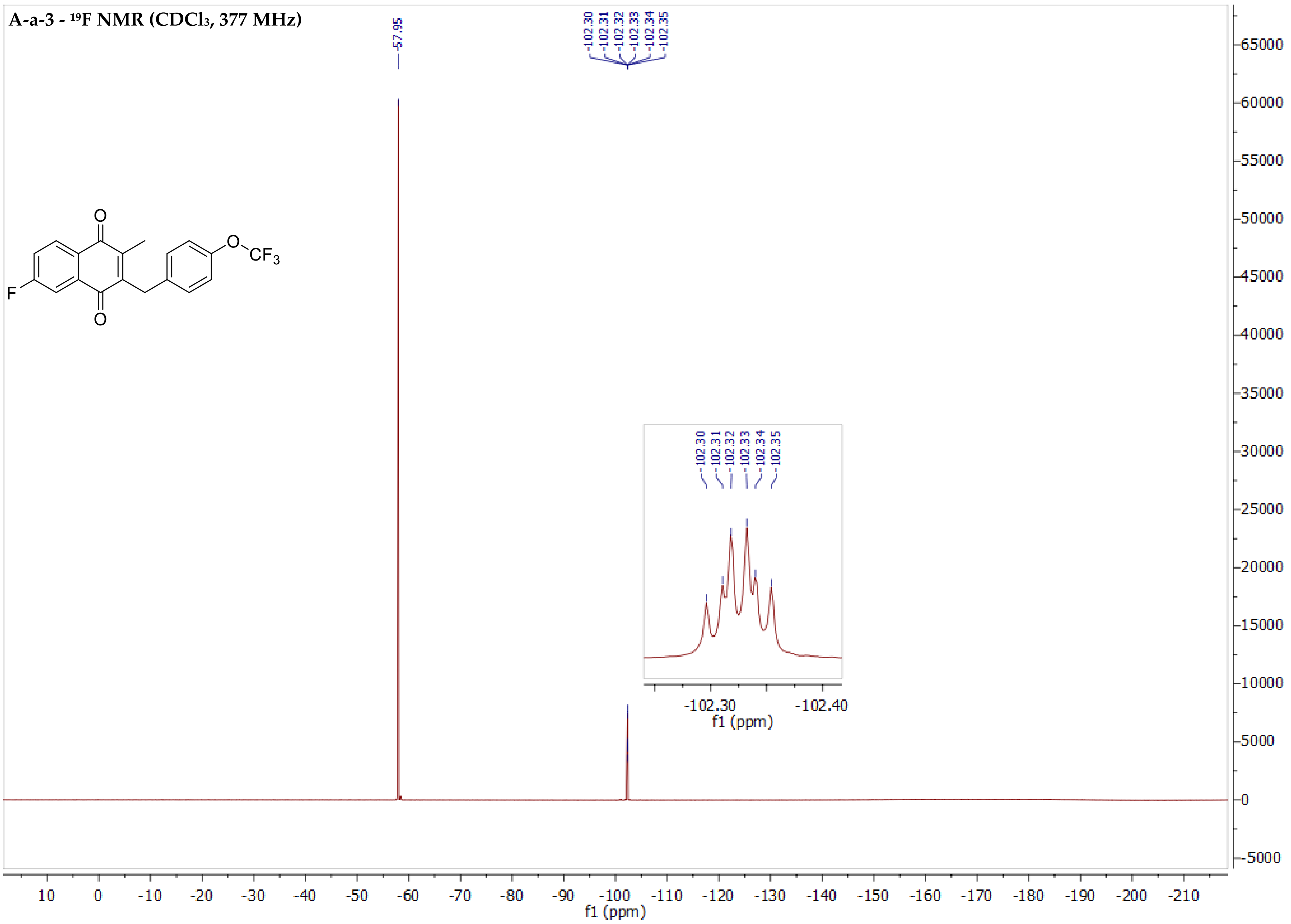
A-a-3 - ¹⁹F NMR (CDCl₃, 377 MHz)
-57.95
-102.30
-102.31
-102.32
-102.33
-102.34
-102.35
f1 (ppm)

A-a-4 - ¹H NMR (CDCl₃, 400 MHz)

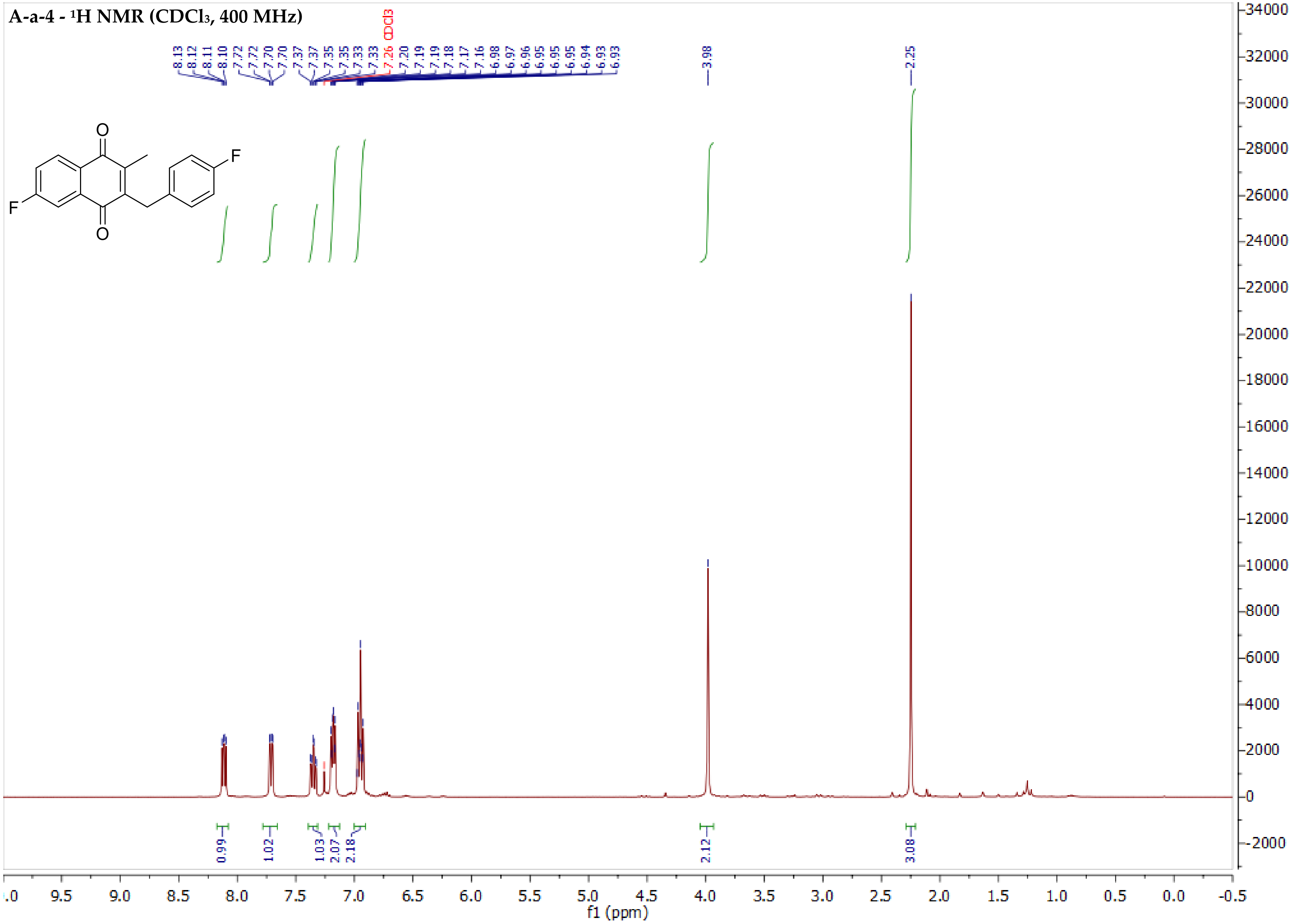

A-a-4 - $^{13}C\{^{1}H\}$ NMR ($CDCl_3$, 101 MHz)

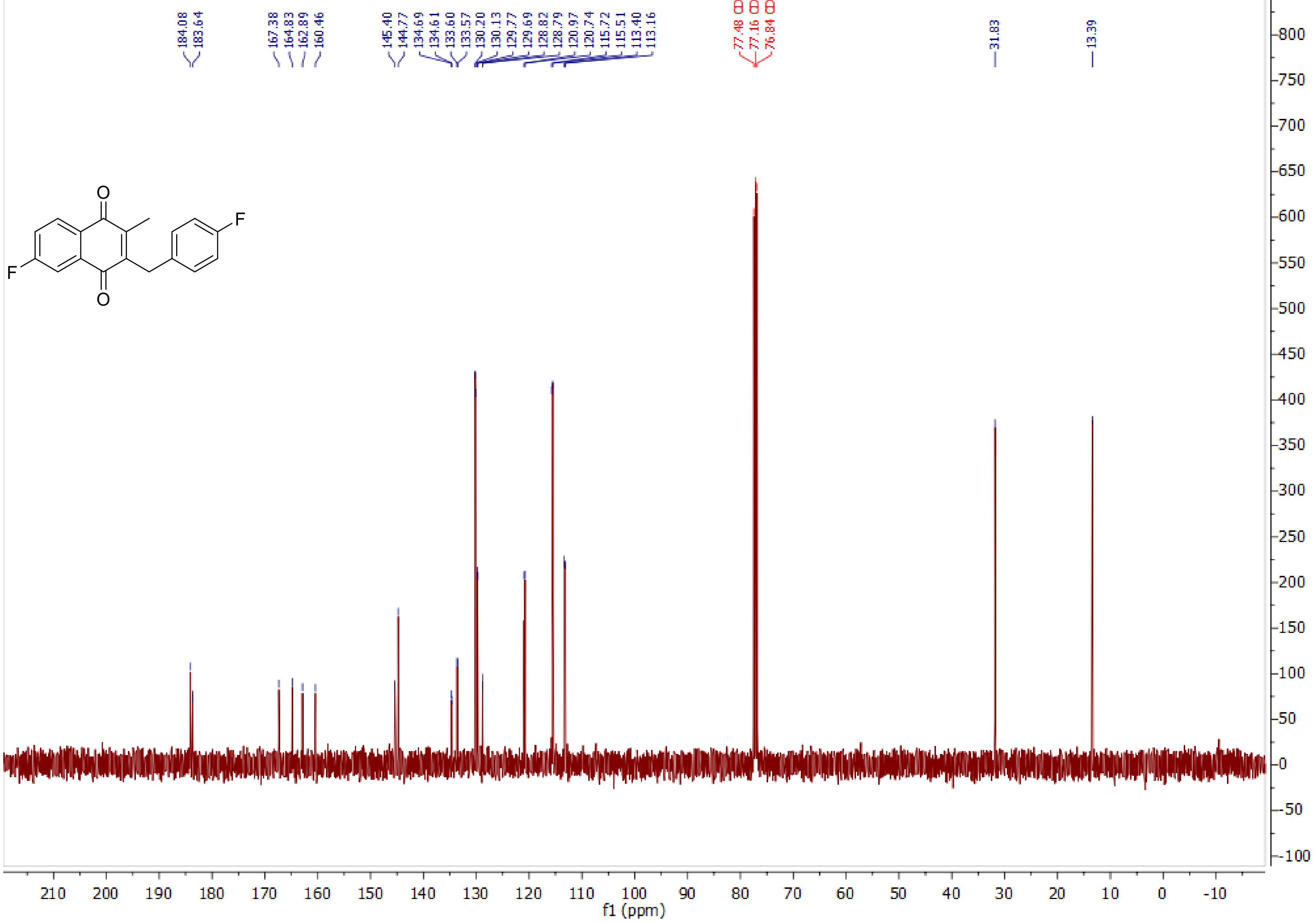

A-a-4 - ^{19}F NMR ($CDCl_3$, 377 MHz)

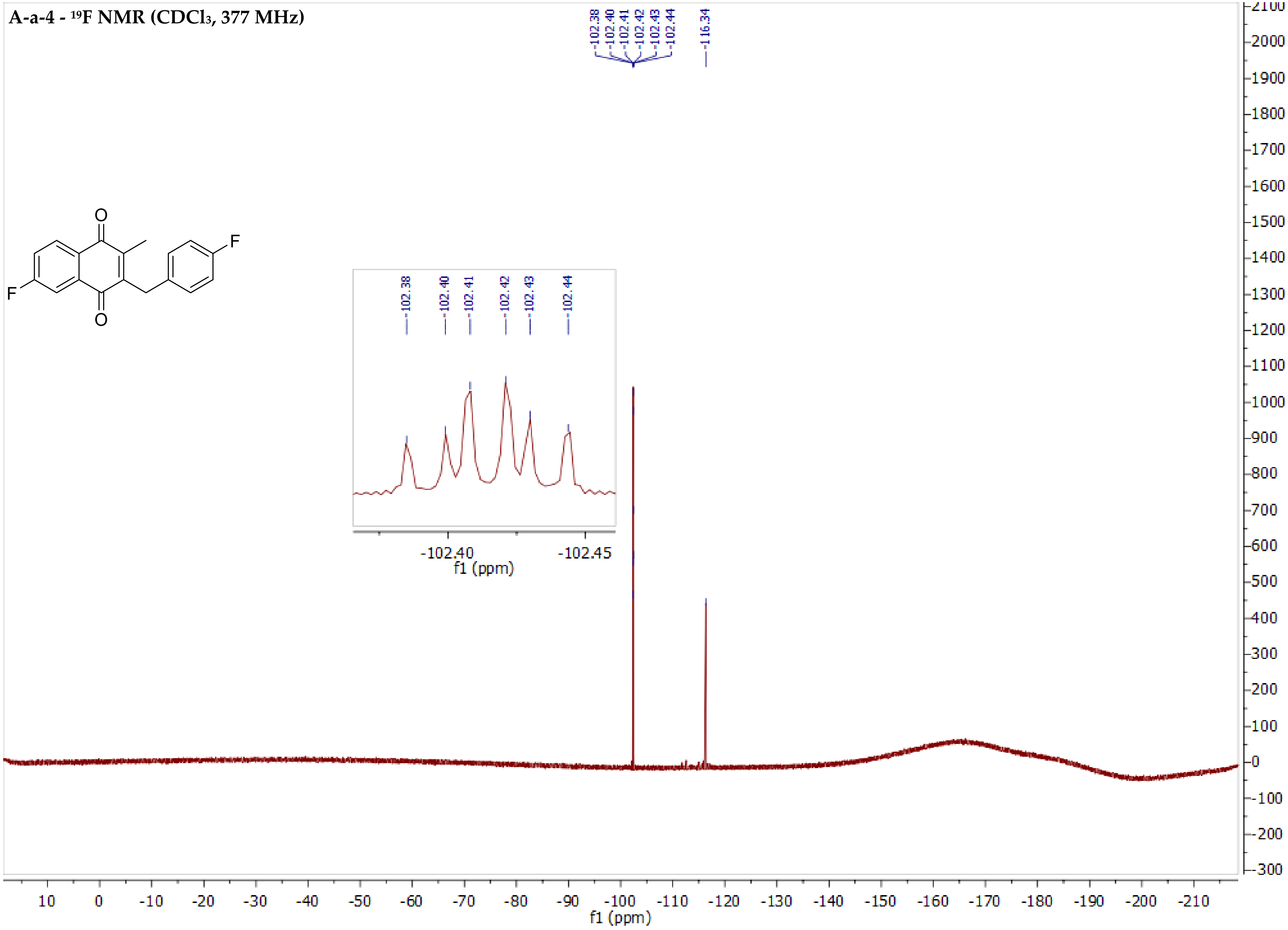

A-a-5 - ¹H NMR (CDCl₃, 400 MHz)

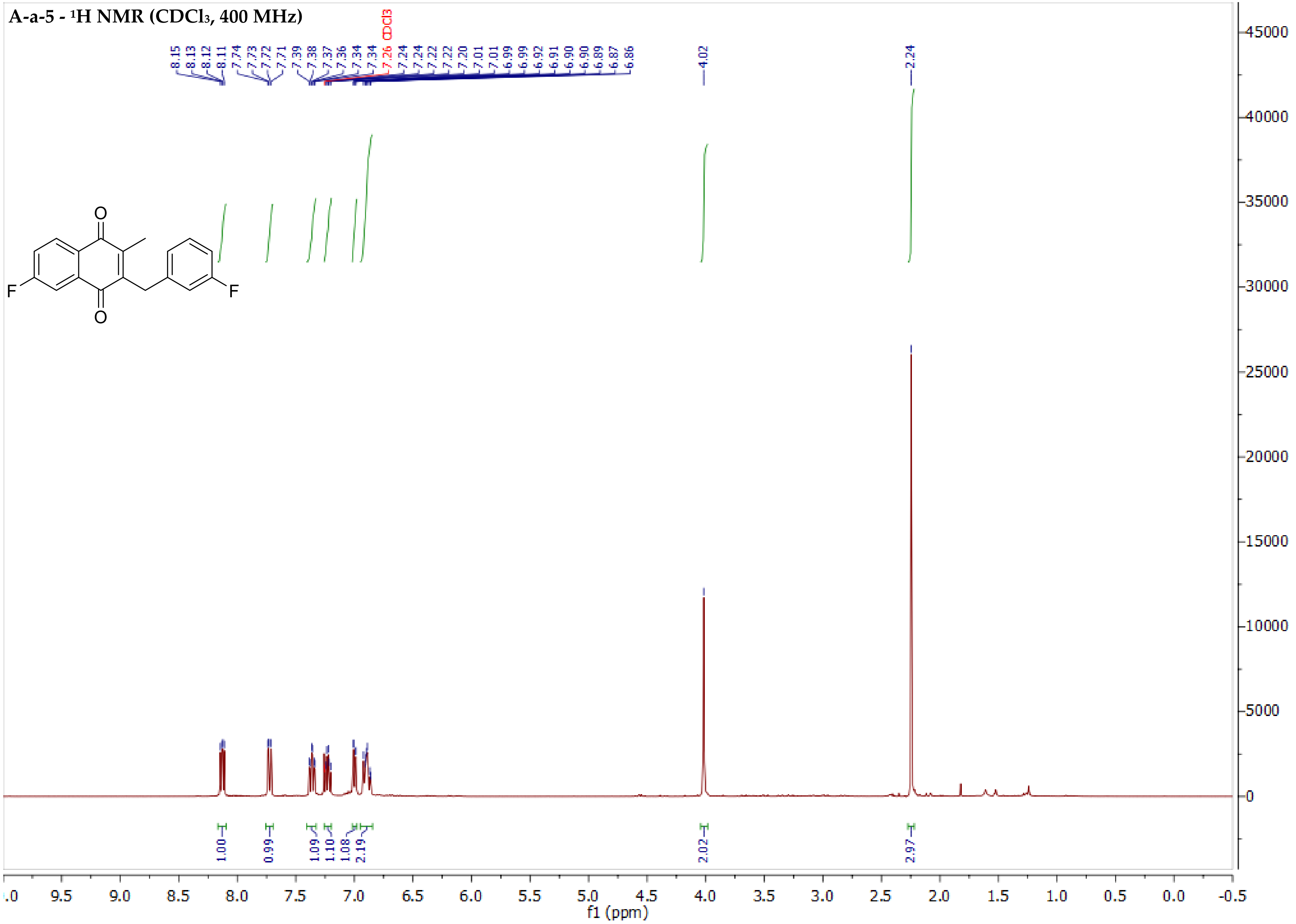

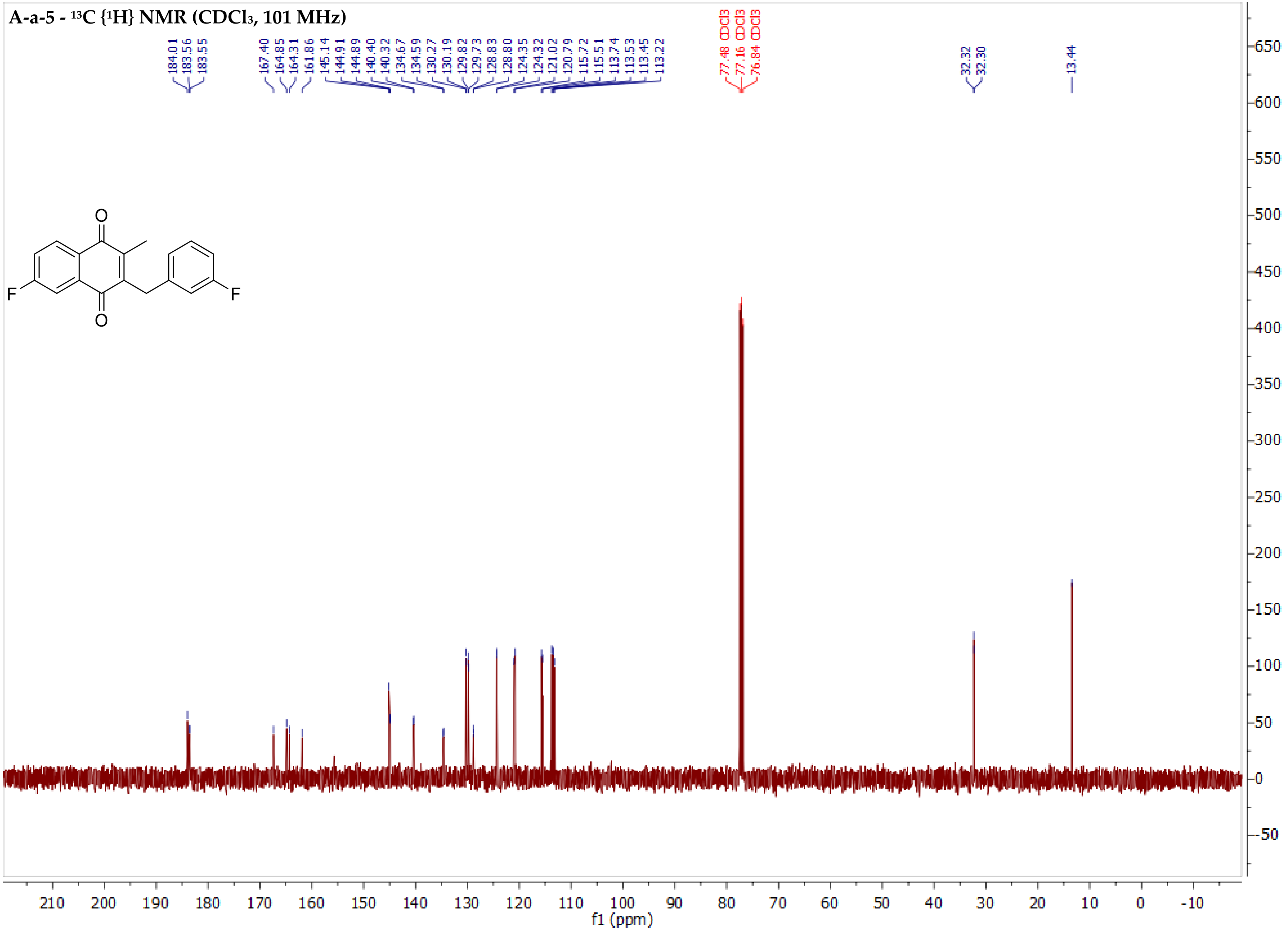

A-a-5 - 13C {1H} NMR (CDCl3, 101 MHz)
184.01
183.56
183.55
167.40
164.85
164.31
161.86
145.14
144.91
144.89
140.40
140.32
134.67
134.59
130.27
130.19
129.82
129.73
128.83
128.80
124.35
124.32
121.02
120.79
115.72
115.51
113.74
113.53
113.45
113.22
77.48 CDCl3
77.16 CDCl3
76.84 CDCl3
32.32
32.30
13.44
O
O
F
F
f1 (ppm)

A-a-5 - ^{19}F NMR (CDCl$_3$, 377 MHz)

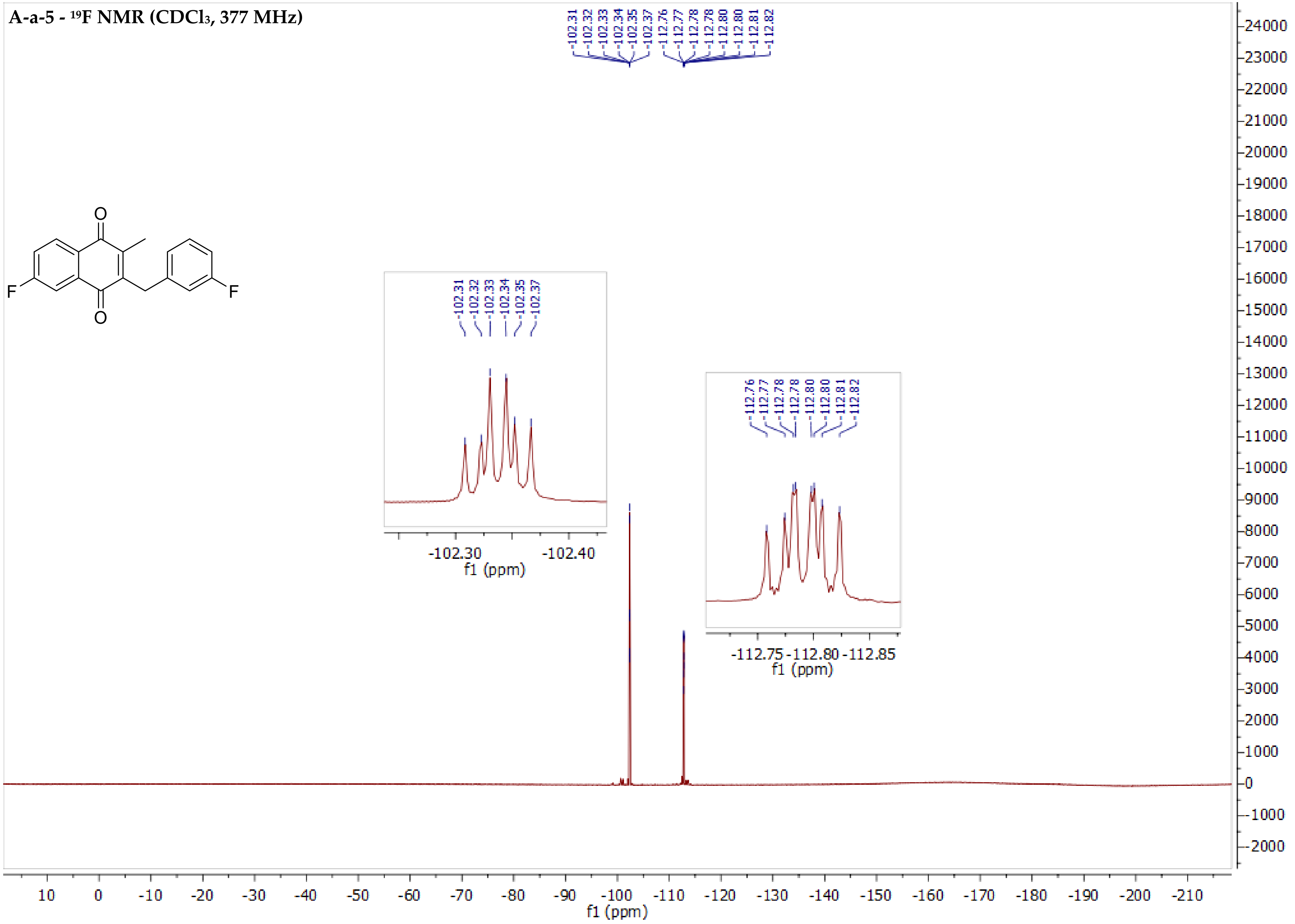

A-a-6 - ^{1}H NMR ($CDCl_3$, 400 MHz)

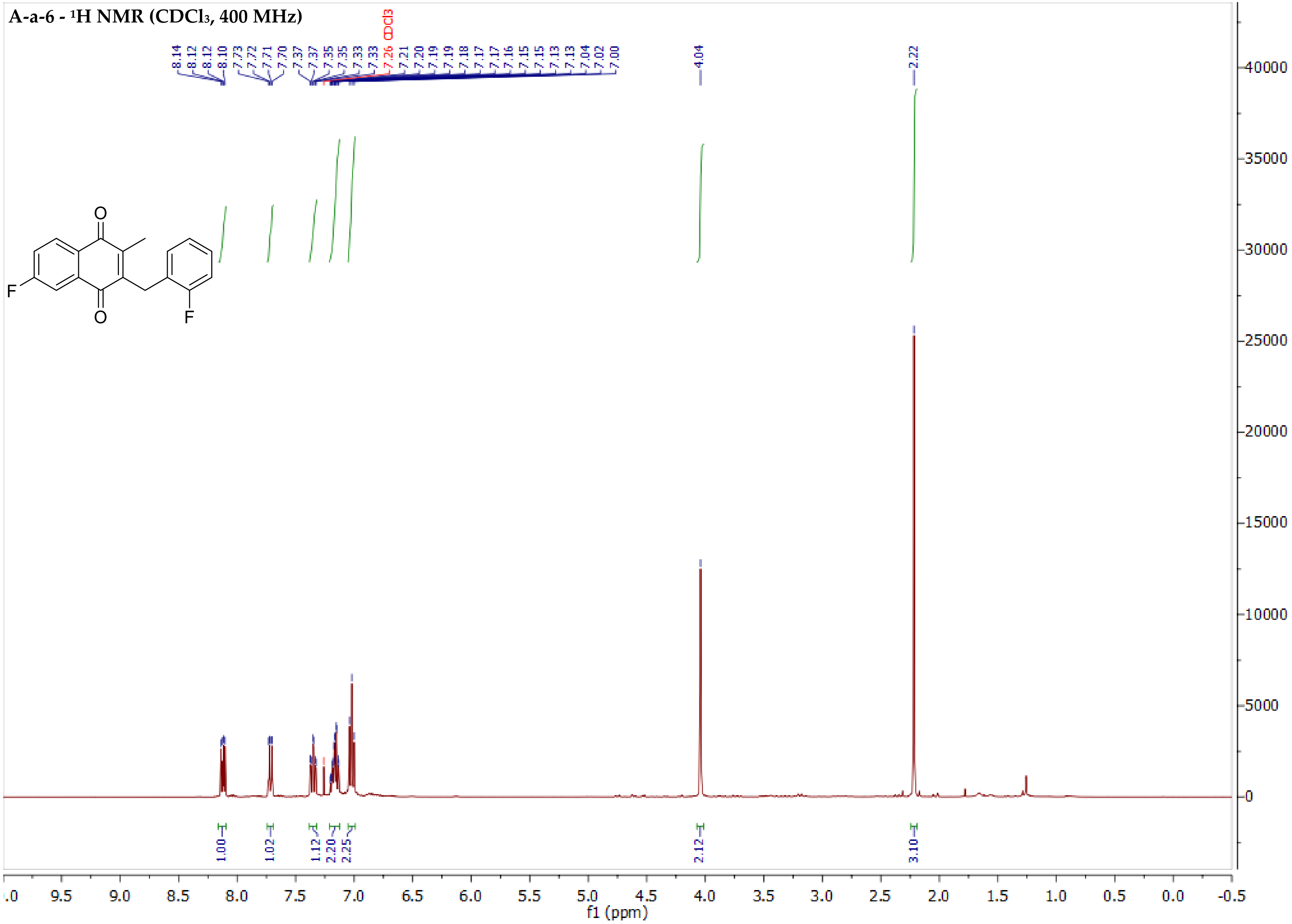

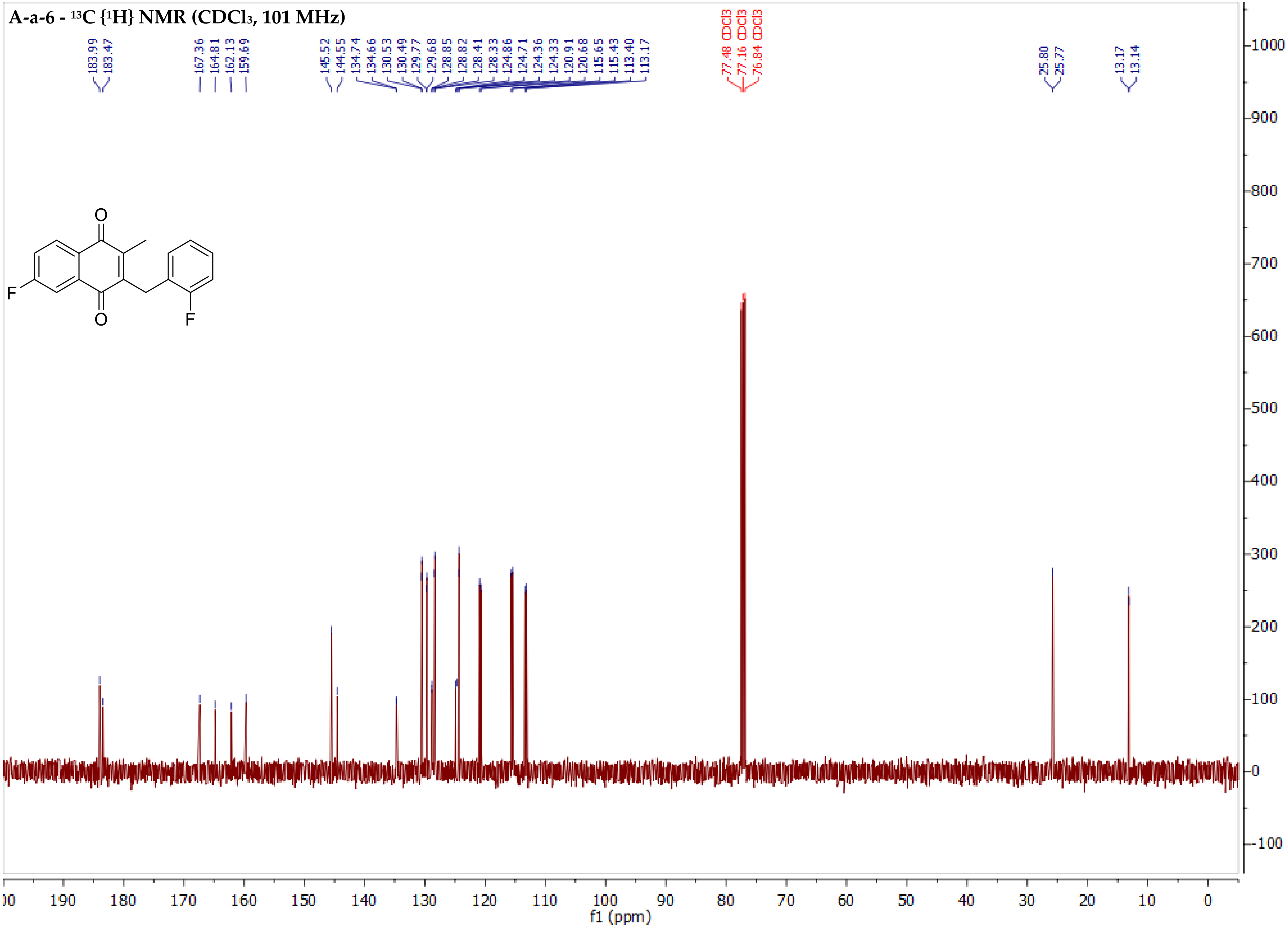
A-a-6 - 13C {1H} NMR (CDCl3, 101 MHz)
183.99
183.47
167.36
164.81
162.13
159.69
145.52
144.55
134.74
134.66
130.53
130.49
129.77
129.68
128.85
128.82
128.41
128.33
124.86
124.71
124.36
124.33
120.91
120.68
115.65
115.43
113.40
113.17
77.48 CDCl3
77.16 CDCl3
76.84 CDCl3
25.80
25.77
13.17
13.14
f1 (ppm)

A-a-6 - ^{19}F NMR ($CDCl_3$, 377 MHz)

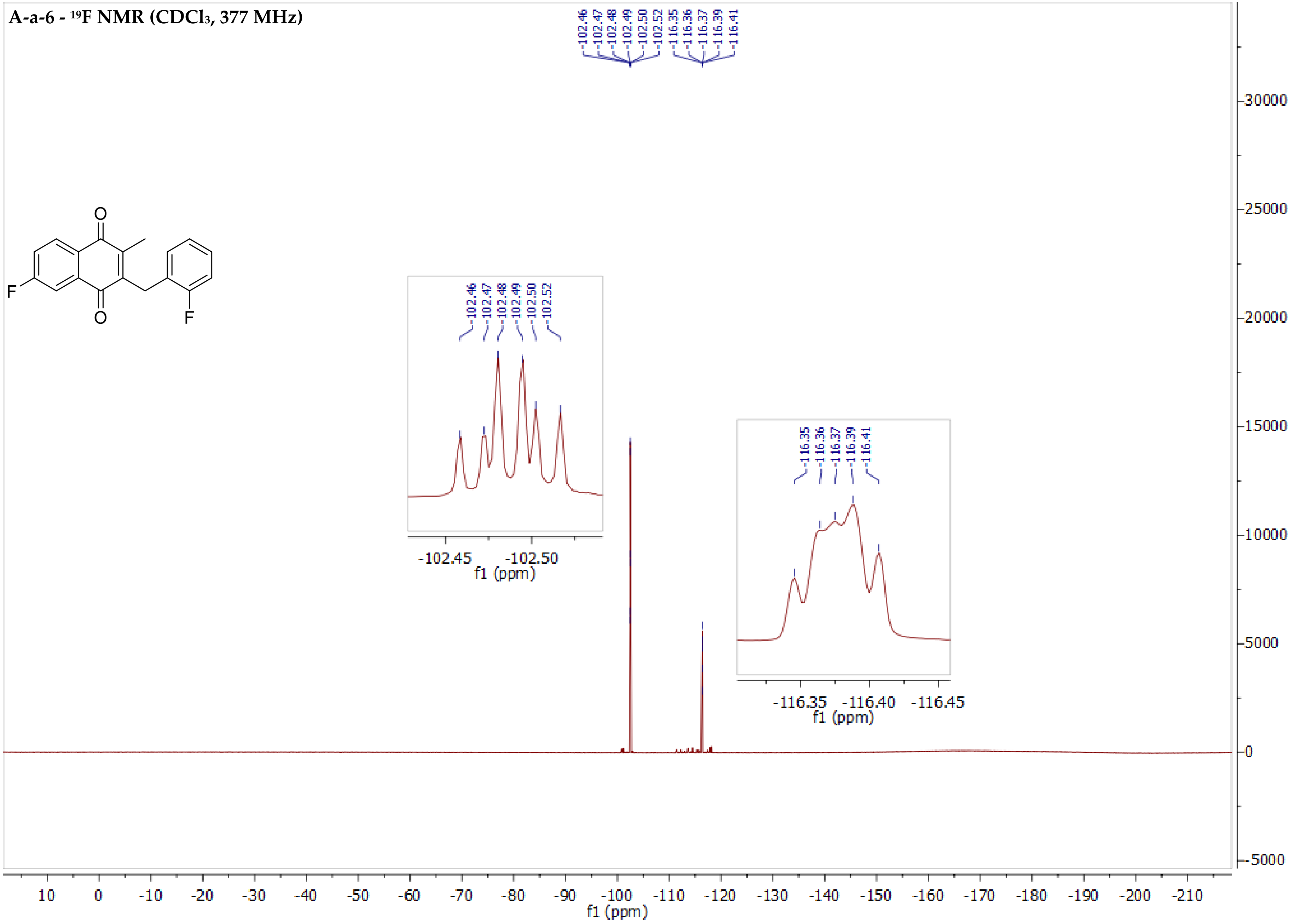

A-a-7 - ^{1}H NMR ($CDCl_3$, 400 MHz)

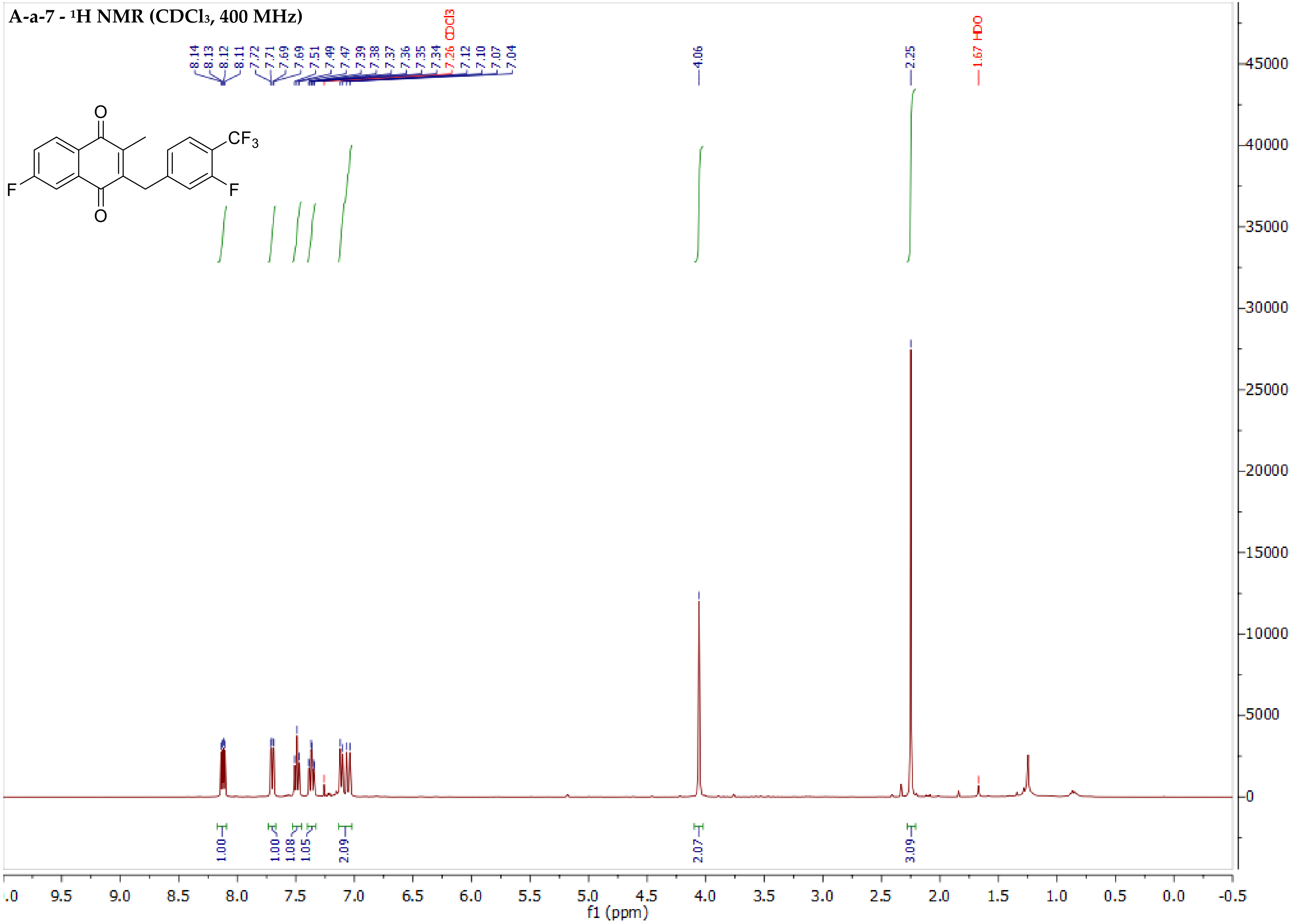

A-a-7 - ^{13}C {^{1}H} NMR ($CDCl_3$, 101 MHz)

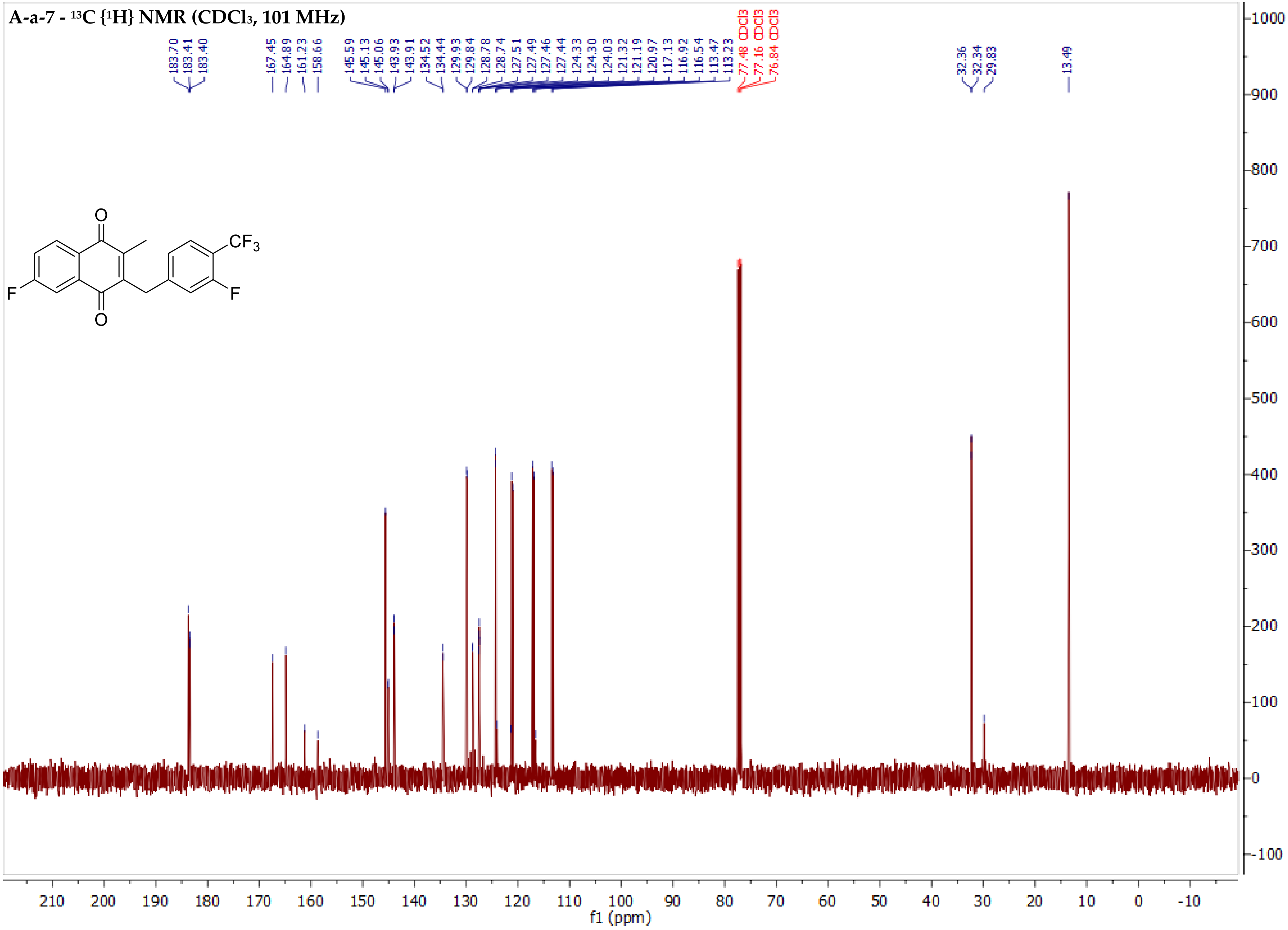

A-a-7 - ^{19}F NMR (CDCl$_3$, 377 MHz)

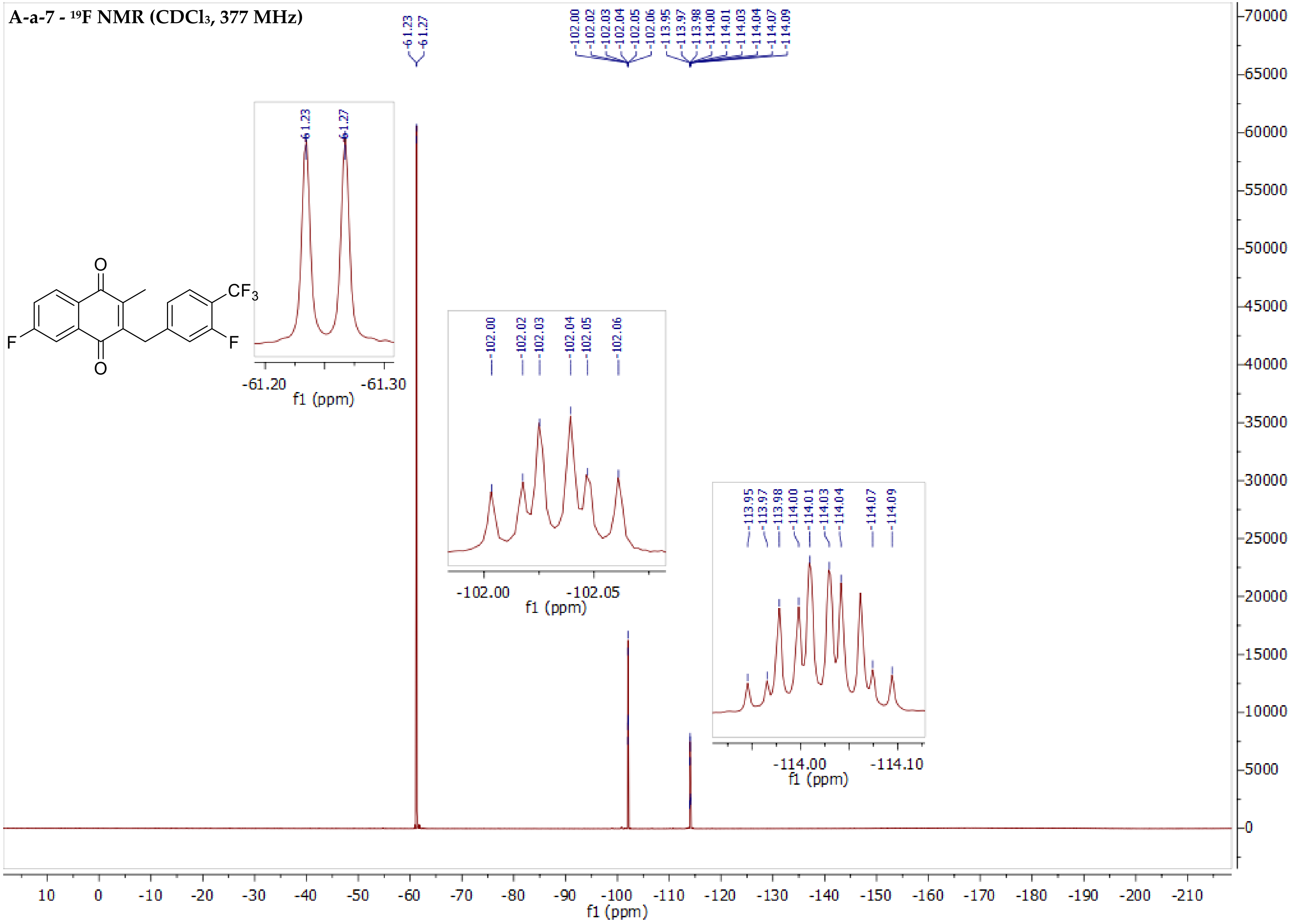

A-a-8 - ^{1}H NMR (CDCl$_3$, 400 MHz)

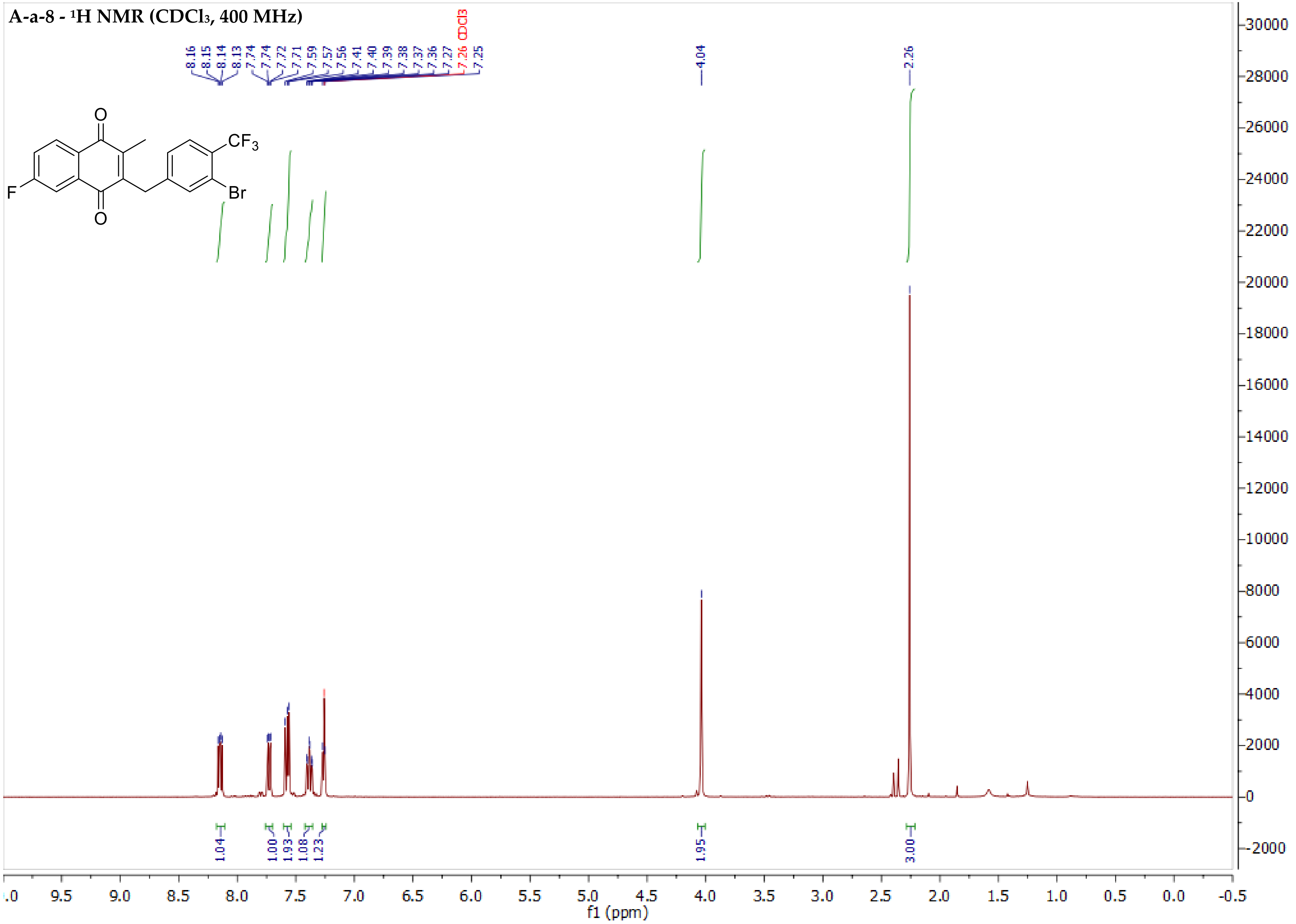

A-a-8 - $^{13}C\{^1H\}$ NMR ($CDCl_3$, 101 MHz)

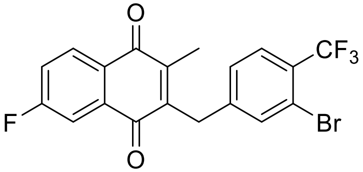

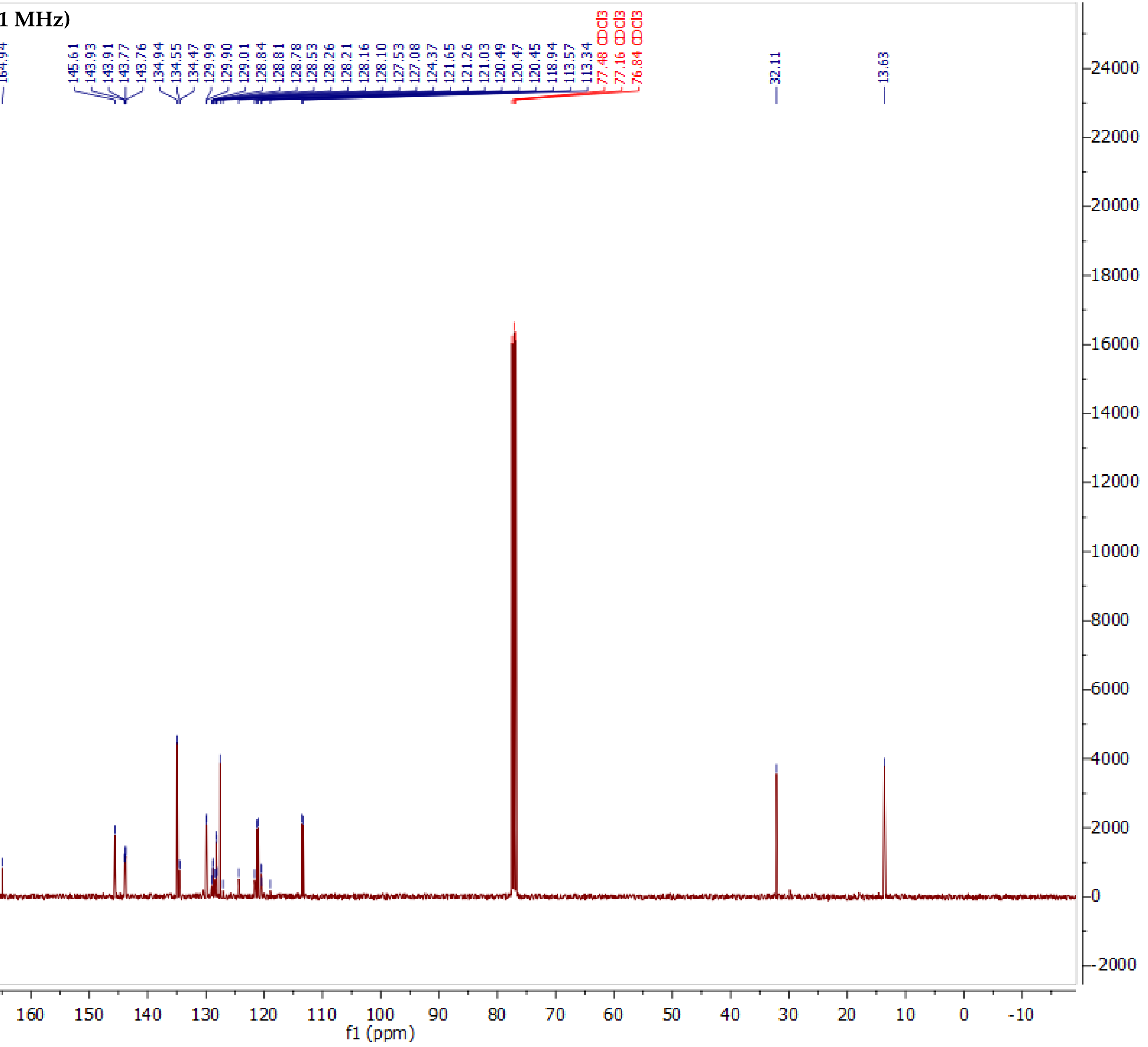

A-a-8 - ^{19}F NMR ($CDCl_3$, 377 MHz)

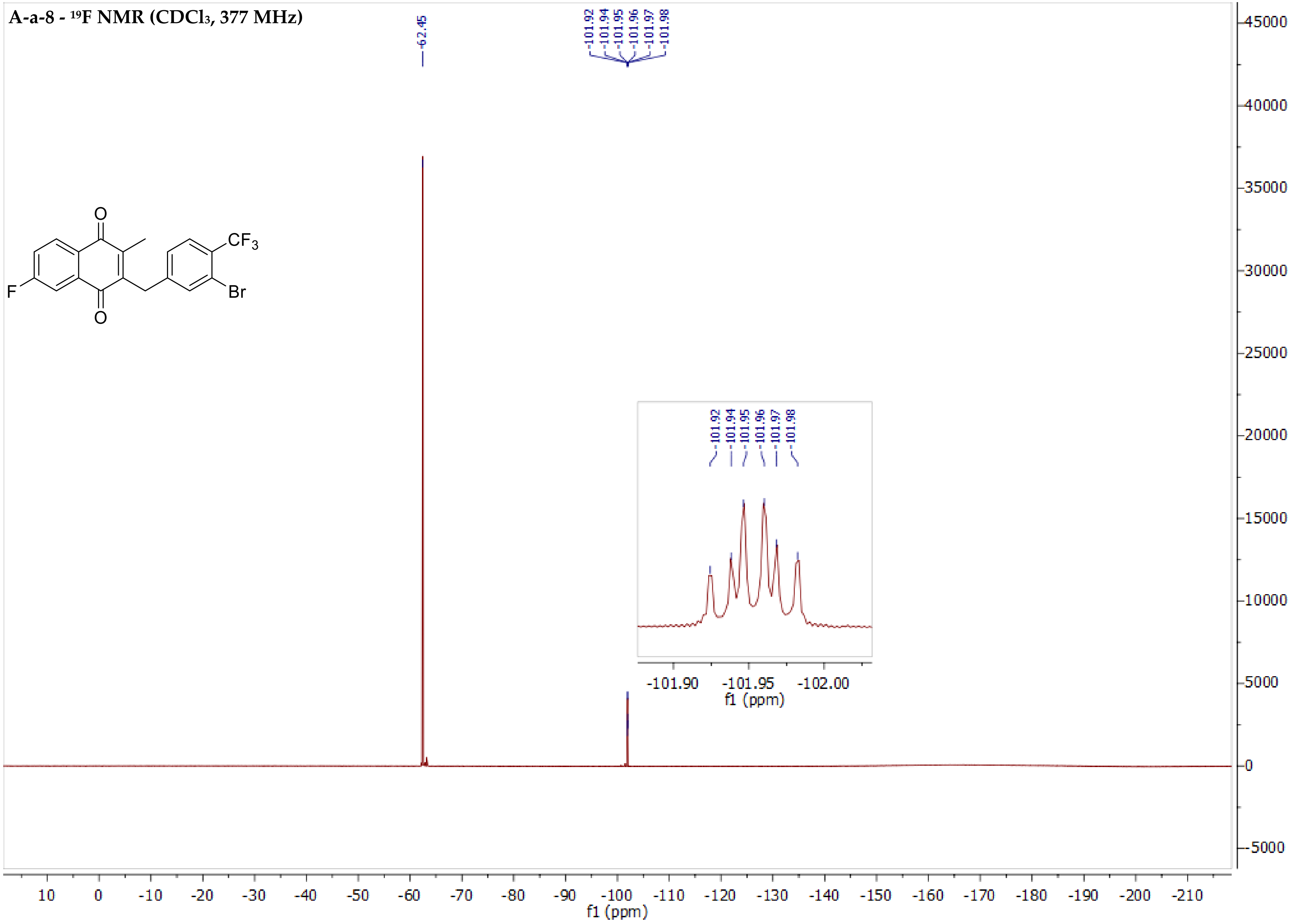

A-a-9 - ^{1}H NMR ($CDCl_3$, 400 MHz)

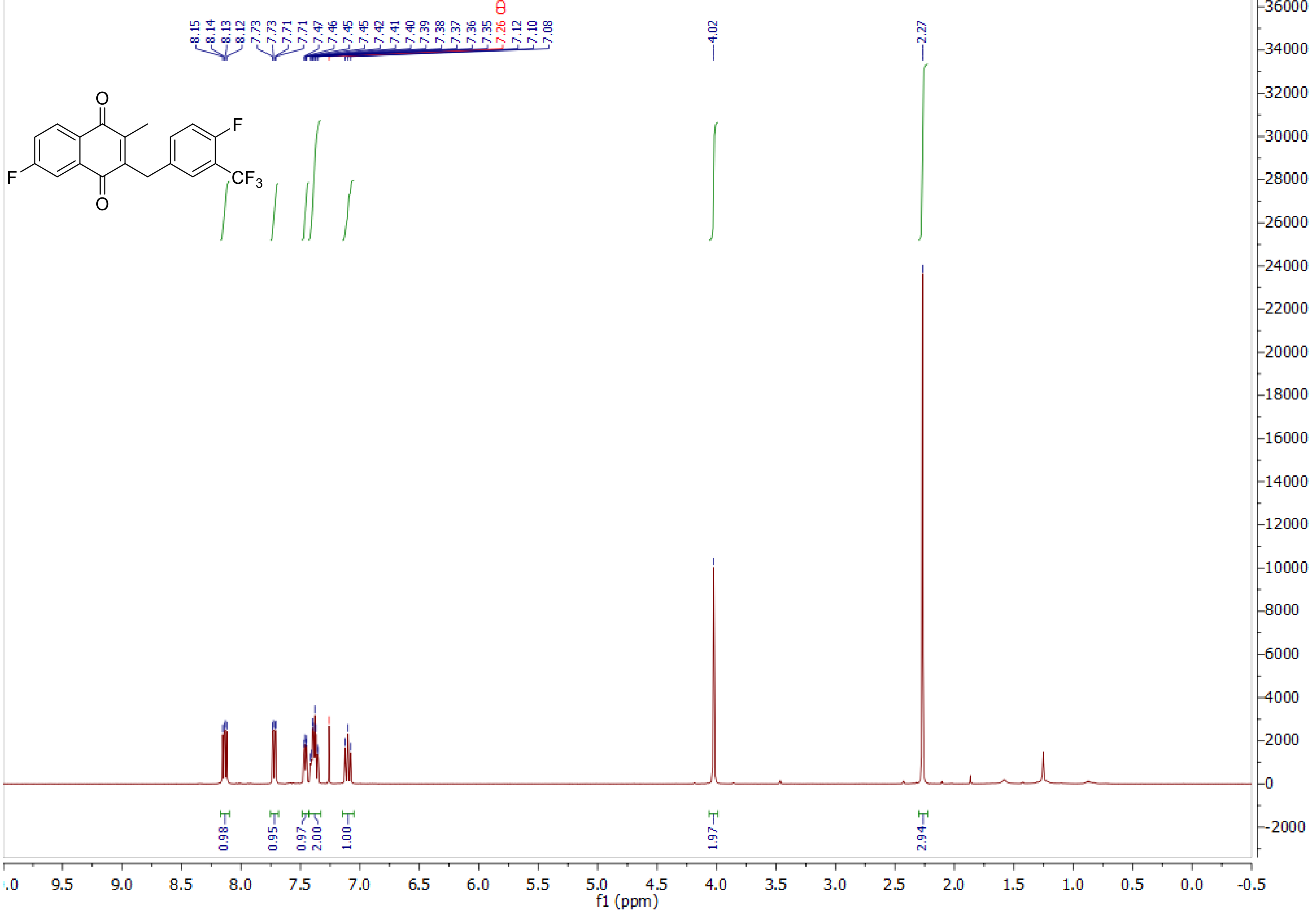

A-a-9 - $^{13}C\{^1H\}$ NMR ($CDCl_3$, 101 MHz)

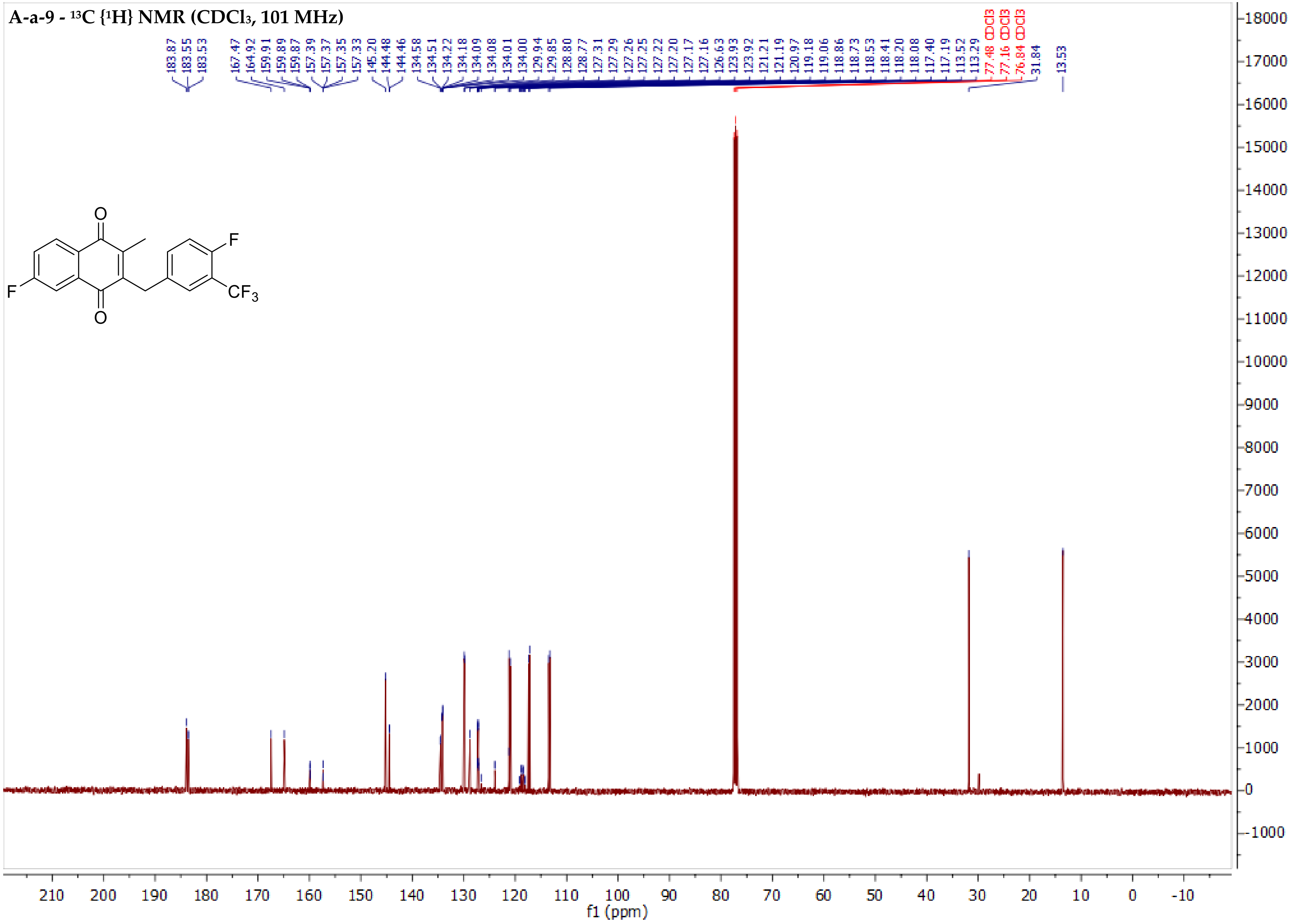

A-a-9 - ^{19}F NMR ($CDCl_3$, 377 MHz)

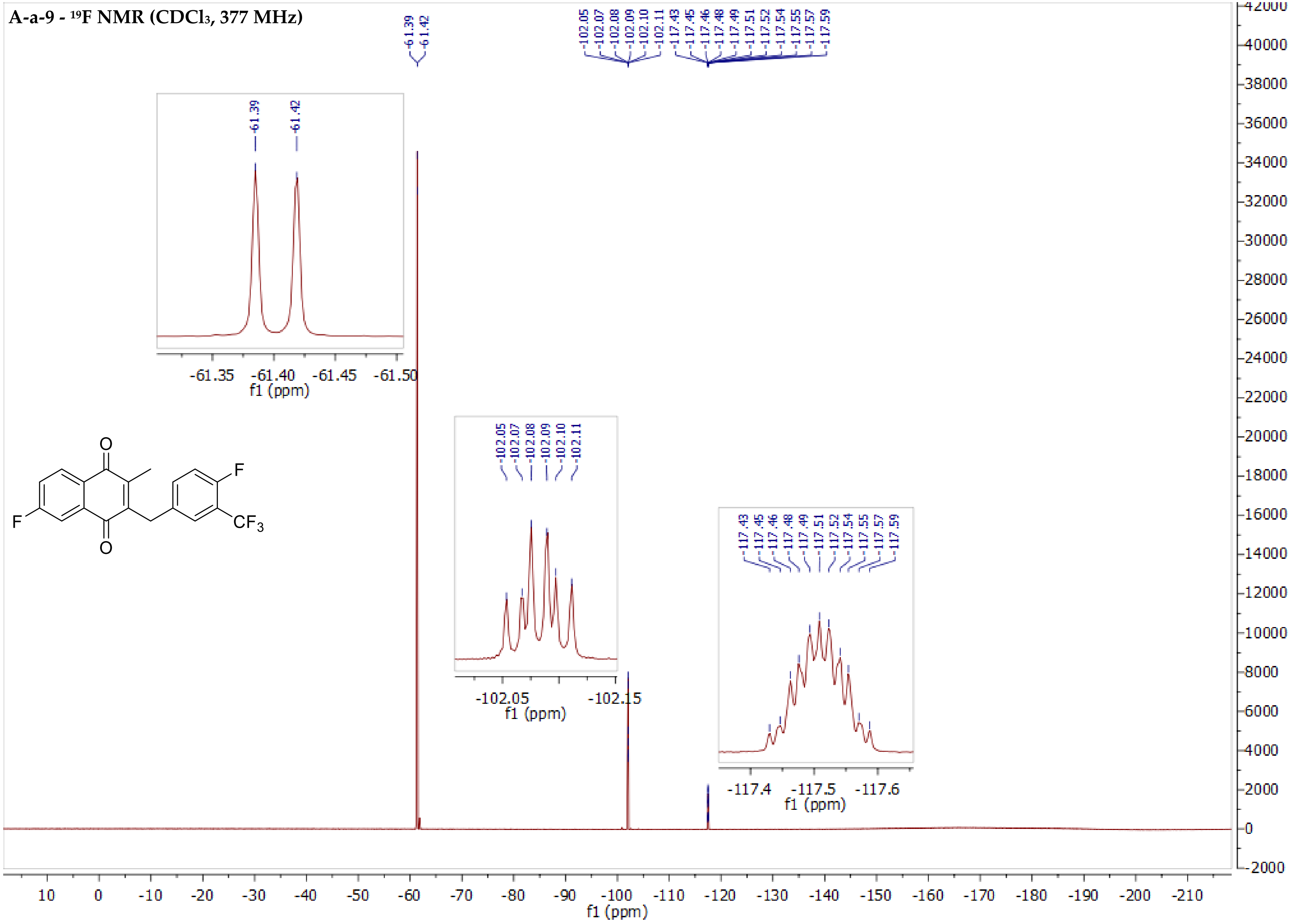

A-a-10 - ¹H NMR (CDCl₃, 400 MHz)

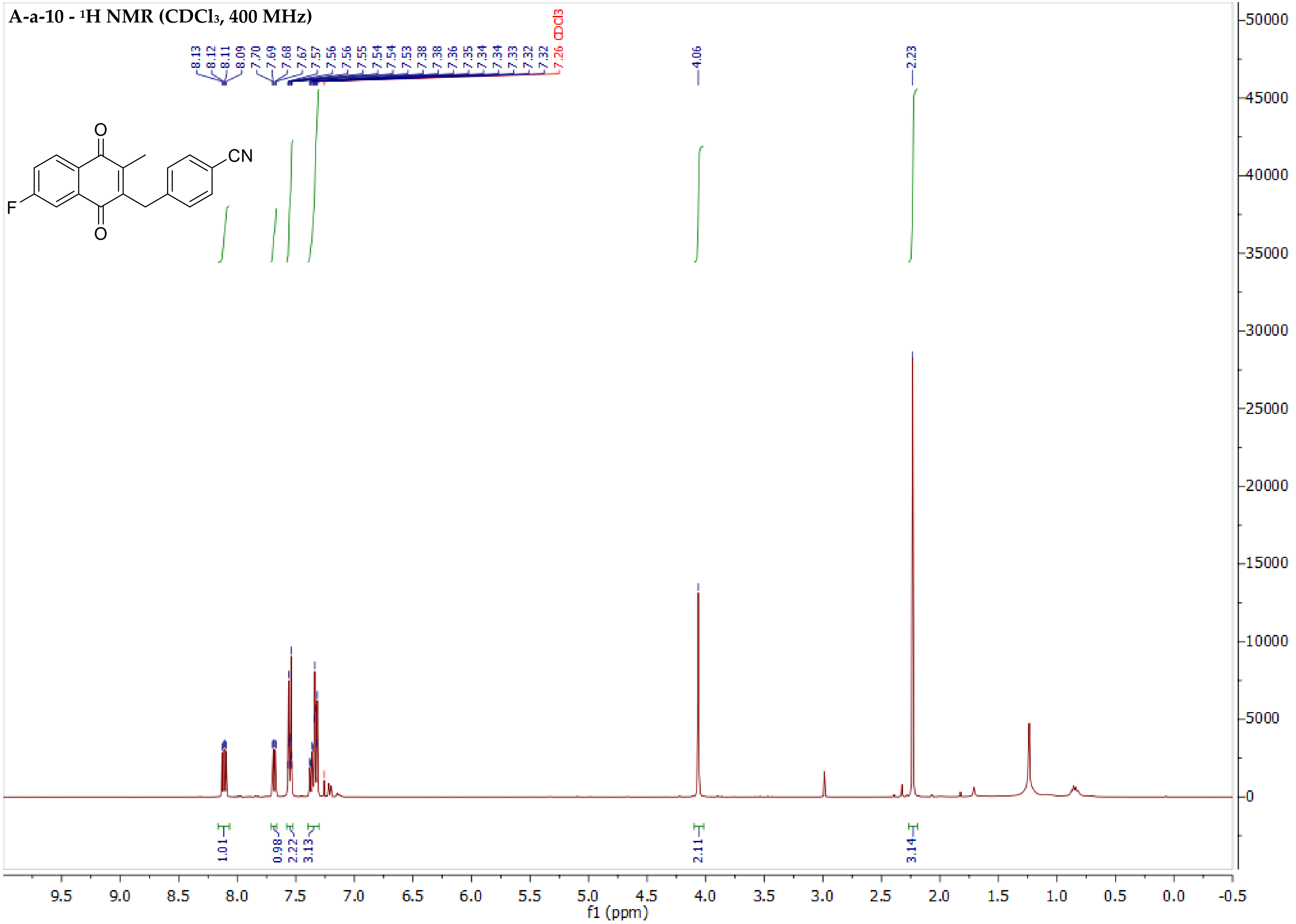

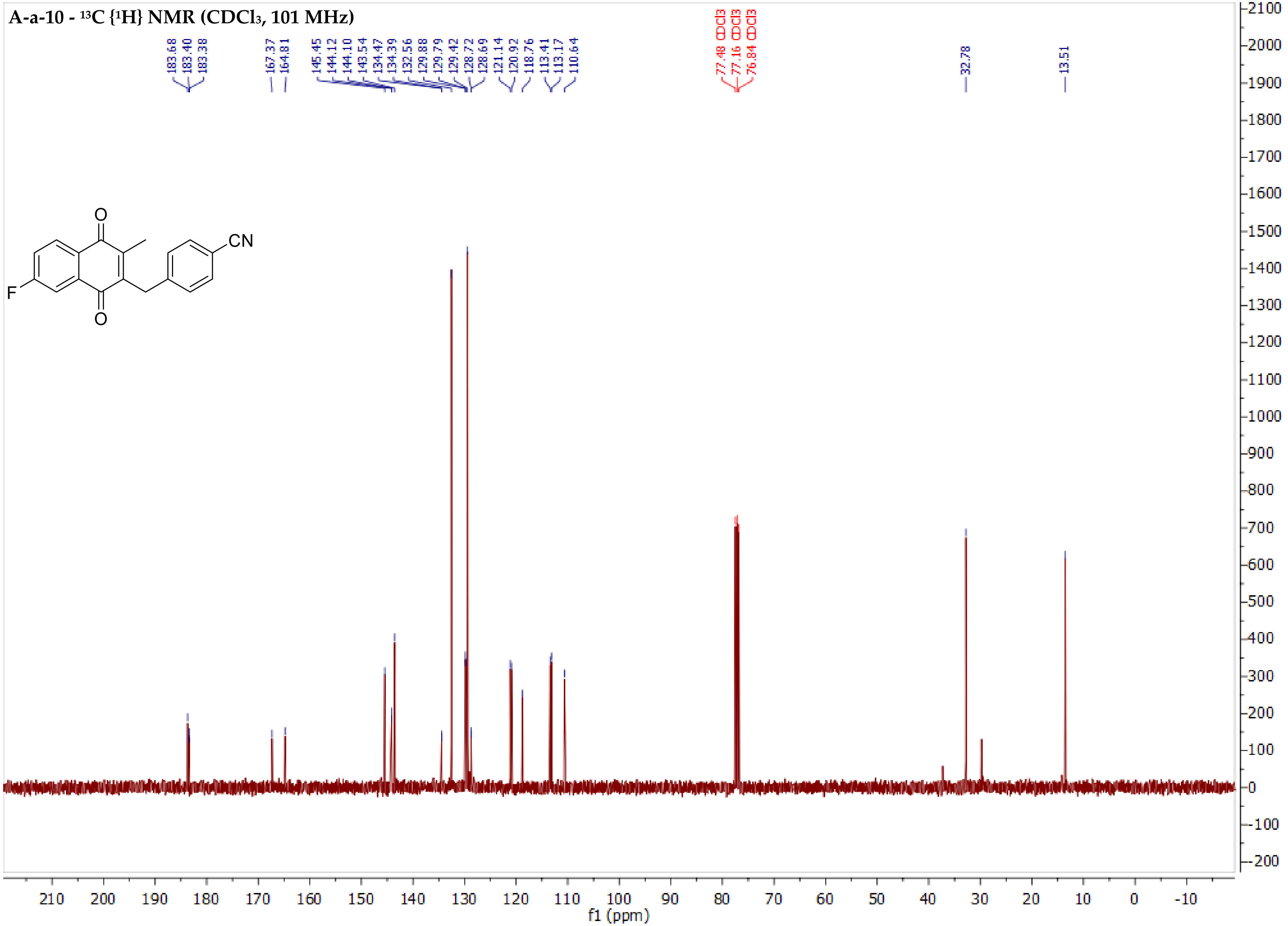
A-a-10 - ^{13}C {^1H} NMR ($CDCl_3$, 101 MHz)
183.68
183.40
183.38
167.37
164.81
145.45
144.12
144.10
143.54
134.47
134.39
132.56
129.88
129.79
129.42
128.72
128.69
121.14
120.92
118.76
113.41
113.17
110.64
77.48 CDCl3
77.16 CDCl3
76.84 CDCl3
32.78
13.51
O
CN
F
O
f1 (ppm)

A-a-10 - ¹⁹F NMR (CDCl₃, 377 MHz)

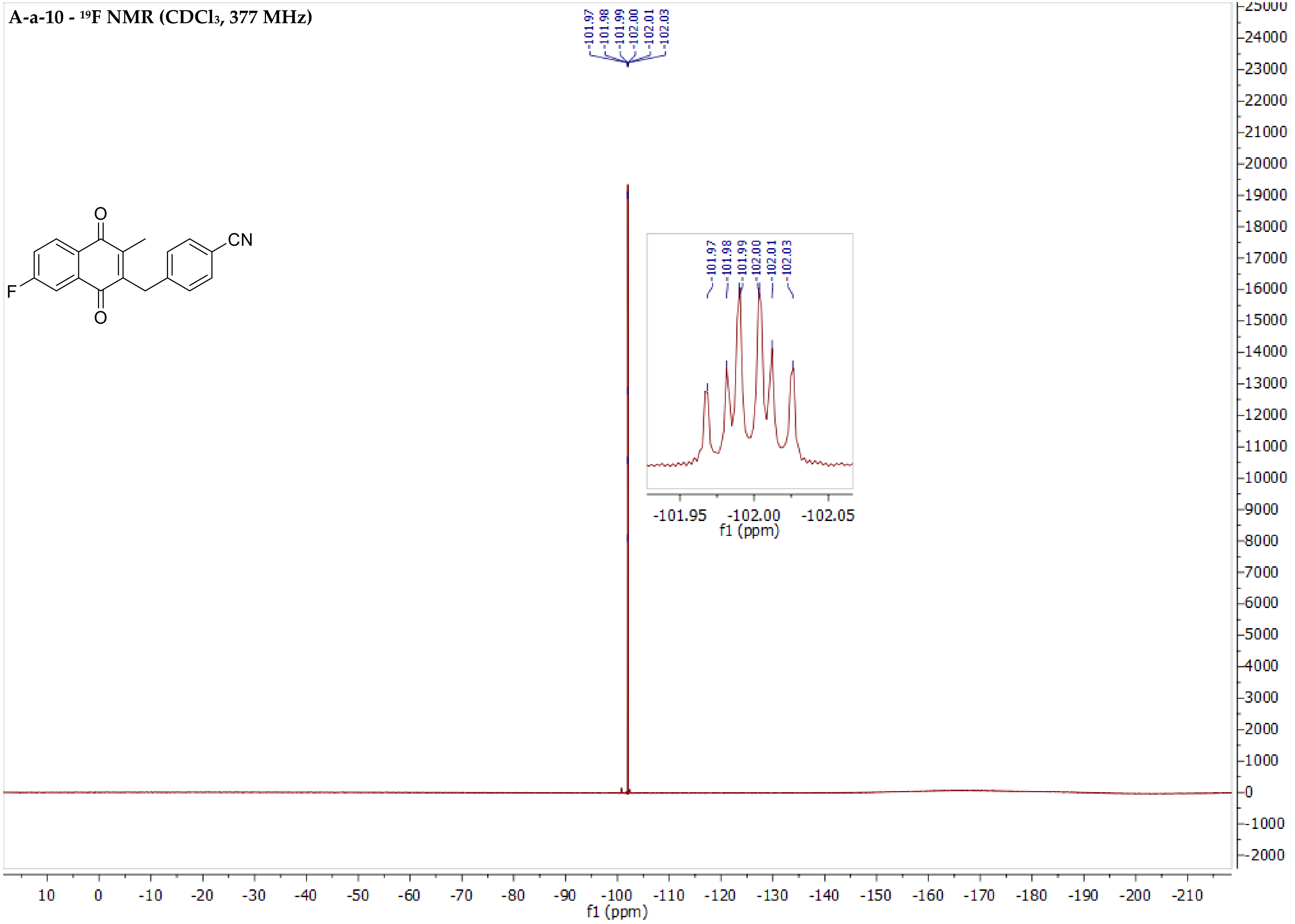

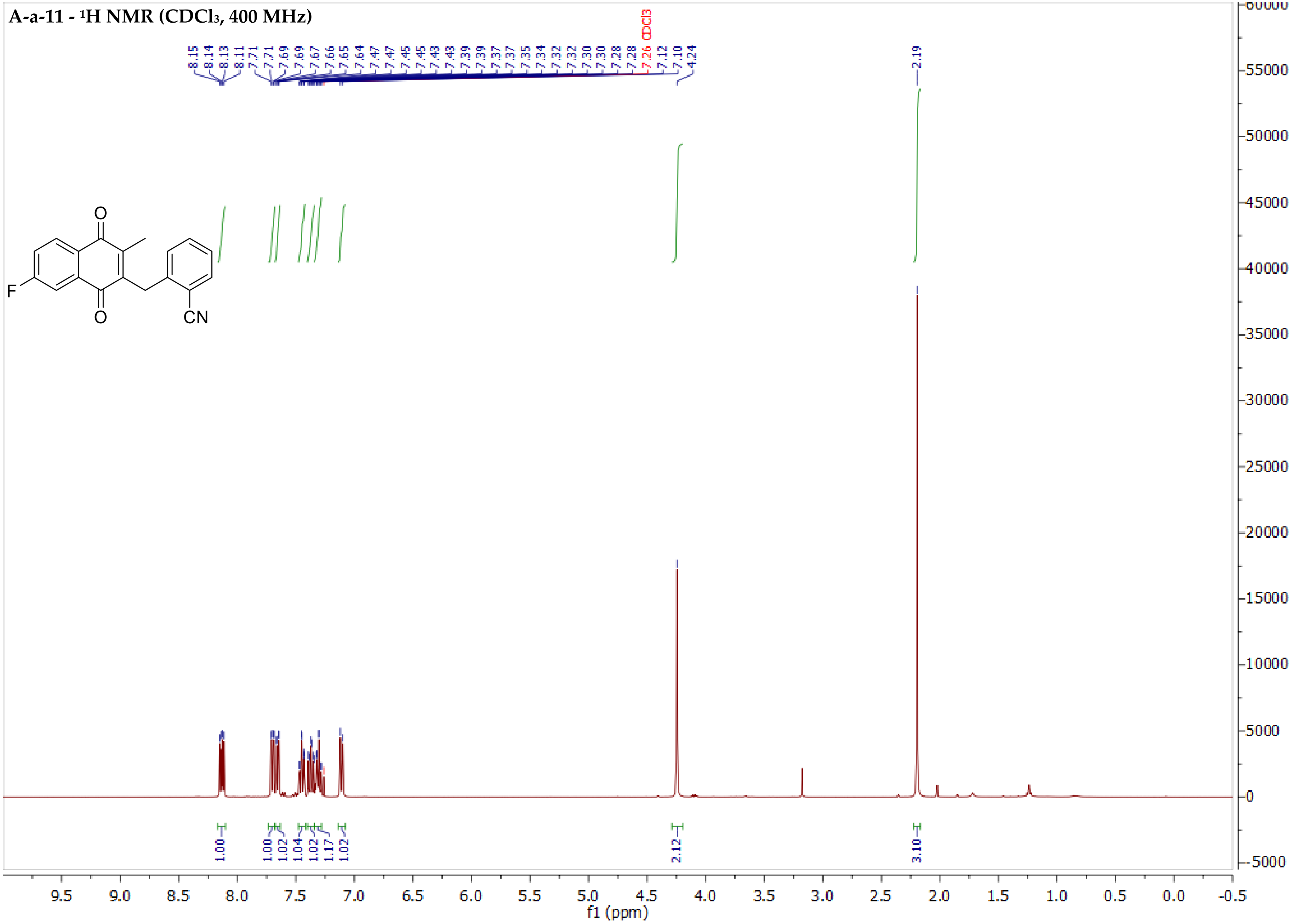
A-a-11 - ¹H NMR (CDCl3, 400 MHz)
F
O
O
CN
8.15
8.14
8.13
8.11
7.71
7.71
7.69
7.69
7.67
7.66
7.65
7.64
7.47
7.47
7.45
7.45
7.43
7.43
7.39
7.39
7.37
7.37
7.35
7.34
7.32
7.32
7.30
7.30
7.28
7.28
7.26 CDCl3
7.12
7.10
4.24
2.19
1.00
1.00
1.02
1.04
1.02
1.17
1.02
2.12
3.10
f1 (ppm)

A-a-11 - $^{13}C\{^{1}H\}$ NMR ($CDCl_3$, 101 MHz)

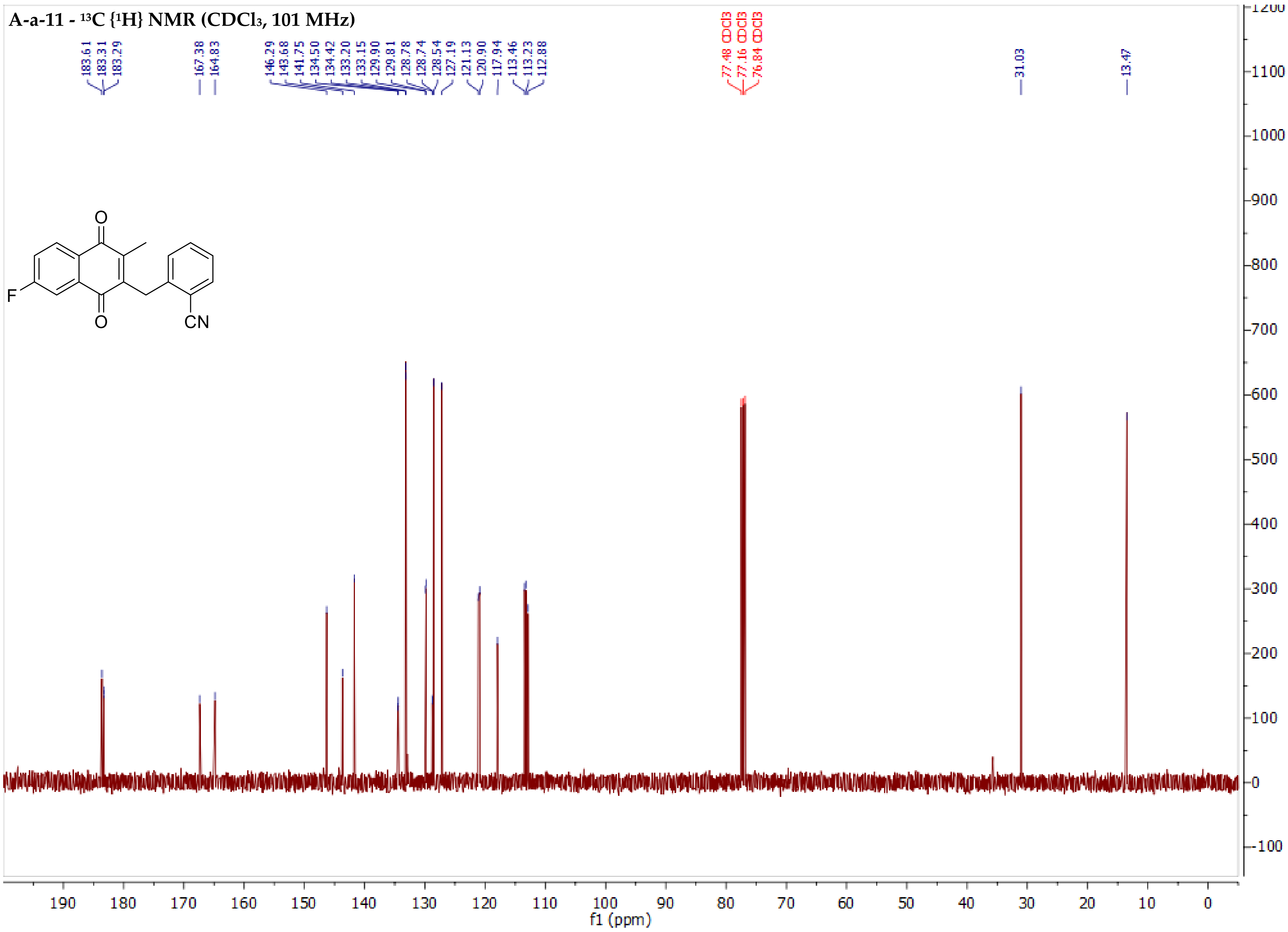

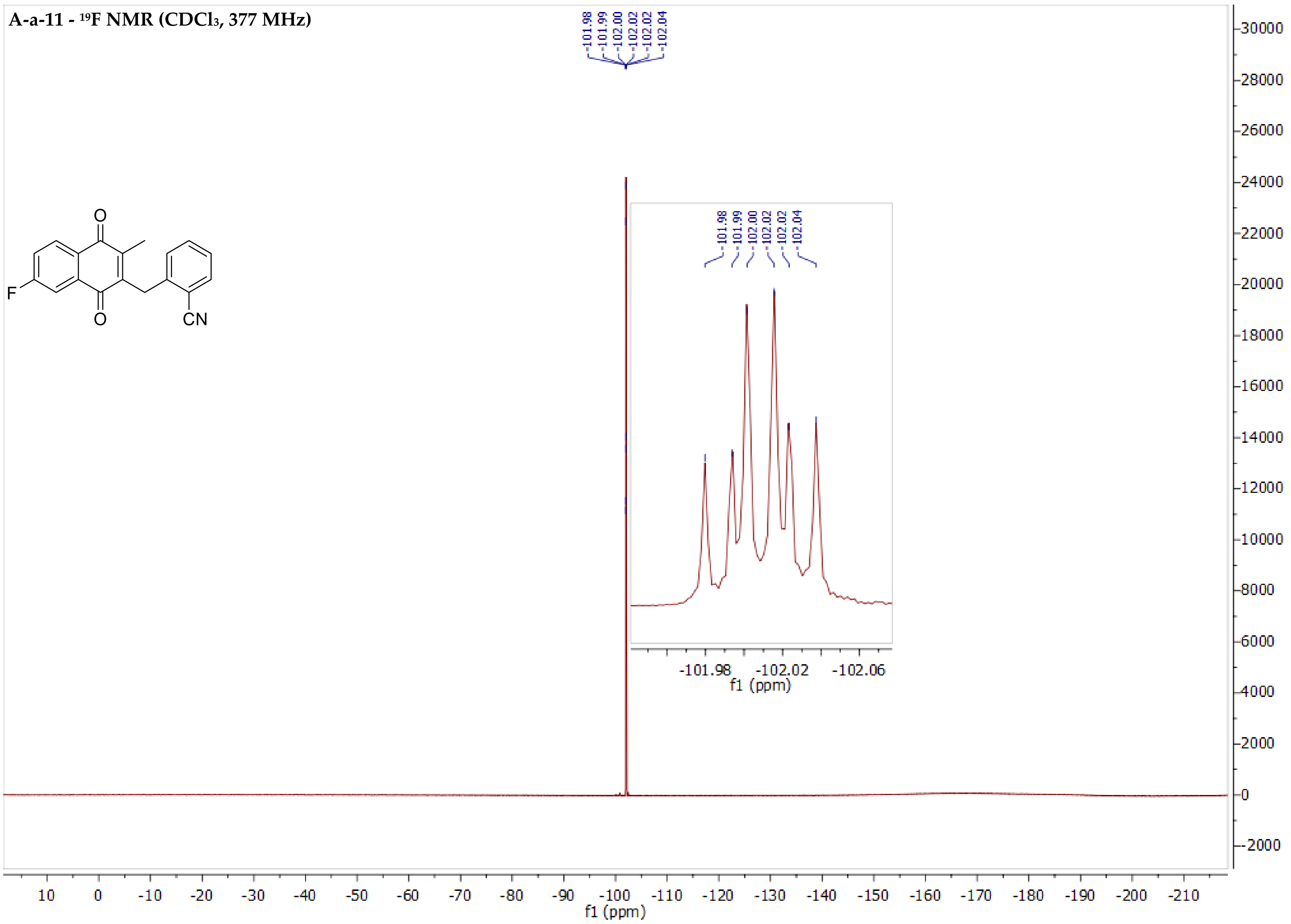
A-a-11 - ¹⁹F NMR (CDCl₃, 377 MHz)
-101.98
-101.99
-102.00
-102.02
-102.02
-102.04
O
O
F
CN
-101.98
-102.02
-102.06
f1 (ppm)
30000
28000
26000
24000
22000
20000
18000
16000
14000
12000
10000
8000
6000
4000
2000
0
-2000
10
0
-10
-20
-30
-40
-50
-60
-70
-80
-90
-100
-110
-120
-130
-140
-150
-160
-170
-180
-190
-200
-210
f1 (ppm)

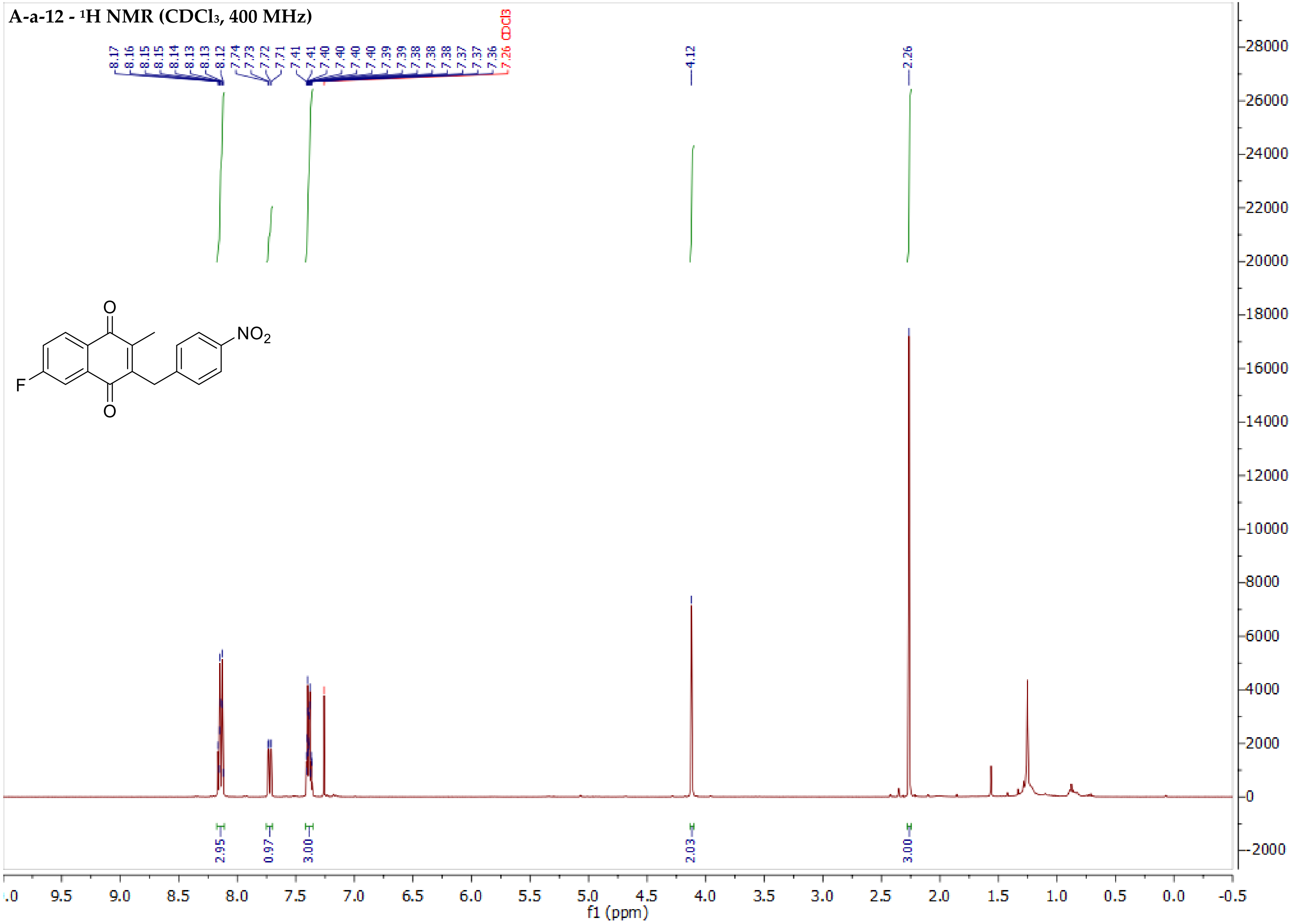
A-a-12 - ¹H NMR (CDCl₃, 400 MHz)
8.17 8.16 8.15 8.15 8.14 8.13 8.13 8.12 7.74 7.73 7.72 7.71 7.41 7.41 7.40 7.40 7.40 7.40 7.39 7.39 7.38 7.38 7.37 7.37 7.36 7.26 CDCl3
4.12
2.26
NO2
F
O
O
2.95
0.97
3.00
2.03
3.00
f1 (ppm)

A-a-12 - $^{13}C\{^1H\}$ NMR ($CDCl_3$, 101 MHz)

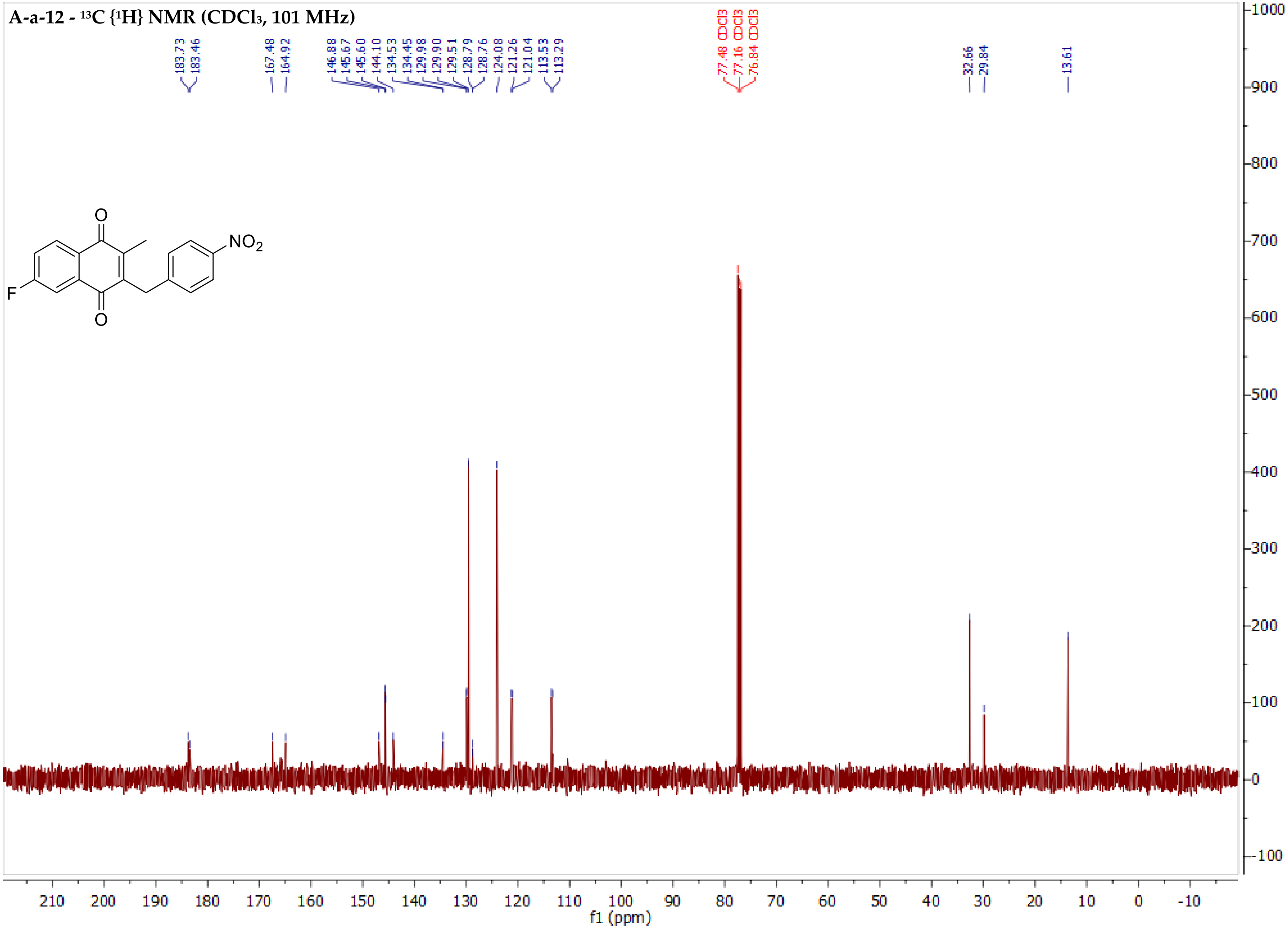

A-a-12 - ^{19}F NMR ($CDCl_3$, 377 MHz)

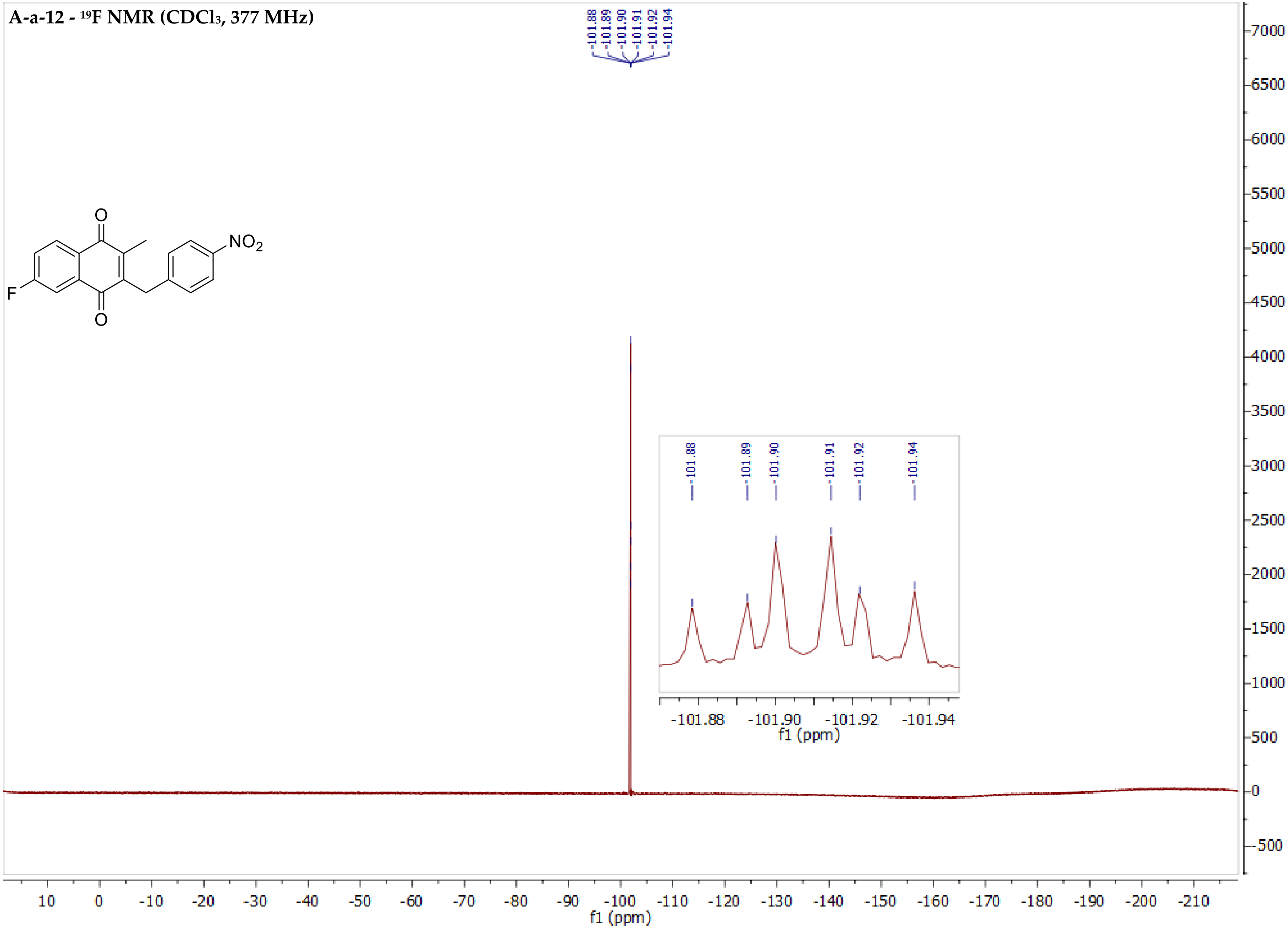

A-a-13 - ^{1}H NMR ($CDCl_3$, 400 MHz)

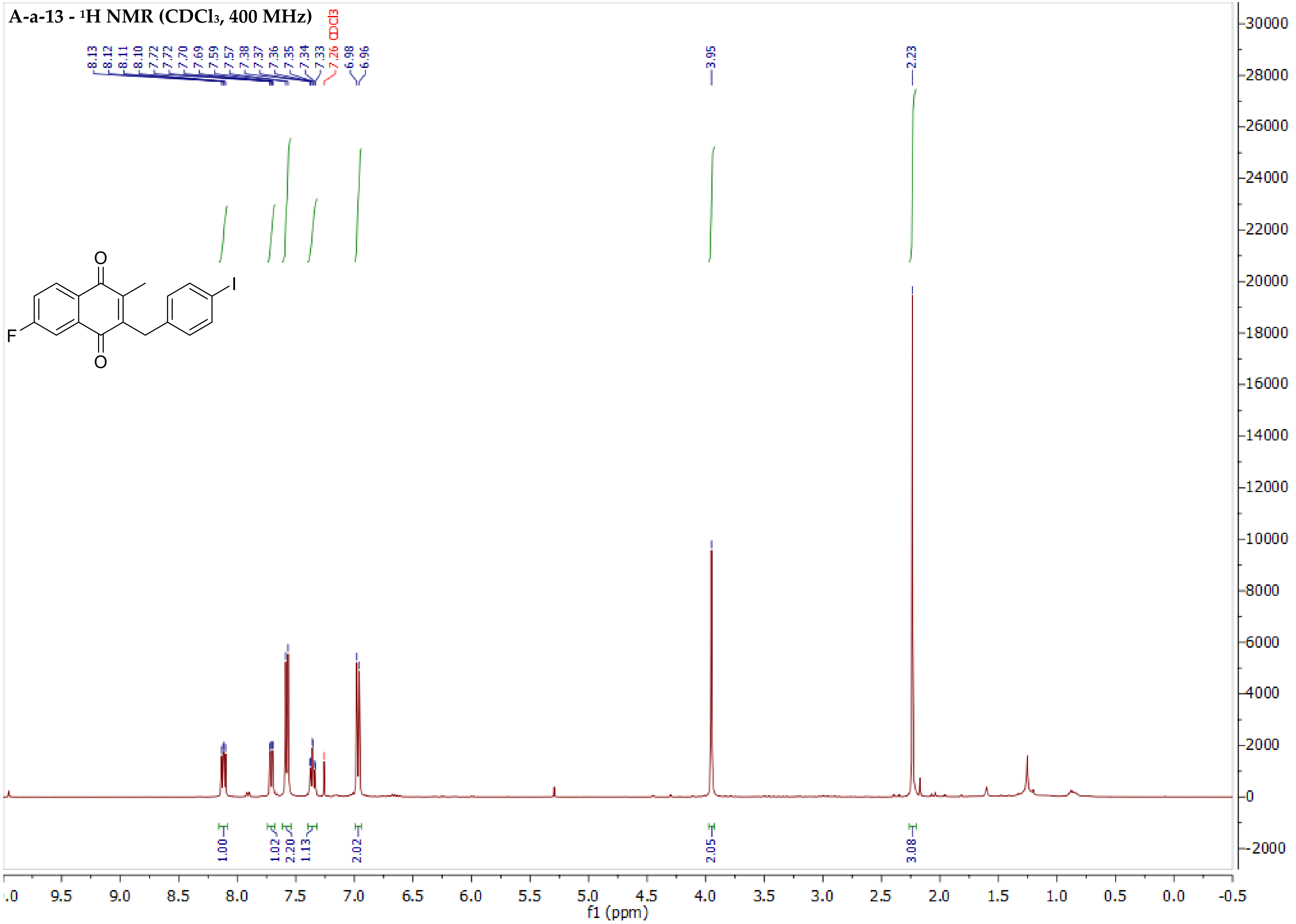

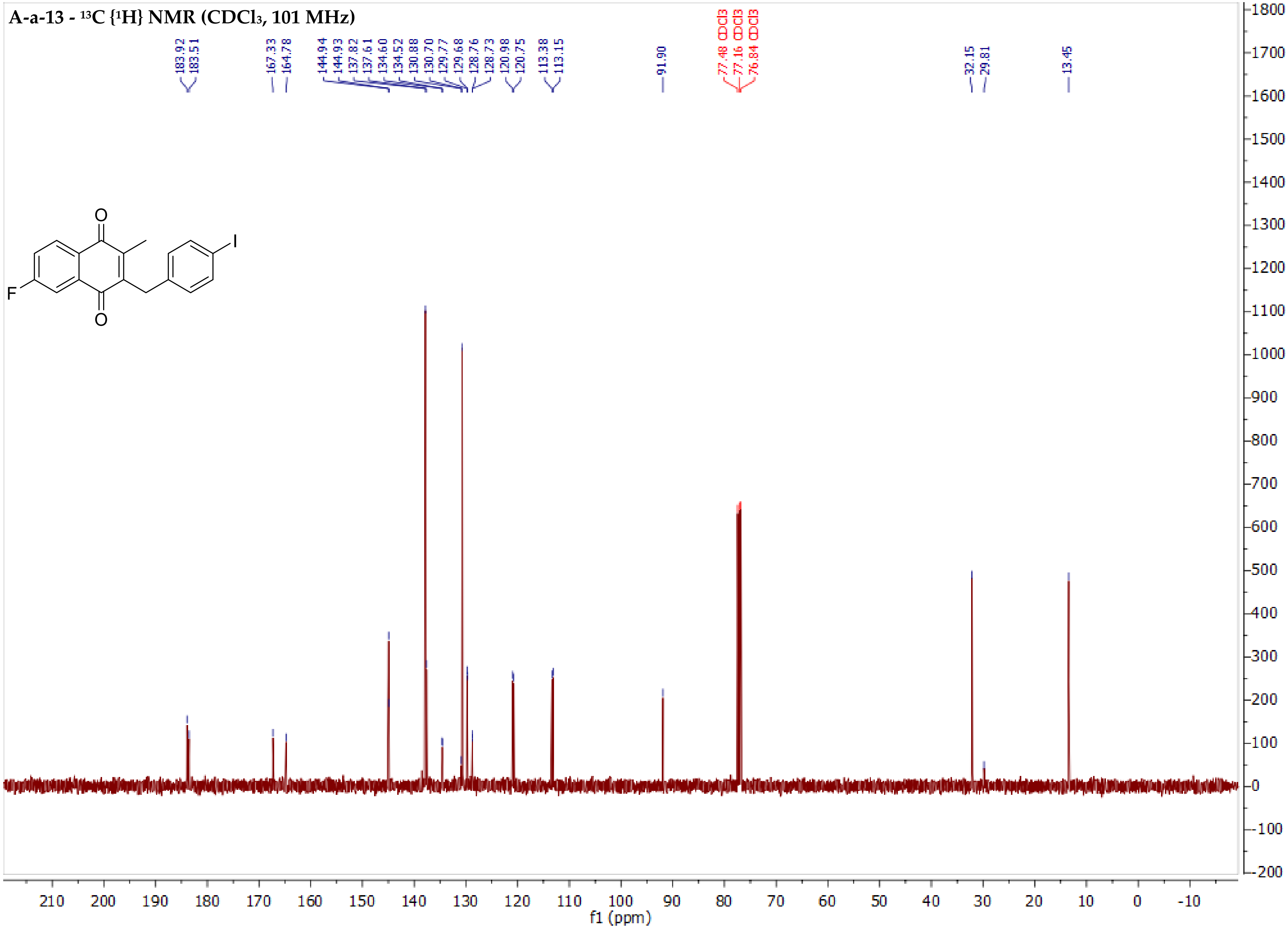
A-a-13 - ¹³C {¹H} NMR (CDCl₃, 101 MHz)
183.92
183.51
167.33
164.78
144.94
144.93
137.82
137.61
134.60
134.52
130.88
130.70
129.77
129.68
128.76
128.73
120.98
120.75
113.38
113.15
91.90
77.48 CDCl3
77.16 CDCl3
76.84 CDCl3
32.15
29.81
13.45
F
O
O
I
f1 (ppm)

A-a-13 - ^{19}F NMR (CDCl$_3$, 377 MHz)

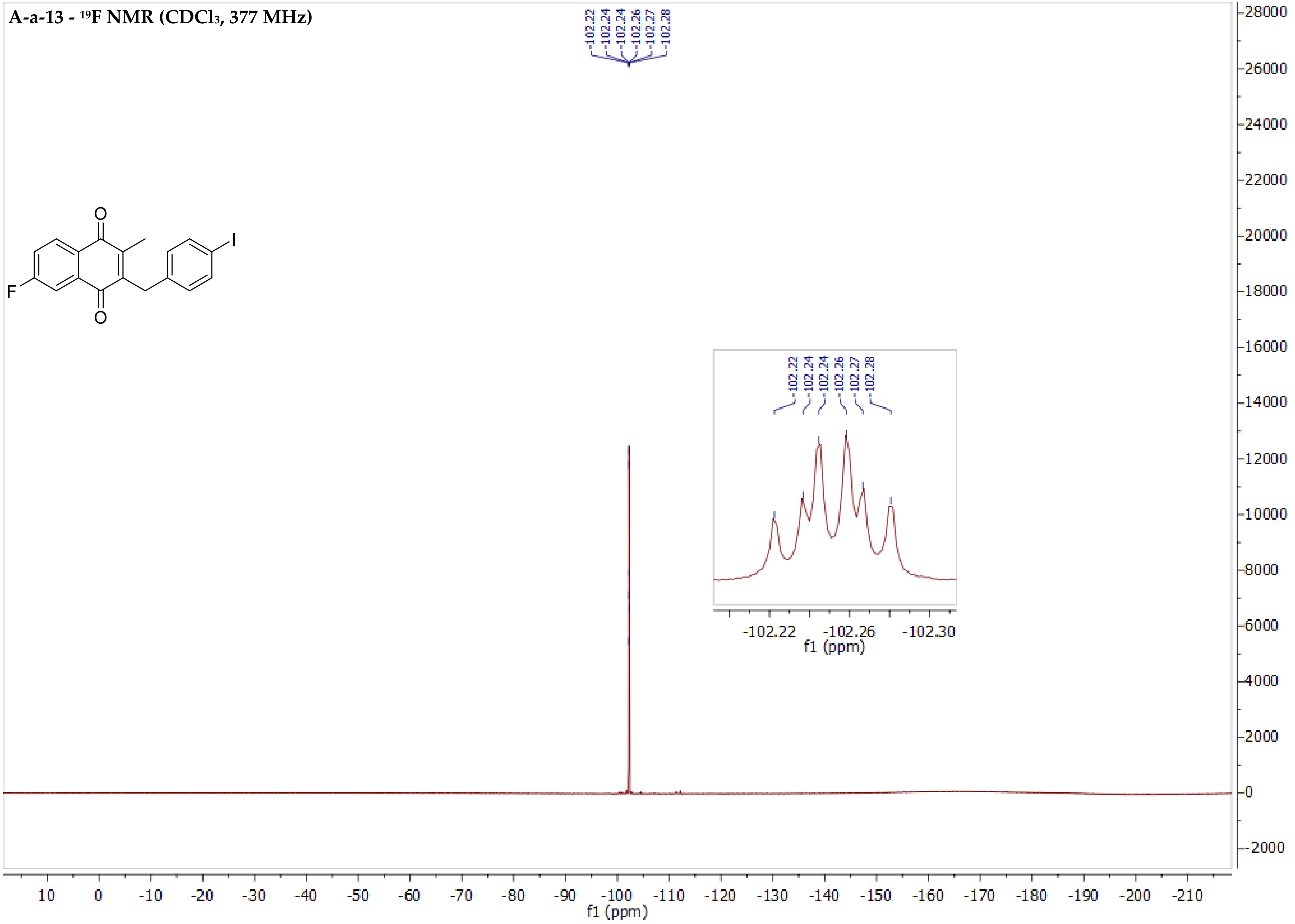

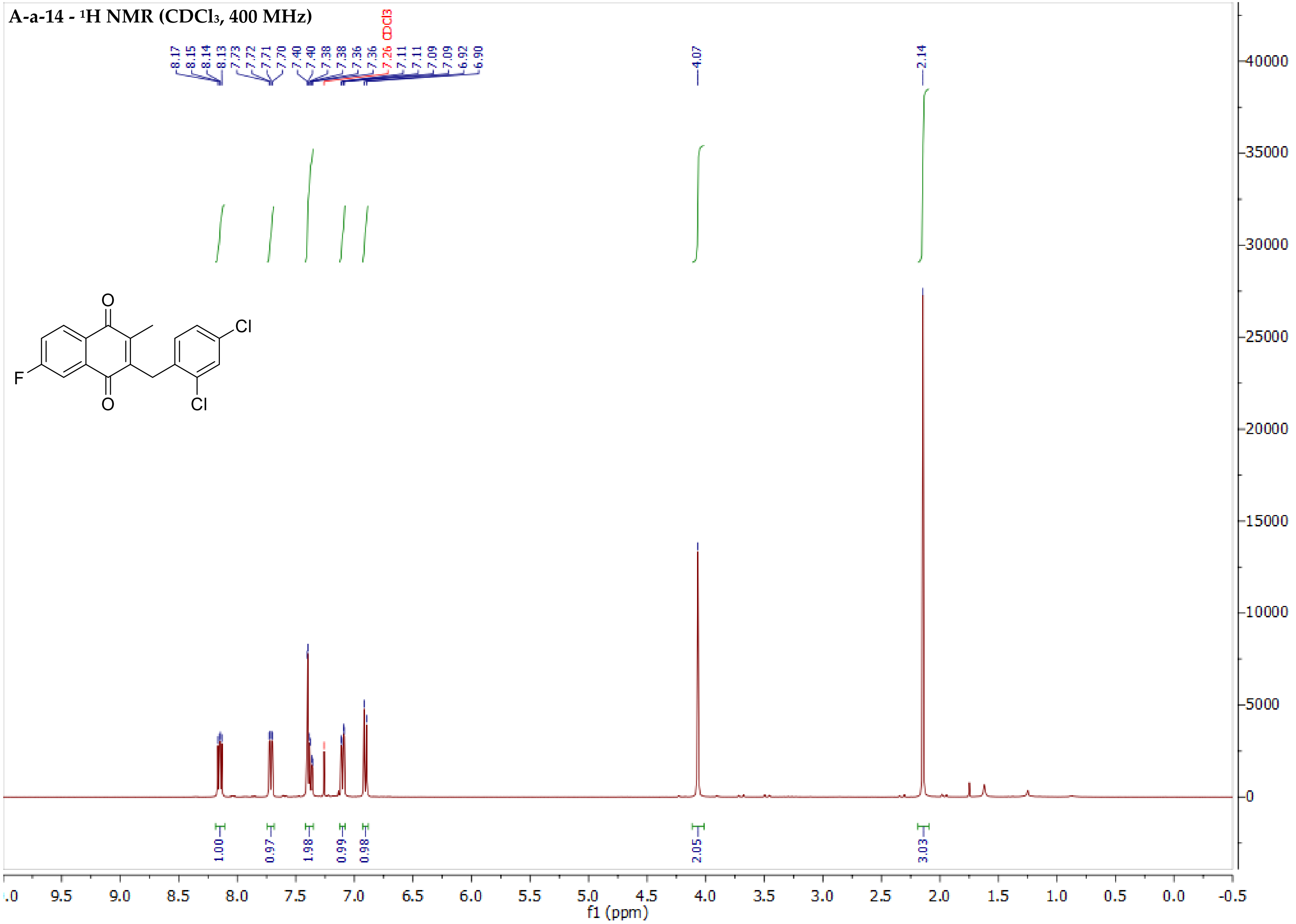
A-a-14 - ¹H NMR (CDCl₃, 400 MHz)
8.17 8.15 8.14 8.13 7.73 7.72 7.71 7.70 7.40 7.40 7.38 7.38 7.36 7.36 7.26 CDCl3 7.11 7.11 7.09 7.09 6.92 6.90
4.07
2.14
O
O
F
Cl
Cl
1.00 0.97 1.98 0.99 0.98 2.05 3.03
f1 (ppm)
40000 35000 30000 25000 20000 15000 10000 5000 0

A-a-14 - $^{13}C\{^1H\}$ NMR ($CDCl_3$, 101 MHz)

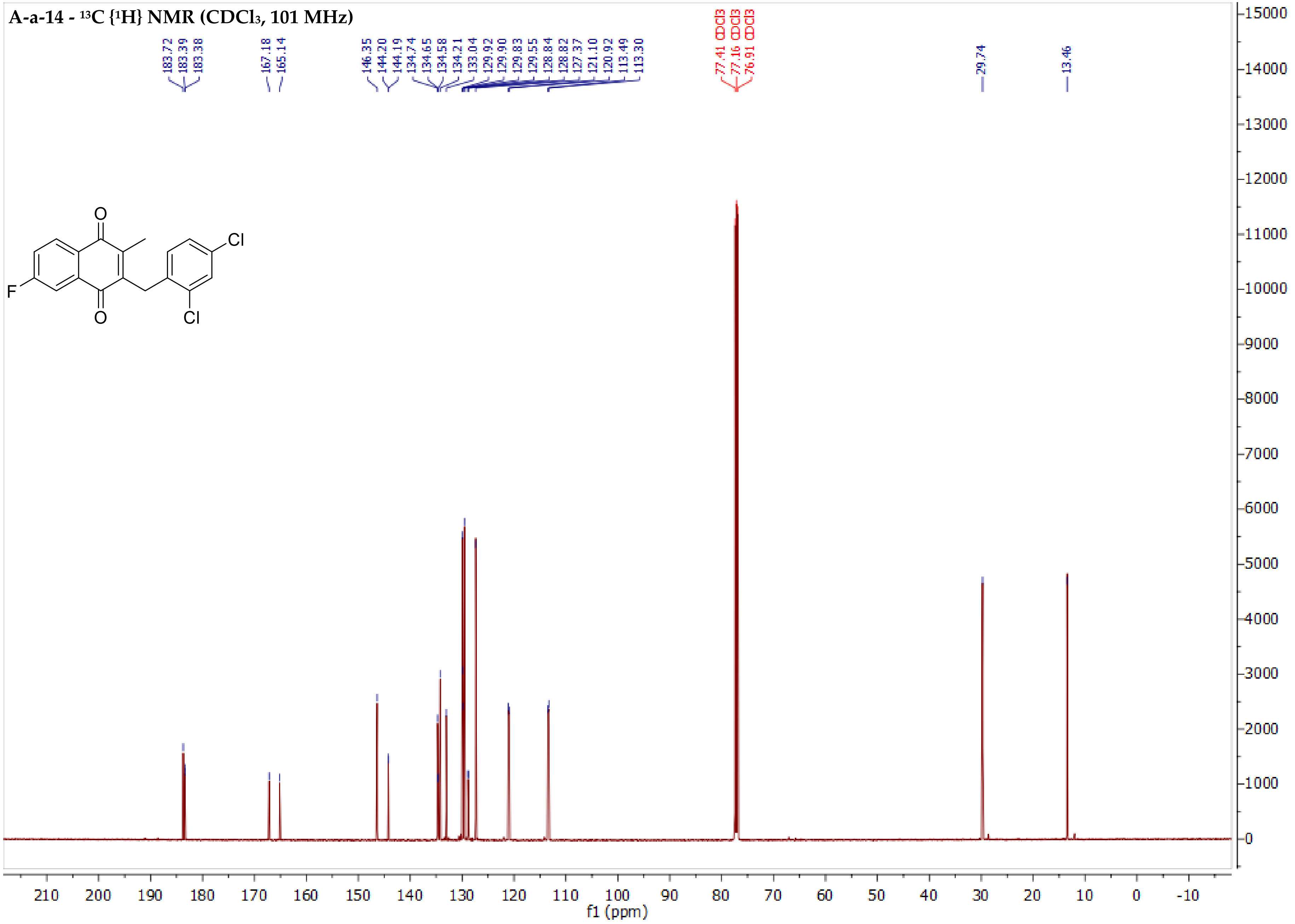

A-a-14 - ^{19}F NMR ($CDCl_3$, 377 MHz)

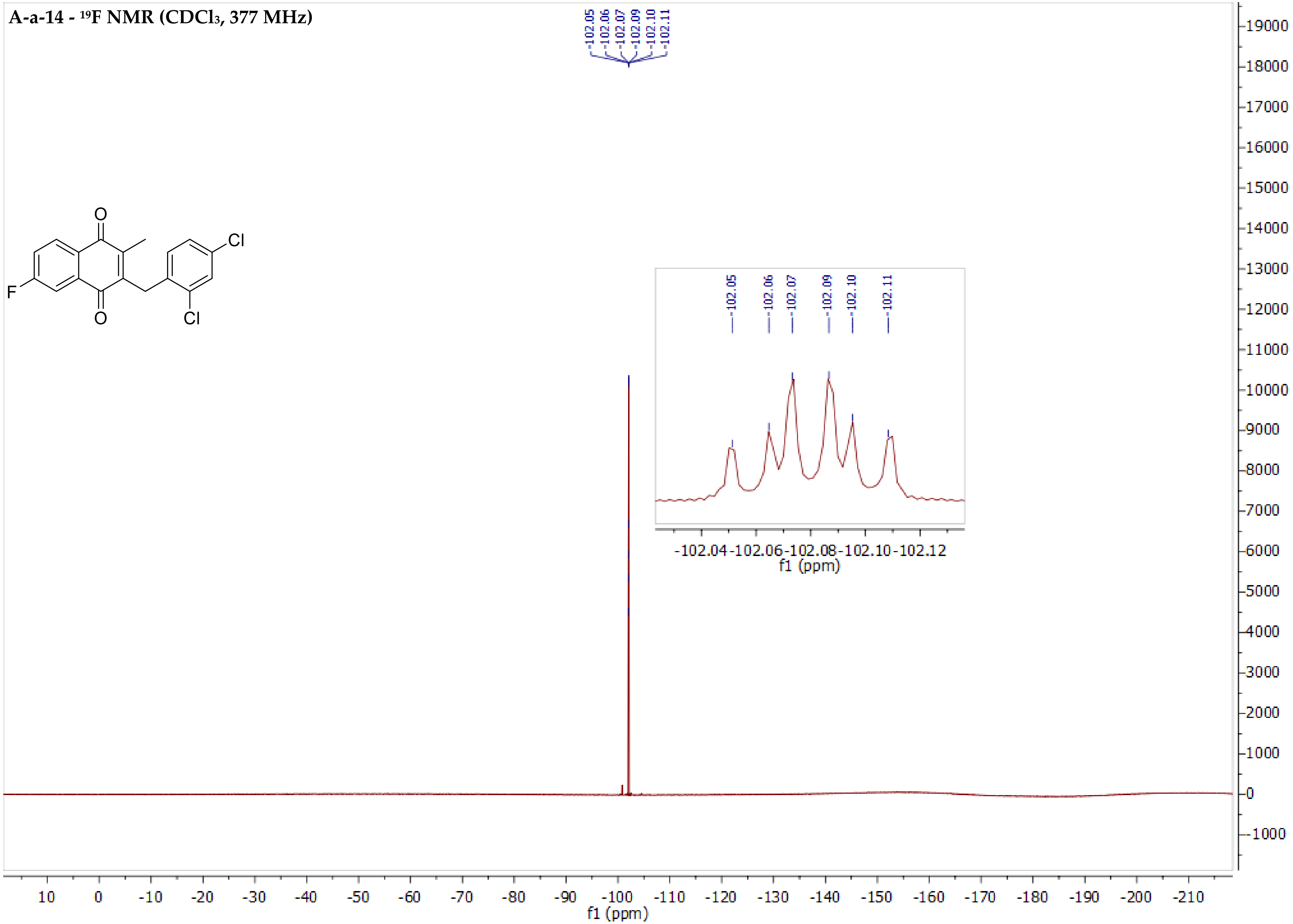

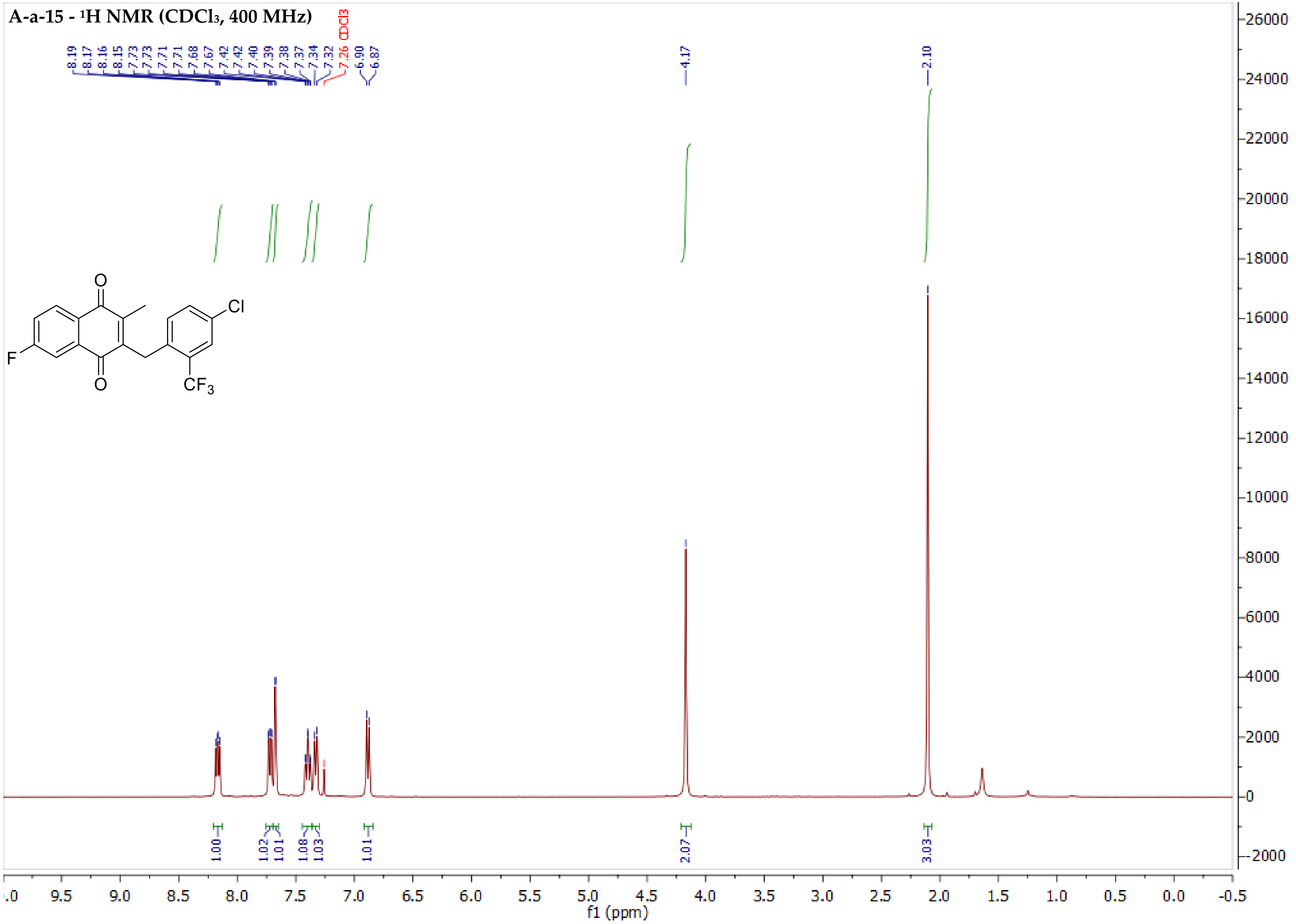
A-a-15 - 1H NMR (CDCl3, 400 MHz)
8.19 8.17 8.16 8.15 7.73 7.73 7.71 7.71 7.68 7.67 7.42 7.42 7.40 7.39 7.38 7.37 7.34 7.32 7.26 CDCl3 6.90 6.87
4.17
2.10
O
O
F
Cl
CF3
1.00 1.02 1.01 1.08 1.03 1.01 2.07 3.03
f1 (ppm)

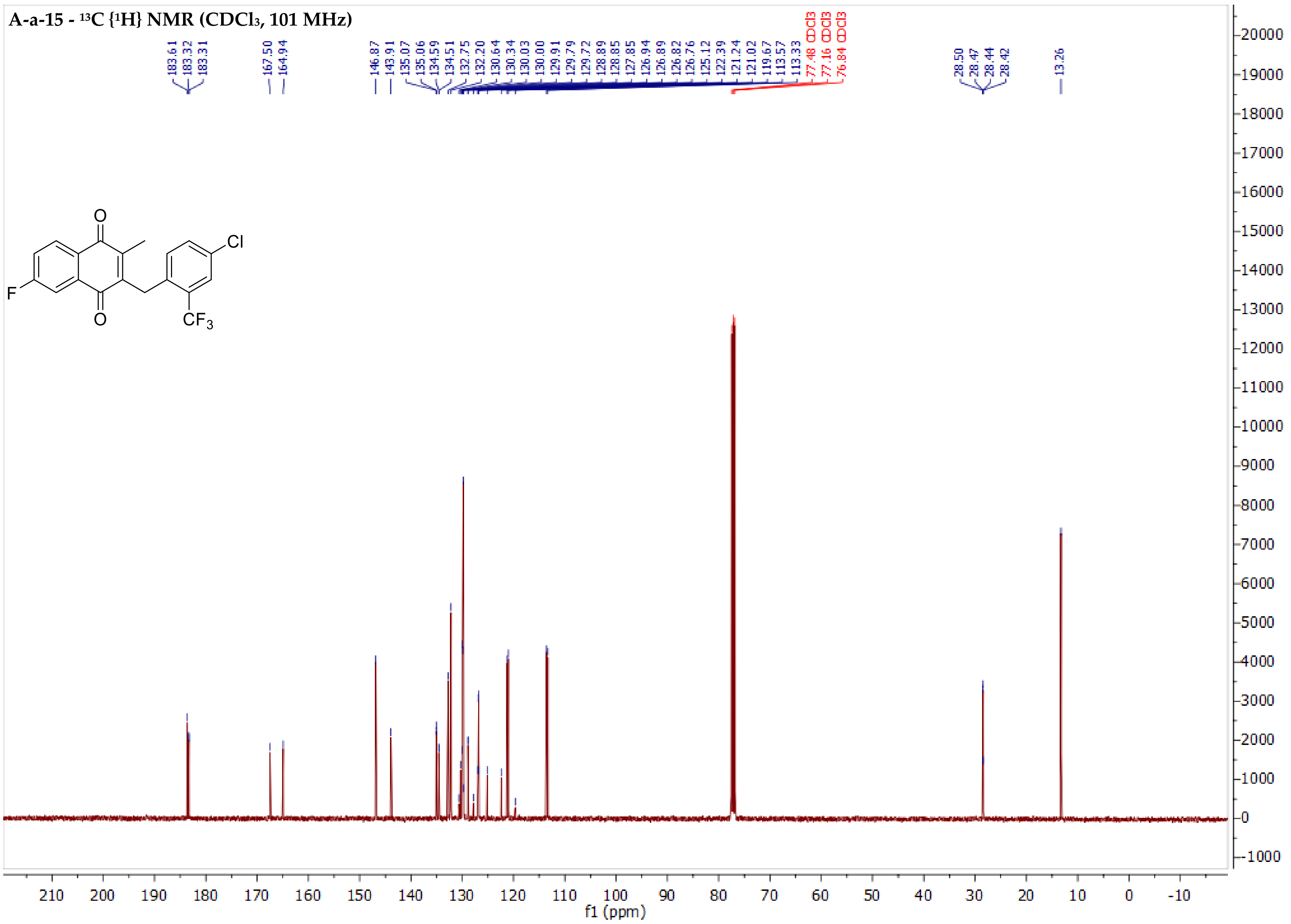
A-a-15 - 13C {1H} NMR (CDCl3, 101 MHz)
183.61
183.32
183.31
167.50
164.94
146.87
143.91
135.07
135.06
134.59
134.51
132.75
132.20
130.64
130.34
130.03
130.00
129.91
129.79
129.72
128.89
128.85
127.85
126.94
126.89
126.82
126.76
125.12
122.39
121.24
121.02
119.67
113.57
113.33
77.48 CDCl3
77.16 CDCl3
76.84 CDCl3
28.50
28.47
28.44
28.42
13.26
O
Cl
F
O
CF3
f1 (ppm)

A-a-15 - ^{19}F NMR ($CDCl_3$, 377 MHz)

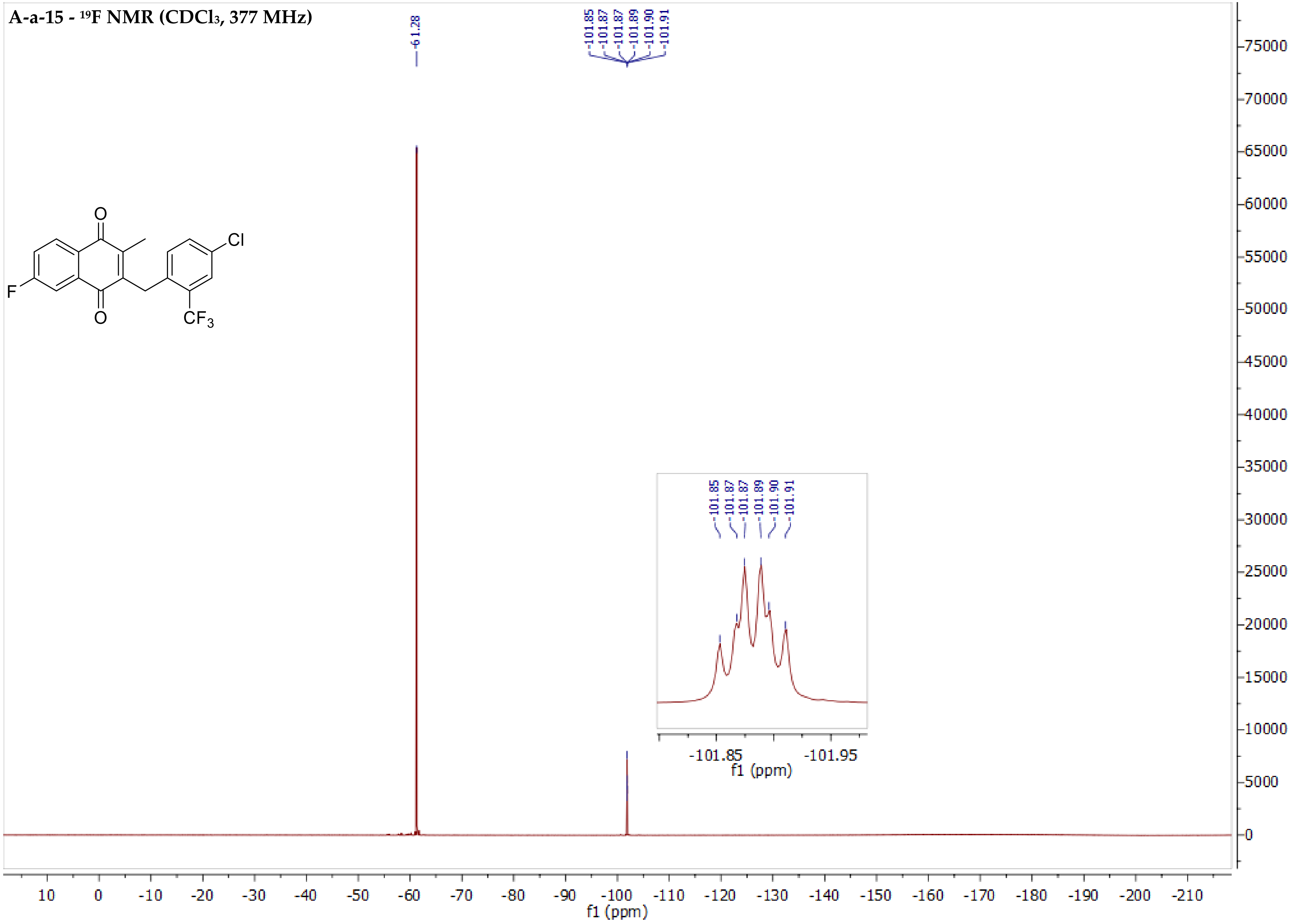

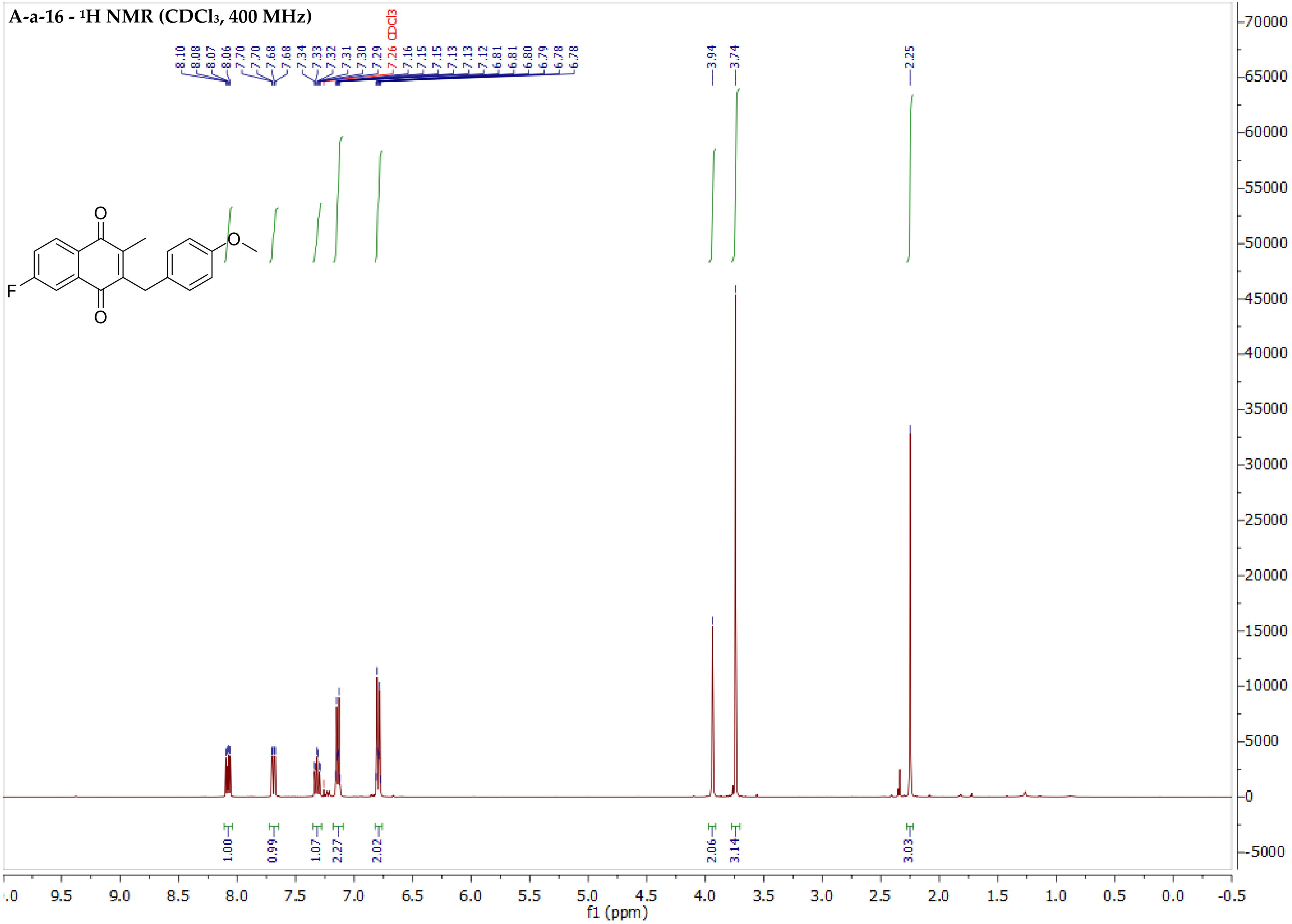
A-a-16 - ¹H NMR (CDCl₃, 400 MHz)
8.10 8.08 8.07 8.06 7.70 7.70 7.68 7.68 7.34 7.33 7.32 7.31 7.30 7.29 7.26 CDCl3 7.16 7.15 7.15 7.13 7.13 7.12 6.81 6.81 6.80 6.79 6.78 6.78
3.94
3.74
2.25
1.00 0.99 1.07 2.27 2.02 2.06 3.14 3.03
f1 (ppm)
F
O
O
O

A-a-16 - $^{13}C\{^1H\}$ NMR ($CDCl_3$, 101 MHz)

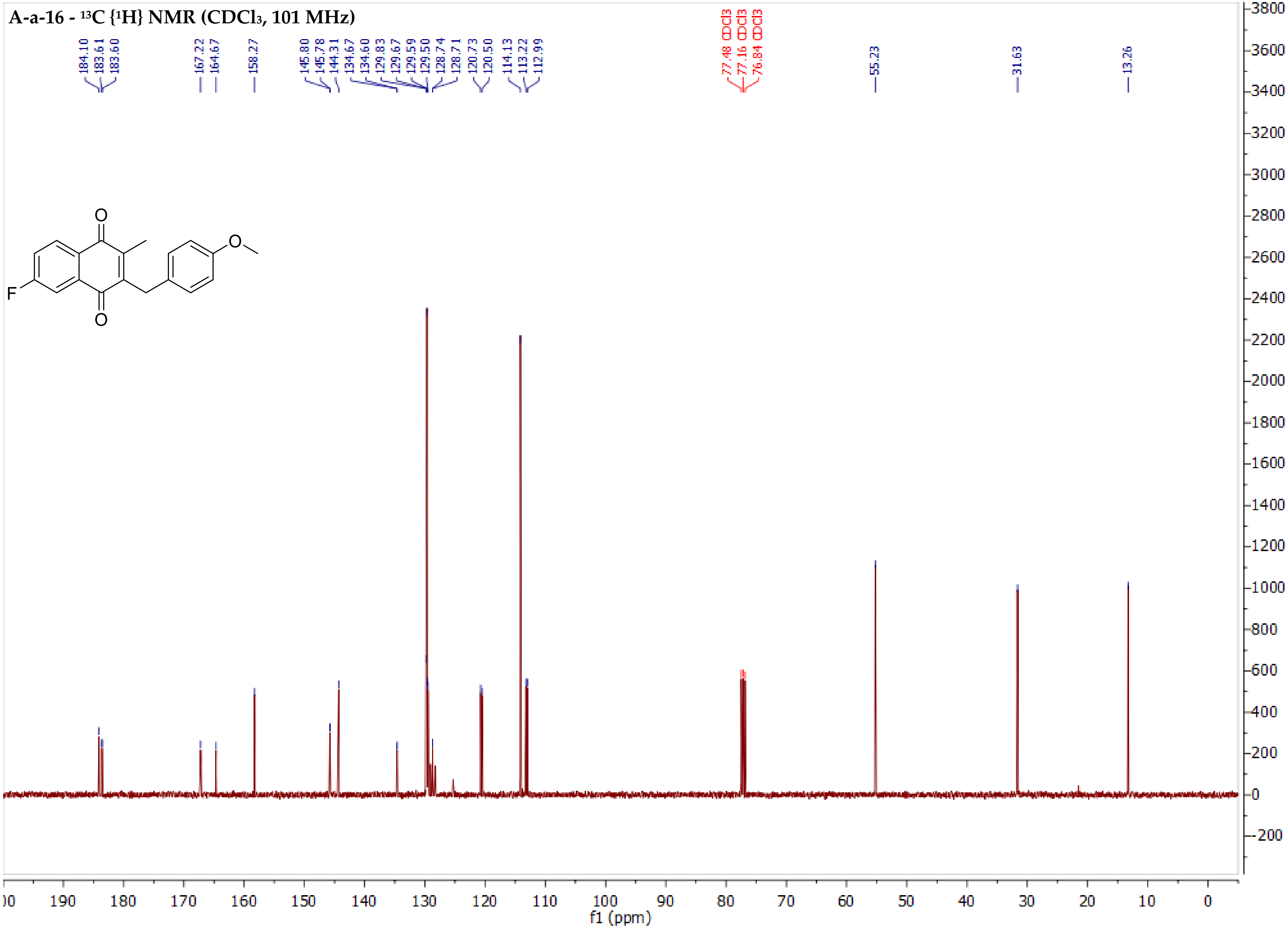

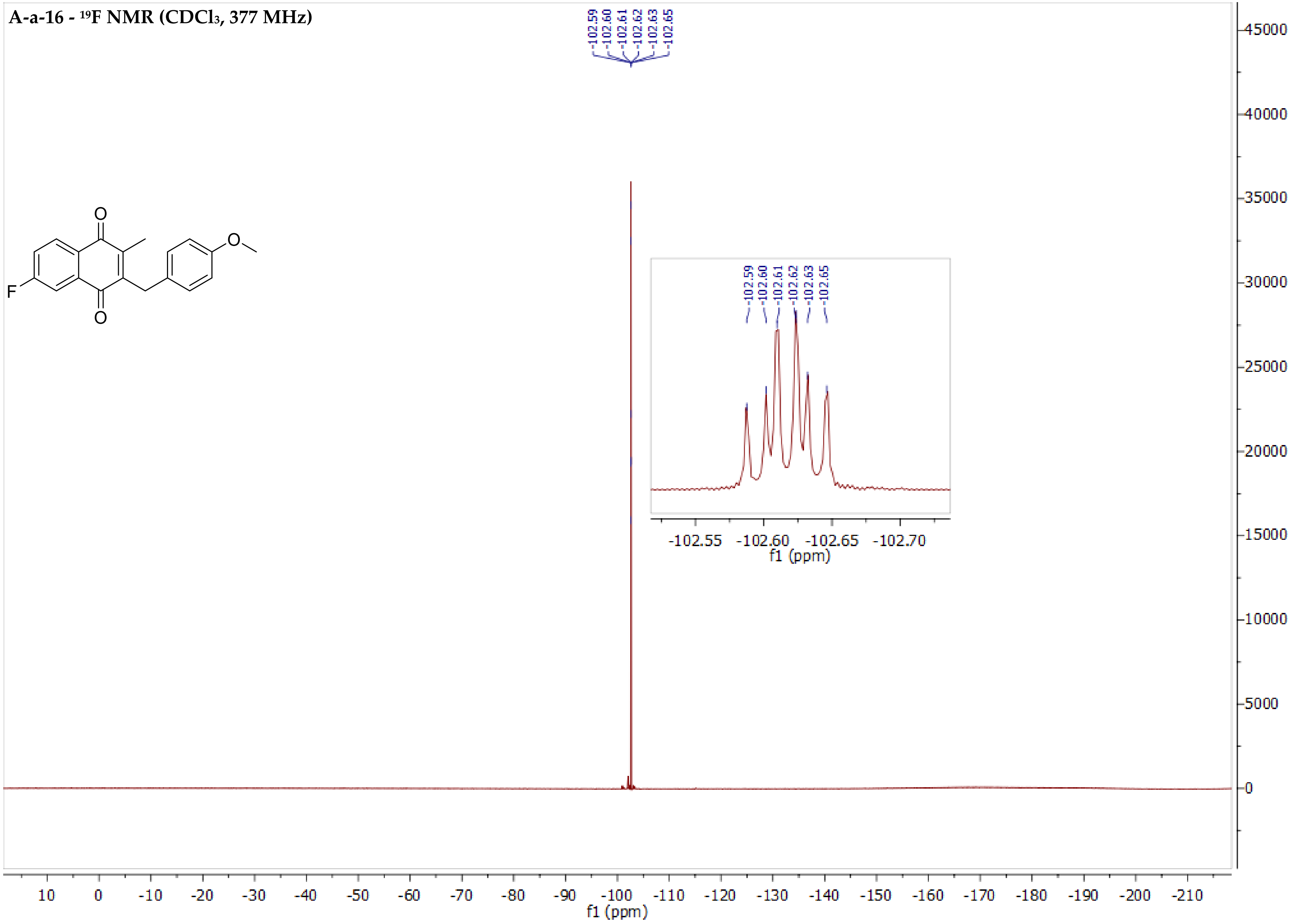

A-a-16 - ¹⁹F NMR (CDCl₃, 377 MHz)
-102.59
-102.60
-102.61
-102.62
-102.63
-102.65
O
F
O
O
-102.55 -102.60 -102.65 -102.70
f1 (ppm)
45000
40000
35000
30000
25000
20000
15000
10000
5000
0
10 0 -10 -20 -30 -40 -50 -60 -70 -80 -90 -100 -110 -120 -130 -140 -150 -160 -170 -180 -190 -200 -210
f1 (ppm)

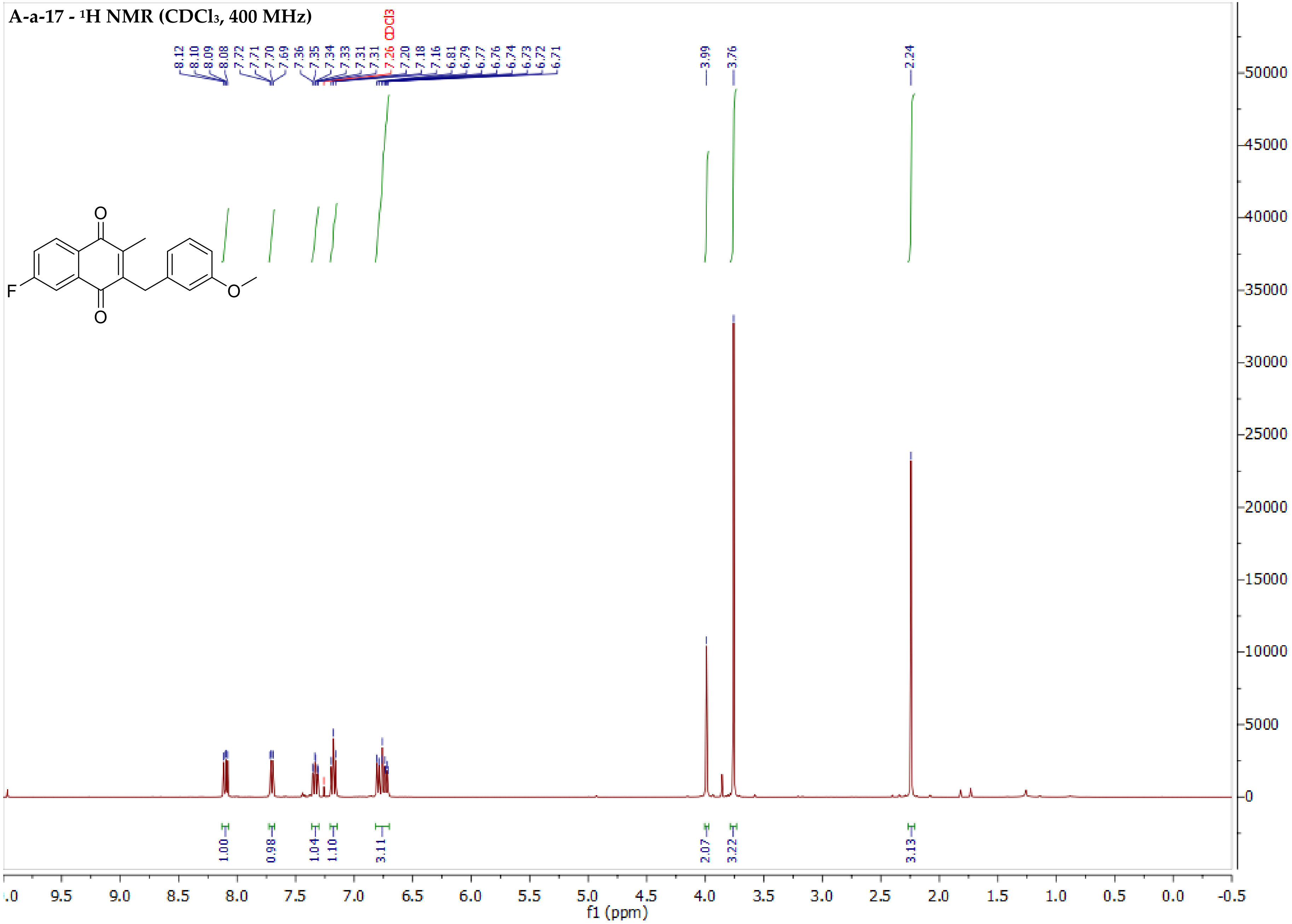
A-a-17 - ¹H NMR (CDCl₃, 400 MHz)
8.12 8.10 8.09 8.08 7.72 7.71 7.70 7.69 7.36 7.35 7.34 7.33 7.31 7.31 7.26 7.20 7.18 7.16 6.81 6.79 6.77 6.76 6.74 6.73 6.72 6.71
CDCl3
3.99 3.76 2.24
1.00 0.98 1.04 1.10 3.11 2.07 3.22 3.13
f1 (ppm)

A-a-17 - $^{13}C\{^{1}H\}$ NMR ($CDCl_3$, 101 MHz)

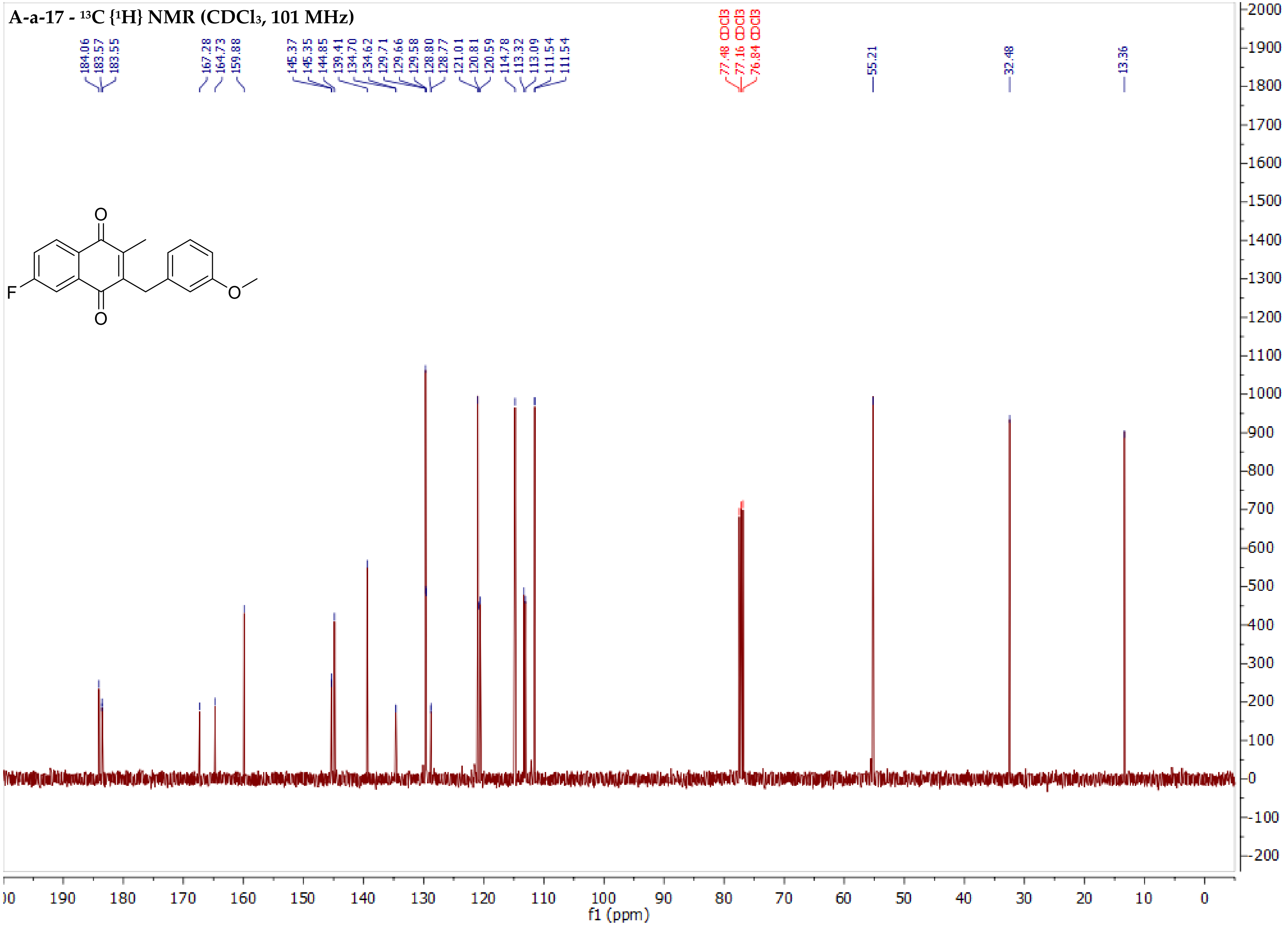

A-a-17 - ^{19}F NMR ($CDCl_3$, 377 MHz)

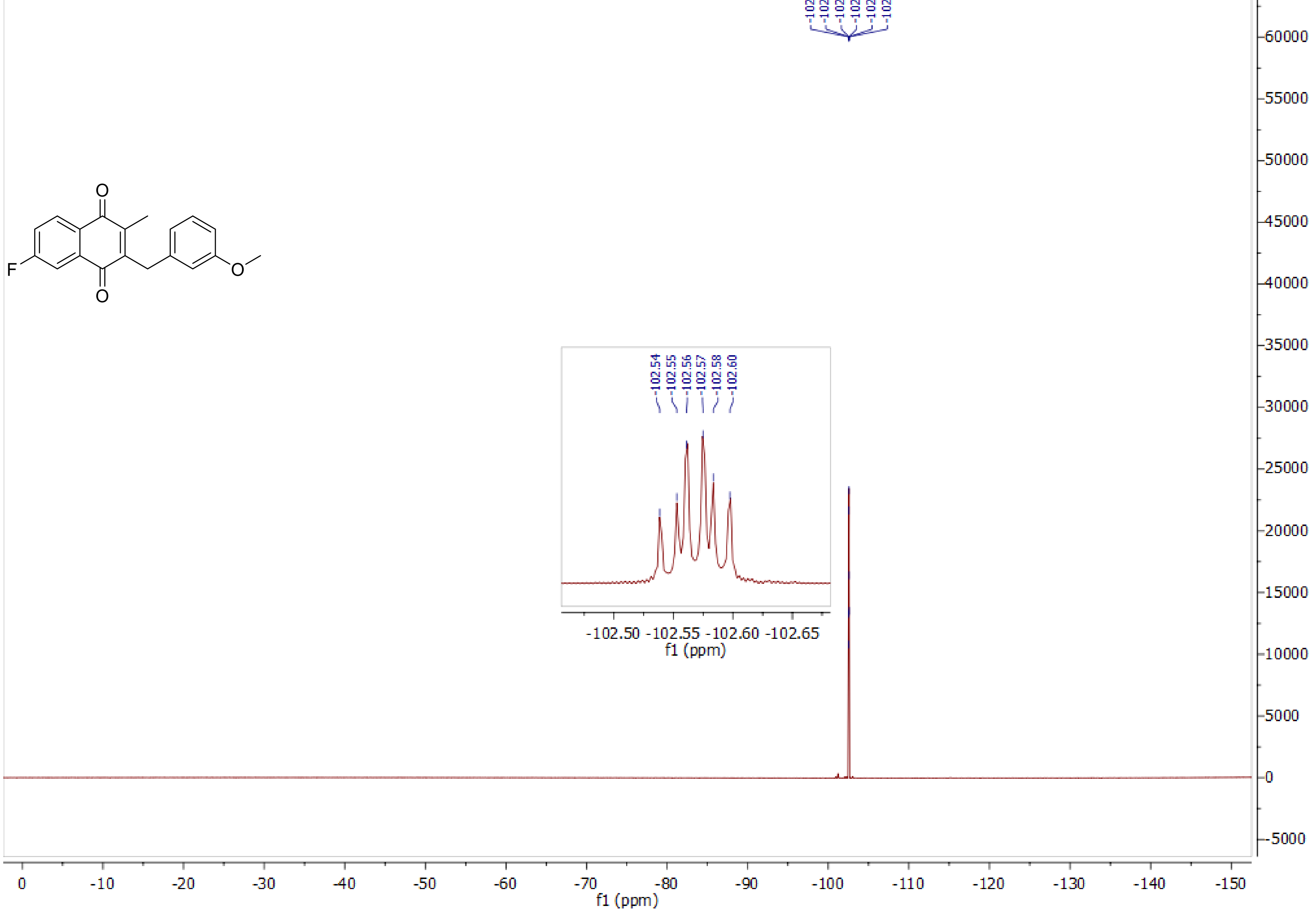

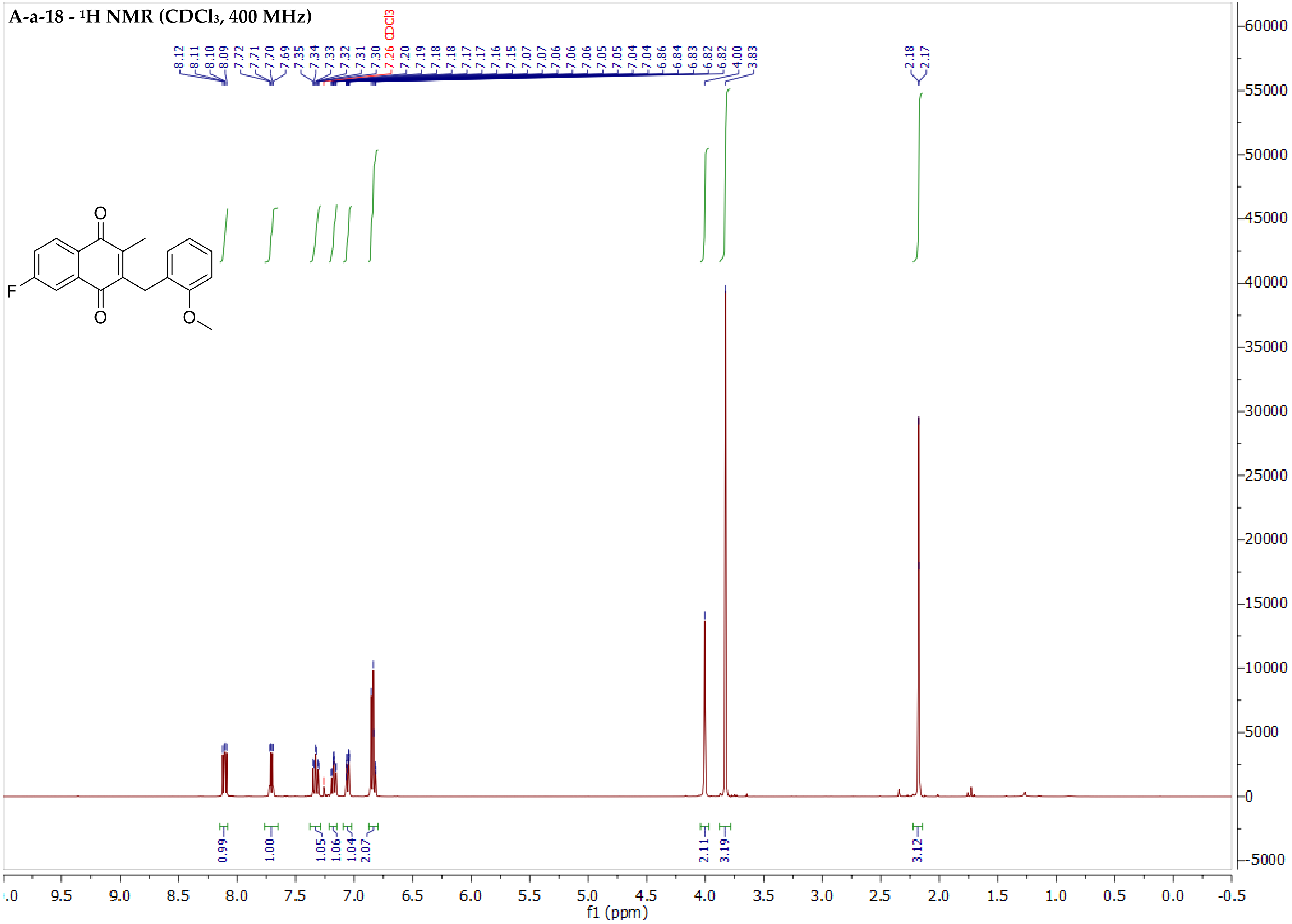
A-a-18 - 1H NMR (CDCl3, 400 MHz)
F
O
O
O
f1 (ppm)

A-a-18 - $^{13}C\{^1H\}$ NMR ($CDCl_3$, 101 MHz)

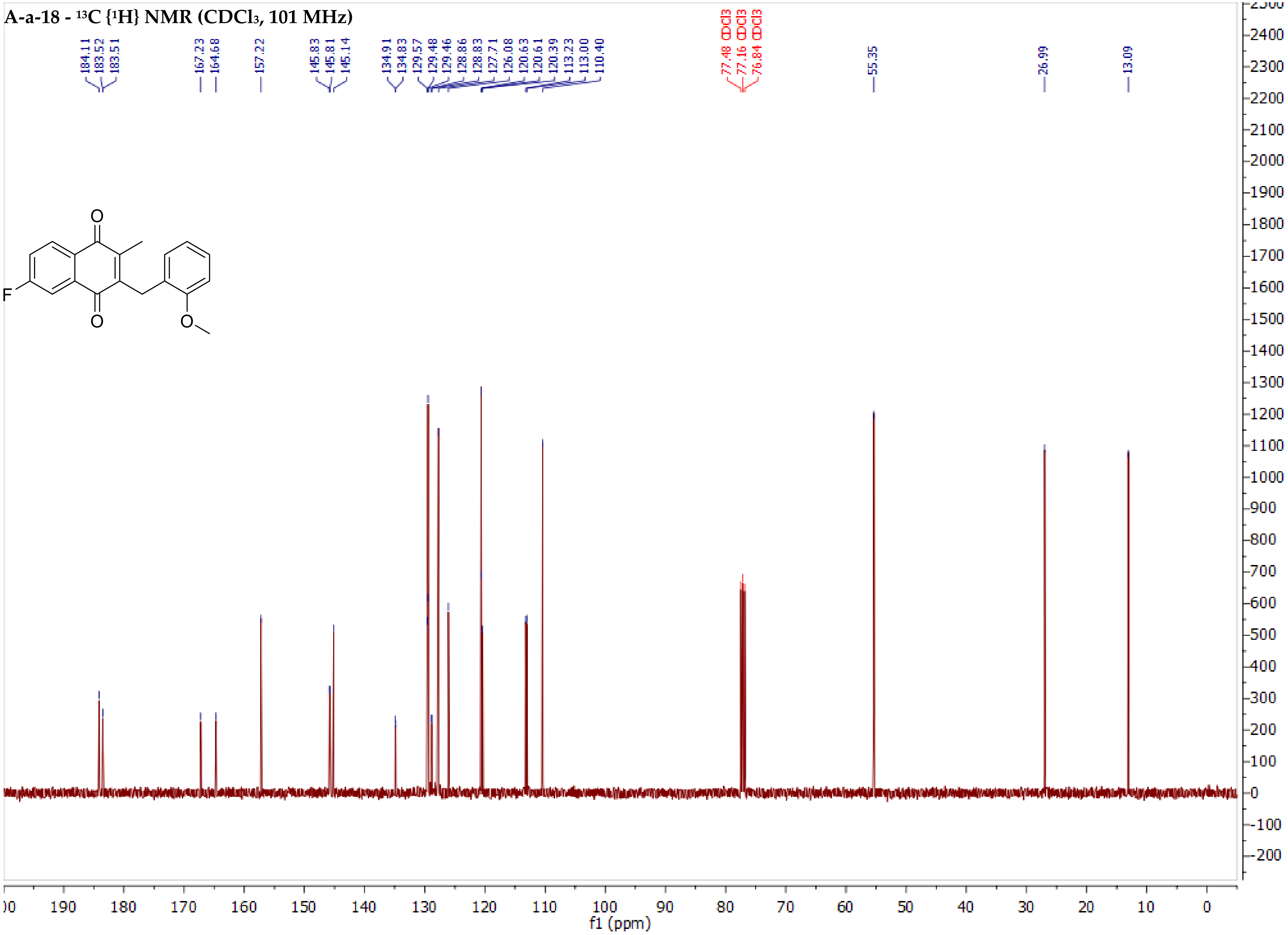

A-a-18 - ^{19}F NMR (CDCl$_3$, 377 MHz)

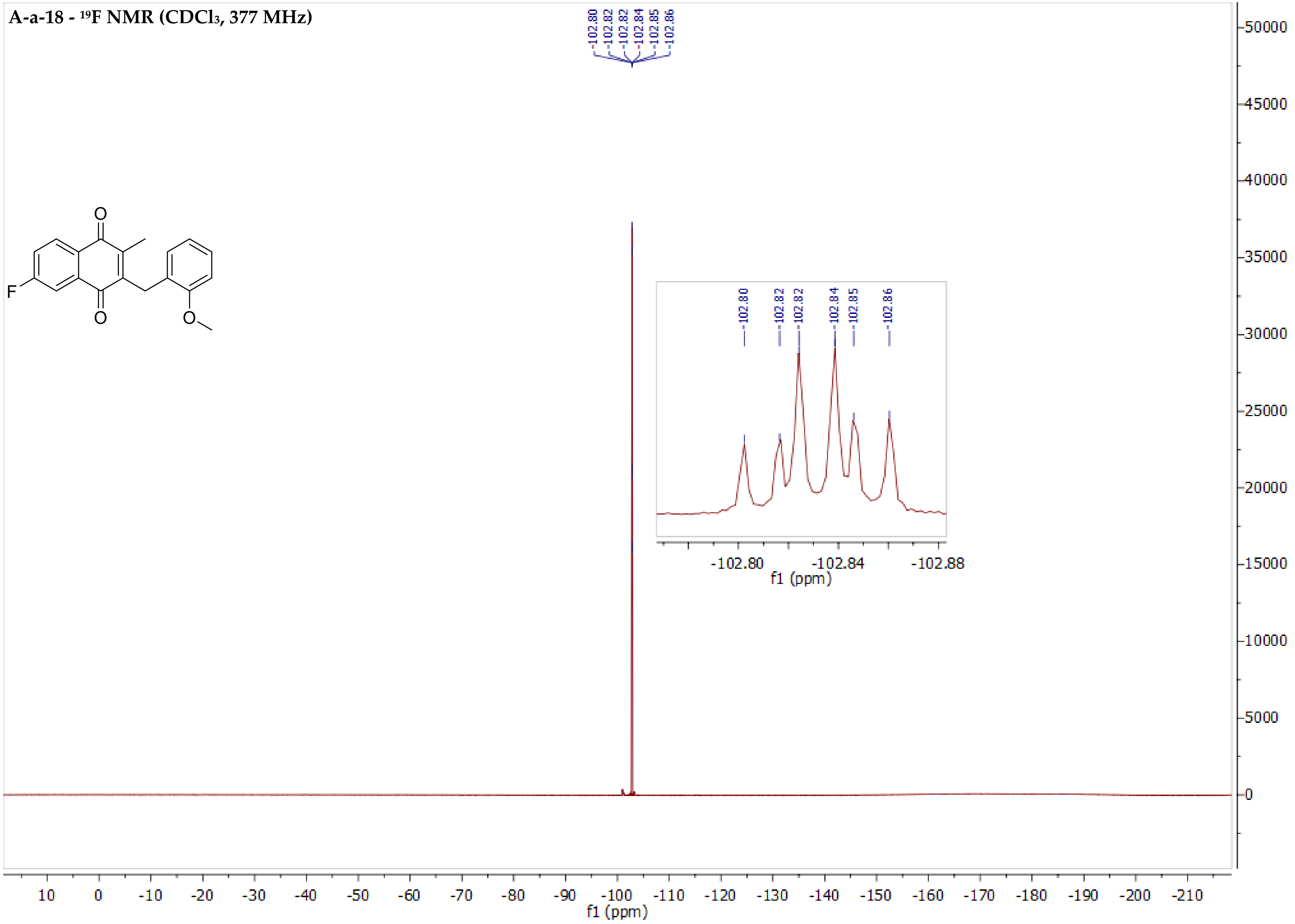

A-a-19 - ^{1}H NMR ($CDCl_3$, 400 MHz)

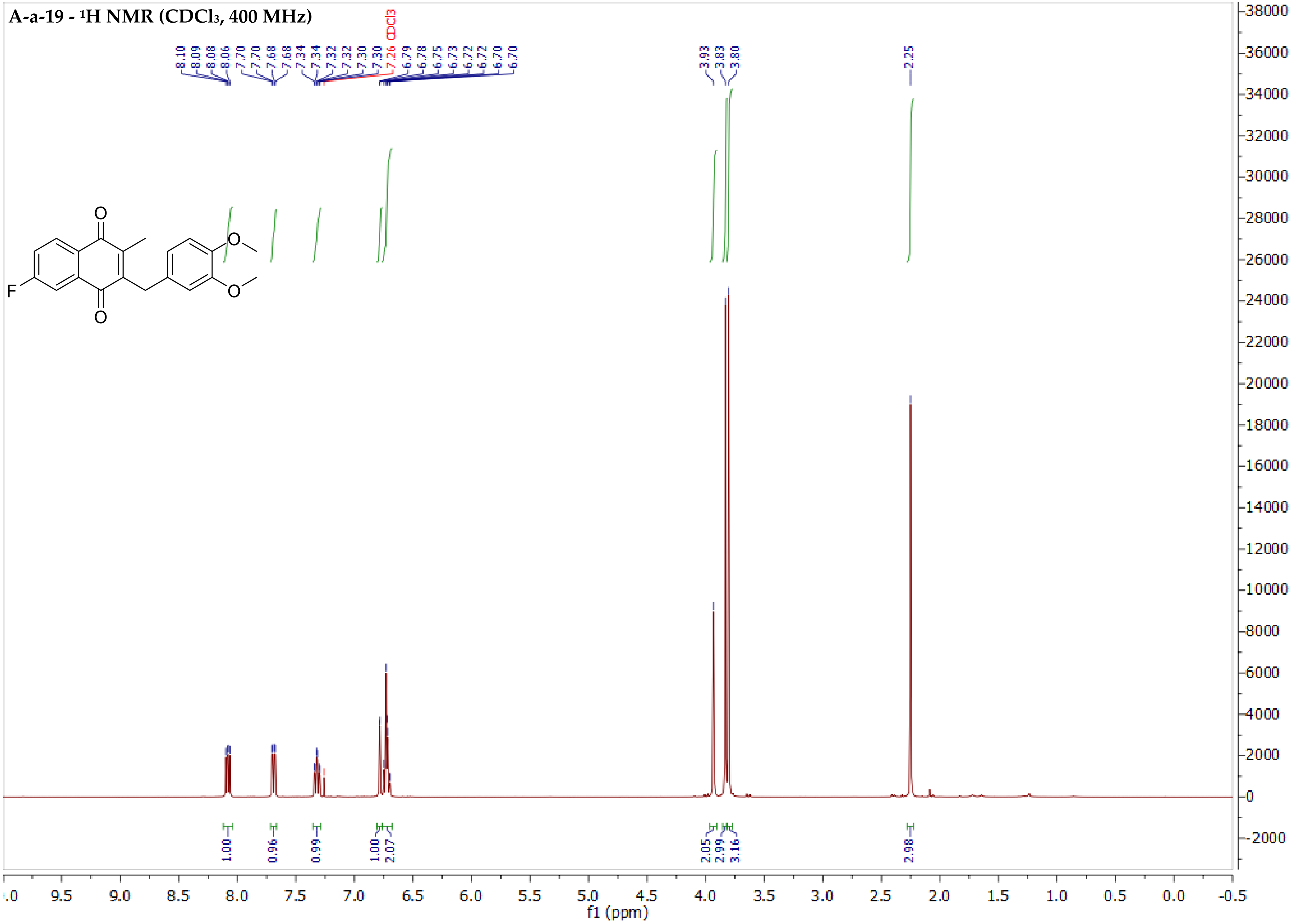

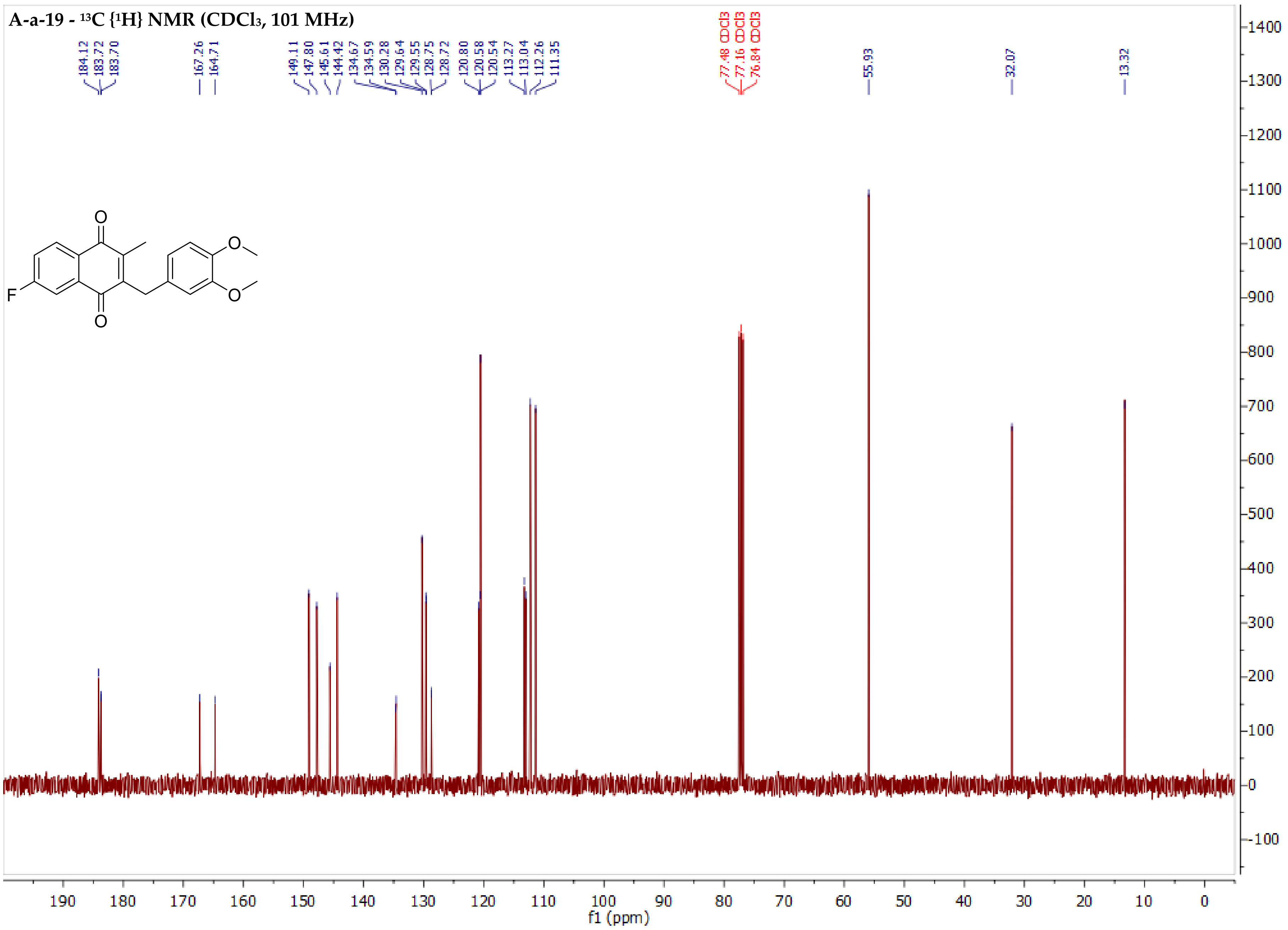
A-a-19 - ^{13}C {^{1}H} NMR ($CDCl_3$, 101 MHz)
184.12
183.72
183.70
167.26
164.71
149.11
147.80
145.61
144.42
134.67
134.59
130.28
129.64
129.55
128.75
128.72
120.80
120.58
120.54
113.27
113.04
112.26
111.35
77.48 CDCl3
77.16 CDCl3
76.84 CDCl3
55.93
32.07
13.32
O
O
O
O
F
f1 (ppm)

A-a-19 - ^{19}F NMR ($CDCl_3$, 377 MHz)

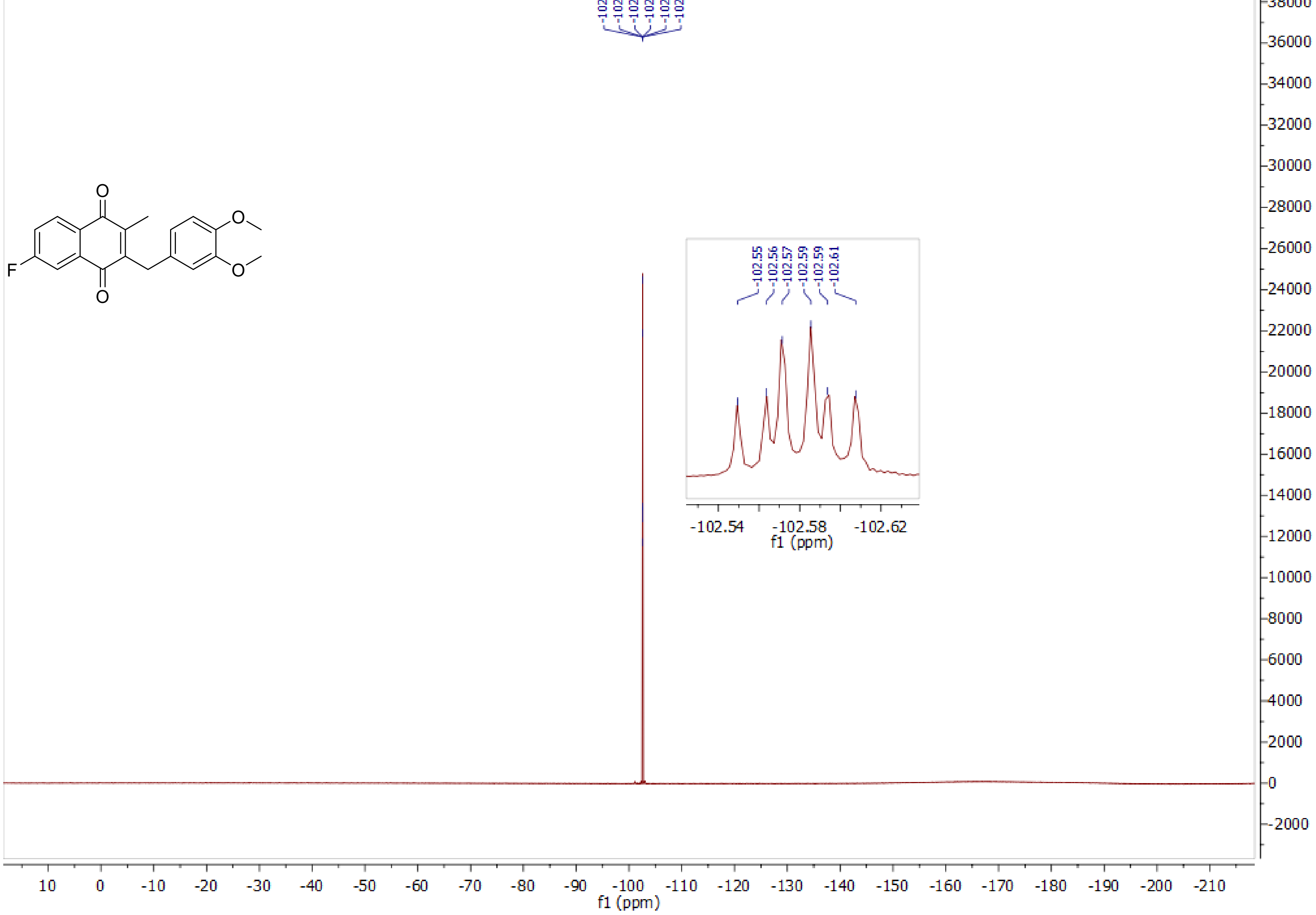

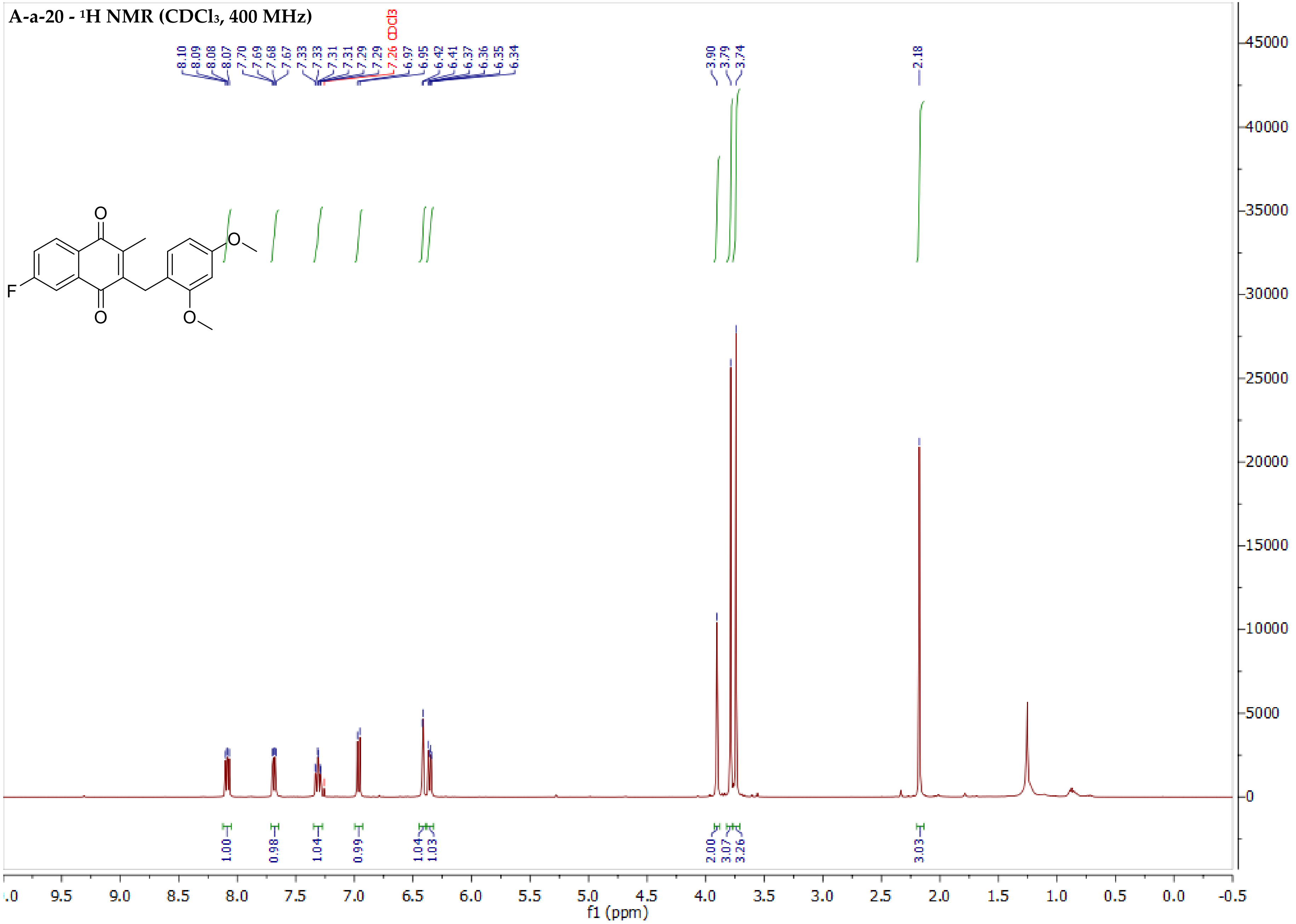
A-a-20 - ¹H NMR (CDCl₃, 400 MHz)
8.10 8.09 8.08 8.07 7.70 7.69 7.68 7.67 7.33 7.33 7.31 7.31 7.29 7.29 7.26 CDCl3 6.97 6.95 6.42 6.41 6.37 6.36 6.35 6.34
3.90 3.79 3.74
2.18
O
O
O
O
F
1.00 0.98 1.04 0.99 1.04 1.03 2.00 3.07 3.26 3.03
f1 (ppm)

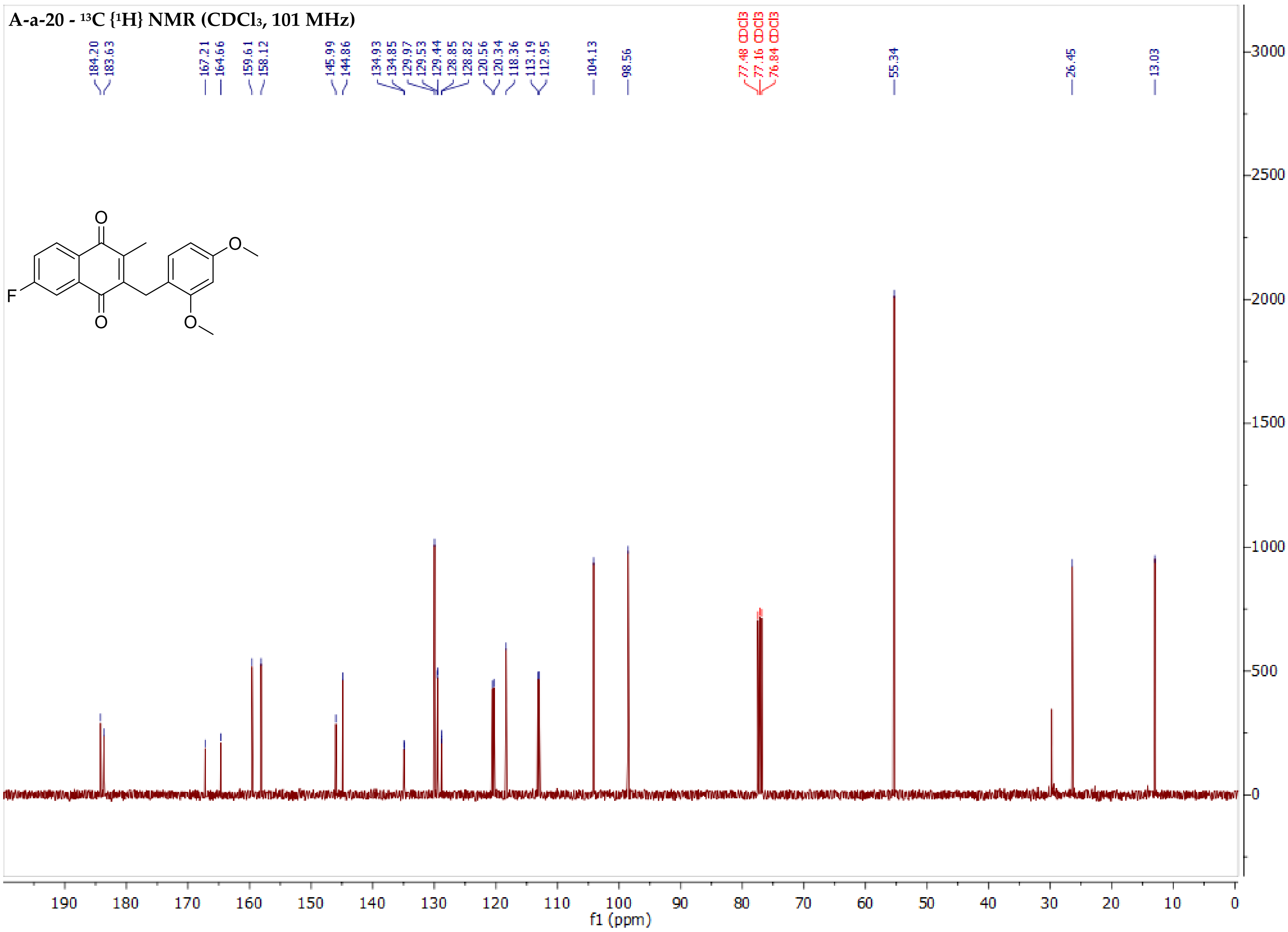

A-a-20 - ^{13}C {^{1}H} NMR ($CDCl_3$, 101 MHz)
184.20
183.63
167.21
164.66
159.61
158.12
145.99
144.86
134.93
134.85
129.97
129.53
129.44
128.85
128.82
120.56
120.34
118.36
113.19
112.95
104.13
98.56
77.48 CDCl3
77.16 CDCl3
76.84 CDCl3
55.34
26.45
13.03
F
O
f1 (ppm)

A-a-20 - ^{19}F NMR ($CDCl_3$, 377 MHz)

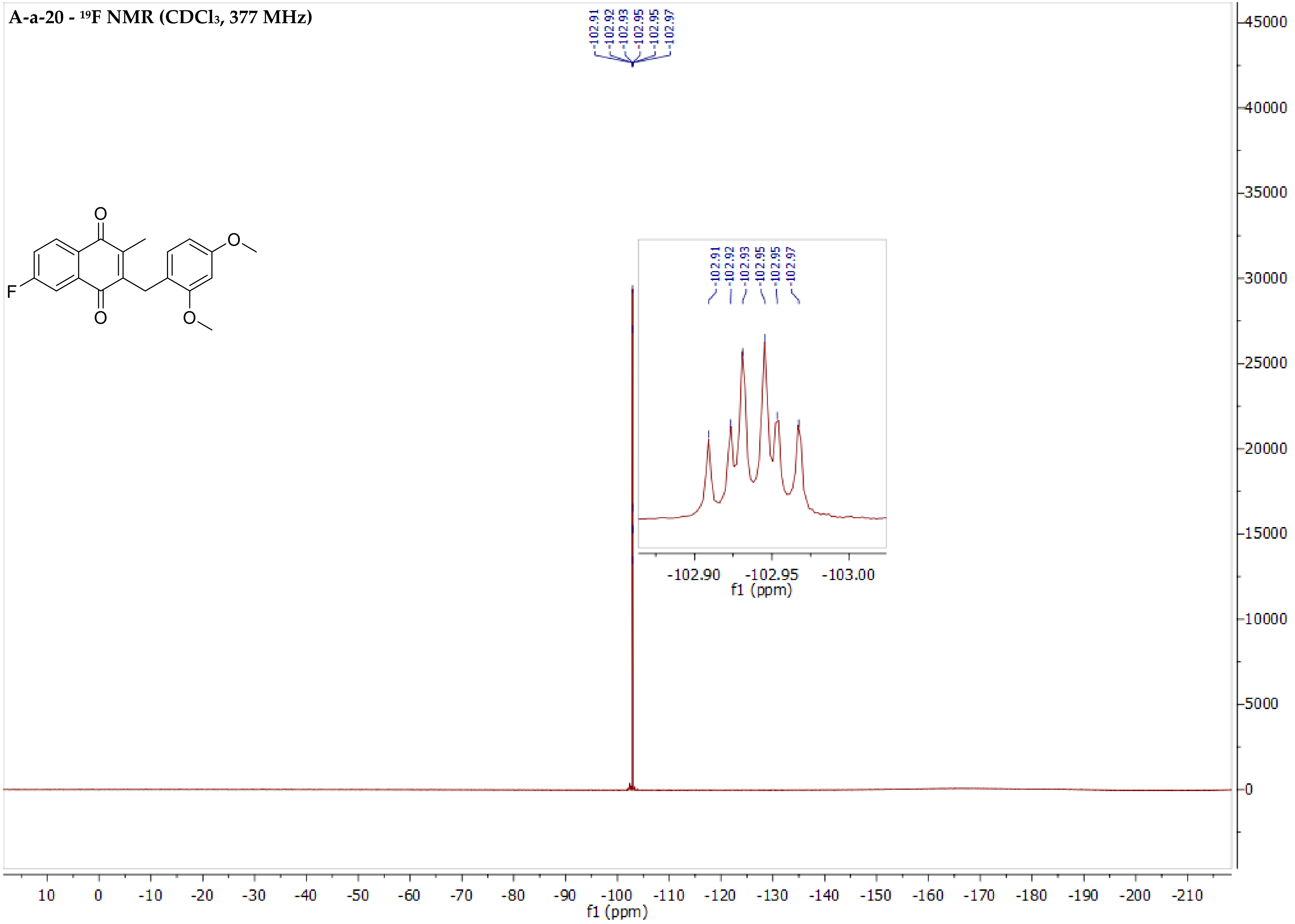

A-a-21 - ¹H NMR (CDCl₃, 400 MHz)

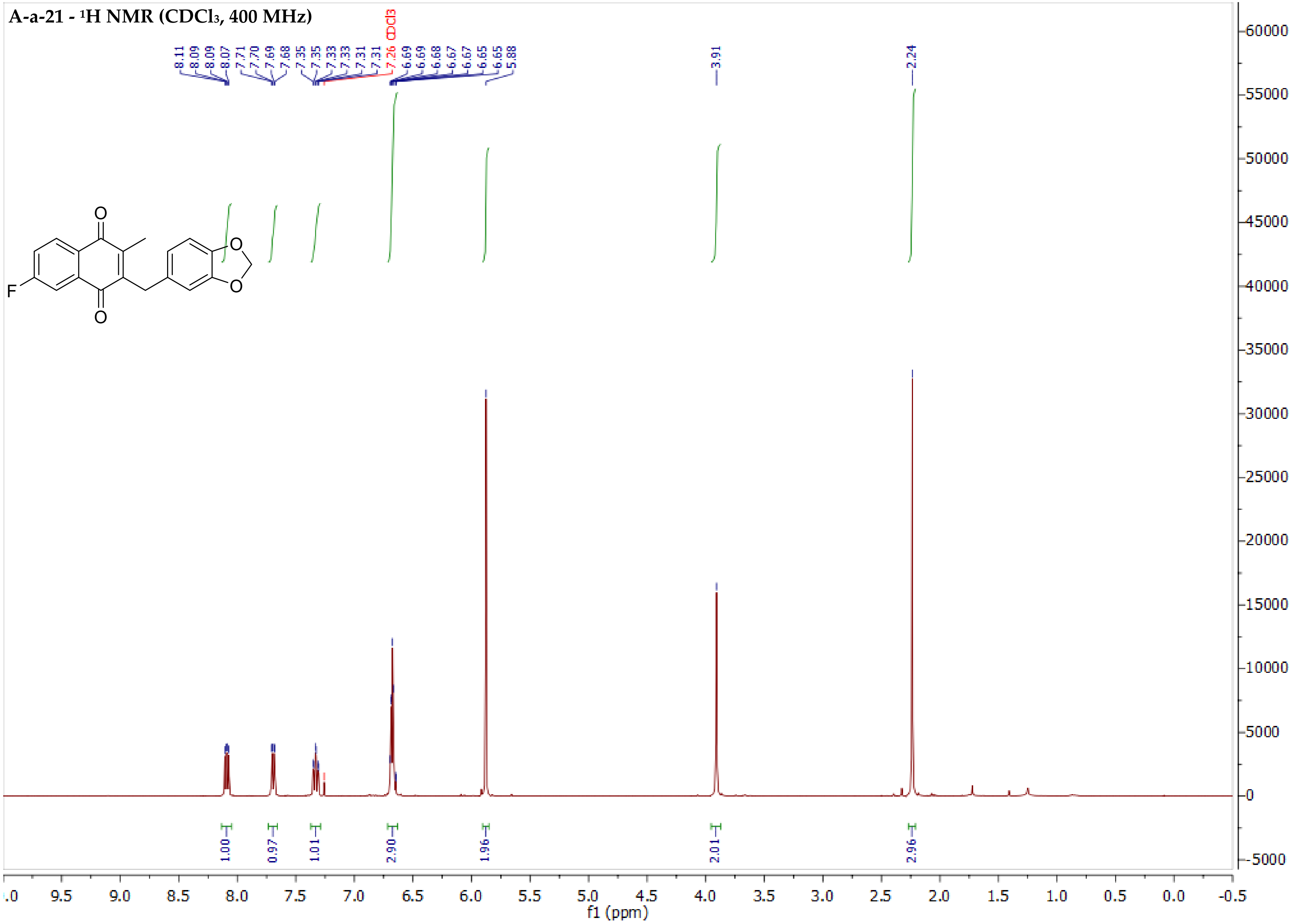

A-a-21 - ^{13}C {^{1}H} NMR (CDCl$_3$, 101 MHz)

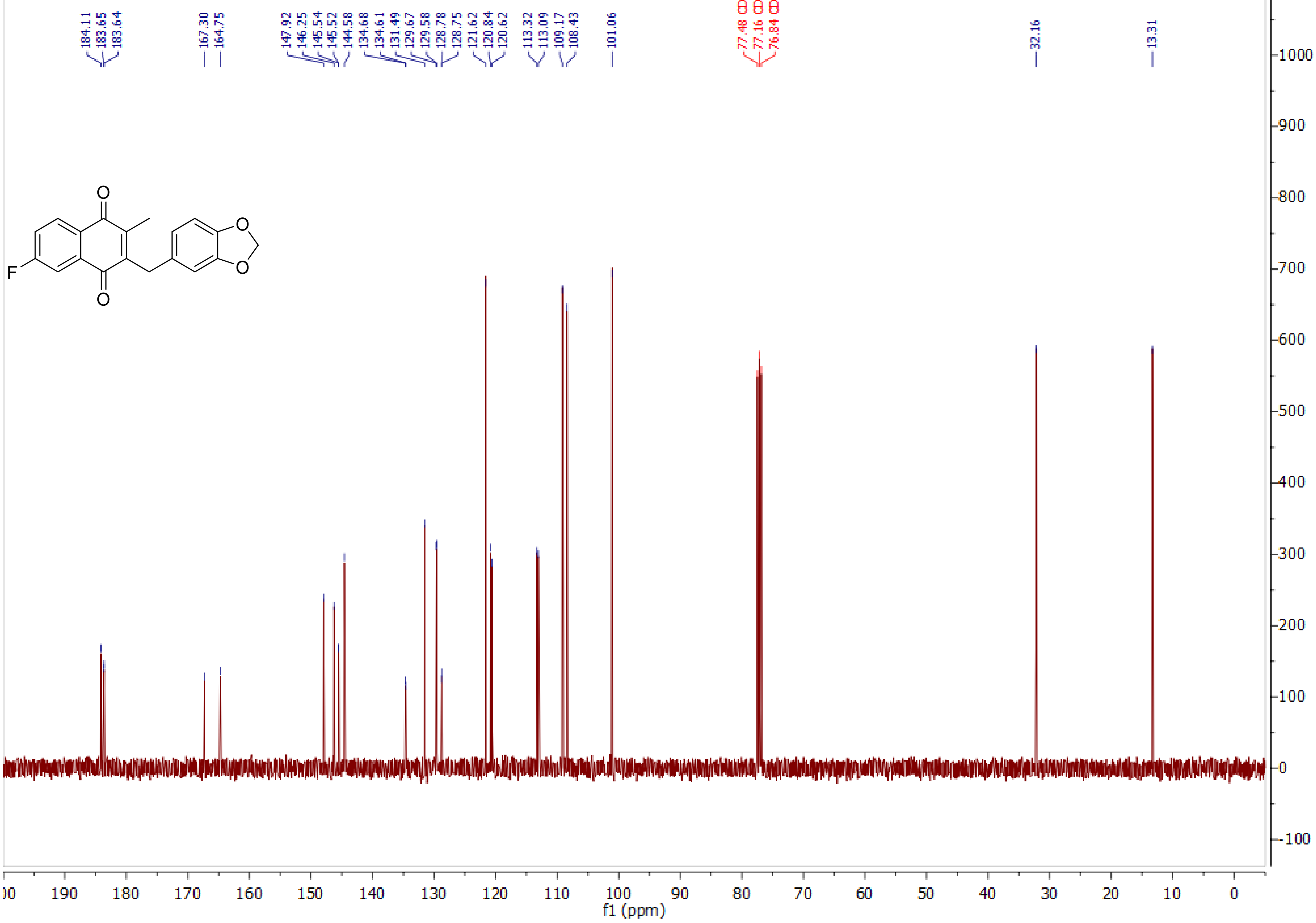

A-a-21 - ^{19}F NMR ($CDCl_3$, 377 MHz)

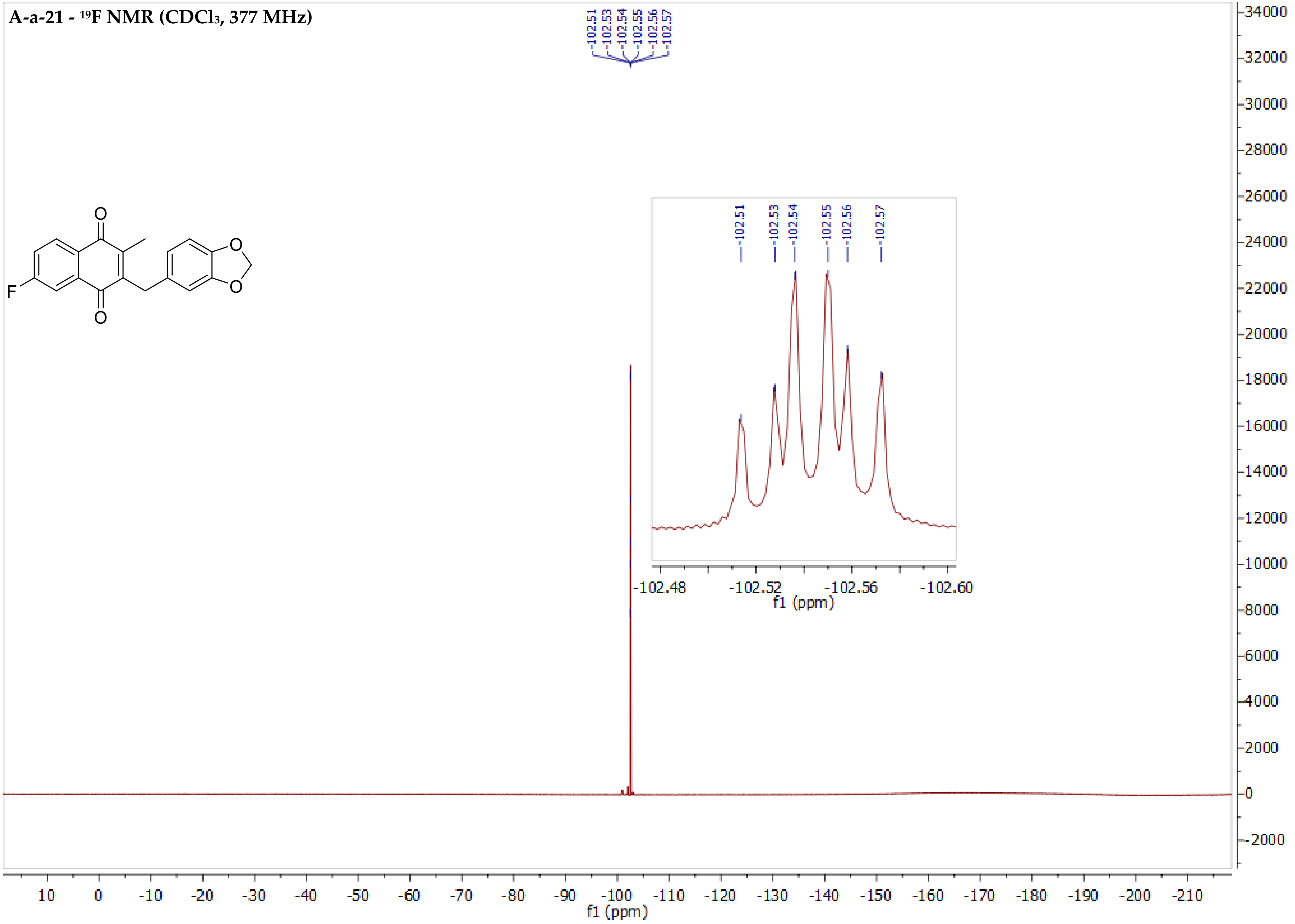

A-a-22 - ^{1}H NMR ($CDCl_3$, 400 MHz)

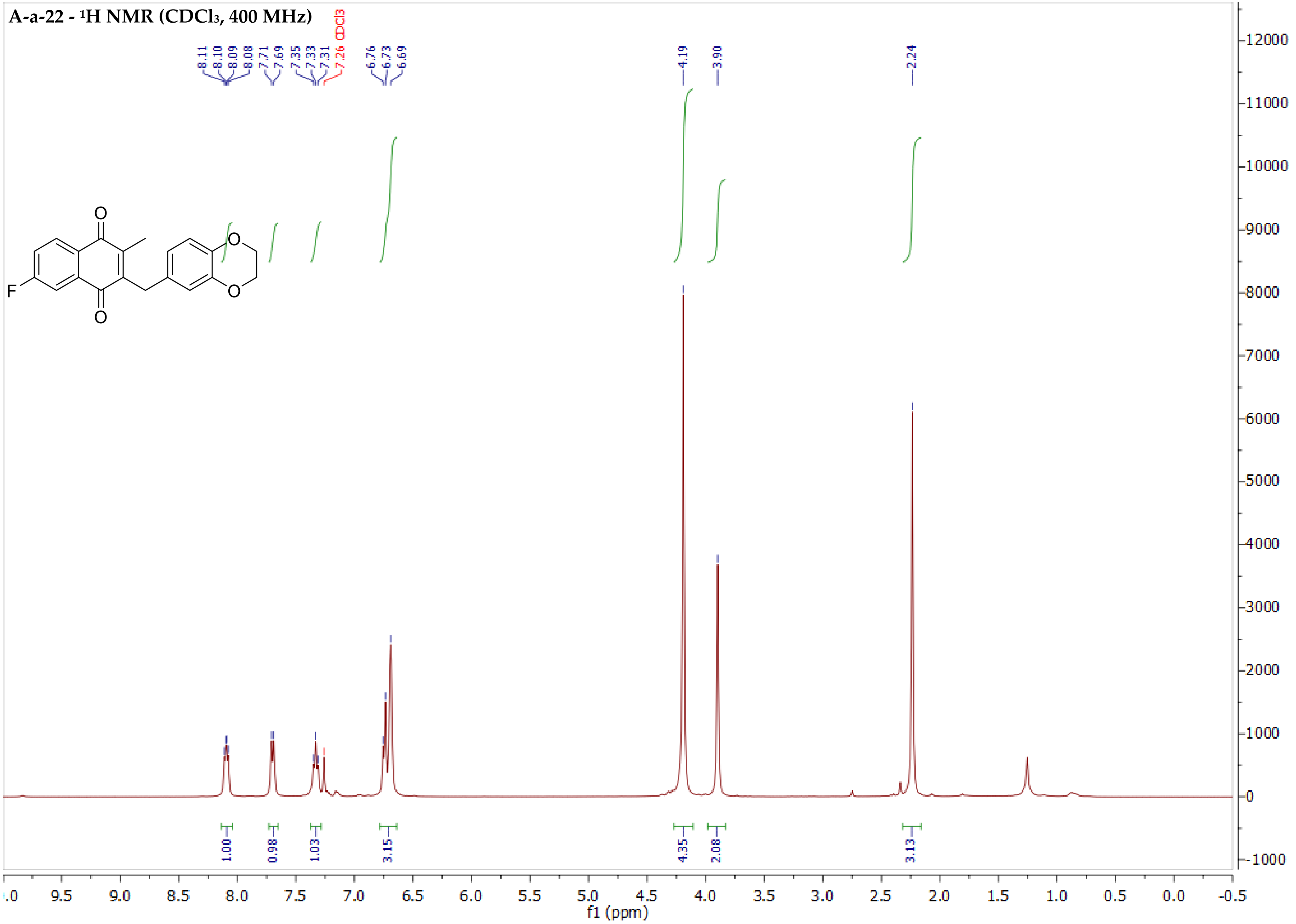

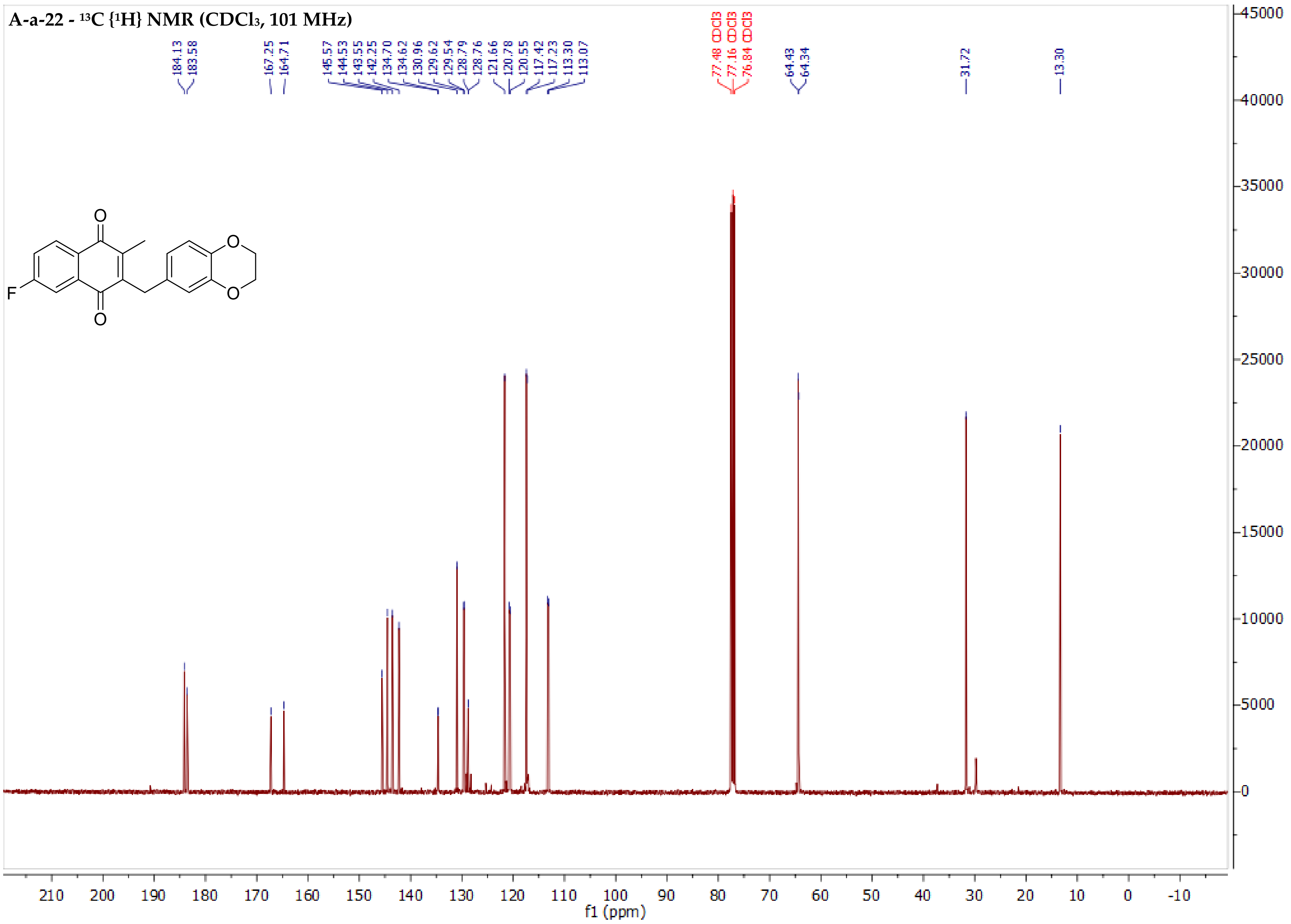

A-a-22 - 13C {1H} NMR (CDCl3, 101 MHz)
184.13
183.58
167.25
164.71
145.57
144.53
143.55
142.25
134.70
134.62
130.96
129.62
129.54
128.79
128.76
121.66
120.78
120.55
117.42
117.23
113.30
113.07
77.48 CDCl3
77.16 CDCl3
76.84 CDCl3
64.43
64.34
31.72
13.30
f1 (ppm)

A-a-22 - ^{19}F NMR ($CDCl_3$, 377 MHz)

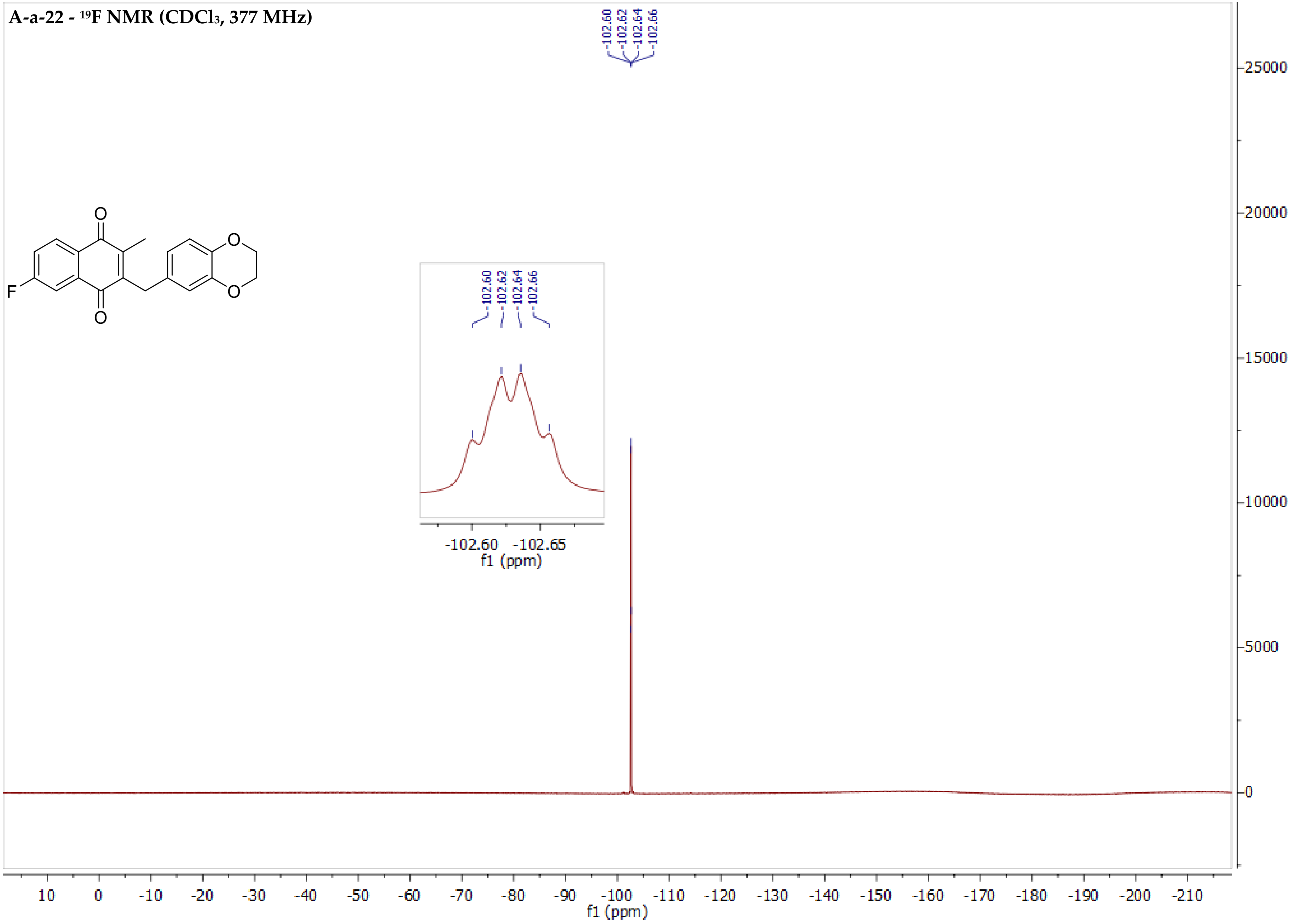

A-a-23 - ¹H NMR (CDCl₃, 400 MHz)

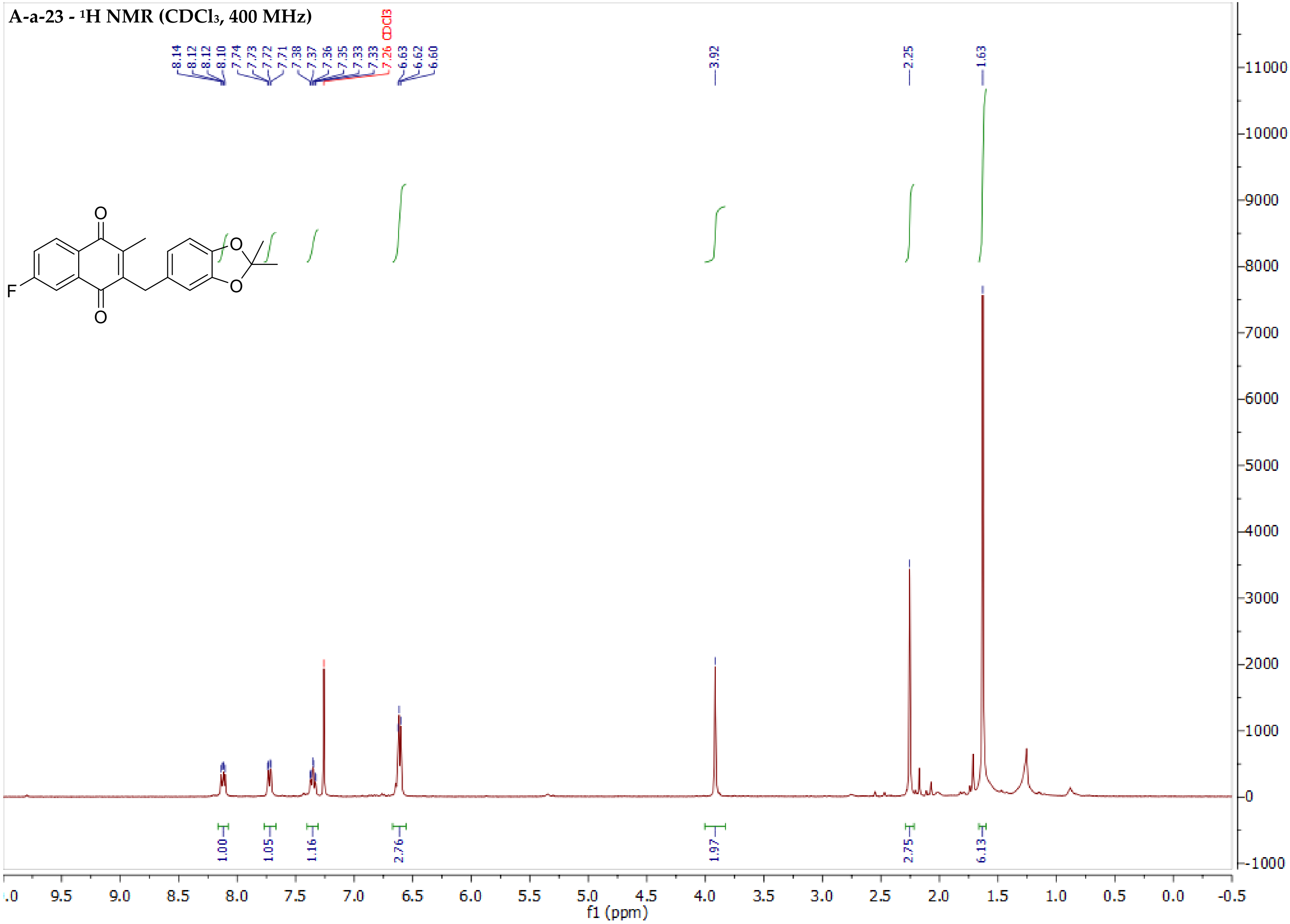

A-a-23 - $^{13}C\{^{1}H\}$ NMR ($CDCl_3$, 101 MHz)

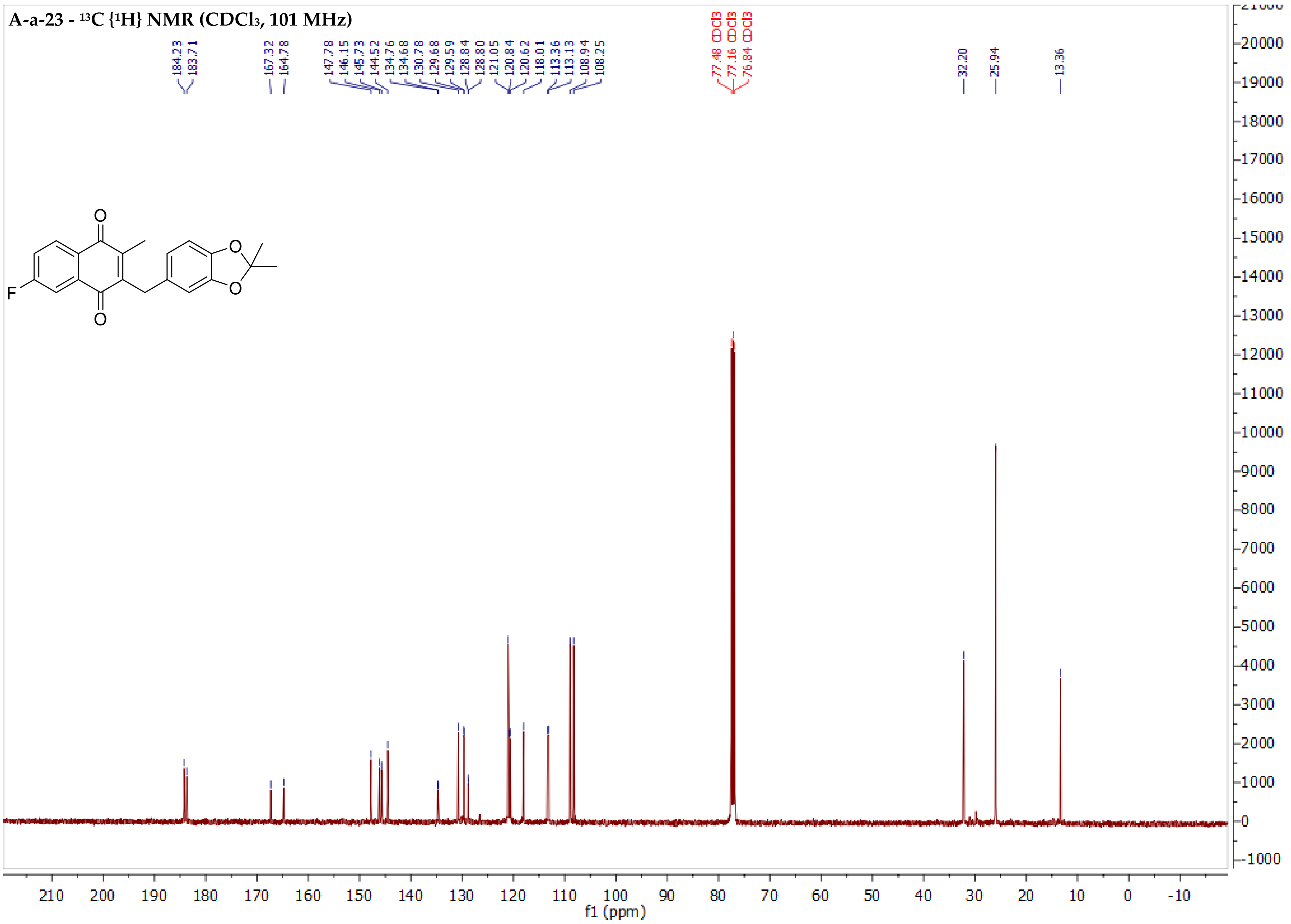

A-a-23 - ^{19}F NMR ($CDCl_3$, 377 MHz)

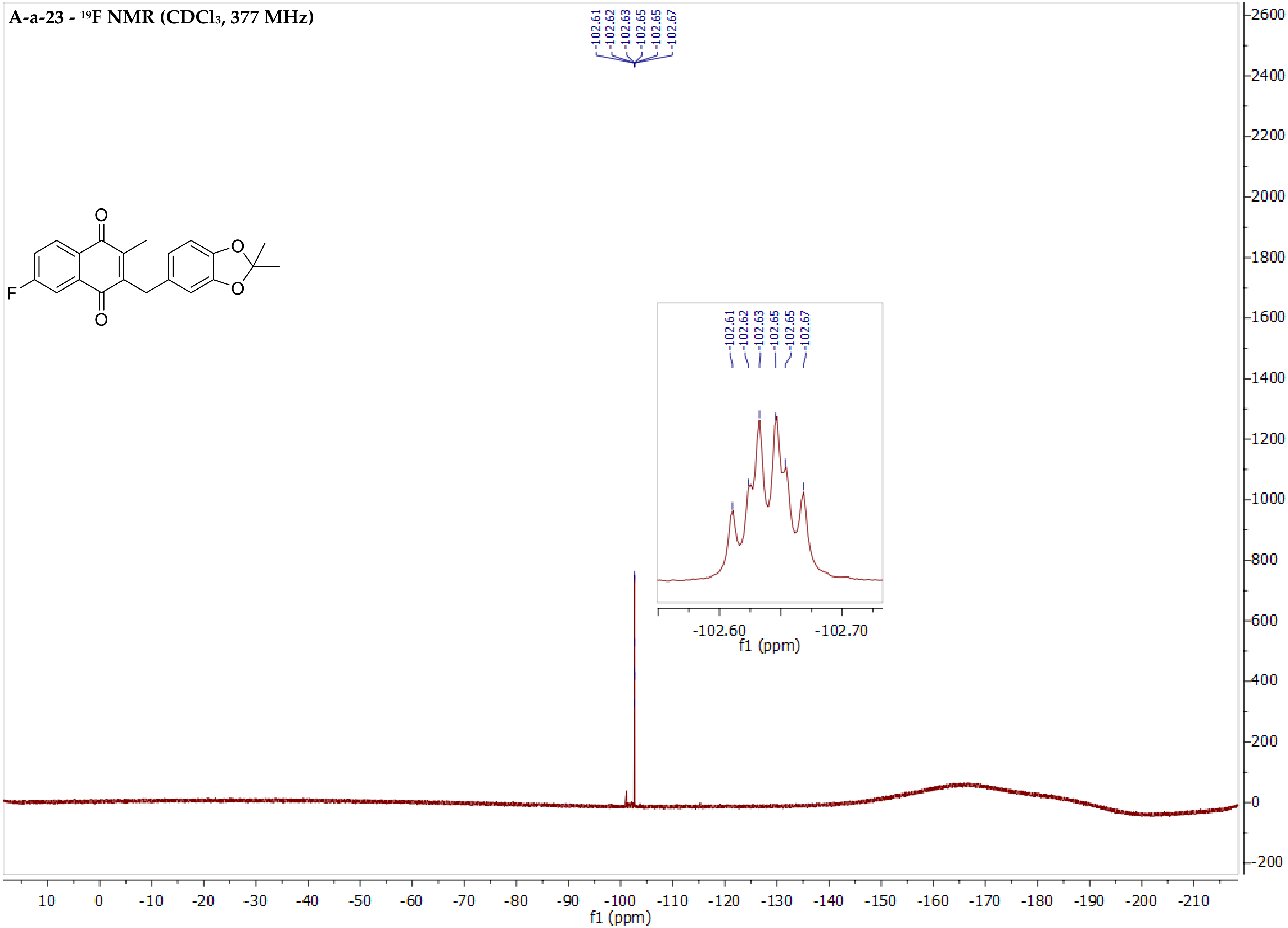

A-a-24 - ^{1}H NMR ($CDCl_3$, 400 MHz)

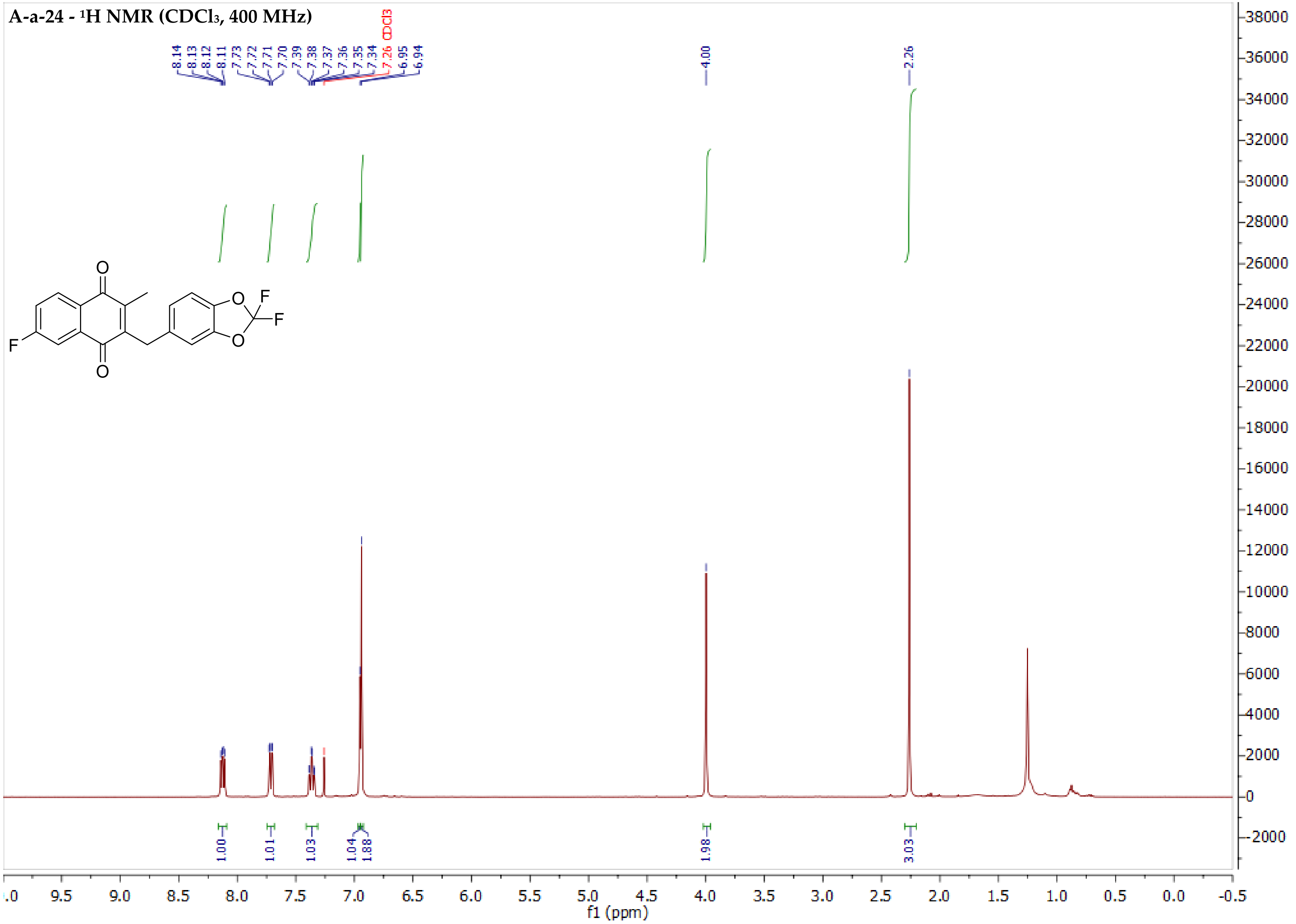

A-a-24 - $^{13}C\{^1H\}$ NMR ($CDCl_3$, 101 MHz)

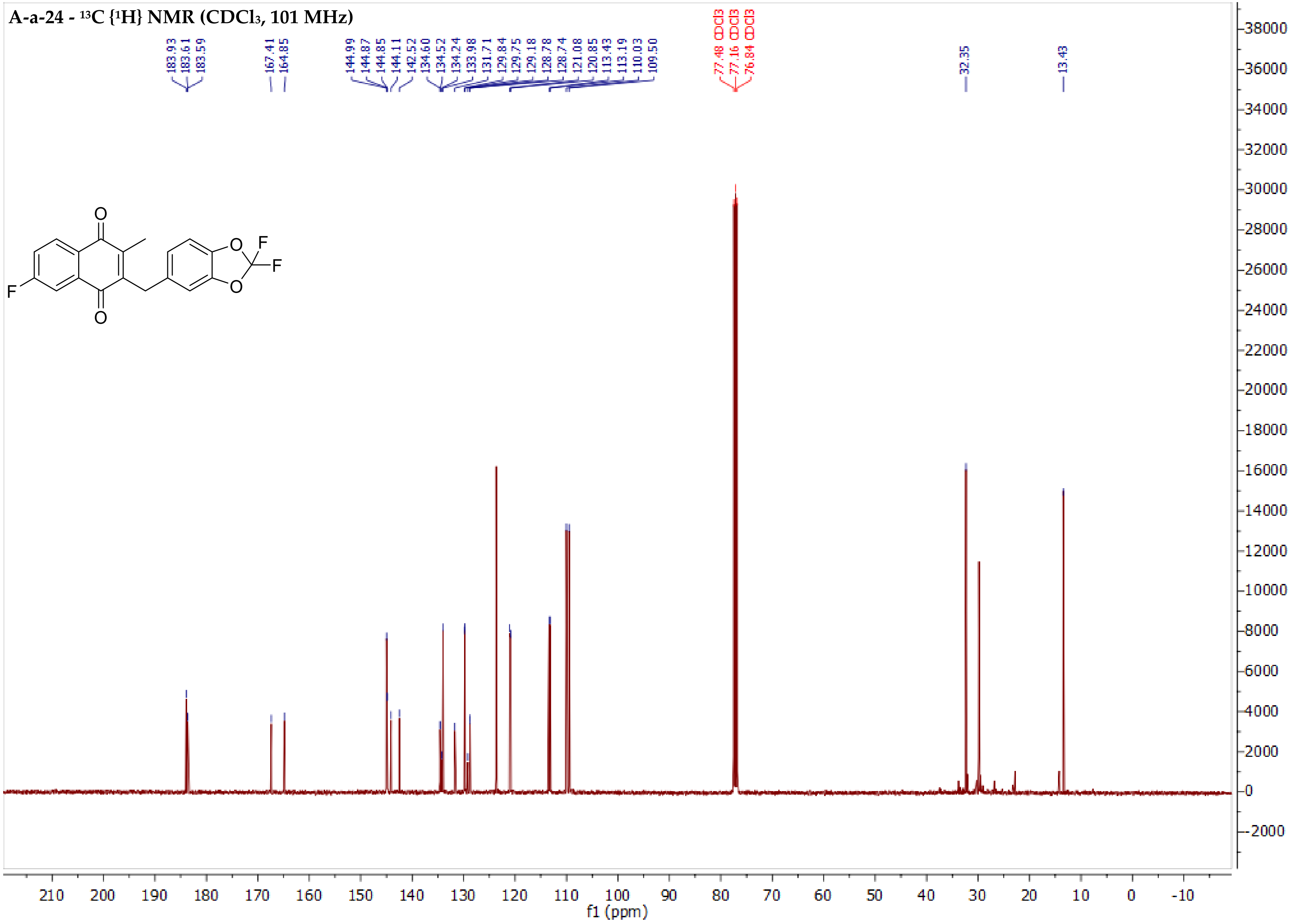

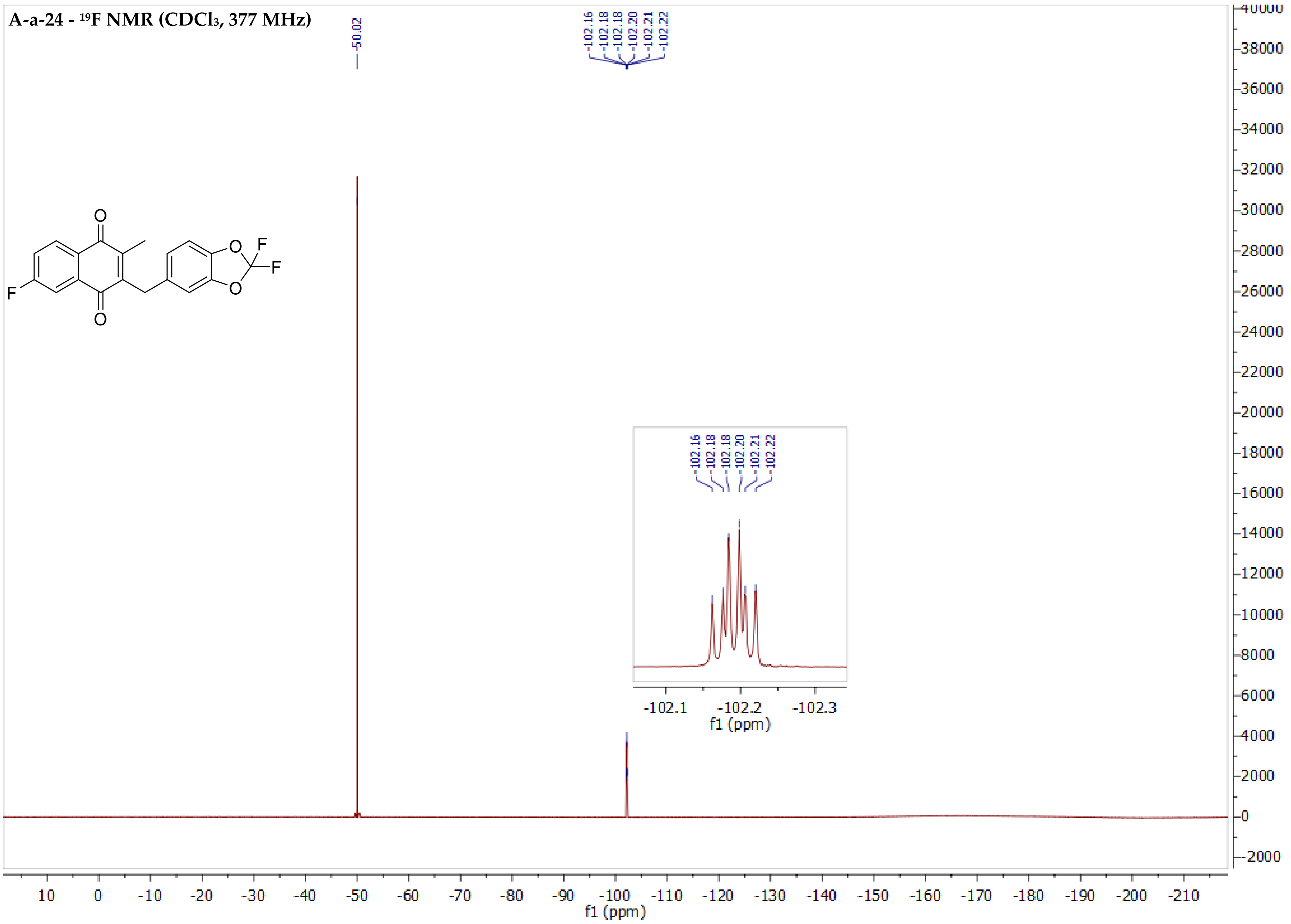

A-a-24 - 19F NMR (CDCl3, 377 MHz)
-50.02
-102.16
-102.18
-102.18
-102.20
-102.21
-102.22
f1 (ppm)

A-a-25 - ^{1}H NMR (CDCl3, 400 MHz)

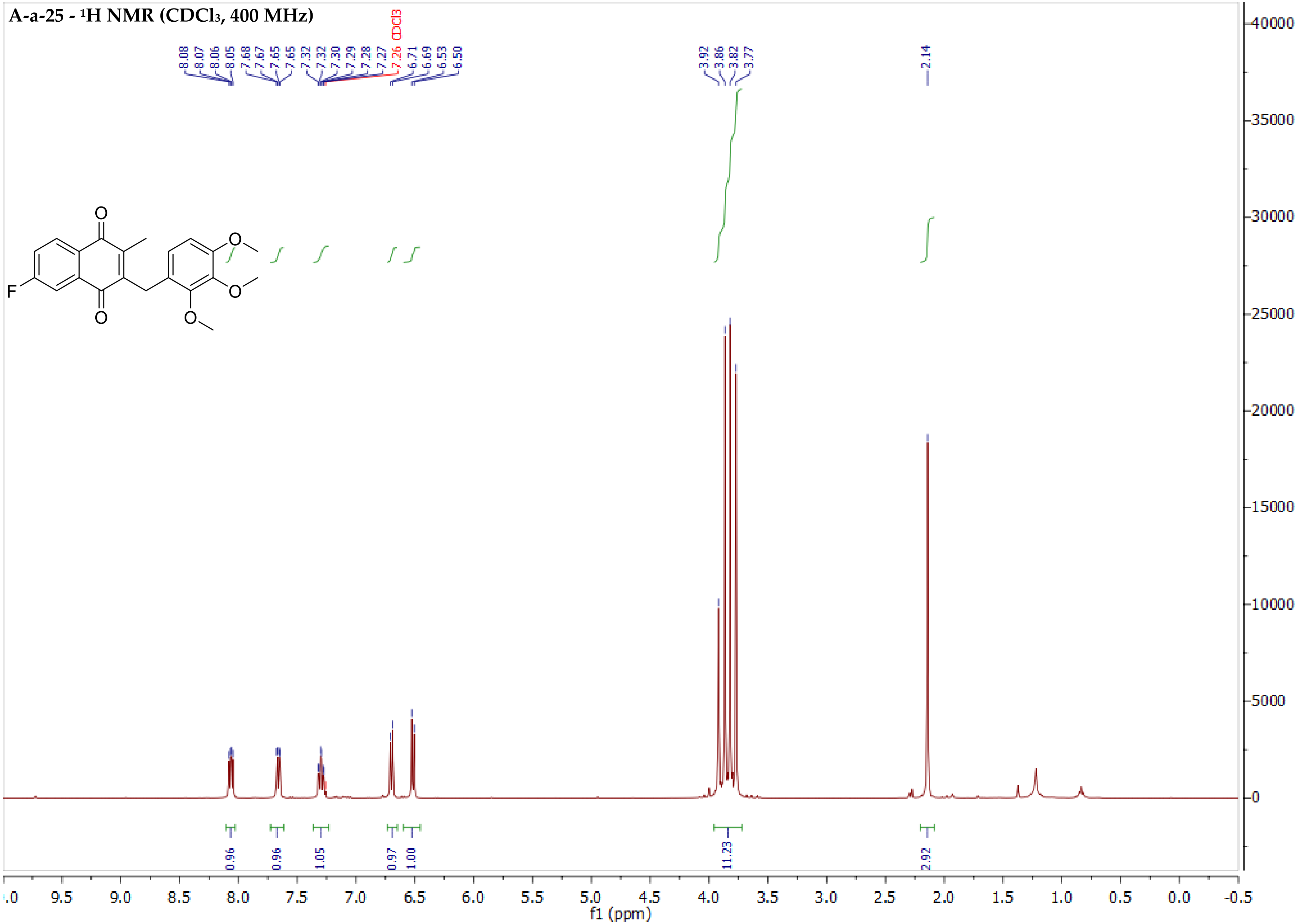

A-a-25 - $^{13}C\{^{1}H\}$ NMR ($CDCl_3$, 101 MHz)

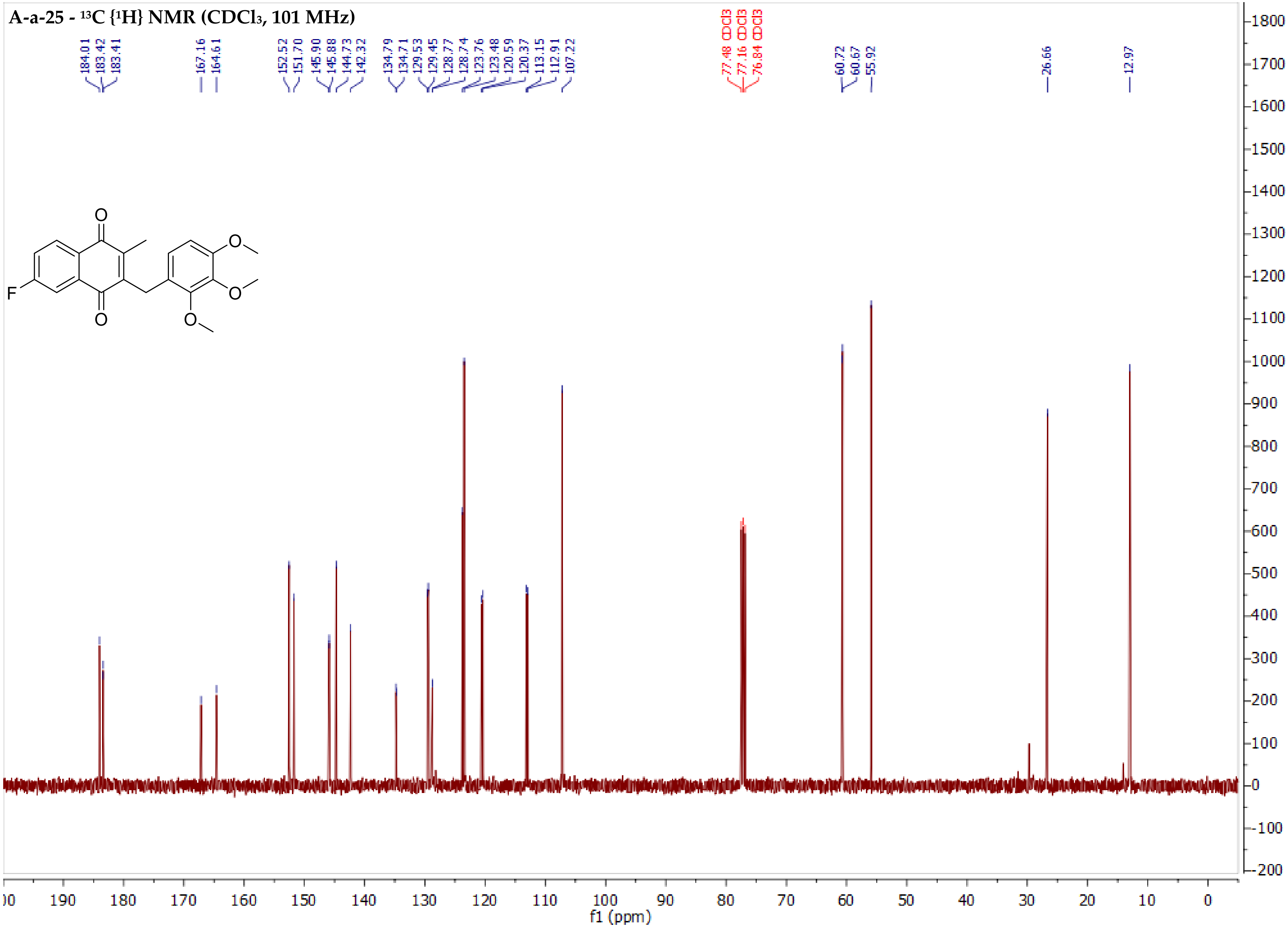

A-a-25 - ^{19}F NMR ($CDCl_3$, 377 MHz)

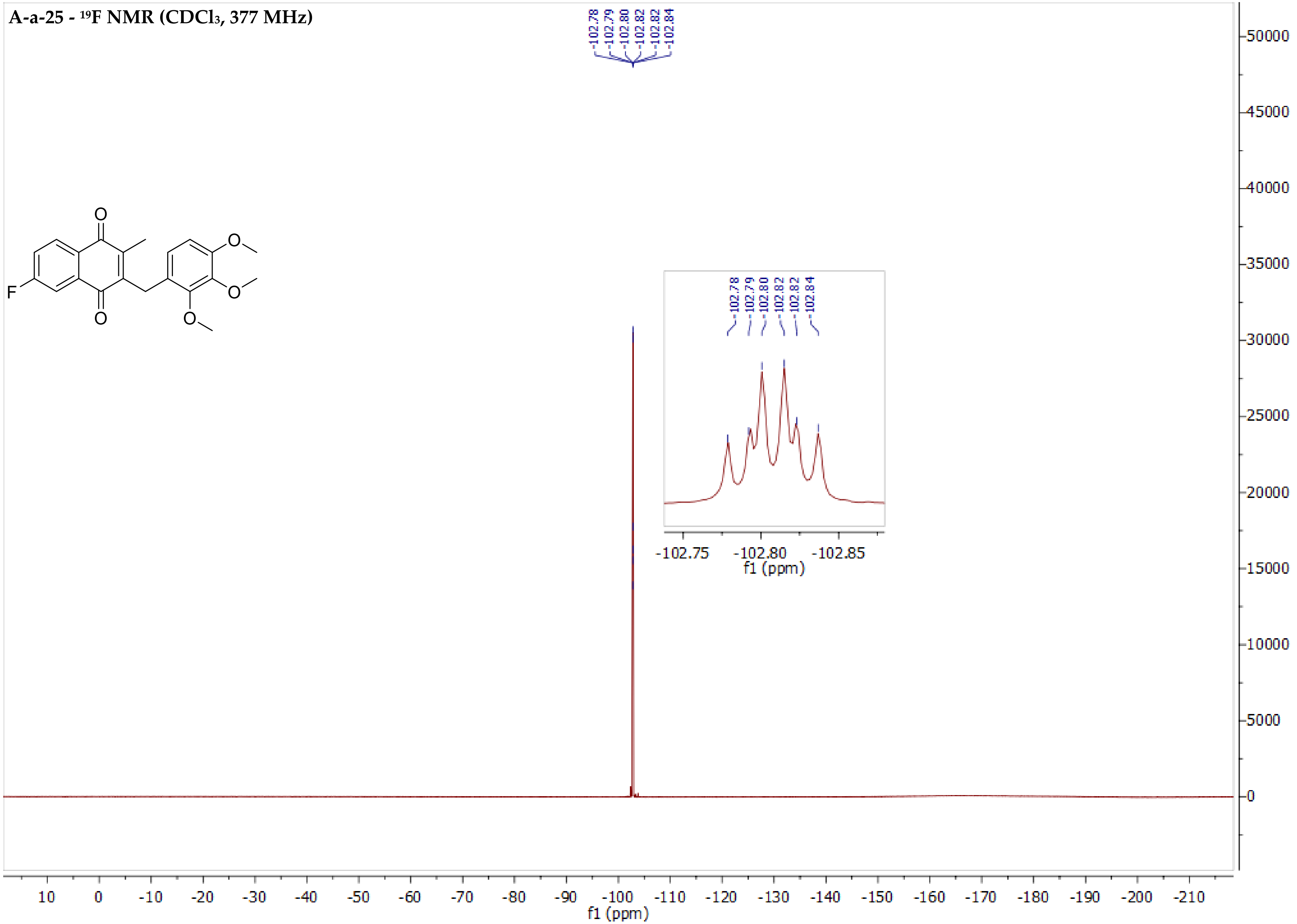

A-a-26 - ^1H NMR ($CDCl_3$, 400 MHz)

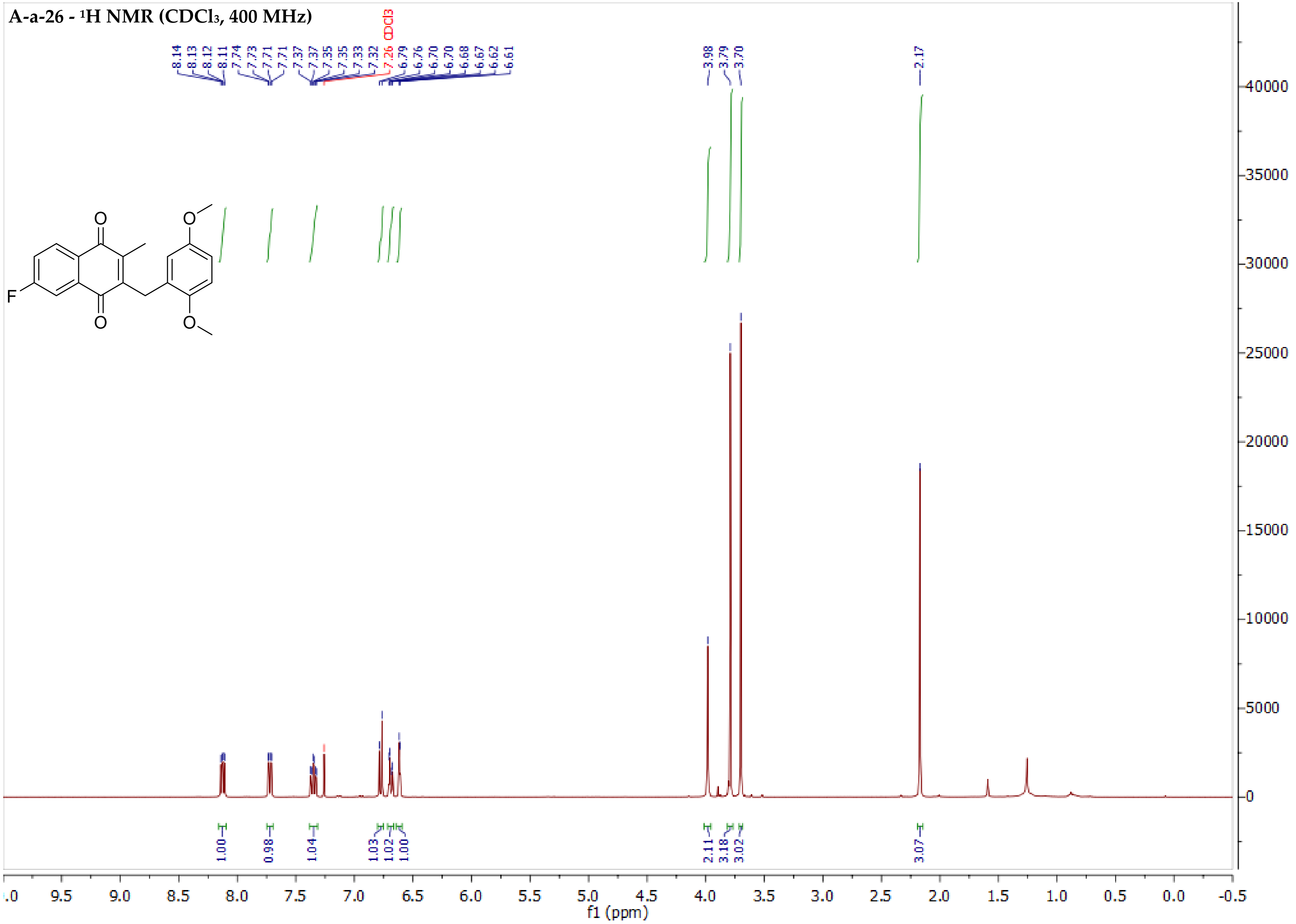

A-a-26 - $^{13}C\{^1H\}$ NMR ($CDCl_3$, 101 MHz)

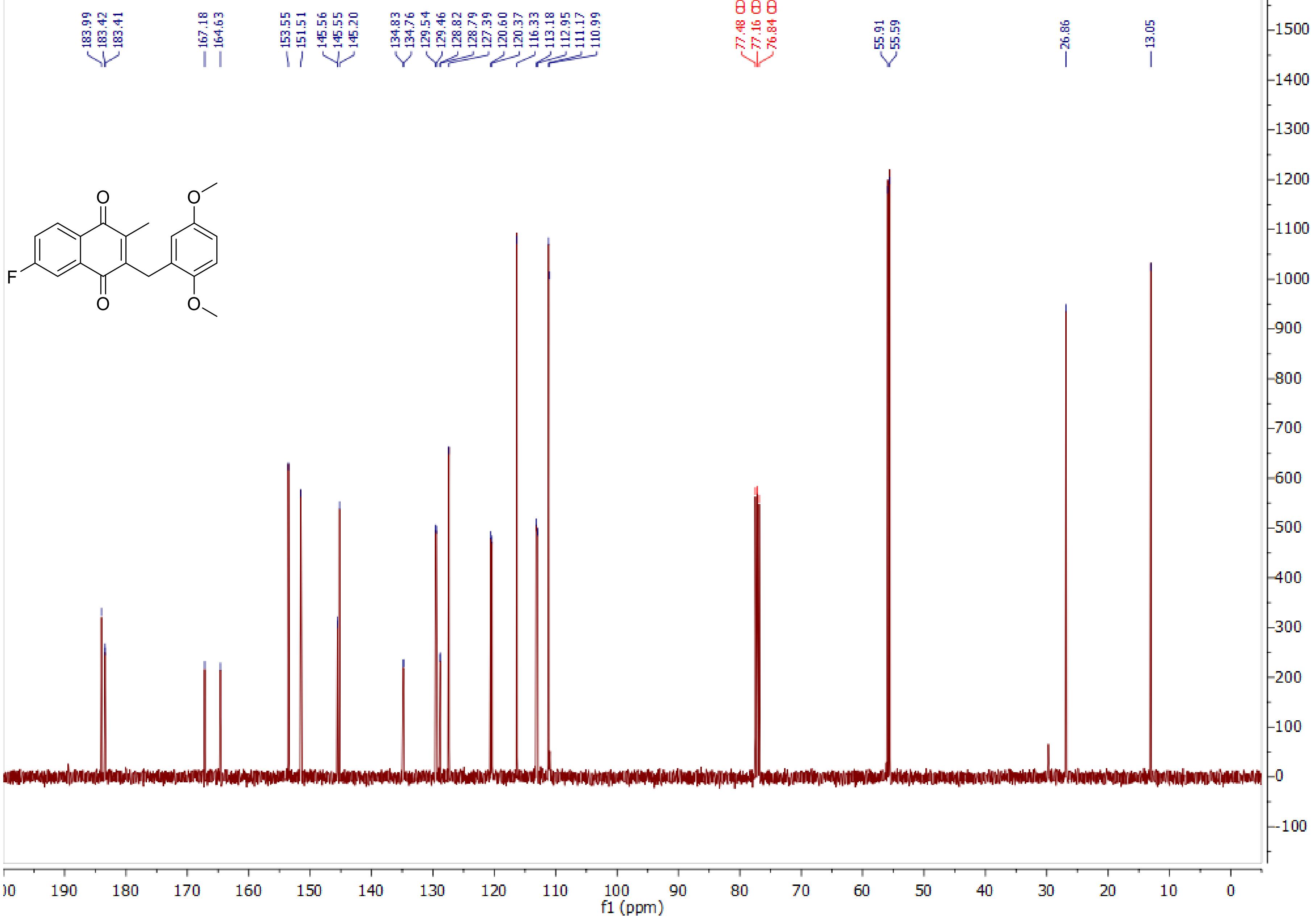

A-a-26 - ^{19}F NMR ($CDCl_3$, 377 MHz)

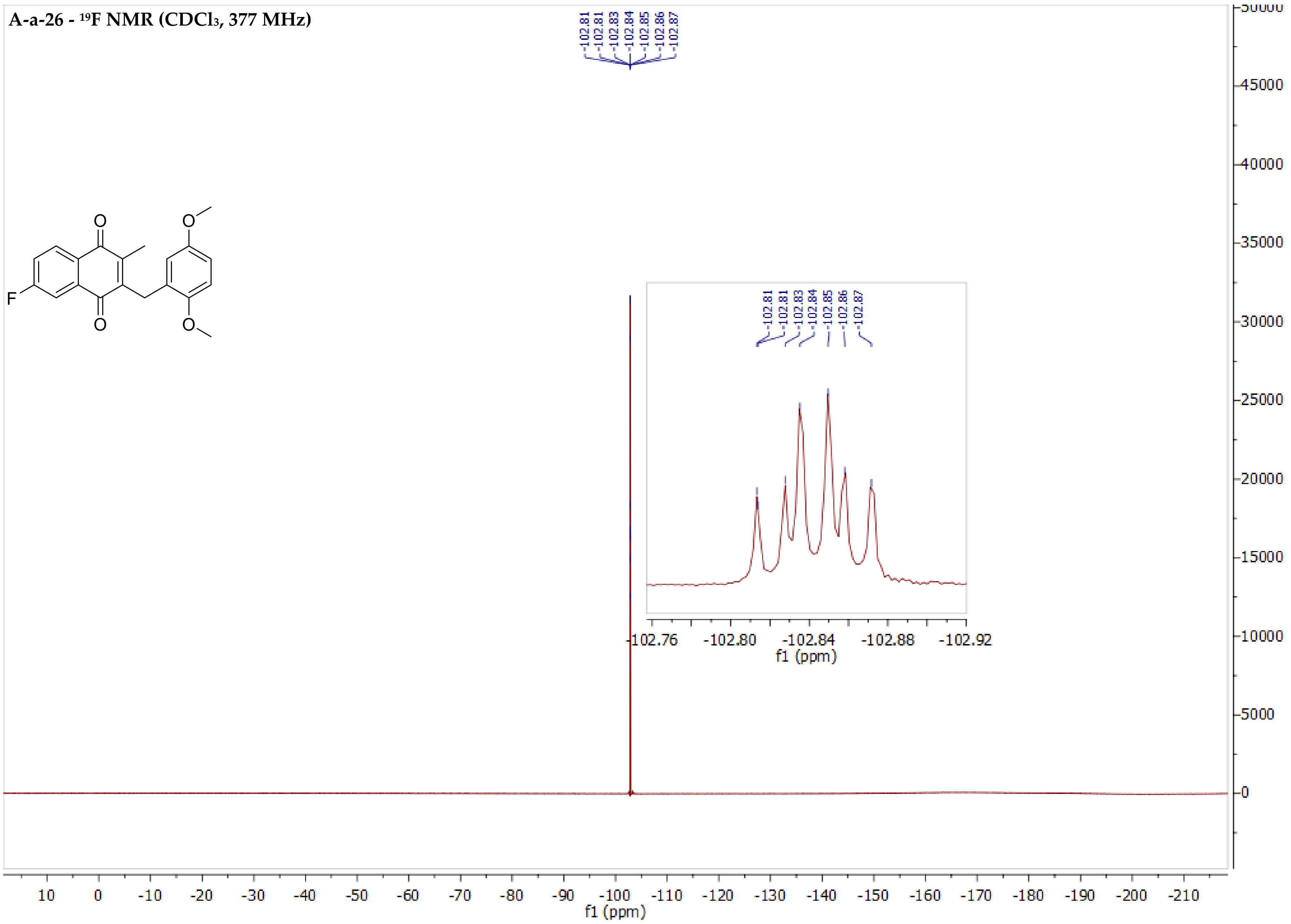

A-a-27 - ^{1}H NMR (CDCl$_3$, 400 MHz)

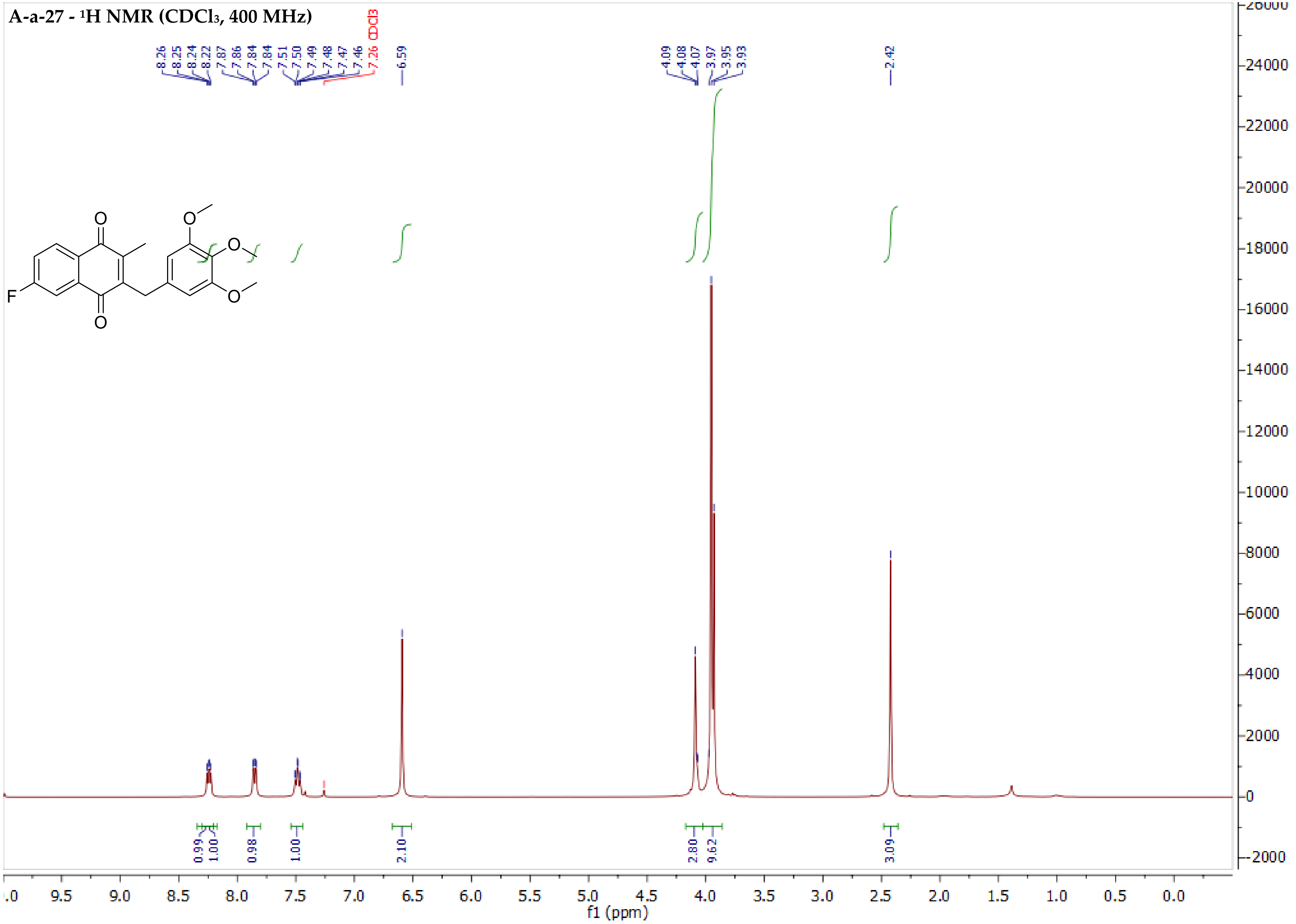

A-a-27 - $^{13}C\{^{1}H\}$ NMR ($CDCl_3$, 101 MHz)

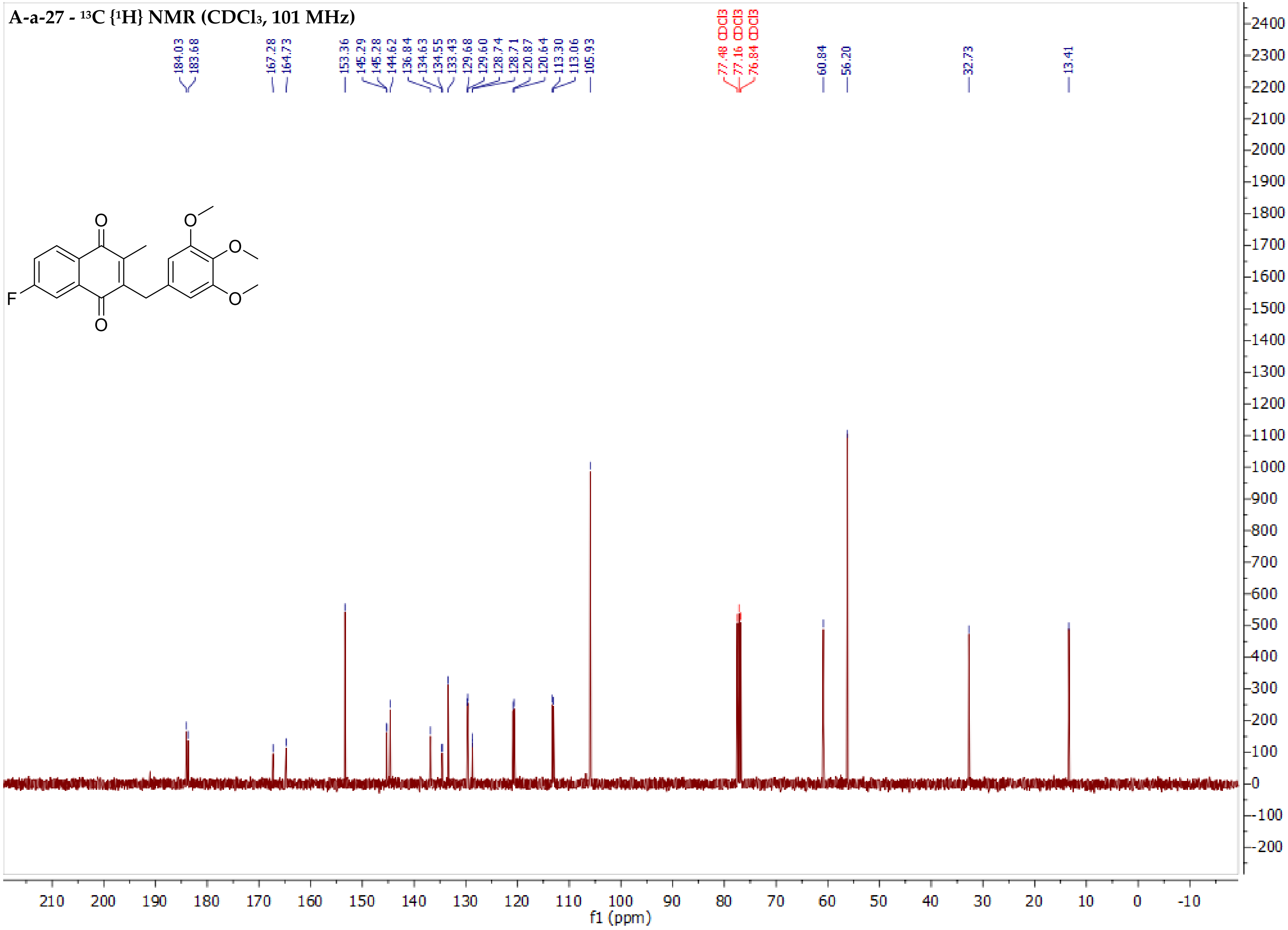

A-a-27 - ^{19}F NMR (CDCl$_3$, 377 MHz)

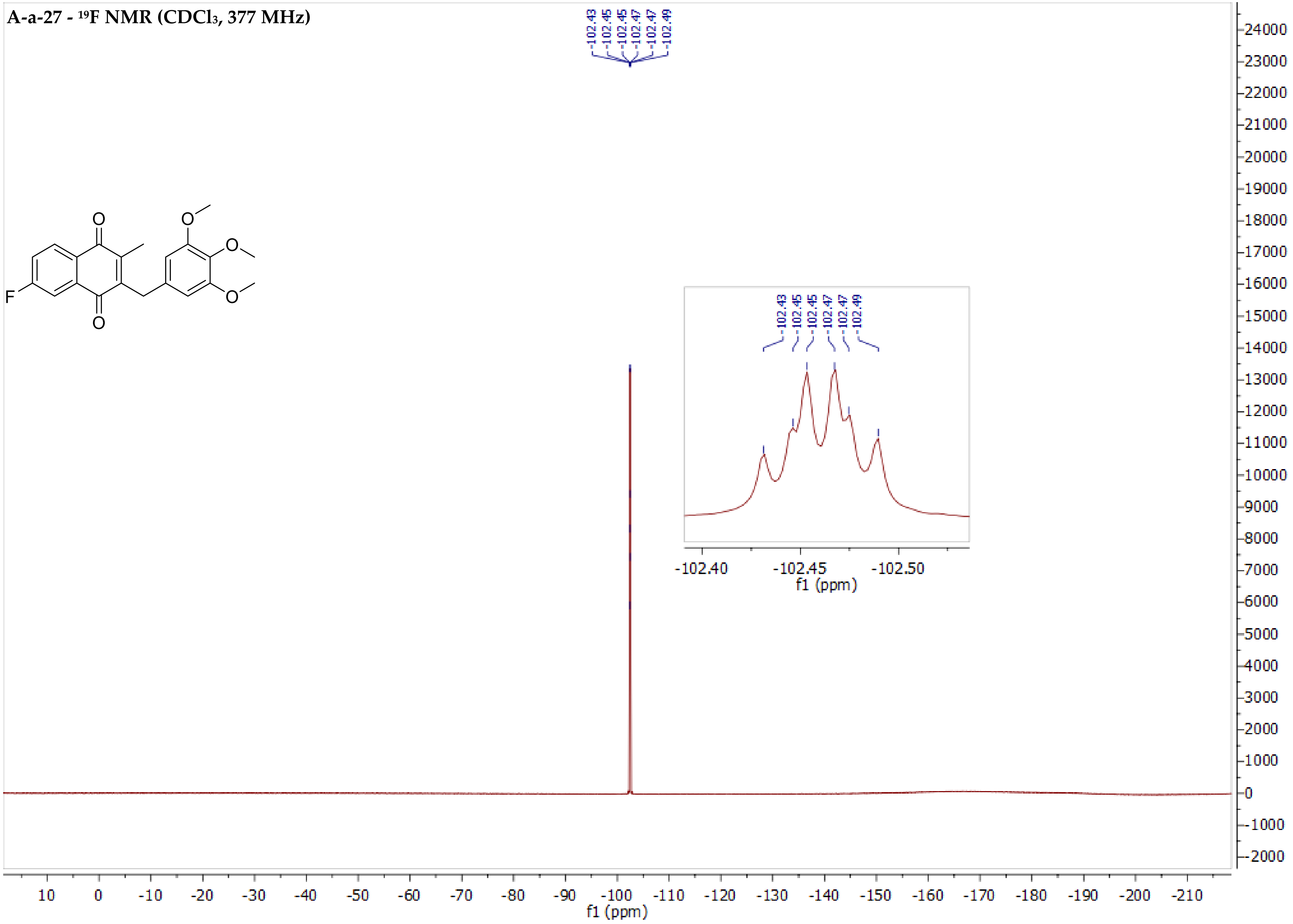

A-a-28 - ¹H NMR (CDCl₃, 400 MHz)

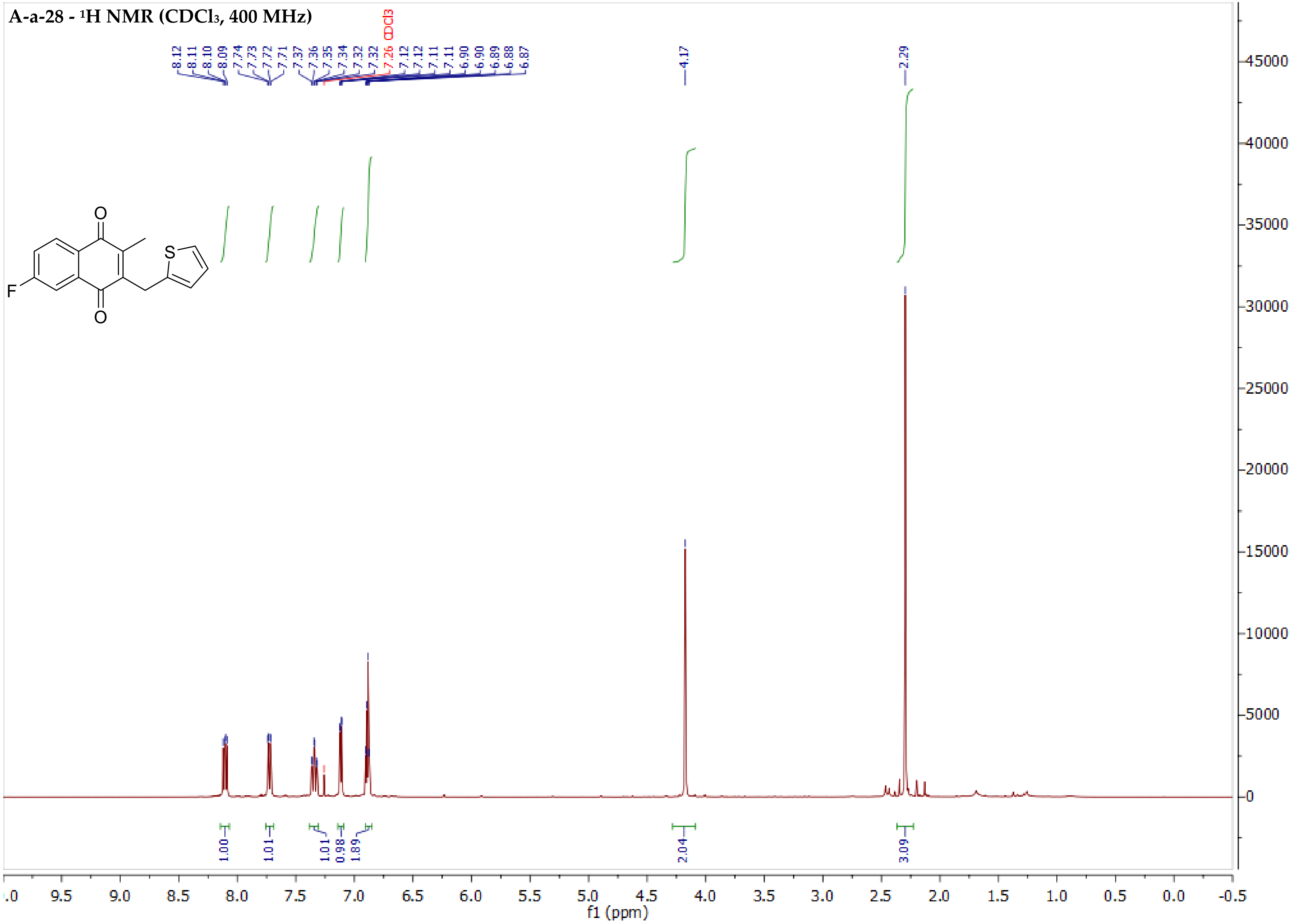

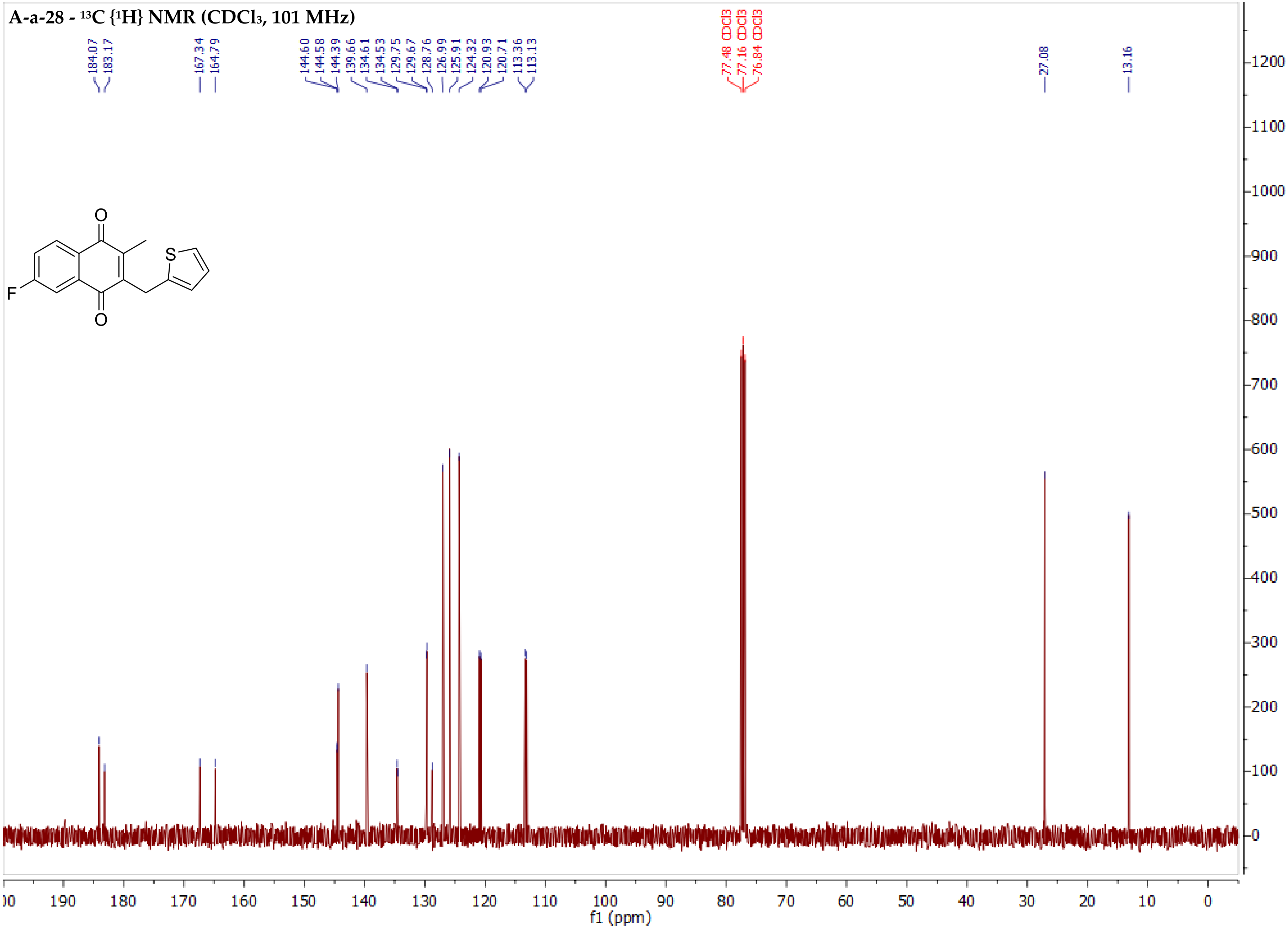

A-a-28 - ¹³C {¹H} NMR (CDCl₃, 101 MHz)
184.07
183.17
167.34
164.79
144.60
144.58
144.39
139.66
134.61
134.53
129.75
129.67
128.76
126.99
125.91
124.32
120.93
120.71
113.36
113.13
77.48 CDCl3
77.16 CDCl3
76.84 CDCl3
27.08
13.16
f1 (ppm)

A-a-28 - ^{19}F NMR ($CDCl_3$, 377 MHz)

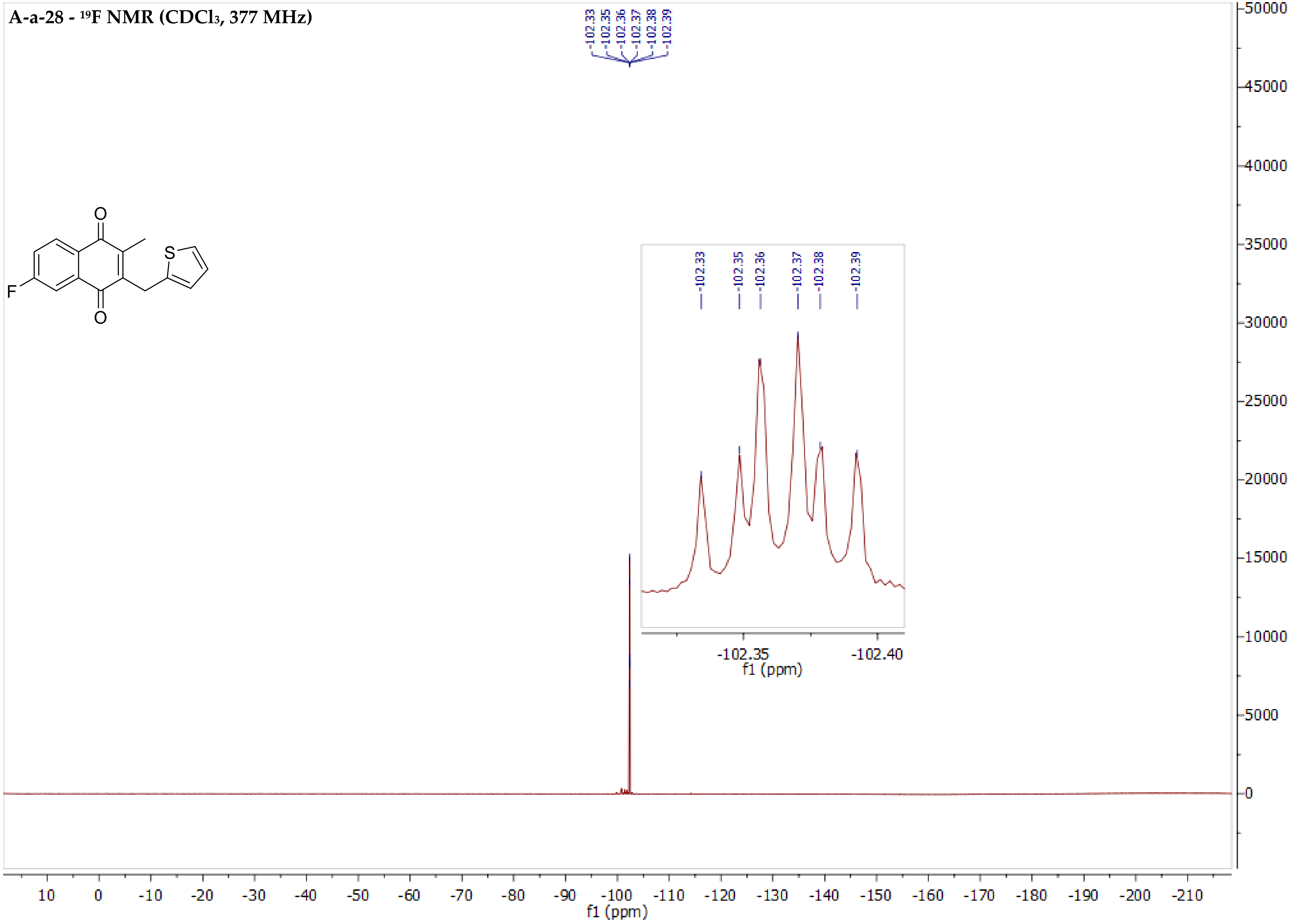

A-a-29 - ^{1}H NMR ($CDCl_3$, 400 MHz)

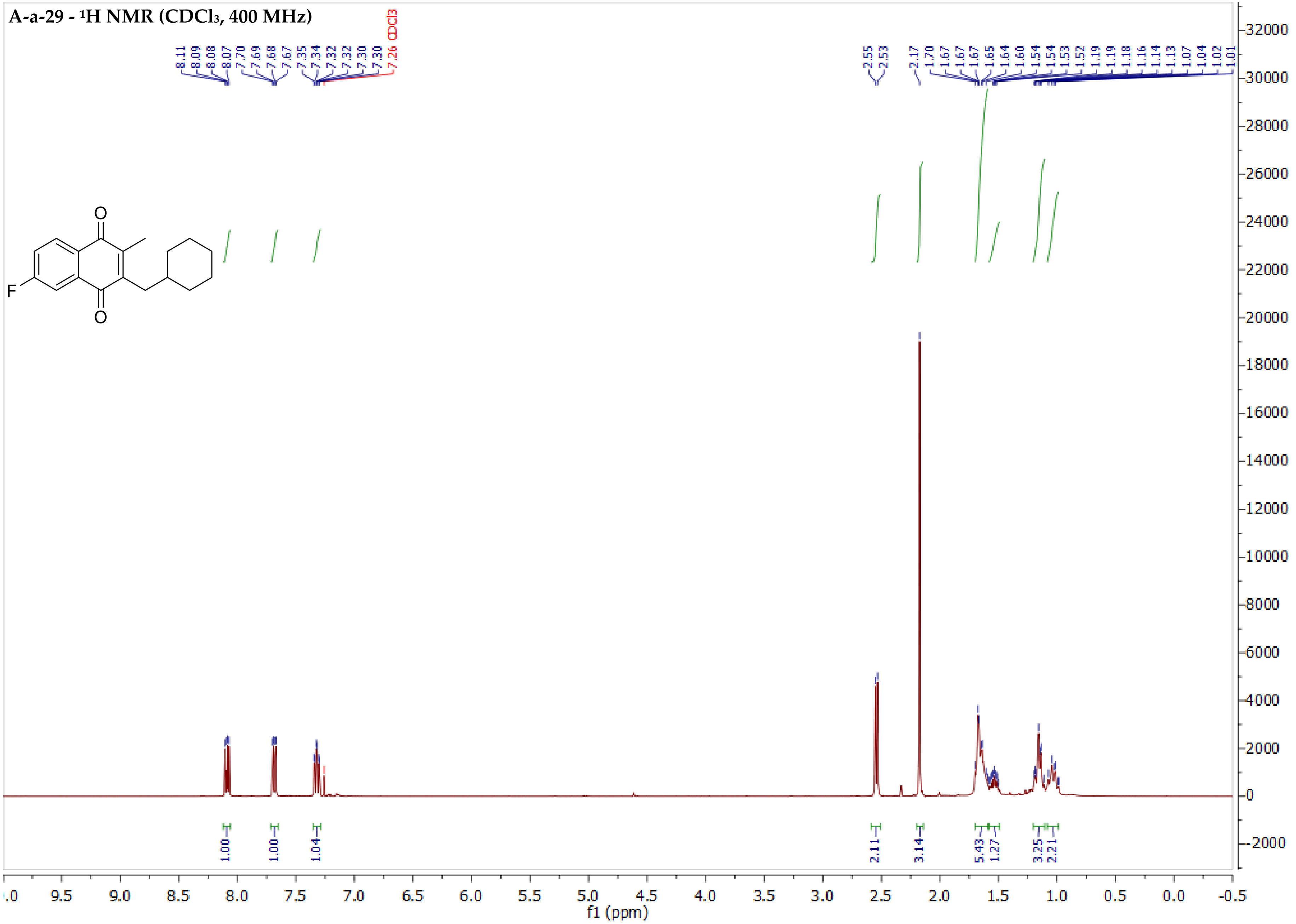

A-a-29 - $^{13}C\{^1H\}$ NMR ($CDCl_3$, 101 MHz)

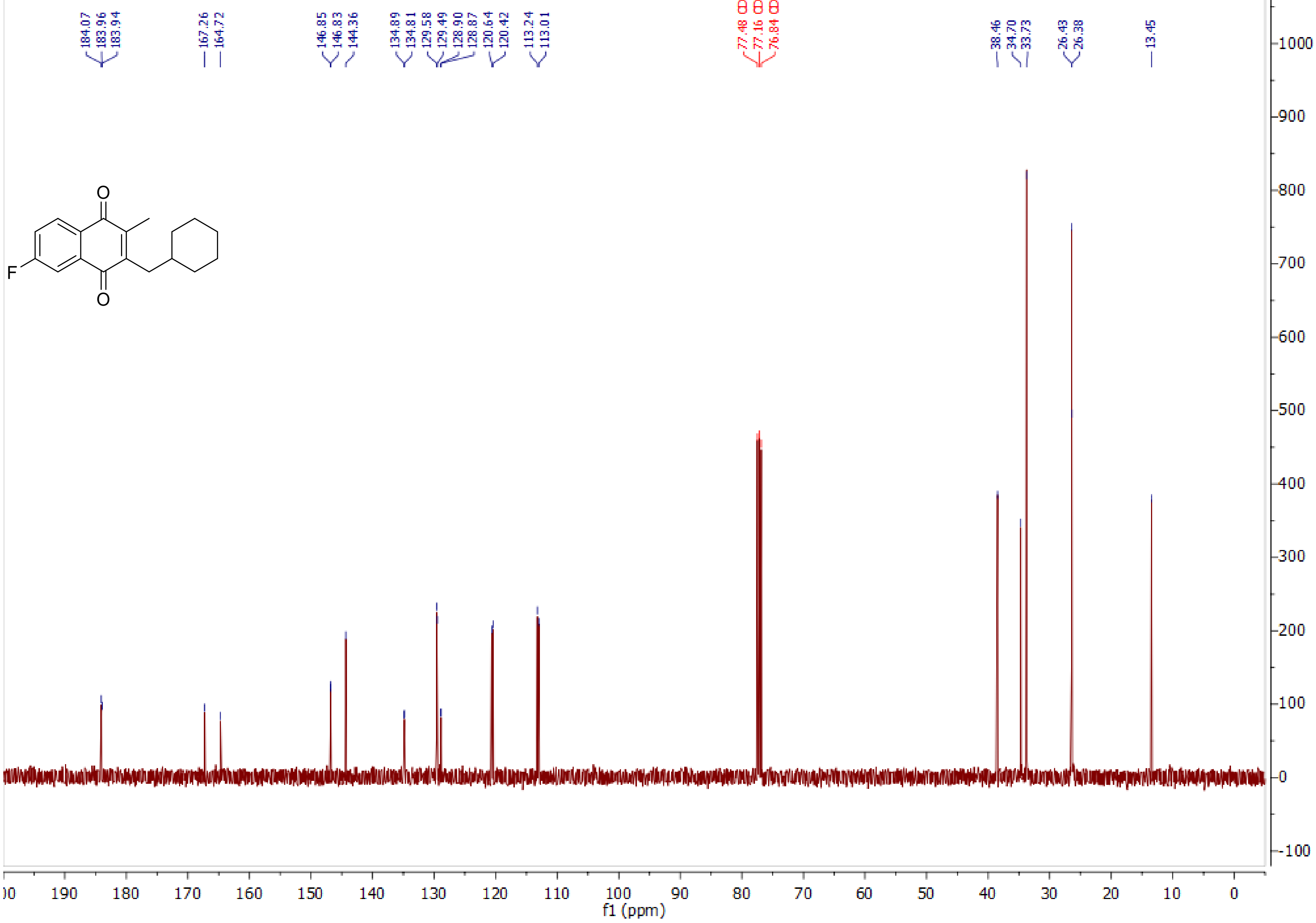

A-a-29 - ^{19}F NMR ($CDCl_3$, 377 MHz)

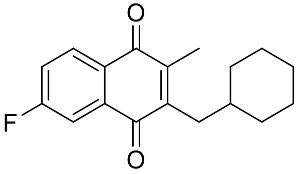

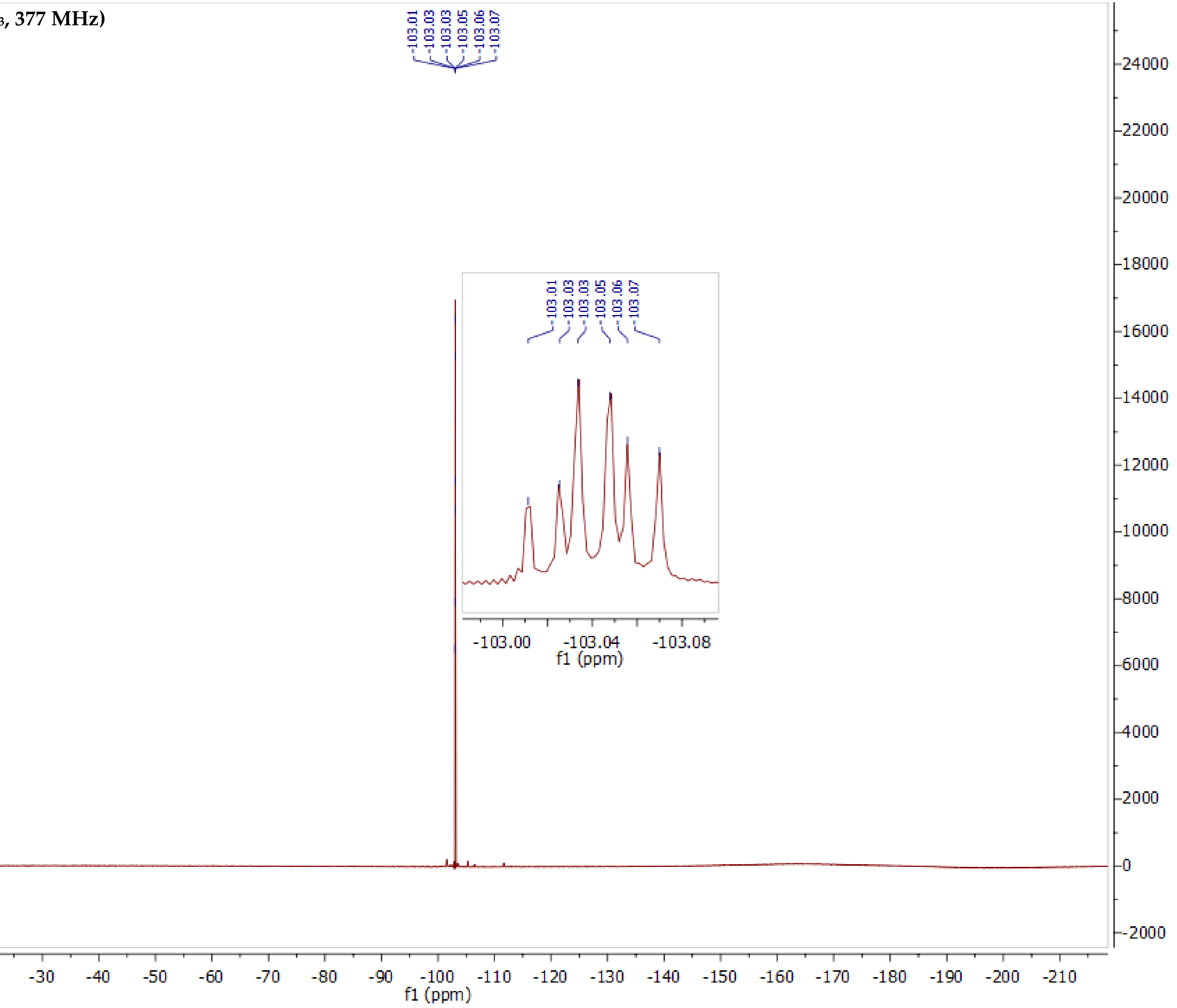

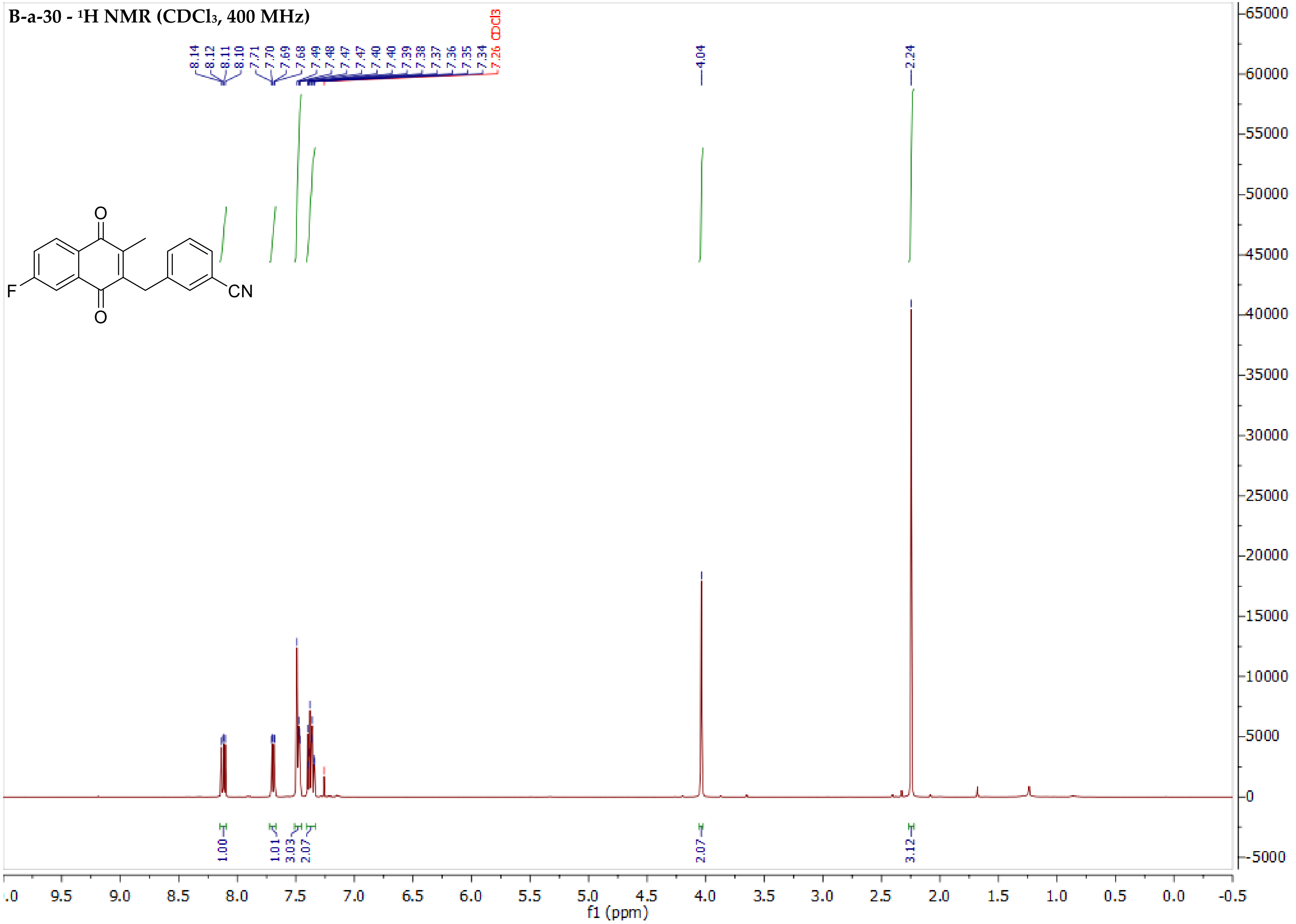
B-a-30 - 1H NMR (CDCl3, 400 MHz)
8.14
8.12
8.11
8.10
7.71
7.70
7.69
7.68
7.49
7.48
7.47
7.47
7.40
7.40
7.39
7.38
7.37
7.36
7.35
7.34
7.26 CDCl3
4.04
2.24
O
O
F
CN
1.00
1.01
3.03
2.07
2.07
3.12
f1 (ppm)

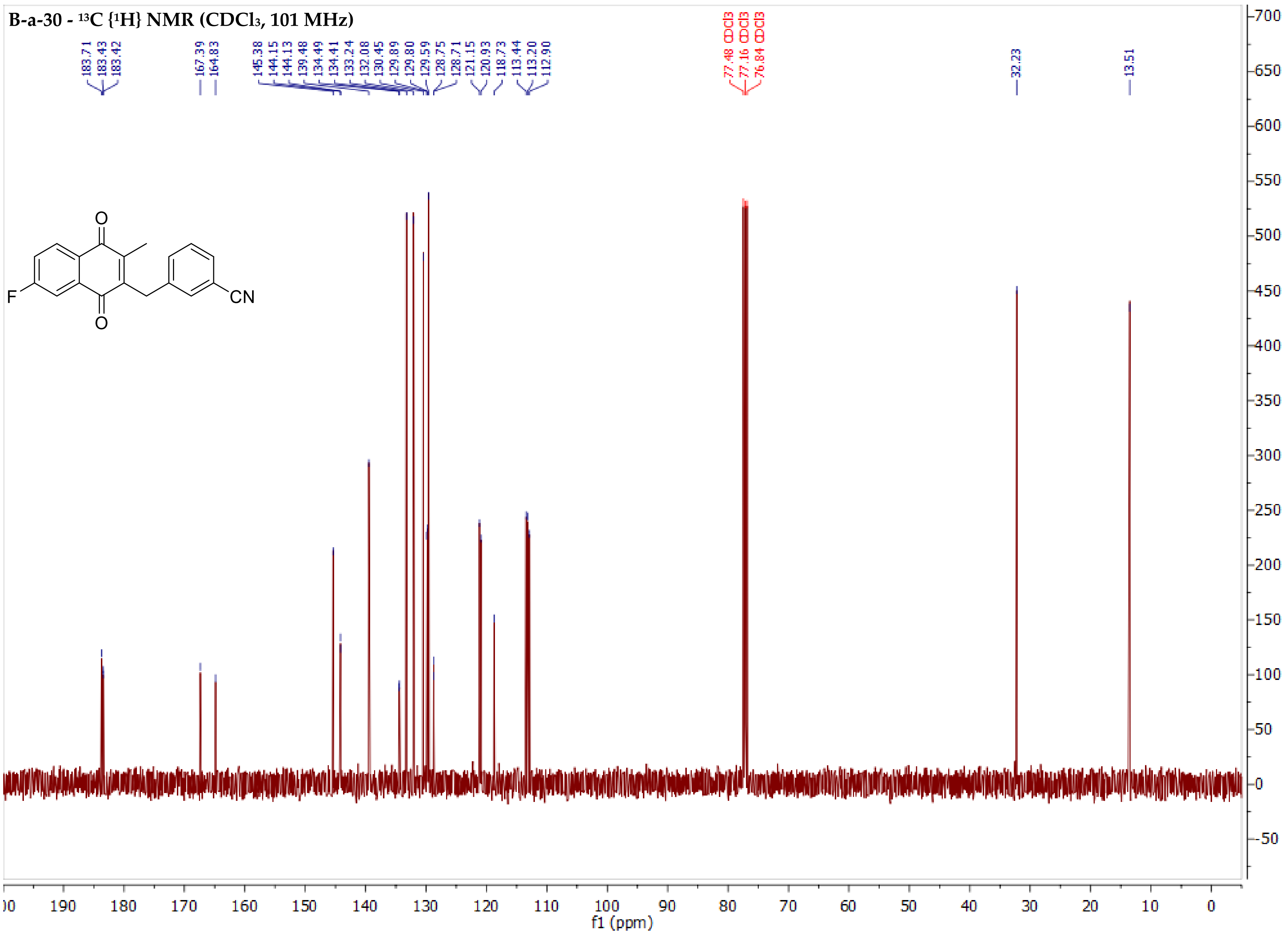
B-a-30 - 13C {1H} NMR (CDCl3, 101 MHz)
183.71
183.43
183.42
167.39
164.83
145.38
144.15
144.13
139.48
134.49
134.41
133.24
132.08
130.45
129.89
129.80
129.59
128.75
128.71
121.15
120.93
118.73
113.44
113.20
112.90
77.48 CDCl3
77.16 CDCl3
76.84 CDCl3
32.23
13.51
O
O
F
CN
f1 (ppm)

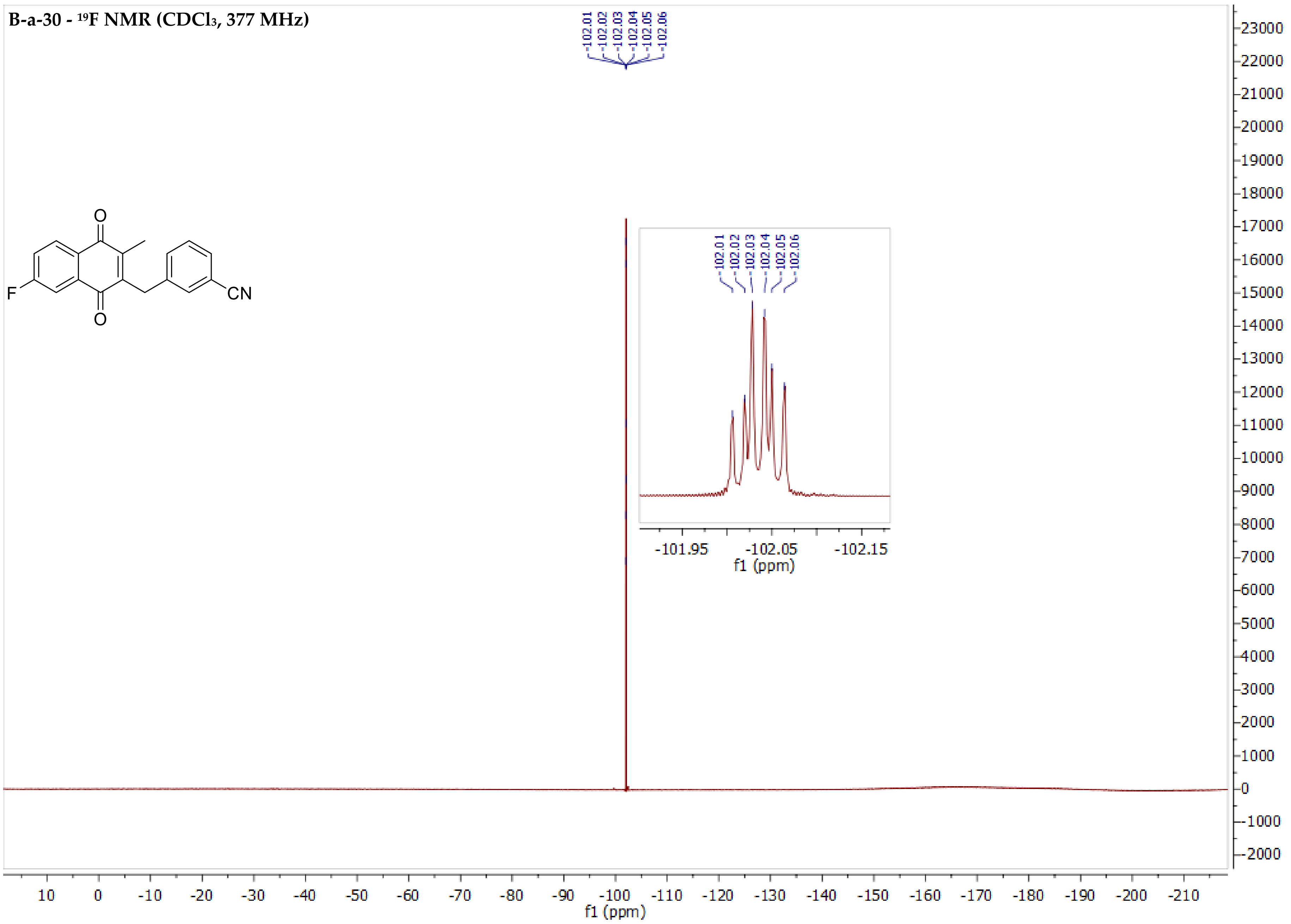

B-a-30 - 19F NMR (CDCl3, 377 MHz)
-102.01
-102.02
-102.03
-102.04
-102.05
-102.06
F
O
O
CN
-101.95
-102.05
-102.15
f1 (ppm)
23000
22000
21000
20000
19000
18000
17000
16000
15000
14000
13000
12000
11000
10000
9000
8000
7000
6000
5000
4000
3000
2000
1000
0
-1000
-2000
10
0
-10
-20
-30
-40
-50
-60
-70
-80
-90
-100
-110
-120
-130
-140
-150
-160
-170
-180
-190
-200
-210
f1 (ppm)

B-a-31 - ^{1}H NMR ($CDCl_3$, 400 MHz)

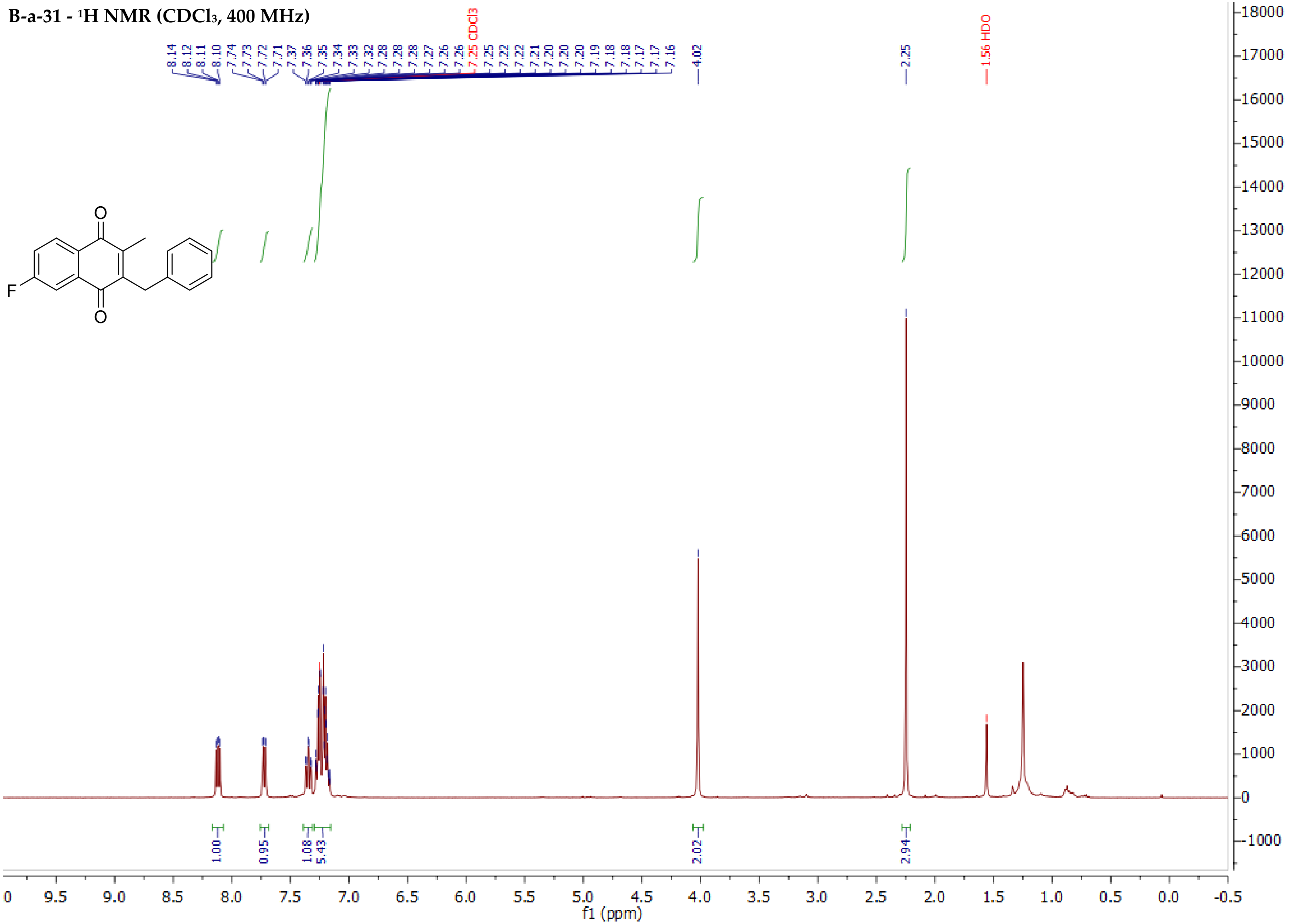

B-a-31 - $^{13}C\{^1H\}$ NMR ($CDCl_3$, 101 MHz)

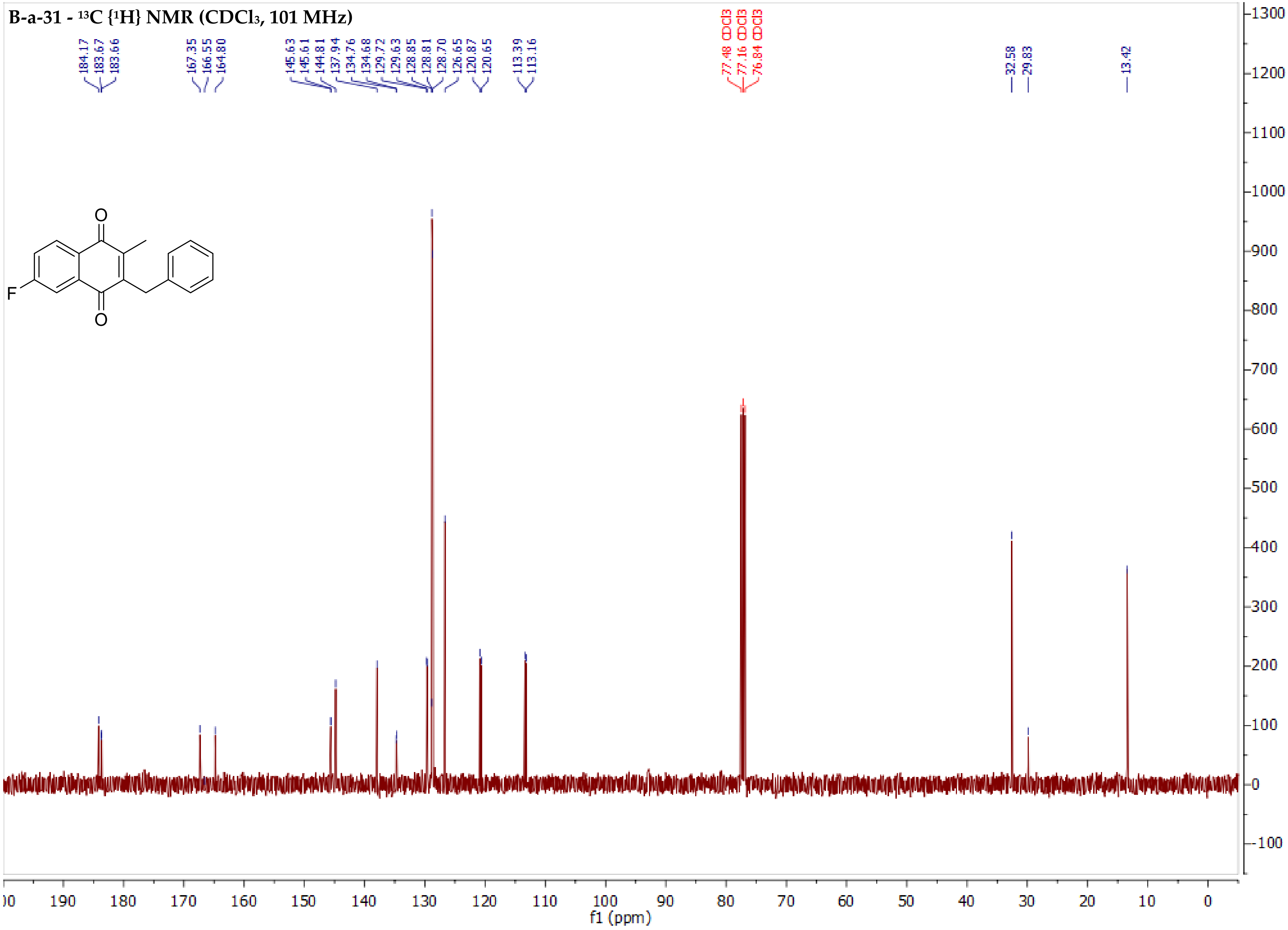

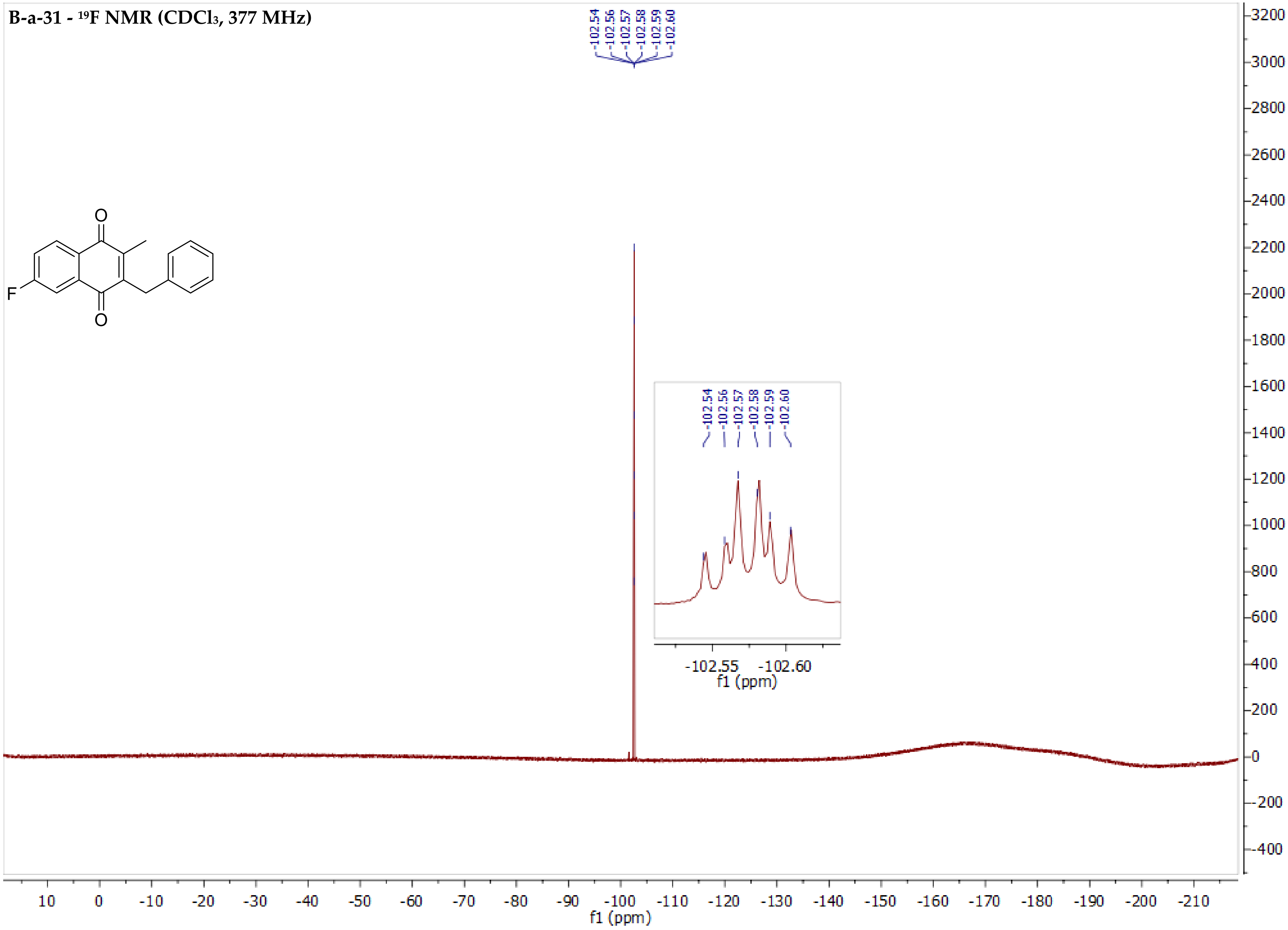
B-a-31 - ^{19}F NMR ($CDCl_3$, 377 MHz)
-102.54
-102.56
-102.57
-102.58
-102.59
-102.60
F
O
O
-102.55
-102.60
f1 (ppm)
3200
3000
2800
2600
2400
2200
2000
1800
1600
1400
1200
1000
800
600
400
200
0
-200
-400
10 0 -10 -20 -30 -40 -50 -60 -70 -80 -90 -100 -110 -120 -130 -140 -150 -160 -170 -180 -190 -200 -210
f1 (ppm)

B-a-32 - ¹H NMR (CDCl₃, 400 MHz)

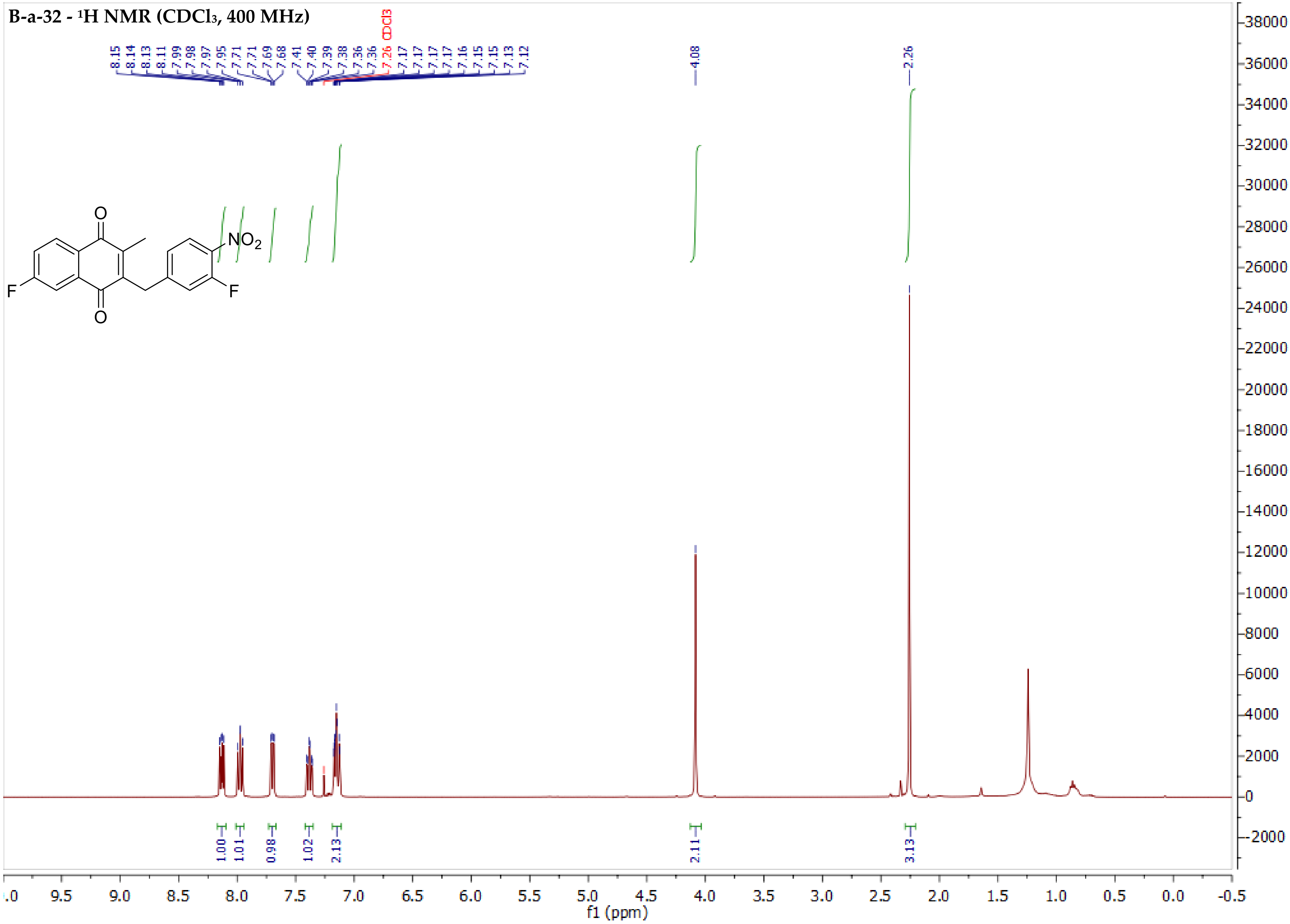

B-a-32 - $^{13}C\{^1H\}$ NMR ($CDCl_3$, 101 MHz)

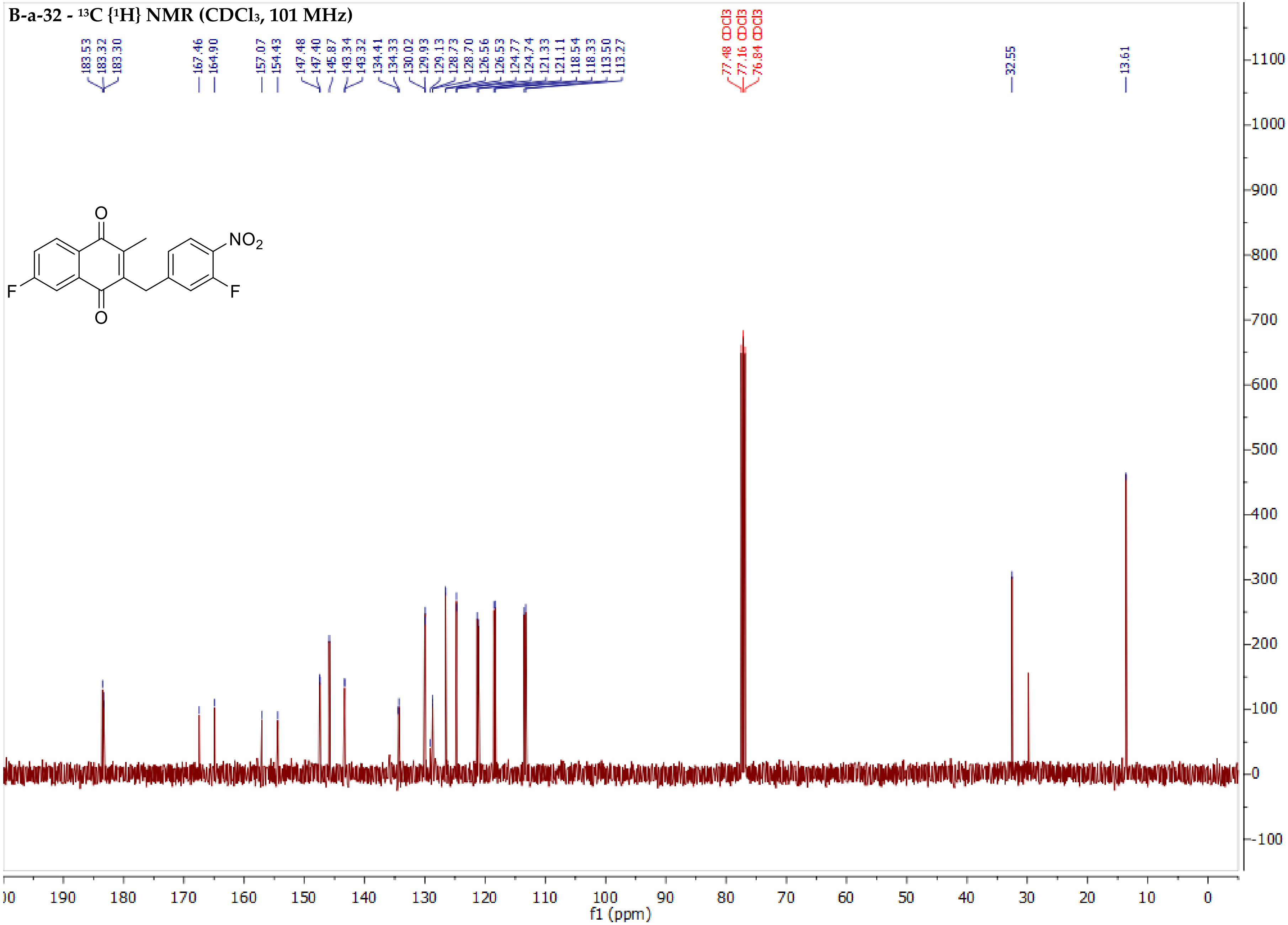

B-a-32 - ^{19}F NMR (CDCl$_3$, 377 MHz)

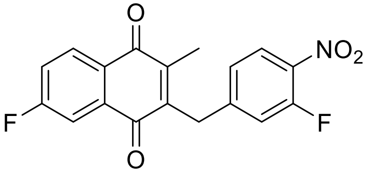

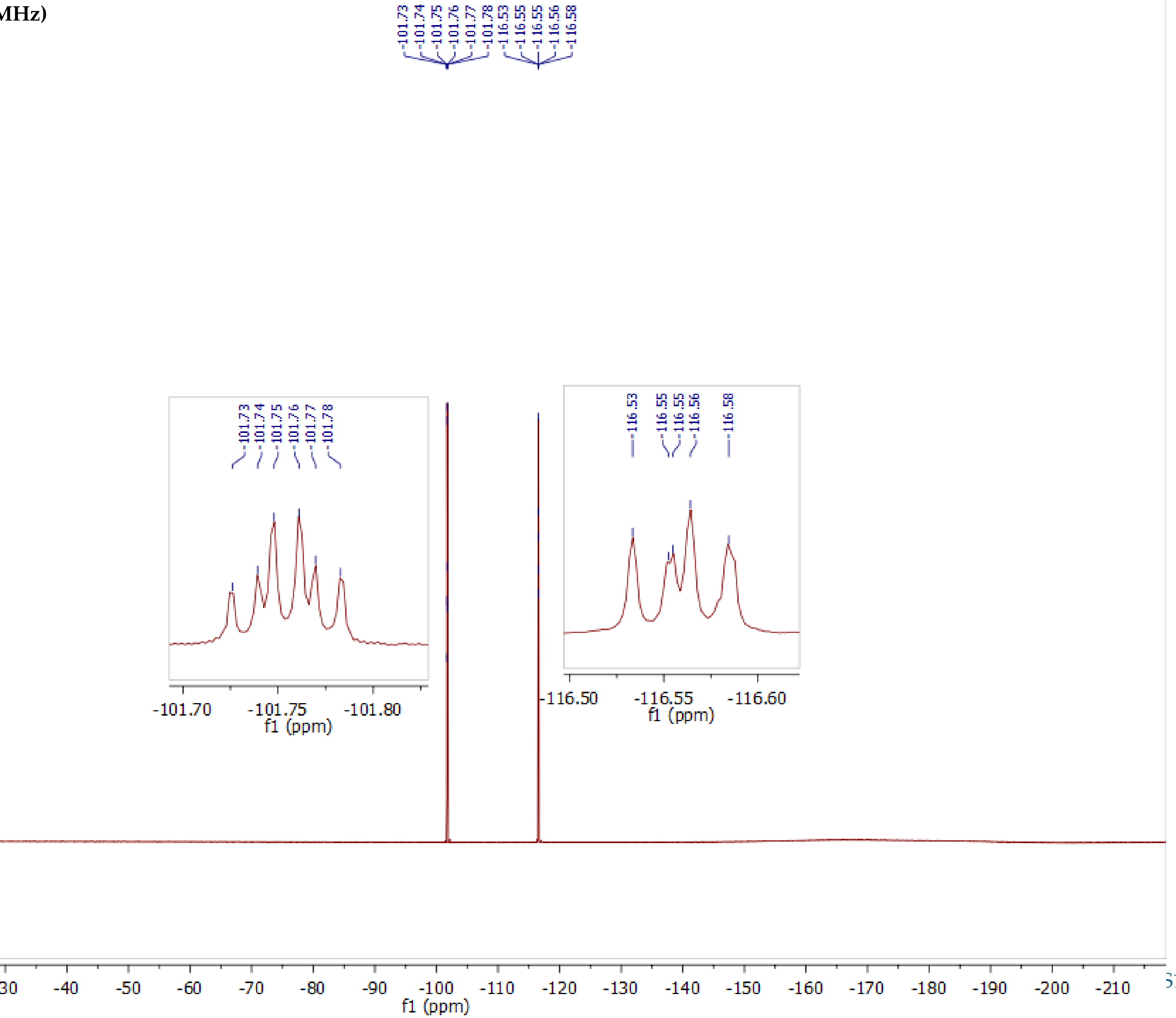

B-a-33 - ^{1}H NMR ($CDCl_3$, 400 MHz)

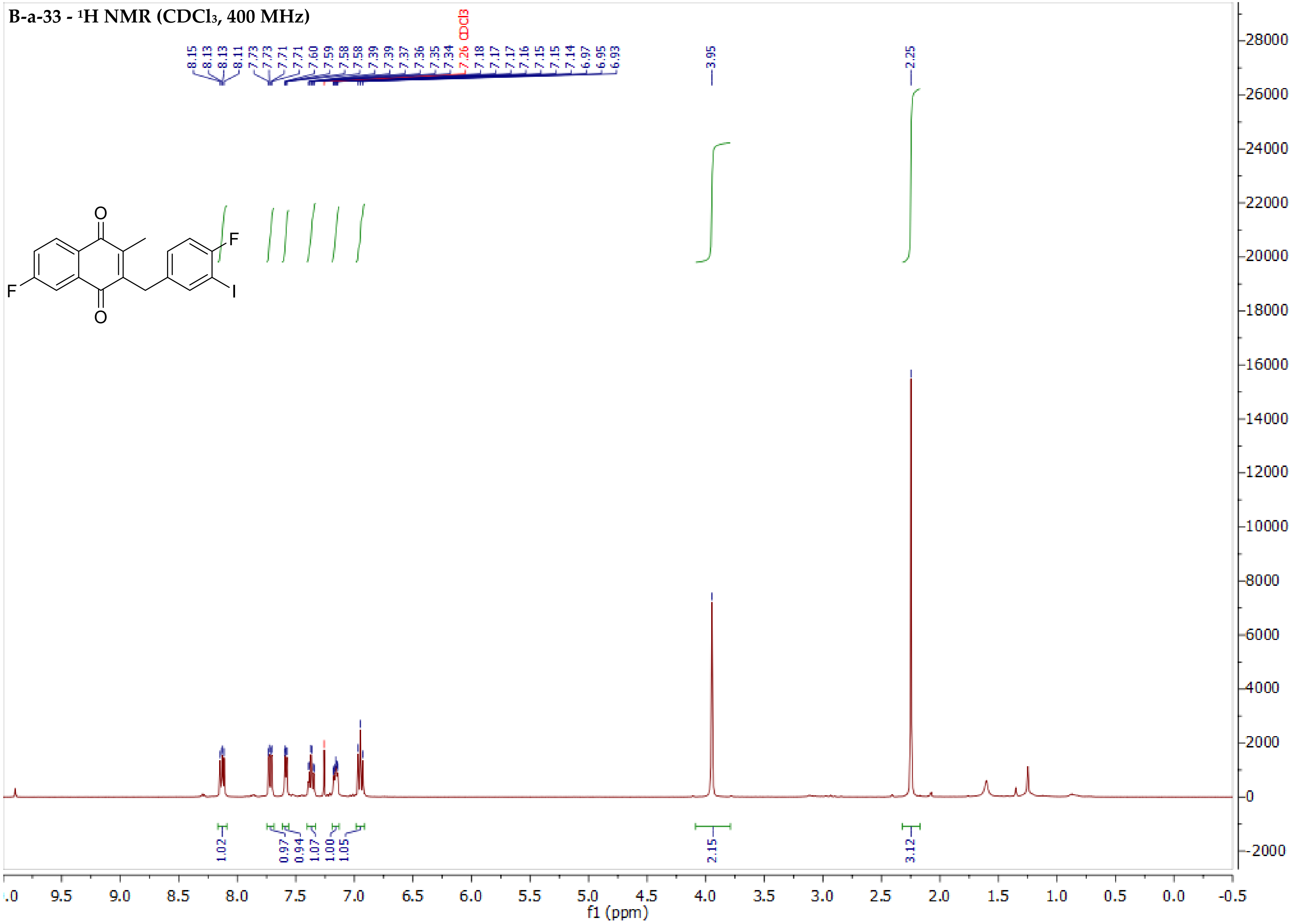

B-a-33 - $^{13}C\{^{1}H\}$ NMR ($CDCl_3$, 101 MHz)

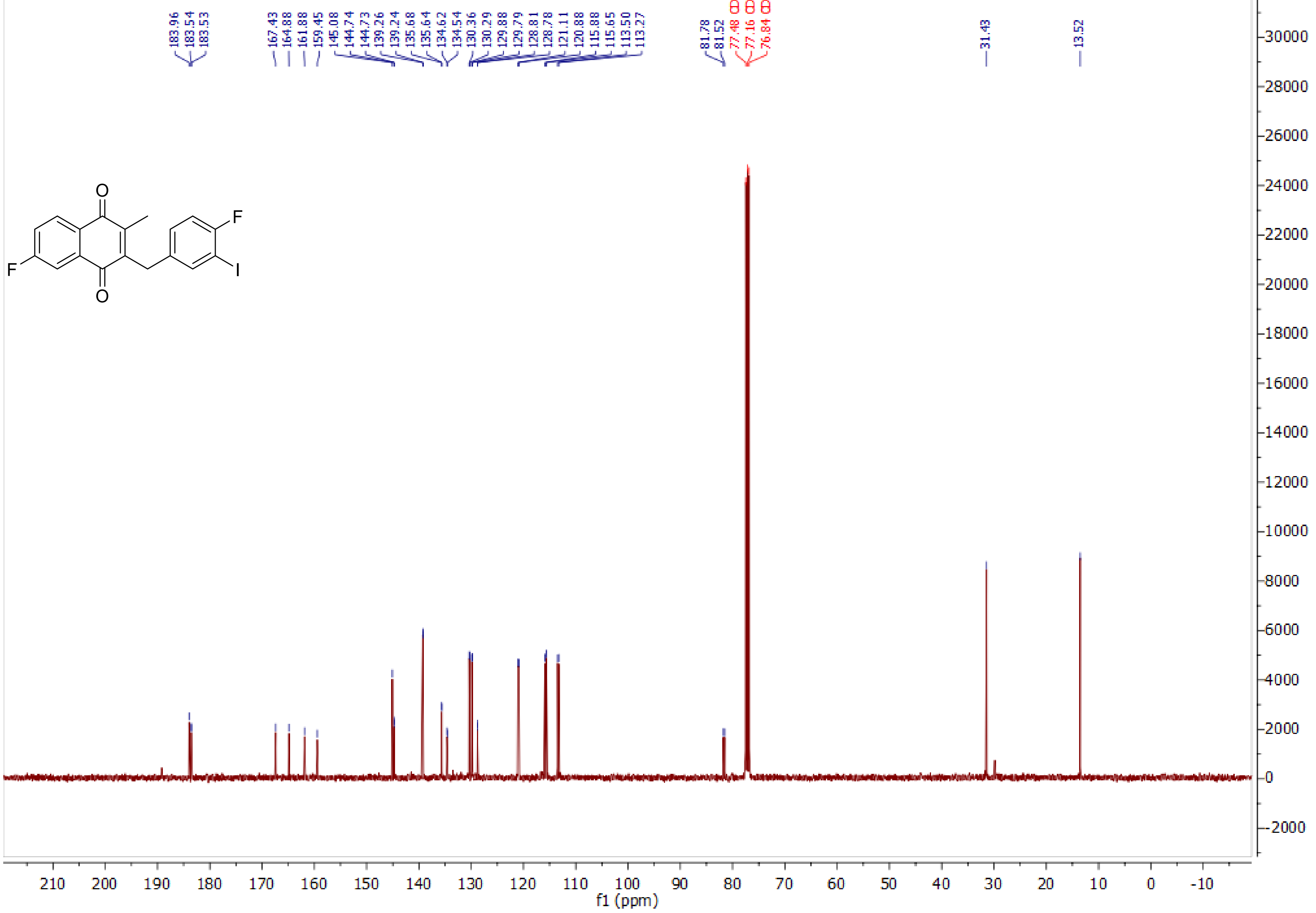

B-a-33 - ^{19}F NMR ($CDCl_3$, 377 MHz)

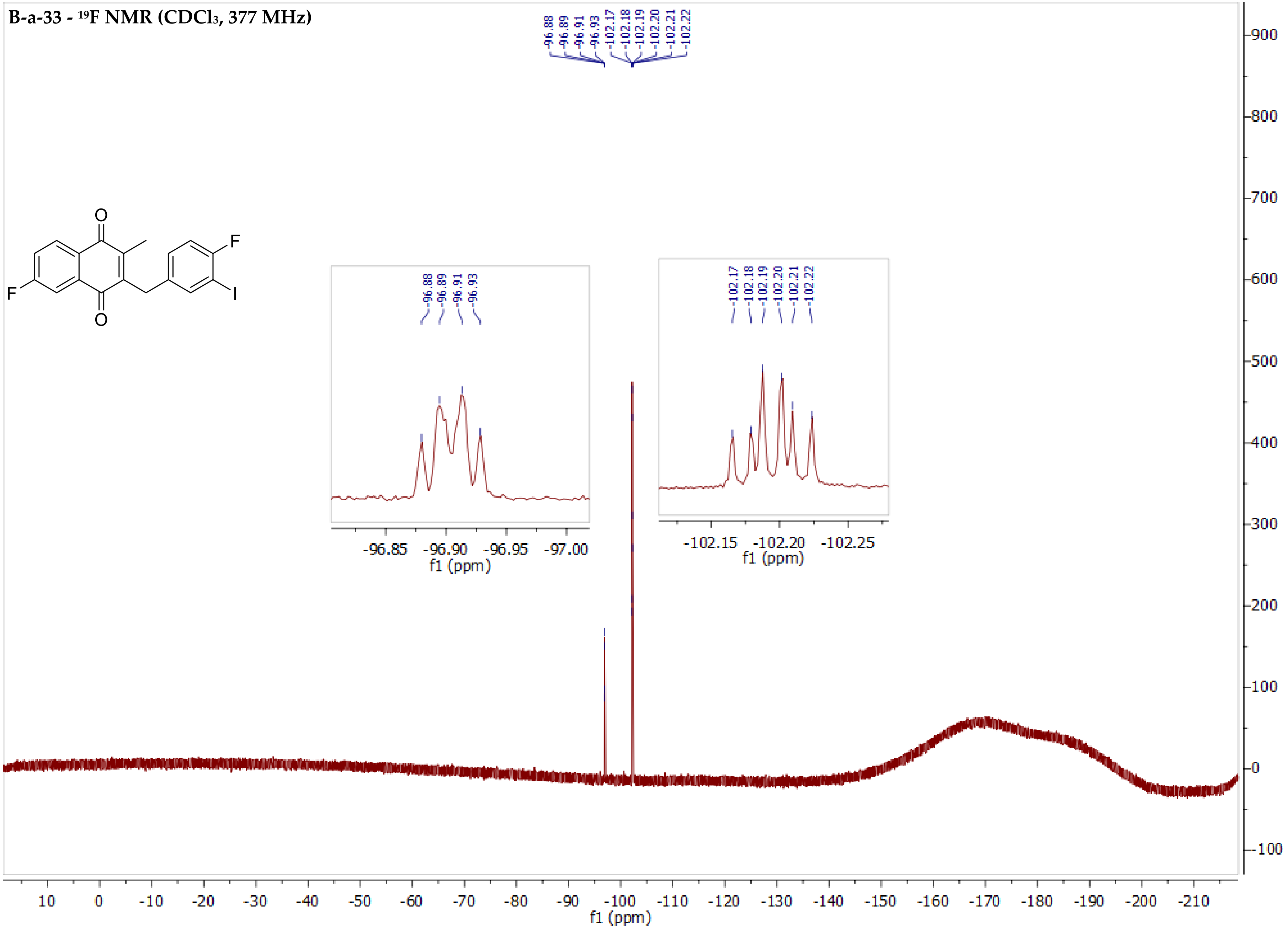

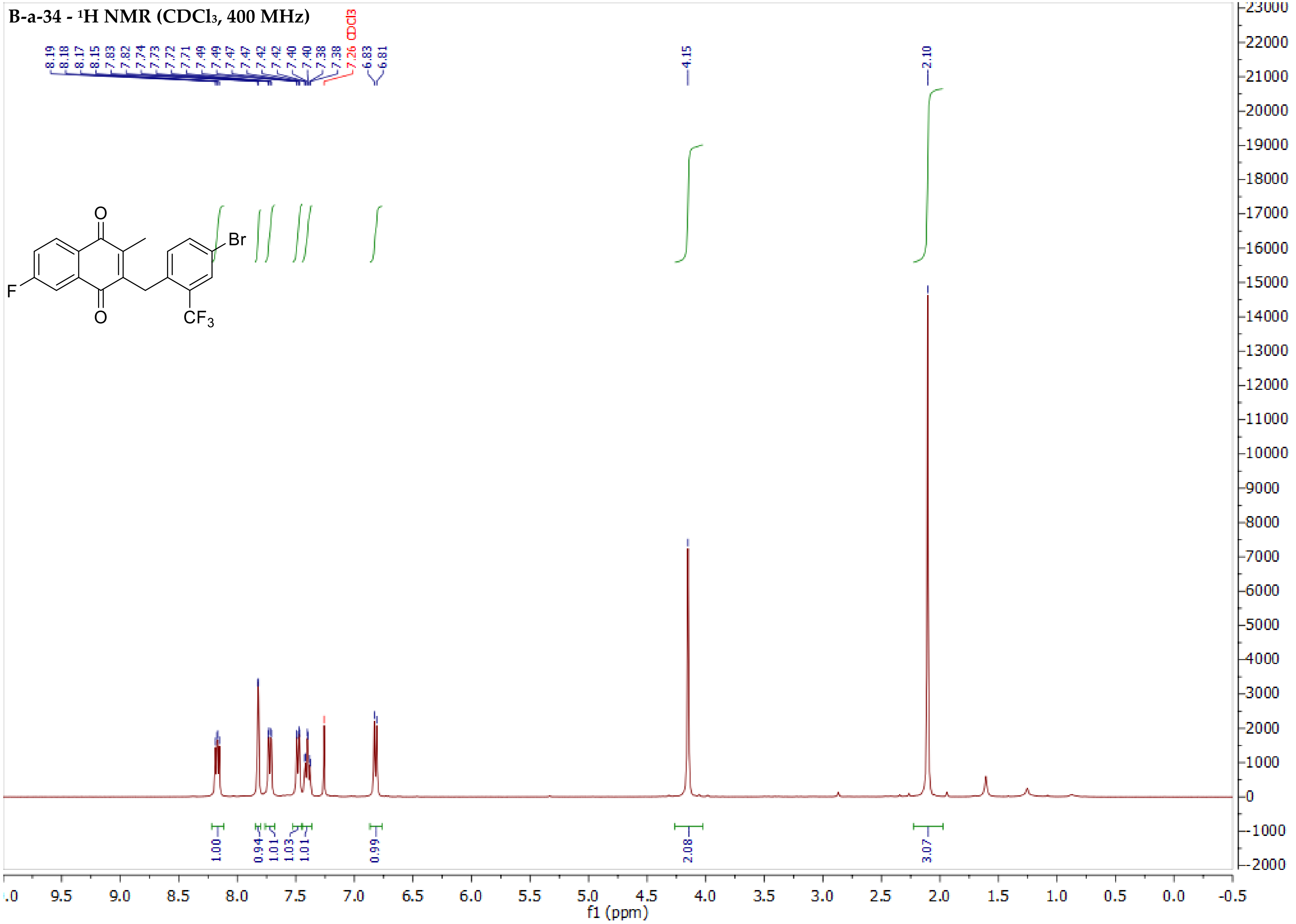
B-a-34 - ¹H NMR (CDCl₃, 400 MHz)
8.19
8.18
8.17
8.15
7.83
7.82
7.74
7.73
7.72
7.71
7.49
7.49
7.47
7.47
7.42
7.42
7.40
7.40
7.38
7.38
7.26 CDCl3
6.83
6.81
4.15
2.10
O
O
F
Br
CF3
1.00
0.94
1.01
1.03
1.01
0.99
2.08
3.07
f1 (ppm)

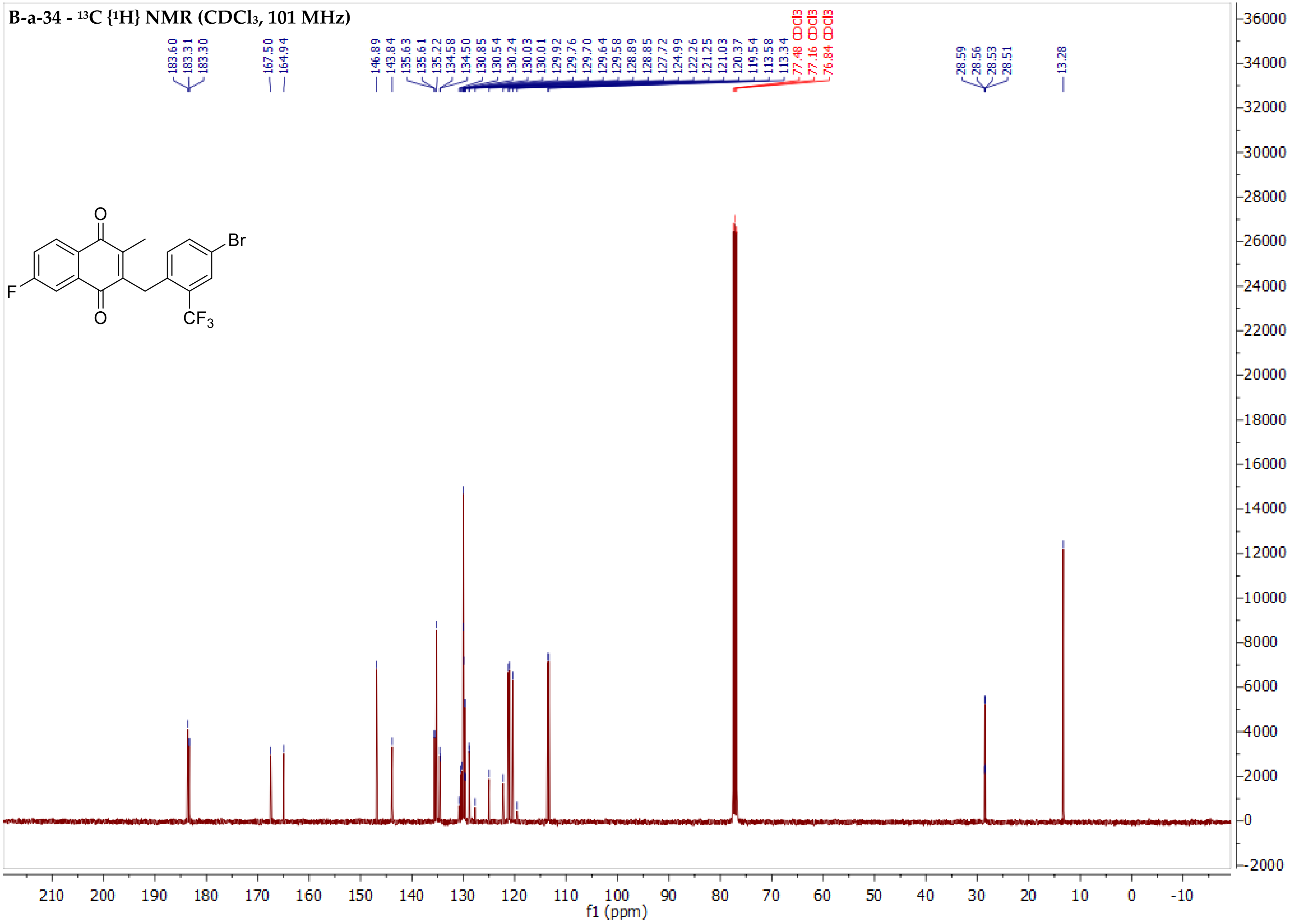
B-a-34 - $^{13}C\{^1H\}$ NMR ($CDCl_3$, 101 MHz)
183.60
183.31
183.30
167.50
164.94
146.89
143.84
135.63
135.61
135.22
134.58
134.50
130.85
130.54
130.24
130.03
130.01
129.92
129.76
129.70
129.64
129.58
128.89
128.85
127.72
124.99
122.26
121.25
121.03
120.37
119.54
113.58
113.34
77.48 CDCl3
77.16 CDCl3
76.84 CDCl3
28.59
28.56
28.53
28.51
13.28
O
O
F
Br
CF_3
f1 (ppm)

B-a-34 - ^{19}F NMR ($CDCl_3$, 377 MHz)

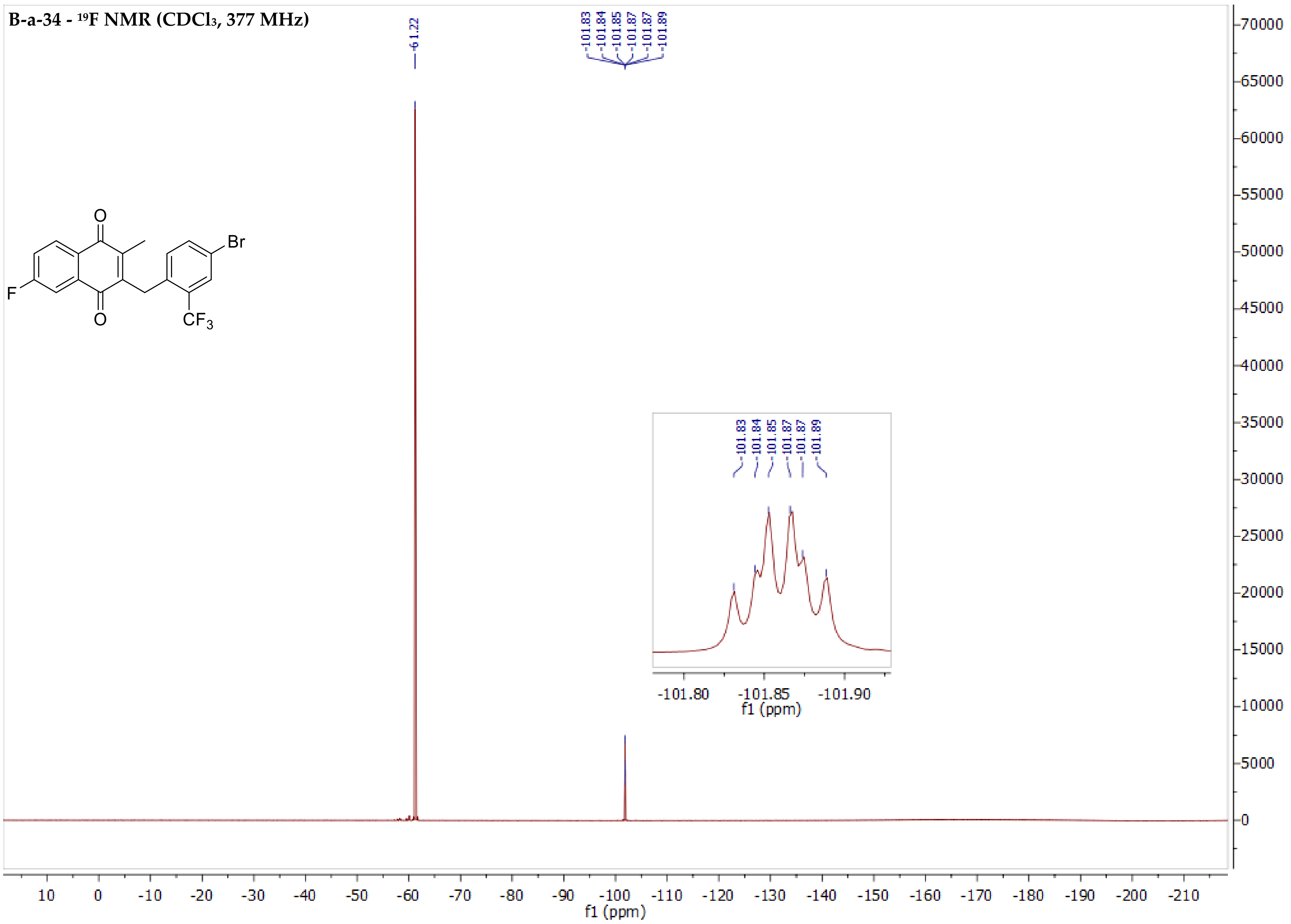

B-a-35 - ^{1}H NMR (CDCl$_3$, 400 MHz)

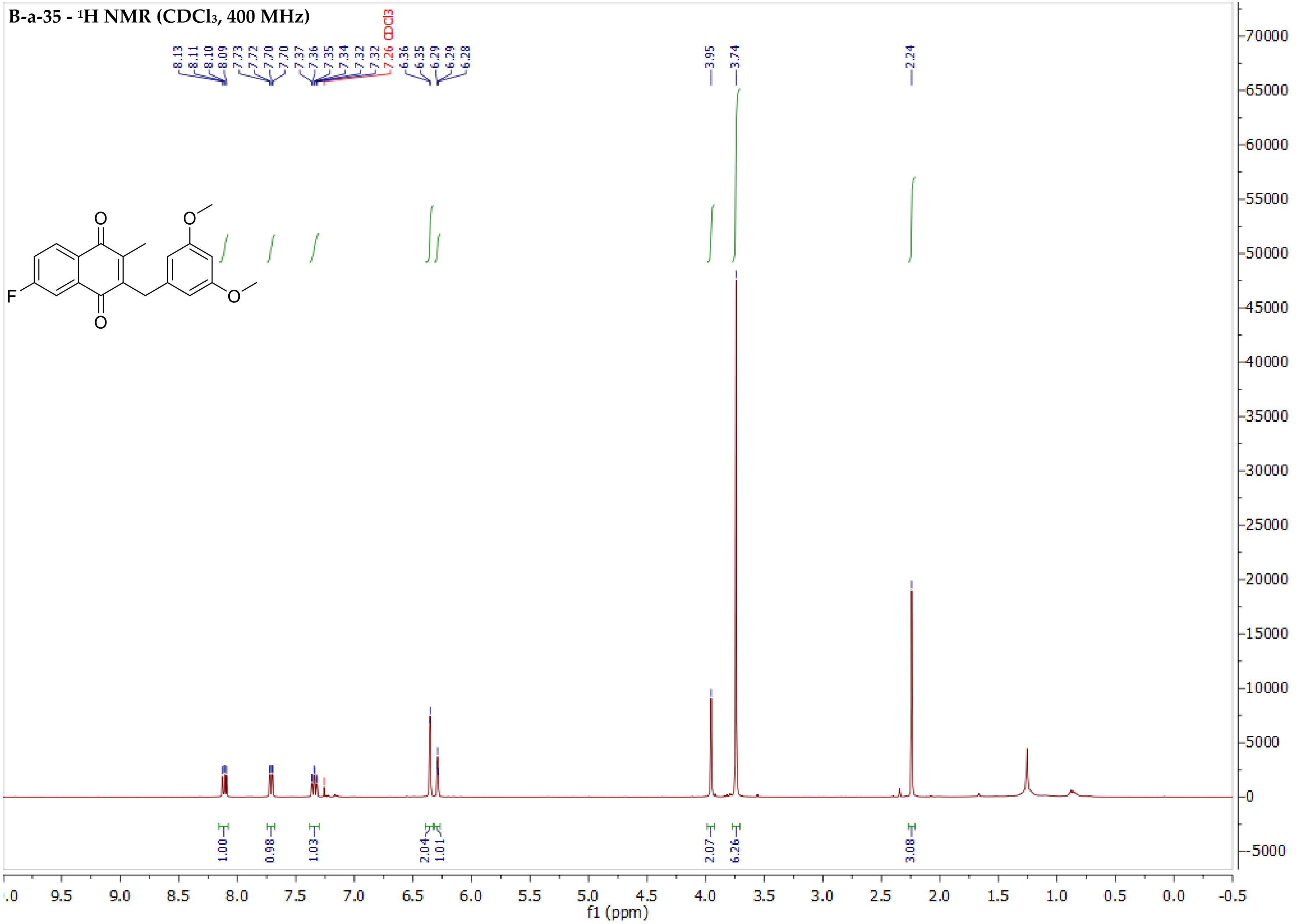

B-a-35 - ¹³C {¹H} NMR (CDCl₃, 101 MHz)

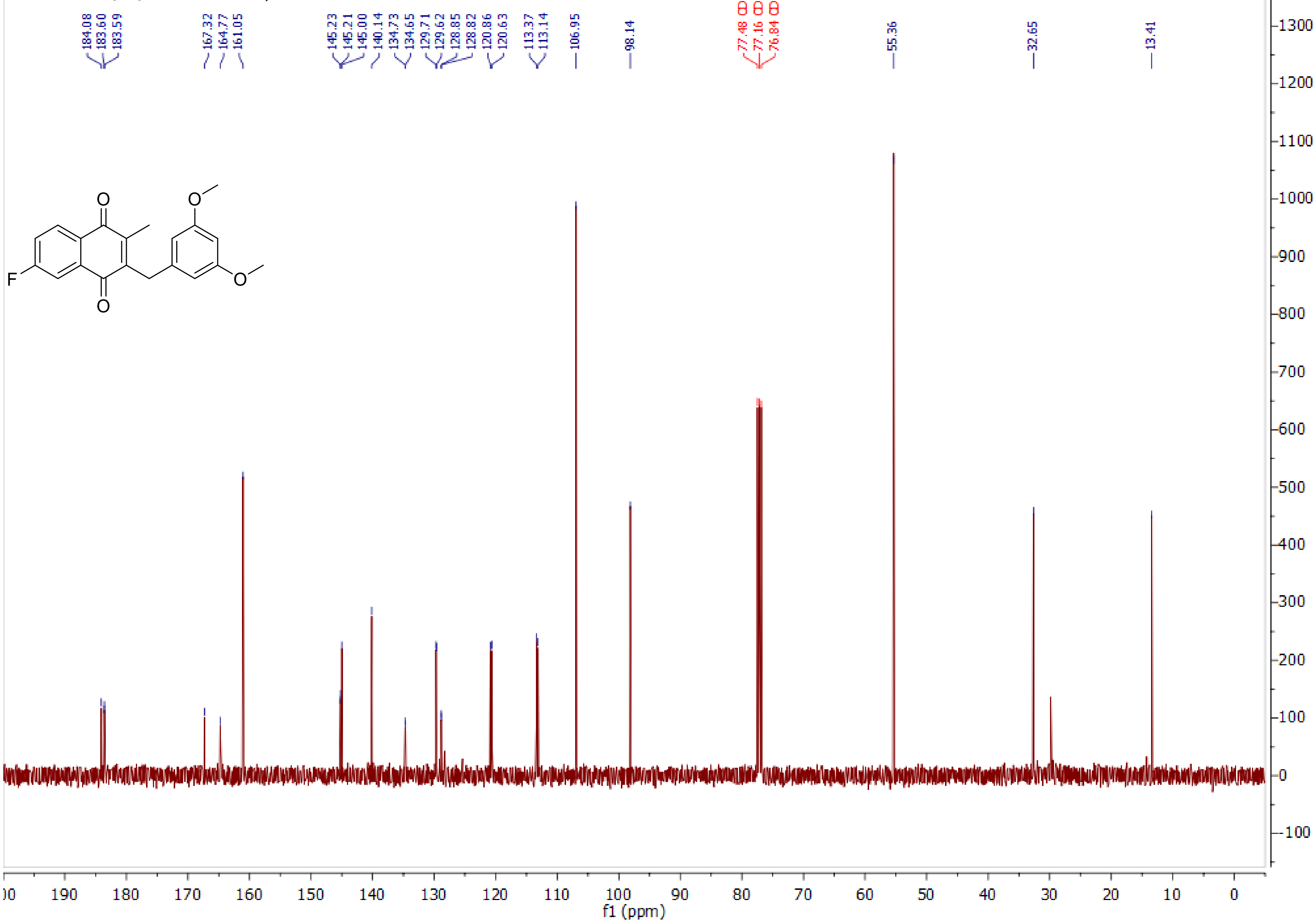

B-a-35 - ^{19}F NMR ($CDCl_3$, 377 MHz)

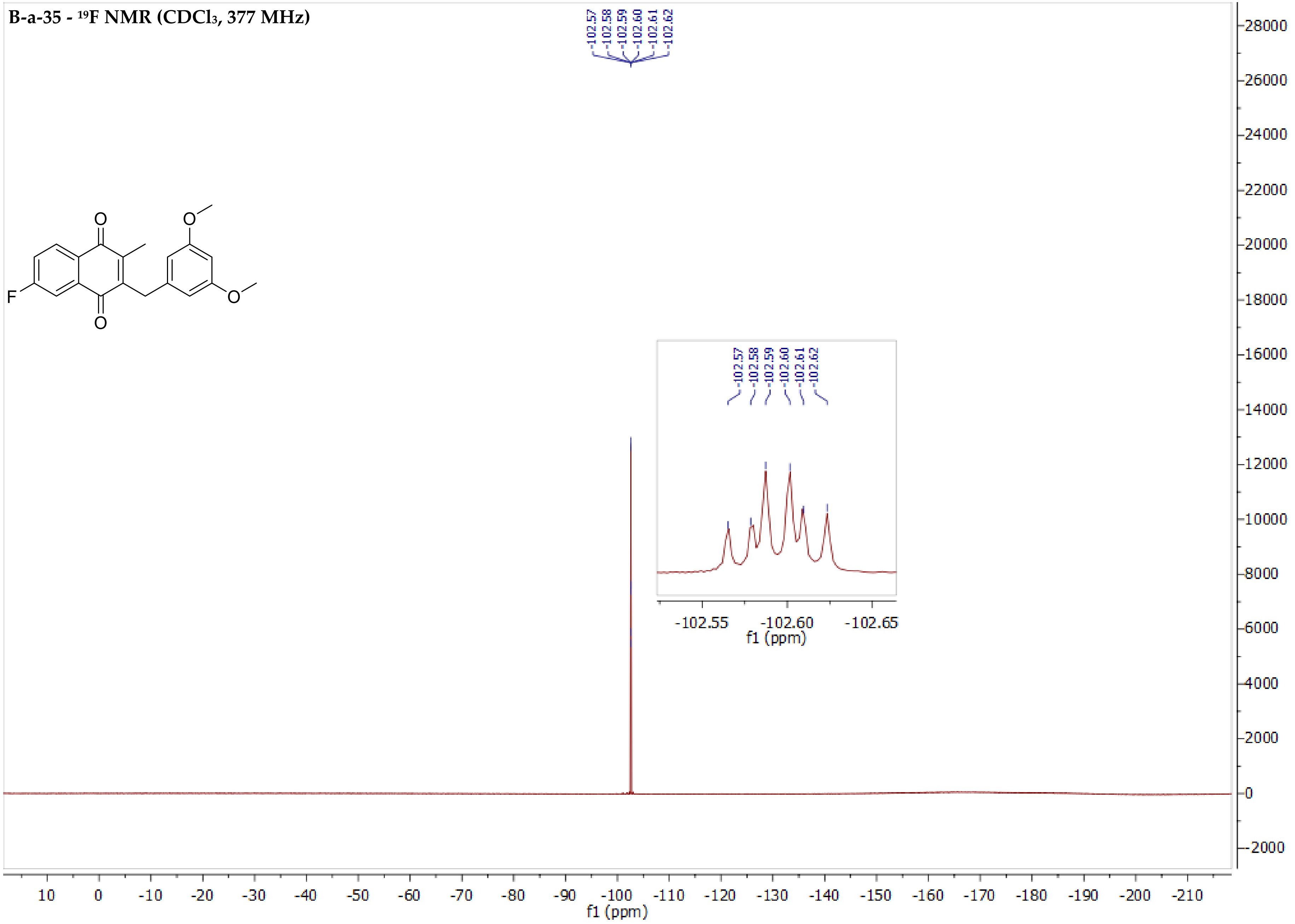

a-36 - ¹H NMR ($CDCl_3$, 400 MHz)

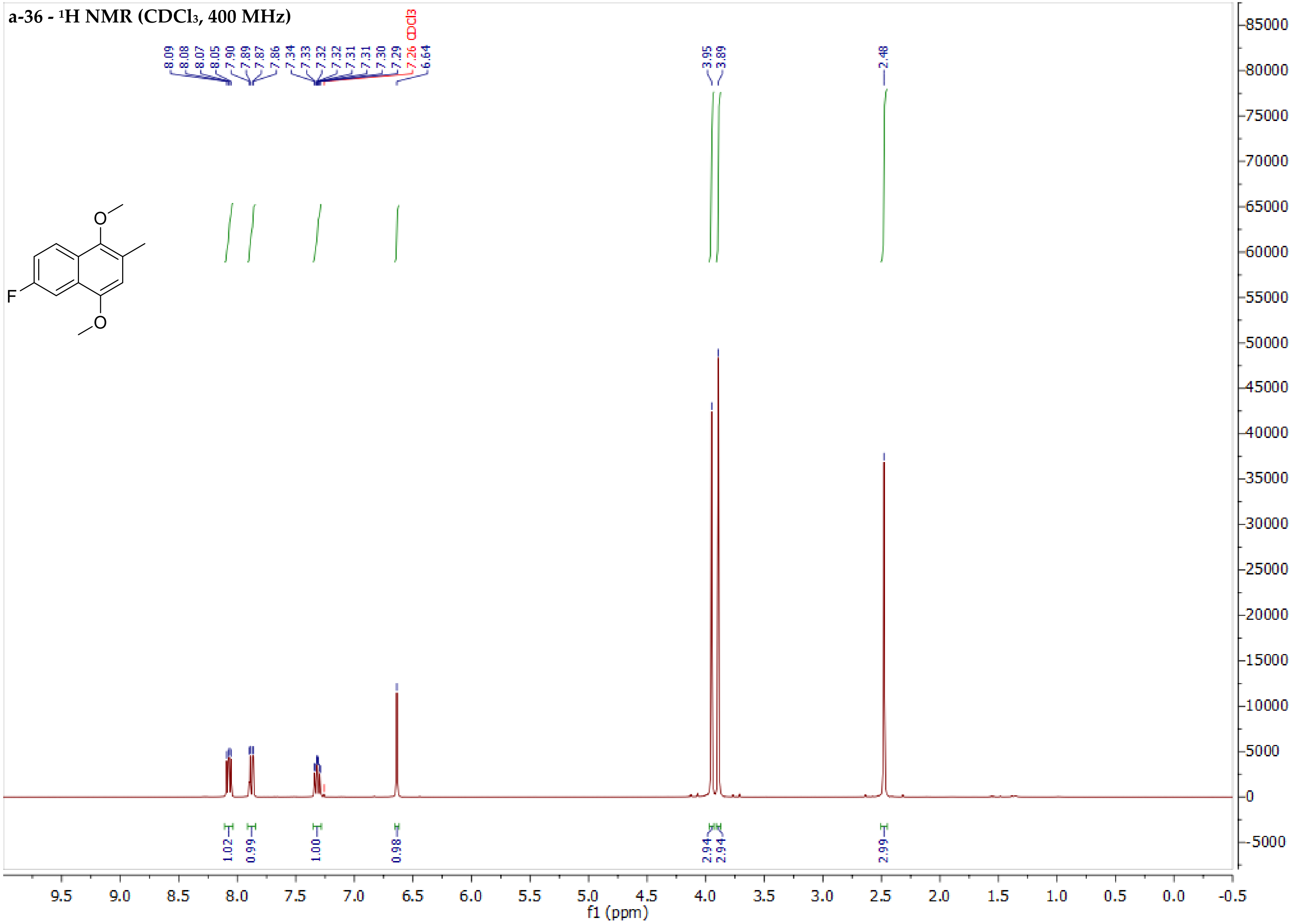

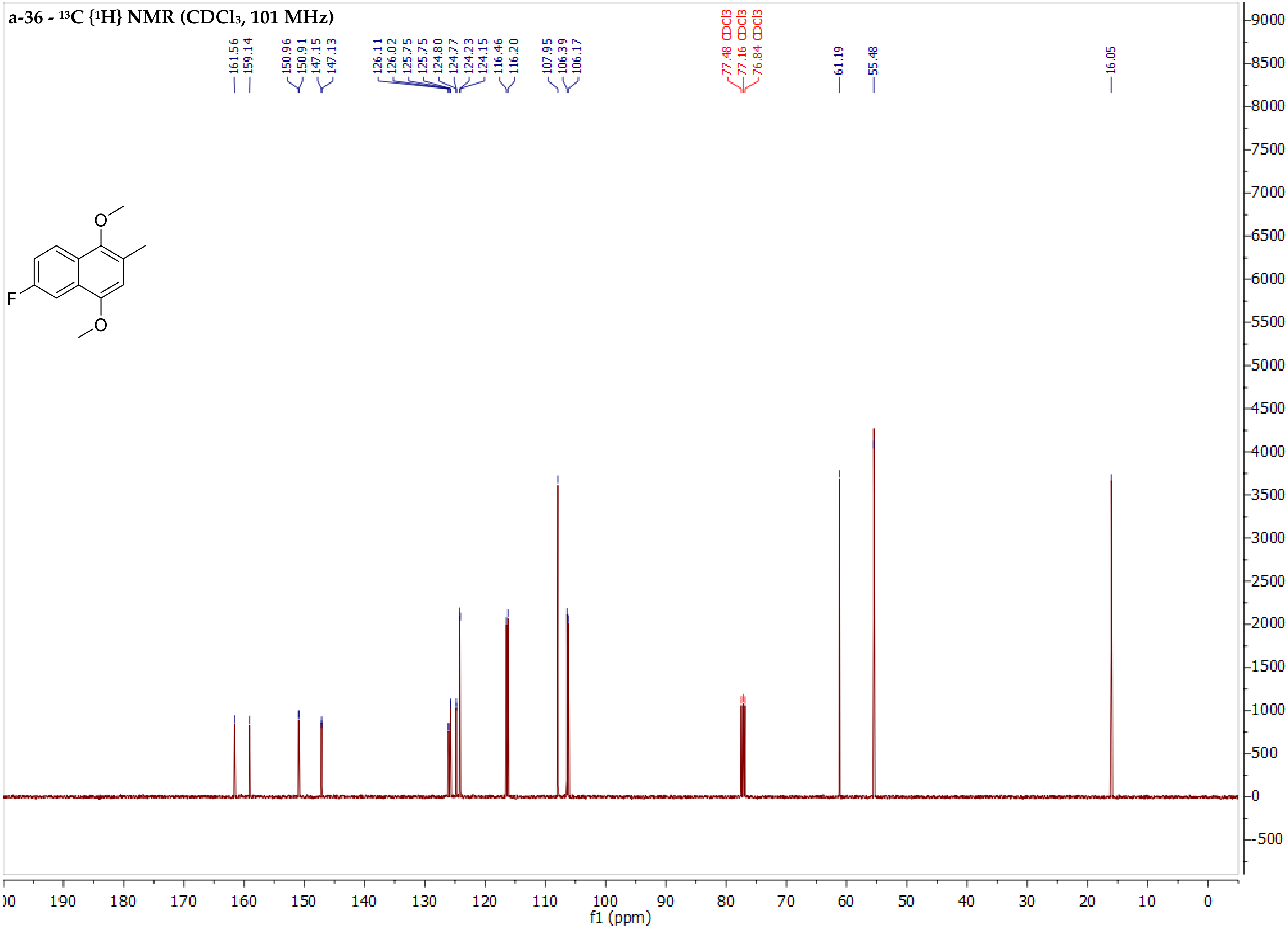
a-36 - 13C {1H} NMR (CDCl3, 101 MHz)
161.56
159.14
150.96
150.91
147.15
147.13
126.11
126.02
125.75
125.75
124.80
124.77
124.23
124.15
116.46
116.20
107.95
106.39
106.17
77.48 CDCl3
77.16 CDCl3
76.84 CDCl3
61.19
55.48
16.05
F
O
O
f1 (ppm)

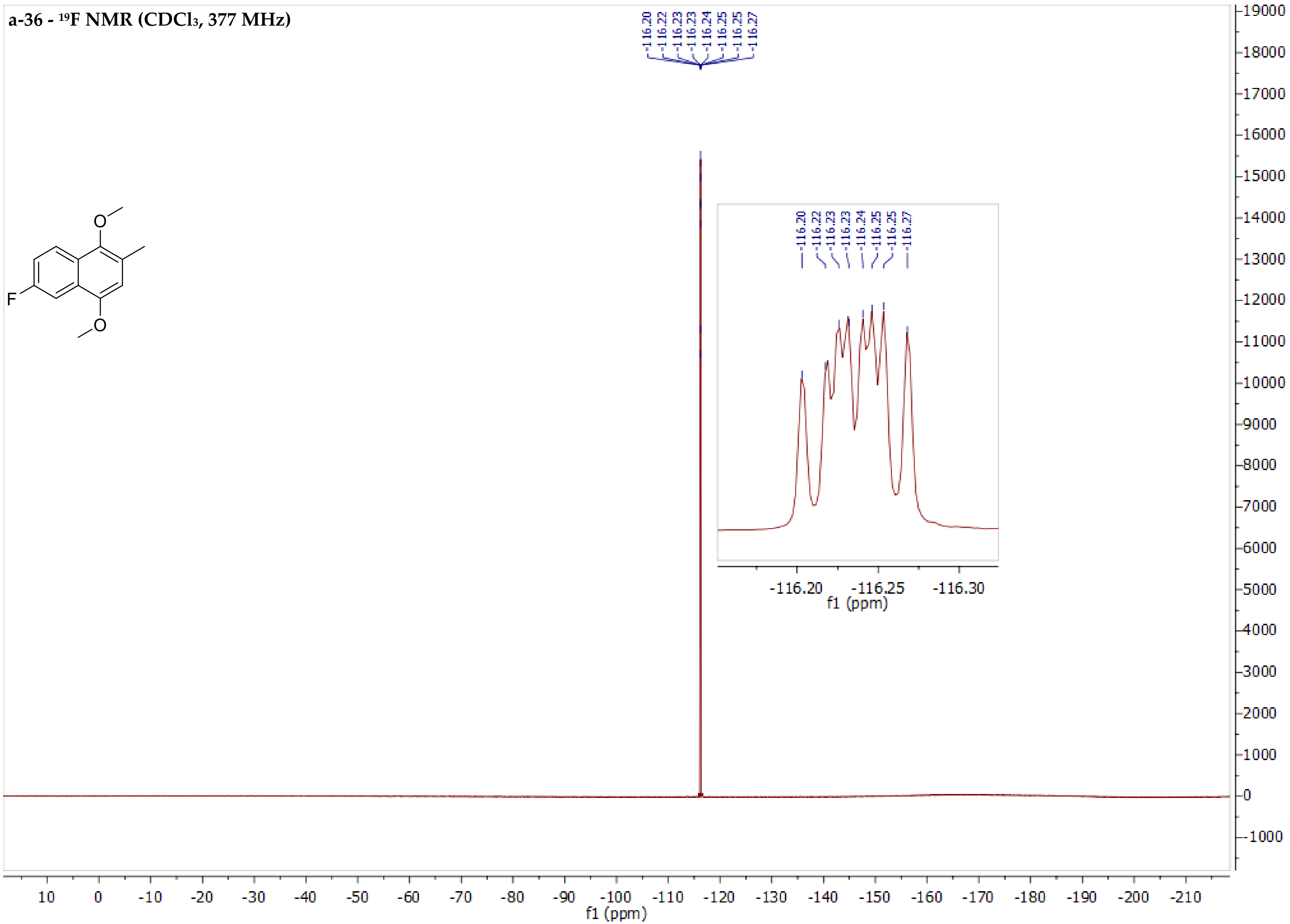
a-36 - ^{19}F NMR ($CDCl_3$, 377 MHz)
-116.20
-116.22
-116.23
-116.23
-116.24
-116.25
-116.25
-116.27
F
O
O
-116.20
-116.25
-116.30
f1 (ppm)
10 0 -10 -20 -30 -40 -50 -60 -70 -80 -90 -100 -110 -120 -130 -140 -150 -160 -170 -180 -190 -200 -210
f1 (ppm)
19000 18000 17000 16000 15000 14000 13000 12000 11000 10000 9000 8000 7000 6000 5000 4000 3000 2000 1000 0 -1000

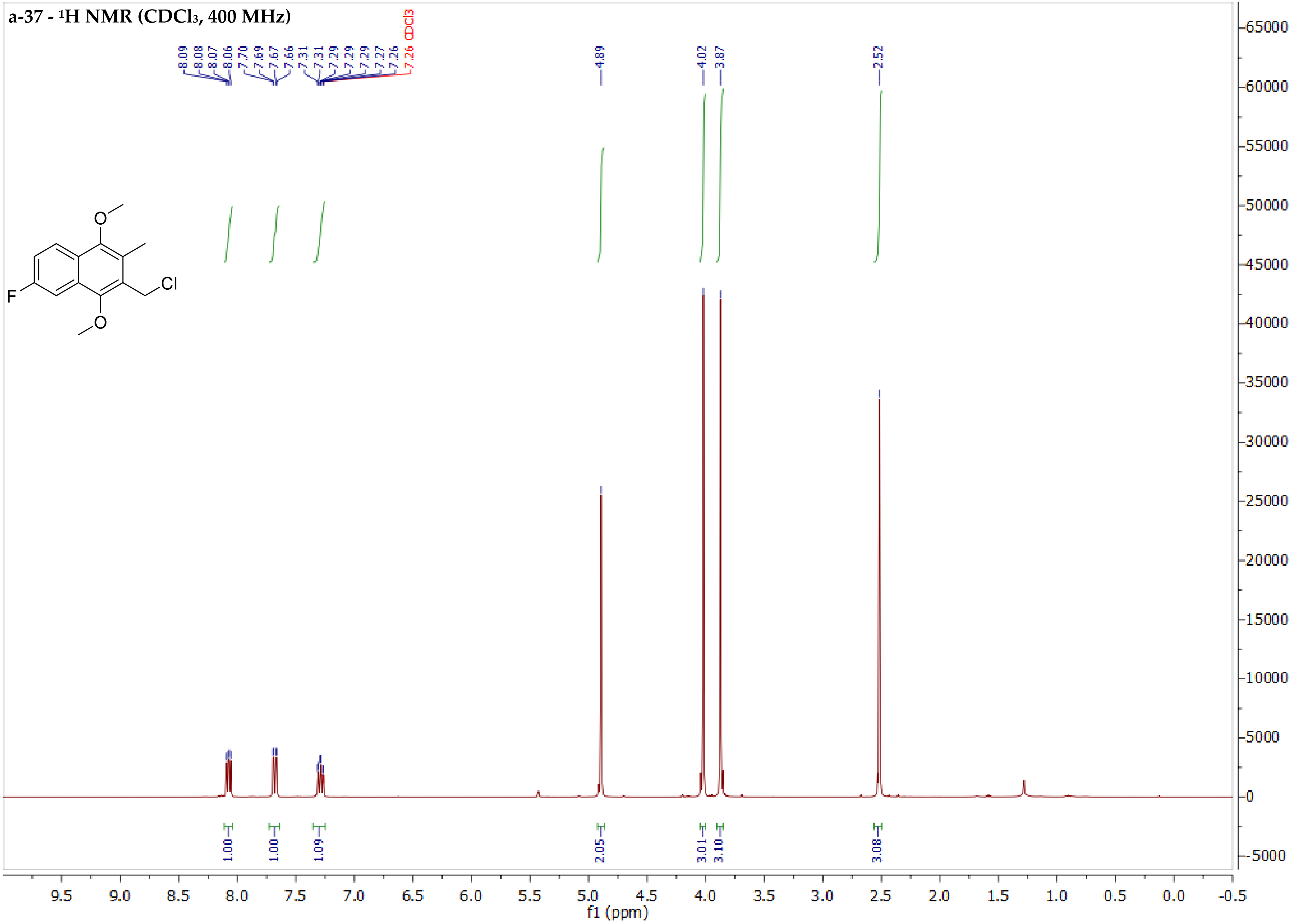
a-37 - ¹H NMR (CDCl₃, 400 MHz)
8.09
8.08
8.07
8.06
7.70
7.69
7.67
7.66
7.31
7.31
7.29
7.29
7.29
7.27
7.26
7.26 CDCl3
4.89
4.02
3.87
2.52
F
Cl
O
O
1.00
1.00
1.09
2.05
3.01
3.10
3.08
f1 (ppm)

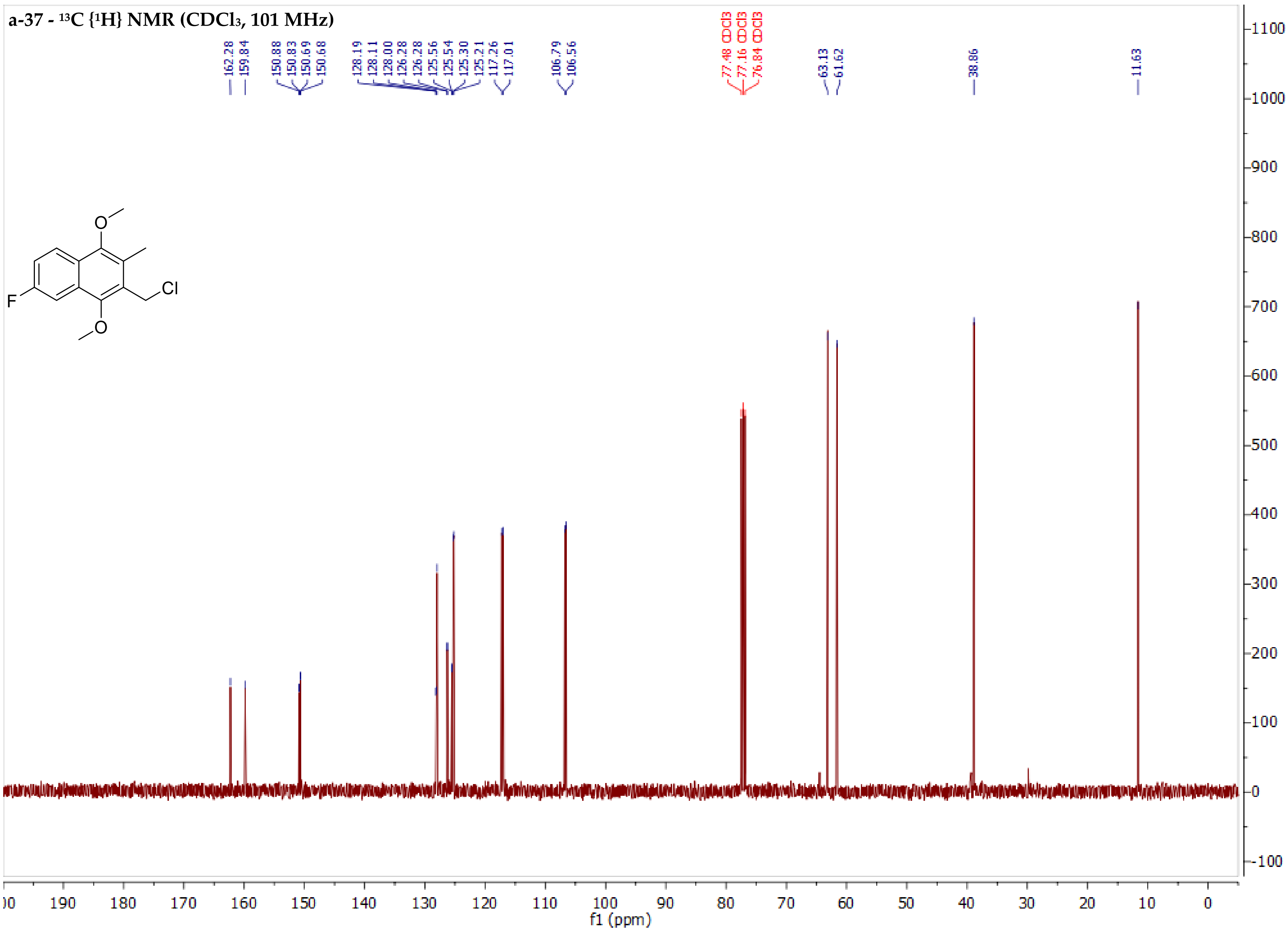

a-37 - 13C {1H} NMR (CDCl3, 101 MHz)
162.28
159.84
150.88
150.83
150.69
150.68
128.19
128.11
128.00
126.28
126.28
125.56
125.54
125.30
125.21
117.26
117.01
106.79
106.56
77.48 CDCl3
77.16 CDCl3
76.84 CDCl3
63.13
61.62
38.86
11.63
F
Cl
O
O
f1 (ppm)

a-37 - 19F NMR (CDCl3, 377 MHz)

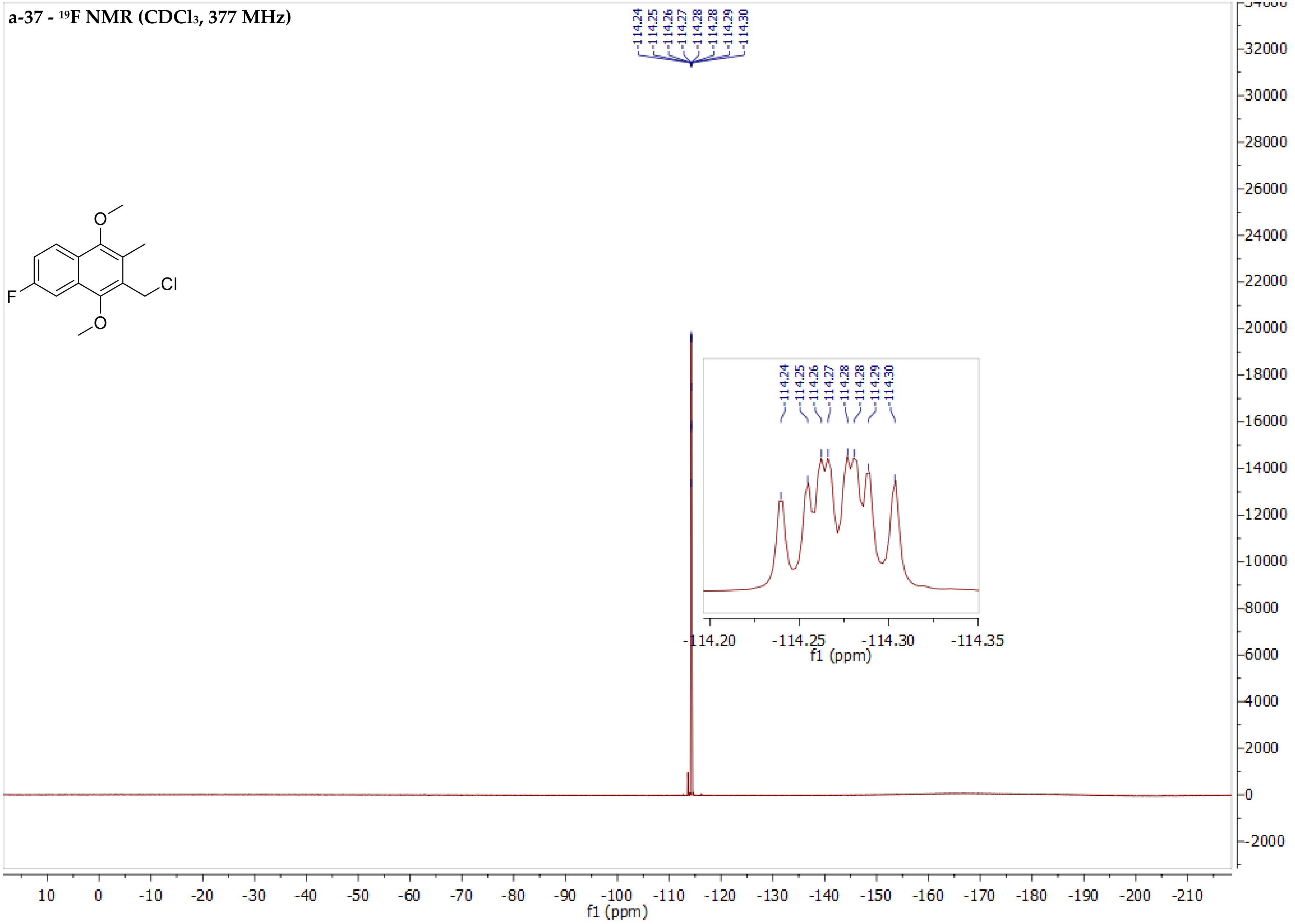

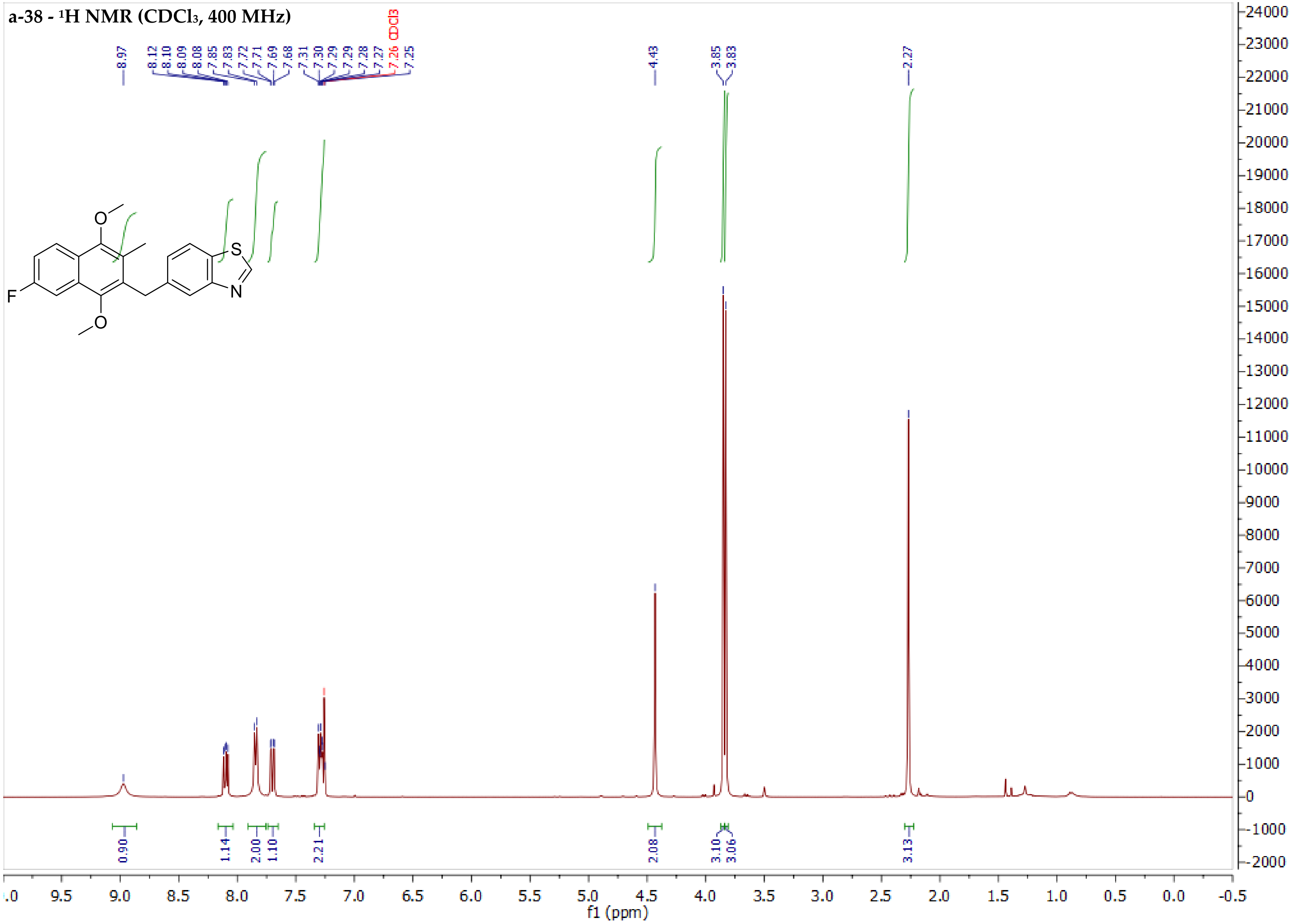
a-38 - 1H NMR (CDCl3, 400 MHz)
8.97
8.12 8.10 8.09 8.08 7.85 7.83 7.72 7.71 7.69 7.68 7.31 7.30 7.29 7.29 7.28 7.27 7.26 7.25
CDCl3
4.43
3.85 3.83
2.27
0.90 1.14 2.00 1.10 2.21 2.08 3.10 3.06 3.13
f1 (ppm)

a-38 - ¹³C {¹H} NMR (CDCl₃, 101 MHz)

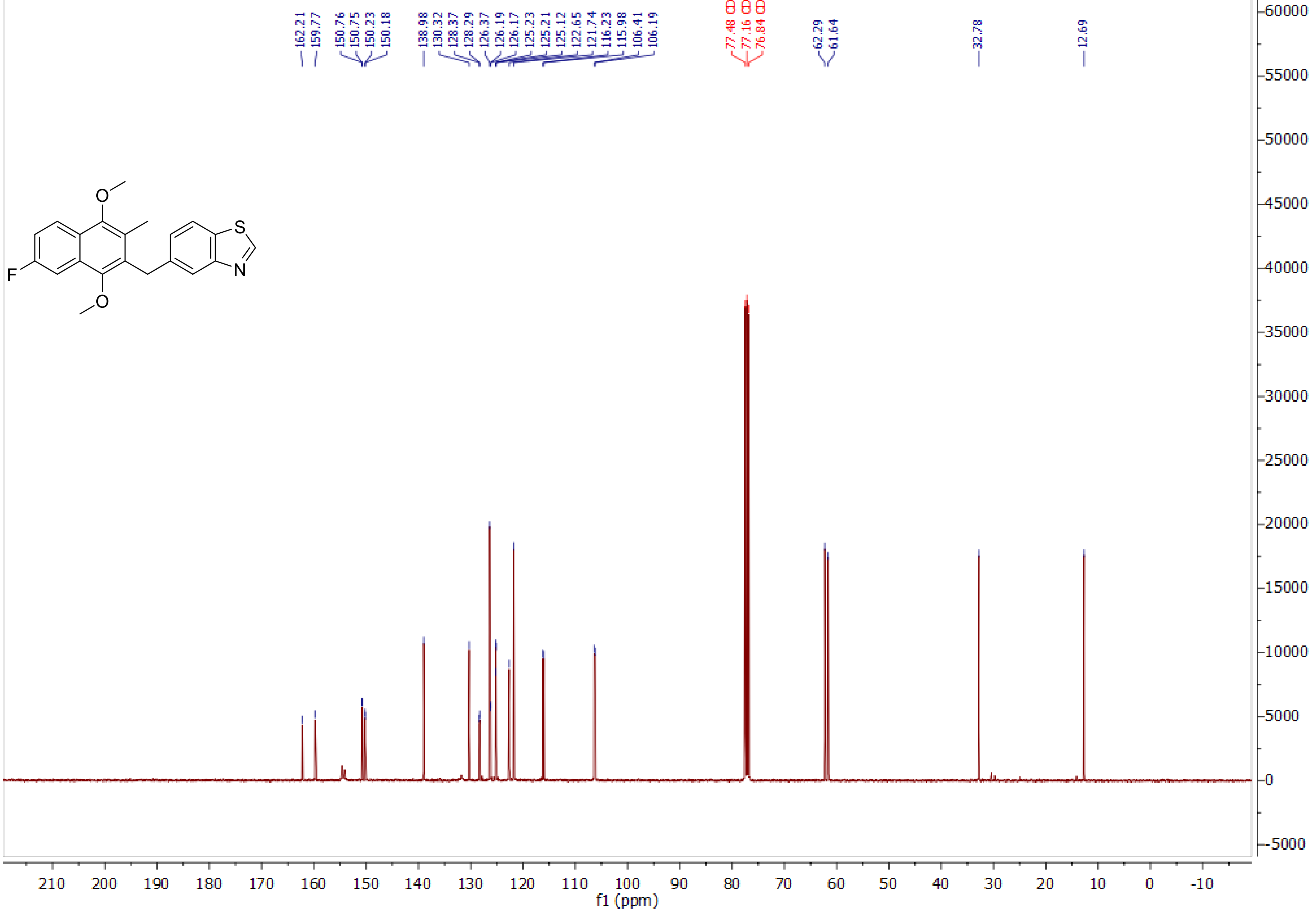

a-38 - ^{19}F NMR (CDCl$_3$, 377 MHz)

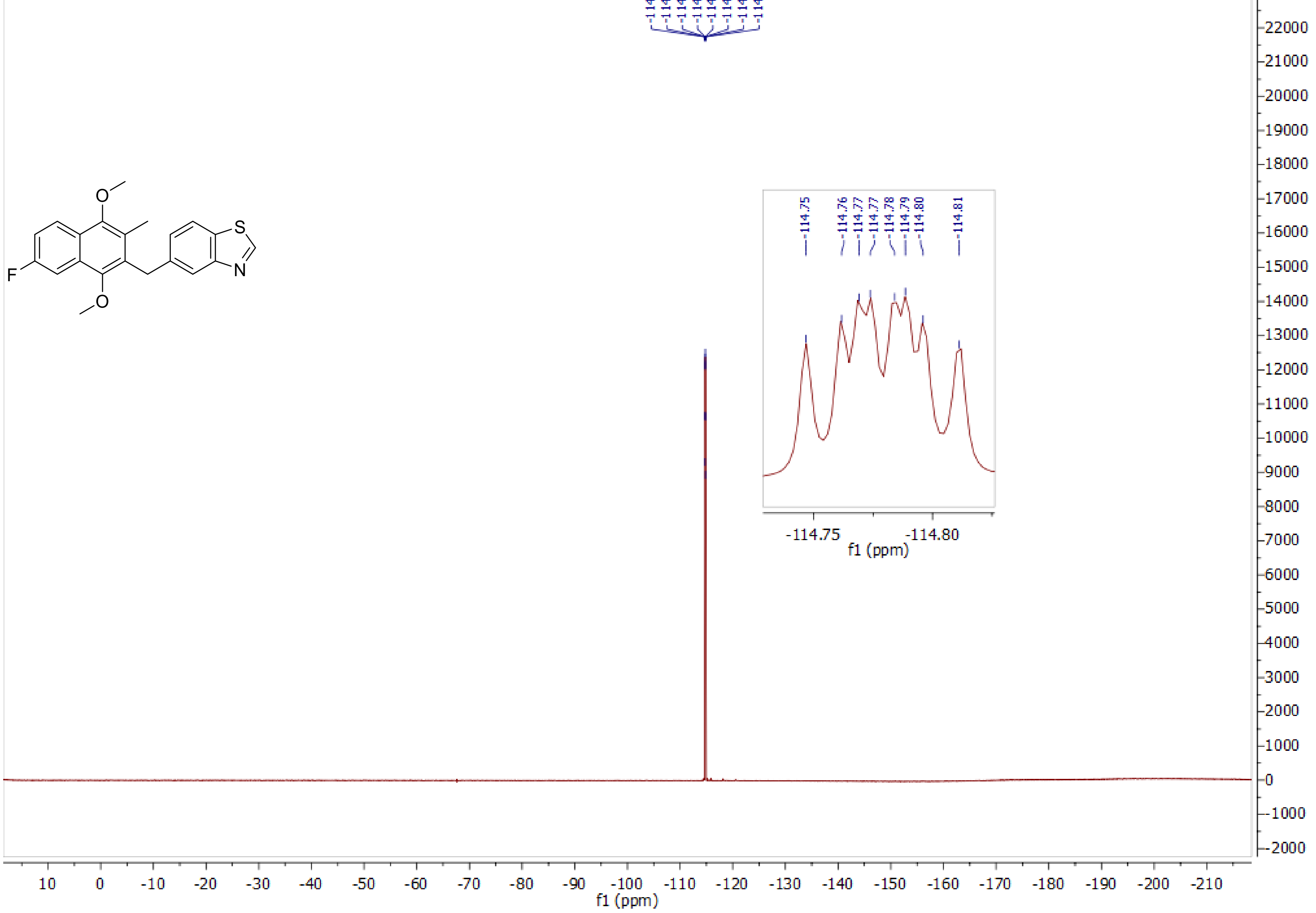

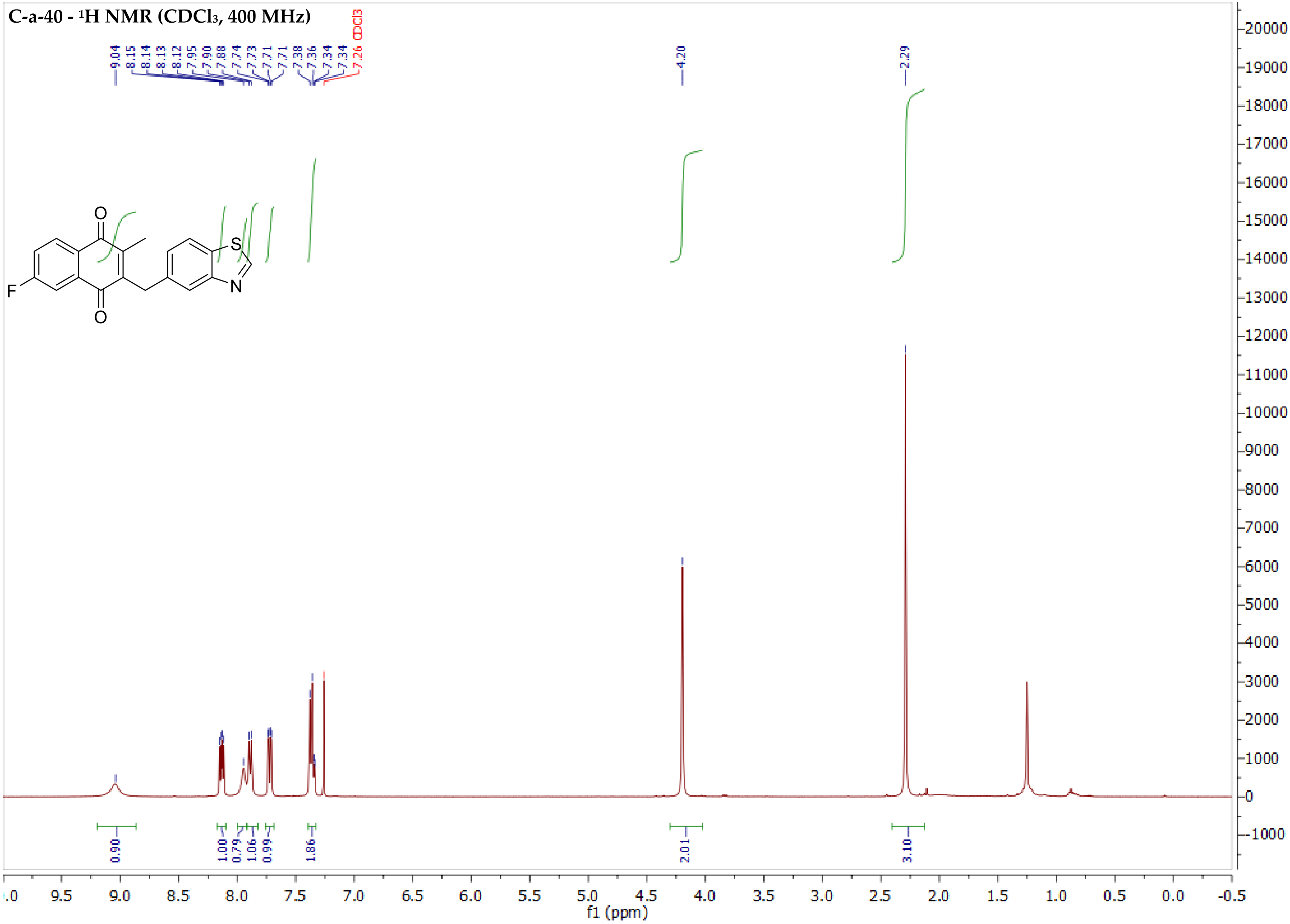
C-a-40 - 1H NMR (CDCl3, 400 MHz)
9.04
8.15
8.14
8.13
8.12
7.95
7.90
7.88
7.74
7.73
7.71
7.71
7.38
7.36
7.34
7.34
7.26 CDCl3
4.20
2.29
O
O
F
S
N
0.90
1.00
0.79
1.06
0.99
1.86
2.01
3.10
f1 (ppm)

C-a-40 - $^{13}C\{^{1}H\}$ NMR ($CDCl_3$, 101 MHz)

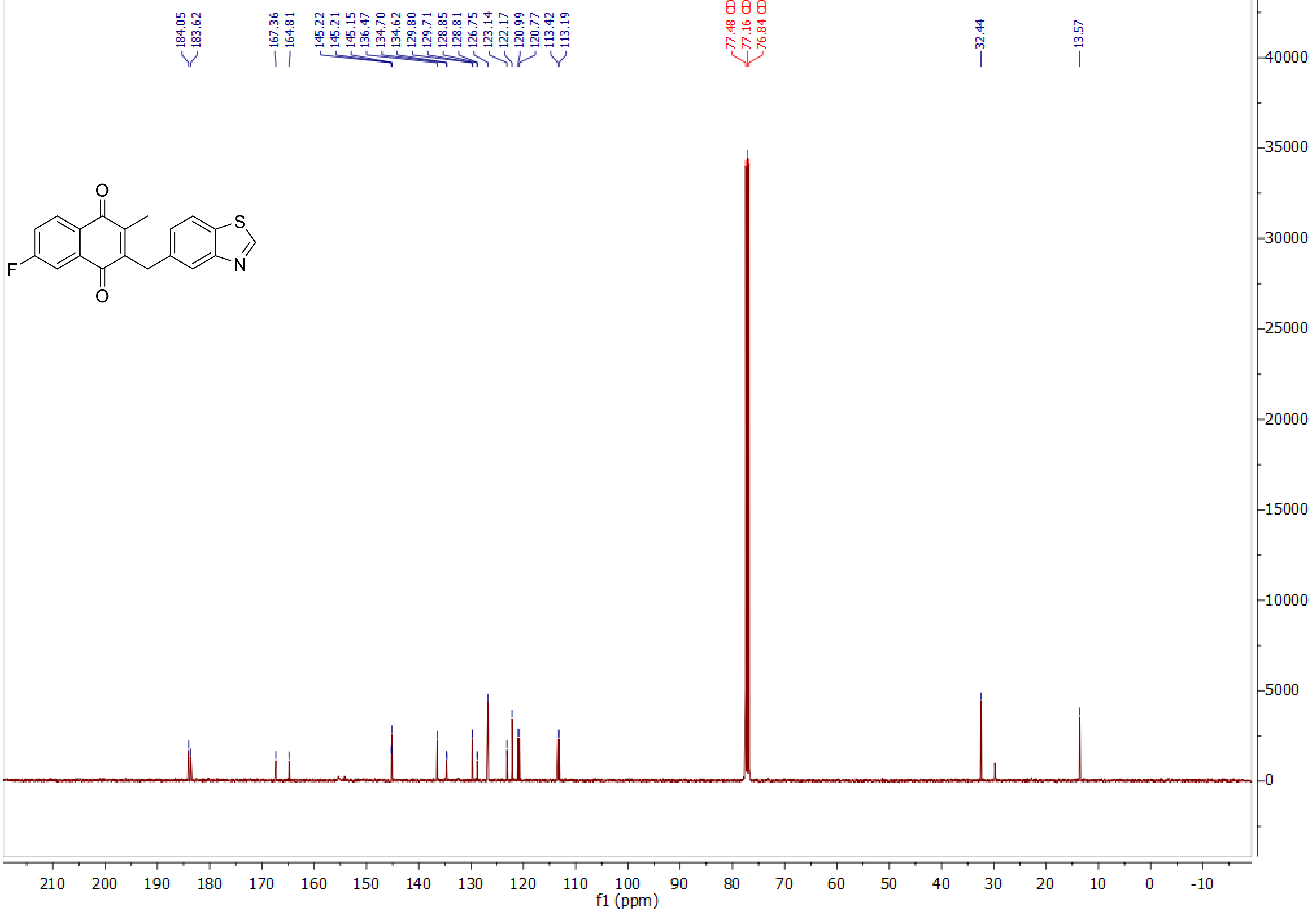

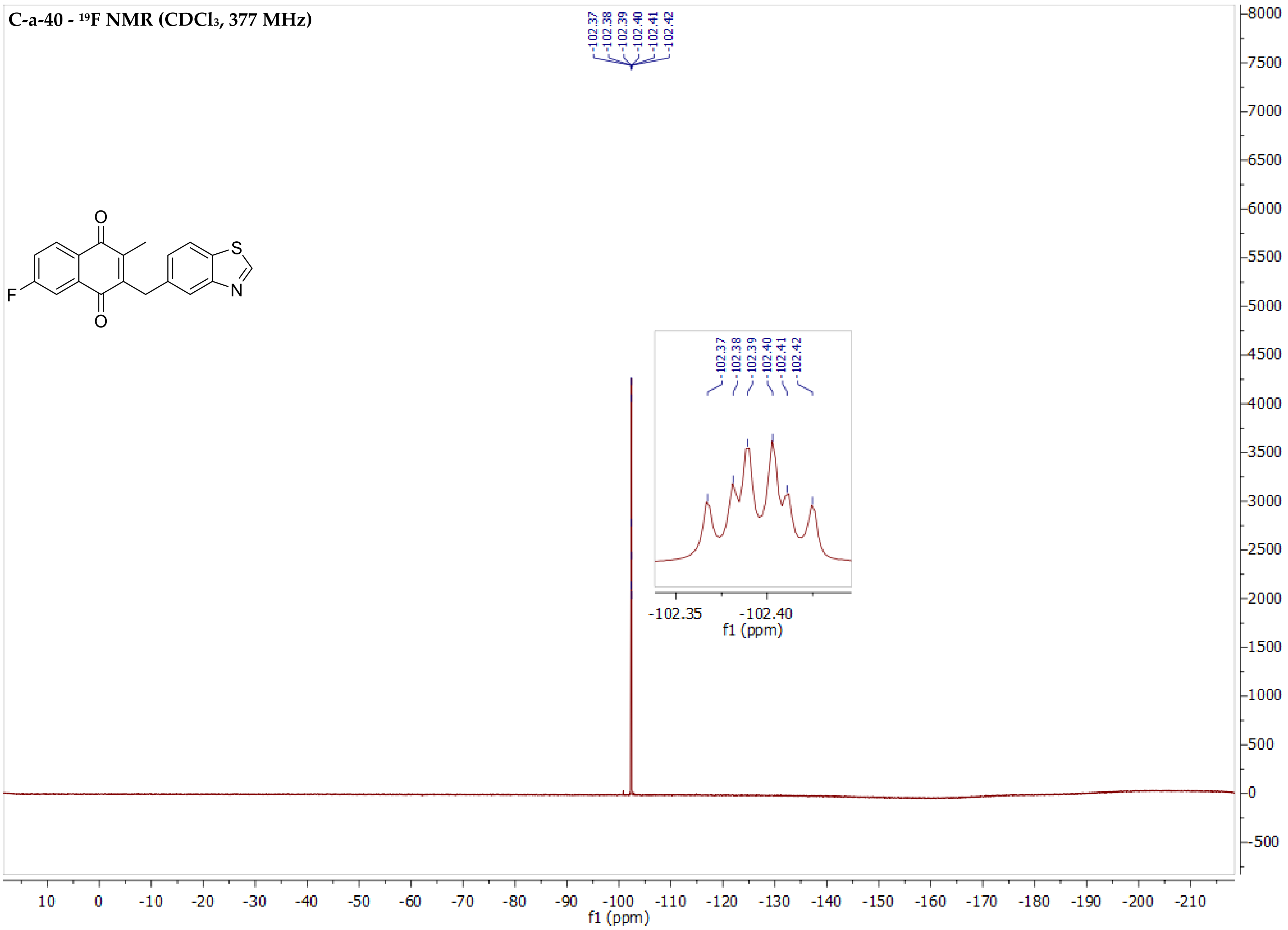
C-a-40 - ¹⁹F NMR (CDCl3, 377 MHz)
-102.37
-102.38
-102.39
-102.40
-102.41
-102.42
O
O
F
S
N
-102.35
-102.40
f1 (ppm)
8000
7500
7000
6500
6000
5500
5000
4500
4000
3500
3000
2500
2000
1500
1000
500
0
-500
10
0
-10
-20
-30
-40
-50
-60
-70
-80
-90
-100
-110
-120
-130
-140
-150
-160
-170
-180
-190
-200
-210
f1 (ppm)

a-39 - ¹H NMR (CDCl₃, 400 MHz)

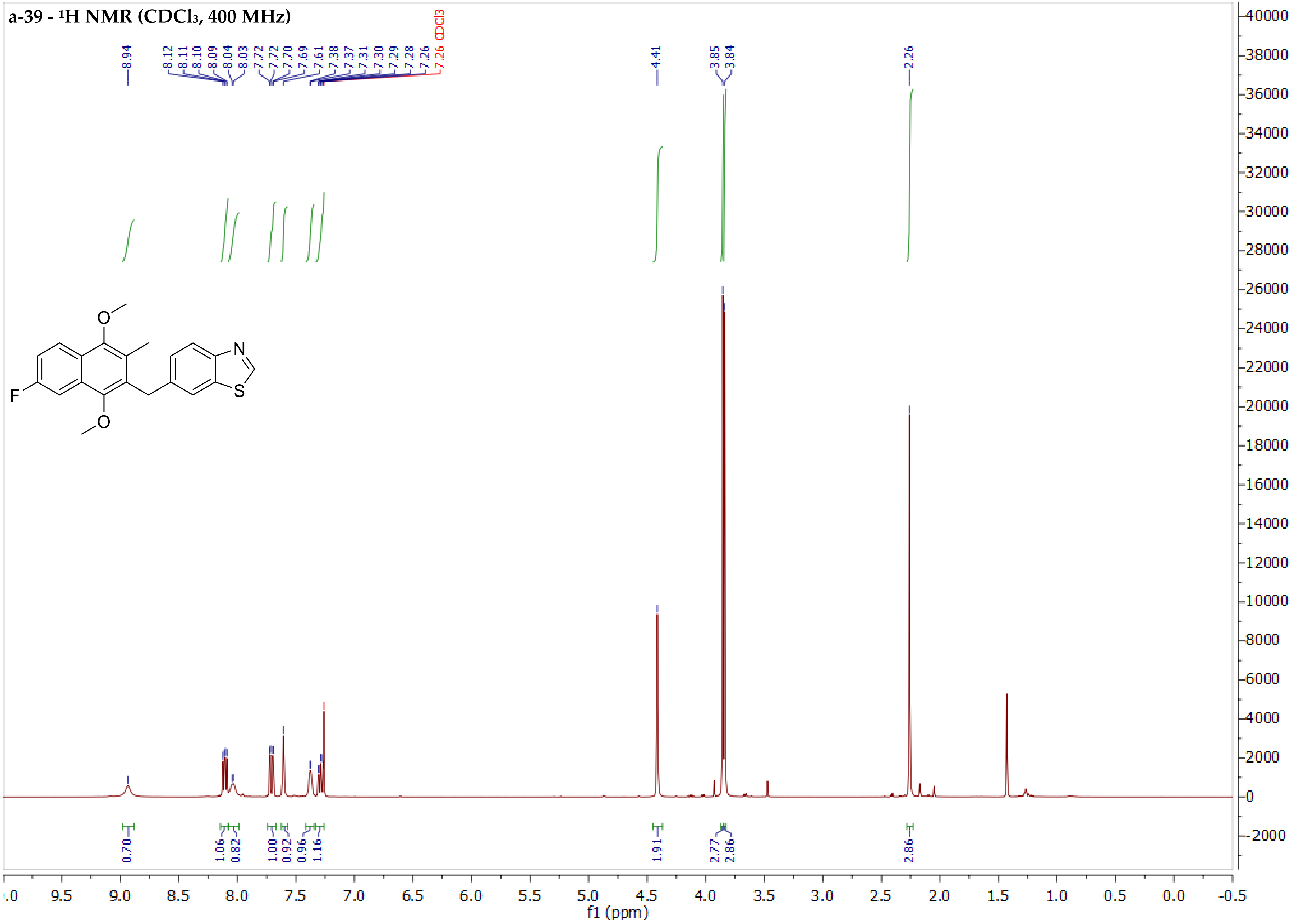

a-39 - ^{13}C {^{1}H} NMR ($CDCl_3$, 101 MHz)

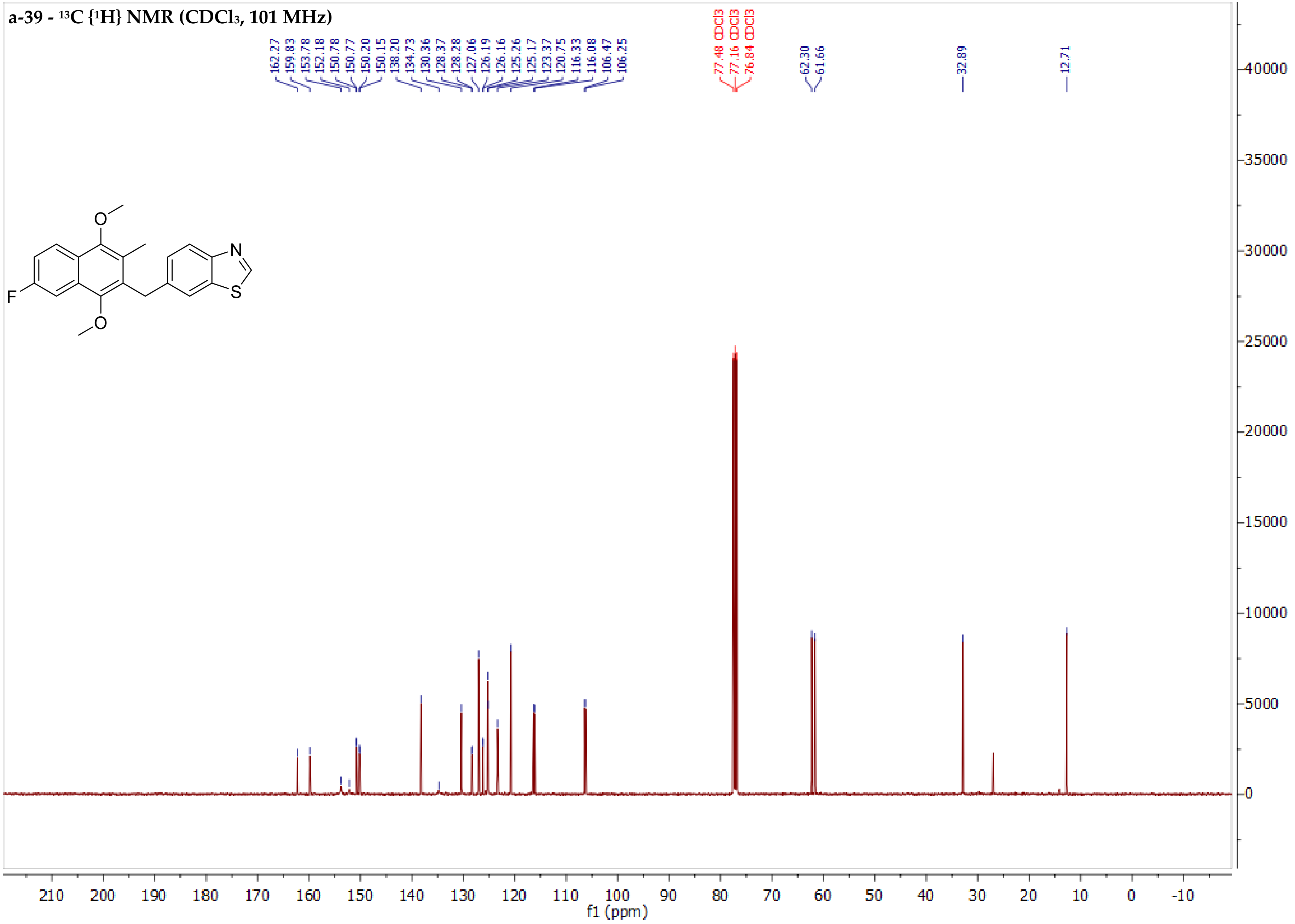

a-39 - ^{19}F NMR ($CDCl_3$, 377 MHz)

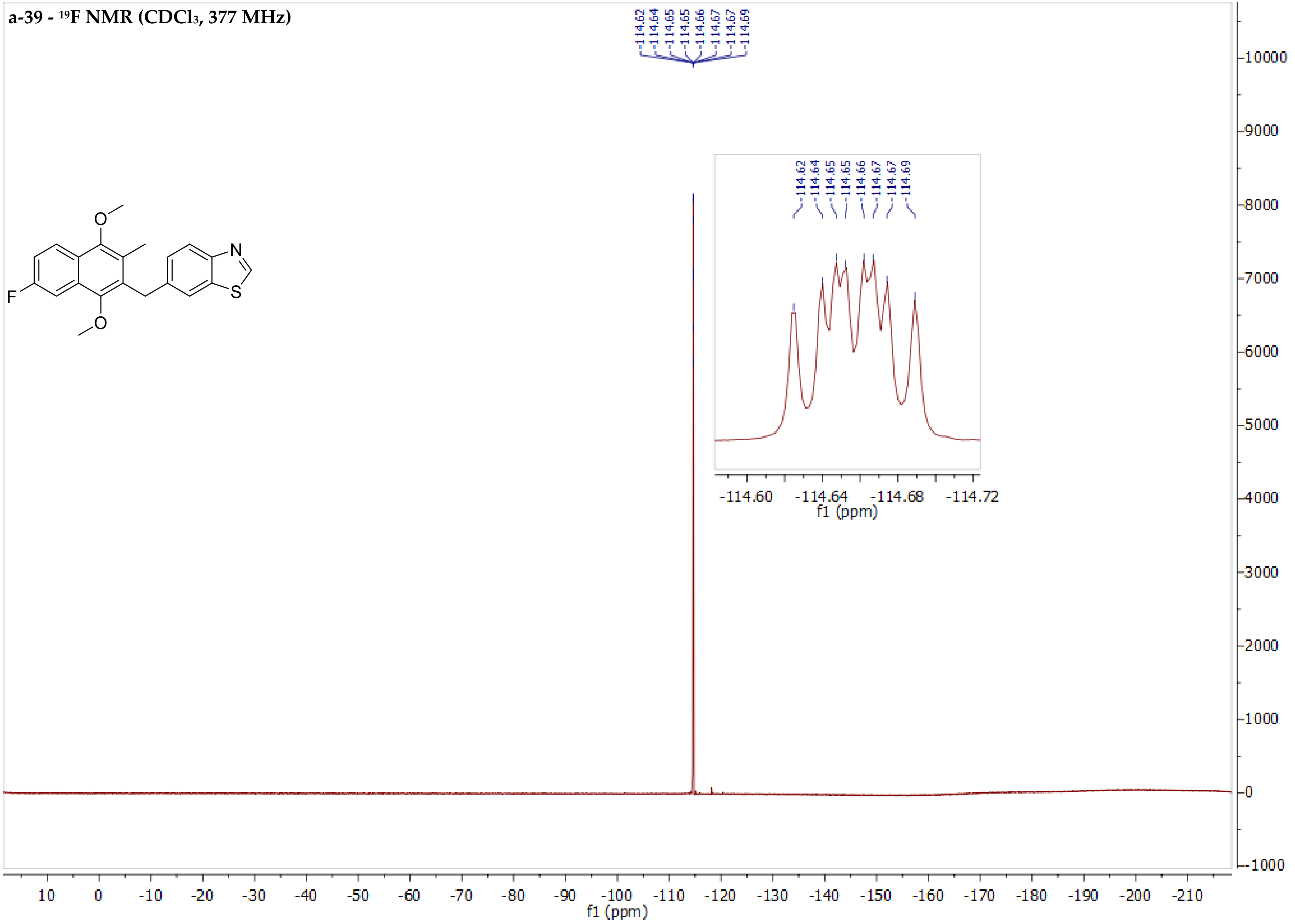

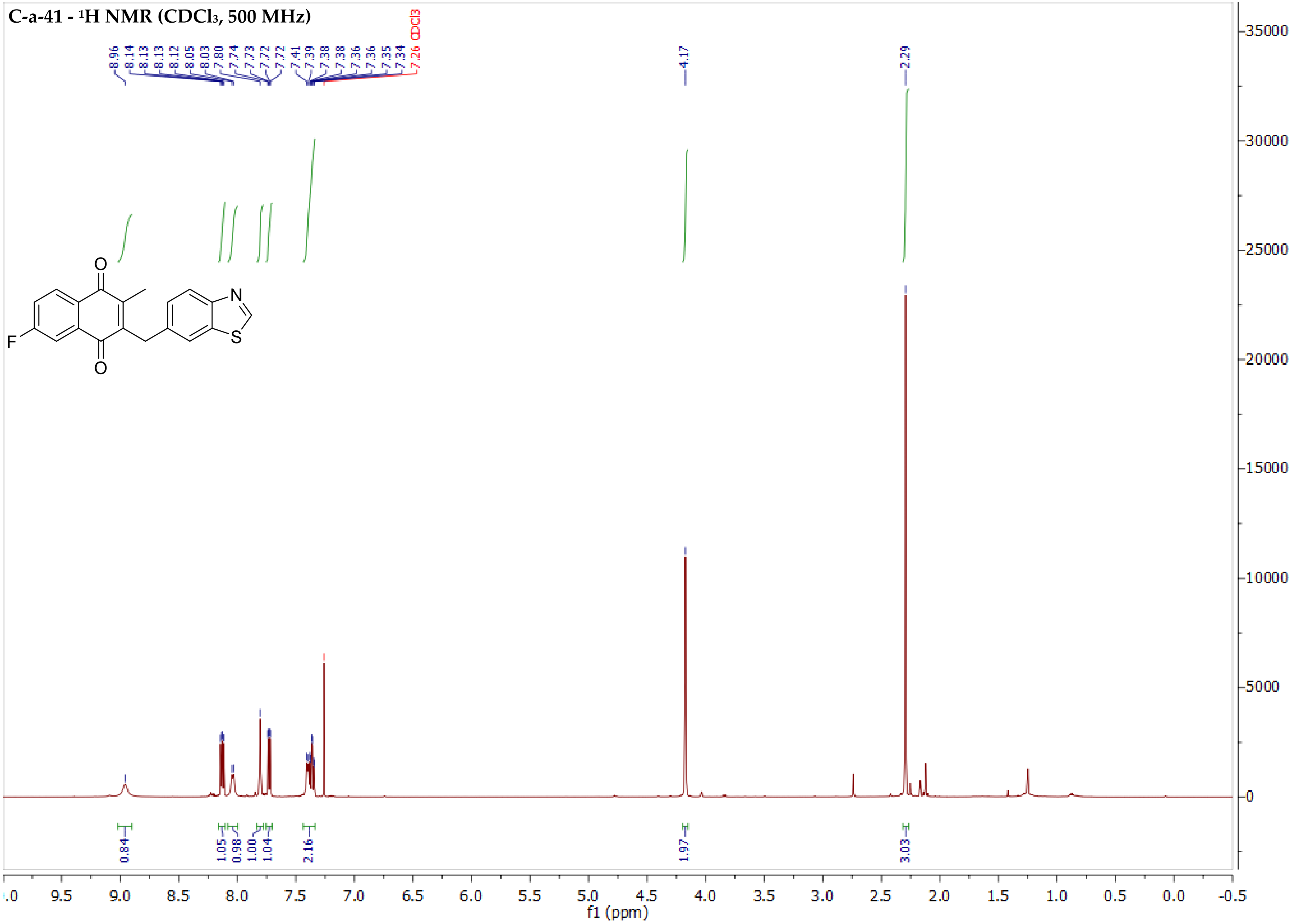
C-a-41 - 1H NMR (CDCl3, 500 MHz)
8.96
8.14
8.13
8.13
8.12
8.05
8.03
7.80
7.74
7.73
7.72
7.72
7.41
7.39
7.38
7.38
7.36
7.36
7.35
7.34
7.26 CDCl3
4.17
2.29
0.84
1.05
0.98
1.00
1.04
2.16
1.97
3.03
f1 (ppm)
O
O
F
N
S

C-a-41 - ^{13}C {^{1}H} NMR ($CDCl_3$, 127 MHz)

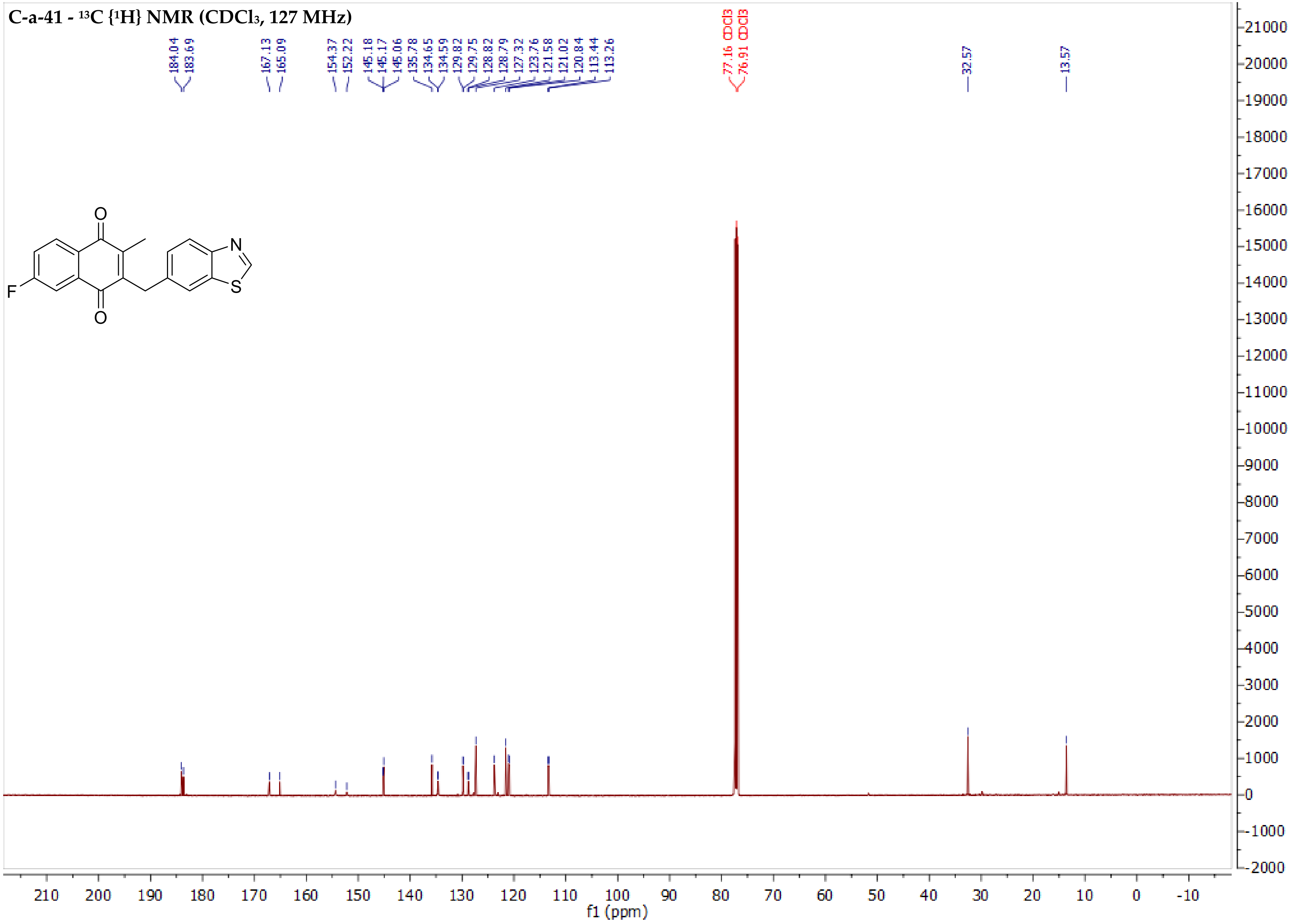

C-a-41 - ^{19}F NMR ($CDCl_3$, 377 MHz)

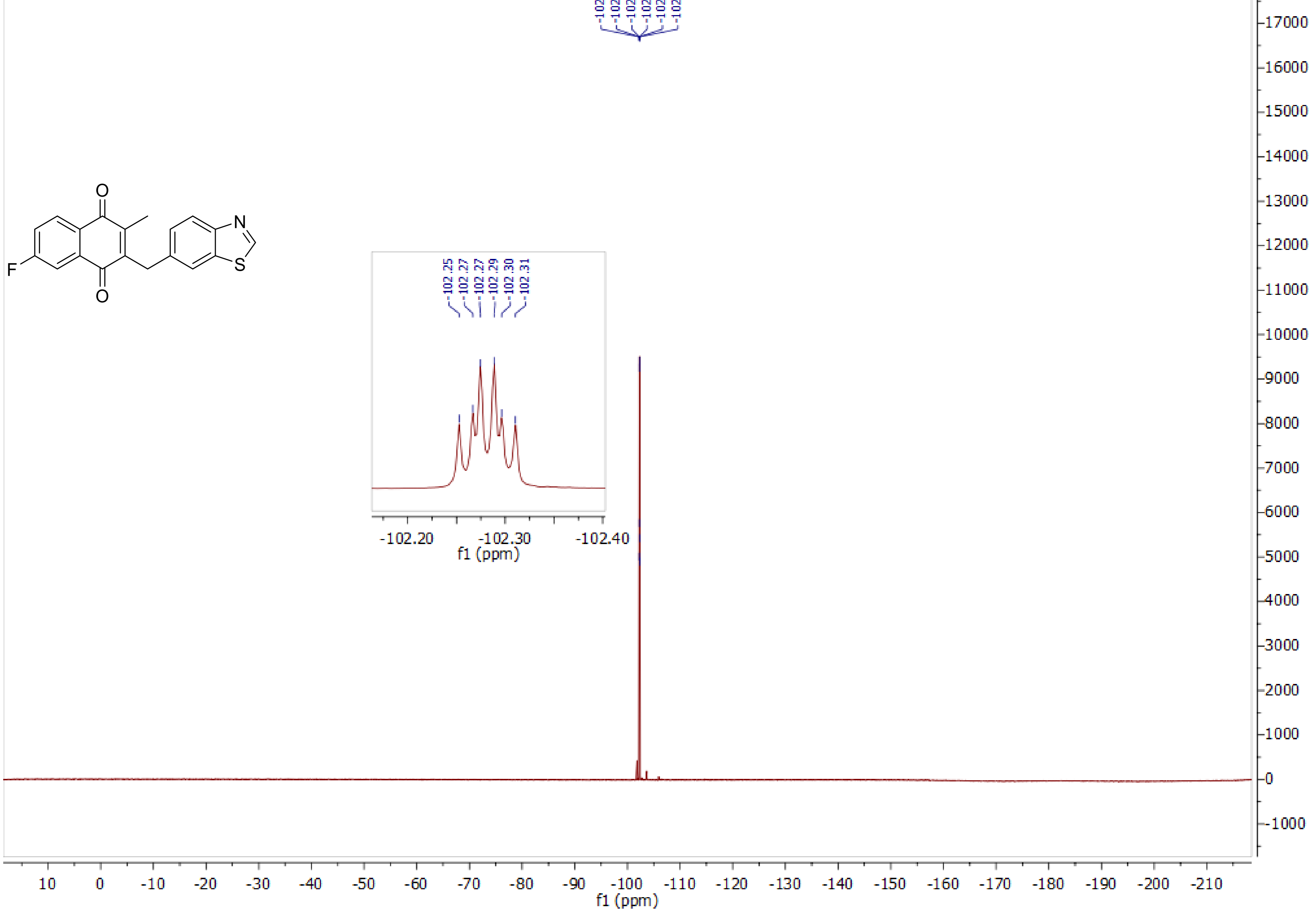

a-42 - ^1H NMR ($CDCl_3$, 400 MHz)

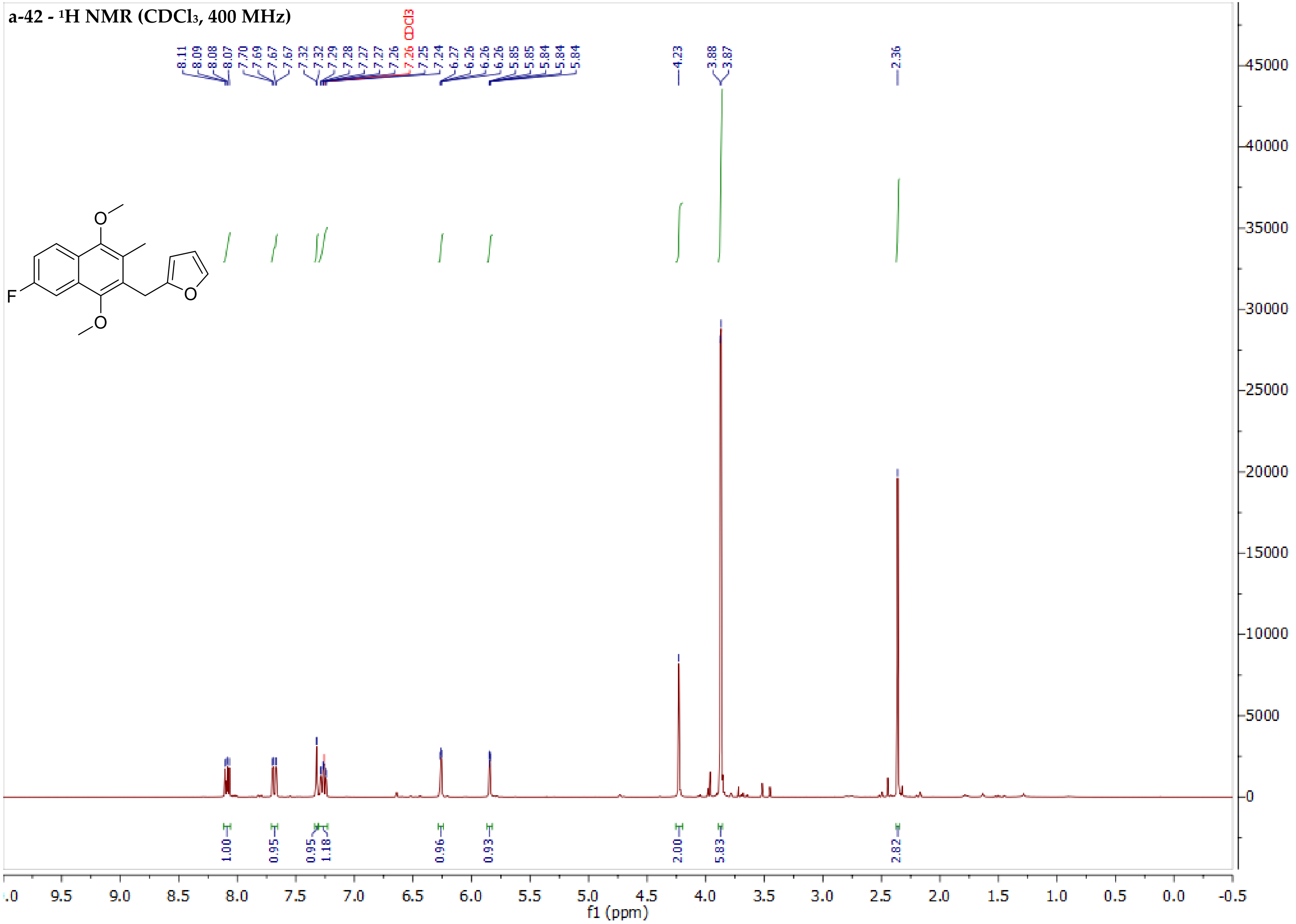

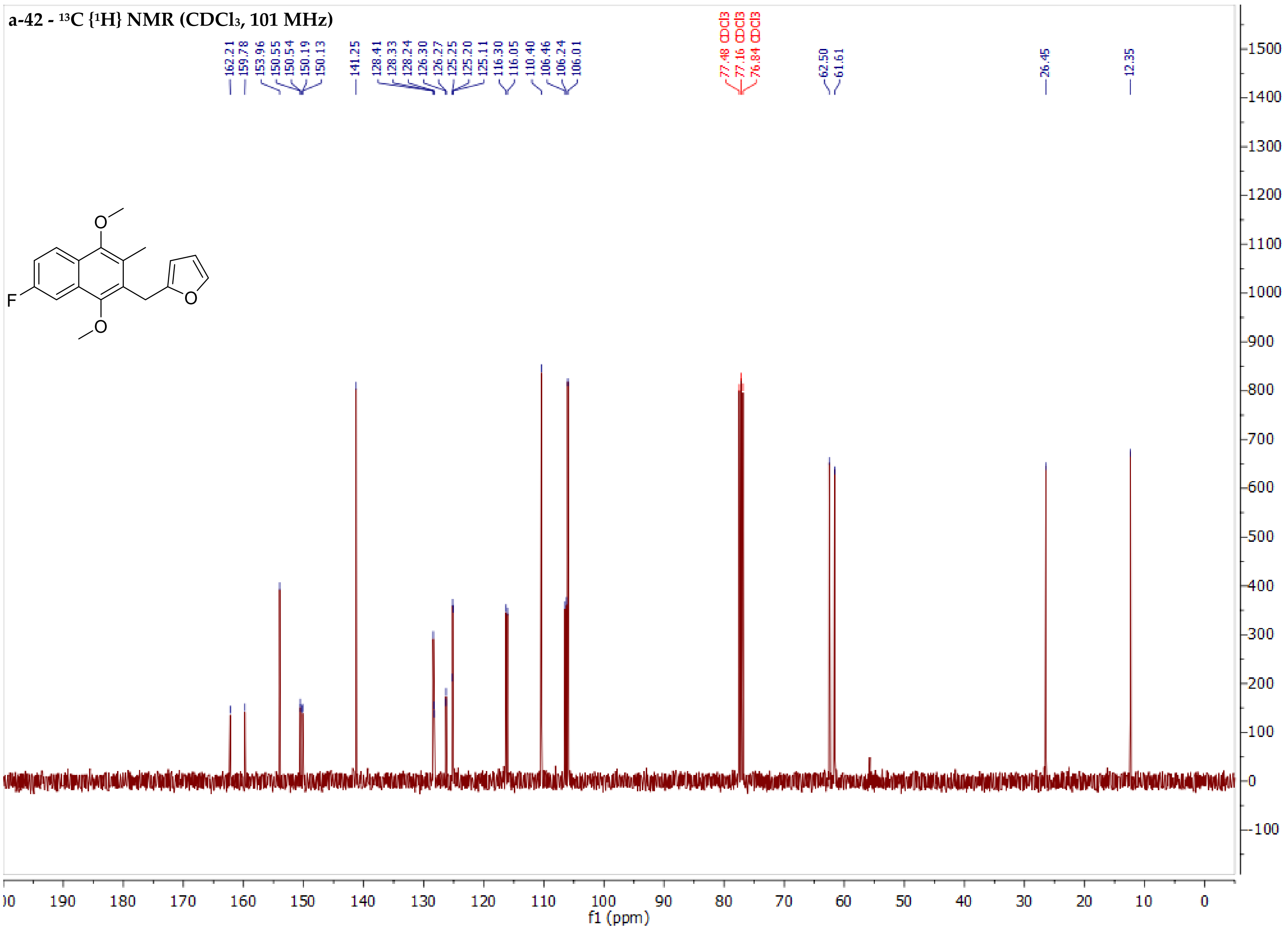
a-42 - ¹³C {¹H} NMR (CDCl₃, 101 MHz)
162.21
159.78
153.96
150.55
150.54
150.19
150.13
141.25
128.41
128.33
128.24
126.30
126.27
125.25
125.20
125.11
116.30
116.05
110.40
106.46
106.24
106.01
77.48 CDCl3
77.16 CDCl3
76.84 CDCl3
62.50
61.61
26.45
12.35
F
O
O
O
f1 (ppm)

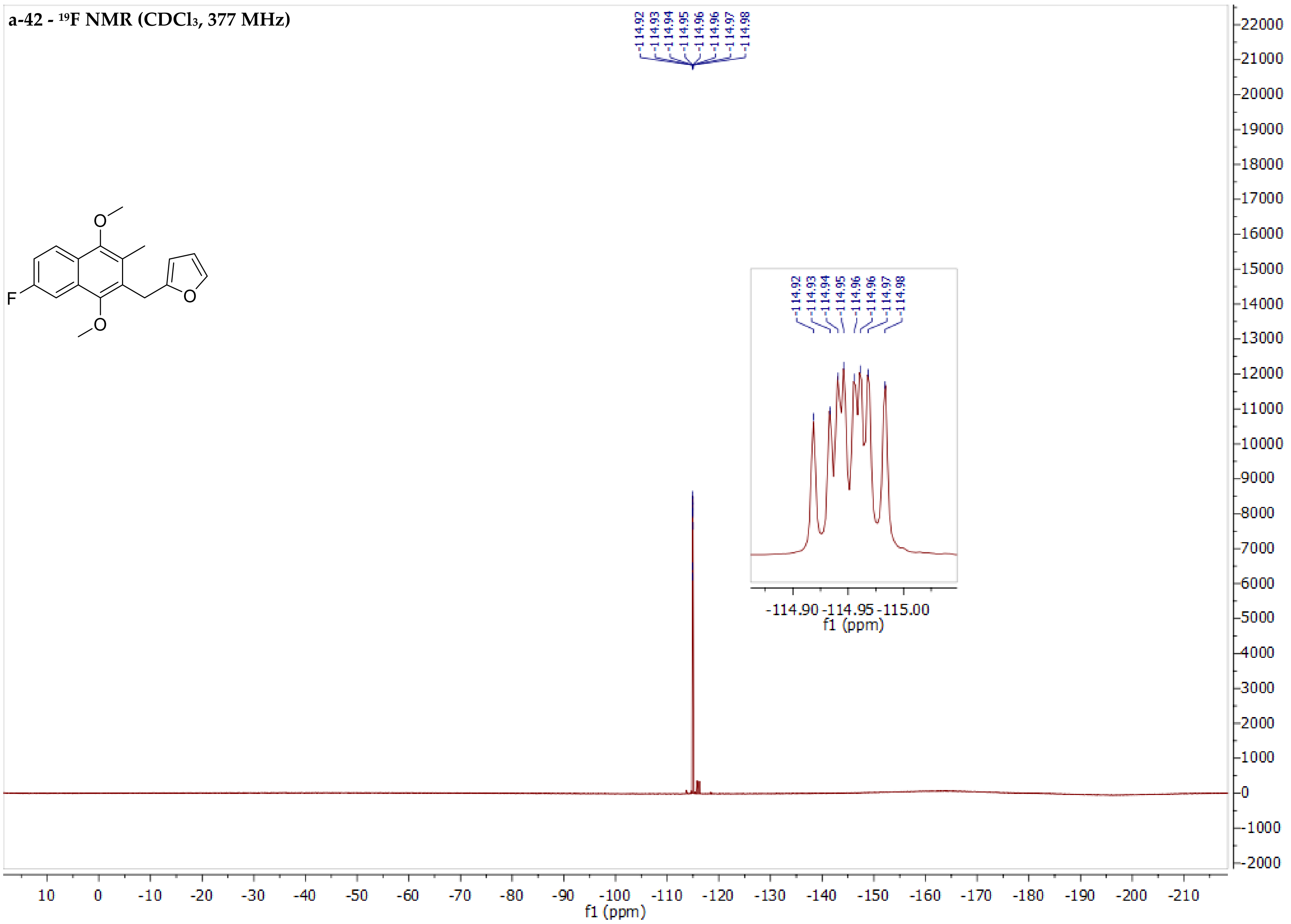
a-42 - 19F NMR (CDCl3, 377 MHz)
-114.92
-114.93
-114.94
-114.95
-114.96
-114.96
-114.97
-114.98
F
O
O
O
-114.90 -114.95 -115.00
f1 (ppm)
f1 (ppm)

C-a-43 - ^{1}H NMR ($CDCl_3$, 400 MHz)

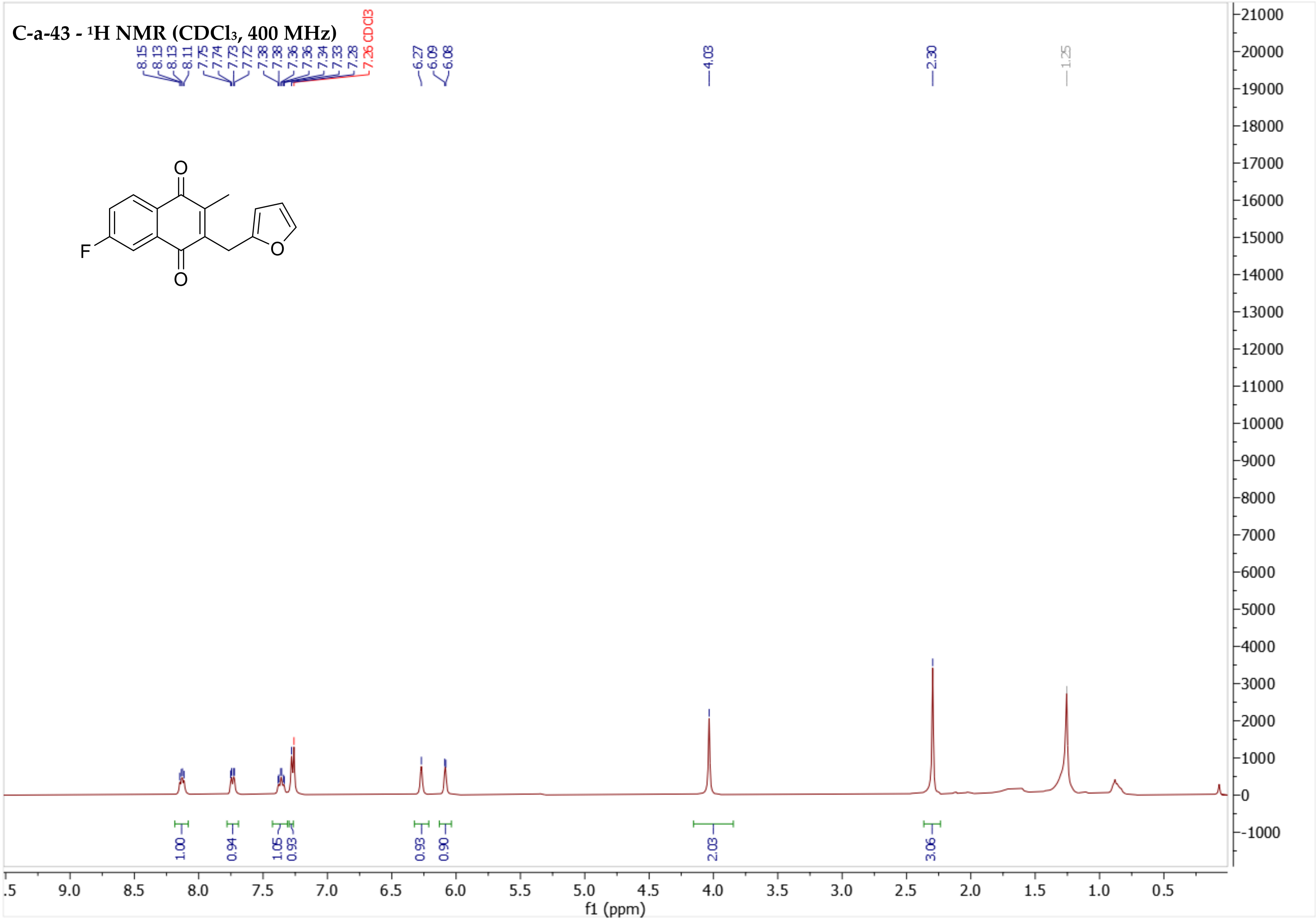

C-a-43 - ^{13}C {^{1}H} NMR ($CDCl_3$, 101 MHz)

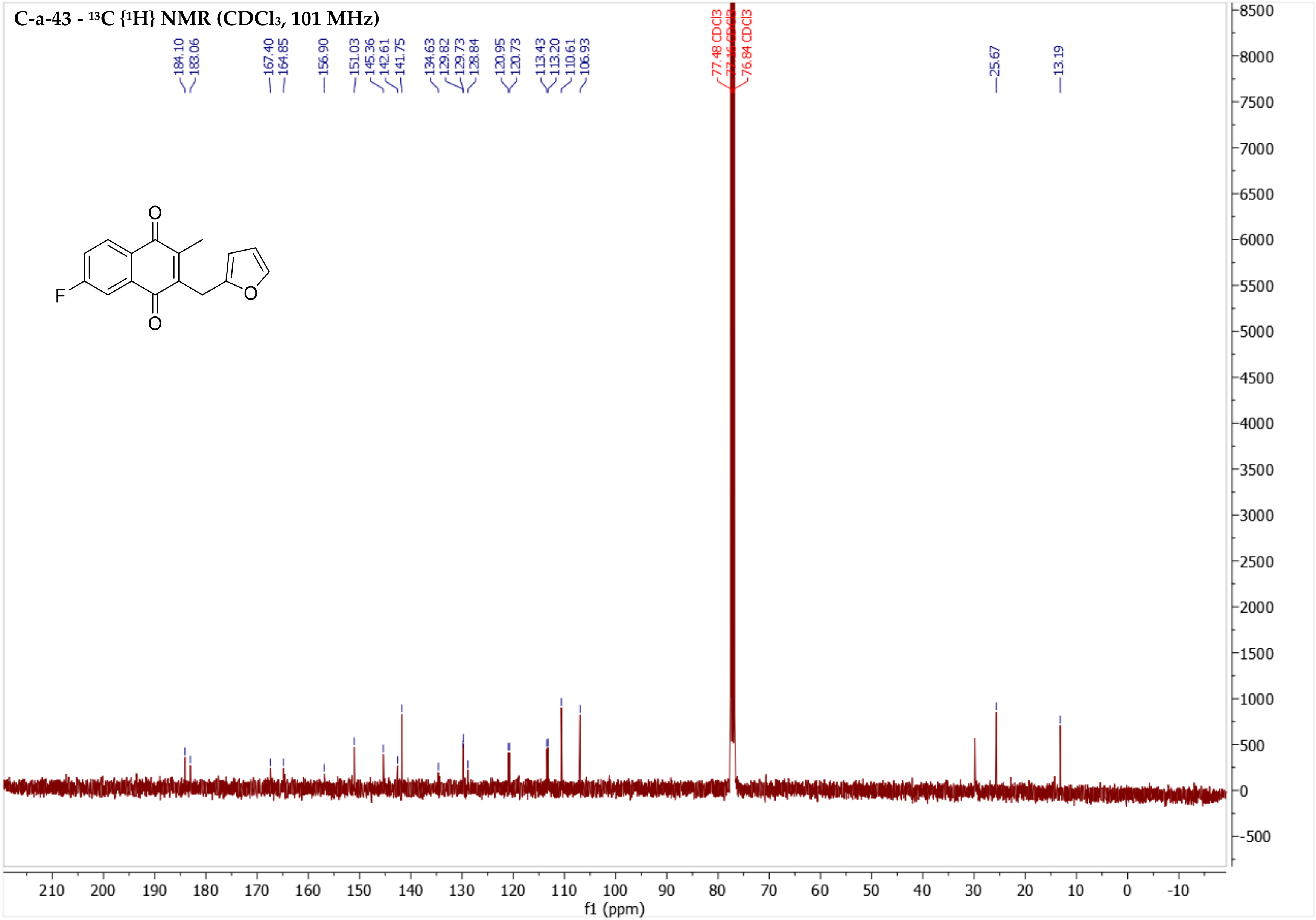

C-a-43 - ^{19}F NMR ($CDCl_3$, 377 MHz)

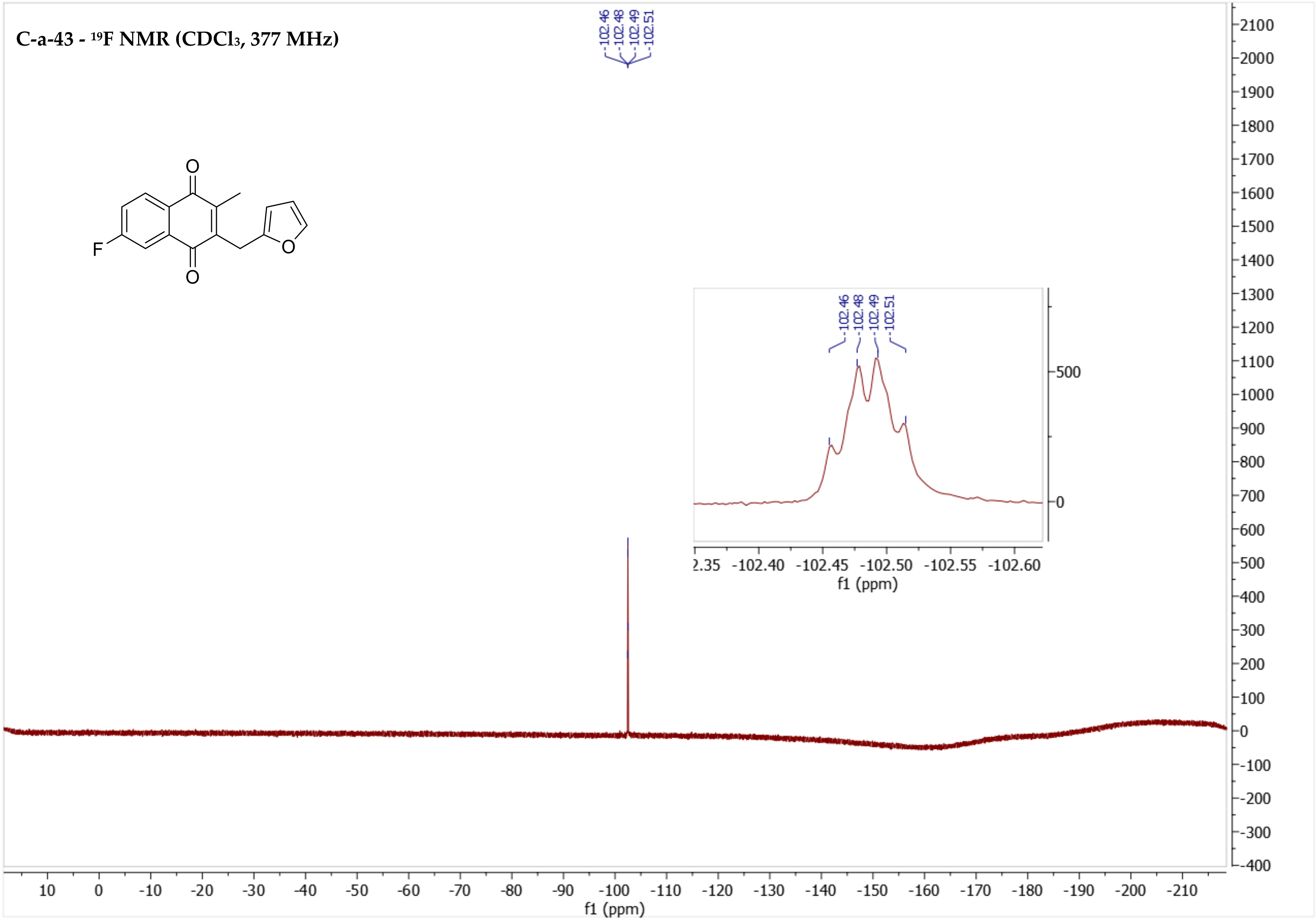

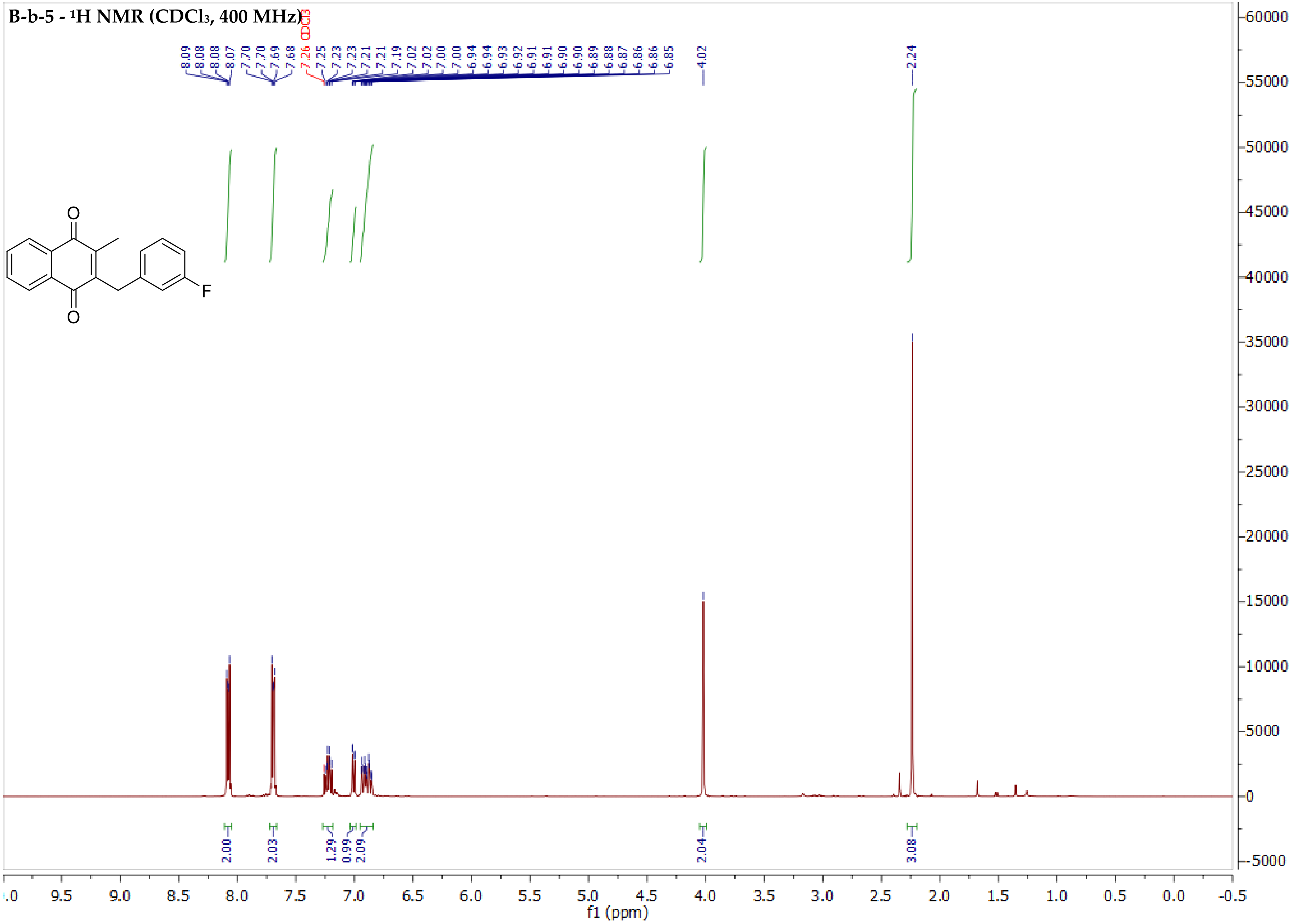
B-b-5 - ¹H NMR (CDCl₃, 400 MHz)
CDCl3
8.09 8.08 8.08 8.07 7.70 7.70 7.69 7.68 7.26 7.25 7.23 7.23 7.21 7.21 7.19 7.02 7.02 7.00 7.00 6.94 6.94 6.93 6.92 6.91 6.91 6.90 6.90 6.89 6.88 6.87 6.86 6.86 6.85
4.02
2.24
O
O
F
2.00 2.03 1.29 0.99 2.09 2.04 3.08
f1 (ppm)

B-b-5 - $^{13}C\{^1H\}$ NMR ($CDCl_3$, 101 MHz)

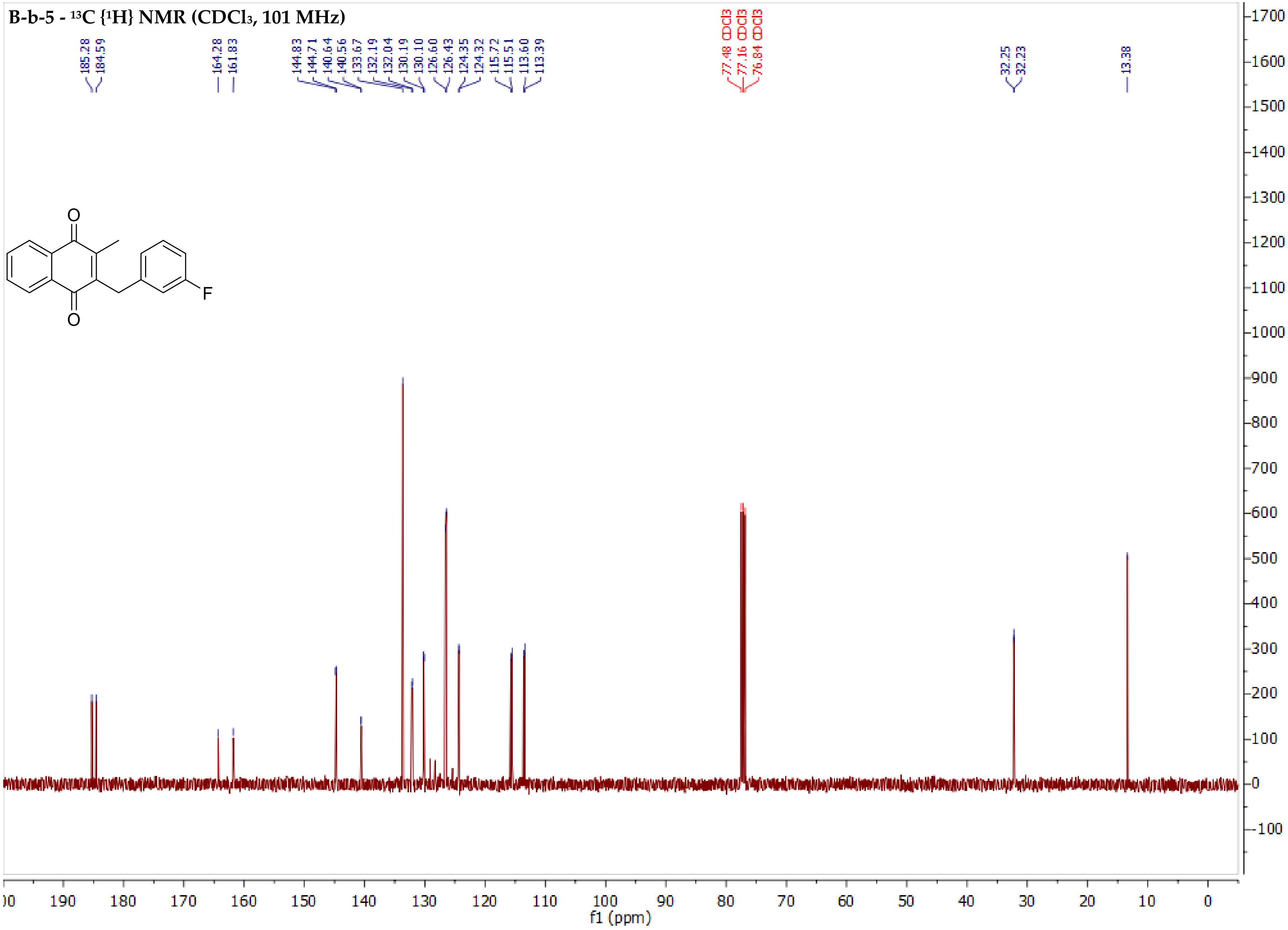

B-b-5 - ^{19}F NMR ($CDCl_3$, 377 MHz)

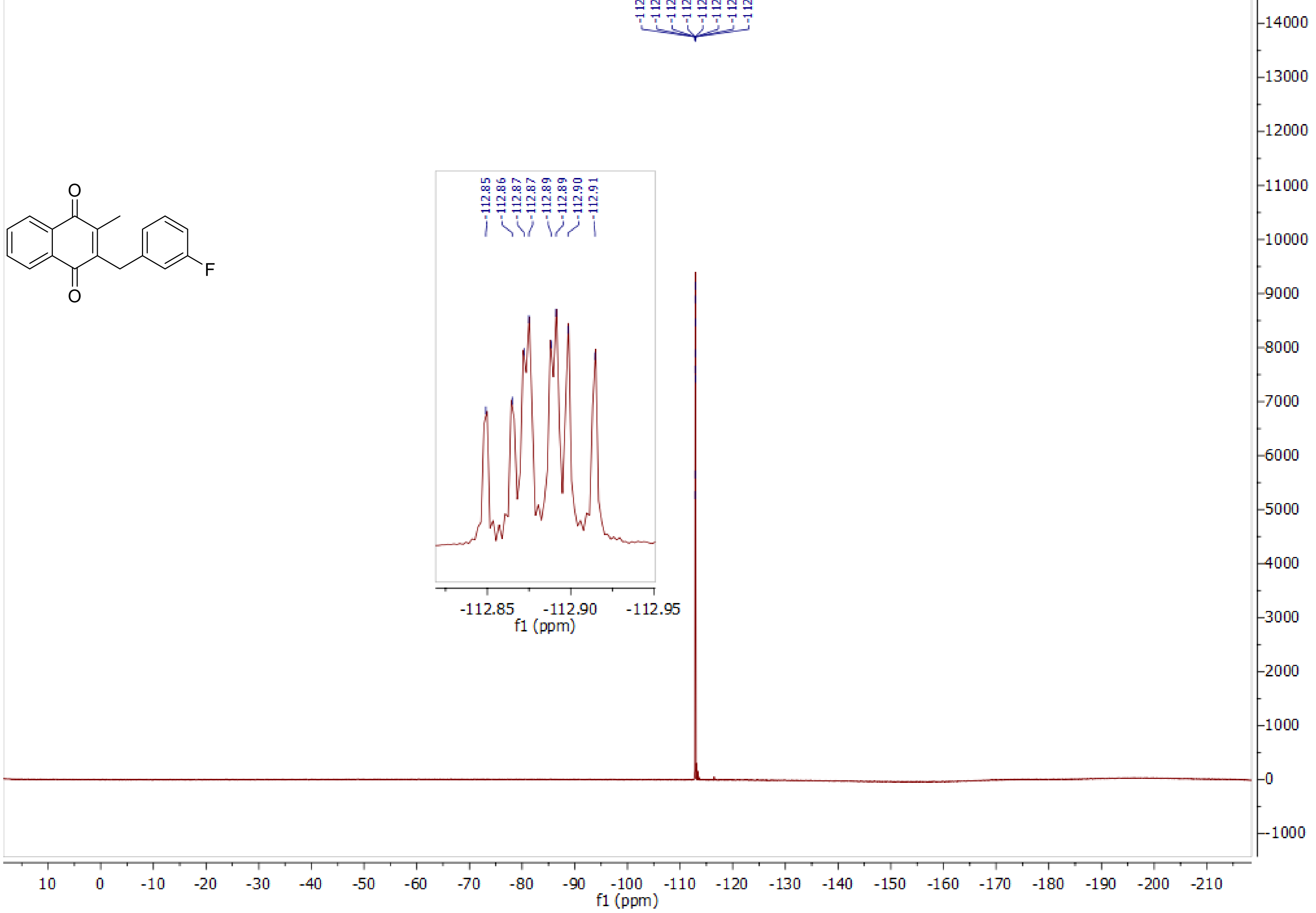

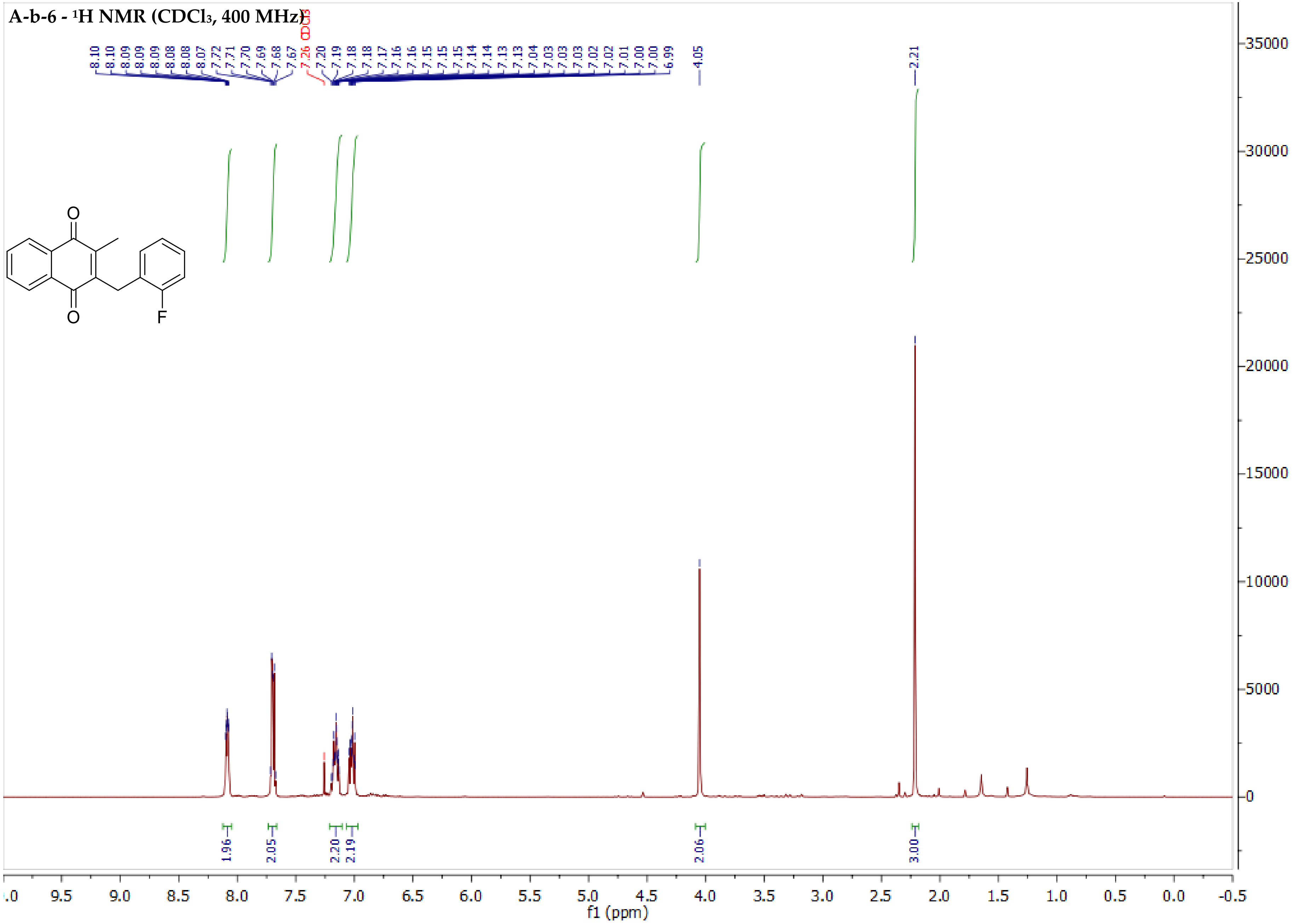
A-b-6 - 1H NMR (CDCl3, 400 MHz)
8.10 8.10 8.09 8.09 8.09 8.08 8.08 8.07 7.72 7.71 7.70 7.69 7.68 7.67 7.26 CDCl3 7.20 7.19 7.18 7.18 7.17 7.16 7.16 7.15 7.15 7.15 7.14 7.14 7.13 7.13 7.04 7.03 7.03 7.03 7.02 7.02 7.01 7.00 7.00 6.99
4.05
2.21
1.96 2.05 2.20 2.19 2.06 3.00
f1 (ppm)

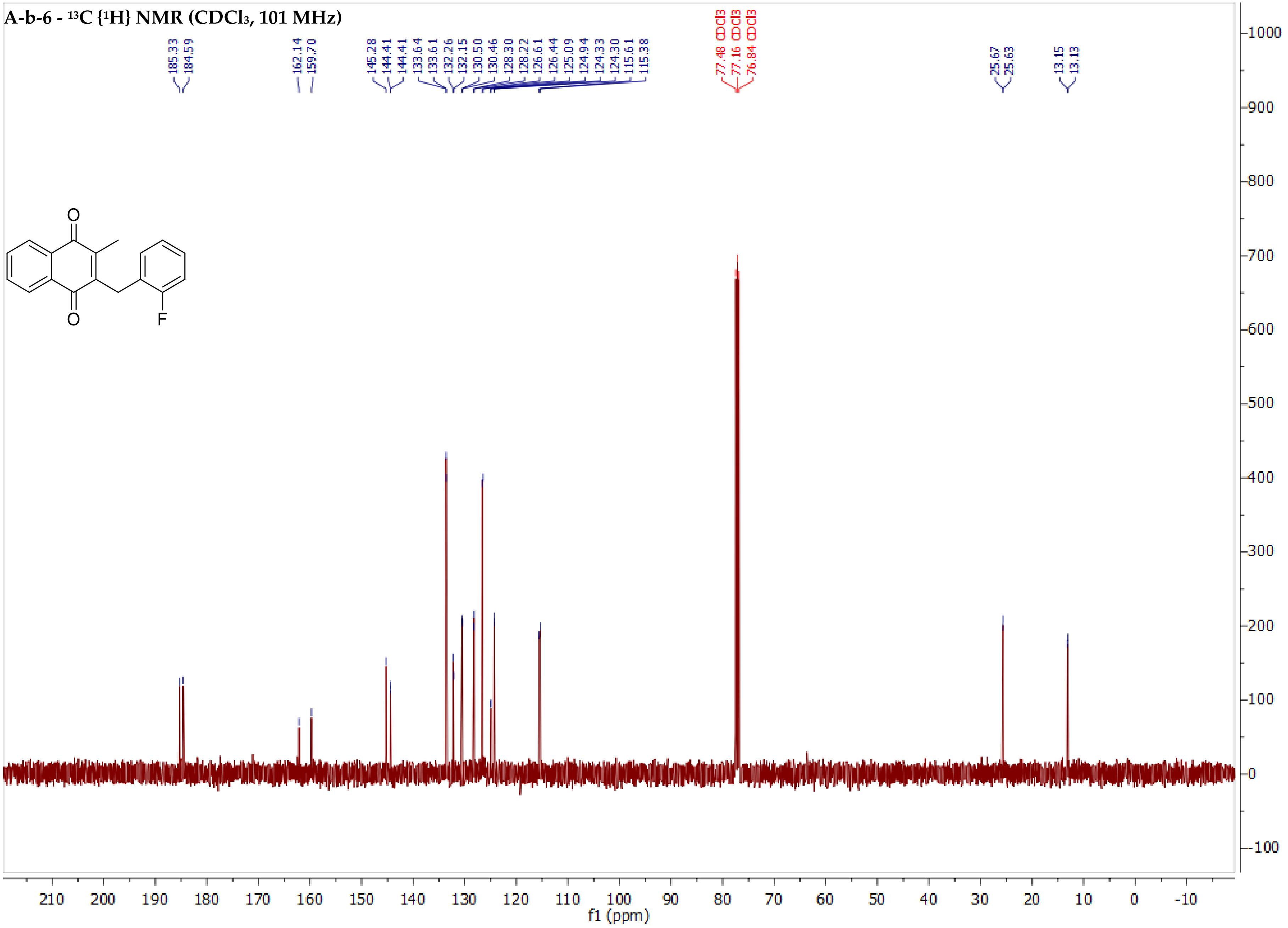
A-b-6 - ^{13}C {^{1}H} NMR ($CDCl_3$, 101 MHz)
185.33
184.59
162.14
159.70
145.28
144.41
144.41
133.64
133.61
132.26
132.15
130.50
130.46
128.30
128.22
126.61
126.44
125.09
124.94
124.33
124.30
115.61
115.38
77.48 CDCl3
77.16 CDCl3
76.84 CDCl3
25.67
25.63
13.15
13.13
O
O
F
f1 (ppm)

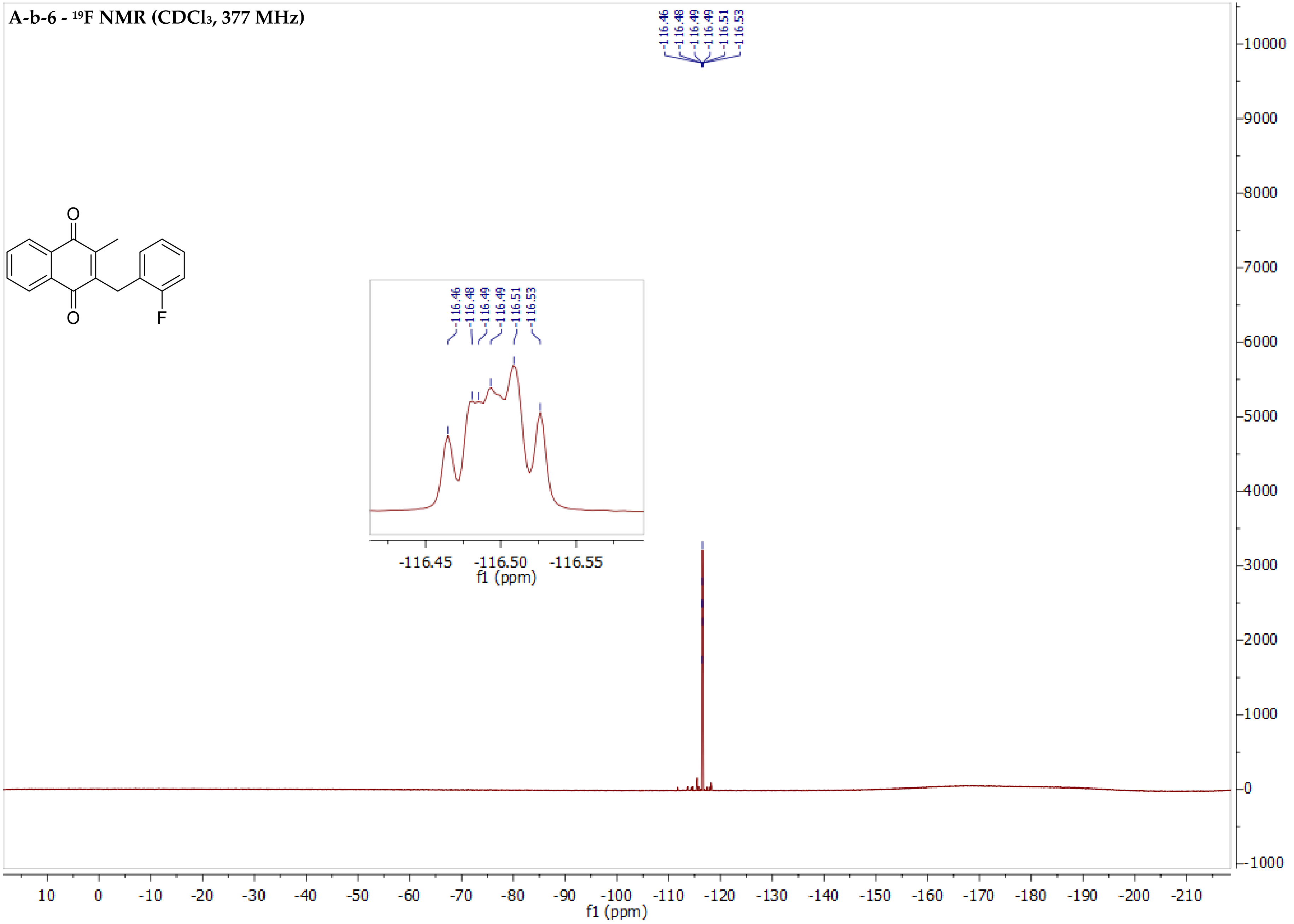
A-b-6 - 19F NMR (CDCl3, 377 MHz)
-116.46
-116.48
-116.49
-116.49
-116.51
-116.53
O
O
F
-116.45
-116.50
-116.55
f1 (ppm)
10000
9000
8000
7000
6000
5000
4000
3000
2000
1000
0
-1000
10
0
-10
-20
-30
-40
-50
-60
-70
-80
-90
-100
-110
-120
-130
-140
-150
-160
-170
-180
-190
-200
-210
f1 (ppm)

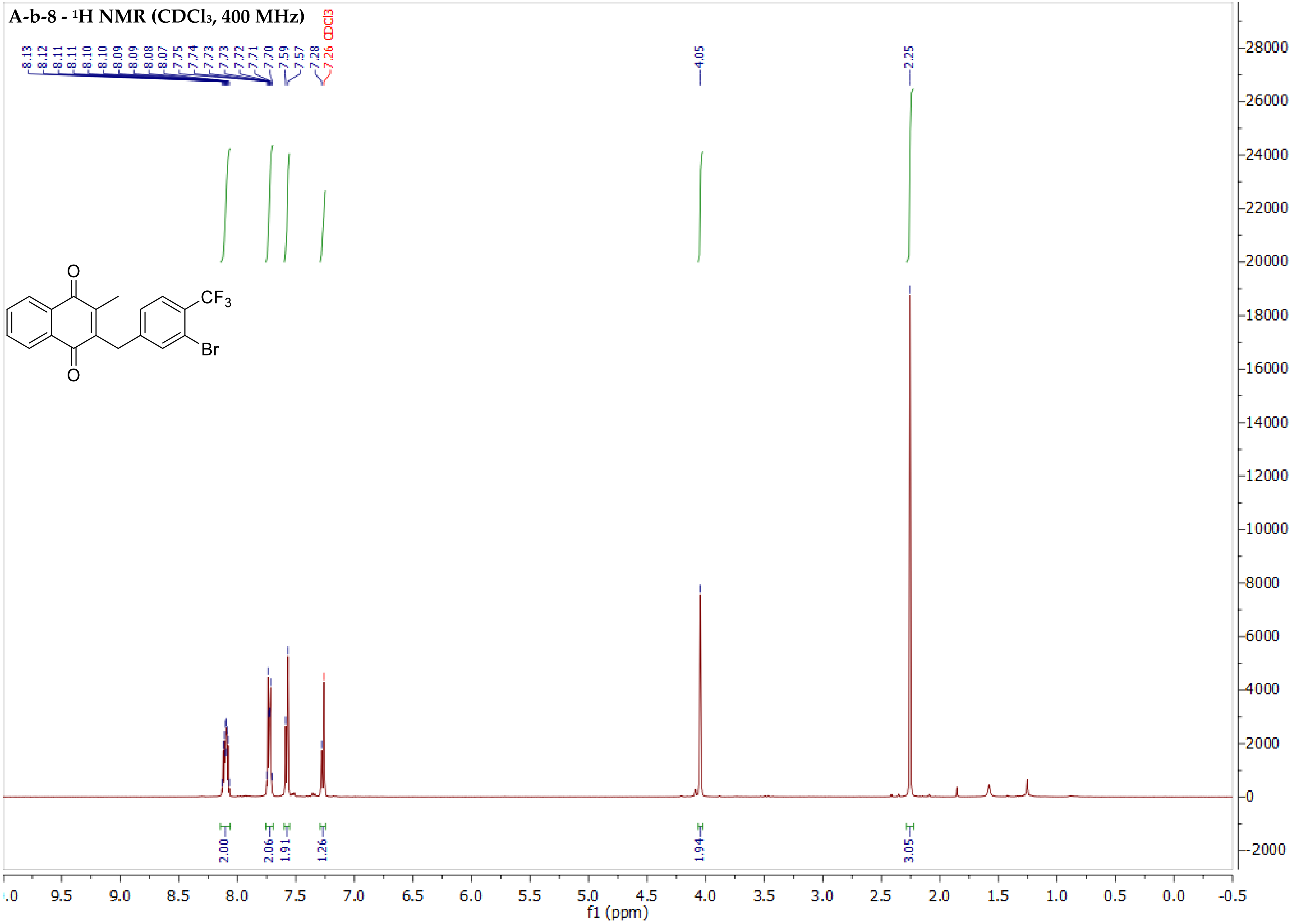
A-b-8 - ¹H NMR (CDCl₃, 400 MHz)
8.13 8.12 8.11 8.11 8.10 8.10 8.09 8.09 8.08 8.07 7.75 7.74 7.73 7.73 7.72 7.71 7.70 7.59 7.57 7.28 7.26
CDCl3
4.05
2.25
CF3
Br
O
O
2.00
2.06
1.91
1.26
1.94
3.05
f1 (ppm)

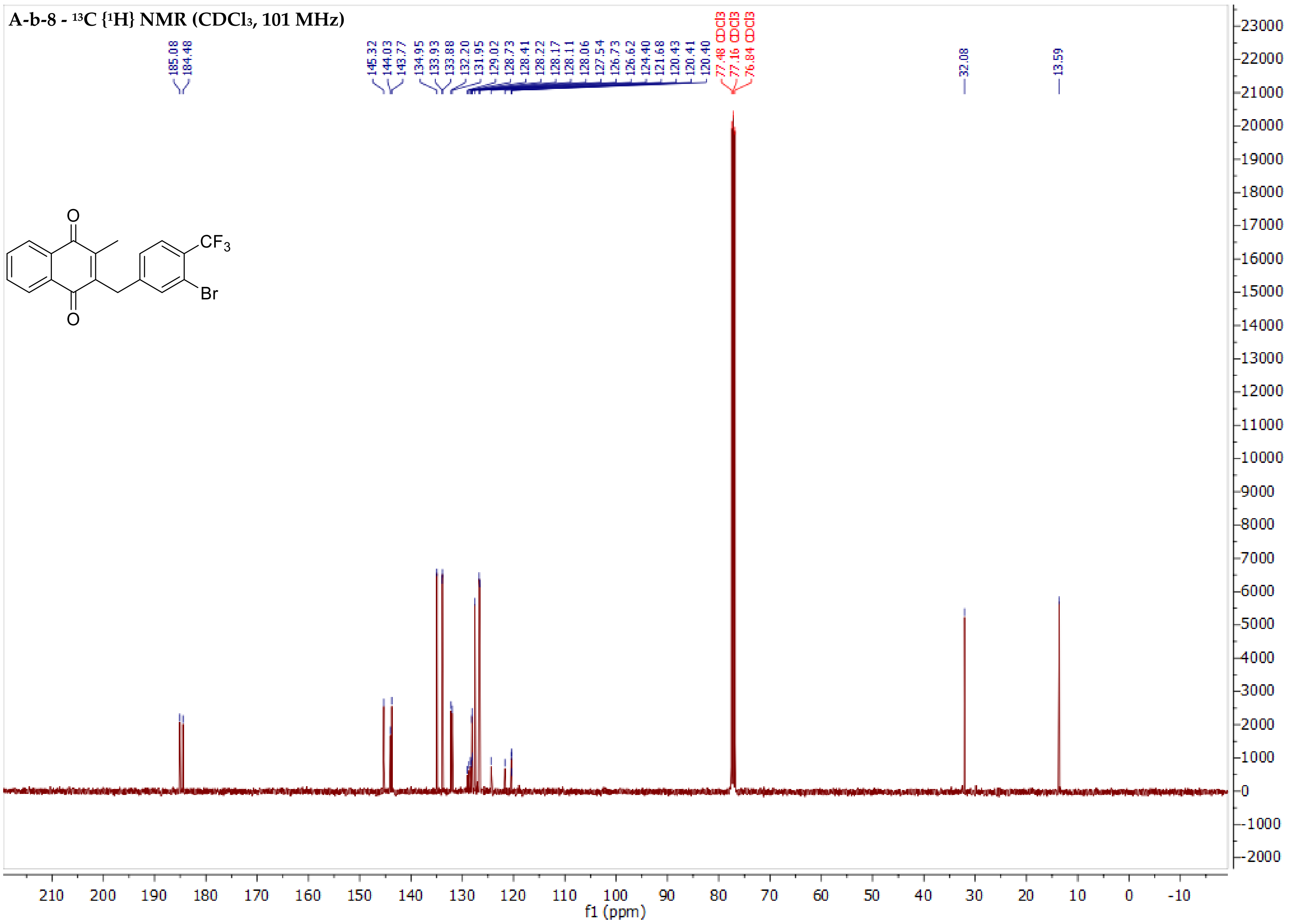
A-b-8 - 13C {1H} NMR (CDCl3, 101 MHz)
185.08
184.48
145.32
144.03
143.77
134.95
133.93
133.88
132.20
131.95
129.02
128.73
128.41
128.22
128.17
128.11
128.06
127.54
126.73
126.62
124.40
121.68
120.43
120.41
120.40
77.48 CDCl3
77.16 CDCl3
76.84 CDCl3
32.08
13.59
O
O
CF3
Br
f1 (ppm)

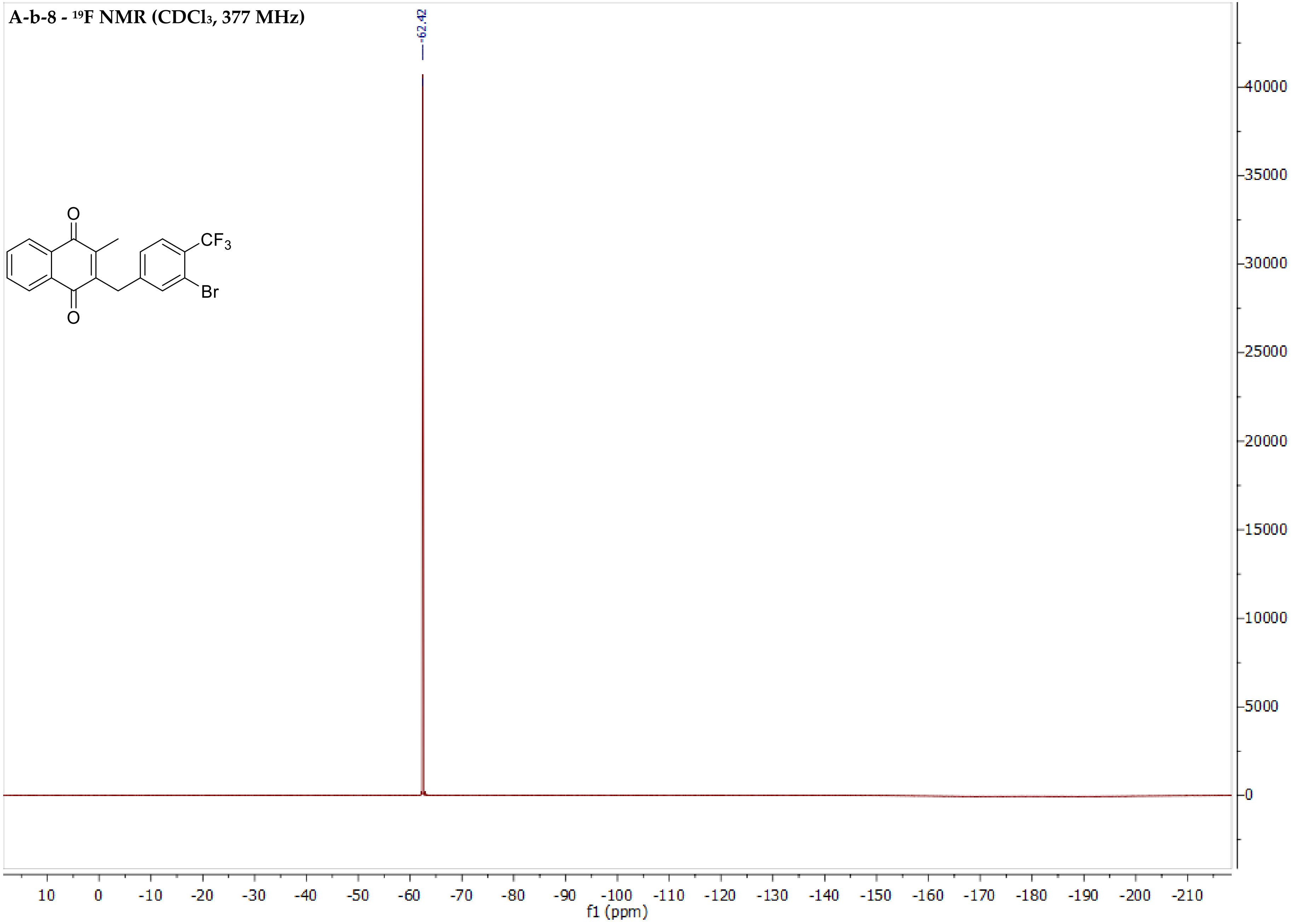
A-b-8 - 19F NMR (CDCl3, 377 MHz)
-62.42
O
O
CF3
Br
40000
35000
30000
25000
20000
15000
10000
5000
0
10 0 -10 -20 -30 -40 -50 -60 -70 -80 -90 -100 -110 -120 -130 -140 -150 -160 -170 -180 -190 -200 -210
f1 (ppm)

A-b-9 - ¹H NMR (CDCl₃, 400 MHz)

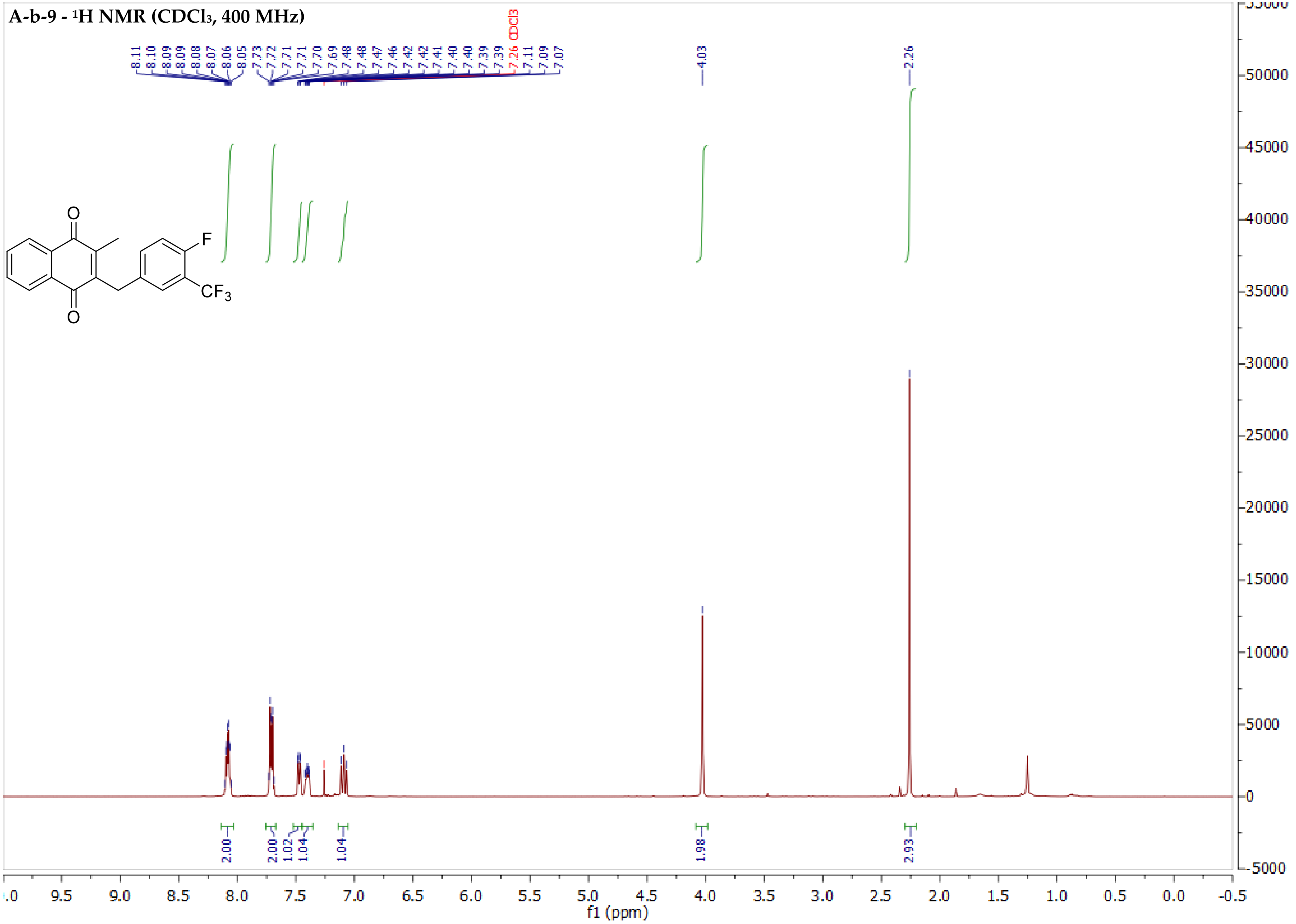

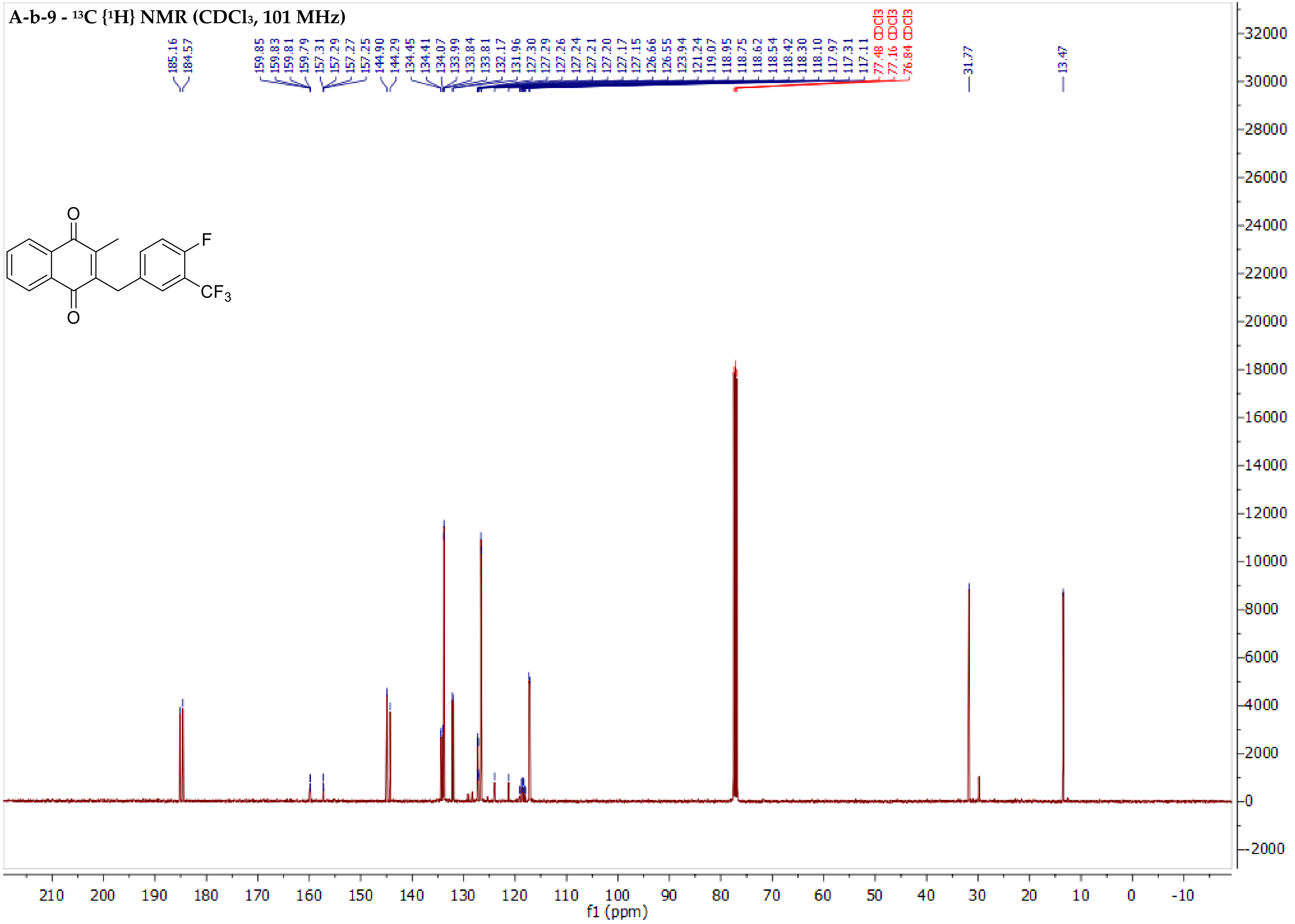
A-b-9 - ¹³C {¹H} NMR (CDCl₃, 101 MHz)
185.16
184.57
159.85
159.83
159.81
159.79
157.31
157.29
157.27
157.25
144.90
144.29
134.45
134.41
134.07
133.99
133.84
133.81
132.17
131.96
127.30
127.29
127.26
127.24
127.21
127.20
127.17
127.15
126.66
126.55
123.94
121.24
119.07
118.95
118.75
118.62
118.54
118.42
118.30
118.10
117.97
117.31
117.11
77.48 CDCl3
77.16 CDCl3
76.84 CDCl3
31.77
13.47
O
O
F
CF3
f1 (ppm)

A-b-9 - ^{19}F NMR ($CDCl_3$, 377 MHz)

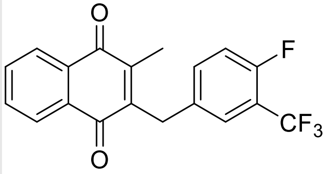

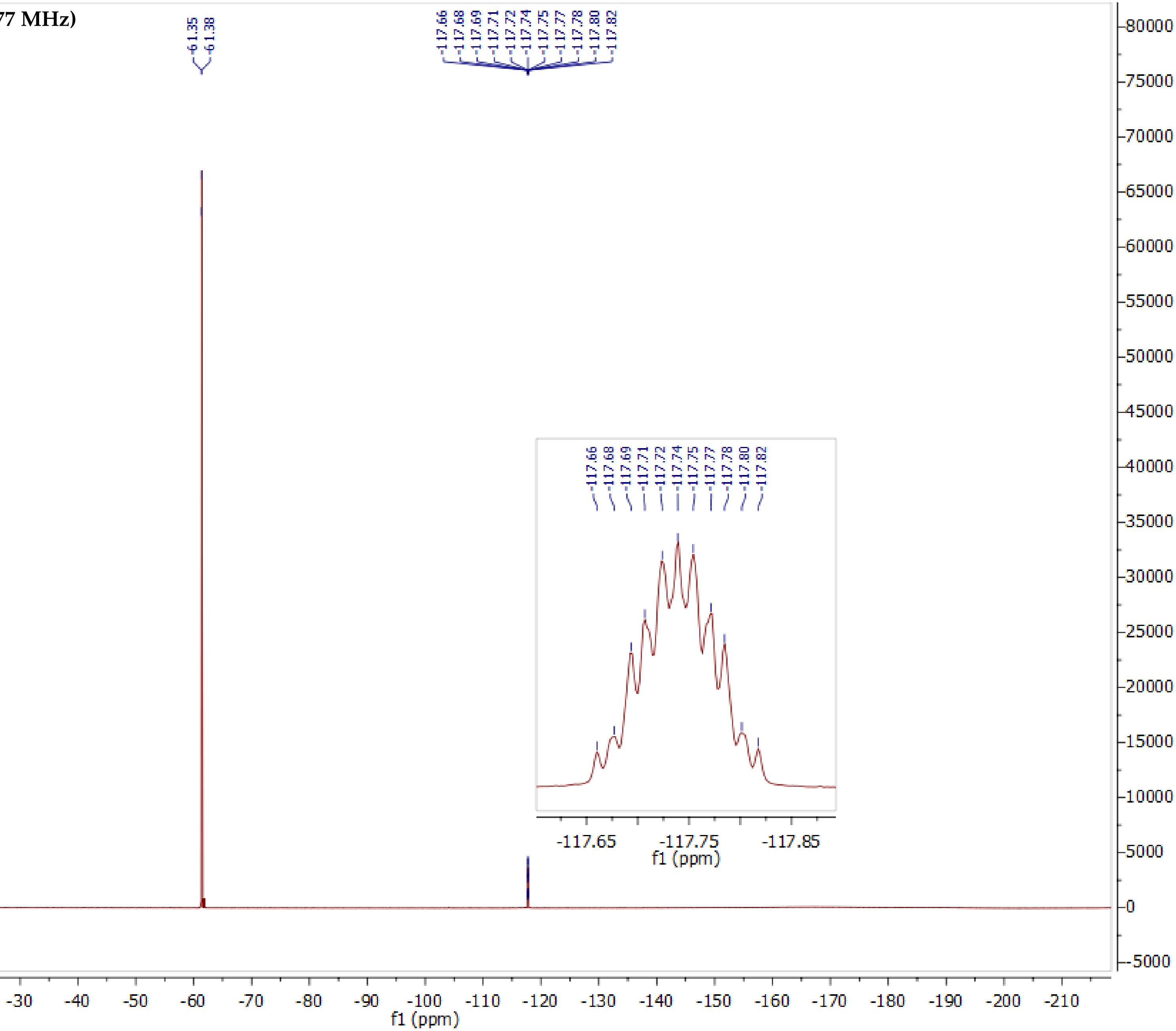

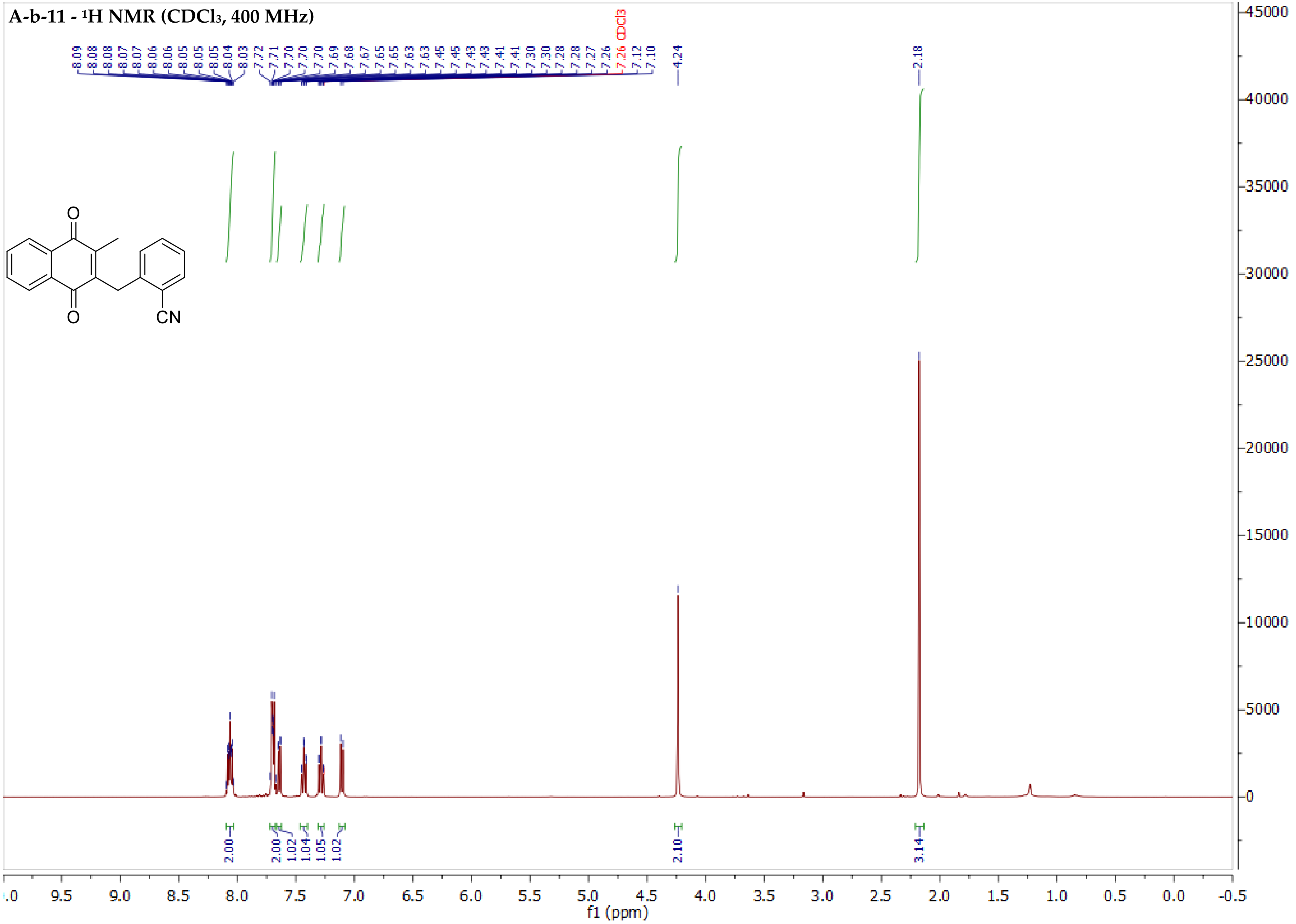
A-b-11 - ¹H NMR (CDCl₃, 400 MHz)
CDCl3
8.09 8.08 8.08 8.07 8.07 8.06 8.06 8.05 8.05 8.04 8.03 7.72 7.71 7.70 7.70 7.70 7.69 7.68 7.67 7.65 7.65 7.63 7.63 7.45 7.45 7.43 7.43 7.41 7.41 7.30 7.30 7.28 7.28 7.27 7.26 7.26 7.12 7.10
4.24
2.18
O
O
CN
2.00 2.00 1.02 1.04 1.05 1.02
2.10
3.14
f1 (ppm)

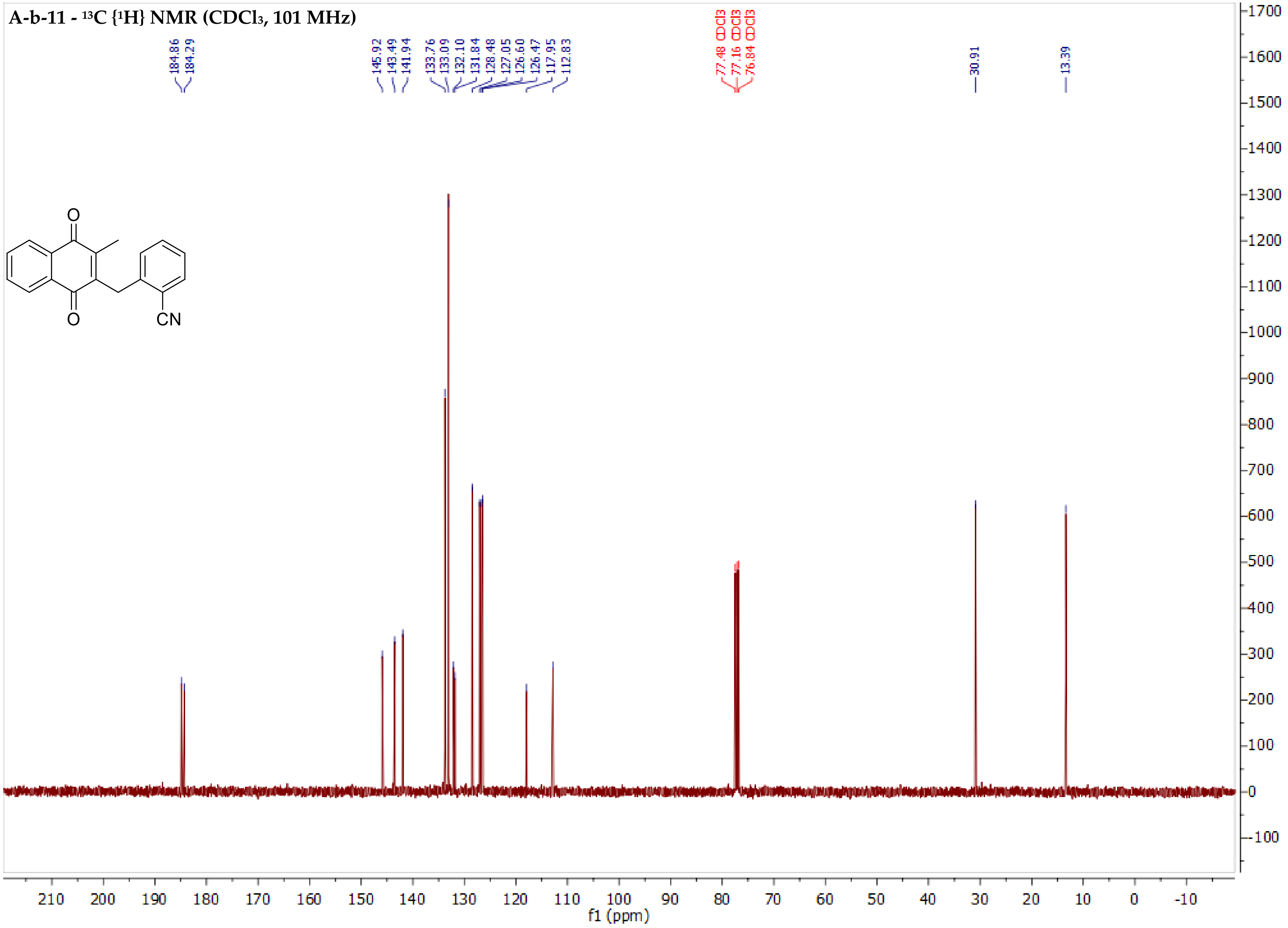
A-b-11 - 13C {1H} NMR (CDCl3, 101 MHz)
184.86
184.29
145.92
143.49
141.94
133.76
133.09
132.10
131.84
128.48
127.05
126.60
126.47
117.95
112.83
77.48 CDCl3
77.16 CDCl3
76.84 CDCl3
30.91
13.39
O
O
CN
f1 (ppm)

A-b-14 - ^{1}H NMR ($CDCl_3$, 400 MHz)

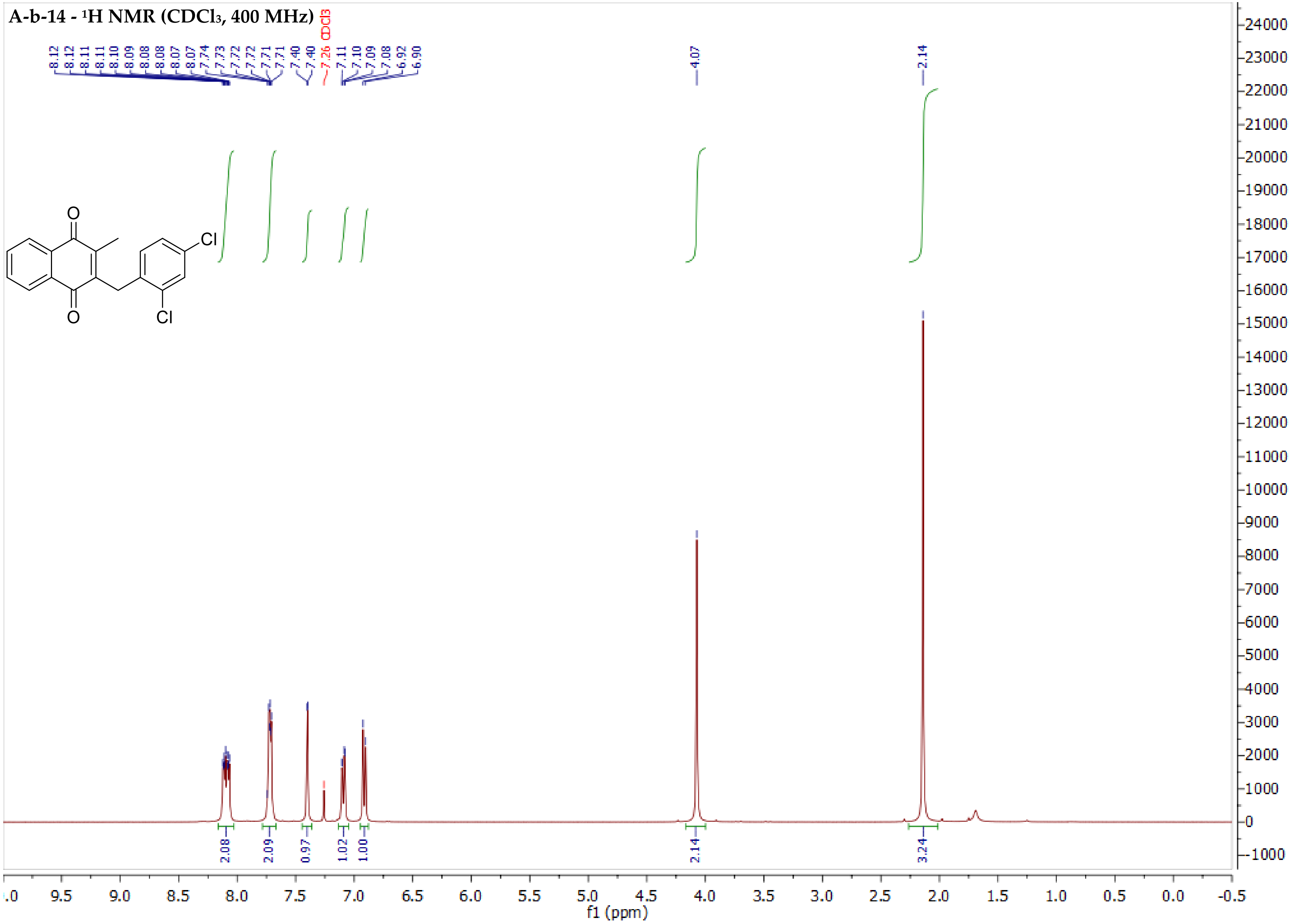

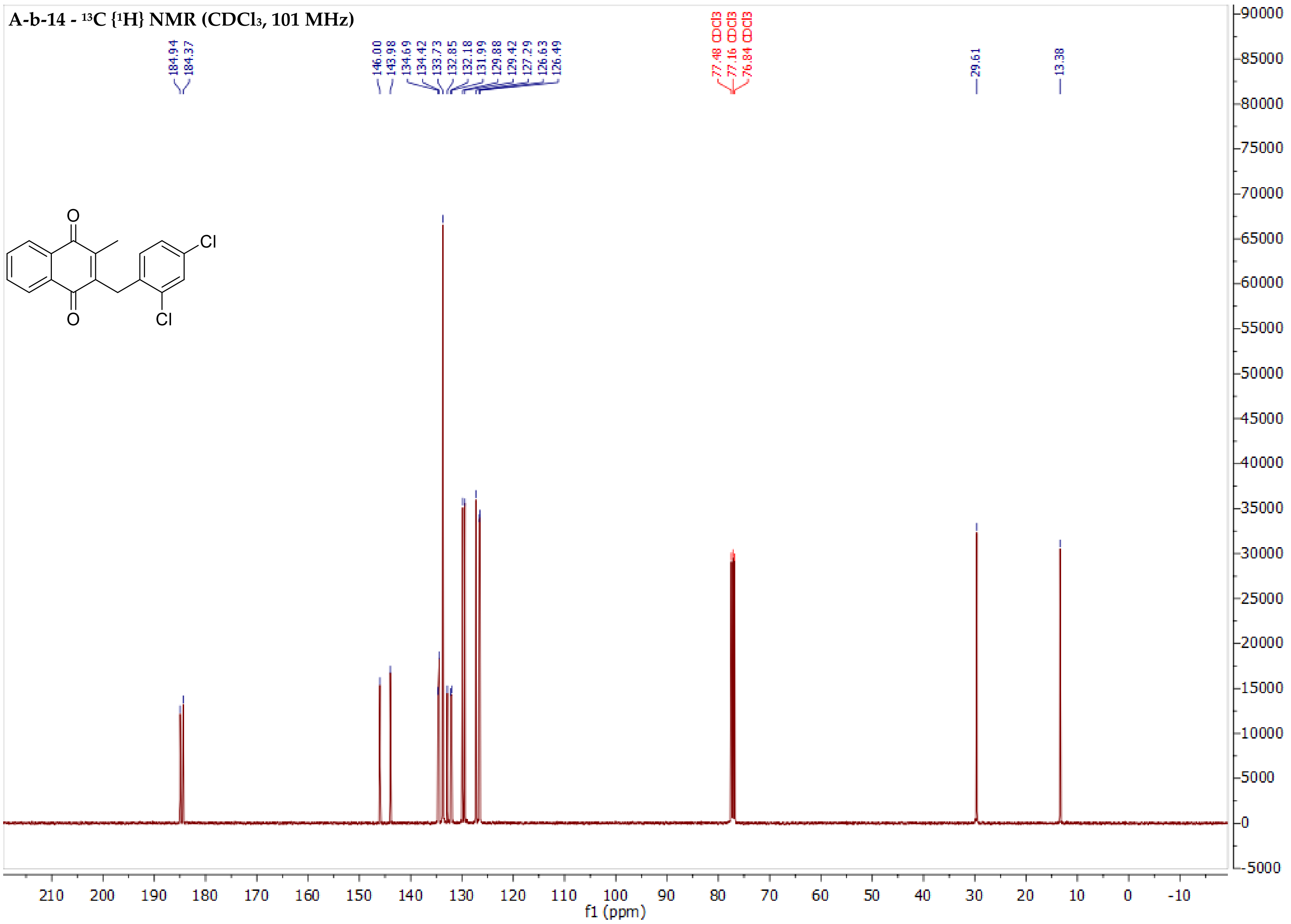
A-b-14 - 13C {1H} NMR (CDCl3, 101 MHz)
184.94
184.37
146.00
143.98
134.69
134.42
133.73
132.85
132.18
131.99
129.88
129.42
127.29
126.63
126.49
77.48 CDCl3
77.16 CDCl3
76.84 CDCl3
29.61
13.38
O
O
Cl
Cl
f1 (ppm)

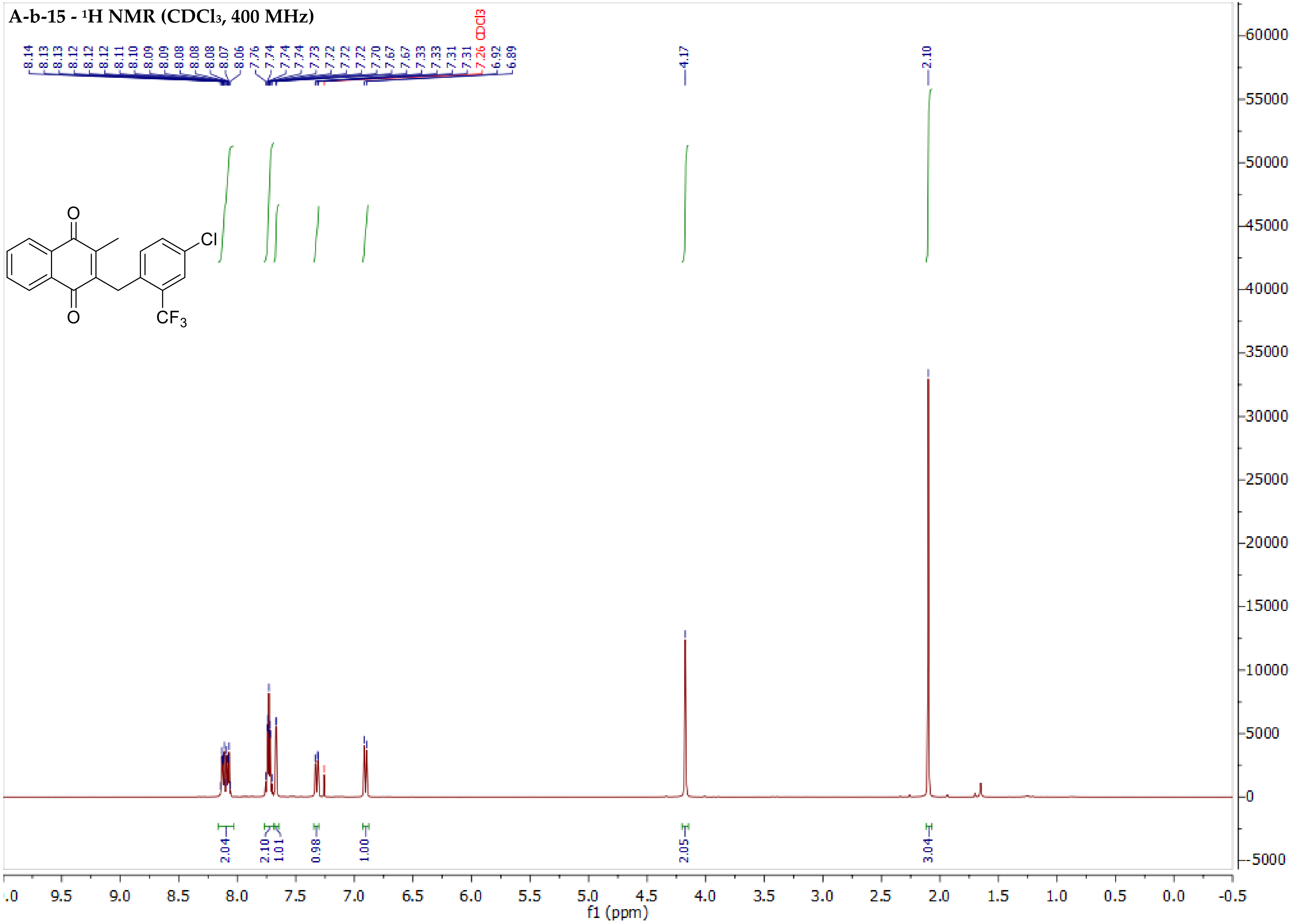
A-b-15 - ¹H NMR (CDCl₃, 400 MHz)
8.14 8.13 8.13 8.12 8.12 8.12 8.11 8.10 8.09 8.09 8.08 8.08 8.07 8.06 7.76 7.74 7.74 7.74 7.73 7.72 7.72 7.72 7.70 7.67 7.67 7.33 7.33 7.31 7.31 7.26 CDCl3 6.92 6.89
4.17
2.10
O
O
Cl
CF3
2.04
2.10
1.01
0.98
1.00
2.05
3.04
f1 (ppm)

A-b-15 - $^{13}C\{^1H\}$ NMR ($CDCl_3$, 101 MHz)

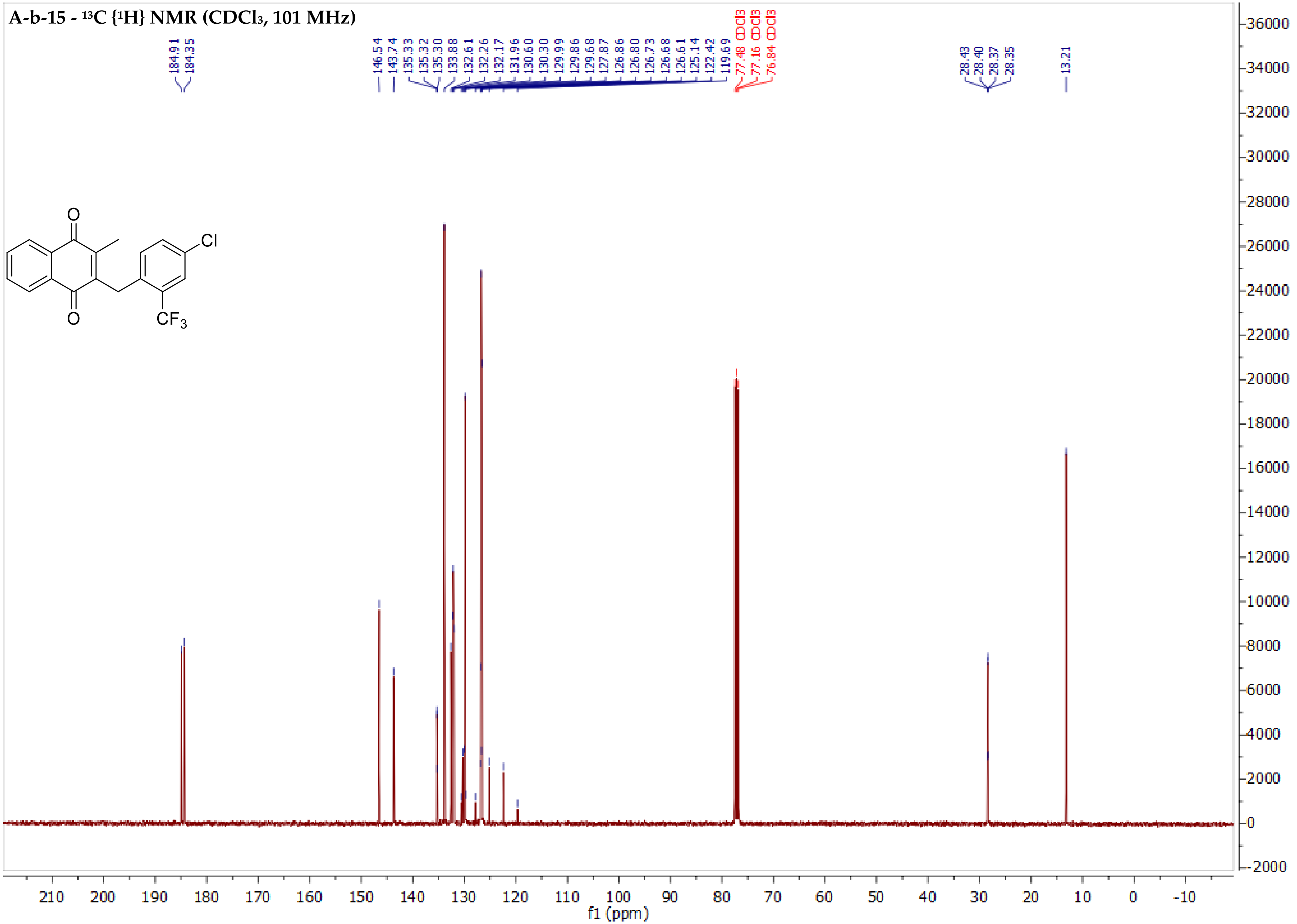

A-b-15 - ^{19}F NMR ($CDCl_3$, 377 MHz)

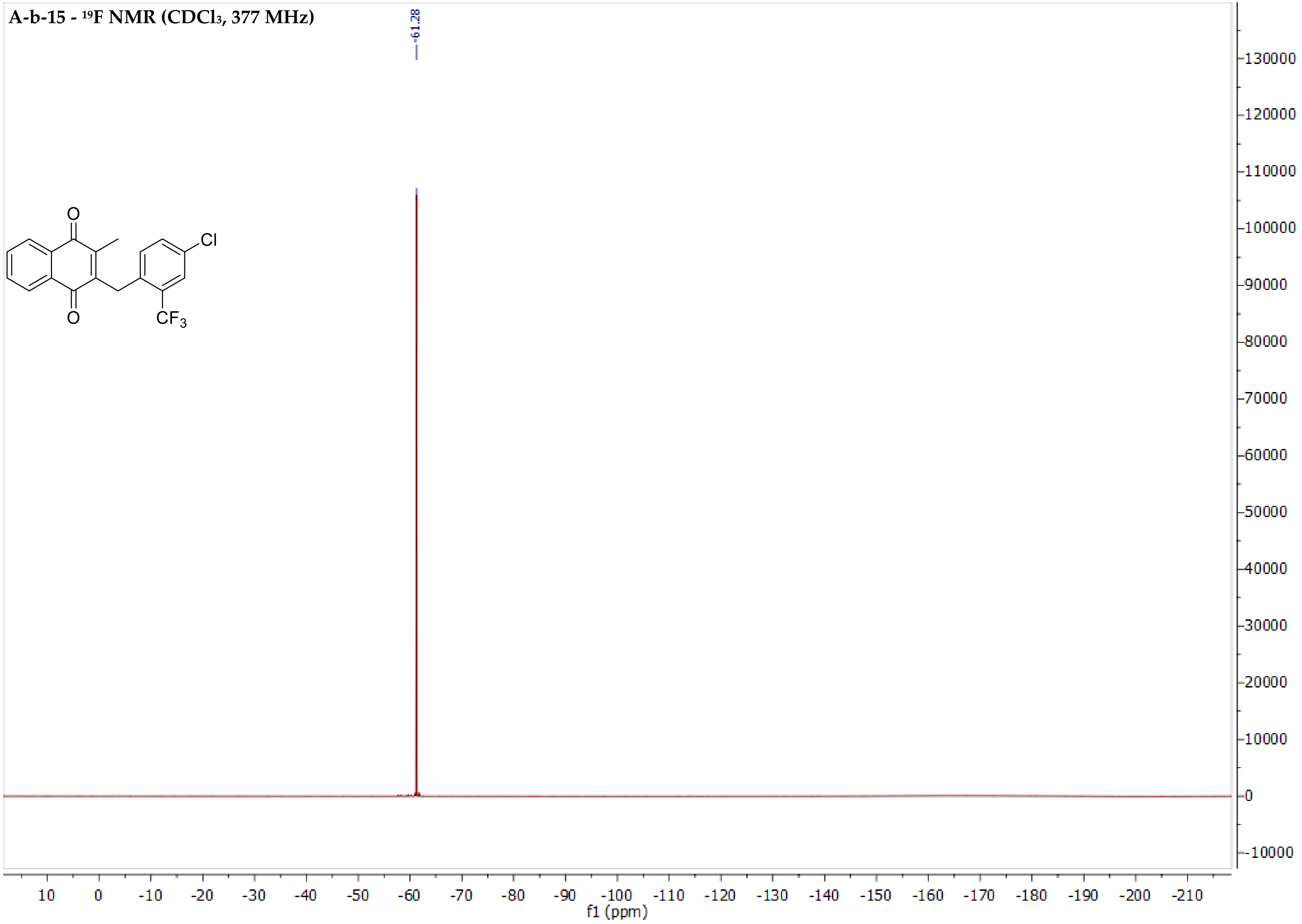

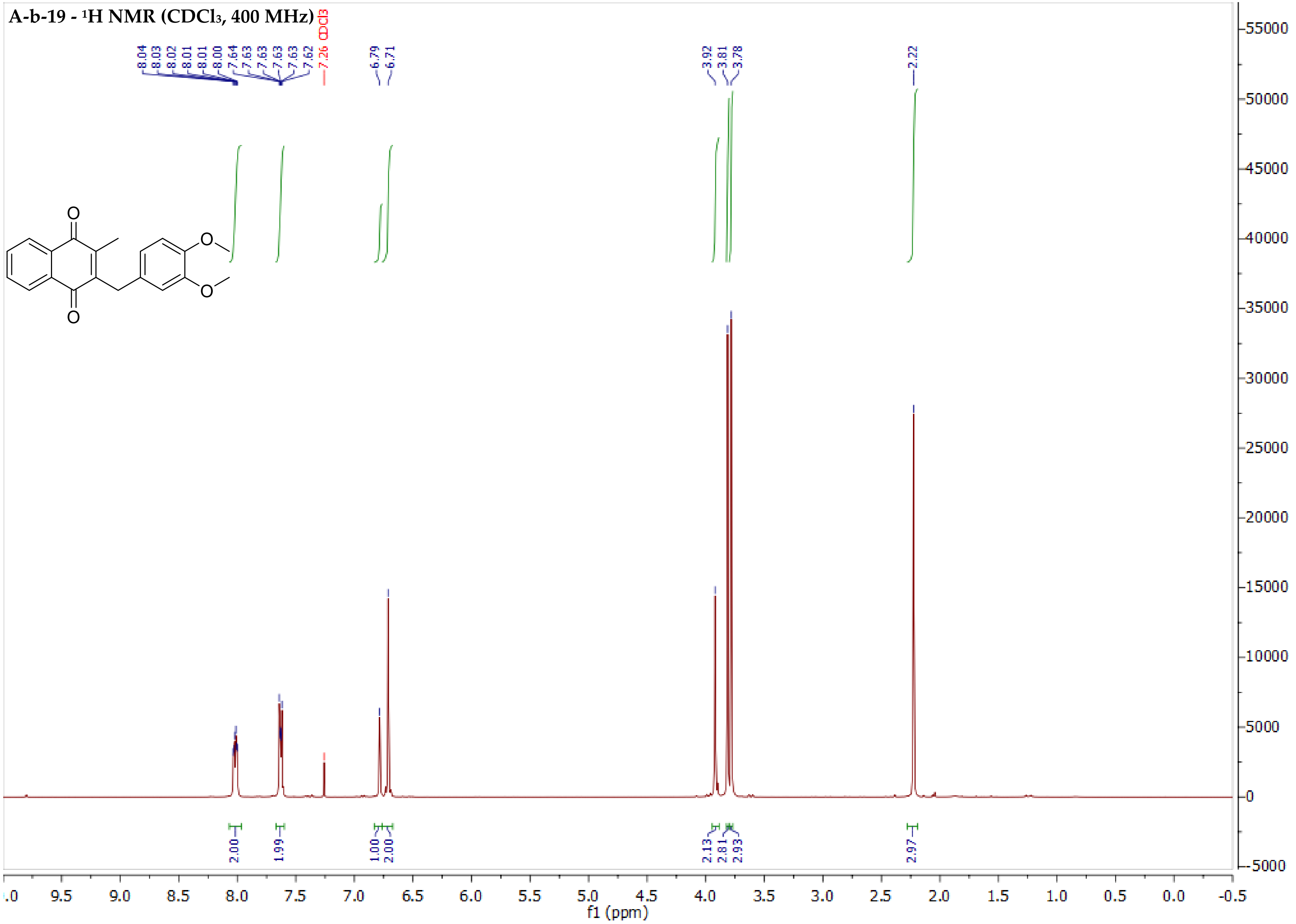
A-b-19 - ¹H NMR (CDCl₃, 400 MHz)
CDCl3
8.04
8.03
8.02
8.01
8.01
8.00
7.64
7.63
7.63
7.63
7.63
7.62
7.26
6.79
6.71
3.92
3.81
3.78
2.22
O
O
O
O
2.00
1.99
1.00
2.00
2.13
2.81
2.93
2.97
f1 (ppm)

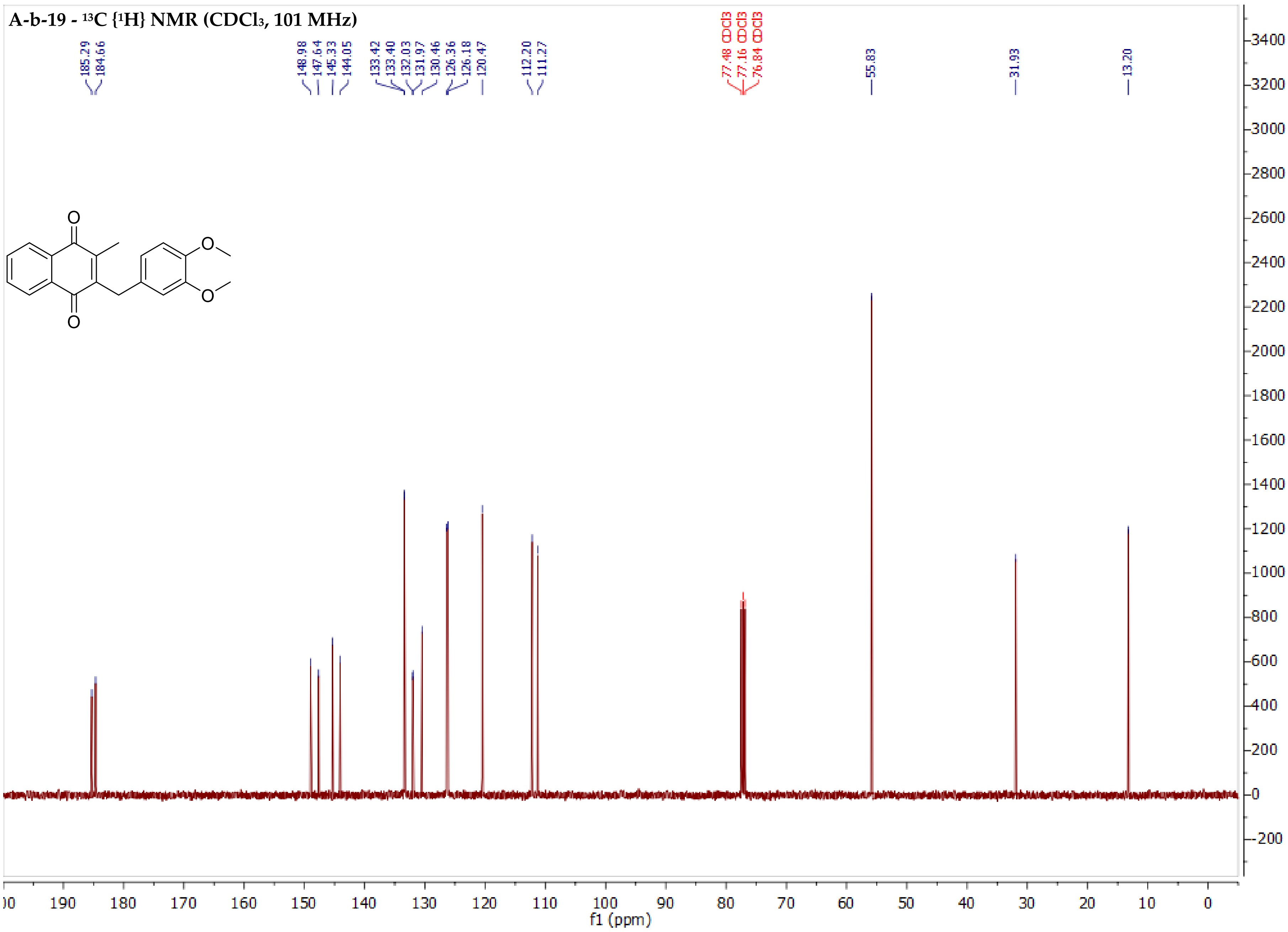
A-b-19 - 13C {1H} NMR (CDCl3, 101 MHz)
185.29
184.66
148.98
147.64
145.33
144.05
133.42
133.40
132.03
131.97
130.46
126.36
126.18
120.47
112.20
111.27
77.48 CDCl3
77.16 CDCl3
76.84 CDCl3
55.83
31.93
13.20
O
O
O
O
f1 (ppm)

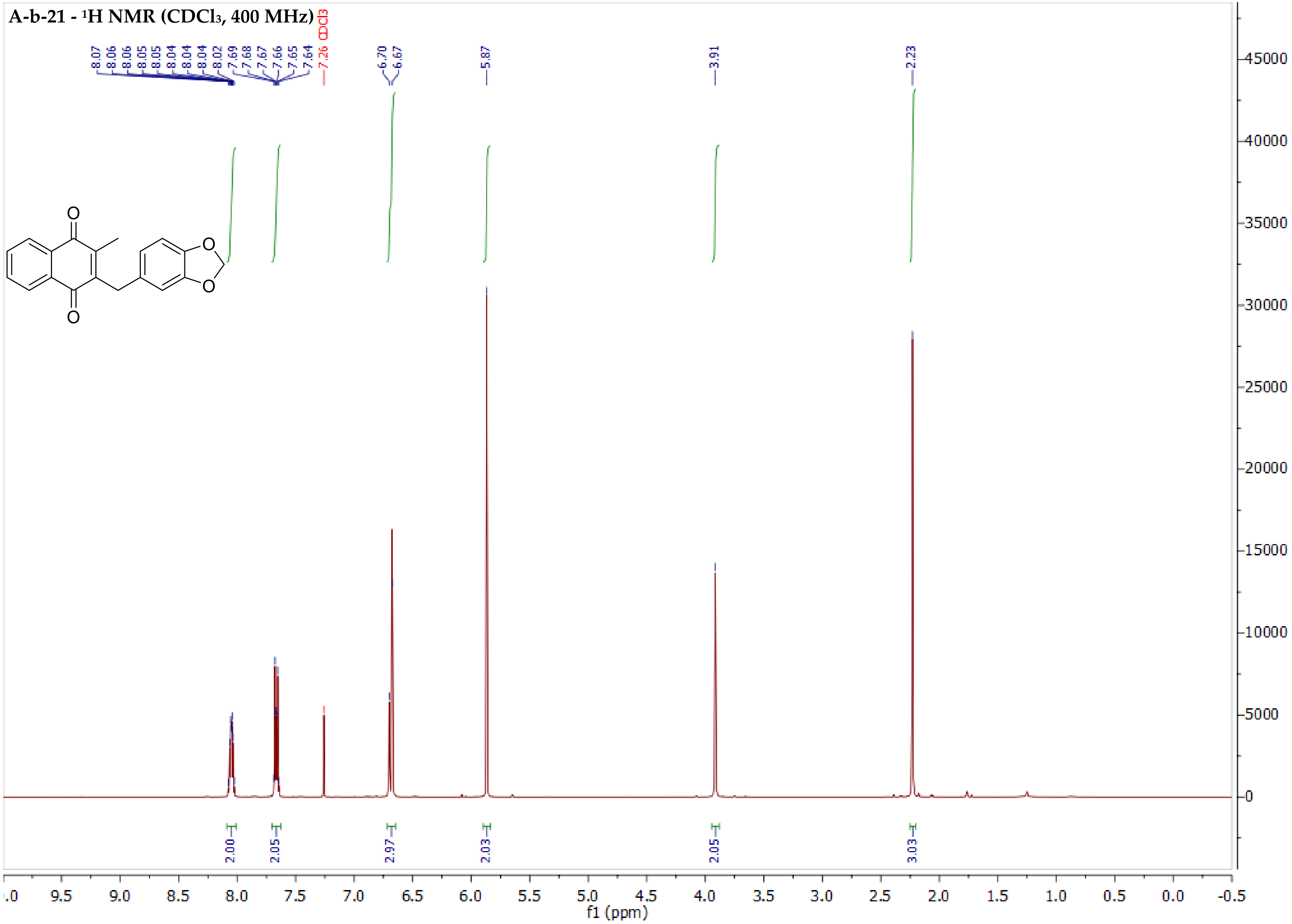
A-b-21 - ¹H NMR (CDCl₃, 400 MHz)
8.07 8.06 8.06 8.05 8.05 8.04 8.04 8.04 8.02 7.69 7.68 7.67 7.66 7.65 7.64 7.26 CDCl3
6.70 6.67
5.87
3.91
2.23
2.00 2.05 2.97 2.03 2.05 3.03
f1 (ppm)

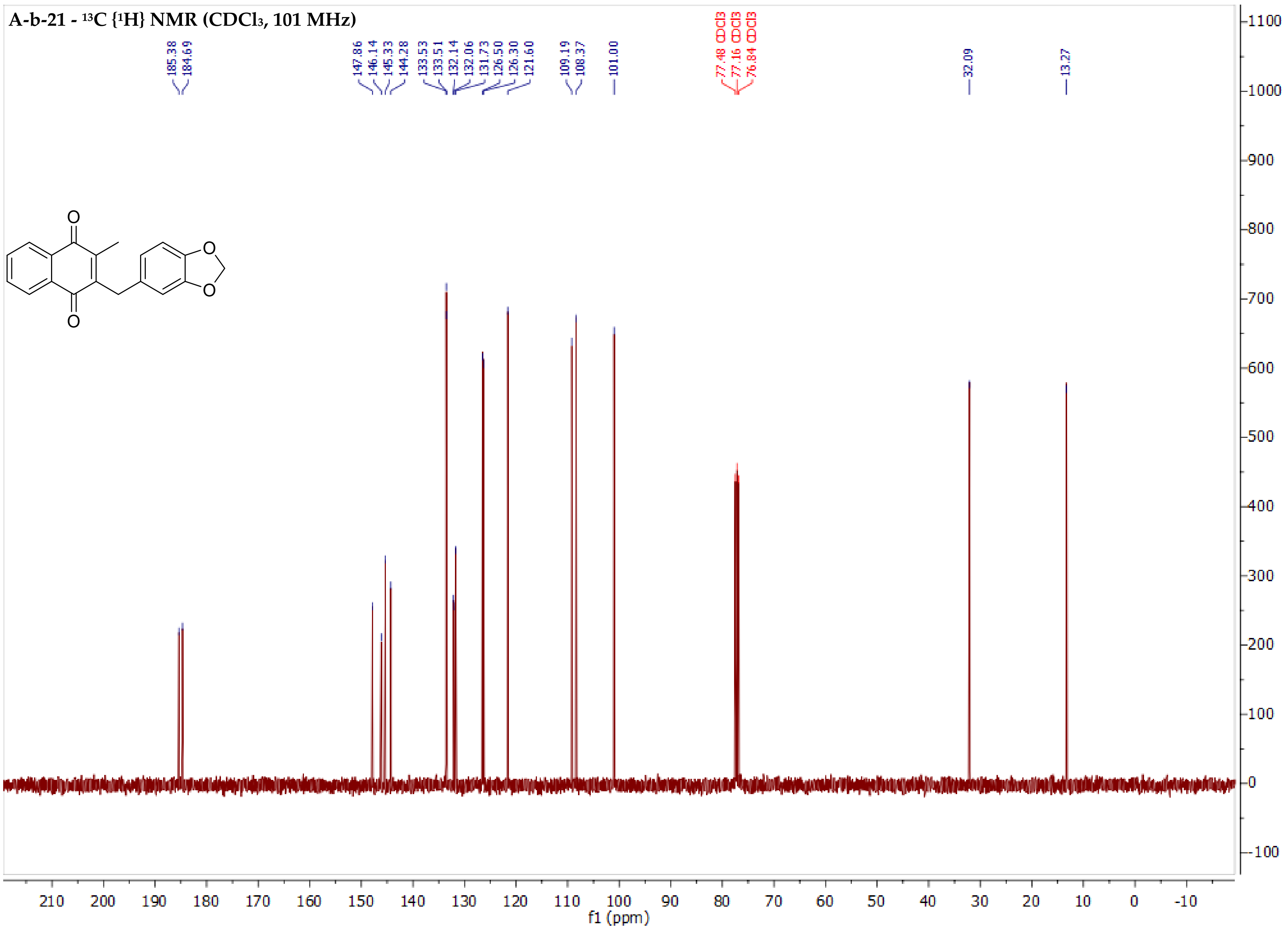
A-b-21 - 13C {1H} NMR (CDCl3, 101 MHz)
185.38
184.69
147.86
146.14
145.33
144.28
133.53
133.51
132.14
132.06
131.73
126.50
126.30
121.60
109.19
108.37
101.00
77.48 CDCl3
77.16 CDCl3
76.84 CDCl3
32.09
13.27
O
O
O
O
f1 (ppm)

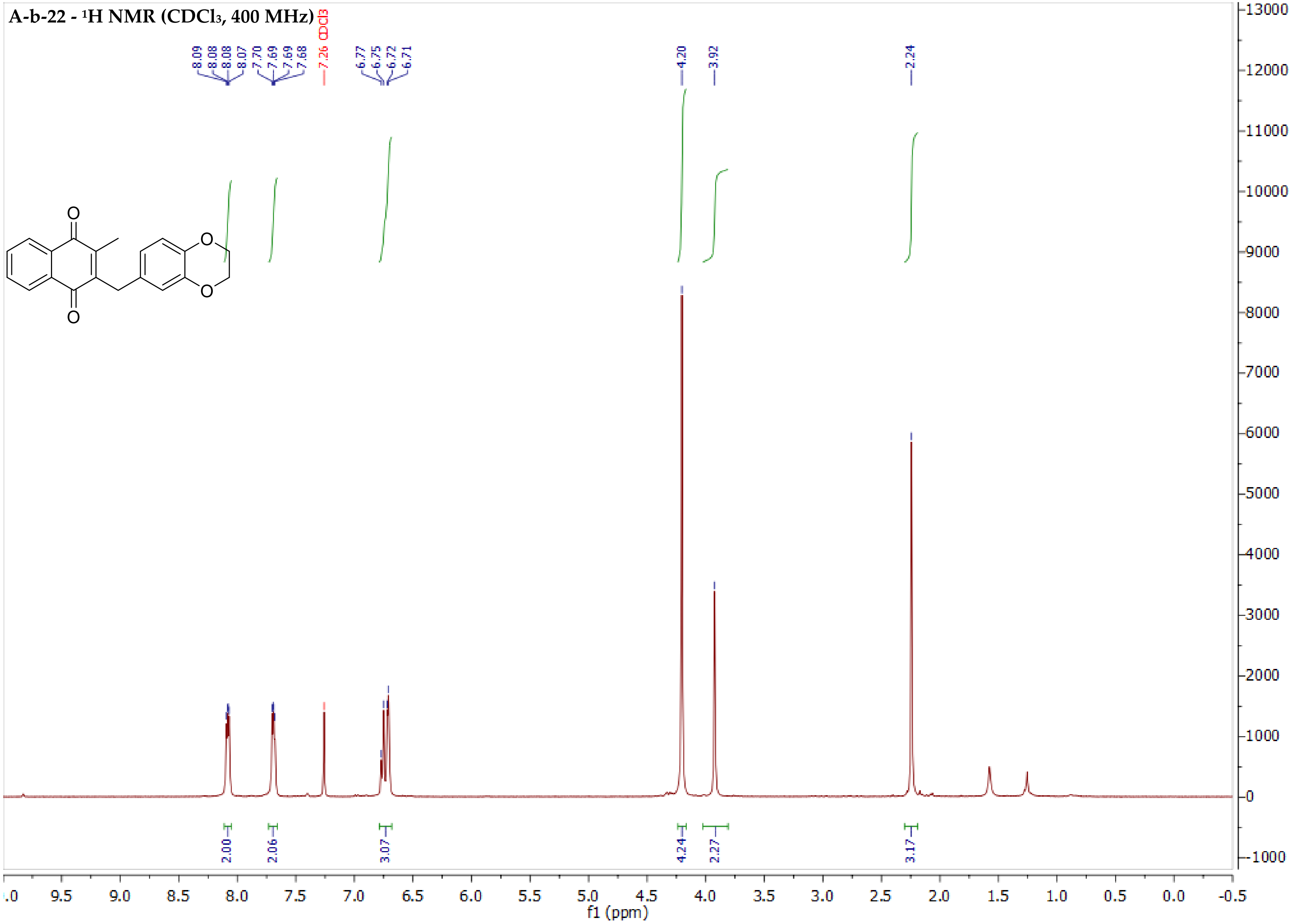
A-b-22 - ¹H NMR (CDCl₃, 400 MHz)
8.09
8.08
8.08
8.07
7.70
7.69
7.69
7.68
7.26 CDCl3
6.77
6.75
6.72
6.71
4.20
3.92
2.24
2.00
2.06
3.07
4.24
2.27
3.17
f1 (ppm)

A-b-22 - $^{13}C\{^1H\}$ NMR ($CDCl_3$, 101 MHz)

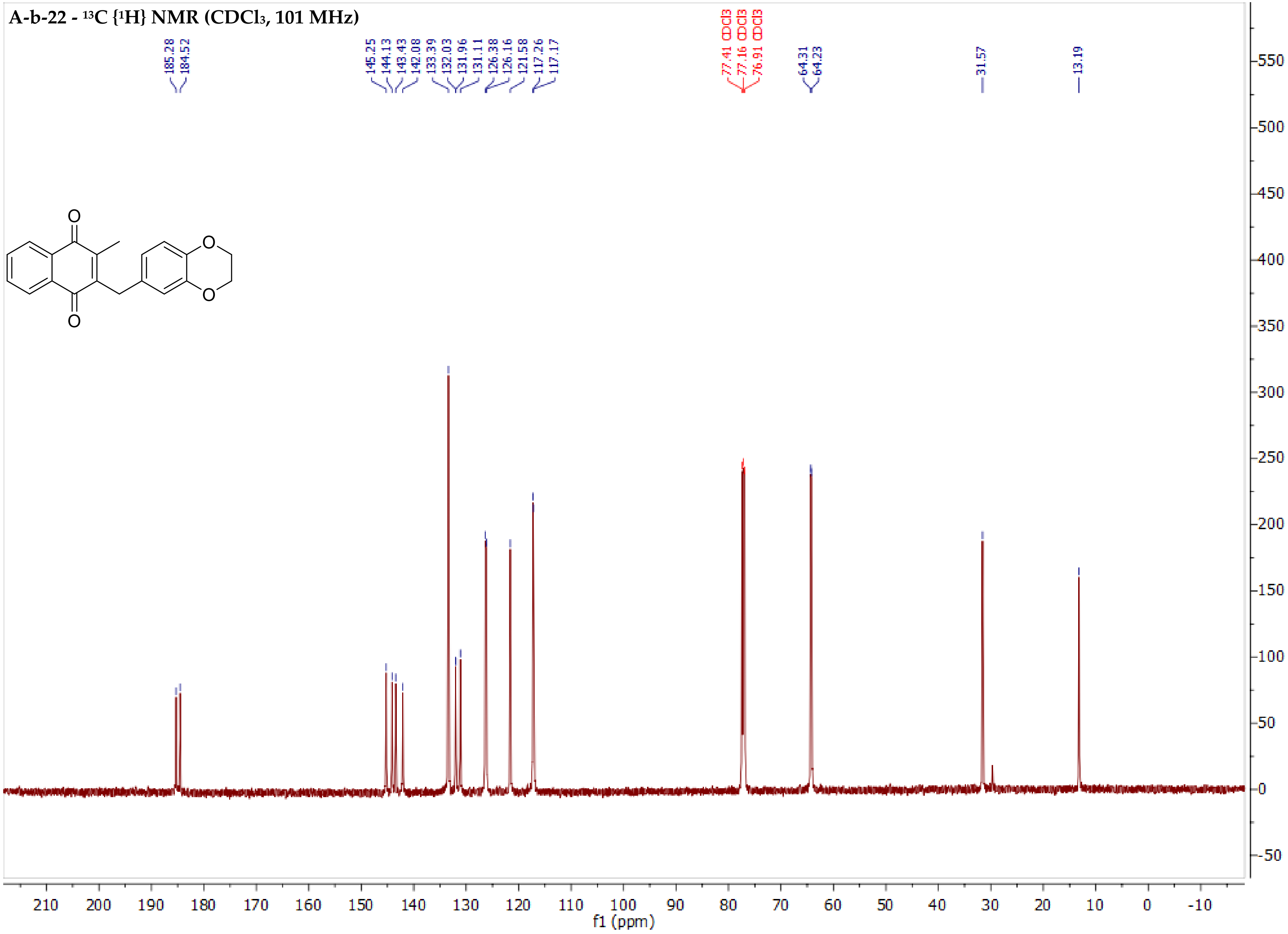

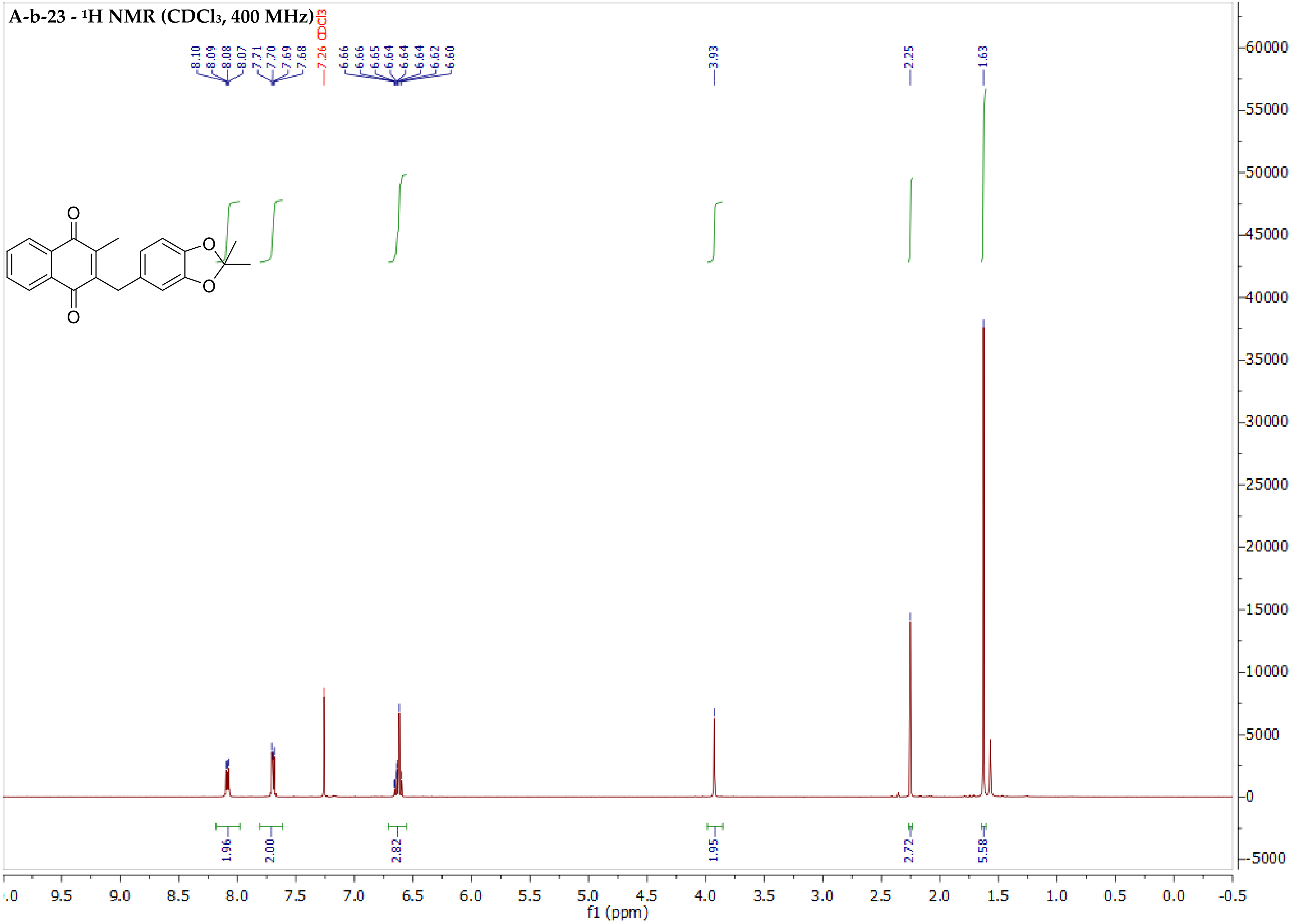

A-b-23 - 1H NMR (CDCl3, 400 MHz)
CDCl3
8.10 8.09 8.08 8.07 7.71 7.70 7.69 7.68
7.26
6.66 6.66 6.65 6.64 6.64 6.64 6.62 6.60
3.93
2.25
1.63
O
O
O
O
1.96
2.00
2.82
1.95
2.72
5.58
60000
55000
50000
45000
40000
35000
30000
25000
20000
15000
10000
5000
0
-5000
9.5 9.0 8.5 8.0 7.5 7.0 6.5 6.0 5.5 5.0 4.5 4.0 3.5 3.0 2.5 2.0 1.5 1.0 0.5 0.0 -0.5
f1 (ppm)

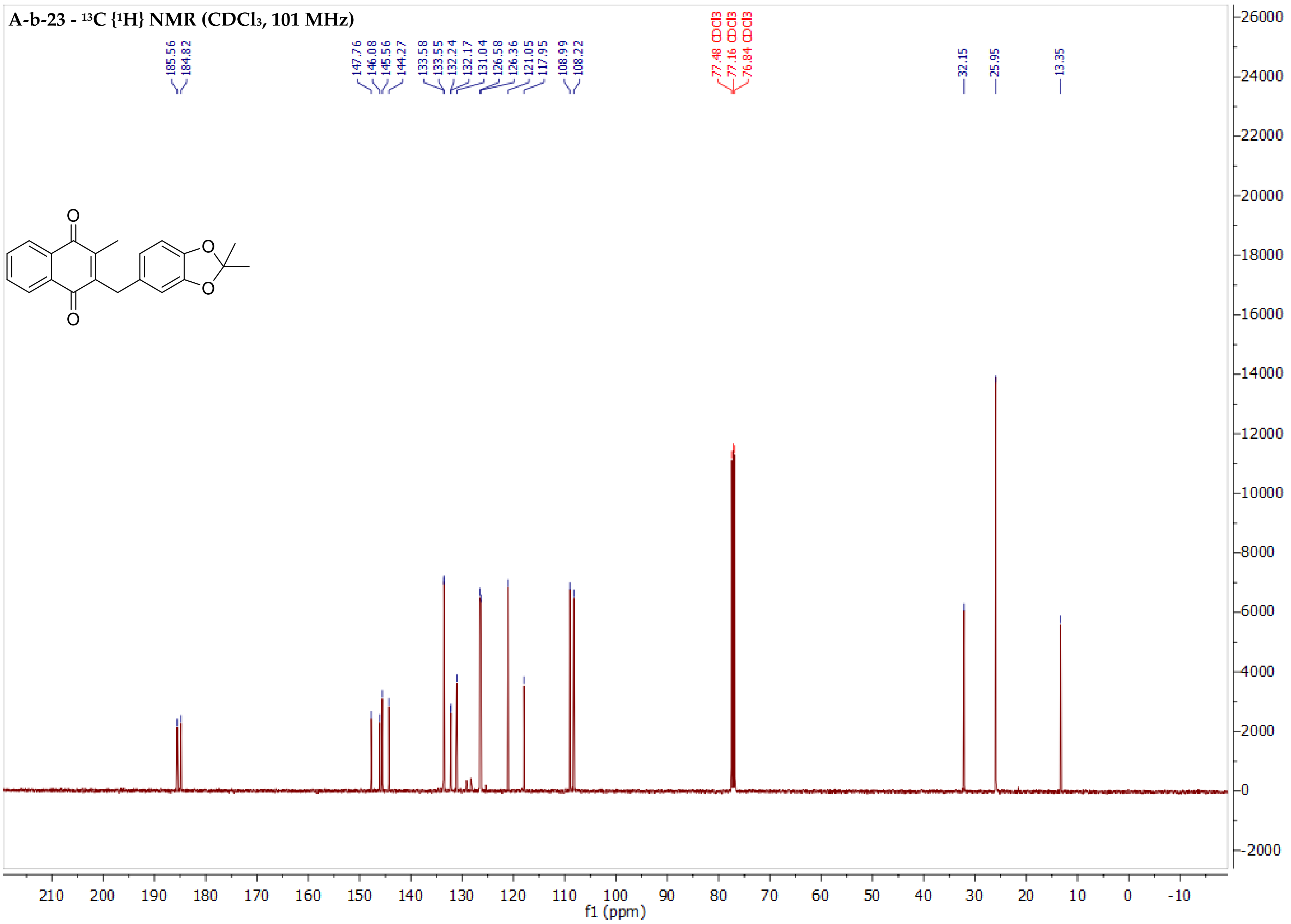

A-b-23 - 13C {1H} NMR (CDCl3, 101 MHz)
185.56
184.82
147.76
146.08
145.56
144.27
133.58
133.55
132.24
132.17
131.04
126.58
126.36
121.05
117.95
108.99
108.22
77.48 CDCl3
77.16 CDCl3
76.84 CDCl3
32.15
25.95
13.35
f1 (ppm)

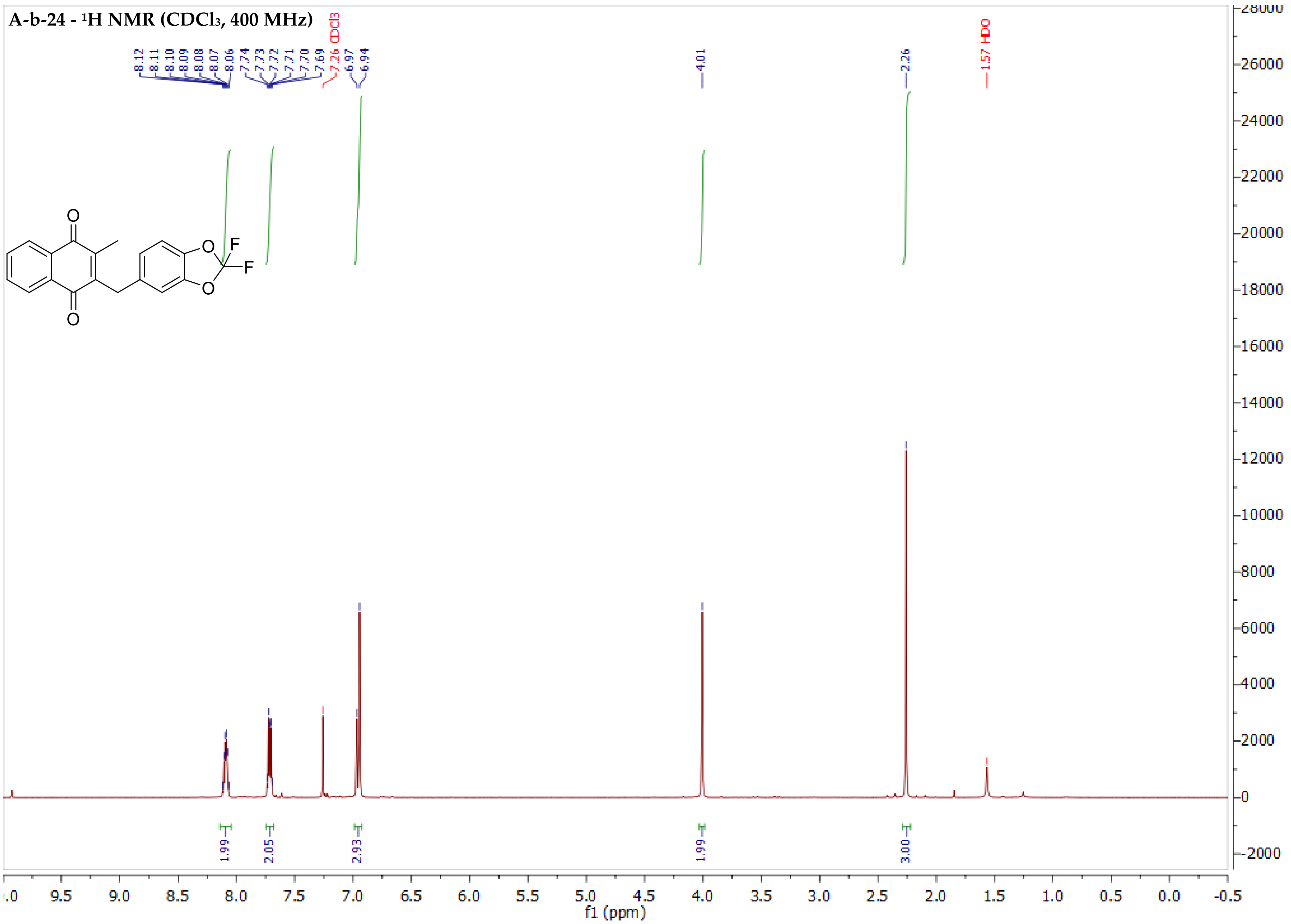
A-b-24 - 1H NMR (CDCl3, 400 MHz)
8.12
8.11
8.10
8.09
8.08
8.07
8.06
7.74
7.73
7.72
7.71
7.70
7.69
7.26 CDCl3
6.97
6.94
4.01
2.26
1.57 HDO
O
O
O
O
F
F
1.99
2.05
2.93
1.99
3.00
f1 (ppm)

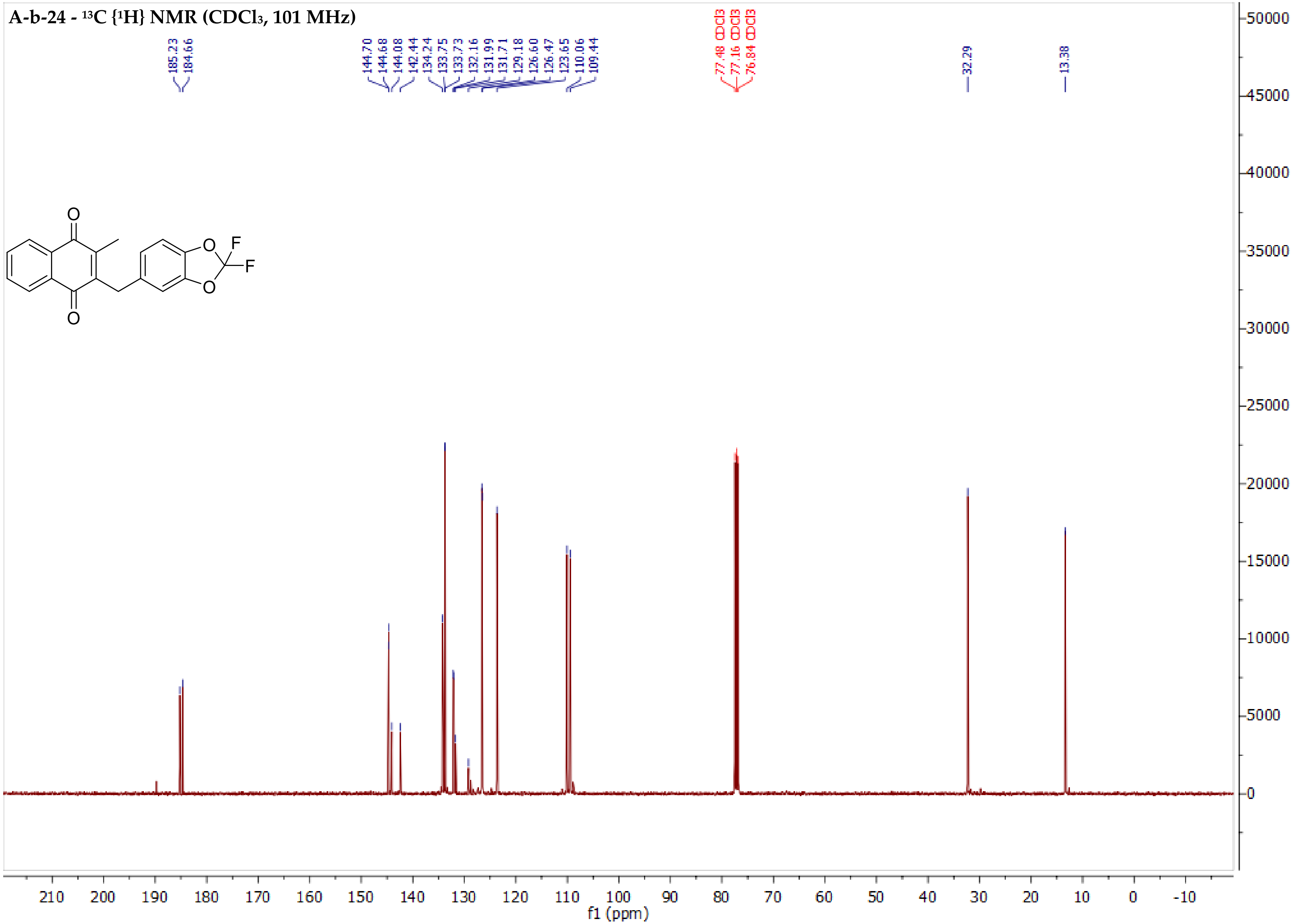
A-b-24 - ^{13}C {^{1}H} NMR ($CDCl_3$, 101 MHz)
185.23
184.66
144.70
144.68
144.08
142.44
134.24
133.75
133.73
132.16
131.99
131.71
129.18
126.60
126.47
123.65
110.06
109.44
77.48 CDCl3
77.16 CDCl3
76.84 CDCl3
32.29
13.38
f1 (ppm)

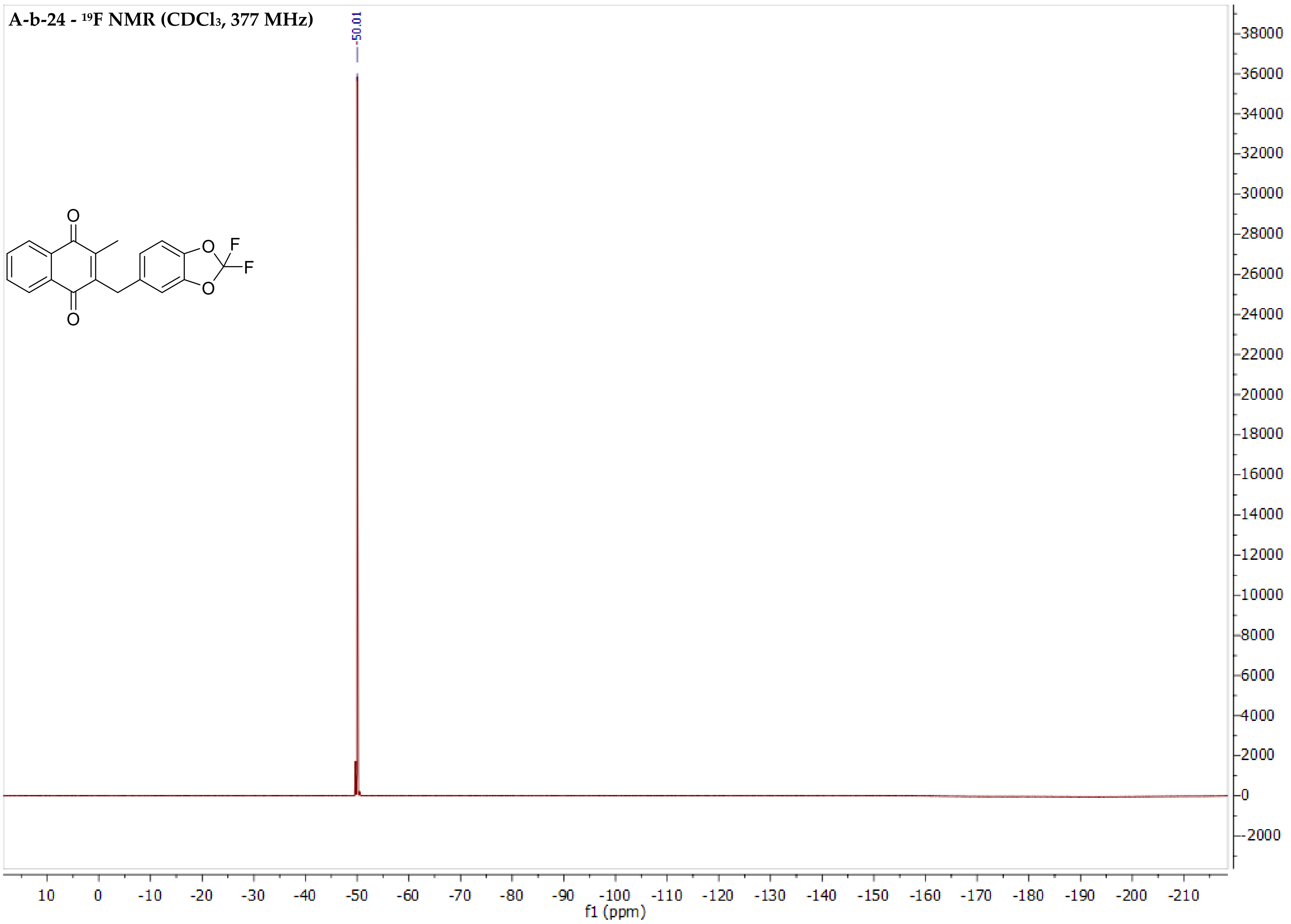
A-b-24 - 19F NMR (CDCl3, 377 MHz)
-50.01
O
O
O
O
F
F
38000
36000
34000
32000
30000
28000
26000
24000
22000
20000
18000
16000
14000
12000
10000
8000
6000
4000
2000
0
-2000
10 0 -10 -20 -30 -40 -50 -60 -70 -80 -90 -100 -110 -120 -130 -140 -150 -160 -170 -180 -190 -200 -210
f1 (ppm)

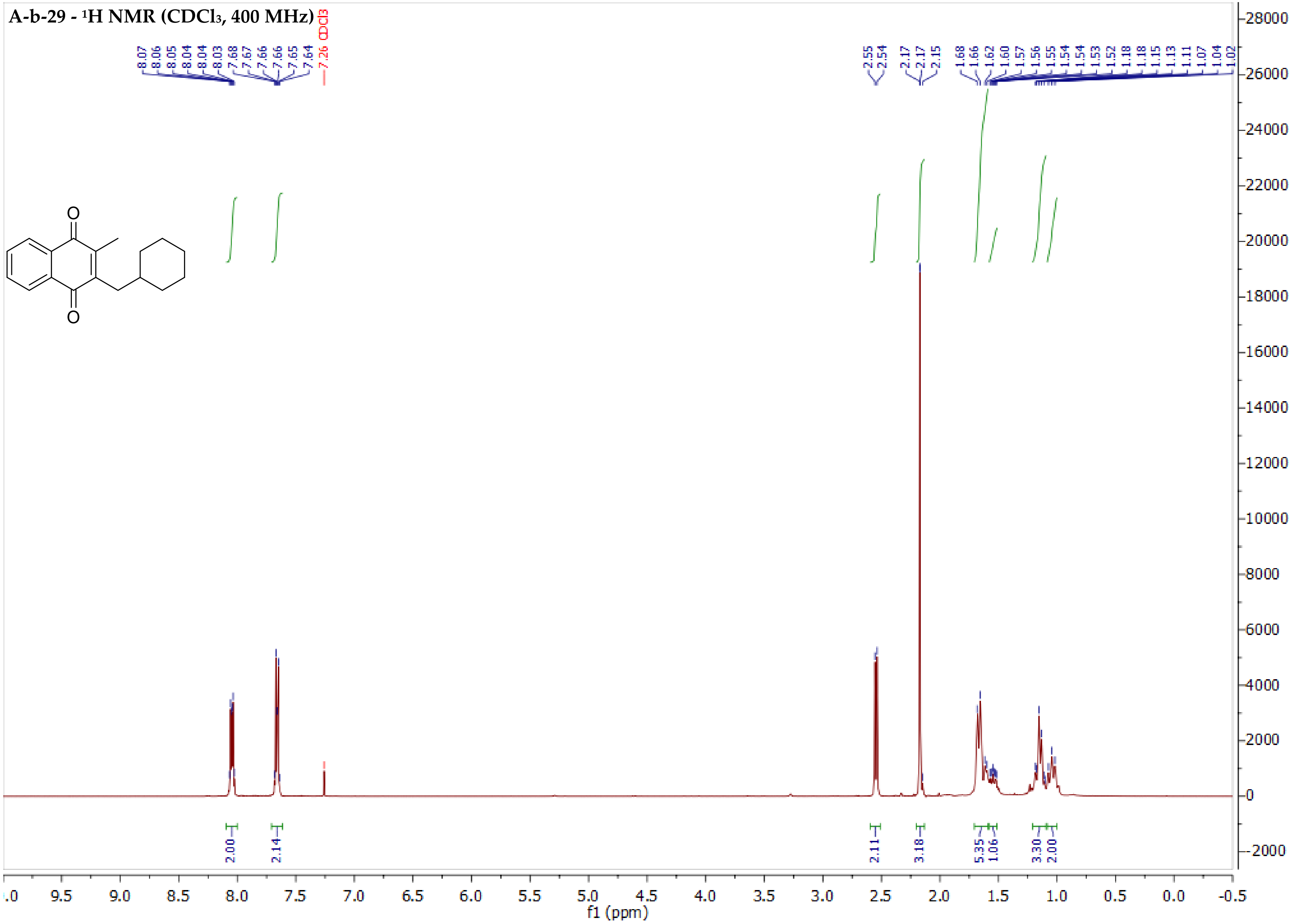
A-b-29 - ¹H NMR (CDCl₃, 400 MHz)
8.07 8.06 8.05 8.04 8.04 8.03 7.68 7.67 7.66 7.66 7.65 7.64
7.26 CDCl3
2.55 2.54 2.17 2.17 2.15 1.68 1.66 1.62 1.60 1.57 1.56 1.55 1.54 1.54 1.53 1.52 1.18 1.18 1.15 1.13 1.11 1.07 1.04 1.02
2.00 2.14 2.11 3.18 5.35 1.06 3.30 2.00
O
O
f1 (ppm)

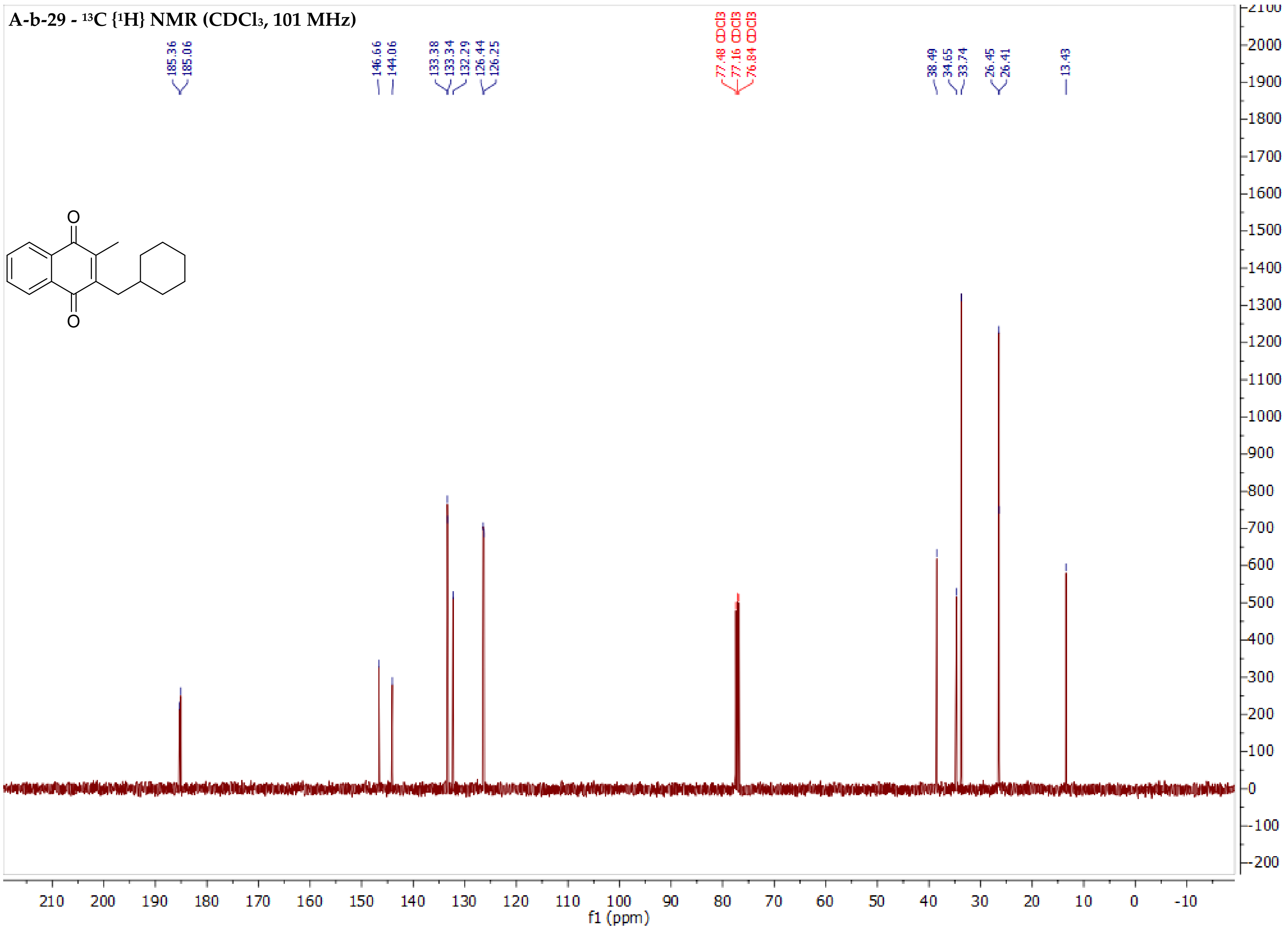

A-b-29 - ^{13}C {^{1}H} NMR ($CDCl_3$, 101 MHz)
185.36
185.06
146.66
144.06
133.38
133.34
132.29
126.44
126.25
77.48 CDCl3
77.16 CDCl3
76.84 CDCl3
38.49
34.65
33.74
26.45
26.41
13.43
O
O
210 200 190 180 170 160 150 140 130 120 110 100 90 80 70 60 50 40 30 20 10 0 -10
f1 (ppm)
2100 2000 1900 1800 1700 1600 1500 1400 1300 1200 1100 1000 900 800 700 600 500 400 300 200 100 0 -100 -200

B-b-30 - ^{1}H NMR ($CDCl_3$, 400 MHz)

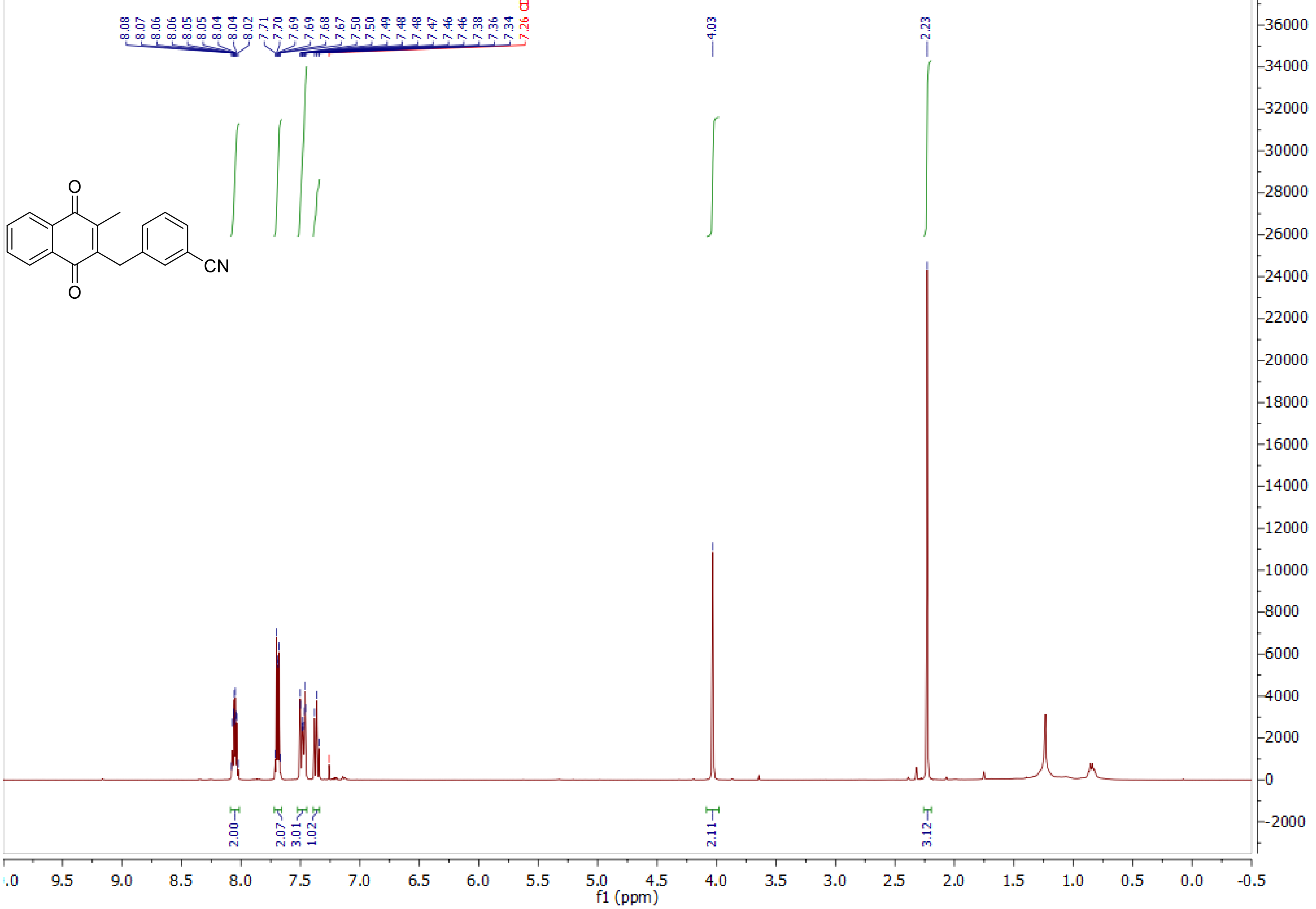

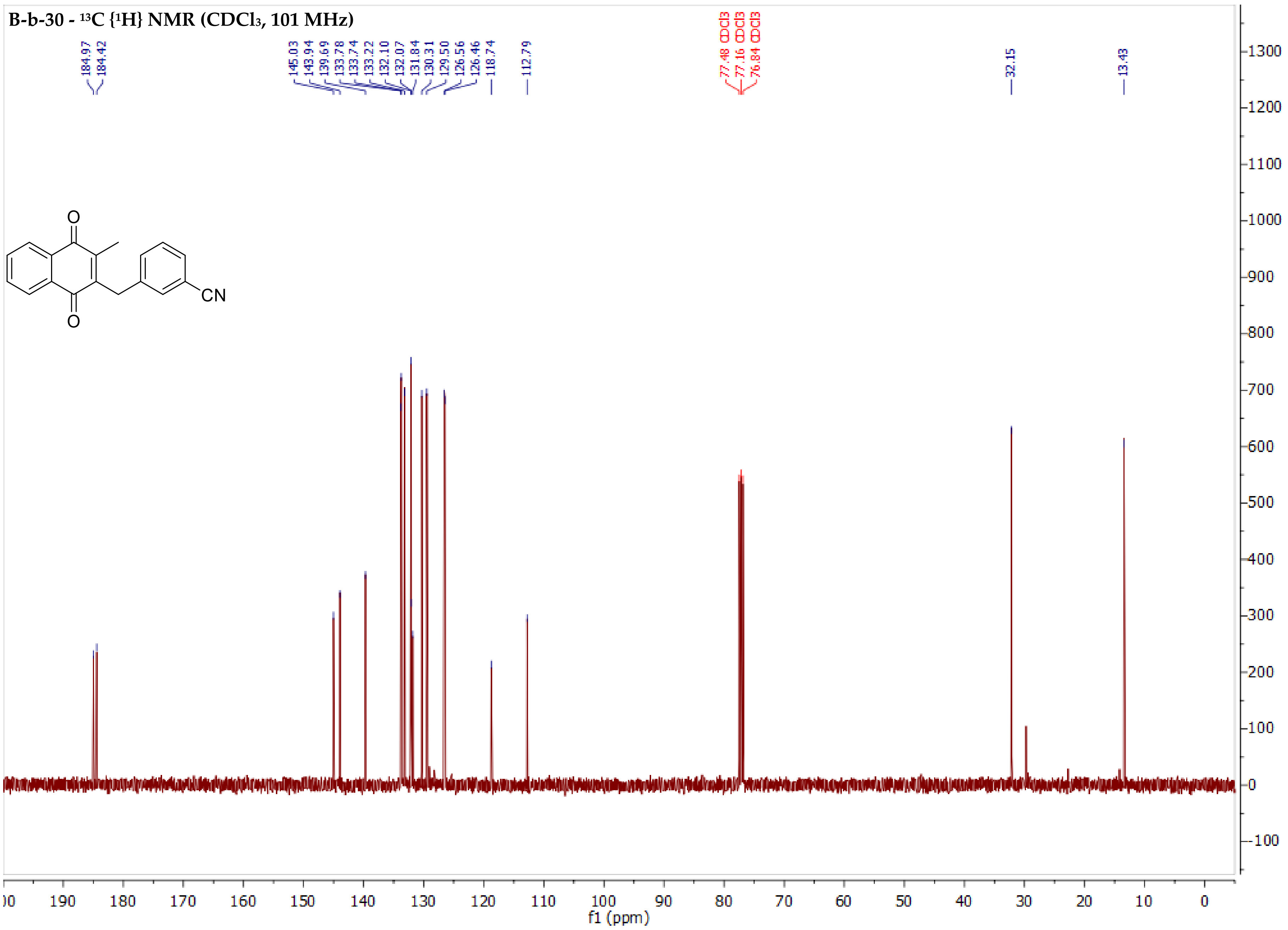
B-b-30 - ¹³C {¹H} NMR (CDCl₃, 101 MHz)
184.97
184.42
145.03
143.94
139.69
133.78
133.74
133.22
132.10
132.07
131.84
130.31
129.50
126.56
126.46
118.74
112.79
77.48 CDCl3
77.16 CDCl3
76.84 CDCl3
32.15
13.43
O
O
CN
f1 (ppm)

B-b-33 - ^{1}H NMR ($CDCl_3$, 400 MHz)

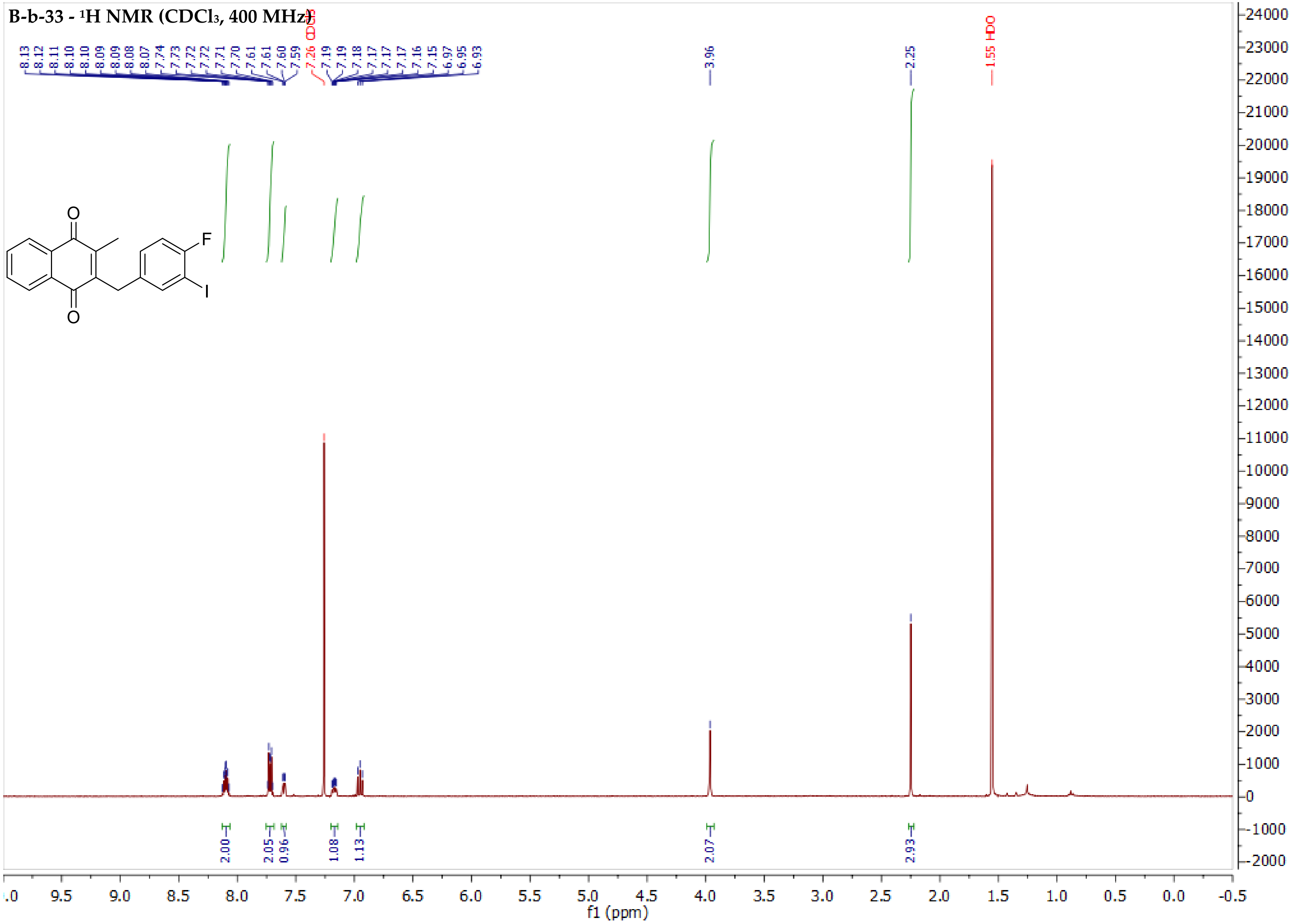

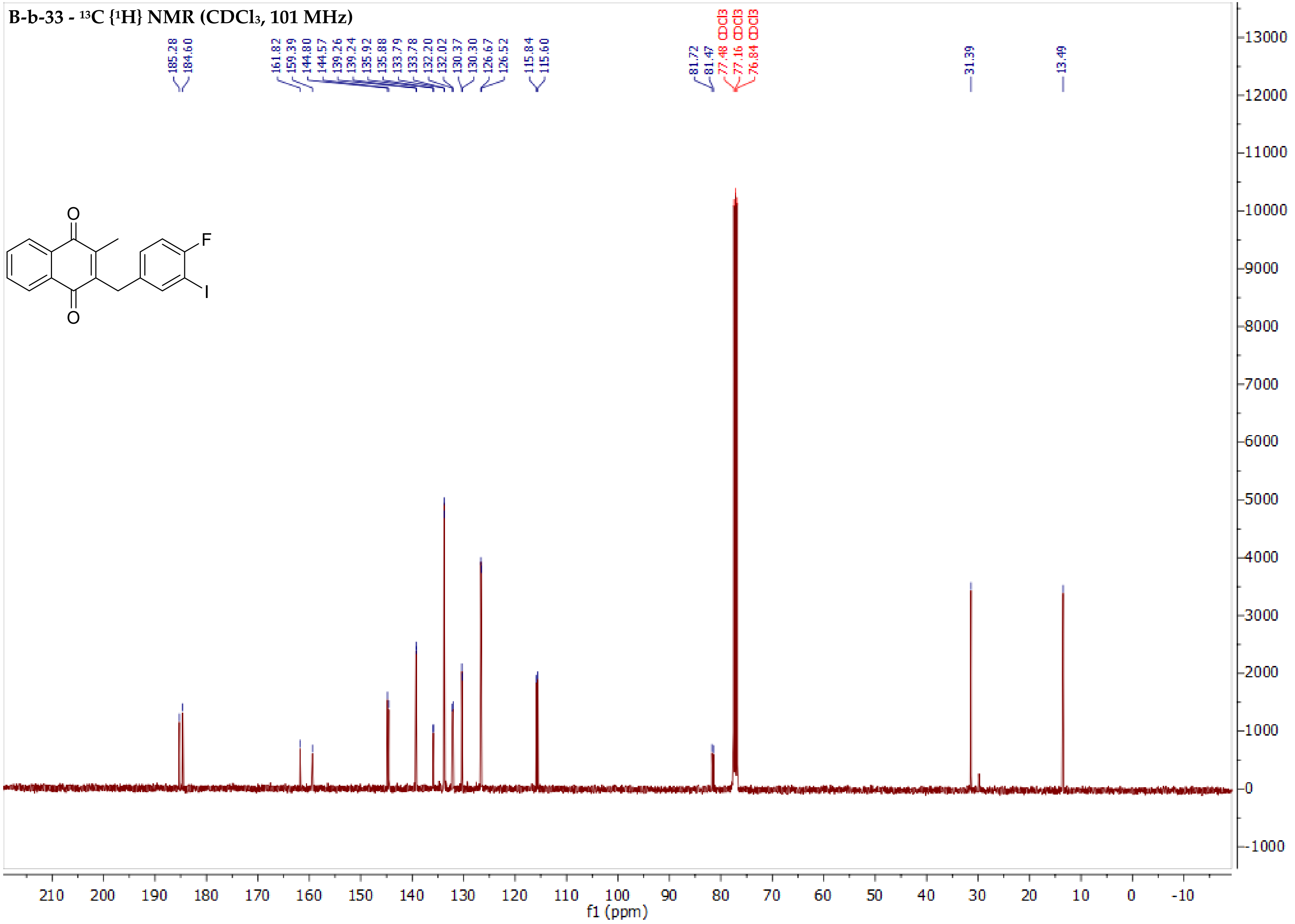
B-b-33 - 13C {1H} NMR (CDCl3, 101 MHz)
185.28
184.60
161.82
159.39
144.80
144.57
139.26
139.24
135.92
135.88
133.79
133.78
132.20
132.02
130.37
130.30
126.67
126.52
115.84
115.60
81.72
81.47
77.48 CDCl3
77.16 CDCl3
76.84 CDCl3
31.39
13.49
O
O
F
I
f1 (ppm)

B-b-33 - ^{19}F NMR (CDCl$_3$, 377 MHz)

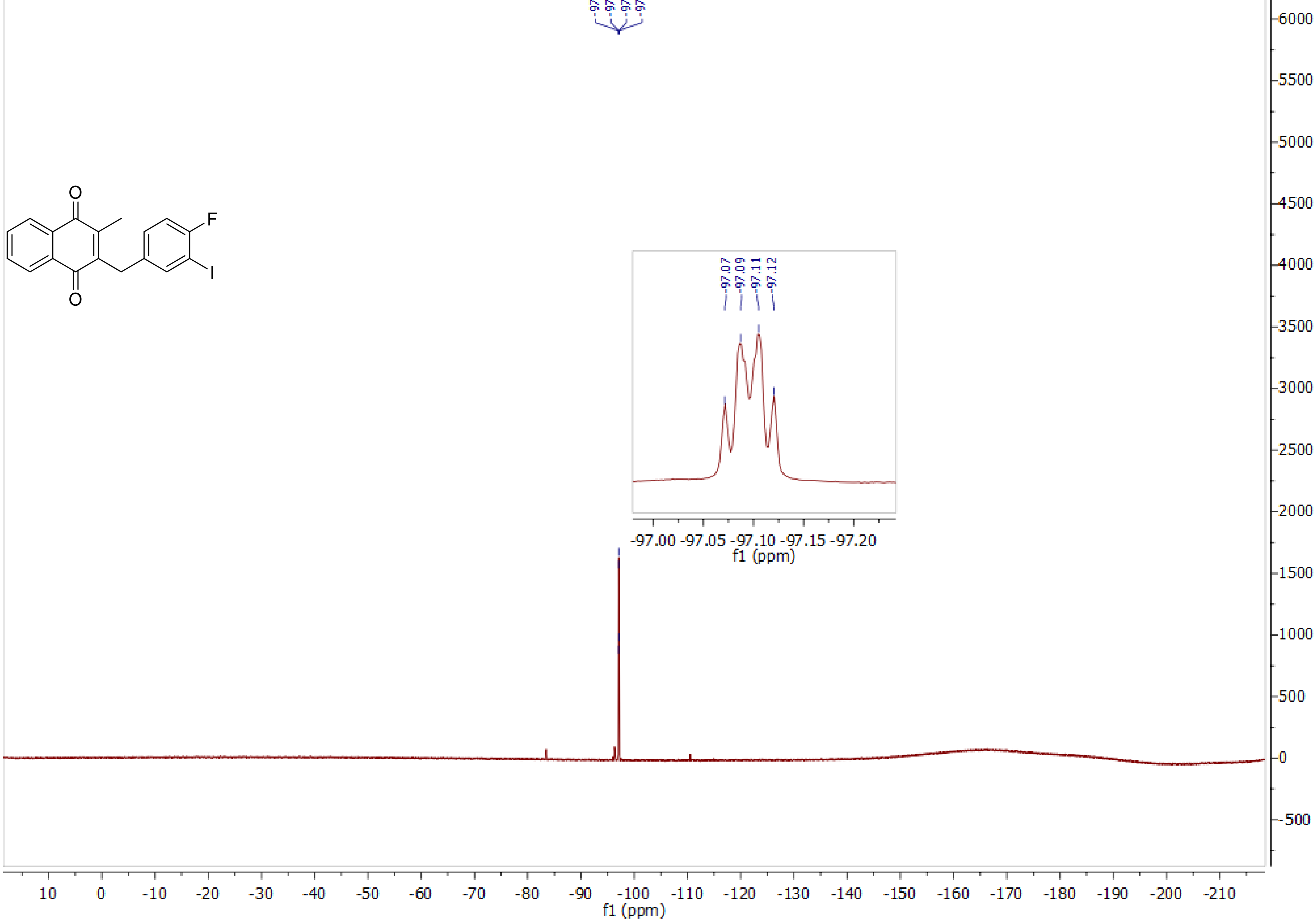

B-b-34 - ^1H NMR ($CDCl_3$, 400 MHz)

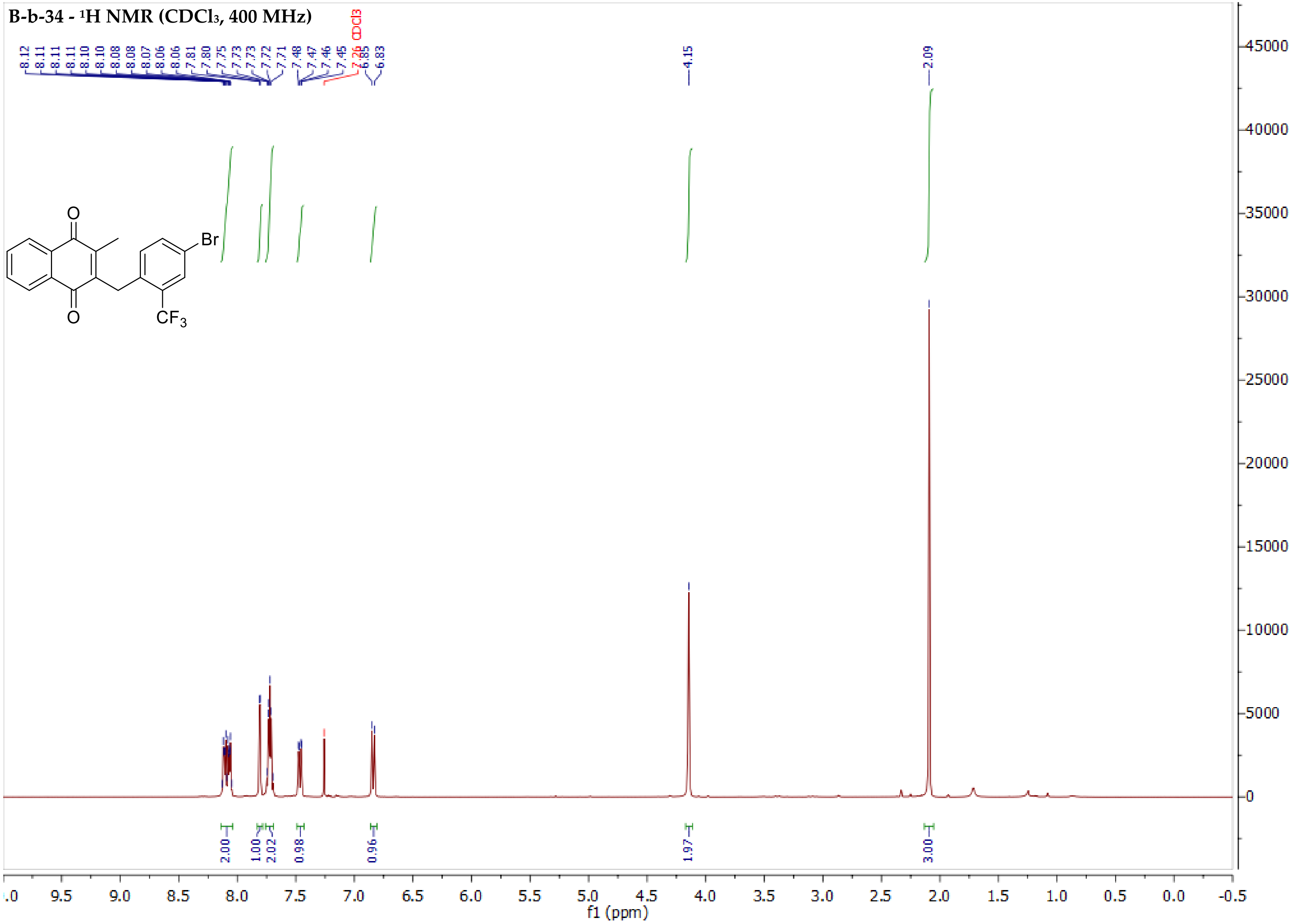

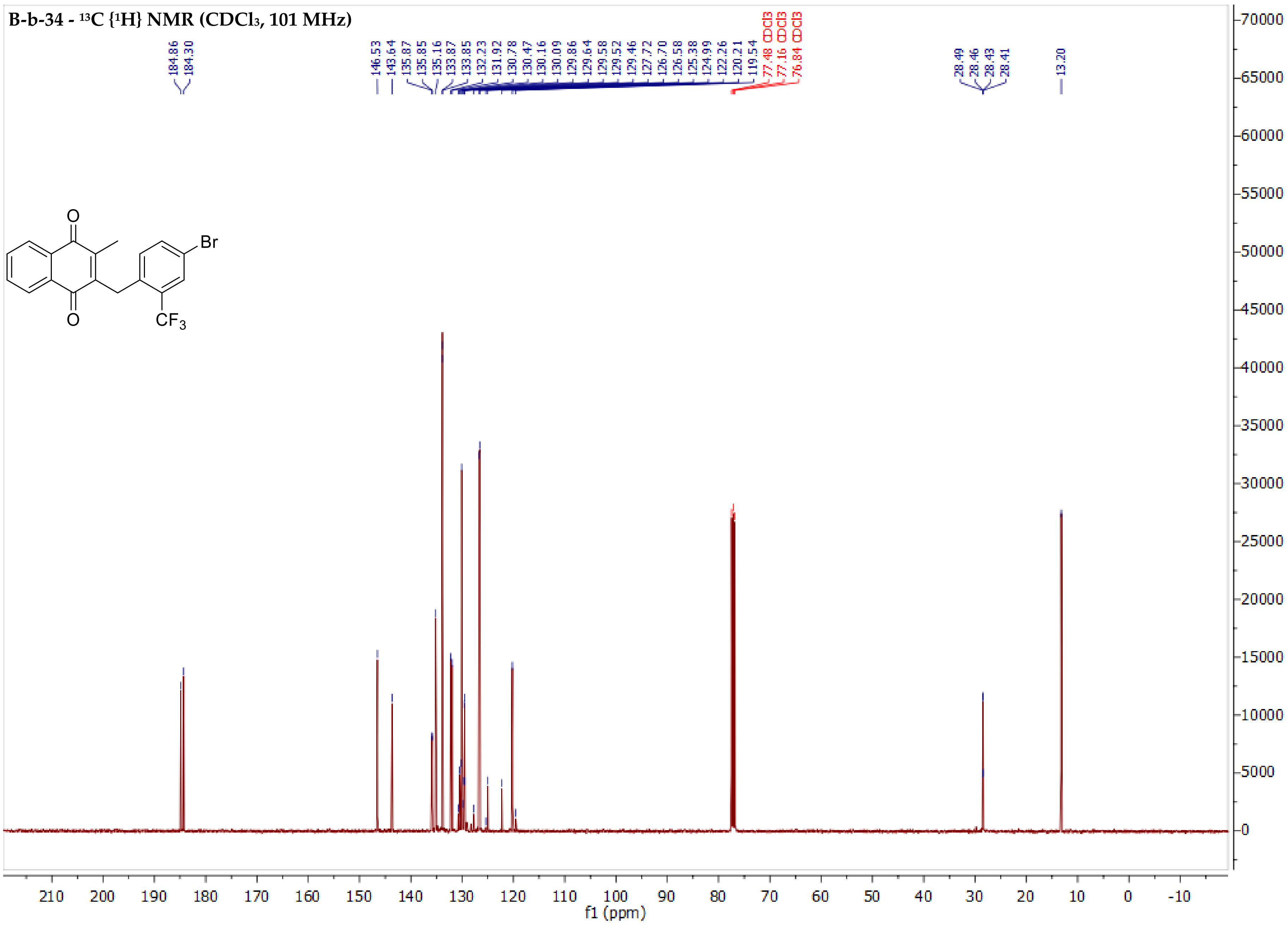
B-b-34 - ¹³C {¹H} NMR (CDCl₃, 101 MHz)
184.86
184.30
146.53
143.64
135.87
135.85
135.16
133.87
133.85
132.23
131.92
130.78
130.47
130.16
130.09
129.86
129.64
129.58
129.52
129.46
127.72
126.70
126.58
125.38
124.99
122.26
120.21
119.54
77.48 CDCl3
77.16 CDCl3
76.84 CDCl3
28.49
28.46
28.43
28.41
13.20
O
O
Br
CF3
f1 (ppm)

B-b-34 - ^{19}F NMR ($CDCl_3$, 377 MHz)

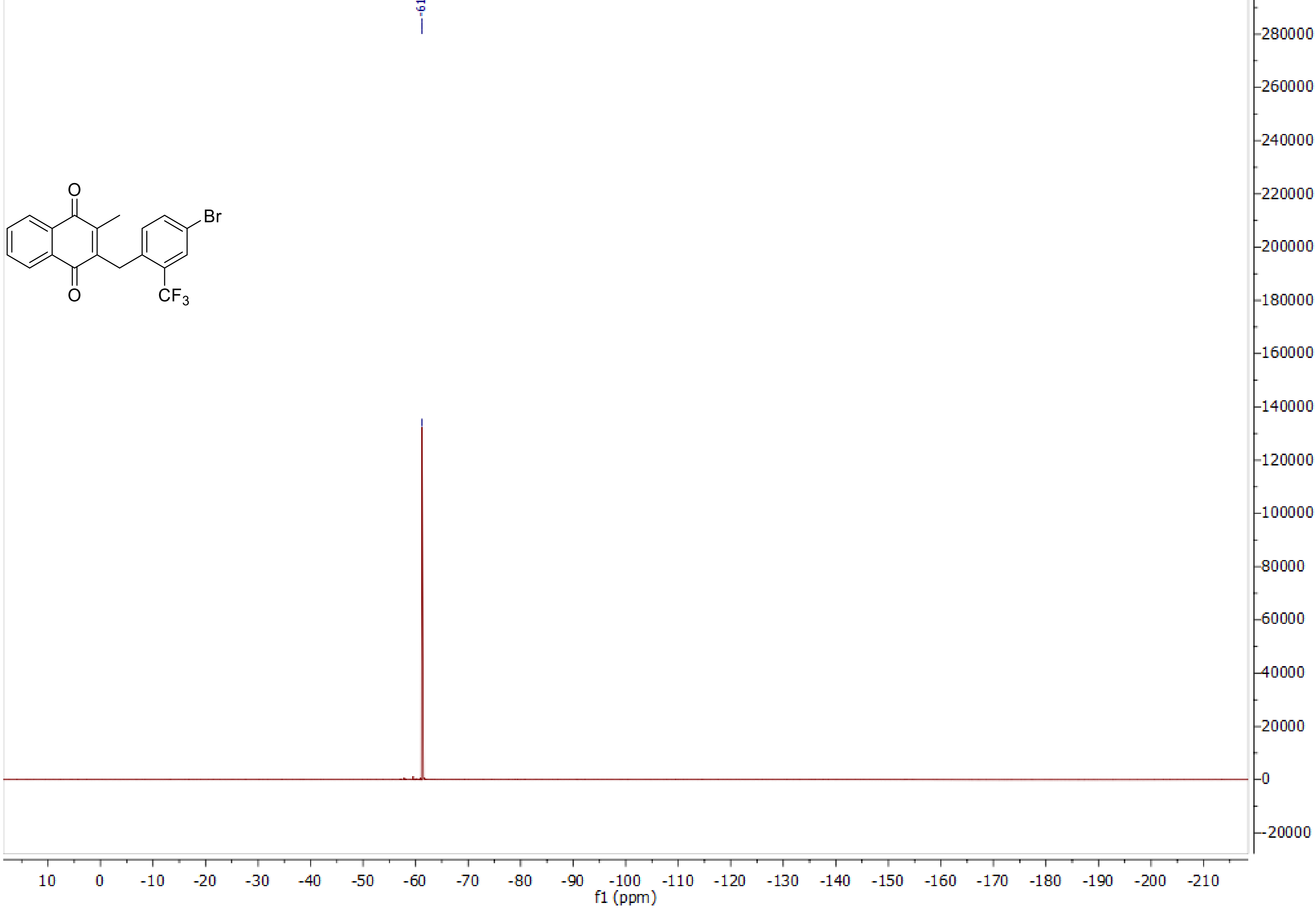

44 - ^{1}H NMR ($CDCl_3$, 400 MHz)

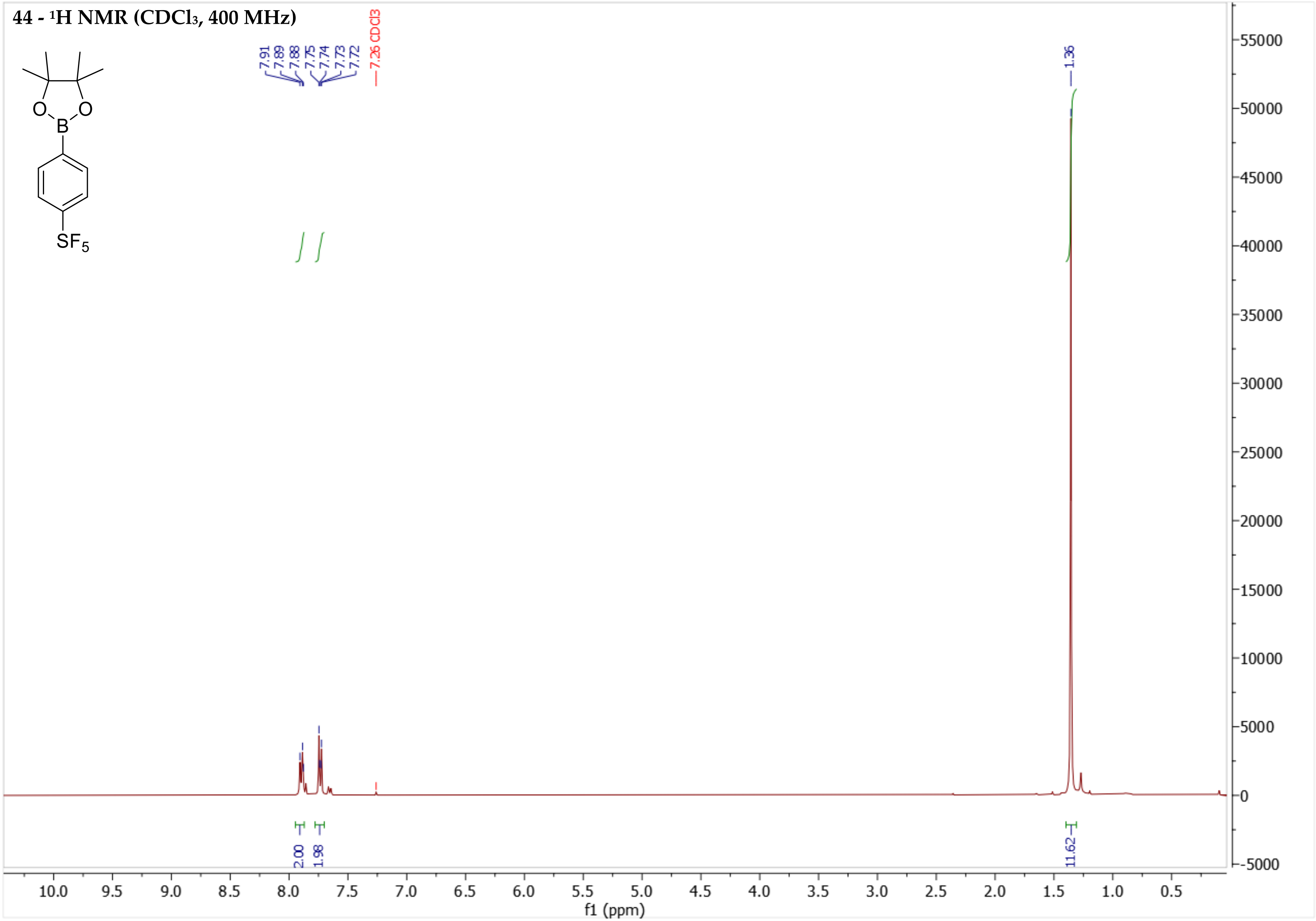

44 - $^{13}C\{^{1}H\}$ NMR ($CDCl_3$, 101 MHz)

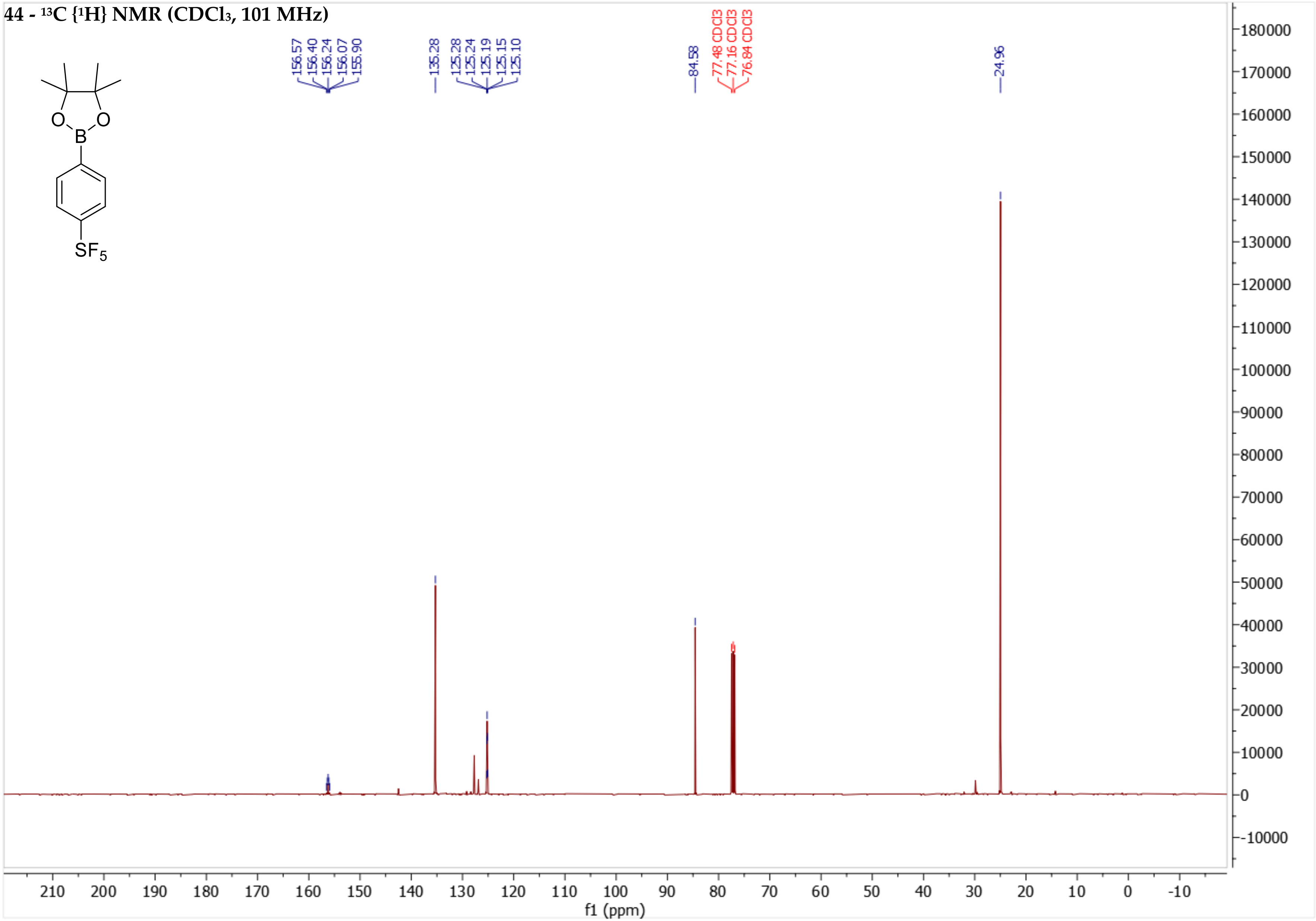

44 – ¹¹B NMR ($CDCl_3$, 128 MHz)

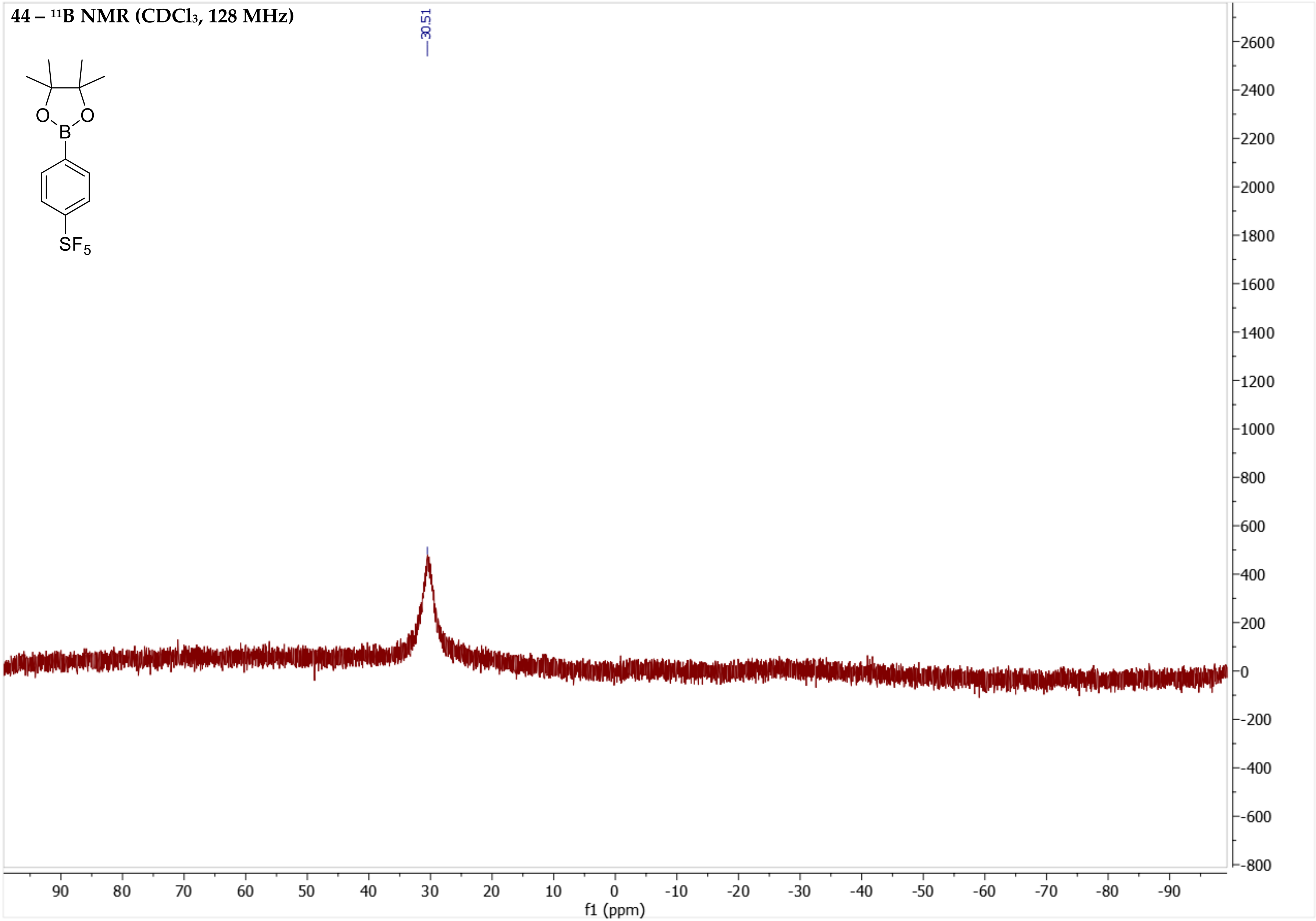

44 - ^{19}F NMR ($CDCl_3$, 377 MHz)

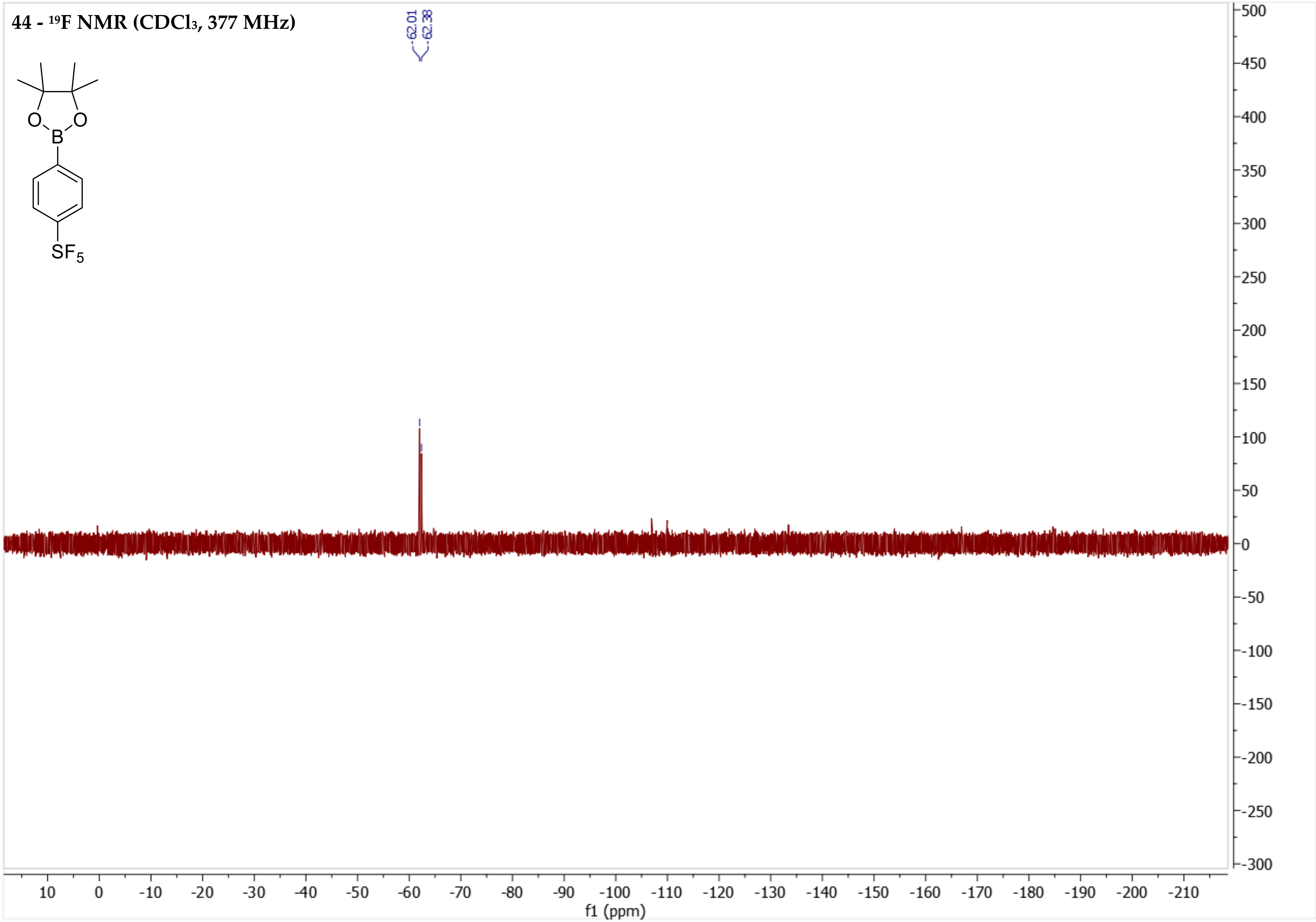

b-48 - ¹H NMR ($CDCl_3$, 400 MHz)

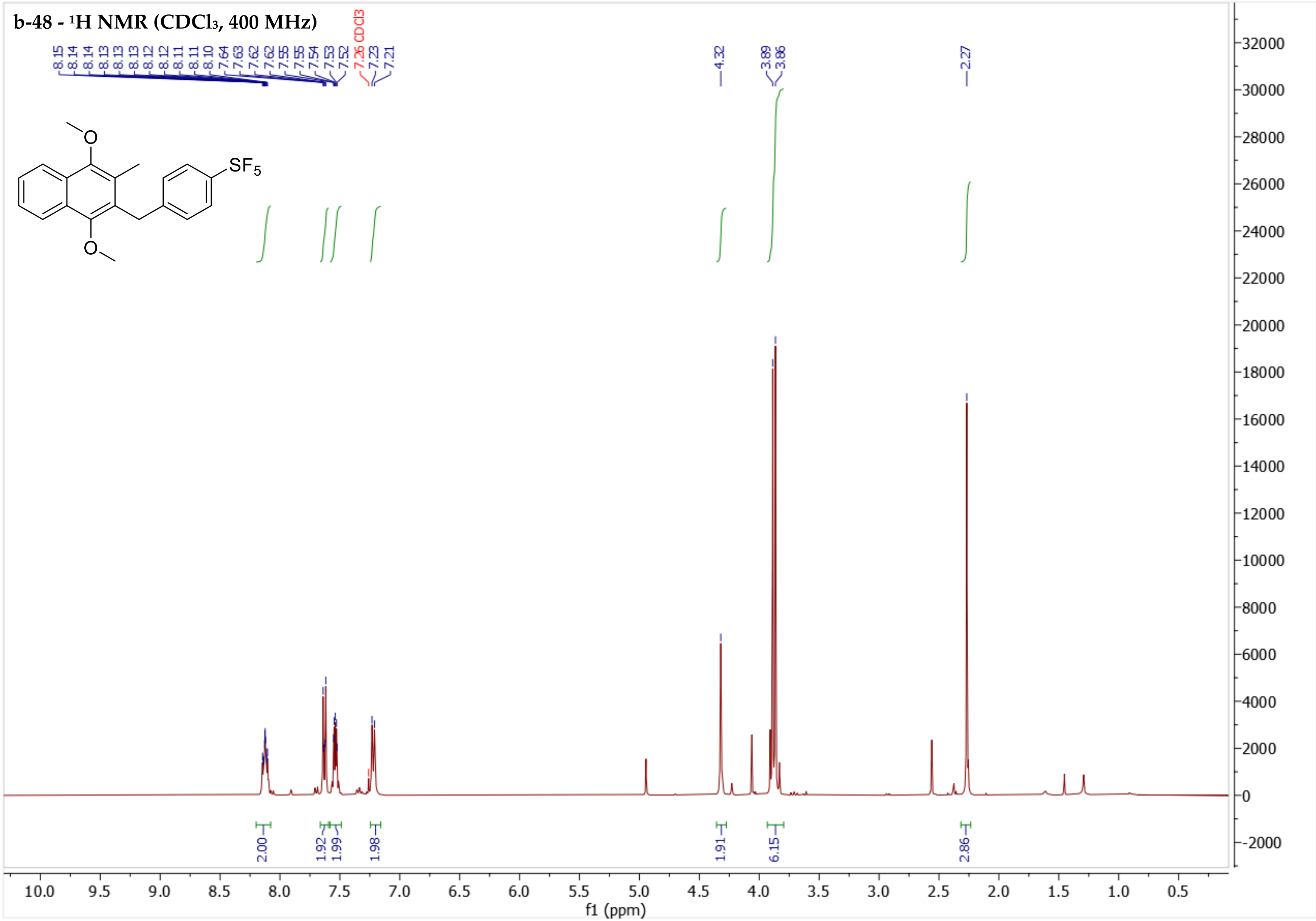

b-48 - $^{13}C\{^{1}H\}$ NMR ($CDCl_3$, 101 MHz)

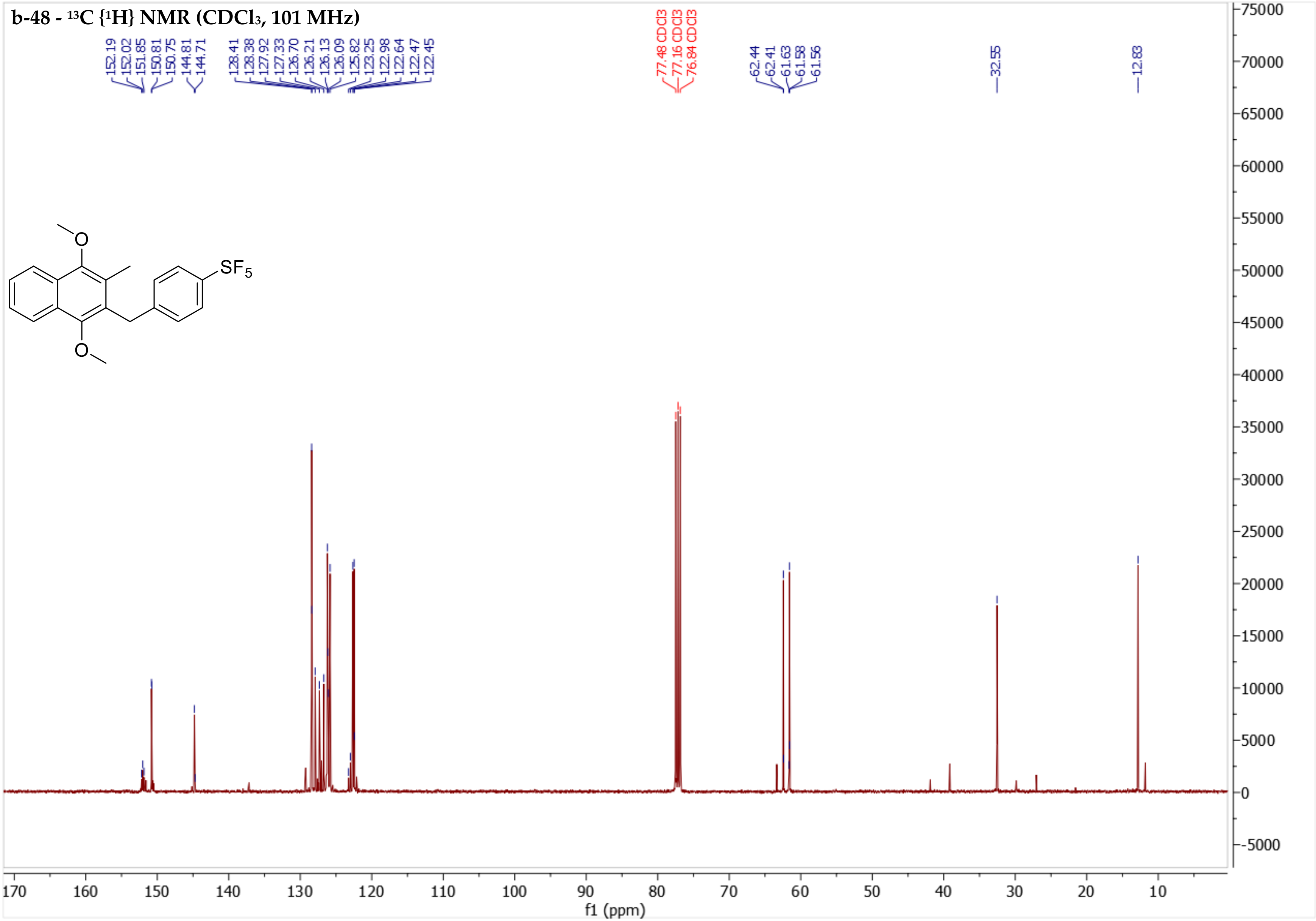

b-48 - ^{19}F NMR ($CDCl_3$, 377 MHz)

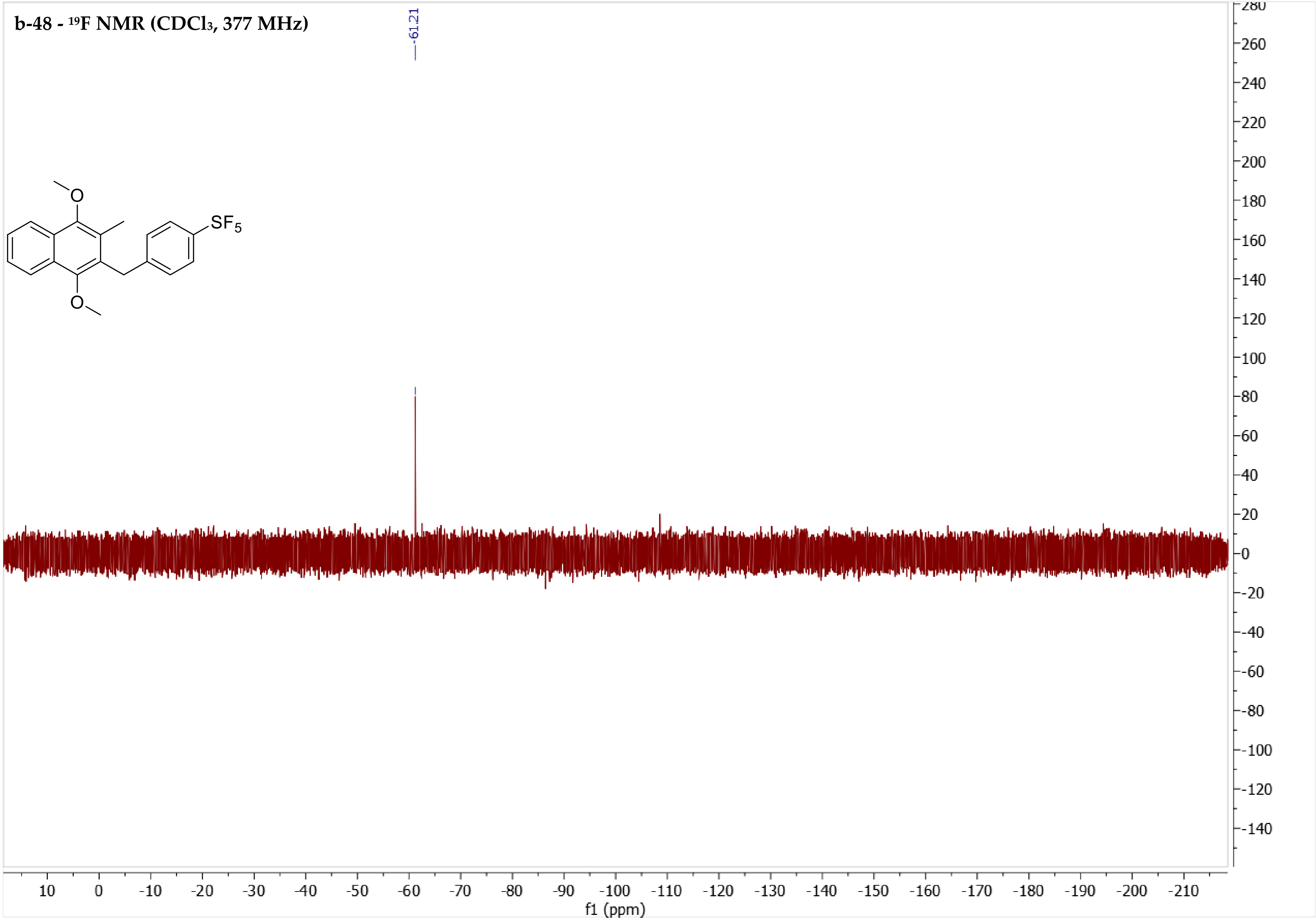

C-b-49 - ^{1}H NMR ($CDCl_3$, 400 MHz)

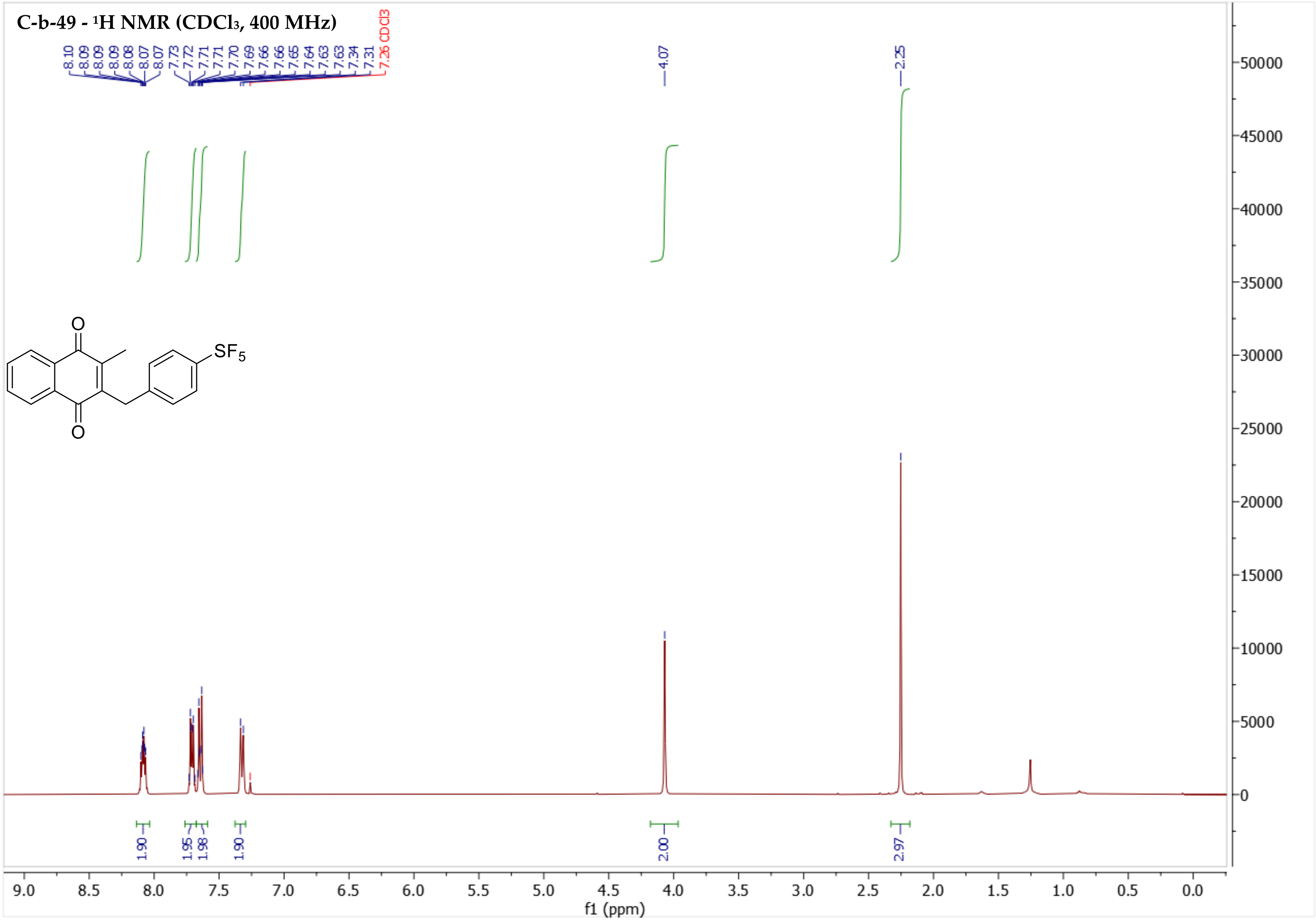

C-b-49 - $^{13}C\{^{1}H\}$ NMR ($CDCl_3$, 101 MHz)

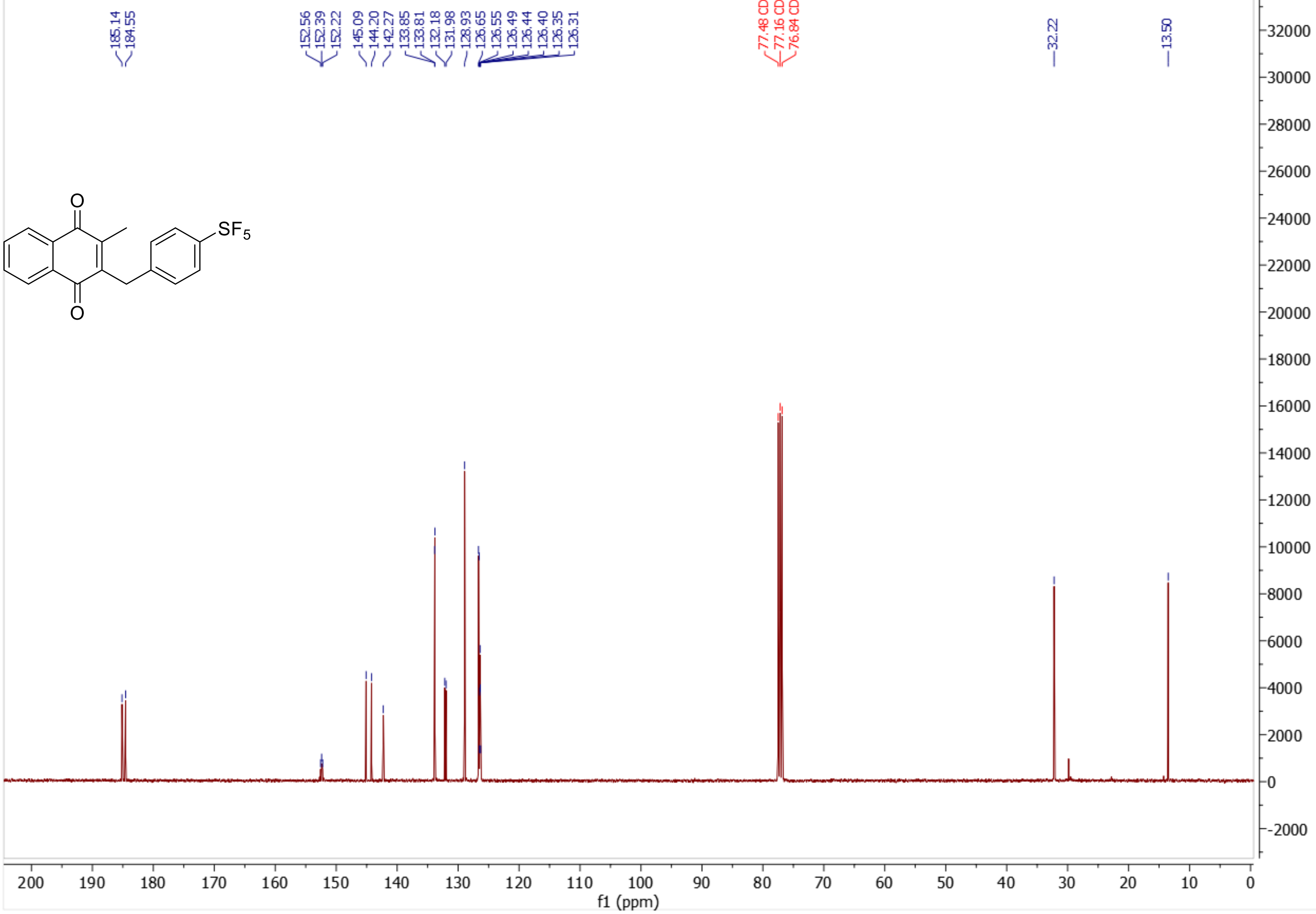

C-b-49 - ^{19}F NMR ($CDCl_3$, 377 MHz)

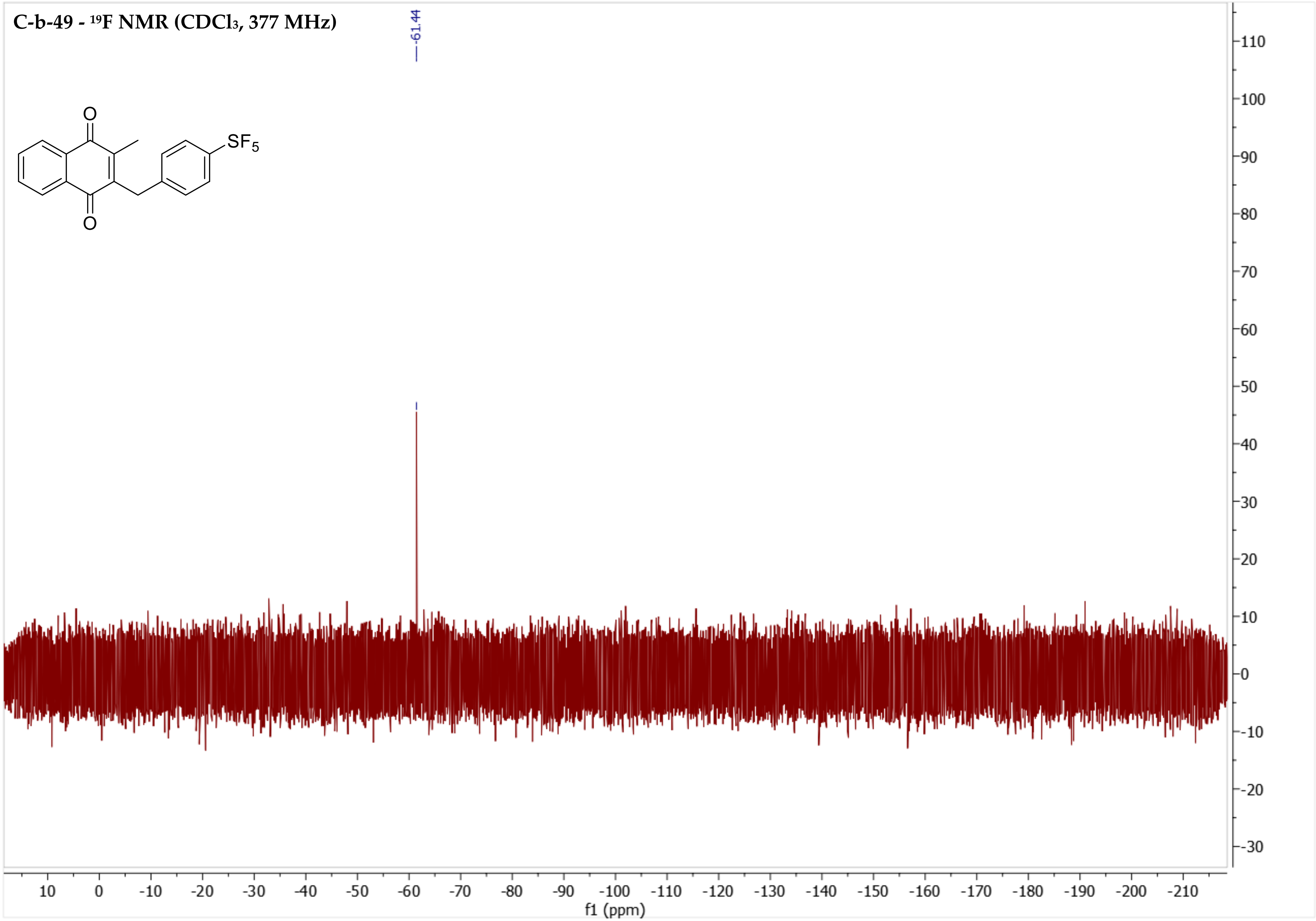

Supplement: Supplementary file 1 [file molecules-30-02446-s001.zip › molecules-3603660-supplementary.pdf]
